# Itaconate-producing neutrophils regulate local and systemic inflammation following trauma

**DOI:** 10.1172/jci.insight.169208

**Published:** 2023-10-23

**Authors:** Janna L. Crossley, Sonya Ostashevskaya-Gohstand, Stefano Comazzetto, Jessica S. Hook, Lei Guo, Neda Vishlaghi, Conan Juan, Lin Xu, Alexander R. Horswill, Gerta Hoxhaj, Jessica G. Moreland, Robert J. Tower, Benjamin Levi

**Affiliations:** 1 Department of Surgery,; 2 Children’s Research Institute and Department of Pediatrics,; 3 Department of Pediatrics, and; 4 Quantitative Biomedical Research Center, Peter O’Donnell Jr. School of Public Health, UT Southwestern Medical Center, Dallas, Texas, USA.; 5 Department of Immunology and Microbiology, University of Colorado School of Medicine, Aurora, Colorado, USA.

**Keywords:** Immunology, Inflammation, Neutrophils

## Abstract

Modulation of the immune response to initiate and halt the inflammatory process occurs both at the site of injury as well as systemically. Due to the evolving role of cellular metabolism in regulating cell fate and function, tendon injuries that undergo normal and aberrant repair were evaluated by metabolic profiling to determine its impact on healing outcomes. Metabolomics revealed an increasing abundance of the immunomodulatory metabolite itaconate within the injury site. Subsequent single-cell RNA-Seq and molecular and metabolomic validation identified a highly mature neutrophil subtype, not macrophages, as the primary producers of itaconate following trauma. These mature itaconate-producing neutrophils were highly inflammatory, producing cytokines that promote local injury fibrosis before cycling back to the bone marrow. In the bone marrow, itaconate was shown to alter hematopoiesis, skewing progenitor cells down myeloid lineages, thereby regulating systemic inflammation. Therapeutically, exogenous itaconate was found to reduce injury-site inflammation, promoting tenogenic differentiation and impairing aberrant vascularization with disease-ameliorating effects. These results present an intriguing role for cycling neutrophils as a sensor of inflammation induced by injury — potentially regulating immune cell production in the bone marrow through delivery of endogenously produced itaconate — and demonstrate a therapeutic potential for exogenous itaconate following tendon injury

## Graphical abstract

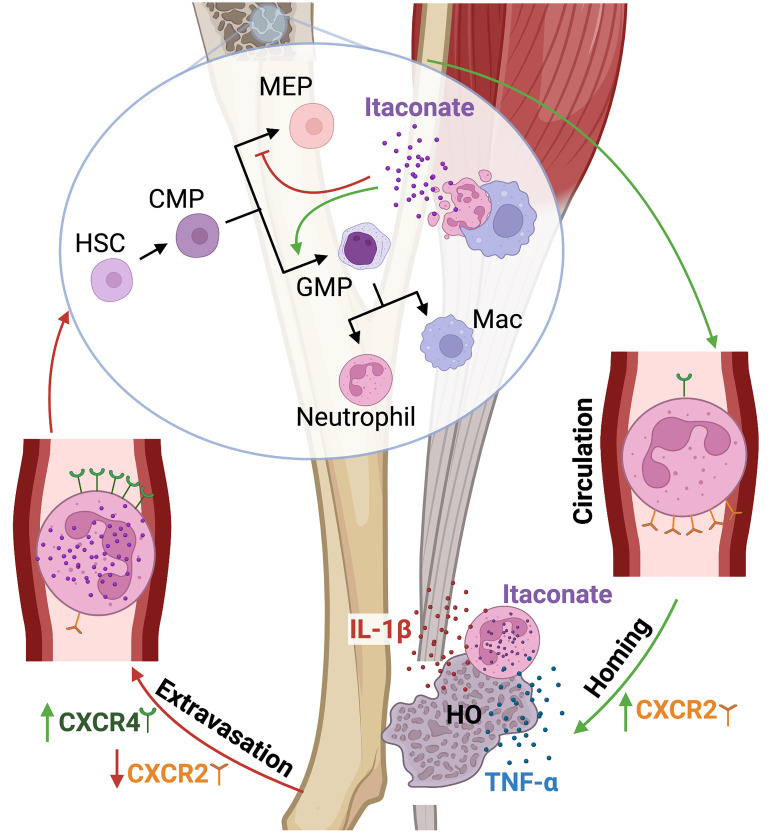

## Introduction

Inflammation is a tightly coordinated response by the body’s immune system as a reaction to harmful stimuli such as injury and infection (1). During the acute inflammatory phase, cellular components of the innate immune system home to the source of stimuli through the local production of cytokines, preventing the establishment or spread of infection. Similar to infection, trauma-induced injuries result in activation of the early innate immune response, which stimulates wound healing and repair (2). However, excessive or prolonged inflammation after injury can lead to aberrant progenitor cell fate determination and fibrosis (3). In the case of severe musculoskeletal soft-tissue injury, this can result in the formation of heterotopic ossification (HO), the building of extra skeletal bone in the surrounding soft tissue. HO is seen in nearly 20% of patients following high-energy extremity trauma, severe burns, or routine total hip arthroplasty (4, 5). Formation of HO causes chronic pain, reduces range of motion, and is often treated through surgical resection, which is associated with a high likelihood of relapse (6, 7). HO and similar fibrotic processes caused by prolonged inflammation underscore the need to regulate inflammation, both spatially and temporally, to allow for restoration of tissue back to homeostasis without abnormal effects induced by prolonged inflammatory stimulation.

One of the primary components of the innate immune system are neutrophils. While studied thoroughly in their role as the first responder to injury and infection, less is known about their role as modulators of the systemic immune response. Neutrophils are granulocytic cells derived from bone marrow hematopoietic stem cells (HSCs) that undergo stepwise differentiation from common myeloid progenitors (CMPs) to granulocyte-monocyte progenitors (GMPs) and finally granulocyte progenitors (GPs) before becoming an immature form of neutrophil present within the marrow cavity. These immature neutrophils undergo a maturation process, associated with increasing nuclear complexity, alternating expression of receptors and inflammatory ligand production (8, 9). Neutrophils derived from the bone marrow enter circulation and track to the sites of inflammation via expression of CXC motif chemokine receptor 2 (CXCR2). There, they serve as a first line of defense against potential infection, as well as support the clearance of dead tissue (10). The past decade has revealed that neutrophils possess more than antimicrobial function, existing in unique subsets across homeostatic and pathological conditions (8, 11). Though previously thought to terminate within the inflamed or injured tissue, neutrophils have garnered increased interest following the discovery that they undergo a short, cyclic lifespan that sees them extravasate from the injury site and cycle back to the bone marrow via a CXCL12/CXCR4 signaling axis where they are turned over by bone marrow macrophages (12, 13). While the exact purpose of this cycling is not entirely understood, recent work suggests that this marrow-mediated neutrophil turnover could regulate HSC fate and function in a circadian rhythm–dependent manner (14, 15). Furthermore, disruption in bone marrow macrophage clearance of neutrophils in aging has been shown to result in hematopoietic skewing toward the megakaryocyte lineage due to increased macrophage IL-1β secretion (16). However, the function of neutrophil turnover in the bone marrow and how this could regulate systemic inflammation following inflammatory stimulation remains unknown.

In this study, we metabolically profiled injury sites in models marked by a normal and aberrant immune response. Specifically, we analyzed the Achilles tendon following HO- and non-HO–inducing musculoskeletal injury. These studies identified itaconate as a differentially abundant metabolite present within our model of aberrant inflammation and HO formation. Contrary to previous literature, our data suggest that mature neutrophils, not macrophages, are the primary producers of itaconate within the tendon injury area. Subsequent sequencing and flow analyses suggest that, when present within the local injury environment, these mature neutrophil subtypes are highly inflammatory, secreting cytokines to perpetuate the prolonged inflammatory local injury environment essential for aberrant differentiation and HO formation. In contrast, itaconate was also found to regulate systemic inflammation through modulation of hematopoiesis, favoring granulocytic and myeloid lineages at the expense of erythrocyte commitment. Finally, exogenous administration of itaconate was found to have a therapeutic effect, mitigating HO formation through its immunomodulatory function.

## Results

### Metabolic profiling identifies itaconate production following burn/tenotomy of the Achilles tendon.

**Figure 1:**
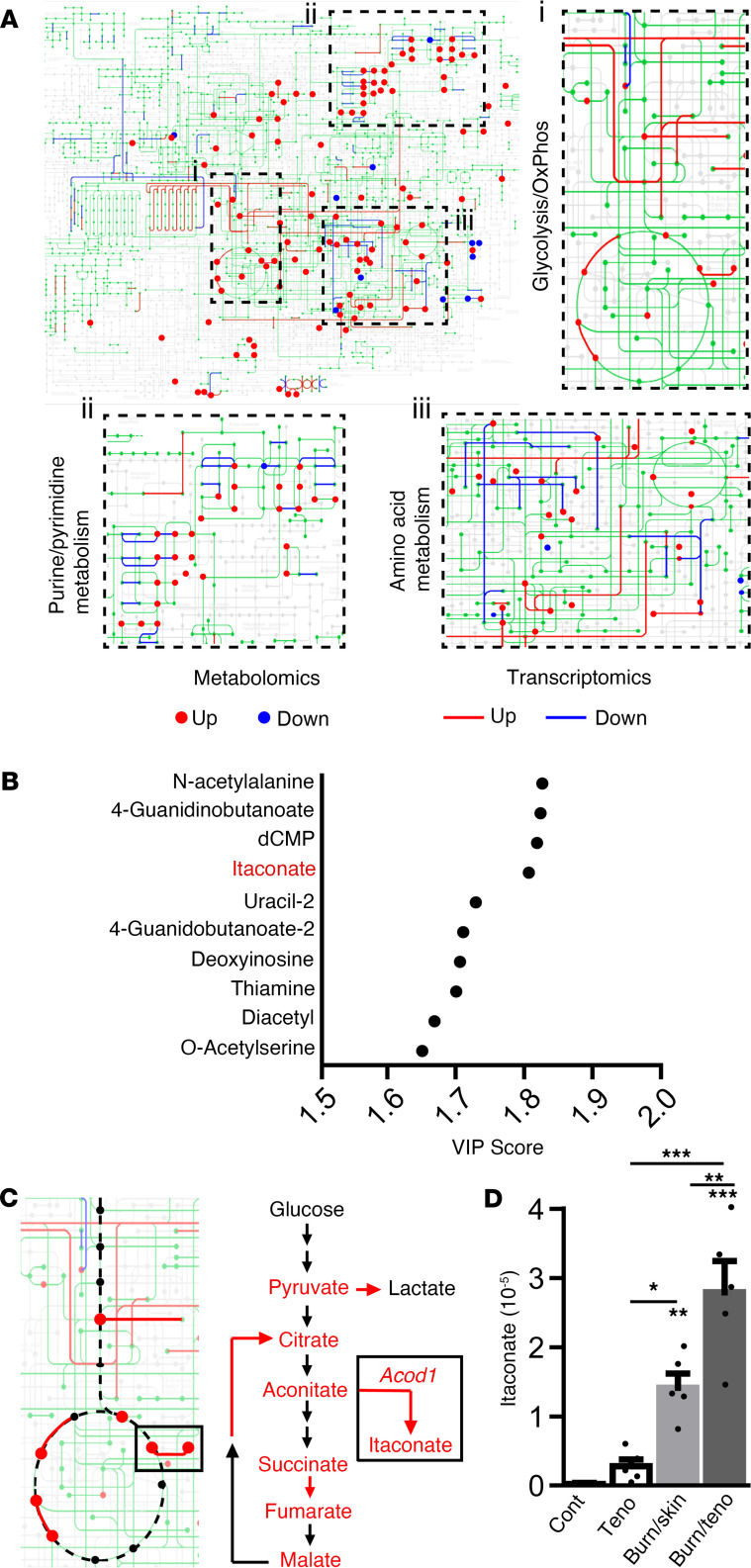
Metabolic analysis shows itaconate accumulation following burn/tenotomy. (**A**) Metabolomics (dots) and transcriptomic (22) (lines) analysis conducted on the tendon injury site mapped on to “Metabolic Pathways” KEGG schematic (green). Colors denote increased (red) and decreased (blue) abundance in injury sites 7 days after burn/tenotomy relative to uninjured controls. Highly regulated regions, including glycolysis/OxPhos (region i), purine/pyrimidine metabolism (region ii), and amino acid metabolism (region iii), are expanded for clarity. (**B**) Top 10 differentially enriched metabolites within the Achilles tendon region from uninjured control, tenotomy, burn/skin incision, and burn/tenotomy. (**C**) Glycolysis/TCA cycle pathways with relevant components shown on right. Red denotes increased metabolites (text) and enzyme expression (arrows). (**D**) Itaconate abundance within each injury condition. *n* = 5. Data are shown as mean ± SD. *P < 0.05, **P < 0.01, ***P < 0.001, determined by 2-way ANOVA followed by Tukey’s multiple-comparison test, relative to uninjured controls unless otherwise noted.

Recent work has demonstrated that metabolism plays a critical role in cell expansion, activity, and fate determination of musculoskeletal tissue under physiological and injury conditions (17–20). To understand the role metabolic reprogramming may play within the tendon in response to trauma, the Achilles tendon region was isolated from uninjured controls, as well as the entire tendon injury area, in mice 7 days following burn/tenotomy. This injury consists of a burn applied to the back of the mouse covering ~30% of the body surface to induce systemic inflammation in addition to a full transection of the Achilles tendon, which results in 100% of mice developing HO by 9 weeks through an endochondral process (21). The resulting differentially abundant metabolites were visualized by mapping them to the mouse “metabolic pathways” reference KEGG pathway (Figure 1A, dots). Additionally, these metabolic data were combined with previously published transcriptional data (22), similarly comparing the Achilles tendon between day 7 burn/tenotomy mice and uninjured controls (Figure 1A, lines). Visual analysis revealed 3 “clusters” of differentially regulated metabolites/transcripts, including glycolysis/oxidative phosphorylation (OxPhos) (Figure 1A, region i), purine/pyrimidine metabolism (Figure 1A, region ii), and amino acid metabolism (Figure 1A, region iii).

To further distinguish changes in metabolism resulting from inflammatory induction or injury, we conducted additional metabolomic analyses on mice 7 days after tenotomy (trauma only) or burn/skin incision (inflammation only), neither of which has previously been reported to form HO. Analyses revealed that both trauma and burn/skin incision–induced inflammation were sufficient to induce the increased presence of metabolic intermediates linked to glycolysis (Supplemental Figure 1A; supplemental material available online with this article; https://doi.org/10.1172/jci.insight.169208DS1) or OxPhos (Supplemental Figure 1B). In addition to increased bioenergetic metabolites, several metabolites involved in the ornithine cycle or glutathione production were additionally found to be abundantly present within the tendon injury site (Supplemental Figure 1C). Analysis of the top 10 differentially regulated metabolites across our 4 injury models identified itaconate (Figure 1B), a metabolite with immunomodulatory properties (23) derived from the tricarboxylic acid (TCA) cycle intermediate aconitate through the enzymatic function of aconitate decarboxylase 1 encoded by the gene *Acod1* (also known as *Irg1*) (Figure 1C). Evaluation of itaconate across our 4 conditions showed a modest increase following tenotomy, a more substantial, significant increase following burn/skin incision, and a synergistic significant increase in our HO-forming burn/tenotomy model (Figure 1D). These data suggest that the combination of trauma and inflammation results in significantly increased abundance of itaconate within the tendon injury area.

### Endogenous itaconate is produced by highly mature neutrophils within the tendon injury site.

**Figure 2:**
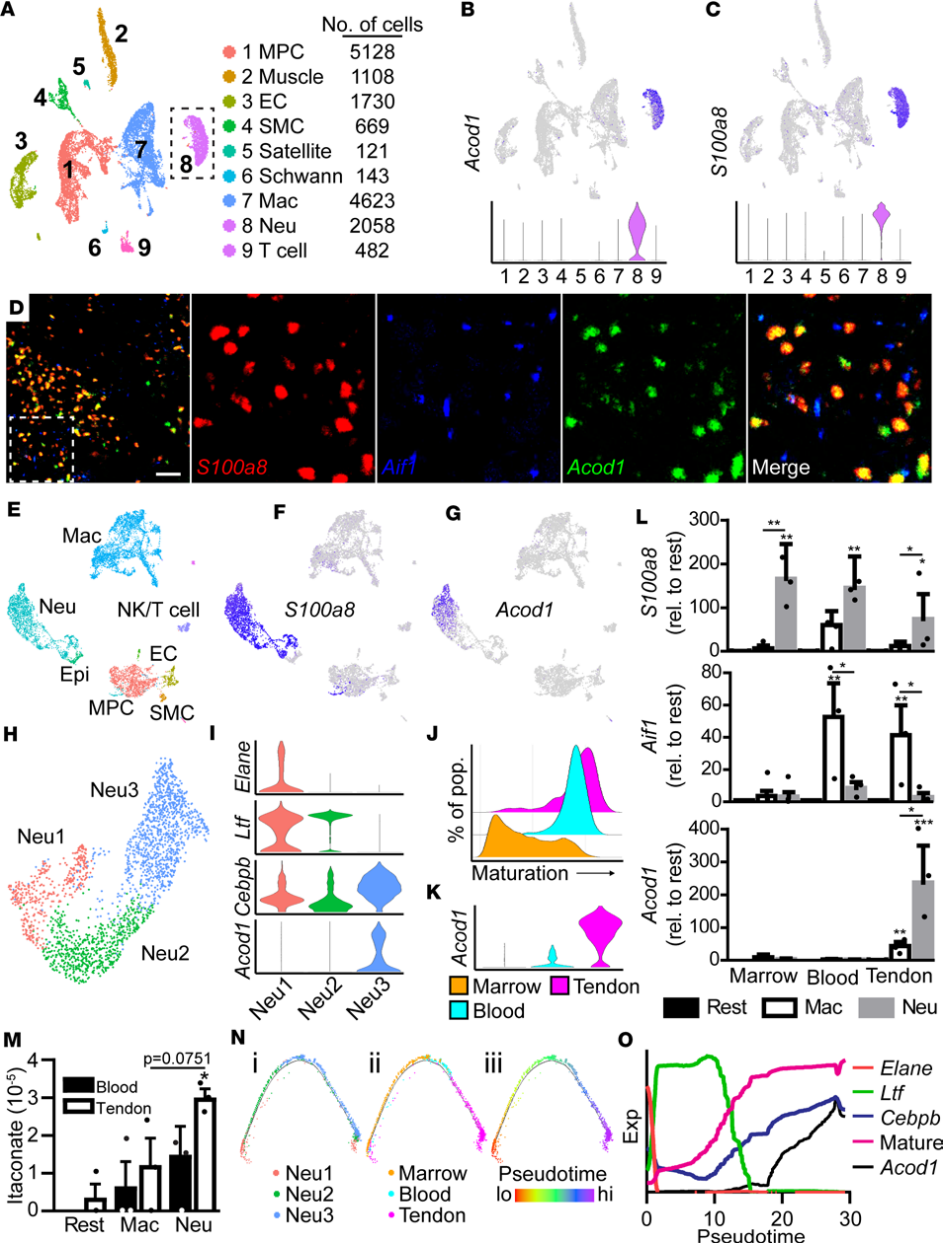
Itaconate is produced by a subset of highly mature neutrophils within the HO injury site. (**A**) UMAP of scRNA-Seq data obtained from the tendon injury area 7 days after burn/tenotomy (62). Dashed box denotes neutrophil cluster. (**B**) Feature plot of *Acod1* expression. (**C**) Feature plot of the neutrophil marker *S100a8*. (**D**) RNAscope of the injured tendon region stained for *Acod1*, as well as for neutrophils (*S100a8*) and macrophages (*Aif1*). Scale bar: 50 µm. (**E**) UMAP of scRNA-Seq data obtained from the bone marrow, blood, and tendon injury site after burn/tenotomy surgery. (**F**) Feature plot of the neutrophil marker *S100a8*. (**G**). Feature plot of *Acod1*. (**H**) UMAP of subclustered neutrophils. (**I**) Expression of *Acod1* and markers of early (*Elane*), middle (*Ltf*), and late-stage (*Cebpb*) neutrophils. (**J**) Ridge plot of neutrophil maturation clustered by physiological site of origin. (**K**) Expression of *Acod1* in neutrophils clustered by site of origin. (**L**) Expression of *Acod1* as well as neutrophil (*S100a8*) and macrophage (*Aif1*) markers in cells fractionated into neutrophils, macrophages, and “rest” from the bone marrow, blood, and tendon injury site. *n* = 4/site. (**M**) Levels of itaconate in neutrophils, macrophages, and “rest” isolated from the blood or tendon injury site. (**N**) Trajectory analysis of neutrophils colored by neutrophil subcluster (region i), site of origin (region ii), or pseudotime (region iii). (**O**) Expression pattern of early (*Elane*), middle (*Ltf*), and late-stage (*Cebpb*) neutrophil markers, overall maturation score, and *Acod1* expression across neutrophil trajectory pseudotime. Data are shown as mean ± SD. *P < 0.05, **P < 0.01, ***P < 0.001, determined by 2-way ANOVA followed by Tukey’s multiple-comparison test, relative to tendon injury site “rest” unless otherwise noted.

Itaconate has previously been shown to be produced in macrophages in response to inflammatory induction, resulting in transcriptional shifts from a more proinflammatory (M1) to antiinflammatory (M2) state (23–25). Due to the previously published role of M2 macrophages in HO progression (3, 26), we further investigated whether this observed itaconate was produced by macrophages. To determine which cells may be responsible for this observed itaconate accumulation, we analyzed single-cell RNA-Seq (scRNA-Seq) data obtained from the entire injury site 7 days after burn/tenotomy surgery (22) (Figure 2A). Expression of *Acod1* was highly restricted to a single cell cluster (Figure 2B). Surprisingly, this cluster was marked by robust expression of the neutrophil marker *S100a8* (Figure 2C). scRNA-Seq requires the use of enzymatic digestion to generate single-cell suspensions. It is possible that the sample preparation procedure may have induced *Acod1* expression. To exclude this possibility, RNAscope was conducted on the injured tendon. Imaging showed that *S100a8*-expressing neutrophils and, to a much lesser extent, *Aif1*-expressing macrophages — were the primary expressors of *Acod1* (Figure 2D). These combined data suggest that neutrophils, not macrophages, may be the primary source of itaconate within the injury site following HO-forming trauma.

In response to inflammatory induction, neutrophils undergo a progressive maturation process (9). To track this maturation, scRNA-Seq was conducted on unsorted cells derived from the bone marrow, blood, and tendon injury site 3 days after burn/tenotomy surgery and integrated into a single object (Figure 2E and Supplemental Figure 2). Neutrophils across all locations were identified by, among other markers (Supplemental Table 1), robust and specific expression of *S100a8* (Figure 2F). Interestingly, while the tendon injury site scRNA-Seq suggested nearly uniform expression across all neutrophils (Figure 2B), this multisite merged scRNA-Seq data suggest that only a subset of neutrophils expressed *Acod1* (Figure 2G). To further elucidate *Acod1* expression across different neutrophil subpopulations, neutrophils were reanalyzed into 3 distinct subsets (Figure 2H and Supplemental Table 2), clustering into early- (Neu1, expressing *Elane*), middle- (Neu2, expressing *Ltf*), and late-stage (Neu3, expressing *Cebpb*) subtypes (Figure 2, H and I). Analysis of *Acod1* showed almost exclusive expression within this late-stage Neu3 population (Figure 2I). These data suggest that neutrophils, not macrophages, are the primary expressors of *Acod1* following musculoskeletal trauma and that this *Acod1* expression is highly restricted to mature, late-stage neutrophils.

To better characterize where these late-stage neutrophils are present, neutrophils were clustered by site of origin and analyzed using a module score for genes previously linked to neutrophil maturation (9) (Figure 2J and Supplemental Table 3). Consistent with the known derivation of mature circulating neutrophils from bone marrow HSCs, bone marrow neutrophils showed the lowest levels of maturation, with increasing maturation scores linked to declines in bone marrow neutrophil populations and a concomitant increase in more mature blood neutrophils (Figure 2J). Interestingly, though the dorsal burn component of our burn/tenotomy surgery is known to induce systemic inflammation, neutrophils within the HO-forming tendon injury site showed a subtle, yet notable, shift in maturation compared with neutrophils within the blood, suggesting the presence of a highly mature form of neutrophil present within the injury environment. Similar to our neutrophil subclustering results (Figure 2I), *Acod1* was expressed primarily within HO-forming neutrophils, with only minimal *Acod1* expression observed in blood neutrophils and no notable expression within the bone marrow (Figure 2K). This preferential expression of *Acod1* in HO-site neutrophils was confirmed by fractionation of the bone marrow, blood, and tendon injury site into neutrophils, macrophages, and the remaining flow through (mesenchymal cells, vascular cells, other immune cells, etc., here referred to as “rest”). Marker genes for neutrophils (*S100a8*) and macrophages (*Aif1*) confirmed successful isolation of desired cell types (Figure 2L). While tendon injury site macrophages showed a significant increase in *Acod1* expression as compared with “rest,” this increase was dwarfed by the drastic enrichment of *Acod1* expressed within tendon injury-site neutrophils as assessed by quantitative PCR (qPCR) (Figure 2L). Similarly, while no notable levels of itaconate were detected prior to injury, metabolomic analyses revealed that neutrophils contained the greatest levels of itaconate within the tendon injury site and, to a lesser extent, within the blood 3 days following the burn/tenotomy injury (Figure 2M). Finally, trajectory analysis was conducted on subclustered neutrophil scRNA-Seq data (Figure 2N), and it confirmed a progressive maturation of neutrophils and increasing expression of *Acod1* along the pseudotemporal axis (Figure 2O). To confirm that this finding was not restricted to our HO model of burn/tenotomy, we performed analyses of published scRNA-Seq data from other musculoskeletal injuries such as calvarial defect and volumetric muscle loss. These analyses confirmed similar findings, with *Acod1* expression highly selective for mature *Cebpb*-expressing neutrophils following calvarial defect (27) or volumetric muscle loss (28) (Supplemental Figure 3). Taken together, these data show that *Acod1* is expressed by a highly mature neutrophil subtype uniquely present within the tendon injury site.

### Prolonged retention of mature neutrophils at the tendon injury site contributes to continued local inflammation.

**Figure 3:**
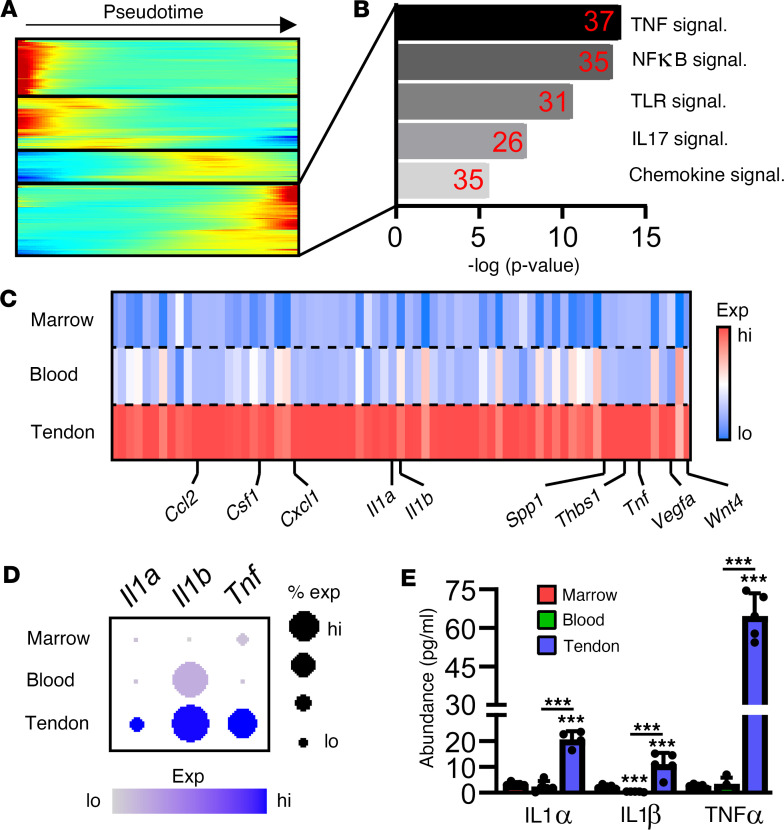
Mature neutrophils within the HO-forming injury site contribute to prolonged local inflammation. (**A**) Expression profile of genes differentially expressed across neutrophil pseudotime. (**B**) Pathway analysis of pseudotime DEGs enriched with the terminal neutrophil stage. Red numbers denote number of differentially expressed genes found within each pathway. (**C**) Heatmap showing expression of terminal neutrophil genes by pseudotime, clustered by site of origin. (**D**) Expression of indicated genes within neutrophils derived from different sites. (**E**) Indicated protein abundance within the bone marrow, blood, and HO site. *n* = 5. Data are shown as mean ± SD. ***P < 0.001, determined by 1-way ANOVA followed by Tukey’s multiple-comparison test, relative to bone marrow unless otherwise noted.

To further characterize the phenotype of these highly mature neutrophils enriched in the tendon injury site, we conducted cell-to-cell communication (CCC) analyses between neutrophils in the bone marrow, blood, and tendon injury site (Supplemental Figure 4). These analyses identify predicted ligand-receptor signaling pairs between cell types at each of our physiological conditions. Both overall cell-to-cell interactions (Supplemental Figure 4A) as well as neutrophil specific interactions (Supplemental Figure 4, B and C) were greatest within the context of the tendon injury site. Looking at downstream targets of these proposed ligand-receptor interactions (Supplemental Figure 4D) identified pathways commonly regulated, though to varying degrees, across all physiological locations (Supplemental Figure 4, E and F, and Supplemental Table 4) as well as pathways specifically regulated within the tendon injury site only (Supplemental Figure 4, G and H). To further characterize these highly mature injury-site neutrophils, we clustered genes differentially expressed across the neutrophil pseudotime maturation axis (Figure 3A). Pathway analysis of genes enriched within this highly mature neutrophil subtype showed enrichment in several canonical inflammatory cascades (Figure 3B). Consistent with the tendon injury site primarily associated with mature neutrophils, late pseudotime genes heavily enriched within injury-site neutrophils, including high levels of inflammatory cytokines (*Ccl2, Csf1, Cxcl1, Il1a, Il1b, Tnf*) as well as factors that promote angiogenesis (*Vegfa*) or chondro/osteogenesis (*Spp1, Thbs2, Wnt4*) (Figure 3C). Robust and prolonged inflammation is a hallmark of HO formation and progression. Expression of the inflammatory cytokines *Il1a*, *Il1b*, and *Tnf* were elevated in injury-site neutrophils (Figure 3D), likely contributing to overall increases in levels of IL-1α/β and TNF-α present within the tendon injury site (Figure 3E). These findings of high *Il1b* and *Tnf* in itaconate-producing neutrophils are contradictory to results previously published in macrophages that demonstrate that itaconate production inhibits expression of these proinflammatory cytokines (24, 25, 29). These data suggest that these highly mature neutrophils do not respond transcriptionally as has previously been described for endogenously produced itaconate in macrophages, instead expressing high levels of cytokines to regulate the local inflammatory environment.

### Itaconate-loaded neutrophils are cycled back to the bone marrow for degradation by macrophages.

**Figure 4:**
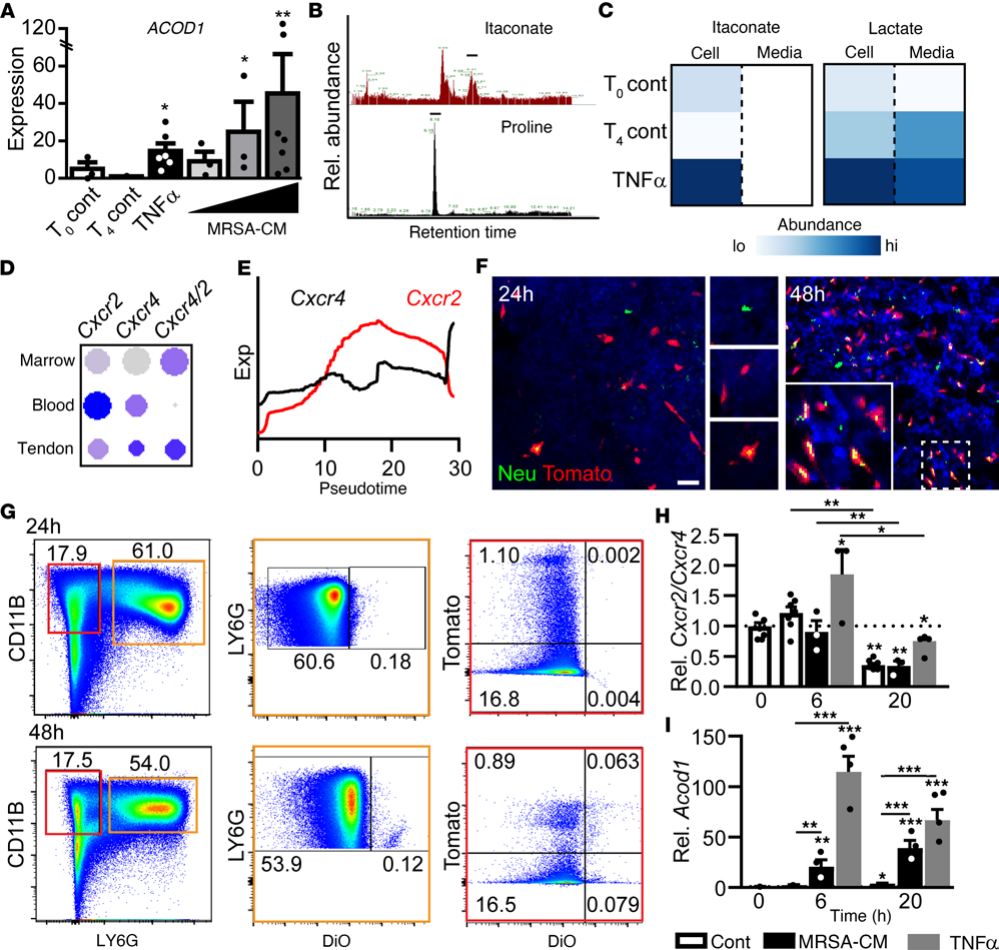
Itaconate produced by stimulated neutrophils is delivered to the bone marrow. (**A**) Expression of *ACOD1* in human neutrophils isolated from peripheral blood and stimulated with TNF-α or varying doses of a cell membrane preparation from *S*. *aureus* (MRSA-CM). (**B**) Metabolomic profiling of itaconate in stimulated neutrophils. Proline is shown as a control. (**C**) Itaconate and lactate levels in cell bodies and conditioned media from freshly isolated, unstimulated, and TNF-α–stimulated human neutrophils. (**D**) Dot plot of *Cxcr2* and *Cxcr4* across different anatomical sites. (**E**) Expression of *Cxcr2* and *Cxcr4* across neutrophil pseudotime. (**F**) Confocal image of bone marrow from *Cd169/Tomato* mice showing injury-site neutrophils harvested from donor mice and dyed using a membrane label following i.v. injection (green) and Tomato^+^ macrophages (red) at indicated time points after injection. Scale bar: 25 μm. (**G**) Flow analysis of neutrophils and monocytes/macrophages from *Cd169/Tomato* mouse bone marrow injected i.v. with membrane-labeled neutrophils at indicated time points after injection. (**H**) *Cxcr2/Cxcr4* ratio assessed by qPCR of neutrophils in vitro cultured with or without stimulation with TNF-α or MRSA-CM. Dotted line denotes a 1:1 ratio of *Cxcr2* and *Cxcr4* transcripts. (**I**) Relative expression of *Acod1* assessed by qPCR in cultured neutrophils with or without TNF-α or MRSA-CM stimulation. Data are shown as mean ± SD. *P < 0.05, **P < 0.01, ***P < 0.001, determined by 2-way ANOVA followed by Tukey’s multiple-comparison test, relative to time 4 hours after stimulation (T_4_) unstimulated controls (**A**) or t_0_ (**H** and **I**) unless otherwise stated.

This observed highly inflammatory nature of injury-site neutrophils is somewhat in contrast to the specific production of itaconate, a proposed antiinflammatory metabolite (23), by these same highly mature neutrophil populations. To understand whether neutrophil-derived itaconate was secreted into the injury site as a mechanism to further regulate the local inflammatory milieu, human neutrophils were incubated with inflammatory agonists. qPCR analysis showed significant upregulation of *ACOD1* in response to stimulation with the inflammatory cytokine TNF-α to mimic trauma as well as in response to a cell membrane prep from a virulent, methicillin-resistant *S*. *aureus* (MRSA-CM) to mimic infection consistent with recently published results (30) (Figure 4A). Metabolomic analysis of TNF-α–stimulated human neutrophils revealed that, while itaconate was readily detected within stimulated neutrophil cell bodies, no itaconate was found within the conditioned media (Figure 4, B and C). In contrast, lactate was easily detected within the conditioned media (Figure 4C). This suggests that, while inflammatory- or infection-stimulated neutrophils may produce itaconate, this metabolite remains intracellular and may not be secreted as a mechanism to regulate the local injury environment.

If not secreted to regulate local inflammation, we next sought to understand the terminal function of this injury-site neutrophil–derived itaconate. When nearing the end of their lifespan, neutrophils typically cycle back to the bone marrow for turnover through the concomitant downregulation of CXCR2 and upregulation of CXCR4 (13). While some neutrophils are likely to terminate within the injury tissue through the formation of neutrophil extracellular traps (NETs) (31, 32), a portion of neutrophils may cycle back to the bone marrow following homing to sites of injury. Expression analysis of *Cxcr2* and *Cxcr4* in our neutrophil population from the marrow, blood, and injury site showed the expected changes in receptor expression (Figure 4D). This included release from the bone marrow being associated with a high *Cxcr2/Cxcr4* ratio while terminal late-stage neutrophils were associated with a switch in expression resulting in a high *Cxcr4/Cxcr2* ratio (Figure 4, D and E). This differential expression coincided with expression of genes linked to CXCR2/CXCR4 cell surface abundance through gene expression or through receptor modification/inactivation or internalization (Supplemental Figure 5). Our analyses show a preference for CXCR4 in early-stage neutrophils (cluster 1) primarily present within the bone marrow and CXCR2 within the highly inflammatory cluster 3 (enriched in the tendon), and a similar expression profile across neutrophil maturation pseudotime as observed for *Cxcr2/4* gene expression (Supplemental Figure 5). This elevated CXCR4/CXCR2 ratio drives extravasation of neutrophils out of the inflamed tissue back to the bone marrow via CXCL12 (also known as stromal cell–derived factor 1 [SDF1]) (13). Next, neutrophil turnover in the bone marrow was monitored. Neutrophils were isolated from the tendon injury site, subjected to membrane staining and transplanted into *CD169-Cre;Rosa-tdTomato* (*CD169/Tomato*) recipient mice in which most tissue resident–like macrophages are labeled by Tomato expression (33, 34) (Figure 4F). Twenty-four hours after injection, membrane-labeled neutrophils were observed within the bone marrow, either alone or associated with Tomato^+^ macrophages. By 48 hours, the majority of the membrane dye was associated with Tomato^+^ macrophages, suggesting engulfment of neutrophils, either directly through efferocytosis or indirectly through phagocytosis of scavenged neutrophil cell membrane (Figure 4F). Flow analysis of recipient mice showed that between 24 and 48 hours after injection, transplanted neutrophils declined while Tomato^+^, and to a lesser extent Tomato^–^, monocyte/macrophages positive for neutrophil-derived membrane dye increased (Figure 4G). These data suggest that itaconate-loaded neutrophils, stimulated by inflammation or infection, cycle back to the bone marrow for destruction either directly by, or in close proximity to, macrophages.

While neutrophils at the end of their life cycle return to the bone marrow for destruction, our data suggest that neutrophils have unique molecular patterns depending on their exposure to inflammatory stimuli. To further confirm this, we next conducted in vitro maturation assays without stimulation or in response to treatment with either TNF-α or MHRSA-CM. Under both stimulated and unstimulated conditions, qPCR analysis of cultured mouse bone marrow neutrophils showed a significant switch in their receptor expression leading to decreased *Cxcr2/Cxcr4* ratios by 20 hours (Figure 4H). Interestingly, while a modest increase in *Acod1* expression was observed at 20 hours without stimulation, neutrophils stimulated with either TNF-α or MRSA-CM resulted in significantly elevated levels of *Acod1* at both 6 and 20 hours after stimulation (Figure 4I). These findings are consistent with reanalysis of neutrophils from multiple locations (35) and suggest that *Acod1* was primarily observed in mature, *Cebpb*-expressing neutrophils following systemic inflammatory stimulation, with minimal expression in neutrophils under unstimulated, physiological conditions (Supplemental Figure 6). Together, these data suggest that, while neutrophils typically cycle back to the bone marrow at the end of their lifespan, levels of itaconate that they deliver back to the bone marrow may reflect the level of inflammatory stimuli or tissue damage.

### Itaconate skews hematopoiesis toward the granulocyte/monocyte lineages.

**Figure 5:**
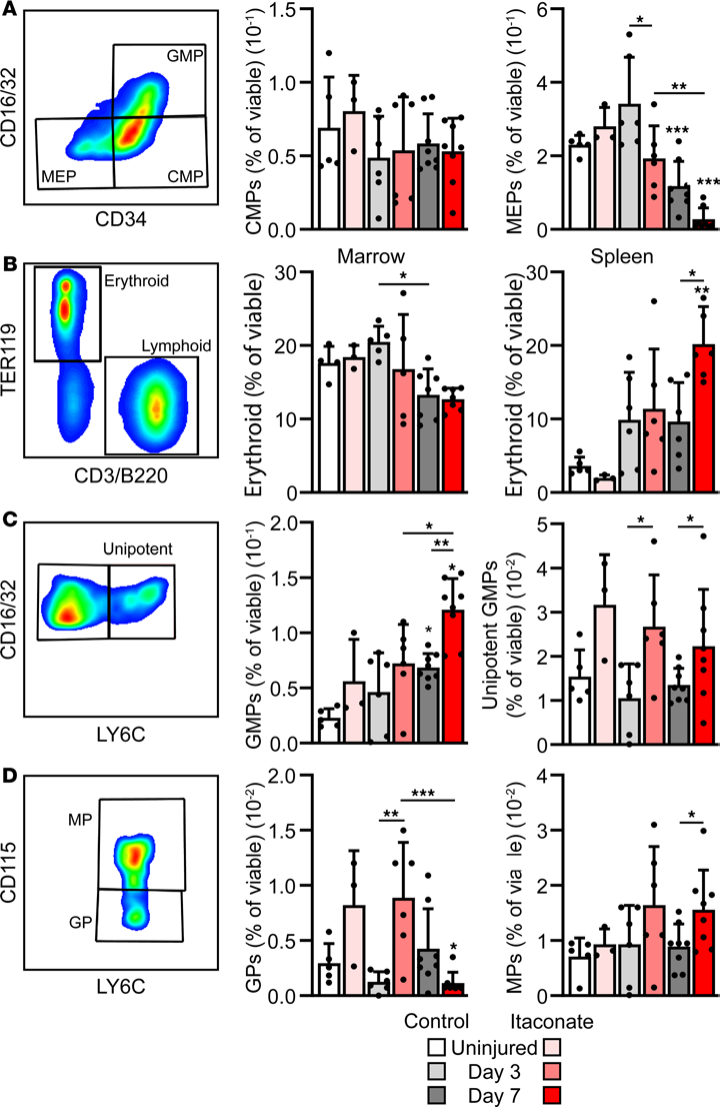
Treatment with exogenous itaconate alters bone marrow hematopoiesis. (**A**) Flow analysis of bone marrow progenitors from uninjured mice, as well as mice 3 and 7 days after burn/tenotomy with or without treatment with exogenous itaconate. (**B**) Flow analysis of bone marrow and spleen erythrocytes. (**C**) Flow analysis of total (from **A**) and unipotent bone marrow myeloid/granulocyte progenitors. (**D**) Flow analysis of granulocyte and monocyte progenitors. Data are shown as mean ± SD. *P < 0.05, **P < 0.01, ***P < 0.001, determined by 2-way ANOVA followed by Tukey’s multiple-comparison test, relative to uninjured saline controls unless otherwise denoted.

To determine the potential effects of itaconate within the bone marrow, hematopoietic progenitors and mature cells were profiled (36–38) (Supplemental Figure 7, A and B) in mice 0, 3, and 7 days after burn/tenotomy treated with itaconate or saline controls (Figure 5). Hematopoietic stem and progenitor cells differentiate into first CMPs before bifurcating into megakaryocyte erythrocyte progenitors (MEPs), eventually giving rise to the erythrocyte lineage, or to GMPs, which eventually give rise to neutrophils and monocytes/macrophages. In response to injury, while no significant changes were observed in CMPs, MEPs were significantly reduced at day 7 after injury, as compared with uninjured controls (Figure 5A). This MEP reduction was further exacerbated in injured mice treated with itaconate (Figure 5A). In response to stress, the spleen often serves as an extramedullary site for compensatory erythropoiesis. To determine the effects of this significant reduction in MEPs, we next profiled both the bone marrow and spleen for erythrocyte abundance. While only mild reductions in erythrocytes were observed in the bone marrow, significant increases in erythrocyte abundance were observed in the spleens of mice treated with itaconate for 7 days after burn/tenotomy, compared with both uninjured and day 7 saline-treated controls (Figure 5B). Conversely to the declines in MEPs, GMPs were significantly increased in day 7 itaconate-treated mice, both in comparison with uninjured controls as well as saline-treated mice on day 7 (Figure 5C). This increased propensity of GMPs in itaconate-treated mice resulted in increased unipotent GMPs at day 3 and day 7, with GPs significantly increased at day 3 and monocyte progenitors (MPs) significantly increased at day 7, relative to saline-treated, time point–matched controls (Figure 5D). When analyzing mature hematopoietic lineage cells within both the bone marrow and spleen, the burn/tenotomy procedure resulted in significantly elevated neutrophils within the bone marrow and a concomitant reduction in the abundance of bone marrow lymphoid cells but with minimal effects observed between itaconate and saline treated mice (Supplemental Figure 8). These data illustrate a role for itaconate in regulating hematopoiesis through modulating the commitment of CMPs to favor GMP differentiation at the expense of MEP lineage cells.

### Therapeutic administration of exogenous itaconate mitigates HO following trauma.

**Figure 6:**
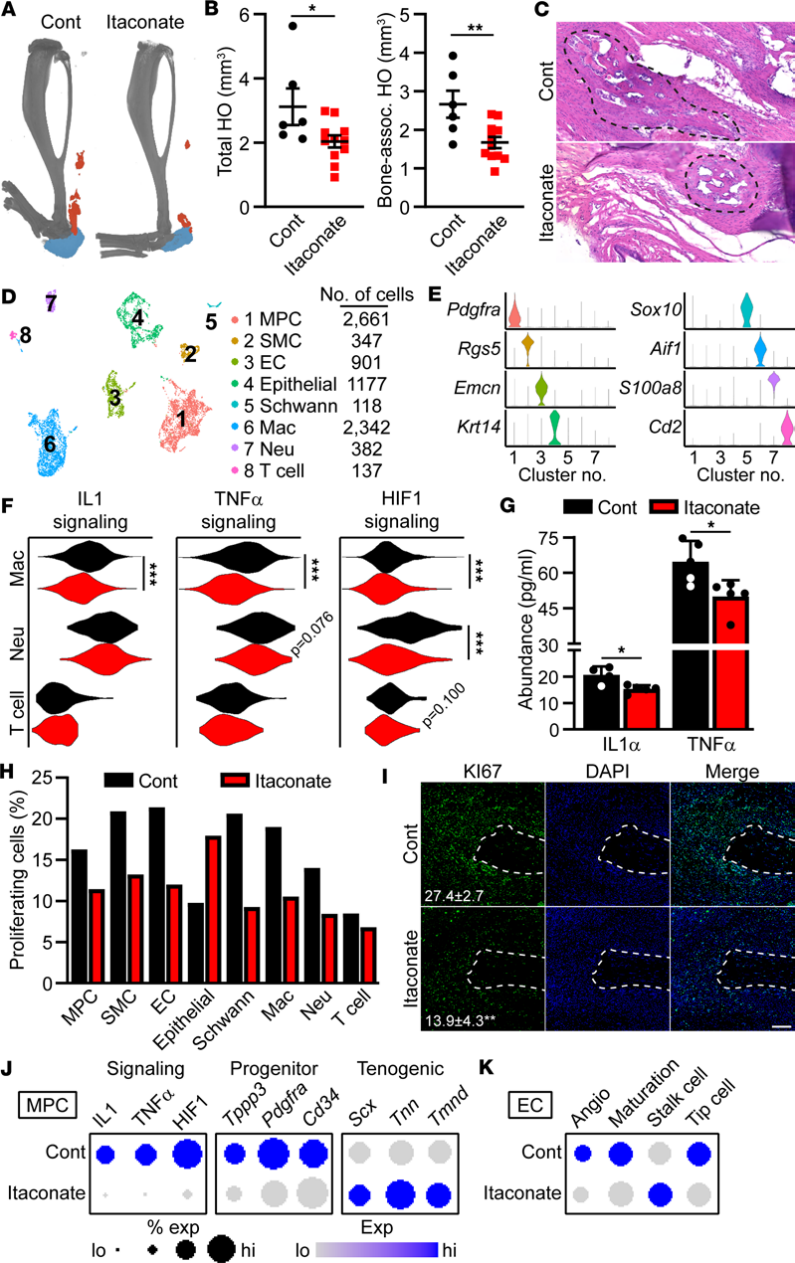
Therapeutic delivery of itaconate mitigates HO formation and progression. (**A**) Representative μ-CT at 9 weeks after burn/tenotomy in mice treated with itaconate or saline control. Heterotopic bone is highlighted in blue (bone-associated) and orange (tendon-associated). (**B**) Quantification of total and bone-associated HO formation by μ-CT. *n* = 6–11 per treatment group. (**C**) H&E of HO sites 9 weeks after burn/tenotomy injury. Dotted lines denote region of HO. (**D**) UMAP of scRNA-Seq data isolated from the tendon injury site of mice treated with itaconate and saline controls. (**E**) Marker genes used to identify cell clusters. (**F**) Module scores of indicated inflammatory pathways within immune cell populations. (**G**) Luminex analysis of protein concentrations within the tendon injury area. (**H**) Percent of scRNA-Seq cells predicted to be proliferating. *n* = 5/treatment. (**I**) Immunofluorescence of KI67 in burn/tenotomy mice 7 days after injury treated with either saline control or itaconate. Dotted line denotes severed Achilles tendon end. Scale bar: 100 µm. (**J**) Expression of genes involved in inflammatory signaling (left) or of markers for more progenitor (middle) or mature (right) tenogenic cells within the MPC cluster. (**K**) Module scores for terms linked to angiogenesis and vessel maturation within the endothelial cell cluster. Data are shown as mean ± SD. *P < 0.05, **P < 0.01, ***P < 0.001, determined by nonparametric 2-tailed *t* test (**B**, **G**, and **I**), Wilcoxon test (**F**), or 2-way ANOVA followed by Tukey’s multiple-comparison test (**G**), relative to saline controls.

Itaconate has previously been identified as an immunomodulatory metabolite (23, 24), promoting the transition of macrophages from a more M1-like proinflammatory to a more M2-like antiinflammatory phenotype. Owing to the critical role of inflammatory neutrophils and macrophages in HO formation and progression (3), we next sought to determine the effects of itaconate modulation in regulating HO formation. To further validate our finding that itaconate is endogenously produced, we made use of *LysM-Cre;Acod^fl/fl^* (*Acod1^LysM^*) mice to ablate itaconate production in both neutrophils and macrophages (39). qPCR analysis of the injury site 7 days after injury showed a significant reduction in *Acod1* expression in *Acod1^LysM^* mice (0.24 ± 0.26 relative to controls, *n* = 4, *P* < 0.05). Nine weeks after burn/tenotomy surgery, *Acod1^LysM^* mice showed significantly increased volumes of total HO, suggesting that itaconate endogenously produced by myeloid lineage cells may negatively regulate HO formation (Supplemental Figure 9, A and B). To determine the potential therapeutic effects of exogenous itaconate, mice were subjected to our burn/tenotomy surgery and treated with either itaconate or saline controls. μ-CT analysis of mice 9 weeks after surgery showed significant reductions in the total and bone-associated HO volumes in response to itaconate treatment (Figure 6, A and B). This reduced volume of HO was due to reduced formation, rather than delay in mineralization, as no notable cartilage remnants remained (Figure 6C). Importantly, while formation of HO was reduced, no observable deficiencies were observed in wound healing of the dorsal burn (Supplemental Figure 10). These data suggest treatment with itaconate can prevent formation and progression of HO following trauma.

To determine the mechanisms through which itaconate prevented HO formation, the tendon injury site from mice treated with itaconate or saline control were subjected to scRNA-Seq 7 days after surgery (Figure 6, D and E). Based on the previously published roles of itaconate in macrophages, we next profiled immune cell clusters for IL-1, TNF-α, and hypoxia inducible factor 1 (HIF1) signaling (Figure 6F). Module scores, defined as the average expression of a gene list (Supplemental Table 4) relative to background expression, demonstrated reduced activation of IL-1, TNF-α, and HIF1 signaling pathways within the macrophage population, with HIF1 signaling also significantly reduced in neutrophils (Figure 6F). This reduced transcriptional signaling observed by scRNA-Seq was accompanied by reduced levels of IL-1 and TNF-α ligands within the tendon injury site 7 days after burn/tenotomy surgery in response to itaconate (Figure 6G).

In addition to its effects on immune cells, scRNA-Seq data were also analyzed for changes in nonimmune cells resulting from treatment with itaconate. With the exception of epithelial cells, which showed a notable increase, cell cycle phase predictions showed marked reduction in cell proliferation within each cell cluster (Figure 6H). Reduced overall proliferation of cells within the tendon injury site following treatment with itaconate was confirmed by KI67 staining (Figure 6I). Similar to the macrophage cluster, MPCs also showed reduced activation of IL-1, TNF-α, and HIF1 in response to itaconate treatment; they also showed a shift in expression of tenogenic markers, with itaconate downregulating expression of the progenitor markers *Tppp3*, *Pdgfra*, and *Cd34* and upregulating the more mature tenogenic markers *Scx*, *Tnn* and *Tmnd* (Figure 6J). In addition to changes in MPCs, analyses also revealed reduced expression of genes linked to sprouting angiogenesis in response to itaconate treatment (Figure 6K and Supplemental Table 5). Collectively, these data suggest that exogenous itaconate can serve as a potential therapeutic strategy to prevent the formation and progression of HO by reducing local inflammation, reducing proliferation, promoting tenogenic differentiation, and reducing angiogenesis, without affecting normal wound healing.

## Discussion

In this study, we report previously unobserved regulatory mechanisms through which neutrophils can regulate both local and systemic inflammation. Following a combination of burn and musculoskeletal injury, we observed an accumulation of the immunomodulatory metabolite itaconate within the Achilles tendon region. Additional metabolic and molecular characterizations revealed that a highly mature subset of neutrophils, not the previously reported macrophages, were the primary producers of itaconate within the injury site. Characterization of injury-site neutrophils showed high levels of inflammatory cytokine production, likely contributing to the prolonged inflammation typified in this trauma model and necessary for aberrant cell differentiation. In contrast, while itaconate was produced within these same highly inflammatory neutrophils present at the injury site, this antiinflammatory metabolite appeared to remain intracellular. Tracking these neutrophils along their life cycle suggests that these highly mature neutrophils migrate back to the bone marrow, where they are engulfed by macrophages, altering hematopoiesis and skewing cells away from the erythrocytes in favor of the monocytic and granulocytic lineages. Within the injury site, treatment with itaconate resulted in reduced inflammation and vascular invasion, and altered differentiation, resulting in reduced aberrant repair.

While factors guiding wound healing have been extensively studied at the transcriptional level, the role of shifting bioenergetics requires further investigation. To this end, we conducted metabolomic profiling of models of musculoskeletal injury or systemic inflammation in combination or alone. In addition to altered bioenergetics, these metabolic studies congruently identified itaconate accumulation within the injury site. Itaconate is synthesized from the TCA cycle intermediate aconitate through the activity of aconitate decarboxylase encoded by *Acod1* (23). Previous reports have identified macrophages as the primary, if not sole, producers of itaconate in response to inflammatory stimulation or infection (23, 25, 40). In the case of stimulation with LPS, macrophages upregulate *Acod1* and subsequent itaconate production (23). In a KEAP1/NRF2-mediated mechanism, itaconate production results in transition of macrophages from a more M1 proinflammatory to an M2 antiinflammatory state (29, 41, 42).

As with infection, macrophages have been shown to play a critical role in injury repair (3, 43). Surprisingly, our subsequent molecular and metabolic studies identified neutrophils, not macrophages, as the major producers of itaconate following trauma. These findings are consistent with recently published results that identify neutrophils as the primary producers of itaconate following respiratory infection (30). Further characterization of neutrophils identified a transcriptionally unique, highly mature subpopulation of neutrophils (9, 35) heavily enriched within the tendon injury site. Despite the presence of systemic inflammation induced by the dorsal burn (44), tendon injury–site (HO) neutrophils were found to be unique from those in circulation in the blood or present within the bone marrow, regulating local inflammation through the robust expression of inflammatory cytokines such as IL-1α/β and TNF-α. Neutrophils typically represent one of the earliest responders to inflammatory stimuli (45). In response to injuries that undergo more faithful repair, the inflammatory phase is typically restricted to the first several days following injury, with neutrophils often entering and vacating the trauma site within the first 48–72 hours (10, 46). In contrast, in injuries with sustained aberrant inflammation such as HO, neutrophils are present out past 6 weeks after injury (22). This prolonged presence of neutrophils within the injury site, the highly inflammatory nature of these mature neutrophils, and the essential role of inflammation in HO formation suggest that neutrophils may regulate the local inflammatory environment, leading to the aberrant differentiation underscoring HO progression.

While our metabolomic data point to itaconate production occurring within the injury site, analysis of conditioned media from human neutrophils studied in vitro suggests that this neutrophil-derived itaconate produced in response to inflammatory induction remains intracellular. While further studies are needed to confirm these results, it appears that neutrophil itaconate may not represent a mechanism through which neutrophils regulate the injury site. While previous views held that sites of injury or infection represented the terminal point of the neutrophil lifespan, more recent work and this study show that neutrophils can extravasate from sites of inflammation and cycle back to the bone marrow (13, 47). This life cycle suggests that neutrophil itaconate produced at the injury site may be delivered back to the bone marrow. Injection of neutrophils derived from the injury site showed a clear and intimate association with bone marrow macrophages. This is consistent with previous works that have suggested macrophage-mediated efferocytosis of neutrophils declines with age (16). To determine the effects of itaconate within the bone marrow, we profiled hematopoietic lineage cells isolated from the bone marrow. Our results indicate that itaconate can result in skewing of hematopoiesis, increasing the relative abundance of GMPs (and their downstream progenitor populations) at the expense of MEPs.

While our data support itaconate-mediated hematopoietic skewing in the bone marrow, further studies are needed to determine the mechanism through which this process is mediated. Limited studies have suggested that neutrophils could regulate the hematopoietic niche (14, 15). One possibility is that itaconate works through modulation of macrophages, which are often associated with the HSC niche. Increased levels of macrophage IL-1β associated with aging has previously been linked to the increased differentiation of MEP-derived cell lineage (16). It has also previously been noted that itaconate can downregulate IL-1β in macrophages (25, 48). This raises the intriguing possibility that neutrophils could deliver itaconate to marrow macrophages, modulating hematopoiesis through regulation of macrophage IL-1β. This concept is more appealing considering our in vitro culture data, which demonstrate that, while neutrophil maturation — including upregulation of CXCR4 driving cycling back to the bone marrow — can be induced without stimulation, these neutrophils did not produce elevated levels of itaconate. This contrasts with in vitro stimulation using TNF-α or a MRSA-derived cell membrane preparation, which both showed robust upregulation of itaconate in mouse and human neutrophils.

One limitation of this study is the use of itaconate injections to observe hematopoietic skewing. It is technically and logistically challenging to isolate neutrophils from the tendon injury site in large quantities. However, it has been proposed that mice can produce about 1 ***×*** 10^7^ neutrophils daily (49) and likely even greater numbers in response to inflammation and trauma. While neutrophils can be cleared through the liver, spleen, and bone marrow under homeostatic conditions (50, 51), previous work suggests that, following injury, neutrophils were selectively cleared by the bone marrow (52). It remains to be determined whether this endogenous neutrophil production could deliver sufficient itaconate to the bone marrow to result in hematopoietic skewing observed following daily exogenous injections. The direct delivery of neutrophils to the bone marrow, and perhaps more specifically bone marrow macrophages, may suggest that targeted delivery of itaconate through neutrophil cycling, rather than systemic delivery, may affect hematopoiesis at levels far reduced from our exogenous administrations. Additionally, it is difficult to distinguish direct and indirect effects of both endogenously produced and exogenously administered itaconate within the tendon injury environment. Though transcriptional and metabolomic analyses point to neutrophils as the primary producers of itaconate, we did observe expression of *Acod1* and production of itaconate in macrophages. Further work is required to disentangle the neutrophil-specific effects from other cell types within the tendon injury area.

Though additional studies are needed, this hypothesis that neutrophils synthesize itaconate and that itaconate can regulate myelopoiesis, if confirmed, could present a potential mechanism through which neutrophils could serve as a “sensor” of inflammation, relaying the extent of distant insult (in the form of itaconate abundance) back to the bone marrow to modulate a proportional mobilization of myeloid cells to further combat ongoing inflammatory cascades.

## Methods

### Animals.

Male C57BL/6J mice were obtained from The Jackson Laboratory (stock no. 000664). Animals were housed and maintained under standard conditions and had access to food and water ad libitum. Control and experimental groups of mice were fed a diet of normal chow and water with exposure to light for 12 hours daily. *LysM-Cre;Acod1^fl/fl^* mice were a gift from Christina L. Stallings (Washington University School of Medicine, St. Louis, Missouri, USA) and bred in house.

An established model of posttraumatic HO formation was used (3, 22, 53) on mice at 10 weeks of age. Mice were anesthetized with isoflurane inhalation. The hair was shaved off the burn and tenotomy sites and cleaned with alcohol and betadine 3 times. Buprenorphine (Buprenex, Reckitt Benckinser Pharmaceuticals) was administered s.c. immediately prior to surgery at 0.06 mg/kg. Mice received a 30% surface area burn to their dorsum with an aluminum block that was heated to 60°C for 18 seconds, immediately followed by complete transection of the Achilles tendon at the midpoint, with placement of a single 5-0 Vicryl suture to close the skin. Mice were monitored during recovery and then at 12, 24, and 72 hours after surgery.

For itaconate therapeutic studies, mice were randomized into control and treatment groups preoperatively. The control group received saline (PBS, Thermo Fisher Scientific), and the treatment group received 400 mg/kg dimethyl itaconate (Sigma Aldrich, 592498-25G) injected i.p. daily starting 2 days prior to injury.

### Metabolomic profiling.

Metabolic profiling was carried out by the CRI Metabolomics core at UT Southwestern (UTSW) as previously published (54, 55). For tendon injury–site metabolic profiling, injury-site soft tissue was isolated, including the inflamed tissue and flanking proximal and distal tendon remnants but excluding overlaying skin, surrounding muscle or underlying bone and flash frozen in liquid nitrogen. Metabolites were isolated using a methanol-based extraction, lyophilized, and resuspended in 80% acetonitrile/water. For metabolomics of cultured cells, fractionating cells, or conditioned media, metabolites were extracted directly with 80% acetonitrile/water. All samples were normalized using the Pierce BCA Protein Assay Kit (Thermo Fisher Scientific, 23225 and 23227). Hydrophilic interaction chromatography (HILIC) chromatographic separation of metabolites was achieved using a MilliporeSigma ZIC-pHILIC column (5 μm, 2.1 × 150 mm) with a binary solvent system of 10 mM ammonium acetate in water, pH 9.8 (solvent A) and acetonitrile (solvent B) with a constant flow rate of 0.25 mL/minute. Metabolites were measured with a Thermo Fisher Scientific QExactive HF-X hybrid quadrupole orbitrap high-resolution mass spectrometer (HRMS) coupled to a Vanquish UHPLC. Metabolite identities were confirmed in 3 ways: (a) precursor ion *m*/*z* was matched within 5 ppm of theoretical mass predicted by the chemical formula; (b) fragment ion spectra were matched within a 5 ppm tolerance to known metabolite fragments; and (c) the retention time of metabolites was within 5% of the retention time of a purified standard run with the same chromatographic method. Metabolites were relatively quantified by integrating the chromatographic peak area of the precursor ion searched within a 5 ppm tolerance.

### Cell suspension and fractionation.

Mice were euthanized with CO_2_, immediately followed by cardiac puncture to retrieve blood. Blood was collected into EDTA-coated tubes to prevent coagulation. Blood was layered onto a Ficoll gradient and centrifuged at 1,750*g* for 10 minutes at 20°C, with a deceleration of 3. Mononucleated cells were collected for further processing. Bone marrow was flushed from both femurs with FACS Buffer (10% FBS and 0.2 mM EDTA [pH 7.4] in PBS). Both blood mononucleated cells and bone marrow were next subjected to red blood cell lysis (Thermo Fisher Scientific, A1049201). For the tendon injury site, single-cell suspensions were generated as previously described (22). Briefly, the entire injury site, from the distal tip of the gastrocnemius to the calcaneus, was harvested and minced to pieces. Tissue was then subjected to enzymatic digestion using Collagenase I at 3 mg/mL (Thermo Fisher Scientific, 17100-017), Collagenase II at 2 mg/mL (Thermo Fisher Scientific, 17101-015), and Dispase at 3mg/mL (MilliporeSigma, D4693) per 1 mL of DMEM (Thermo Fisher Scientific, 11960044) for 20–30 minutes at 37°C under constant agitation at 160 rpm.

For cellular fractionation, prepared cell suspensions were first overlayed on a histopaque gradient (Sigma Aldrich, 11191 and 10771) and then centrifuged at 900*g* for 30 minutes at 25°C. Neutrophils were collected from the internal histopaque interface. The top layer of cells was collected and subsequently subjected to magnetic separation using anti-CD11b beads (Stem Cell Technologies, 18970A) to collect monocytes/macrophages. The remaining unbound cells were pooled and termed “rest.”

### scRNA-Seq.

Single-cell libraries were generated from single cell suspensions described above using the 10x Genomics Chromium controller following the manufacturer’s protocol. Libraries were sequenced on an Illumina NovaSeq SP 100 cycles. CellRanger was used to perform sample demultiplexing, barcode processing, and single-cell gene counting (alignment, barcoding, and unique molecular identifier [UMI] count). Downstream analysis steps were performed using Seurat (56). Pathway activation or module scores were generated using the AddModuleScore function of Seurat using validated gene lists from KEGG pathways. Module scores were calculated as the level of gene expression enrichment of a set gene list relative to a random control list, with higher module score values representing positive enrichment beyond background.

To determine the potential interactions between different cell clusters, we performed CCC analysis using the *R* package CellChat (57). To further explore the functional outcomes mediated by these identified ligand-receptor pair interactions, we constructed scRNA-Seq–based multilayer networks using the R package scMLnet (58). ScMLnet constructs multilevel networks by integrating intercellular pathways (ligand-receptor interactions) and intracellular subnetworks (receptor-transcription factor and transcription factor–target gene interactions) as well as the intercellular/intracellular signaling pathways constructed between the central cell and neighboring cells. For analyses related to the regulation of receptor presentation, gene lists were pulled from published works related to CXCR2/4 gene expression, dephosphorylation, and recycling of internal receptors back to the surface (denoted activators) as well as genes linked to ligand binding and internalization, desensitization, phosphorylation, and degradation (inactivators).

### Luminex.

Bone marrow was processed as above with the addition 3 cycles of freeze/thaw. After centrifugation at 10,000*g* for 20 minutes at 4°C, supernatant was collected and flash frozen. Whole blood was collected via intracardiac puncture. Samples were centrifuged at 9,600*g* for 10 minutes at 4°C, and plasma was then transferred to cryo-vials and flash frozen. The injury-site tissue was collected in cold PBS and lightly minced with scissors in 500 μL of wash buffer before being replaced with 500 μL of lysis buffer (9.9 mL lysis buffer, 1 tablet Complete mini-EDTA free protease inhibitor [Sigma-Aldrich, 11836170001], 40 μL Factor 1, 20 μL Factor 2 from the cell lysis kit). Cells were lysed with 4 cycles of sonication, with the probe set to 50% at 5 seconds on and 10 seconds off (Thermo Fisher Scientific, Model 120 Sonic Dismemberator, 12337338). Samples were held on ice after 2 rounds of sonication. Samples were centrifuged at 4,500*g* for 10 minutes at 4°C. A Pierce BCA Protein Assay Kit (Thermo Fisher Scientific, 23225 and 23227) was used to determine protein concentration. All samples were processed using the Bio-Rad cell lysis kit (171-304011).

### Flow cytometry analysis of hematopoiesis.

Analysis of bone marrow and spleen hematopoietic cells were carried out as previously described (36–38). Briefly, following burn/tenotomy surgeries at 3- and 7-day time points, bone marrow and spleens were harvested for flow cytometry data collection. Bone marrow was flushed from both femurs with 3 mL ice-cold PBS and filtered through a 100 μm cell strainer. Spleens were mechanically dissociated through a 40 μm cell strainer with 5 mL of ice-cold PBS. For hematopoietic progenitors, 9 fluorophore-conjugated antibodies were used to sort for viability, erythroid, lymphoid and myeloid cells. The antibody master mixture contained c-kit 1:200 (eBiosciences, 47-1171-82, clone 2B8), Sca1 1:200 (BioLegend, 108114, clone D7), Lineage (CD2, 1:400 [BioLegend, 100112, clone RM2-5], CD3, 1:400 [Tonbo Biosciences, 20-0032-U100, clone 17A2], CD5 1:400 [BioLegend, 100626, clone 53-7.3], CD8a 1:400 [Tonbo Biosciences, 20-0081-U100, clone 53-6.7], B220/CD45R 1:400 [Tonbo Biosciences 20-0452-U100, clone RA3-6B2], Ter119 1:400 [Tonbo Biosciences, 20-5921-U100], and Gr1 1:400 [Tonbo Biosciences, 20-5931-U100]), LY6G 1:400 (BioLegend, 127627, clone 1A8), CD34 1:100 (eBiosciences, 11-0341-85, clone RAM34), CD16/32 1:200 (BioLegend,101333, clone 93), CD115 1:200 (Tonbo Biosciences, 50-1152-U100, clone AFS98), and propidium iodide (Thermo Fisher Scientific, BDB556463). For differentiated cells, the antibody master mixture included CD71 1:400 (eBiosciences, 11-0711-82, clone RI7 217.1.4), Ter119 1:200 (BioLegend, 116237), B220-CD3 1:200 (Tonbo Biosciences, 20-0452-U100, clone RA3-6B2), and Mac1 1:400 (eBiosciences, 47-0112-82, clone M1/70). Data were collected using a FACS Lyric (BD Biosciences).

### Neutrophil transplant studies.

Following histopaque-mediated neutrophil isolation from the HO site 7 days after burn/tenotomy in WT mice, cell membranes were labeled with the DiO Lipophilic Carbocyanine fluorescent green dye kit (Biotium, 60011). Labeled neutrophils were injected into *CD169-Cre;Rosa-tdTomato* (Riken). Following tail vein injection, mice were harvested for bone marrow and HO site as described above. Cells were stained with fluorophore-conjugate antibodies LY6G (Tonbo Biosciences, 60-1276-U100, clone 1A8) and CD11B (Thermo Fisher Scientific, 47-0112-82, clone M1/70).

### Isolation and in vitro stimulation of human neutrophils.

Human neutrophils were isolated according to standard techniques from acid citrate dextrose anticoagulated venous blood. Neutrophils were isolated using Hypaque-Ficoll density-gradient separation and dextran sedimentation followed by hypotonic lysis of erythrocytes as previously described (59, 60). Neutrophil purity is routinely monitored via cytospin and found to be > 98%. Freshly isolated neutrophils were incubated with specified stimuli at a neutrophil concentration of 5 ***×*** 10^6^/mL in HBSS containing 0.1 % glucose and 1% human serum albumin (assay buffer) for 4 hours tumbling in a 37°C/5 % CO_2_ incubator. Neutrophils were stimulated with either recombinant TNF-α (1 ng/mL for human, 25 ng/mL for mouse) or MRSA-CM prepared from *S*. *aureus* (100 ng/mL) as previously described (61). In addition, a control time 0 sample was collected by adding freshly isolated neutrophils to assay buffer and placing them immediately on ice. Conditioned media and neutrophil cell pellets were collected. For gene expression, pellets were resuspended in lysis buffer and stored at –80°C. For mass spectrometry, conditioned media and pellets were flash frozen and stored at –80°C.

### Histological analyses.

Hind limb samples were harvested at 7 days and 9 weeks after burn/tenotomy surgery. Samples were fixed in 4% paraformaldehyde (PFA) for 24 hours at 4°C, washed with PBS, and decalcified using 14% EDTA for 4–5 weeks. Tissue was then either embedded in optimal cutting temperature media (Sakura) or sent to the histology core at UTSW for paraffin embedding. Cryosections were sectioned at a thickness of 35 μm and imaged for neutrophil transplantation studies. Paraffin sections were cut at a thickness of 6 μm and stained for H&E or anti-KI67 (R&D systems, AF7649).

RNAscope Multiplex Fluorescent Reagent Kit v2 Assay (ACD Biosciences) was used for RNA in situ hybridization. The injury area was collected from mice 7 days after burn/tenotomy and fixed in PFA. Sagittal sections were collected at 12 μm onto Fisher SuperFrost Microscope slides. Two slides were used for RNAscope staining. AIF-C2 (ACD Biosciences, 319141-C2), Acod1-C1 (ACD Biosciences, 450241), and S01008-C3 (ACD Biosciences, 478511-C3) markers were used with correlated Akoya Biosciences Opal Dye 520 (NC1601877) and 690 (NC1605064) and Fisher Scientific Opal 570 Reagent Pack (NC1601878), respectively.

### μ-CT.

μ-CT images were taken at the University of Texas Southwestern using a Mediso USA nano Scan PET/CT system. The scanning parameters were max zoom with 720 helical projections with x-ray power of 70 kV at 980 μA, an exposure time of 300 ms, and a voxel size of 45 μm^3^. Scans were analyzed using Dragonfly ORS by a blinded operator manually scoring ectopic bone at a single threshold of 800 Hounsfield unit. In addition to total ectopic bone, results were further subdivided between “bone-associated” ectopic bone contiguous with the calcaneus and “floating” bone proximal to the calcaneus.

### Statistics.

Statistical analyses were performed using GraphPad Prism (v9) or the *R* package *ggpubr*. A 2-tailed Student’s *t* test or 2-way ANOVA followed by Tukey’s multiple-comparison test was used to evaluate statistical significance. *P* values less than 0.05 were considered statistically significant.

### Study approval.

All animal experiments described were approved by the IACUC at the University of Texas at Southwestern (Animal Protocol Number [APN] 2021-103130, 2020-102949). This study was carried out in strict accordance with the guidelines provided in the *Guide for the Care and Use of Laboratory Animals* (National Academies Press, 2011). Human neutrophils were isolated from healthy consenting adults following written informed consent and in accordance with a protocol approved by the IRB for Human Subjects at the University of Texas Southwestern Medical Center.

### Data availability.

scRNA-Seq data generated for this study has been deposited in the Gene Expression Omnibus (GEO) database and is freely available under the accession code GSE221134. Individual data points are available in the Supporting Data Values file.

## Author contributions

Conception or design of the work was contributed by RJT and BL; data collection was contributed by JLC, SOG, JSH, NV, ARH, and RJT; data analysis and interpretation were contributed by JLC, SOG, SC, LG, CJ, LX, GH, JGM, RJT, and BL; drafting the article was contributed by RJT; and critical revision of the article was contributed by BL. All authors approved the final version of the manuscript.

## Supplementary Material

Supplemental data

Supplemental tables 1-5

Supporting data values

## Version 1 09/14/2023

In-Press Preview

## Version 2 10/23/2023

Electronic publication

## References

[1] Medzhitov R (2010). Inflammation 2010: new adventures of an old flame. Cell.

[2] Osuka A (2014). Immune response to traumatic injury: harmony and discordance of immune system homeostasis. Acute Med Surg.

[3] Sorkin M (2020). Regulation of heterotopic ossification by monocytes in a mouse model of aberrant wound healing. Nat Commun.

[4] Brown KV (2010). Comparison of development of heterotopic ossification in injured US and UK Armed Services personnel with combat-related amputations: preliminary findings and hypotheses regarding causality. J Trauma.

[5] Ranganathan K (2015). Heterotopic ossification: basic-science principles and clinical correlates. J Bone Joint Surg Am.

[6] Pavey GJ (2015). What risk factors predict recurrence of heterotopic ossification after excision in combat-related amputations?. Clin Orthop Relat Res.

[7] Levi B (2015). Risk factors for the development of heterotopic ossification in seriously burned adults: a National Institute on Disability, Independent Living and Rehabilitation Research burn model system database analysis. J Trauma Acute Care Surg.

[8] Rosales C (2018). Neutrophil: a cell with many roles in inflammation or several cell types?. Front Physiol.

[9] Xie X (2020). Single-cell transcriptome profiling reveals neutrophil heterogeneity in homeostasis and infection. Nat Immunol.

[10] Wang J (2018). Neutrophils in tissue injury and repair. Cell Tissue Res.

[11] Tsuda Y (2004). Three different neutrophil subsets exhibited in mice with different susceptibilities to infection by methicillin-resistant Staphylococcus aureus. Immunity.

[12] Bratton DL, Henson PM (2011). Neutrophil clearance: when the party is over, clean-up begins. Trends Immunol.

[13] Hidalgo A (2019). The neutrophil life cycle. Trends Immunol.

[14] Cossio I (2019). Neutrophils as regulators of the hematopoietic niche. Blood.

[15] Casanova-Acebes M (2013). Rhythmic modulation of the hematopoietic niche through neutrophil clearance. Cell.

[16] Frisch BJ (2019). Aged marrow macrophages expand platelet-biased hematopoietic stem cells via interleukin-1B. JCI Insight.

[17] Tower RJ (2022). Spatial transcriptomics reveals metabolic changes underly age-dependent declines in digit regeneration. Elife.

[18] Shen LY (2022). Bioenergetic metabolism in osteoblast differentiation. Curr Osteoporos Rep.

[19] Zhang K (2018). Modulating glucose metabolism and lactate synthesis in injured mouse tendons: treatment with dichloroacetate, a lactate synthesis inhibitor, improves tendon healing. Am J Sports Med.

[20] Evans WJ, Cannon JG (1991). The metabolic effects of exercise-induced muscle damage. Exerc Sport Sci Rev.

[21] Peterson JR (2014). Treatment of heterotopic ossification through remote ATP hydrolysis. Sci Transl Med.

[22] Pagani CA (2021). Novel lineage-tracing system to identify site-specific ectopic bone precursor cells. Stem Cell Reports.

[23] O’Neill LAJ, Artyomov MN (2019). Itaconate: the poster child of metabolic reprogramming in macrophage function. Nat Rev Immunol.

[24] Li RD (2020). Itaconate: a metabolite regulates inflammation response and oxidative stress. Oxid Med Cell Longev.

[25] Lampropoulou V (2016). Itaconate links inhibition of succinate dehydrogenase with macrophage metabolic remodeling and regulation of inflammation. Cell Metab.

[26] Patel NK (2022). Macrophage TGF-β signaling is critical for wound healing with heterotopic ossification after trauma. JCI Insight.

[27] Xu J (2022). NGF-p75 signaling coordinates skeletal cell migration during bone repair. Sci Adv.

[28] Larouche JA (2022). Neutrophil and natural killer cell imbalances prevent muscle stem cell-mediated regeneration following murine volumetric muscle loss. Proc Natl Acad Sci U S A.

[29] He RY (2022). Itaconate inhibits ferroptosis of macrophage via Nrf2 pathways against sepsis-induced acute lung injury. Cell Death Discov.

[30] Tomlinson KL (2023). Staphylococcus aureus stimulates neutrophil itaconate production that suppresses the oxidative burst. Cell Rep.

[31] Agarwal S (2019). Disruption of neutrophil extracellular traps (NETs) links mechanical strain to post-traumatic inflammation. Front Immunol.

[32] Nunez JH Neutrophil and NETosis modulation in traumatic heterotopic ossification. Ann Surg.

[33] Karasawa K (2015). Vascular-resident CD169-positive monocytes and macrophages control neutrophil accumulation in the kidney with ischemia-reperfusion injury. J Am Soc Nephrol.

[34] Gupta P (2016). Tissue-resident CD169(+) macrophages form a crucial front line against plasmodium infection. Cell Rep.

[35] Grieshaber-Bouyer R (2021). The neutrotime transcriptional signature defines a single continuum of neutrophils across biological compartments. Nat Commun.

[36] Akashi K (2000). A clonogenic common myeloid progenitor that gives rise to all myeloid lineages. Nature.

[37] Comazzetto S (2019). Restricted hematopoietic progenitors and erythropoiesis require SCF from leptin receptor plus niche cells in the bone marrow. Cell Stem Cell.

[38] Yanez A (2015). IRF8 acts in lineage-committed rather than oligopotent progenitors to control neutrophil vs monocyte production. Blood.

[39] Abram CL (2014). Comparative analysis of the efficiency and specificity of myeloid-Cre deleting strains using ROSA-EYFP reporter mice. J Immunol Methods.

[40] Runtsch MC (2022). Itaconate and itaconate derivatives target JAK1 to suppress alternative activation of macrophages. Cell Metab.

[41] Mills EL (2018). Itaconate is an anti-inflammatory metabolite that activates Nrf2 via alkylation of KEAP1. Nature.

[42] Ryan DG (2022). Nrf2 activation reprograms macrophage intermediary metabolism and suppresses the type I interferon response. iScience.

[43] Krzyszczyk P (2018). The role of macrophages in acute and chronic wound healing and interventions to promote pro-wound healing phenotypes. Front Physiol.

[44] Mulder PPG (2022). Burn-induced local and systemic immune response: systematic review and meta-analysis of animal studies. J Invest Dermatol.

[45] Nathan C (2006). Neutrophils and immunity: challenges and opportunities. Nat Rev Immunol.

[46] Wilgus TA (2013). Neutrophils and wound repair: positive actions and negative reactions. Adv Wound Care (New Rochelle).

[47] Brostjan C, Oehler R (2020). The role of neutrophil death in chronic inflammation and cancer. Cell Death Discov.

[48] Kelly B, O’Neill LAJ (2015). Metabolic reprogramming in macrophages and dendritic cells in innate immunity. Cell Res.

[49] Boxio R (2004). Mouse bone marrow contains large numbers of functionally competent neutrophils. J Leukoc Biol.

[50] De Filippo K, Rankin SM (2018). CXCR4, the master regulator of neutrophil trafficking in homeostasis and disease. Eur J Clin Invest.

[51] Furze RC, Rankin SM (2008). The role of the bone marrow in neutrophil clearance under homeostatic conditions in the mouse. FASEB J.

[52] Wang J (2017). Visualizing the function and fate of neutrophils in sterile injury and repair. Science.

[53] Lee S (2021). NGF-TrkA signaling dictates neural ingrowth and aberrant osteochondral differentiation after soft tissue trauma. Nat Commun.

[54] DeVilbiss AW (2021). Metabolomic profiling of rare cell populations isolated by flow cytometry from tissues. Elife.

[55] Tasdogan A (2020). Metabolic heterogeneity confers differences in melanoma metastatic potential. Nature.

[56] Hao Y (2021). Integrated analysis of multimodal single-cell data. Cell.

[57] Jin S (2021). Inference and analysis of cell-cell communication using CellChat. Nat Commun.

[58] Cheng J (2021). Inferring microenvironmental regulation of gene expression from single-cell RNA sequencing data using scMLnet with an application to COVID-19. Brief Bioinform.

[59] Nauseef WM (2014). Isolation of human neutrophils from venous blood. Methods Mol Biol.

[60] Kuhns DB (2015). Isolation and functional analysis of human neutrophils. Curr Protoc Immunol.

[61] Hook JS (2021). Lipoproteins from Staphylococcus aureus drive neutrophil extracellular trap formation in a TLR2/1- and PAD-dependent manner. J Immunol.

[62] Huber AK (2020). Immobilization after injury alters extracellular matrix and stem cell fate. J Clin Invest.

