# Supplementary material for: Itaconate-producing neutrophils regulate local and systemic inflammation following trauma
Source: JCI Insight. 2023 Oct 23;8(20):e169208. doi: 10.1172/jci.insight.169208 (PMC10619500; doi:10.1172/jci.insight.169208)
Supplement: Supplemental data [file jciinsight-8-169208-s115.pdf]

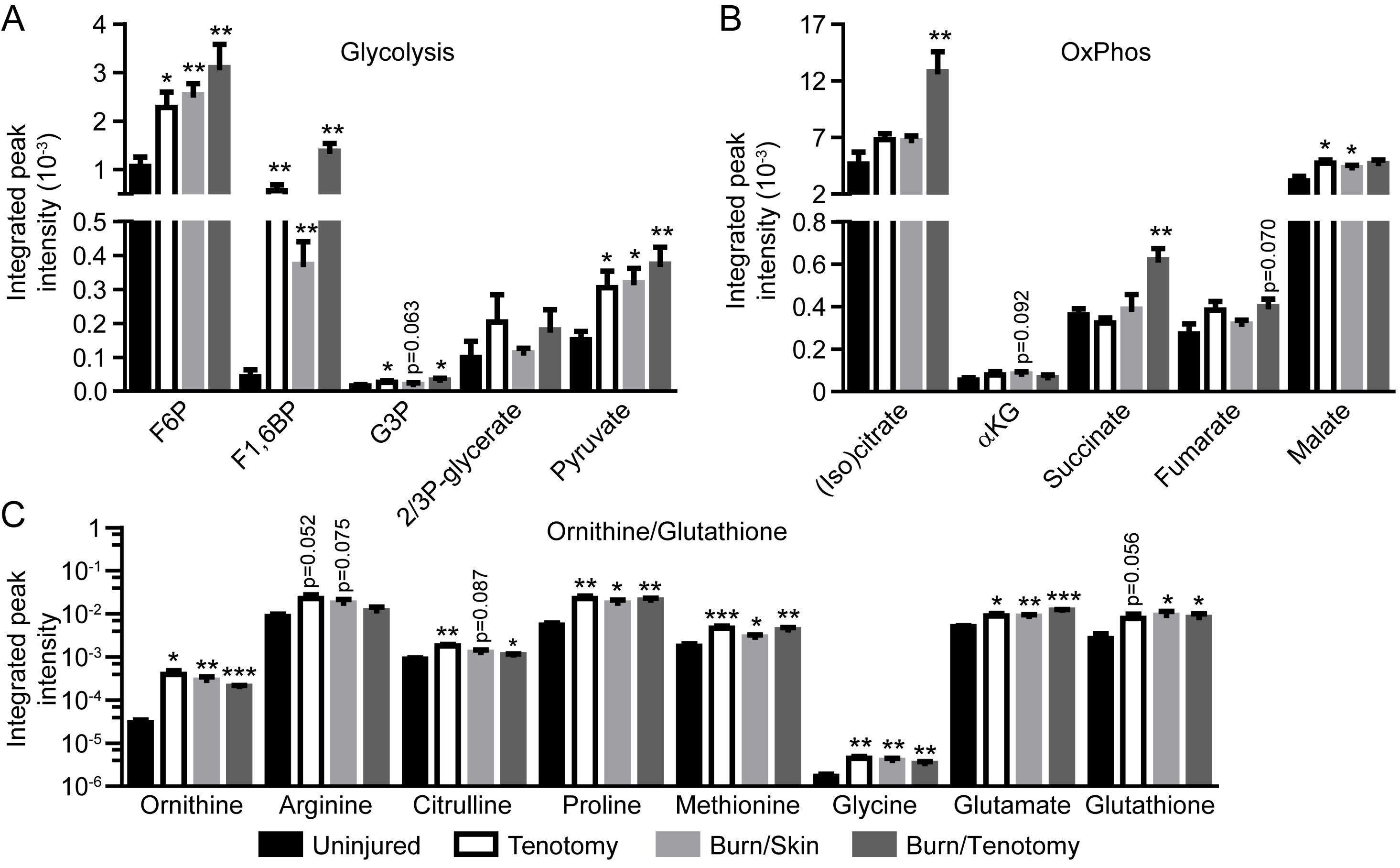

A
Glycolysis
Integrated peak intensity (10^-3)
F6P
F1,6BP
G3P
2/3P-glycerate
Pyruvate
p=0.063
B
OxPhos
(Iso)citrate
αKG
Succinate
Fumarate
Malate
p=0.092
p=0.070
C
Ornithine/Glutathione
Integrated peak intensity
Ornithine
Arginine
Citrulline
Proline
Methionine
Glycine
Glutamate
Glutathione
p=0.052
p=0.075
p=0.087
p=0.056
Uninjured
Tenotomy
Burn/Skin
Burn/Tenotomy

Figure S1

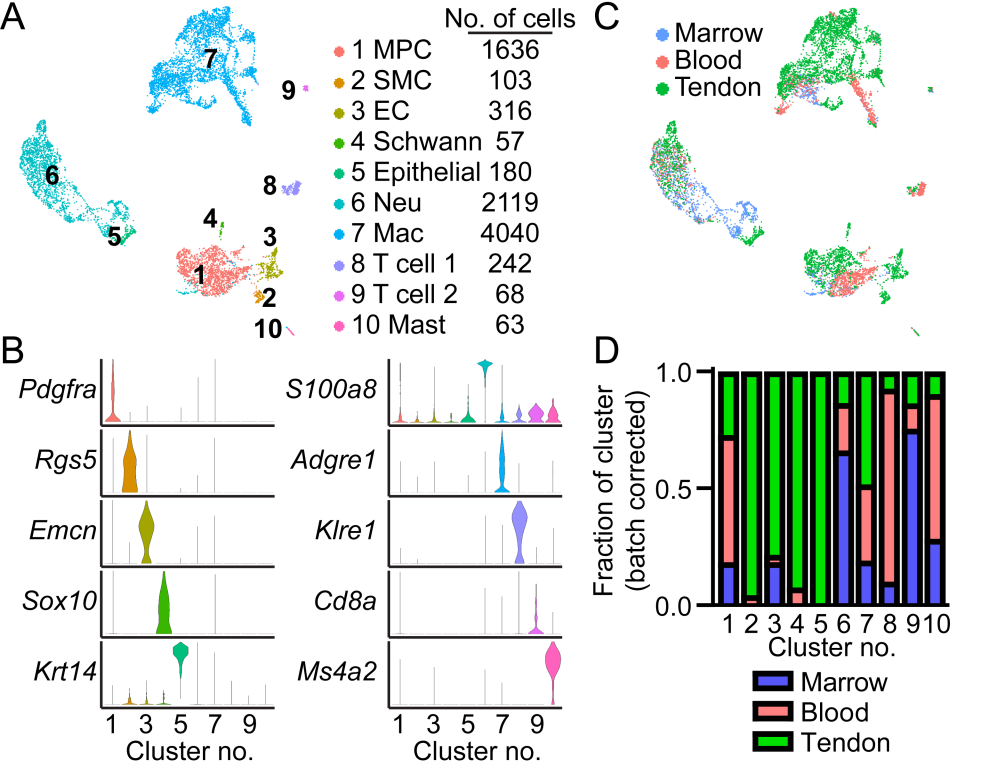

A
No. of cells
1 MPC 1636
2 SMC 103
3 EC 316
4 Schwann 57
5 Epithelial 180
6 Neu 2119
7 Mac 4040
8 T cell 1 242
9 T cell 2 68
10 Mast 63
B
Pdgfra
Rgs5
Emcn
Sox10
Krt14
S100a8
Adgre1
Klre1
Cd8a
Ms4a2
Cluster no.
C
Marrow
Blood
Tendon
D
Fraction of cluster (batch corrected)
1.0
0.5
0.0
Cluster no.
Marrow
Blood
Tendon

Figure S2

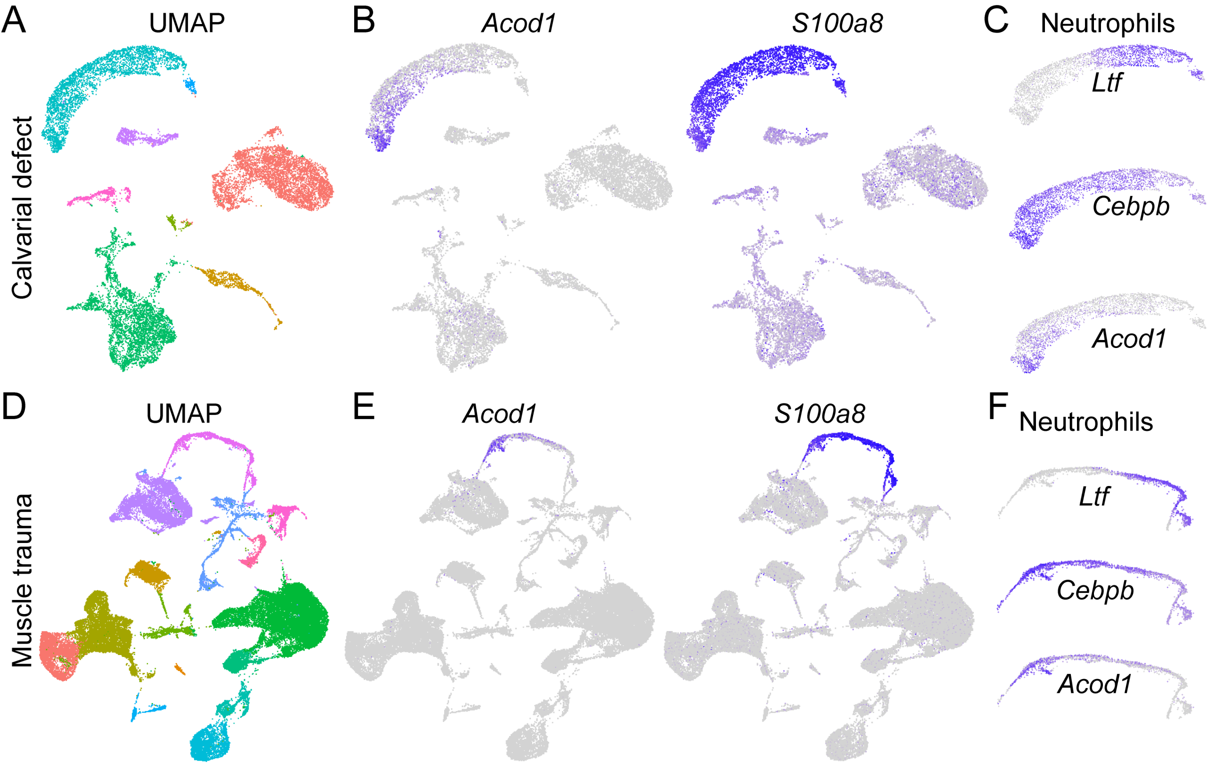
A
UMAP
Calvarial defect
B
Acod1
S100a8
C
Neutrophils
Ltf
Cebpb
Acod1
D
UMAP
Muscle trauma
E
Acod1
S100a8
F
Neutrophils
Ltf
Cebpb
Acod1

Figure S3

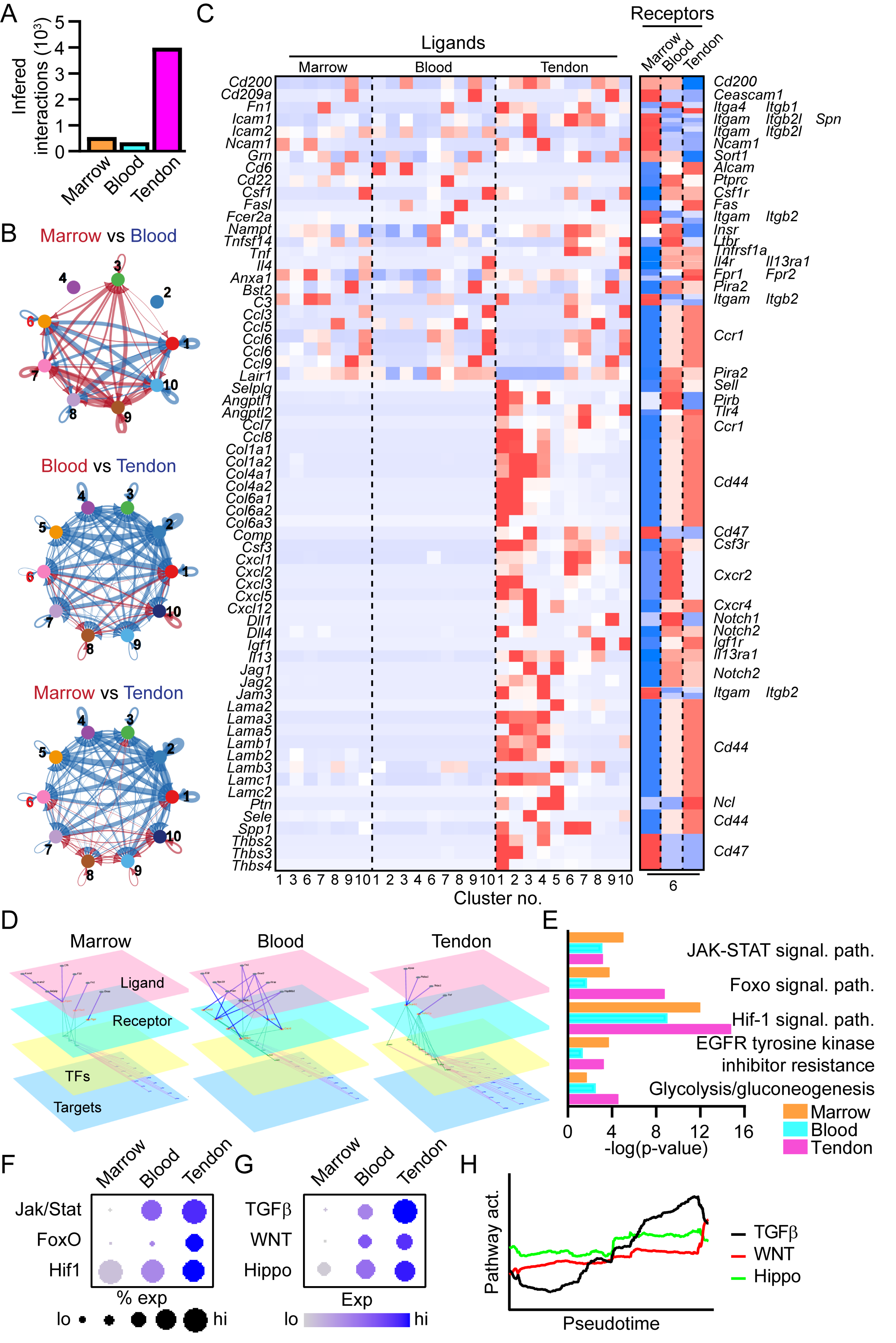
A
Infered interactions (10³)
5
4
3
2
1
0
Marrow
Blood
Tendon
B
Marrow vs Blood
Blood vs Tendon
Marrow vs Tendon
1
2
3
4
5
6
7
8
9
10
C
Ligands
Receptors
Marrow
Blood
Tendon
Cd200
Cd209a
Fn1
Icam1
Icam2
Ncam1
Grn
Cd6
Cd22
Csf1
Fasl
Fcer2a
Nampt
Tnfsf14
Tnf
Il4
Anxa1
Bst2
C3
Ccl3
Ccl5
Ccl6
Ccl6
Ccl9
Lair1
Selplg
Angptl1
Angptl2
Ccl7
Ccl8
Col1a1
Col1a2
Col4a1
Col4a2
Col6a1
Col6a2
Col6a3
Comp
Csf3
Cxcl1
Cxcl2
Cxcl3
Cxcl5
Cxcl12
Dll1
Dll4
Igf1
Il13
Jag1
Jag2
Jam3
Lama2
Lama3
Lama5
Lamb1
Lamb2
Lamb3
Lamc1
Lamc2
Ptn
Sele
Spp1
Thbs2
Thbs3
Thbs4
Cd200
Ceascam1
Itga4 Itgb1
Itgam Itgb2l Spn
Itgam Itgb2l
Ncam1
Sort1
Alcam
Ptprc
Csf1r
Fas
Itgam Itgb2
Insr
Ltbr
Tnfrsf1a
Il4r Il13ra1
Fpr1 Fpr2
Pira2
Itgam Itgb2
Ccr1
Pira2
Sell
Pirb
Tlr4
Ccr1
Cd44
Cd47
Csf3r
Cxcr2
Cxcr4
Notch1
Notch2
Igf1r
Il13ra1
Notch2
Itgam Itgb2
Cd44
Ncl
Cd44
Cd47
1 3 6 7 8 9 10 1 2 3 4 6 7 8 9 10 1 2 3 4 5 6 7 8 9 10
Cluster no.
6
D
Marrow
Blood
Tendon
Ligand
Receptor
TFs
Targets
E
JAK-STAT signal. path.
Foxo signal. path.
Hif-1 signal. path.
EGFR tyrosine kinase inhibitor resistance
Glycolysis/gluconeogenesis
0
4
8
12
16
-log(p-value)
Marrow
Blood
Tendon
F
Marrow
Blood
Tendon
Jak/Stat
FoxO
Hif1
% exp
lo
hi
G
Marrow
Blood
Tendon
TGFβ
WNT
Hippo
Exp
lo
hi
H
Pathway act.
Pseudotime
TGFβ
WNT
Hippo

Figure S4

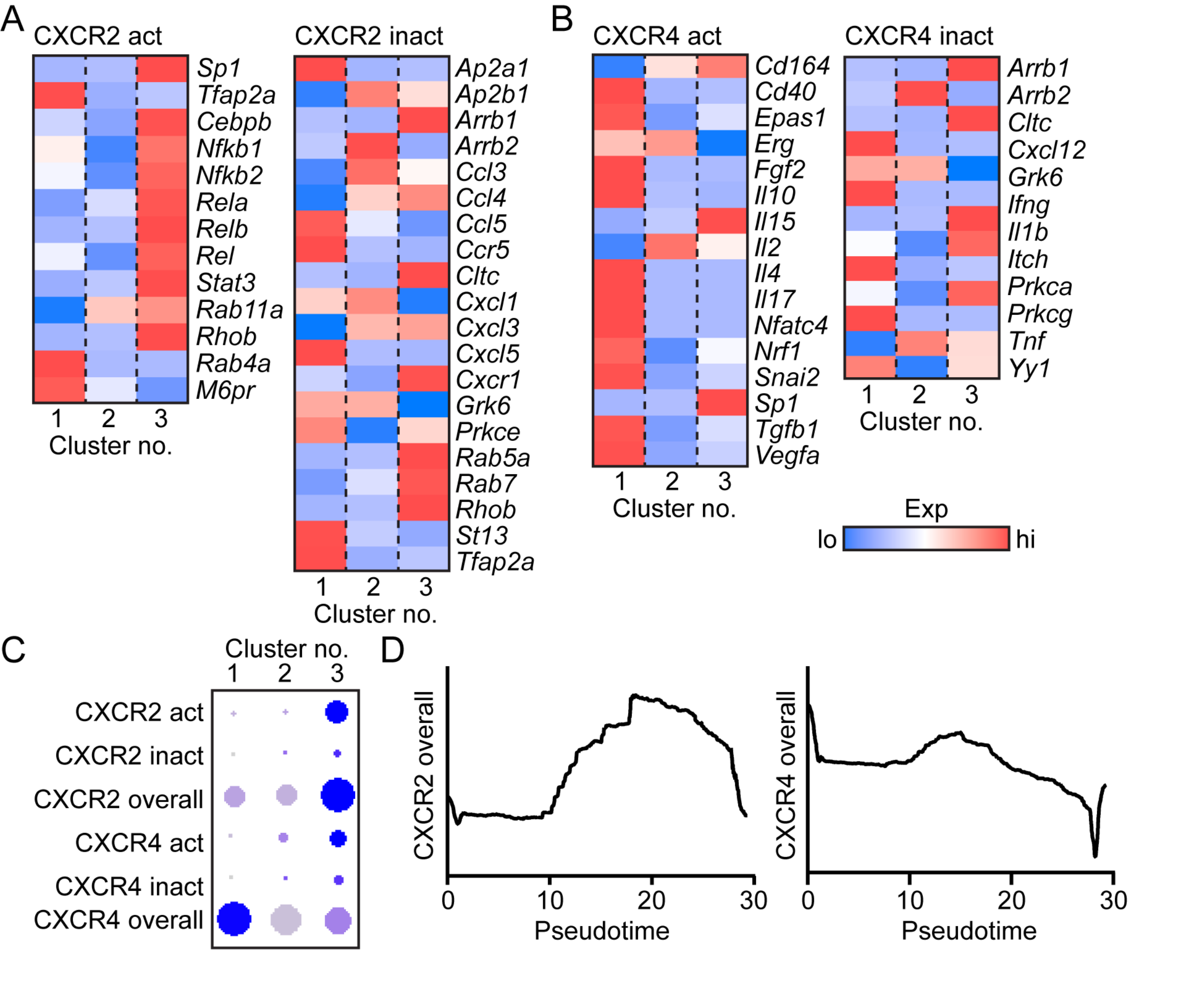
A
CXCR2 act
Sp1
Tfap2a
Cebpb
Nfkb1
Nfkb2
Rela
Relb
Rel
Stat3
Rab11a
Rhob
Rab4a
M6pr
1 2 3
Cluster no.
CXCR2 inact
Ap2a1
Ap2b1
Arrb1
Arrb2
Ccl3
Ccl4
Ccl5
Ccr5
Cltc
Cxcl1
Cxcl3
Cxcl5
Cxcr1
Grk6
Prkce
Rab5a
Rab7
Rhob
St13
Tfap2a
1 2 3
Cluster no.
B
CXCR4 act
Cd164
Cd40
Epas1
Erg
Fgf2
Il10
Il15
Il2
Il4
Il17
Nfatc4
Nrf1
Snai2
Sp1
Tgfb1
Vegfa
1 2 3
Cluster no.
CXCR4 inact
Arrb1
Arrb2
Cltc
Cxcl12
Grk6
Ifng
Il1b
Itch
Prkca
Prkcg
Tnf
Yy1
1 2 3
Cluster no.
Exp
lo
hi
C
Cluster no.
1 2 3
CXCR2 act
CXCR2 inact
CXCR2 overall
CXCR4 act
CXCR4 inact
CXCR4 overall
D
CXCR2 overall
0 10 20 30
Pseudotime
CXCR4 overall
0 10 20 30
Pseudotime

Figure S5

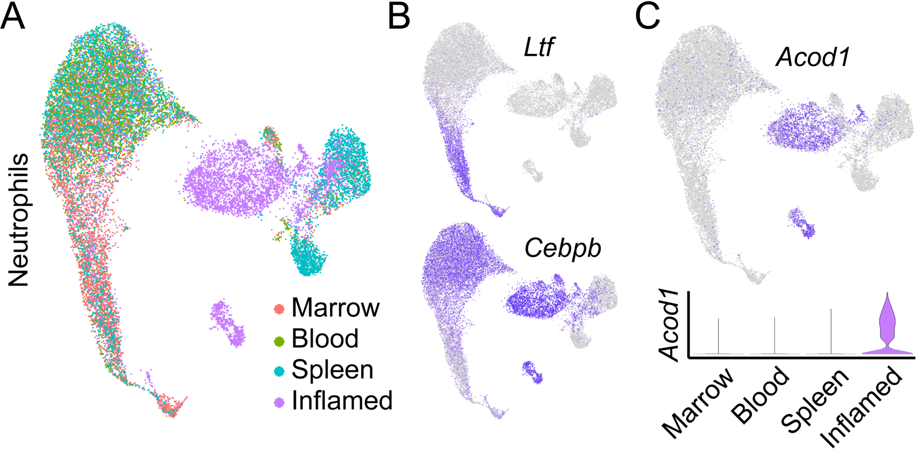
A
Neutrophils
Marrow
Blood
Spleen
Inflamed
B
Ltf
Cebpb
C
Acod1
Acod1
Marrow
Blood
Spleen
Inflamed

Figure S6

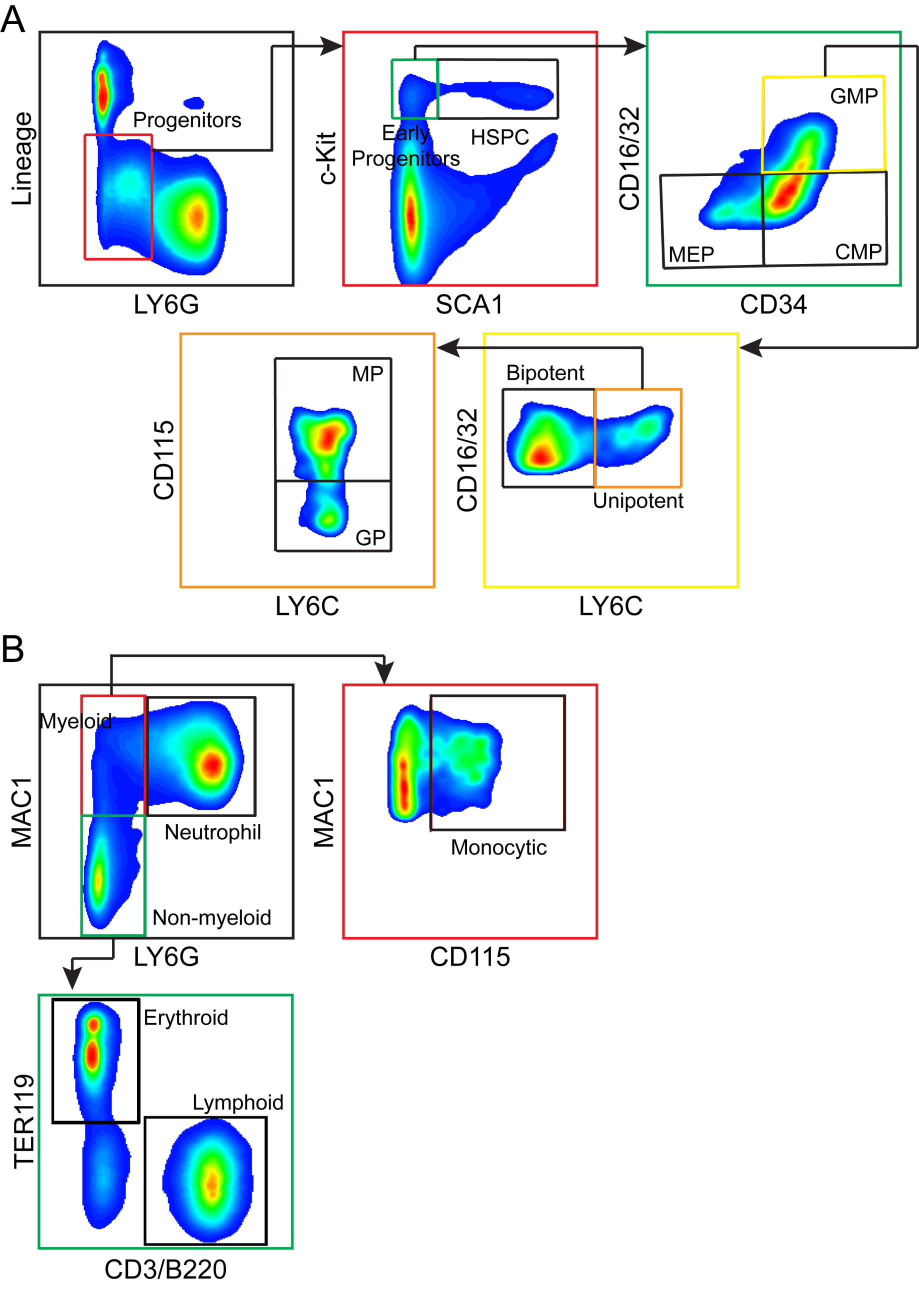
A
Lineage
Progenitors
LY6G
c-Kit
Early
Progenitors
HSPC
SCA1
CD16/32
GMP
MEP
CMP
CD34
CD115
MP
GP
LY6C
CD16/32
Bipotent
Unipotent
LY6C
B
MAC1
Myeloid
Neutrophil
Non-myeloid
LY6G
MAC1
Monocytic
CD115
TER119
Erythroid
Lymphoid
CD3/B220

Figure S7

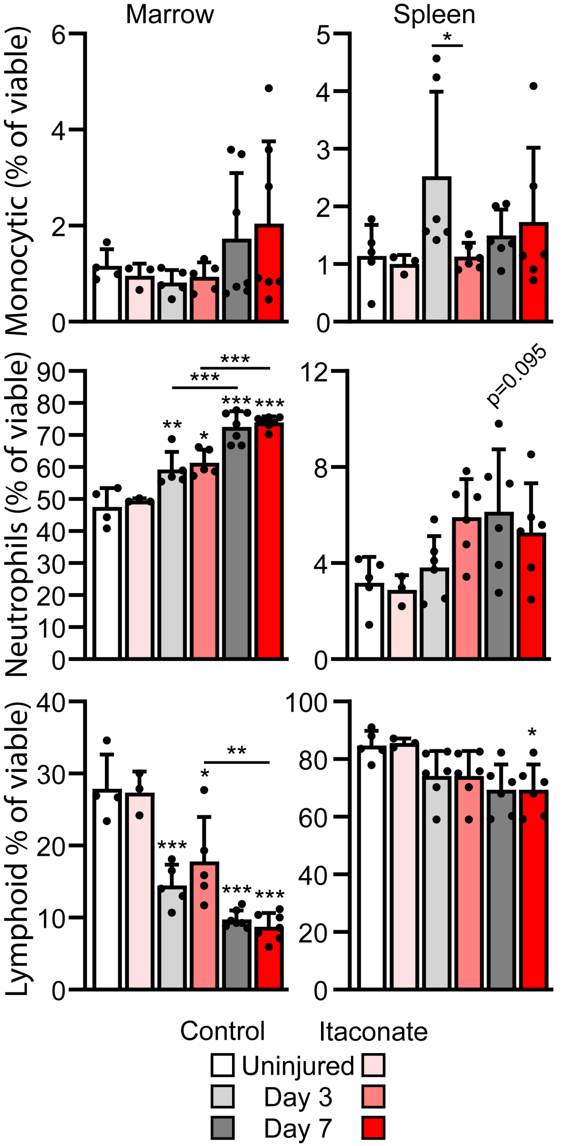

Marrow
Spleen
Monocytic (% of viable)
Neutrophils (% of viable)
Lymphoid % of viable)
p=0.095
Control
Itaconate
Uninjured
Day 3
Day 7

Figure S8

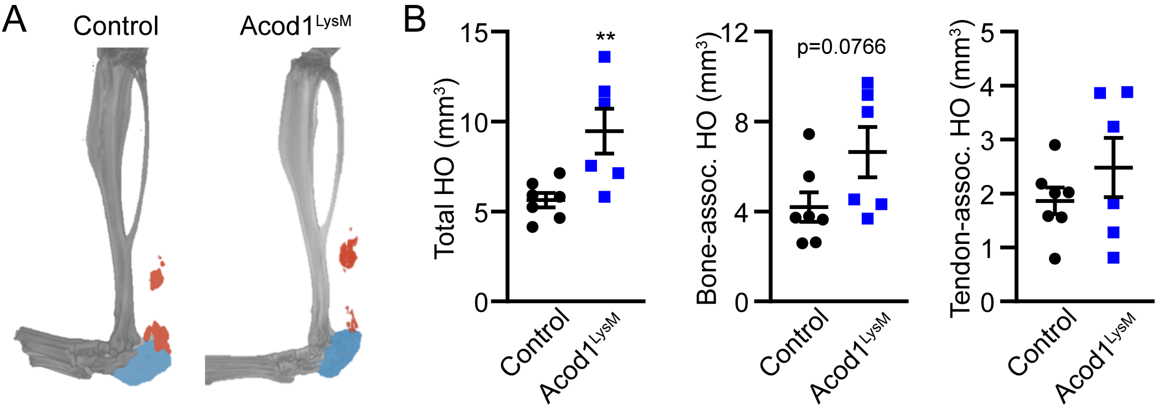

A
Control
Acod1 LysM
B
Total HO (mm3)
15
10
5
0
**
Control
Acod1 LysM
Bone-assoc. HO (mm3)
12
8
4
0
p=0.0766
Control
Acod1 LysM
Tendon-assoc. HO (mm3)
5
4
3
2
1
0
Control
Acod1 LysM

Figure S9

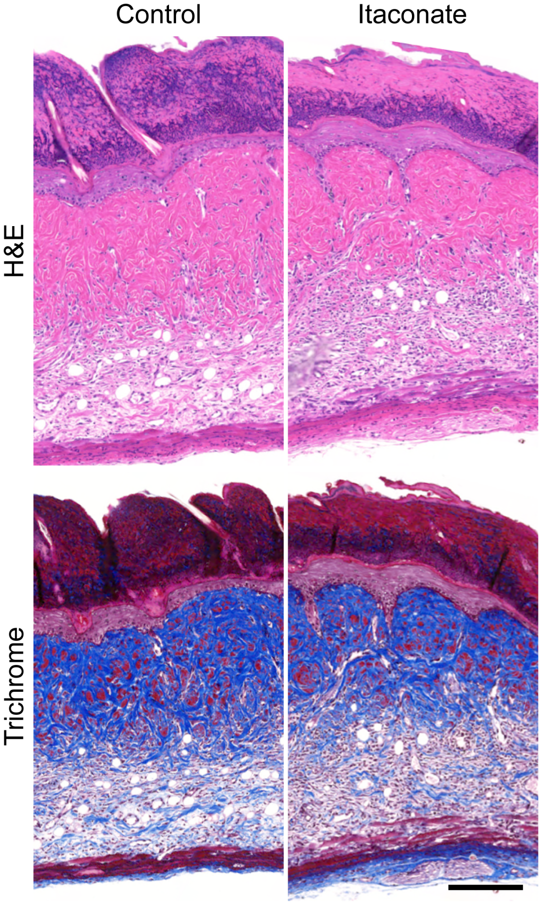

Figure S10
