# Supplementary material for: Itaconate-producing neutrophils regulate local and systemic inflammation following trauma
Source: JCI Insight. 2023 Oct 23;8(20):e169208. doi: 10.1172/jci.insight.169208 (PMC10619500; doi:10.1172/jci.insight.169208)
Supplement: Supplemental tables 1-5 [file jciinsight-8-169208-s116.pdf]

Table S1 Cluster-specific DEGs across combined day 3 data set.

gene	p_val	avg_log2F(	pct.1	pct.2	p_val_adj	cluster
Prg4	5.18E-283	4.55903	0.393	0.076	8.67E-279	1
Dcn	0	4.306431	0.454	0.071	0	1
Col3a1	1.42E-227	4.295922	0.458	0.159	2.38E-223	1
Col1a2	2.63E-298	4.177114	0.454	0.113	4.41E-294	1
Col1a1	2.31E-294	4.17121	0.457	0.117	3.86E-290	1
Timp1	3.92E-152	3.523453	0.448	0.212	6.57E-148	1
Cxcl14	4.08E-184	3.518554	0.336	0.086	6.83E-180	1
Mgp	3.58E-210	3.054406	0.331	0.066	5.99E-206	1
Sparc	3.54E-223	2.998888	0.464	0.157	5.93E-219	1
Bgn	0	2.812584	0.457	0.08	0	1
Cxcl5	1.76E-208	2.705266	0.286	0.049	2.95E-204	1
Fstl1	0	2.662348	0.449	0.075	0	1
Elane	5.16E-10	2.514611	0.101	0.063	8.63E-06	1
Serpinh1	0	2.394217	0.459	0.108	0	1
Prtn3	9.74E-09	2.36479	0.141	0.219	########	1
Mmp3	9.34E-249	2.332073	0.257	0.026	1.56E-244	1
Lox	0	2.279202	0.416	0.037	0	1
Apod	3.46E-288	2.259034	0.288	0.027	5.78E-284	1
Igfbp4	0	2.245809	0.756	0.269	0	1
Col5a2	0	2.213337	0.445	0.053	0	1
Postn	9.20E-250	2.192784	0.322	0.05	1.54E-245	1
Mt2	8.12E-137	2.169614	0.465	0.22	1.36E-132	1
Cxcl12	0	2.009119	0.416	0.056	0	1
Thbs4	4.85E-166	1.968838	0.167	0.015	8.11E-162	1
Lum	0	1.966268	0.408	0.016	0	1
Fbln2	0	1.949598	0.429	0.066	0	1
Mfap5	0	1.925697	0.377	0.026	0	1
Ms4a4b	5.92E-202	1.925555	0.415	0.127	9.90E-198	1
Cd3d	0	1.90497	0.41	0.039	0	1
Serpinf1	0	1.904854	0.459	0.04	0	1
Fbn1	0	1.876463	0.424	0.047	0	1
Saa3	1.01E-08	1.869538	0.203	0.15	########	1
Pcolce	0	1.835846	0.44	0.044	0	1
Serping1	0	1.835114	0.414	0.039	0	1
Igfbp5	1.05E-262	1.806936	0.319	0.043	1.76E-258	1
Mpo	4.82E-33	1.77601	0.116	0.043	8.06E-29	1
Cd3g	0	1.750345	0.409	0.016	0	1
Ly6a	0	1.70918	0.614	0.187	0	1
Selm	5.00E-262	1.697081	0.536	0.182	8.37E-258	1
Il7r	2.34E-262	1.655309	0.386	0.078	3.91E-258	1
Ptx3	0	1.604796	0.336	0.018	0	1
Ccdc80	0	1.5725	0.411	0.056	0	1
Aebp1	0	1.571314	0.435	0.048	0	1
Col5a1	0	1.564172	0.423	0.041	0	1
Mt1	1.46E-29	1.553215	0.566	0.55	2.45E-25	1
Rpl12	0	1.540761	0.998	0.867	0	1
Abi3bp	0	1.512839	0.371	0.013	0	1
Rarres2	0	1.511318	0.358	0.036	0	1
Tnc	4.47E-224	1.498425	0.284	0.042	7.48E-220	1

Lef1	0	1.493301	0.385	0.023	0	1
Mmp2	0	1.483024	0.422	0.032	0	1
Cd8b1	3.41E-285	1.465262	0.212	0.007	5.70E-281	1
Tcf7	0	1.432745	0.4	0.029	0	1
Tnfaip6	0	1.417445	0.377	0.055	0	1
Rcn3	0	1.399734	0.496	0.094	0	1
Cthrc1	0	1.386925	0.354	0.025	0	1
Rpl22l1	0	1.366732	0.987	0.766	0	1
Serpina3n	0	1.342019	0.347	0.023	0	1
Aspn	3.79E-293	1.340943	0.285	0.025	6.35E-289	1
Rplp1	0	1.328003	0.998	0.97	0	1
Col6a1	0	1.31893	0.428	0.037	0	1
Pmepa1	1.14E-269	1.318579	0.484	0.131	1.90E-265	1
Rps27	5.45E-145	1.317604	0.996	0.973	9.11E-141	1
Nedd4	1.38E-236	1.302086	0.592	0.24	2.31E-232	1
Cald1	5.88E-286	1.300014	0.444	0.094	9.84E-282	1
Rpl36a	0	1.281024	0.998	0.84	0	1
Dapl1	5.12E-272	1.278094	0.222	0.012	8.56E-268	1
Fkbp11	0	1.2652	0.466	0.058	0	1
Loxl1	0	1.255569	0.41	0.021	0	1
Sod3	0	1.252858	0.399	0.027	0	1
Cxcl1	5.26E-82	1.251762	0.403	0.204	8.80E-78	1
Col6a3	0	1.251138	0.412	0.033	0	1
Csrp2	1.27E-97	1.24097	0.473	0.252	2.13E-93	1
Plpp3	0	1.23859	0.422	0.071	0	1
Rps15a	0	1.224703	0.999	0.943	0	1
Gas1	0	1.220777	0.395	0.038	0	1
Ogn	0	1.220765	0.322	0.012	0	1
Rps7	0	1.20876	0.998	0.938	0	1
Rps2	0	1.207249	0.998	0.915	0	1
Rps24	1.79E-284	1.201177	0.999	0.969	2.99E-280	1
Tuba1a	1.97E-181	1.199906	0.691	0.409	3.30E-177	1
Rps20	0	1.198916	1	0.922	0	1
Olfml3	0	1.19746	0.431	0.074	0	1
Rps18	0	1.184784	0.999	0.894	0	1
Rpl5	0	1.183844	0.996	0.886	0	1
Npm1	0	1.18272	0.988	0.777	0	1
Eef1b2	0	1.180219	0.996	0.91	0	1
Rps21	0	1.176816	0.999	0.969	0	1
Eef1g	0	1.171542	0.98	0.765	0	1
Il11ra1	6.08E-264	1.156218	0.49	0.14	1.02E-259	1
Slc25a4	2.38E-208	1.152195	0.757	0.466	3.98E-204	1
Igfbp7	1.34E-248	1.133422	0.454	0.108	2.25E-244	1
Thy1	0	1.131565	0.624	0.044	0	1
Tmed3	2.15E-125	1.1254	0.631	0.504	3.59E-121	1
Prrx1	0	1.123296	0.425	0.026	0	1
Lgals1	3.20E-42	1.120377	0.749	0.639	5.35E-38	1
Rpl36	0	1.119324	0.997	0.887	0	1
Rpl39	0	1.119252	0.998	0.951	0	1
Sfrp2	6.90E-272	1.104939	0.208	0.008	1.15E-267	1
Rps28	7.76E-225	1.104583	0.999	0.925	1.30E-220	1
Rpl13a	0	1.104291	0.996	0.956	0	1

Gpx3	3.51E-127	1.1004	0.311	0.095	5.87E-123	1
Dpt	0	1.097577	0.375	0.01	0	1
Rpl15	0	1.09753	0.985	0.794	0	1
Thbs2	0	1.086864	0.367	0.022	0	1
Satb1	2.80E-127	1.082026	0.443	0.2	4.68E-123	1
Rps3a1	0	1.073304	1	0.962	0	1
Col6a2	0	1.071931	0.418	0.027	0	1
Ms4a3	1.30E-08	1.067011	0.103	0.068	########	1
Rpl14	0	1.06437	0.998	0.864	0	1
Maged1	2.48E-295	1.063862	0.43	0.093	4.16E-291	1
Ets1	8.31E-221	1.061593	0.741	0.308	1.39E-216	1
Angptl4	5.94E-291	1.059068	0.428	0.089	9.94E-287	1
Pdgfra	0	1.056788	0.404	0.006	0	1
Rpl30	0	1.054814	0.999	0.971	0	1
Rplp0	0	1.047826	1	0.957	0	1
Meg3	0	1.046238	0.322	0.022	0	1
Rpl35	0	1.044542	0.996	0.886	0	1
Crabp1	6.92E-158	1.040873	0.147	0.011	1.16E-153	1
Rps4x	0	1.040731	0.999	0.926	0	1
Adamts5	0	1.032614	0.356	0.028	0	1
Rpsa	0	1.025526	0.999	0.921	0	1
Hspe1	0	1.023406	0.944	0.616	0	1
Tmsb10	6.28E-189	1.023386	0.992	0.858	1.05E-184	1
Igf1	2.63E-134	1.022912	0.349	0.112	4.39E-130	1
Rps8	0	1.020682	0.999	0.957	0	1
Rpl3	0	1.018089	0.996	0.84	0	1
Cd3e	0	1.016622	0.357	0.013	0	1
Rpl13	0	1.015916	0.999	0.953	0	1
Txk	2.40E-291	1.013247	0.361	0.051	4.01E-287	1
Rps3	0	1.012823	0.998	0.96	0	1
Kdelr2	7.93E-145	1.01263	0.621	0.433	1.33E-140	1
Wisp2	0	1.010716	0.321	0.016	0	1
Rplp2	3.89E-276	1.004825	0.999	0.966	6.51E-272	1
Rpl21	0	1.001015	0.996	0.939	0	1
Rps19	6.12E-294	1.00024	0.998	0.934	1.02E-289	1
Comp	4.12E-162	########	0.137	0.008	6.89E-158	1
Efemp2	0	########	0.428	0.068	0	1
Cilp	7.42E-224	########	0.163	0.005	1.24E-219	1
Il6st	1.38E-245	########	0.588	0.226	2.30E-241	1
Nfix	5.93E-157	########	0.525	0.252	9.93E-153	1
Skap1	0	########	0.375	0.036	0	1
Rps6	0	########	0.998	0.925	0	1
Rps23	0	########	0.998	0.972	0	1
Cd63	8.57E-36	########	0.571	0.594	1.43E-31	1
Bicc1	0	########	0.41	0.021	0	1
Serpinb6a	1.27E-71	########	0.537	0.378	2.13E-67	1
Calu	2.26E-135	########	0.567	0.33	3.78E-131	1
Pdcd4	1.06E-197	########	0.778	0.394	1.77E-193	1
Rpl10a	0	########	0.998	0.846	0	1
Ablim1	0	########	0.551	0.123	0	1
Rpl6	0	########	0.999	0.93	0	1
Ppp1r14b	2.80E-179	########	0.794	0.507	4.69E-175	1

Rpl38	2.15E-246	########	0.997	0.954	3.60E-242	1
Prkcdbp	4.28E-263	########	0.439	0.093	7.16E-259	1
Rpl17	5.10E-274	########	0.998	0.981	8.54E-270	1
Krtcap2	3.10E-244	########	0.892	0.62	5.18E-240	1
Ccnd2	0	########	0.746	0.15	0	1
Rpl23	0	########	1	0.98	0	1
Rpl8	0	########	0.997	0.938	0	1
Rpl11	0	########	0.999	0.941	0	1
Rps16	5.98E-265	########	1	0.987	1.00E-260	1
Cpxm1	0	########	0.326	0.006	0	1
Bcl11b	0	########	0.334	0.036	0	1
Bcl2	2.65E-197	########	0.45	0.133	4.44E-193	1
Sepw1	8.25E-241	########	0.919	0.618	1.38E-236	1
Lck	0	########	0.372	0.04	0	1
Rpl27a	0	########	0.999	0.974	0	1
Rps15	0	########	0.996	0.924	0	1
Mif	9.78E-179	########	0.903	0.625	1.64E-174	1
Pcsk5	0	########	0.326	0.012	0	1
H2-Q7	1.65E-152	########	0.476	0.185	2.77E-148	1
Sfrp1	3.85E-257	########	0.249	0.021	6.44E-253	1
Il1rl1	2.23E-204	########	0.234	0.028	3.72E-200	1
Nenf	4.79E-124	########	0.529	0.296	8.02E-120	1
Gimap3	3.84E-247	########	0.37	0.067	6.43E-243	1
Medag	0	########	0.402	0.025	0	1
Hspd1	1.05E-174	########	0.774	0.488	1.76E-170	1
Gm12840	4.64E-244	########	0.378	0.076	7.76E-240	1
Rpl32	0	########	0.999	0.923	0	1
Rps13	1.32E-295	########	0.998	0.969	2.22E-291	1
Rpl9	0	########	0.999	0.971	0	1
Pdpn	1.07E-232	########	0.41	0.097	1.79E-228	1
Rps29	9.28E-110	########	0.999	0.989	1.55E-105	1
Rpl18	0	########	0.999	0.948	0	1
Rpl27	1.14E-207	########	0.981	0.853	1.92E-203	1
Rpl4	0	########	0.991	0.862	0	1
Gpx8	0	########	0.421	0.034	0	1
Eef1d	1.21E-306	########	0.953	0.685	2.02E-302	1
Rps10	0	########	1	0.979	0	1
Hsp90b1	9.17E-80	########	0.854	0.749	1.53E-75	1
Vkorc1	1.54E-224	########	0.554	0.214	2.57E-220	1
Rpl19	0	########	0.999	0.955	0	1
Ccr7	1.92E-289	########	0.352	0.048	3.20E-285	1
Nfib	9.03E-274	########	0.432	0.087	1.51E-269	1
Nnmt	0	########	0.406	0.033	0	1
Cygb	0	########	0.327	0.019	0	1
Ctsk	0	########	0.421	0.049	0	1
Ppic	0	########	0.487	0.073	0	1
Gm8369	3.20E-203	########	0.337	0.071	5.36E-199	1
S1pr1	1.04E-165	########	0.421	0.137	1.75E-161	1
Rpl37a	9.68E-253	########	1	0.98	1.62E-248	1
Zbtb20	1.33E-182	########	0.606	0.271	2.22E-178	1
Calr	5.46E-92	########	0.878	0.752	9.14E-88	1
Fkbp10	0	########	0.419	0.043	0	1

Manf	7.09E-120	########	0.757	0.568	1.19E-115	1
Rps5	1.19E-305	########	0.998	0.925	1.99E-301	1
Tubb5	2.22E-200	########	0.927	0.637	3.72E-196	1
Ly6c1	0	########	0.447	0.058	0	1
Hmgn1	4.13E-260	########	0.765	0.336	6.92E-256	1
Rps17	0	########	0.996	0.868	0	1
Rpl23a	3.60E-278	########	0.991	0.893	6.02E-274	1
Lat	0	########	0.341	0.027	0	1
Col12a1	0	########	0.341	0.033	0	1
Has1	0	########	0.336	0.014	0	1
Rpl22	1.23E-291	########	0.998	0.904	2.06E-287	1
Mxra8	0	########	0.417	0.038	0	1
Lman1	5.97E-136	########	0.578	0.356	9.99E-132	1
Rpl18a	1.89E-269	########	0.999	0.973	3.17E-265	1
Ran	4.48E-118	########	0.88	0.68	7.50E-114	1
Tbrg1	9.18E-280	########	0.771	0.4	1.54E-275	1
Ssr2	1.01E-135	########	0.673	0.474	1.69E-131	1
Rpl31	0	########	0.996	0.886	0	1
Rpl34	1.18E-273	########	0.999	0.974	1.97E-269	1
1500015O1	0	########	0.232	0.006	0	1
Srm	2.32E-263	########	0.519	0.155	3.88E-259	1
Rpl37	1.73E-250	########	0.999	0.978	2.90E-246	1
Cd248	0	########	0.34	0.017	0	1
Rcn1	0	########	0.513	0.098	0	1
Rpl7	0	########	0.999	0.96	0	1
Col14a1	0	########	0.273	0.018	0	1
Rpl29	1.37E-281	########	0.995	0.892	2.29E-277	1
Ssr4	7.39E-99	########	0.878	0.748	1.24E-94	1
Ostc	4.10E-142	########	0.778	0.618	6.86E-138	1
Ppib	3.58E-146	########	0.95	0.817	5.99E-142	1
Fkbp9	0	########	0.434	0.078	0	1
Rps26	1.38E-220	########	0.998	0.938	2.31E-216	1
Hdlbp	3.03E-69	########	0.658	0.587	5.07E-65	1
Rpl41	0	########	0.999	0.99	0	1
Gpx7	0	########	0.407	0.04	0	1
Uap1	1.63E-114	########	0.495	0.247	2.73E-110	1
Rpl35a	2.02E-208	########	1	0.976	3.38E-204	1
Snrpf	4.49E-234	########	0.94	0.656	7.52E-230	1
Chd3	3.18E-303	########	0.663	0.205	5.33E-299	1
Ppa1	3.10E-236	########	0.598	0.234	5.19E-232	1
Rpl24	0	########	0.999	0.952	0	1
Rps14	3.10E-208	########	0.999	0.981	5.18E-204	1
Gnb2l1	2.33E-249	########	0.996	0.907	3.89E-245	1
Ugdh	1.32E-92	########	0.502	0.306	2.21E-88	1
Rps12	2.74E-272	########	0.998	0.979	4.59E-268	1
Slamf6	4.53E-161	########	0.309	0.076	7.57E-157	1
Dut	5.41E-221	########	0.576	0.201	9.06E-217	1
Snhg18	0	########	0.409	0.031	0	1
Copz2	0	########	0.414	0.071	0	1
Hsp90ab1	1.24E-180	########	0.992	0.9	2.07E-176	1
Eln	8.87E-149	########	0.209	0.033	1.48E-144	1
Dpysl3	0	########	0.388	0.047	0	1

P4hb	5.27E-50	########	0.656	0.646	8.82E-46	1
Il33	0	########	0.287	0.015	0	1
Rps11	2.57E-199	########	0.996	0.959	4.30E-195	1
Cyb5a	5.91E-191	########	0.826	0.513	9.89E-187	1
Tgfbr3	0	########	0.468	0.059	0	1
Dclk1	0	########	0.363	0.018	0	1
Pdia6	7.51E-74	########	0.696	0.591	1.26E-69	1
Smoc2	0	########	0.251	0.008	0	1
Snrpg	1.52E-209	########	0.942	0.65	2.55E-205	1
Smc4	1.07E-149	########	0.719	0.389	1.79E-145	1
Sec61g	1.90E-120	########	0.952	0.847	3.18E-116	1
Nop10	1.90E-219	########	0.881	0.531	3.17E-215	1
Gnas	1.71E-120	########	0.945	0.869	2.86E-116	1
Rpl28	3.27E-264	########	0.999	0.955	5.47E-260	1
Il1r1	2.11E-304	########	0.382	0.058	3.53E-300	1
Rpl26	3.33E-217	########	0.997	0.962	5.56E-213	1
Serpine2	4.42E-221	########	0.329	0.059	7.40E-217	1
Fgfr1	0	########	0.417	0.066	0	1
Prelp	0	########	0.32	0.017	0	1
Slc39a14	1.37E-147	########	0.403	0.15	2.29E-143	1
Mlec	2.69E-196	########	0.699	0.42	4.50E-192	1
Il6	4.91E-218	########	0.319	0.055	8.22E-214	1
Gimap4	9.19E-170	########	0.352	0.09	1.54E-165	1
Erh	1.20E-124	########	0.884	0.679	2.01E-120	1
Rhoc	4.68E-138	########	0.439	0.178	7.82E-134	1
Gas6	3.18E-44	########	0.313	0.16	5.32E-40	1
Nbl1	0	########	0.391	0.043	0	1
Plod2	0	########	0.355	0.023	0	1
Kdelr3	0	########	0.389	0.03	0	1
Igfbp6	2.17E-38	########	0.38	0.231	3.63E-34	1
Ctgf	1.13E-189	########	0.228	0.029	1.89E-185	1
Ncl	4.12E-123	########	0.954	0.796	6.89E-119	1
Kcnq1ot1	1.57E-181	########	0.644	0.277	2.63E-177	1
Clec3b	8.10E-235	########	0.163	0.003	1.35E-230	1
Rbp1	1.21E-223	########	0.308	0.048	2.02E-219	1
Nid1	1.14E-304	########	0.389	0.057	1.90E-300	1
C1qbp	2.86E-206	########	0.708	0.342	4.79E-202	1
Itm2a	0	########	0.344	0.031	0	1
Loxl2	0	########	0.383	0.028	0	1
Fndc1	0	########	0.299	0.008	0	1
Uba52	8.73E-143	########	0.972	0.808	1.46E-138	1
Saraf	3.51E-168	########	0.807	0.446	5.88E-164	1
Cd81	1.14E-113	########	0.58	0.331	1.91E-109	1
Eef1a1	6.52E-238	########	1	0.984	1.09E-233	1
Maged2	0	########	0.41	0.068	0	1
Eprs	4.41E-181	########	0.704	0.401	7.38E-177	1
Serpine1	2.99E-197	########	0.331	0.069	5.00E-193	1
Fkbp3	1.17E-242	########	0.803	0.41	1.97E-238	1
Fkbp7	0	########	0.425	0.061	0	1
Pebp1	1.49E-155	########	0.803	0.579	2.49E-151	1
1-Sep	2.50E-138	########	0.525	0.23	4.19E-134	1
Cyr61	6.20E-275	########	0.377	0.061	1.04E-270	1

Ltb	1.28E-40	########	0.437	0.305	2.15E-36	1
Ift20	6.60E-137	########	0.706	0.503	1.10E-132	1
Pdia3	5.61E-85	########	0.829	0.738	9.39E-81	1
Gm10076	7.48E-191	########	0.987	0.866	1.25E-186	1
Ranbp1	8.87E-113	########	0.785	0.544	1.48E-108	1
Mmp23	0	########	0.345	0.015	0	1
Dad1	7.81E-157	########	0.913	0.699	1.31E-152	1
Srpx2	0	########	0.356	0.009	0	1
Fermt2	1.17E-287	########	0.416	0.072	1.95E-283	1
Tspan4	1.52E-123	########	0.475	0.226	2.54E-119	1
Chl1	1.15E-219	########	0.239	0.026	1.93E-215	1
Naca	4.15E-216	########	0.993	0.888	6.94E-212	1
Cnpy2	6.37E-124	########	0.659	0.467	1.07E-119	1
Col4a1	2.30E-197	########	0.368	0.08	3.84E-193	1
Rrbp1	6.85E-09	########	0.708	0.807	########	1
Hmgb1	1.29E-148	########	0.971	0.86	2.15E-144	1
Klk8	2.00E-234	########	0.307	0.049	3.34E-230	1
Swi5	2.68E-115	########	0.786	0.673	4.49E-111	1
Npdc1	0	########	0.415	0.066	0	1
Wbp5	6.85E-98	########	0.6	0.388	1.15E-93	1
Nop58	1.06E-215	########	0.675	0.297	1.77E-211	1
Lhfp	0	########	0.408	0.035	0	1
Lbp	1.94E-86	########	0.388	0.176	3.25E-82	1
Itk	1.64E-263	########	0.307	0.04	2.75E-259	1
Dgka	8.80E-125	########	0.418	0.177	1.47E-120	1
Cd28	1.08E-245	########	0.26	0.028	1.80E-241	1
Adamts1	1.75E-231	########	0.397	0.083	2.93E-227	1
Rps27a	5.45E-251	########	1	0.987	9.12E-247	1
Twist1	0	########	0.367	0.016	0	1
Ndufa4l2	1.53E-141	########	0.213	0.036	2.56E-137	1
Cd27	1.69E-203	########	0.332	0.07	2.83E-199	1
Adamts2	0	########	0.391	0.02	0	1
Cd247	5.63E-276	########	0.287	0.029	9.42E-272	1
Smad7	1.32E-219	########	0.722	0.317	2.20E-215	1
Serbp1	2.08E-148	########	0.955	0.774	3.48E-144	1
Ppia	8.71E-161	########	0.995	0.884	1.46E-156	1
Crlf3	6.06E-29	########	0.596	0.496	1.01E-24	1
Sec61b	2.83E-89	########	0.958	0.88	4.74E-85	1
Cbx3	5.12E-199	########	0.849	0.504	8.56E-195	1
Dnajc3	8.26E-127	########	0.743	0.552	1.38E-122	1
Gfpt2	0	########	0.359	0.015	0	1
Rapgef6	7.93E-71	########	0.731	0.503	1.33E-66	1
Rpl7a	1.91E-196	########	0.998	0.903	3.19E-192	1
Ifi27	1.04E-116	########	0.7	0.437	1.74E-112	1
2700094K1	3.25E-108	########	0.657	0.418	5.44E-104	1
Set	7.82E-129	########	0.903	0.673	1.31E-124	1
Sar1a	2.88E-86	########	0.642	0.491	4.81E-82	1
Lpar1	0	########	0.386	0.044	0	1
Sptbn1	1.92E-194	########	0.82	0.49	3.22E-190	1
Mrc2	0	########	0.385	0.012	0	1
Atp5g1	7.39E-103	########	0.889	0.697	1.24E-98	1
Vasn	0	########	0.392	0.043	0	1

Snrpd2	7.17E-150	########	0.878	0.653	1.20E-145	1
Txndc5	6.11E-120	########	0.495	0.258	1.02E-115	1
Id3	7.06E-73	########	0.461	0.237	1.18E-68	1
Ackr3	0	########	0.337	0.037	0	1
Tpt1	4.43E-85	########	1	0.998	7.41E-81	1
Dtymk	3.79E-128	########	0.597	0.331	6.35E-124	1
S100a16	3.74E-222	########	0.413	0.09	6.25E-218	1
Cd2	7.10E-178	########	0.333	0.073	1.19E-173	1
Nme1	1.24E-96	########	0.847	0.631	2.07E-92	1
Prkcq	3.97E-248	########	0.277	0.032	6.65E-244	1
Nupr1	5.30E-28	########	0.445	0.332	8.87E-24	1
Eif5a	6.84E-50	########	0.905	0.854	1.15E-45	1
Gm11808	1.50E-134	########	0.817	0.486	2.51E-130	1
Cd8a	1.26E-220	########	0.17	0.007	2.10E-216	1
Ikbip	1.97E-227	########	0.452	0.131	3.29E-223	1
Fam189b	0	########	0.422	0.062	0	1
Park7	6.67E-151	########	0.896	0.693	1.12E-146	1
Bmp1	0	########	0.378	0.035	0	1
Oat	8.39E-110	########	0.583	0.356	1.40E-105	1
Fam162a	2.37E-132	########	0.638	0.352	3.96E-128	1
Rexo2	3.17E-155	########	0.752	0.456	5.30E-151	1
Ptgis	4.60E-281	########	0.213	0.008	7.69E-277	1
Sfrp4	5.45E-175	########	0.126	0.003	9.13E-171	1
Tubb2a	3.99E-50	########	0.46	0.316	6.68E-46	1
Ybx3	2.12E-74	########	0.573	0.394	3.54E-70	1
Tceal8	1.28E-129	########	0.482	0.226	2.15E-125	1
Egfr	0	########	0.381	0.035	0	1
Eva1b	1.44E-161	########	0.513	0.208	2.41E-157	1
Prdx4	1.20E-70	########	0.5	0.326	2.01E-66	1
Ptprcap	7.41E-159	########	0.441	0.137	1.24E-154	1
Eif3e	5.86E-146	########	0.934	0.689	9.81E-142	1
Gimap1	2.24E-107	########	0.367	0.133	3.75E-103	1
Tmed9	1.36E-112	########	0.799	0.644	2.27E-108	1
Fgf7	0	########	0.323	0.013	0	1
Kbtbd11	2.55E-247	########	0.295	0.039	4.26E-243	1
Hspg2	1.05E-278	########	0.406	0.071	1.77E-274	1
Rps25	4.28E-163	########	0.998	0.96	7.16E-159	1
mt-Nd2	2.96E-134	########	0.992	0.87	4.95E-130	1
Cct3	3.10E-138	########	0.74	0.457	5.19E-134	1
Srpk1	2.51E-180	########	0.723	0.367	4.19E-176	1
Csgalnact1	0	########	0.362	0.034	0	1
Vimp	4.61E-110	########	0.733	0.552	7.71E-106	1
C1qtnf6	0	########	0.375	0.045	0	1
mt-Cytb	1.93E-186	########	0.995	0.958	3.23E-182	1
Klf9	1.05E-129	########	0.478	0.207	1.76E-125	1
Rpl36al	6.59E-128	########	0.99	0.808	1.10E-123	1
Clmp	2.81E-293	########	0.378	0.062	4.70E-289	1
Col5a3	0	########	0.317	0.028	0	1
Ryk	5.77E-270	########	0.403	0.077	9.65E-266	1
Fbln1	0	########	0.35	0.023	0	1
Grb10	0	########	0.377	0.041	0	1
Ddb1	3.09E-104	########	0.6	0.385	5.17E-100	1

Pik3ip1	1.58E-229	########	0.382	0.08	2.65E-225	1
Sdc2	0	########	0.388	0.03	0	1
Gm10073	3.28E-135	########	0.787	0.513	5.48E-131	1
Nucks1	4.14E-132	########	0.813	0.528	6.93E-128	1
Dpm3	1.72E-141	########	0.809	0.553	2.87E-137	1
Fst	4.28E-305	########	0.3	0.027	7.16E-301	1
Antxr1	0	########	0.374	0.02	0	1
Nap1l1	3.66E-74	########	0.806	0.64	6.13E-70	1
C1s1	0	########	0.279	0.019	0	1
Peli1	3.89E-52	########	0.684	0.481	6.51E-48	1
Ndufa5	3.84E-144	########	0.788	0.515	6.42E-140	1
Eif2s2	6.46E-120	########	0.906	0.736	1.08E-115	1
Ptrf	4.83E-210	########	0.422	0.104	8.08E-206	1
Phgdh	0	########	0.501	0.093	0	1
Mxra7	0	########	0.391	0.044	0	1
Tpm1	2.19E-137	########	0.524	0.232	3.66E-133	1
Gdf10	2.96E-254	########	0.181	0.004	4.95E-250	1
Sec61a1	1.02E-77	########	0.63	0.466	1.70E-73	1
Col8a1	2.01E-166	########	0.171	0.016	3.37E-162	1
Ddr2	0	########	0.381	0.025	0	1
Gpm6b	0	########	0.32	0.022	0	1
Tbca	7.97E-123	########	0.881	0.684	1.33E-118	1
Plxdc2	6.50E-286	########	0.342	0.045	1.09E-281	1
Gramd3	1.69E-297	########	0.435	0.079	2.83E-293	1
Svep1	0	########	0.273	0.005	0	1
Erdr1	9.22E-138	########	0.689	0.357	1.54E-133	1
Dpep1	0	########	0.284	0.004	0	1
Emilin1	1.04E-161	########	0.479	0.187	1.74E-157	1
Fxyd1	0	########	0.326	0.027	0	1
Galk1	2.35E-184	########	0.5	0.186	3.94E-180	1
Cdk4	6.40E-128	########	0.638	0.362	1.07E-123	1
Gtf2i	1.45E-230	########	0.634	0.228	2.42E-226	1
Ltbp2	0	########	0.232	0.004	0	1
Scn7a	4.05E-276	########	0.208	0.008	6.78E-272	1
Lrrc59	2.46E-55	########	0.548	0.407	4.12E-51	1
Rhoj	0	########	0.408	0.061	0	1
Rps27rt	5.16E-117	########	0.634	0.326	8.64E-113	1
2810428I1!	3.22E-83	########	0.704	0.521	5.38E-79	1
Eef2	9.04E-138	########	0.976	0.874	1.51E-133	1
Ccl2	1.01E-13	########	0.378	0.293	1.69E-09	1
Arf4	4.01E-55	########	0.709	0.622	6.72E-51	1
Ddost	9.25E-94	########	0.691	0.517	1.55E-89	1
Eif3i	1.46E-125	########	0.883	0.652	2.44E-121	1
Mfap4	1.49E-222	########	0.156	0.003	2.49E-218	1
H19	2.76E-129	########	0.123	0.009	4.63E-125	1
Angptl2	0	########	0.347	0.028	0	1
Impdh2	1.66E-145	########	0.668	0.358	2.78E-141	1
Eny2	2.08E-124	########	0.694	0.447	3.48E-120	1
Fam78a	2.82E-135	########	0.263	0.067	4.71E-131	1
Tm4sf1	4.05E-216	########	0.371	0.074	6.78E-212	1
Slc16a1	4.60E-235	########	0.441	0.11	7.69E-231	1
Uqcc2	1.85E-89	########	0.716	0.499	3.09E-85	1

Inpp4b	8.15E-117	########	0.256	0.071	1.36E-112	1
Gem	1.86E-190	########	0.376	0.092	3.12E-186	1
Prmt1	3.69E-113	########	0.651	0.386	6.18E-109	1
Slc16a2	0	########	0.368	0.027	0	1
Nsg2	0	########	0.22	0.004	0	1
Sulf1	0	########	0.355	0.023	0	1
Surf4	2.44E-48	########	0.632	0.568	4.09E-44	1
Arl1	3.62E-82	########	0.644	0.487	6.05E-78	1
Ndufa4	5.04E-81	########	0.957	0.803	8.44E-77	1
Gstm2	0	########	0.344	0.04	0	1
Snrpd1	2.39E-78	########	0.748	0.534	3.99E-74	1
Creb3l1	0	########	0.358	0.045	0	1
Tomm5	2.77E-119	########	0.755	0.514	4.63E-115	1
Rpn1	7.29E-61	########	0.627	0.502	1.22E-56	1
Bola2	9.18E-111	########	0.885	0.623	1.54E-106	1
Psip1	9.03E-212	########	0.68	0.272	1.51E-207	1
Fxyd6	2.99E-236	########	0.241	0.023	4.99E-232	1
Nudcd2	1.53E-161	########	0.583	0.283	2.55E-157	1
Morf4l2	2.95E-61	########	0.633	0.494	4.94E-57	1
Themis	1.02E-301	########	0.217	0.006	1.70E-297	1
Tuba1b	1.00E-71	########	0.784	0.56	1.68E-67	1
Anp32b	1.49E-65	########	0.88	0.713	2.50E-61	1
Nsa2	1.13E-82	########	0.946	0.777	1.89E-78	1
Npm3	1.16E-171	########	0.7	0.344	1.94E-167	1
Slit3	0	########	0.339	0.017	0	1
Psmd7	2.55E-84	########	0.656	0.472	4.26E-80	1
Cks1b	3.13E-123	########	0.476	0.203	5.24E-119	1
2410015M2	1.38E-141	########	0.858	0.643	2.30E-137	1
Plpp1	1.05E-290	########	0.371	0.055	1.75E-286	1
Vcam1	2.79E-234	########	0.377	0.075	4.67E-230	1
Sec31a	5.20E-114	########	0.604	0.359	8.70E-110	1
Shisa5	9.26E-20	########	0.851	0.726	1.55E-15	1
Lamb1	9.35E-272	########	0.333	0.046	1.56E-267	1
Nrip1	1.06E-151	########	0.575	0.248	1.78E-147	1
Crtap	5.12E-191	########	0.446	0.141	8.57E-187	1
Has2	0	########	0.274	0.01	0	1
Snrpd3	7.78E-111	########	0.847	0.633	1.30E-106	1
Limd2	1.03E-27	########	0.763	0.639	1.72E-23	1
Nop56	4.87E-160	########	0.661	0.317	8.15E-156	1
Timm13	5.65E-88	########	0.845	0.663	9.45E-84	1
Dnajc25	4.87E-178	########	0.462	0.164	8.14E-174	1
Frmd6	1.28E-306	########	0.42	0.073	2.15E-302	1
Lama4	0	########	0.361	0.043	0	1
Aes	9.69E-90	########	0.913	0.704	1.62E-85	1
Oaf	1.62E-212	########	0.403	0.098	2.70E-208	1
Sec11a	3.03E-135	########	0.801	0.564	5.07E-131	1
Cct7	9.92E-100	########	0.788	0.586	1.66E-95	1
Efemp1	1.46E-269	########	0.204	0.008	2.44E-265	1
Cdh11	0	########	0.292	0.007	0	1
Tmem263	2.19E-253	########	0.459	0.116	3.67E-249	1
Tln2	7.50E-303	########	0.35	0.046	1.26E-298	1
Pdap1	4.97E-76	########	0.738	0.601	8.32E-72	1

Lrrc17	0	########	0.251	0.003	0	1
Pdgfrb	0	########	0.361	0.028	0	1
Pa2g4	1.33E-103	########	0.647	0.389	2.23E-99	1
Tmem97	1.20E-161	########	0.423	0.148	2.01E-157	1
Ttc3	3.01E-250	########	0.541	0.157	5.03E-246	1
Bola3	2.89E-170	########	0.632	0.289	4.84E-166	1
Prrx2	0	########	0.317	0.005	0	1
Rabac1	7.53E-106	########	0.922	0.806	1.26E-101	1
Cnn3	3.05E-229	########	0.427	0.095	5.10E-225	1
Axl	1.02E-247	########	0.388	0.077	1.70E-243	1
Nhp2	1.10E-87	########	0.685	0.449	1.84E-83	1
Cdkn1c	1.14E-105	########	0.19	0.039	1.90E-101	1
Gja1	8.09E-87	########	0.385	0.164	1.35E-82	1
Denr	1.71E-127	########	0.699	0.436	2.87E-123	1
Gspt1	3.61E-93	########	0.693	0.467	6.03E-89	1
Hnrnpa0	1.90E-114	########	0.938	0.774	3.18E-110	1
P3h1	0	########	0.384	0.047	0	1
Rab34	0	########	0.385	0.057	0	1
Adam12	0	########	0.318	0.015	0	1
Rsl1d1	8.69E-136	########	0.671	0.362	1.45E-131	1
Ube2s	7.49E-56	########	0.802	0.669	1.25E-51	1
Atp5g2	2.44E-87	########	0.965	0.783	4.09E-83	1
Mydgf	1.60E-63	########	0.632	0.491	2.68E-59	1
Bzw2	5.53E-198	########	0.564	0.202	9.25E-194	1
Pam	8.69E-86	########	0.468	0.245	1.45E-81	1
Rhoh	1.72E-71	########	0.387	0.196	2.87E-67	1
Sned1	2.45E-230	########	0.231	0.021	4.10E-226	1
Entpd2	6.49E-303	########	0.24	0.011	1.09E-298	1
Emg1	2.50E-127	########	0.803	0.514	4.18E-123	1
Aldh1a2	4.60E-169	########	0.14	0.007	7.69E-165	1
Loxl3	0	########	0.343	0.019	0	1
Ndufab1	1.78E-86	########	0.759	0.559	2.98E-82	1
Lyar	2.92E-108	########	0.548	0.289	4.89E-104	1
Gimap6	5.16E-95	########	0.359	0.134	8.64E-91	1
Mfap2	0	########	0.26	0.013	0	1
Rbbp7	7.83E-135	########	0.658	0.356	1.31E-130	1
Lsm4	1.66E-98	########	0.841	0.652	2.78E-94	1
Mmp14	2.58E-66	########	0.408	0.213	4.32E-62	1
Gstm5	2.28E-292	########	0.429	0.08	3.81E-288	1
Rpl10	3.13E-83	########	0.993	0.943	5.23E-79	1
Snrpe	4.62E-90	########	0.939	0.742	7.73E-86	1
Tnxb	2.94E-252	########	0.235	0.018	4.91E-248	1
Gnl3	1.57E-143	########	0.578	0.263	2.63E-139	1
Srpx	0	########	0.28	0.009	0	1
Ddx18	7.55E-160	########	0.65	0.303	1.26E-155	1
Cdc42ep5	0	########	0.37	0.047	0	1
Htra3	0	########	0.279	0.016	0	1
Tubb4b	2.18E-59	########	0.716	0.551	3.65E-55	1
Mrps16	7.07E-107	########	0.737	0.524	1.18E-102	1
Parva	2.88E-292	########	0.388	0.063	4.82E-288	1
Ifi47	1.75E-64	########	0.389	0.201	2.93E-60	1
Nfia	2.29E-134	########	0.385	0.129	3.83E-130	1

Copb2	7.83E-58	########	0.673	0.566	1.31E-53	1
Nolc1	6.94E-151	########	0.554	0.238	1.16E-146	1
mt-Nd1	8.14E-99	########	0.993	0.887	1.36E-94	1
Mrpl17	1.38E-71	########	0.609	0.434	2.31E-67	1
Snhg6	1.96E-161	########	0.61	0.286	3.28E-157	1
Hspa5	9.29E-14	########	0.815	0.842	1.55E-09	1
Ndn	0	########	0.351	0.023	0	1
Cd34	5.06E-252	########	0.355	0.055	8.46E-248	1
Hint1	1.81E-61	########	0.95	0.753	3.03E-57	1
Cyb5r3	1.64E-52	########	0.517	0.35	2.75E-48	1
Sh2d1a	1.34E-192	########	0.207	0.021	2.25E-188	1
Laptm4a	7.73E-29	########	0.693	0.633	1.29E-24	1
Sfr1	1.57E-52	########	0.776	0.689	2.63E-48	1
Colec12	0	########	0.362	0.026	0	1
Etfb	3.42E-26	########	0.661	0.592	5.73E-22	1
Tspan11	0	########	0.285	0.003	0	1
mt-Nd3	4.76E-102	########	0.987	0.815	7.96E-98	1
Rnase4	8.90E-30	########	0.432	0.306	1.49E-25	1
Ifi205	1.34E-111	########	0.2	0.042	2.24E-107	1
Col16a1	0	########	0.348	0.02	0	1
Srp19	1.33E-87	########	0.725	0.557	2.23E-83	1
P4ha2	0	########	0.329	0.032	0	1
Pcolce2	0	########	0.276	0.004	0	1
Ckap4	4.58E-09	########	0.529	0.541	7.67E-05	1
Gadd45gip	7.12E-145	########	0.654	0.33	1.19E-140	1
Hnrnpa1	1.35E-81	########	0.873	0.641	2.25E-77	1
Med21	1.40E-59	########	0.595	0.383	2.35E-55	1
Cacybp	1.66E-74	########	0.693	0.494	2.77E-70	1
Trib2	4.46E-189	########	0.232	0.031	7.46E-185	1
Skp1a	8.00E-126	########	0.774	0.488	1.34E-121	1
Tcp1	3.33E-79	########	0.742	0.541	5.58E-75	1
Tmem258	4.32E-71	########	0.844	0.73	7.24E-67	1
Ssb	3.63E-92	########	0.839	0.662	6.07E-88	1
Itgbl1	9.08E-296	########	0.191	0.002	1.52E-291	1
Rdx	3.55E-95	########	0.739	0.515	5.94E-91	1
mt-Nd4l	2.32E-89	########	0.941	0.708	3.87E-85	1
Spcs2	1.87E-69	########	0.857	0.697	3.14E-65	1
Dkc1	2.32E-176	########	0.517	0.188	3.88E-172	1
Ssr1	2.16E-52	########	0.668	0.565	3.61E-48	1
Ost4	1.02E-68	########	0.872	0.778	1.70E-64	1
Pdk1	5.14E-137	########	0.406	0.14	8.60E-133	1
Lamc1	8.04E-139	########	0.391	0.133	1.34E-134	1
Myc	4.12E-254	########	0.431	0.093	6.89E-250	1
Olfml2b	1.10E-126	########	0.371	0.126	1.84E-122	1
Errfi1	7.66E-48	########	0.408	0.236	1.28E-43	1
Col4a2	3.35E-181	########	0.336	0.07	5.60E-177	1
Stub1	4.13E-90	########	0.653	0.444	6.92E-86	1
Eif4g1	7.20E-68	########	0.688	0.508	1.20E-63	1
Fubp1	7.81E-109	########	0.765	0.47	1.31E-104	1
C1ra	3.90E-275	########	0.314	0.038	6.53E-271	1
Auts2	0	########	0.393	0.054	0	1
Ldha	2.88E-29	########	0.877	0.831	4.83E-25	1

Ptma	2.09E-51	########	0.997	0.843	3.50E-47	1
Rbp4	0	########	0.25	0.002	0	1
Ddx21	1.04E-95	########	0.737	0.459	1.74E-91	1
Ccl11	7.71E-163	########	0.149	0.01	1.29E-158	1
mt-Atp6	3.58E-118	########	0.998	0.988	5.99E-114	1
Mycbp2	3.32E-73	########	0.812	0.617	5.55E-69	1
Kdelr1	2.36E-59	########	0.66	0.54	3.95E-55	1
Gimap9	2.43E-84	########	0.32	0.128	4.06E-80	1
Vcan	2.72E-38	########	0.402	0.252	4.56E-34	1
Igsf10	0	########	0.312	0.008	0	1
Plat	8.00E-299	########	0.318	0.034	1.34E-294	1
Tmem167	4.58E-51	########	0.762	0.694	7.67E-47	1
Cope	1.68E-49	########	0.716	0.619	2.80E-45	1
Mrpl12	9.01E-86	########	0.641	0.409	1.51E-81	1
Sec13	5.02E-69	########	0.598	0.424	8.40E-65	1
Pura	9.46E-124	########	0.765	0.459	1.58E-119	1
Tsn	1.52E-96	########	0.752	0.55	2.54E-92	1
Wwtr1	7.73E-250	########	0.394	0.073	1.29E-245	1
Higd1a	4.93E-55	########	0.718	0.558	8.26E-51	1
Dnm3os	0	########	0.289	0.011	0	1
Ddah2	2.38E-132	########	0.355	0.11	3.98E-128	1
Lmna	1.56E-09	########	0.46	0.45	2.61E-05	1
Wdr43	2.18E-136	########	0.586	0.267	3.65E-132	1
Fkbp1a	1.27E-103	########	0.764	0.513	2.12E-99	1
Cct2	7.57E-86	########	0.853	0.64	1.27E-81	1
Slc50a1	1.24E-145	########	0.639	0.314	2.07E-141	1
Bsg	8.92E-32	########	0.727	0.702	1.49E-27	1
Crlf1	7.75E-206	########	0.218	0.021	1.30E-201	1
P3h3	0	########	0.364	0.036	0	1
Timp3	1.38E-100	########	0.244	0.066	2.30E-96	1
Banf1	3.23E-91	########	0.757	0.508	5.41E-87	1
Gxylt2	0	########	0.275	0.015	0	1
Gm4955	3.94E-78	########	0.215	0.068	6.59E-74	1
Nudc	1.82E-77	########	0.724	0.497	3.04E-73	1
Pole4	2.78E-141	########	0.68	0.348	4.66E-137	1
Cct5	3.60E-75	########	0.842	0.655	6.02E-71	1
Fam114a1	6.00E-161	########	0.399	0.126	1.00E-156	1
Zeb1	3.59E-209	########	0.523	0.16	6.01E-205	1
Mrps28	3.07E-115	########	0.613	0.321	5.13E-111	1
Romo1	7.66E-75	########	0.81	0.661	1.28E-70	1
Fam92a	1.66E-253	########	0.468	0.115	2.78E-249	1
Chchd1	1.41E-88	########	0.731	0.502	2.35E-84	1
Lamb2	1.62E-275	########	0.338	0.046	2.72E-271	1
Nkg7	2.78E-106	########	0.289	0.091	4.65E-102	1
Fbl	9.54E-100	########	0.727	0.441	1.60E-95	1
Figf	0	########	0.268	0.014	0	1
Rwdd1	6.53E-71	########	0.754	0.577	1.09E-66	1
Sesn3	1.15E-178	########	0.402	0.111	1.92E-174	1
Cd320	1.04E-274	########	0.406	0.077	1.74E-270	1
1810022K0	6.30E-91	########	0.751	0.505	1.05E-86	1
P3h4	0	########	0.354	0.038	0	1
Sfxn1	1.09E-127	########	0.548	0.264	1.83E-123	1

Ereg	1.96E-148	########	0.145	0.012	3.27E-144	1
Psma3	5.98E-64	########	0.874	0.753	1.00E-59	1
Nsg1	0	########	0.312	0.021	0	1
Dst	7.31E-151	########	0.402	0.125	1.22E-146	1
Scml4	2.92E-188	########	0.218	0.025	4.88E-184	1
Amotl2	1.92E-236	########	0.328	0.055	3.22E-232	1
Aimp1	5.98E-85	########	0.791	0.568	1.00E-80	1
Prss23	3.13E-182	########	0.274	0.048	5.23E-178	1
Sec62	1.28E-90	########	0.877	0.74	2.14E-86	1
Minos1	8.78E-59	########	0.837	0.757	1.47E-54	1
Yif1b	2.21E-87	########	0.556	0.336	3.69E-83	1
Rbms3	0	########	0.331	0.025	0	1
Lsm6	1.84E-58	########	0.698	0.525	3.08E-54	1
Ptov1	2.12E-86	########	0.479	0.257	3.55E-82	1
Fzd2	0	########	0.294	0.016	0	1
Cycs	2.47E-52	########	0.739	0.562	4.13E-48	1
Dph5	1.13E-182	########	0.441	0.133	1.90E-178	1
Glrx5	4.00E-94	########	0.675	0.422	6.69E-90	1
Cd5	8.26E-192	########	0.166	0.01	1.38E-187	1
Pydc3	1.42E-125	########	0.198	0.036	2.37E-121	1
Ssrp1	9.11E-86	########	0.649	0.409	1.52E-81	1
Cct4	1.63E-78	########	0.807	0.599	2.72E-74	1
Arl4c	1.48E-23	########	0.54	0.408	2.47E-19	1
Tshz2	1.07E-285	########	0.35	0.046	1.78E-281	1
Stt3a	4.40E-68	########	0.583	0.401	7.36E-64	1
Rpn2	7.88E-59	########	0.69	0.54	1.32E-54	1
Mrfap1	6.00E-48	########	0.773	0.687	1.00E-43	1
Fkbp4	2.33E-124	########	0.606	0.299	3.90E-120	1
Atp5o	9.43E-65	########	0.88	0.727	1.58E-60	1
Thoc7	3.15E-90	########	0.674	0.431	5.27E-86	1
Il11	2.47E-135	########	0.152	0.017	4.13E-131	1
Mtch1	1.59E-25	########	0.636	0.585	2.66E-21	1
Yipf5	7.65E-96	########	0.547	0.307	1.28E-91	1
Ms4a4d	0	########	0.245	0.007	0	1
Pdgfrl	3.21E-266	########	0.231	0.015	5.36E-262	1
Atp5b	1.25E-66	########	0.927	0.825	2.09E-62	1
2700060E0	4.68E-70	########	0.828	0.656	7.82E-66	1
Hspa9	3.30E-74	########	0.629	0.395	5.52E-70	1
Cd4	3.41E-114	########	0.139	0.018	5.71E-110	1
Htra1	6.32E-177	########	0.295	0.056	1.06E-172	1
Hsp90aa1	4.91E-30	########	0.883	0.791	8.21E-26	1
Ebna1bp2	8.68E-79	########	0.621	0.405	1.45E-74	1
Srsf7	3.43E-54	########	0.65	0.467	5.74E-50	1
Tead1	1.59E-294	########	0.36	0.05	2.66E-290	1
Cttn	1.84E-234	########	0.368	0.071	3.08E-230	1
Prkca	2.88E-285	########	0.425	0.075	4.81E-281	1
Adm	4.13E-162	########	0.197	0.025	6.92E-158	1
Srpr	2.60E-71	########	0.658	0.486	4.36E-67	1
Bcl7c	1.38E-77	########	0.597	0.396	2.30E-73	1
Gstp1	3.77E-140	########	0.585	0.265	6.31E-136	1
Zfhx4	0	########	0.315	0.007	0	1
Nt5dc2	1.29E-39	########	0.411	0.261	2.15E-35	1

Rasgrp1	2.33E-100	########	0.207	0.051	3.89E-96	1
Ccdc34	2.38E-80	########	0.472	0.243	3.97E-76	1
Nhp2l1	9.08E-71	########	0.768	0.557	1.52E-66	1
Ddx39b	2.17E-91	########	0.759	0.503	3.64E-87	1
Clec11a	3.04E-256	########	0.24	0.019	5.09E-252	1
Lrp1	2.55E-07	########	0.472	0.459	########	1
Eif1ax	3.79E-82	########	0.715	0.507	6.34E-78	1
Praf2	1.85E-239	########	0.377	0.076	3.09E-235	1
Atp1b3	1.33E-22	########	0.857	0.68	2.23E-18	1
Sf3b5	6.02E-58	########	0.787	0.67	1.01E-53	1
Cnbp	1.89E-75	########	0.859	0.676	3.17E-71	1
Wdr12	8.95E-148	########	0.468	0.17	1.50E-143	1
Mettl1	2.02E-245	########	0.488	0.125	3.39E-241	1
Glrx3	1.53E-71	########	0.696	0.495	2.55E-67	1
Tyms	3.45E-114	########	0.417	0.162	5.77E-110	1
Tmed1	2.44E-200	########	0.425	0.119	4.08E-196	1
Tubb2b	4.63E-247	########	0.303	0.04	7.74E-243	1
Eif3m	7.98E-78	########	0.899	0.672	1.33E-73	1
Ndufa12	7.45E-76	########	0.749	0.528	1.25E-71	1
Gna11	7.74E-174	########	0.449	0.147	1.29E-169	1
Fzd1	0	########	0.318	0.027	0	1
Ltbp3	6.72E-272	########	0.312	0.038	1.12E-267	1
Fndc3b	1.48E-36	0.341593	0.47	0.337	2.47E-32	1
Ssr3	6.32E-35	########	0.685	0.642	1.06E-30	1
Tecpr1	3.29E-73	########	0.331	0.148	5.50E-69	1
Cct8	1.04E-62	########	0.812	0.627	1.73E-58	1
Alg5	5.04E-94	########	0.586	0.346	8.43E-90	1
Tnfrsf12a	6.63E-25	########	0.4	0.287	1.11E-20	1
Mybbp1a	2.60E-110	########	0.568	0.282	4.35E-106	1
Sars	2.92E-98	########	0.679	0.395	4.89E-94	1
Tmed2	5.12E-51	########	0.832	0.773	8.57E-47	1
Lsm5	2.71E-76	########	0.724	0.493	4.53E-72	1
Tnfsf8	1.58E-248	########	0.188	0.007	2.65E-244	1
Gars	8.87E-93	########	0.54	0.301	1.48E-88	1
Serf1	1.57E-241	########	0.43	0.098	2.62E-237	1
Ssbp1	1.71E-87	########	0.676	0.426	2.85E-83	1
Abce1	4.89E-114	########	0.565	0.282	8.18E-110	1
Angptl1	1.20E-248	########	0.174	0.004	2.00E-244	1
Smc6	2.18E-104	########	0.746	0.436	3.65E-100	1
Clpp	2.90E-144	########	0.515	0.215	4.86E-140	1
Snrpa1	2.81E-88	########	0.582	0.336	4.70E-84	1
6-Sep	5.15E-121	########	0.462	0.182	8.62E-117	1
Polr2f	4.23E-69	########	0.729	0.529	7.08E-65	1
Pdia4	2.65E-98	########	0.578	0.318	4.44E-94	1
Phb2	2.13E-56	########	0.743	0.565	3.56E-52	1
Klhdc2	1.45E-145	########	0.592	0.266	2.42E-141	1
Ptgfrn	1.82E-196	########	0.359	0.082	3.04E-192	1
Gar1	2.50E-162	########	0.496	0.18	4.18E-158	1
Tgfb2	0	########	0.281	0.018	0	1
Yif1a	3.01E-83	########	0.517	0.293	5.04E-79	1
Mrps34	3.80E-114	########	0.627	0.339	6.36E-110	1
Imp3	8.39E-100	########	0.743	0.443	1.40E-95	1

Sumo2	3.23E-64	########	0.904	0.756	5.41E-60	1
Birc5	2.70E-55	########	0.321	0.15	4.51E-51	1
Ecm1	8.09E-12	########	0.446	0.365	1.35E-07	1
Prss57	2.10E-10	########	0.105	0.065	3.51E-06	1
Mbd3	6.15E-93	########	0.61	0.358	1.03E-88	1
Hnrnpab	2.38E-48	########	0.803	0.637	3.97E-44	1
Flrt2	3.12E-266	########	0.29	0.032	5.23E-262	1
Cox7c	3.22E-53	########	0.991	0.904	5.39E-49	1
Rora	1.89E-140	########	0.339	0.097	3.16E-136	1
Dnajc1	5.47E-103	########	0.594	0.325	9.14E-99	1
Tomm20	2.72E-65	########	0.911	0.725	4.56E-61	1
Mrps26	1.12E-90	########	0.617	0.364	1.88E-86	1
Pdcd5	6.16E-59	########	0.781	0.627	1.03E-54	1
Cfdp1	2.13E-78	########	0.669	0.45	3.57E-74	1
Hnrnpa3	1.17E-54	########	0.943	0.811	1.97E-50	1
Copg1	3.80E-109	########	0.518	0.26	6.36E-105	1
Hacd1	6.21E-202	########	0.415	0.112	1.04E-197	1
15-Sep	1.35E-48	########	0.846	0.763	2.25E-44	1
Pnn	3.43E-72	########	0.704	0.468	5.75E-68	1
Tesc	1.52E-78	########	0.152	0.036	2.55E-74	1
Psma4	2.81E-44	########	0.79	0.674	4.69E-40	1
Snhg12	4.05E-160	########	0.526	0.195	6.77E-156	1
Ddx1	9.79E-109	########	0.532	0.268	1.64E-104	1
Exosc8	9.49E-95	########	0.579	0.314	1.59E-90	1
Btf3	4.66E-60	########	0.982	0.905	7.80E-56	1
Wdr89	2.15E-106	########	0.656	0.346	3.59E-102	1
Atic	1.07E-146	########	0.498	0.192	1.78E-142	1
Ilf2	7.03E-123	########	0.564	0.268	1.18E-118	1
Eif3h	1.25E-67	########	0.943	0.772	2.10E-63	1
Pcdh19	2.86E-284	########	0.279	0.025	4.78E-280	1
Slc43a3	6.27E-170	########	0.383	0.106	1.05E-165	1
Atf5	7.75E-165	########	0.384	0.112	1.30E-160	1
Mbnl1	2.07E-23	########	0.932	0.773	3.47E-19	1
Rps27l	6.65E-24	########	0.806	0.73	1.11E-19	1
Gorasp2	9.23E-70	########	0.534	0.339	1.55E-65	1
Tceb2	4.43E-43	########	0.953	0.9	7.41E-39	1
Pkdcc	1.51E-250	########	0.294	0.037	2.52E-246	1
Slc39a7	1.12E-67	########	0.531	0.335	1.87E-63	1
Nucb2	6.00E-47	########	0.506	0.349	1.00E-42	1
Mrto4	4.30E-72	########	0.528	0.311	7.19E-68	1
Hp1bp3	1.12E-67	########	0.75	0.512	1.87E-63	1
Lsm2	2.37E-70	########	0.553	0.329	3.96E-66	1
Podn	3.17E-231	########	0.166	0.004	5.31E-227	1
Pmm1	1.03E-167	########	0.463	0.155	1.72E-163	1
Ptn	2.74E-102	########	0.149	0.024	4.58E-98	1
Fam102a	2.66E-112	########	0.292	0.089	4.46E-108	1
Idh2	3.18E-66	########	0.528	0.317	5.32E-62	1
Fundc2	3.67E-83	########	0.71	0.448	6.13E-79	1
Smco4	8.45E-249	########	0.457	0.108	1.41E-244	1
Mrpl15	4.53E-85	########	0.631	0.389	7.58E-81	1
Sod2	1.73E-08	########	0.658	0.598	########	1
Anxa6	1.10E-80	########	0.638	0.361	1.84E-76	1

Bag3	1.99E-70	########	0.411	0.21	3.33E-66	1
Rer1	2.44E-59	########	0.796	0.692	4.09E-55	1
Ift27	1.30E-151	########	0.513	0.204	2.17E-147	1
Polr2m	3.05E-111	########	0.651	0.348	5.10E-107	1
Abhd14a	2.11E-264	########	0.422	0.085	3.53E-260	1
Col11a1	1.46E-164	########	0.112	0.002	2.45E-160	1
Rbfox2	8.56E-256	########	0.35	0.057	1.43E-251	1
Adgrd1	0	########	0.239	0.005	0	1
Dap3	4.77E-92	########	0.609	0.362	7.98E-88	1
Pfdn1	4.95E-76	########	0.661	0.43	8.29E-72	1
Mesdc2	7.69E-138	########	0.519	0.221	1.29E-133	1
Wisp1	0	########	0.257	0.013	0	1
Mrps24	5.16E-55	########	0.79	0.623	8.64E-51	1
Cav1	5.77E-163	########	0.39	0.107	9.66E-159	1
Gpatch4	1.56E-177	########	0.433	0.128	2.61E-173	1
Ppid	6.22E-91	########	0.518	0.271	1.04E-86	1
Zranb2	3.29E-82	########	0.697	0.43	5.51E-78	1
Erlec1	1.42E-83	########	0.566	0.328	2.37E-79	1
Prrc1	6.39E-139	########	0.461	0.181	1.07E-134	1
Trip6	1.20E-228	########	0.369	0.076	2.01E-224	1
Tmem45a	0	########	0.323	0.027	0	1
Mllt3	8.47E-140	########	0.303	0.079	1.42E-135	1
Siva1	2.67E-27	########	0.577	0.47	4.47E-23	1
Uchl3	1.02E-93	########	0.574	0.308	1.71E-89	1
Hmgn3	8.69E-180	########	0.389	0.102	1.45E-175	1
Tomm40	1.34E-69	########	0.548	0.332	2.24E-65	1
Sema3c	2.98E-227	########	0.24	0.024	4.99E-223	1
Tars	8.21E-105	########	0.476	0.22	1.37E-100	1
Foxp1	2.11E-77	########	0.853	0.623	3.53E-73	1
Islr	1.04E-221	########	0.186	0.01	1.75E-217	1
Hypk	3.52E-84	########	0.688	0.43	5.89E-80	1
Pla2g16	1.09E-163	########	0.477	0.154	1.82E-159	1
Tnik	1.48E-156	########	0.224	0.036	2.47E-152	1
Hivep2	4.00E-102	########	0.558	0.267	6.70E-98	1
Gnpnat1	6.04E-209	########	0.462	0.131	1.01E-204	1
Ndufs5	2.94E-42	########	0.795	0.72	4.93E-38	1
Ptges3	3.33E-47	########	0.765	0.616	5.57E-43	1
Osmr	3.35E-254	########	0.342	0.053	5.61E-250	1
Ngfrap1	2.37E-111	########	0.482	0.209	3.97E-107	1
Ubl4a	1.55E-82	########	0.548	0.313	2.59E-78	1
Cyth3	8.32E-115	########	0.418	0.161	1.39E-110	1
Cyp1b1	3.84E-265	########	0.197	0.007	6.43E-261	1
Sidt1	6.25E-153	########	0.144	0.011	1.05E-148	1
Ddt	1.19E-91	########	0.628	0.354	2.00E-87	1
Pls3	8.03E-189	########	0.38	0.091	1.34E-184	1
Mrpl18	8.58E-40	########	0.691	0.538	1.44E-35	1
Pros1	2.33E-71	########	0.381	0.182	3.89E-67	1
Prrc2c	3.70E-45	########	0.894	0.767	6.19E-41	1
Trp53	3.52E-84	########	0.657	0.389	5.90E-80	1
Eif3g	3.69E-75	########	0.751	0.506	6.18E-71	1
Zc3h15	4.77E-67	########	0.724	0.53	7.98E-63	1
Gpr153	0	########	0.304	0.008	0	1

Dnajc9	2.37E-103	########	0.524	0.245	3.97E-99	1
Aars	4.39E-91	########	0.595	0.332	7.34E-87	1
2810004N2	8.01E-121	########	0.529	0.237	1.34E-116	1
Ccl7	4.59E-48	########	0.359	0.186	7.68E-44	1
Pdha1	5.60E-80	########	0.65	0.411	9.36E-76	1
Srsf3	4.10E-46	########	0.895	0.758	6.85E-42	1
Lima1	8.95E-51	########	0.407	0.236	1.50E-46	1
Dap	3.66E-32	########	0.622	0.528	6.12E-28	1
Llph	5.36E-54	########	0.816	0.622	8.96E-50	1
Anapc5	1.39E-72	########	0.664	0.439	2.33E-68	1
Fibin	6.23E-180	########	0.12	0.002	1.04E-175	1
U2af1	3.21E-43	########	0.715	0.571	5.38E-39	1
Rps12-ps3	1.74E-66	########	0.718	0.509	2.92E-62	1
Map1b	5.16E-181	########	0.314	0.063	8.63E-177	1
Acp1	2.39E-53	########	0.62	0.45	4.00E-49	1
Naa10	2.18E-80	########	0.61	0.358	3.65E-76	1
Fam46a	1.75E-41	########	0.615	0.443	2.93E-37	1
Sh3pxd2a	6.25E-111	########	0.392	0.149	1.05E-106	1
F3	2.33E-187	########	0.245	0.035	3.90E-183	1
Man1a2	8.76E-86	########	0.628	0.367	1.47E-81	1
Steap1	0	########	0.271	0.009	0	1
Sgk1	6.96E-23	########	0.489	0.378	1.16E-18	1
Scara3	0	########	0.27	0.01	0	1
Cp	1.35E-124	########	0.174	0.026	2.25E-120	1
Fam169b	2.22E-130	########	0.192	0.032	3.71E-126	1
2310039H(	1.06E-102	########	0.512	0.244	1.78E-98	1
Dnaja1	1.03E-68	########	0.921	0.833	1.73E-64	1
Lix1l	2.41E-248	########	0.35	0.058	4.04E-244	1
Hdgf	8.50E-48	########	0.704	0.546	1.42E-43	1
Hspb8	1.12E-281	########	0.311	0.036	1.87E-277	1
Cbx5	8.62E-110	########	0.506	0.225	1.44E-105	1
Ddah1	1.28E-150	########	0.198	0.028	2.15E-146	1
Dctpp1	3.42E-52	########	0.491	0.307	5.72E-48	1
Wdr75	1.49E-159	########	0.422	0.136	2.49E-155	1
Churc1	2.08E-58	########	0.726	0.49	3.49E-54	1
Ssbp2	8.11E-139	########	0.432	0.15	1.36E-134	1
Utrn	3.34E-92	########	0.547	0.274	5.59E-88	1
Dlc1	2.77E-231	########	0.328	0.052	4.63E-227	1
Svbp	6.25E-60	########	0.631	0.415	1.05E-55	1
Mrps5	4.17E-111	########	0.521	0.24	6.98E-107	1
Tsen34	5.72E-62	########	0.51	0.312	9.58E-58	1
Abhd8	4.18E-192	########	0.353	0.079	6.99E-188	1
mt-Nd4	1.46E-63	########	0.994	0.954	2.44E-59	1
Fscn1	3.29E-190	########	0.309	0.059	5.51E-186	1
Camk4	2.19E-191	########	0.167	0.01	3.66E-187	1
Steap4	2.28E-109	########	0.312	0.094	3.82E-105	1
Lsm3	7.62E-56	########	0.603	0.406	1.27E-51	1
F2rl1	1.86E-221	########	0.203	0.015	3.11E-217	1
Ndufb2	8.32E-46	########	0.74	0.575	1.39E-41	1
Scd2	2.84E-120	########	0.507	0.22	4.76E-116	1
Bcat1	0	########	0.271	0.006	0	1
Col27a1	0	########	0.276	0.016	0	1

Suclg1	6.79E-61	########	0.696	0.498	1.14E-56	1
Ifi203	8.45E-32	########	0.485	0.331	1.41E-27	1
Spcs1	4.35E-31	########	0.741	0.67	7.28E-27	1
Gadd45g	1.28E-55	########	0.411	0.218	2.14E-51	1
Txnl4a	5.80E-81	########	0.562	0.324	9.71E-77	1
Mrpl21	3.94E-69	########	0.661	0.425	6.59E-65	1
Dnlz	4.70E-95	########	0.498	0.246	7.86E-91	1
Ndrg3	5.91E-120	########	0.368	0.126	9.89E-116	1
Taf1d	1.51E-71	########	0.671	0.42	2.53E-67	1
Dynll2	2.74E-85	########	0.513	0.273	4.58E-81	1
Slc1a5	7.18E-111	########	0.518	0.237	1.20E-106	1
Hes1	3.77E-48	########	0.389	0.213	6.31E-44	1
Zcchc7	2.14E-75	########	0.663	0.387	3.58E-71	1
Pole3	8.07E-106	########	0.498	0.239	1.35E-101	1
Acat1	3.60E-74	########	0.602	0.369	6.03E-70	1
Sae1	1.74E-106	########	0.536	0.258	2.91E-102	1
Rdh10	4.62E-98	########	0.266	0.082	7.72E-94	1
Gpr180	3.49E-188	########	0.404	0.11	5.84E-184	1
Adamtsl1	0	########	0.277	0.01	0	1
Ttc28	4.91E-209	########	0.334	0.063	8.22E-205	1
Eif2s1	4.12E-56	########	0.649	0.457	6.89E-52	1
Kmt2a	3.79E-66	########	0.655	0.4	6.34E-62	1
P4ha1	4.57E-76	########	0.518	0.282	7.65E-72	1
Cnih1	5.14E-71	########	0.567	0.351	8.59E-67	1
Pabpc1	1.97E-64	########	0.968	0.916	3.30E-60	1
Tra2b	1.17E-59	########	0.834	0.61	1.96E-55	1
Nckap1	6.12E-211	########	0.37	0.077	1.02E-206	1
Mrpl30	1.66E-54	########	0.831	0.639	2.78E-50	1
Esf1	6.79E-93	########	0.588	0.309	1.14E-88	1
Adk	5.30E-113	########	0.481	0.215	8.87E-109	1
Trmt112	7.27E-58	########	0.801	0.584	1.22E-53	1
mt-Atp8	6.34E-46	########	0.893	0.698	1.06E-41	1
Leprotl1	7.62E-40	########	0.786	0.589	1.28E-35	1
Ict1	8.67E-72	########	0.662	0.423	1.45E-67	1
Bola1	8.68E-99	########	0.507	0.244	1.45E-94	1
Tspan6	1.24E-286	########	0.324	0.038	2.08E-282	1
Eif3j1	8.20E-58	########	0.7	0.49	1.37E-53	1
Pam16	7.06E-87	########	0.567	0.309	1.18E-82	1
Nop14	9.87E-111	########	0.489	0.211	1.65E-106	1
Metap2	1.82E-36	########	0.741	0.615	3.04E-32	1
Ctps	1.05E-189	########	0.364	0.088	1.76E-185	1
Shox2	0	########	0.265	0.01	0	1
Foxo1	4.64E-89	########	0.388	0.162	7.76E-85	1
Arsi	0	########	0.228	0.006	0	1
Tmem119	2.67E-232	########	0.309	0.045	4.46E-228	1
Cep97	8.75E-141	########	0.224	0.042	1.46E-136	1
Gmppb	7.77E-132	########	0.409	0.15	1.30E-127	1
Mphosph1(	4.29E-71	########	0.51	0.285	7.18E-67	1
Tspan32	2.09E-12	########	0.314	0.254	3.49E-08	1
Mrpl13	5.88E-77	########	0.573	0.337	9.83E-73	1
Pabpc4	4.36E-194	########	0.443	0.129	7.30E-190	1
Ccdc53	4.76E-77	########	0.633	0.373	7.96E-73	1

Alyref	4.89E-33	########	0.602	0.452	8.18E-29	1
Tpr	9.39E-32	########	0.881	0.767	1.57E-27	1
Slc38a10	1.34E-55	########	0.501	0.312	2.24E-51	1
Cdk5rap3	5.85E-96	########	0.543	0.276	9.79E-92	1
Sec24d	1.13E-129	########	0.383	0.132	1.90E-125	1
Kpnb1	4.24E-55	########	0.601	0.401	7.09E-51	1
Smim11	8.38E-67	########	0.659	0.433	1.40E-62	1
Ccdc3	9.63E-107	########	0.141	0.02	1.61E-102	1
Sec23a	3.82E-143	########	0.403	0.136	6.39E-139	1
Hnrnpr	1.11E-70	########	0.682	0.424	1.85E-66	1
Nudt21	4.49E-59	########	0.678	0.469	7.50E-55	1
Grap2	1.80E-129	########	0.159	0.02	3.01E-125	1
Tgfbr2	1.42E-48	########	0.585	0.372	2.38E-44	1
Zfp260	1.29E-138	########	0.406	0.135	2.16E-134	1
Mrpl51	2.73E-44	########	0.652	0.499	4.56E-40	1
Dag1	1.61E-108	########	0.432	0.176	2.69E-104	1
Ak1	0	########	0.309	0.024	0	1
Sra1	5.54E-44	########	0.702	0.576	9.27E-40	1
Stt3b	5.08E-76	########	0.621	0.389	8.50E-72	1
C4b	4.31E-101	########	0.213	0.051	7.22E-97	1
Atp5g3	3.85E-44	########	0.853	0.722	6.44E-40	1
Prnp	8.97E-118	########	0.402	0.146	1.50E-113	1
Eif4a2	1.26E-51	########	0.799	0.565	2.11E-47	1
Steap3	3.50E-116	########	0.336	0.109	5.85E-112	1
Eif3b	1.87E-51	########	0.583	0.399	3.12E-47	1
Rasal3	4.17E-59	########	0.254	0.108	6.98E-55	1
Spon1	3.26E-200	########	0.225	0.025	5.45E-196	1
Top2b	2.59E-51	########	0.753	0.535	4.34E-47	1
Pgp	2.86E-41	########	0.568	0.415	4.79E-37	1
Brix1	2.57E-97	########	0.523	0.258	4.30E-93	1
Chpf	0	########	0.333	0.031	0	1
Cd69	5.55E-58	########	0.297	0.133	9.29E-54	1
Ftsj3	1.59E-99	########	0.442	0.192	2.65E-95	1
Mtap	1.59E-155	########	0.411	0.129	2.66E-151	1
Cd96	1.21E-164	########	0.145	0.009	2.03E-160	1
Fads3	1.21E-236	########	0.318	0.048	2.03E-232	1
Pop5	7.95E-70	########	0.619	0.357	1.33E-65	1
Txnl1	3.14E-37	########	0.677	0.567	5.25E-33	1
Arhgap29	1.49E-198	########	0.312	0.056	2.49E-194	1
Twist2	0	########	0.251	0.008	0	1
Gm10020	2.36E-111	########	0.472	0.199	3.94E-107	1
Rrp15	1.95E-141	########	0.447	0.16	3.26E-137	1
Tipin	4.00E-86	########	0.423	0.19	6.69E-82	1
Mrpl42	4.03E-54	########	0.639	0.428	6.74E-50	1
Fam3c	6.05E-112	########	0.568	0.27	1.01E-107	1
Guk1	1.74E-75	########	0.57	0.341	2.92E-71	1
Bmper	0	########	0.237	0.003	0	1
Klhdc1	5.47E-135	########	0.197	0.034	9.16E-131	1
Gimap8	5.70E-69	########	0.202	0.064	9.54E-65	1
Rcn2	1.46E-88	########	0.479	0.238	2.44E-84	1
Grpel1	1.33E-48	########	0.67	0.504	2.23E-44	1
Hdac2	5.34E-101	########	0.51	0.246	8.93E-97	1

Cdk1	2.56E-51	########	0.292	0.134	4.28E-47	1
Fdx1l	6.95E-95	########	0.531	0.265	1.16E-90	1
Ass1	3.44E-67	########	0.26	0.102	5.76E-63	1
Ndufa11	3.33E-31	########	0.762	0.666	5.58E-27	1
Cenpw	5.00E-81	########	0.351	0.152	8.37E-77	1
Klf2	1.60E-07	########	0.802	0.768	########	1
Hspb11	1.75E-86	########	0.484	0.236	2.93E-82	1
Mprip	1.47E-88	########	0.537	0.281	2.47E-84	1
Gm2000	1.11E-107	########	0.53	0.242	1.86E-103	1
Ddx39	2.51E-45	########	0.553	0.375	4.19E-41	1
BC094916	1.09E-38	########	0.174	0.075	1.83E-34	1
Dsel	7.94E-264	########	0.307	0.038	1.33E-259	1
Pigp	2.61E-129	########	0.517	0.209	4.37E-125	1
Srsf1	1.68E-60	########	0.642	0.427	2.81E-56	1
Nans	1.35E-71	########	0.556	0.329	2.27E-67	1
Casc4	0	########	0.325	0.028	0	1
Atp2a2	1.35E-36	########	0.607	0.469	2.27E-32	1
H2afv	1.30E-47	########	0.779	0.575	2.17E-43	1
Ybx1	2.04E-18	########	0.955	0.902	3.42E-14	1
Eif3c	3.25E-39	########	0.845	0.75	5.44E-35	1
Map1lc3a	3.52E-19	########	0.471	0.364	5.89E-15	1
Glo1	1.05E-97	########	0.601	0.314	1.75E-93	1
Nt5c3b	6.88E-219	########	0.395	0.087	1.15E-214	1
1110065P2	2.09E-95	########	0.438	0.199	3.50E-91	1
Tbx15	0	########	0.29	0.009	0	1
Cd6	2.00E-157	########	0.124	0.005	3.35E-153	1
Psmg1	1.16E-120	########	0.452	0.186	1.95E-116	1
Tox	2.07E-140	########	0.139	0.012	3.46E-136	1
Tomm70a	8.94E-70	########	0.546	0.32	1.50E-65	1
Tdrp	4.07E-167	########	0.131	0.005	6.81E-163	1
Thbs3	0	########	0.263	0.015	0	1
Sec22b	8.34E-72	########	0.561	0.344	1.40E-67	1
Phb	1.33E-115	########	0.537	0.239	2.22E-111	1
Pxdn	4.47E-253	########	0.32	0.044	7.48E-249	1
Uri1	2.40E-103	########	0.512	0.232	4.01E-99	1
Jakmip1	3.92E-96	########	0.188	0.042	6.57E-92	1
Ndufaf2	2.41E-107	########	0.457	0.195	4.04E-103	1
Ruvbl1	7.09E-89	########	0.513	0.263	1.19E-84	1
Cst7	1.86E-54	########	0.188	0.068	3.10E-50	1
1110004F1	1.02E-50	########	0.743	0.55	1.71E-46	1
Acta2	2.21E-161	6.460225	0.981	0.145	3.70E-157	2
Tagln	0	5.966368	0.951	0.042	0	2
Tpm2	7.14E-208	4.894654	0.981	0.104	1.19E-203	2
Tpm1	1.73E-104	4.03764	1	0.278	2.90E-100	2
Myl9	0	4.004519	0.951	0.04	0	2
Cald1	6.62E-162	3.890932	1	0.149	1.11E-157	2
Gm13889	0	3.841982	0.951	0.028	0	2
Igfbp7	1.68E-136	3.69045	0.981	0.163	2.81E-132	2
Rgs5	0	3.689598	0.767	0.019	0	2
Mustn1	1.77E-302	3.682272	0.893	0.051	2.96E-298	2
Myh11	0	3.679164	0.942	0.008	0	2
Mylk	0	3.476923	0.961	0.044	0	2

Actg2	0	3.114832	0.592	0.003	0	2
Csrp1	2.09E-94	3.072433	1	0.3	3.50E-90	2
Col4a1	3.02E-154	2.975728	0.951	0.123	5.05E-150	2
Crip1	1.37E-52	2.904723	0.981	0.718	2.29E-48	2
Sparcl1	6.74E-237	2.884943	0.835	0.055	1.13E-232	2
Ppp1r14a	0	2.850135	0.922	0.027	0	2
Sparc	1.26E-96	2.791328	0.971	0.205	2.11E-92	2
Sncg	0	2.75391	0.874	0.035	0	2
Tuba1a	1.14E-63	2.712389	0.961	0.455	1.91E-59	2
Hspb1	2.35E-132	2.709698	0.932	0.133	3.94E-128	2
Col18a1	9.75E-221	2.707265	0.942	0.084	1.63E-216	2
Mfge8	1.18E-152	2.658536	0.981	0.147	1.98E-148	2
Col6a3	7.50E-189	2.645151	0.932	0.094	1.26E-184	2
Ctgf	6.25E-68	2.545097	0.476	0.061	1.05E-63	2
Des	0	2.513556	0.854	0.02	0	2
Ndufa4l2	5.15E-213	2.359529	0.816	0.06	8.62E-209	2
Procr	1.29E-163	2.358551	0.854	0.091	2.16E-159	2
Igfbp5	4.85E-148	2.354996	0.825	0.085	8.11E-144	2
Pdgfa	3.28E-175	2.329995	0.951	0.114	5.49E-171	2
Gng11	3.51E-139	2.31948	0.981	0.145	5.88E-135	2
Cxcl1	1.88E-63	2.295304	0.883	0.233	3.15E-59	2
Prkcdbp	4.08E-131	2.294237	0.961	0.148	6.83E-127	2
Col4a2	4.41E-157	2.293947	0.922	0.11	7.38E-153	2
Ptp4a3	3.55E-88	2.290139	0.942	0.249	5.94E-84	2
Crispld2	6.84E-72	2.267323	0.825	0.194	1.14E-67	2
Col6a1	1.61E-149	2.266429	0.874	0.1	2.69E-145	2
Fermt2	1.76E-162	2.256271	0.971	0.126	2.95E-158	2
Filip1l	2.83E-62	2.156647	0.922	0.344	4.74E-58	2
Notch3	0	2.117448	0.961	0.021	0	2
Col3a1	4.77E-66	2.106416	0.864	0.207	7.98E-62	2
Fabp4	3.86E-75	2.101996	0.767	0.145	6.47E-71	2
Ckb	5.54E-79	2.100857	0.942	0.253	9.26E-75	2
Tinagl1	2.98E-132	2.092654	0.845	0.111	4.99E-128	2
Col6a2	1.39E-146	2.084207	0.825	0.091	2.33E-142	2
Lmod1	0	2.076582	0.854	0.004	0	2
Lpp	4.47E-84	2.055641	0.961	0.279	7.48E-80	2
Cnn1	0	2.054617	0.466	0.003	0	2
Meg3	1.55E-144	2.048276	0.738	0.07	2.59E-140	2
Id3	4.20E-71	2.036256	0.942	0.271	7.02E-67	2
Crip2	5.90E-142	2.017173	0.971	0.135	9.87E-138	2
Prrx1	4.47E-168	1.981549	0.883	0.091	7.48E-164	2
Bgn	3.02E-113	1.975418	0.932	0.14	5.06E-109	2
Serpinh1	3.32E-102	1.969874	0.951	0.164	5.56E-98	2
Sdc1	2.46E-85	1.925371	0.748	0.128	4.12E-81	2
Slc25a4	1.83E-63	1.915905	0.99	0.514	3.07E-59	2
Col5a3	2.42E-173	1.912551	0.806	0.073	4.05E-169	2
Pi15	1.16E-186	1.909545	0.68	0.046	1.94E-182	2
Pdgfrb	1.31E-206	1.905545	0.903	0.08	2.19E-202	2
Cryab	1.20E-220	1.894643	0.922	0.075	2.00E-216	2
Mcam	5.82E-262	1.888947	0.883	0.056	9.73E-258	2
Lgals1	5.38E-43	1.878569	0.981	0.655	9.01E-39	2
Adamts1	7.41E-128	1.859132	0.932	0.132	1.24E-123	2

Map1b	1.34E-162	1.857487	0.893	0.1	2.24E-158	2
Rasl11a	1.34E-273	1.839183	0.738	0.035	2.24E-269	2
Ptrf	8.56E-127	1.823144	0.971	0.153	1.43E-122	2
Itgb1	3.57E-52	1.820546	0.99	0.646	5.97E-48	2
Rarres2	9.48E-185	1.814674	0.913	0.086	1.59E-180	2
Il6	8.66E-89	1.799756	0.68	0.097	1.45E-84	2
Cd81	1.04E-66	1.799056	0.981	0.37	1.73E-62	2
Tpm4	1.73E-46	1.787052	0.981	0.671	2.89E-42	2
Tmem176a	2.43E-46	1.78044	0.816	0.266	4.07E-42	2
Cav1	2.15E-126	1.778531	0.961	0.15	3.60E-122	2
Rhoj	9.54E-160	1.775387	0.932	0.115	1.60E-155	2
Ehd2	4.23E-157	1.773159	0.951	0.123	7.07E-153	2
Lhfp	2.41E-189	1.772965	0.932	0.094	4.04E-185	2
Mprip	3.64E-81	1.771077	0.971	0.321	6.08E-77	2
Dstn	2.93E-27	1.755908	1	0.708	4.90E-23	2
Htra1	6.03E-202	1.750698	0.942	0.091	1.01E-197	2
Fstl1	1.82E-120	1.737111	0.951	0.135	3.04E-116	2
Csrp2	3.25E-66	1.733498	0.942	0.285	5.44E-62	2
Mgst3	2.23E-72	1.713343	0.903	0.267	3.73E-68	2
Prss23	7.83E-169	1.693408	0.835	0.081	1.31E-164	2
Myl6	3.68E-46	1.692006	1	0.977	6.15E-42	2
Rbpms	1.37E-117	1.678042	0.942	0.173	2.30E-113	2
Ptms	5.44E-59	1.67795	0.99	0.497	9.10E-55	2
Eln	5.74E-146	1.669496	0.68	0.058	9.60E-142	2
Pde3a	0	1.656594	0.893	0.017	0	2
Sepw1	4.62E-51	1.655504	0.981	0.67	7.73E-47	2
Cyb5r3	4.89E-66	1.652389	0.971	0.374	8.18E-62	2
Rhoc	2.08E-85	1.652141	0.932	0.218	3.48E-81	2
Il1r1	3.63E-145	1.639765	0.903	0.109	6.08E-141	2
Serping1	5.77E-165	1.63327	0.942	0.098	9.66E-161	2
Col1a1	3.30E-73	1.609048	0.845	0.172	5.51E-69	2
Nedd4	1.45E-63	1.597808	0.951	0.297	2.42E-59	2
Nr2f2	1.70E-227	1.583141	0.913	0.07	2.84E-223	2
Vim	8.41E-41	1.582649	0.981	0.898	1.41E-36	2
Selm	2.71E-78	1.579059	0.951	0.239	4.53E-74	2
Epas1	6.24E-230	1.575257	0.893	0.066	1.04E-225	2
Gucy1a3	0	1.57029	0.874	0.013	0	2
Nexn	0	1.563883	0.854	0.043	0	2
Adamts2	1.35E-184	1.554764	0.864	0.079	2.27E-180	2
Lrrc32	3.95E-156	1.551119	0.757	0.07	6.61E-152	2
Vcl	1.35E-60	1.548396	0.951	0.364	2.25E-56	2
Aoc3	0	1.536241	0.932	0.033	0	2
Rras	8.52E-68	1.526971	0.942	0.305	1.43E-63	2
Serpine2	2.24E-133	1.526233	0.825	0.1	3.75E-129	2
Rrad	6.41E-69	1.523481	0.757	0.15	1.07E-64	2
Flna	9.56E-32	1.471695	0.981	0.758	1.60E-27	2
Tmem176b	1.19E-42	1.463716	0.854	0.295	1.99E-38	2
Sorbs2	1.19E-270	1.458348	0.583	0.021	1.99E-266	2
Cystm1	9.45E-199	1.456524	0.854	0.074	1.58E-194	2
Tubb4b	4.07E-38	1.449325	0.951	0.577	6.81E-34	2
Calu	1.09E-57	1.444194	0.951	0.367	1.82E-53	2
Thy1	8.89E-50	1.443493	0.65	0.145	1.49E-45	2

Cd151	1.09E-110	1.432334	0.981	0.197	1.82E-106	2
Lgalsl	1.21E-162	1.417439	0.874	0.095	2.02E-158	2
Crlf1	3.08E-100	1.388635	0.534	0.052	5.15E-96	2
Hspb2	0	1.355496	0.883	0.025	0	2
Fxyd1	2.78E-194	1.347963	0.874	0.073	4.65E-190	2
Ecm1	1.28E-37	1.346492	0.883	0.374	2.15E-33	2
Timp3	1.07E-135	1.34524	0.825	0.091	1.78E-131	2
Errfi1	3.05E-61	1.342674	0.922	0.26	5.10E-57	2
Bcam	3.58E-229	1.338968	0.796	0.05	6.00E-225	2
mt-Cytb	1.38E-44	1.325556	1	0.964	2.31E-40	2
Col1a2	3.92E-92	1.324751	0.932	0.167	6.55E-88	2
Pls3	6.83E-113	1.323641	0.883	0.136	1.14E-108	2
Actn4	3.74E-43	1.315695	0.971	0.555	6.26E-39	2
Tns1	1.13E-99	1.306904	0.932	0.177	1.89E-95	2
Rexo2	3.09E-47	1.304616	0.961	0.505	5.18E-43	2
Laptm4a	7.84E-38	1.303384	0.961	0.64	1.31E-33	2
Gp1bb	1.41E-19	1.301622	0.117	0.012	2.36E-15	2
15-Sep	4.12E-50	1.294164	1	0.776	6.90E-46	2
Zak	7.09E-85	1.293176	0.883	0.196	1.19E-80	2
Ccl11	5.28E-127	1.292926	0.466	0.031	8.84E-123	2
Gnas	3.28E-45	1.283979	1	0.881	5.49E-41	2
Lmna	1.00E-38	1.274964	0.971	0.446	1.68E-34	2
Actn1	8.70E-28	1.270668	0.913	0.453	1.46E-23	2
Clic4	2.20E-43	1.270304	0.932	0.413	3.69E-39	2
Palld	2.89E-142	1.262342	0.864	0.104	4.83E-138	2
Pcp4l1	0	1.261338	0.728	0.027	0	2
Synpo2	0	1.258801	0.786	0.006	0	2
Gucy1b3	0	1.258298	0.883	0.012	0	2
Khdrbs3	2.50E-298	1.255369	0.864	0.047	4.18E-294	2
Cd9	5.04E-28	1.248654	0.971	0.545	8.43E-24	2
Lamb2	1.37E-159	1.234138	0.864	0.091	2.30E-155	2
Il6st	2.83E-53	1.220303	0.903	0.286	4.74E-49	2
Ift43	1.68E-146	1.218346	0.932	0.126	2.81E-142	2
Cxcl5	2.98E-19	1.211391	0.35	0.09	4.98E-15	2
Nenf	8.15E-55	1.207358	0.913	0.332	1.36E-50	2
Dnajb4	1.30E-62	1.206698	0.816	0.209	2.17E-58	2
Dynll1	1.98E-40	1.19946	0.981	0.843	3.32E-36	2
Klf9	9.22E-66	1.182733	0.922	0.249	1.54E-61	2
Rcn3	4.87E-82	1.182443	0.864	0.161	8.15E-78	2
Cspg4	7.59E-115	1.180373	0.466	0.035	1.27E-110	2
Ednra	0	1.178747	0.796	0.035	0	2
Gstt1	4.24E-127	1.174339	0.602	0.053	7.10E-123	2
Snhg18	3.90E-154	1.169266	0.874	0.092	6.53E-150	2
Tm4sf1	1.01E-109	1.165114	0.874	0.12	1.68E-105	2
Col5a2	3.37E-104	1.16326	0.864	0.117	5.64E-100	2
Olfml2b	1.23E-87	1.16085	0.845	0.164	2.05E-83	2
Tppp3	6.88E-57	1.159727	0.786	0.177	1.15E-52	2
Prdx4	5.60E-42	1.15535	0.903	0.352	9.36E-38	2
Ifi27	3.90E-43	1.154563	0.961	0.48	6.53E-39	2
Loxl2	4.32E-130	1.152069	0.777	0.086	7.22E-126	2
Gjc1	1.96E-209	1.143168	0.777	0.055	3.27E-205	2
Nr4a1	6.83E-32	1.139192	0.864	0.38	1.14E-27	2

Atp2b4	2.24E-80	1.136375	0.874	0.187	3.75E-76	2
Fkbp1a	2.97E-44	1.136232	0.971	0.554	4.97E-40	2
Lamc1	4.21E-91	1.133307	0.883	0.173	7.05E-87	2
Timp4	1.16E-112	1.132653	0.252	0.009	1.95E-108	2
Sh3bgrl	5.29E-35	1.131914	0.951	0.609	8.85E-31	2
Abcc9	1.35E-272	1.128228	0.602	0.022	2.26E-268	2
Rock2	1.81E-34	1.126306	0.922	0.522	3.04E-30	2
Wtip	4.17E-126	1.120211	0.796	0.097	6.97E-122	2
Cyr61	6.76E-90	1.11953	0.757	0.112	1.13E-85	2
Fbxl22	7.74E-103	1.114892	0.524	0.049	1.30E-98	2
Tgfb1i1	1.89E-201	1.114164	0.874	0.075	3.16E-197	2
Atp2a2	1.91E-35	1.113869	0.951	0.489	3.19E-31	2
Tubb5	1.22E-24	1.111912	0.961	0.687	2.04E-20	2
2810428I15	3.03E-39	1.108792	0.971	0.55	5.06E-35	2
Akap12	8.71E-118	1.106938	0.709	0.078	1.46E-113	2
Pcolce	3.41E-106	1.106681	0.835	0.109	5.70E-102	2
Hist1h2bc	9.25E-31	1.103169	0.874	0.365	1.55E-26	2
Inhba	2.75E-48	1.10158	0.573	0.118	4.60E-44	2
Nudt4	2.83E-37	1.096049	0.951	0.514	4.73E-33	2
Cnn3	1.60E-89	1.093443	0.854	0.148	2.67E-85	2
Pdlim7	7.36E-55	1.093007	0.883	0.289	1.23E-50	2
Aspn	3.30E-145	1.088739	0.728	0.065	5.53E-141	2
P2rx1	2.95E-40	1.086187	0.291	0.038	4.93E-36	2
Gja4	0	1.084648	0.65	0.011	0	2
Nbl1	1.39E-142	1.083075	0.864	0.098	2.32E-138	2
Gem	1.61E-79	1.076892	0.777	0.137	2.70E-75	2
Adamts9	2.35E-183	1.076517	0.777	0.061	3.94E-179	2
Enah	8.04E-130	1.075767	0.835	0.105	1.34E-125	2
Map1lc3a	2.07E-44	1.075335	0.913	0.377	3.46E-40	2
Cd63	1.66E-27	1.0726	0.971	0.585	2.79E-23	2
Fads3	6.61E-146	1.070478	0.816	0.09	1.11E-141	2
Rgs4	0	1.070289	0.602	0.012	0	2
mt-Nd1	6.65E-31	1.07009	1	0.906	1.11E-26	2
Hes1	1.65E-51	1.067733	0.883	0.238	2.76E-47	2
Il11	2.13E-41	1.067574	0.301	0.039	3.56E-37	2
Apold1	1.23E-189	1.065583	0.689	0.045	2.06E-185	2
7-Sep	7.47E-38	1.065209	0.981	0.757	1.25E-33	2
Gdf15	3.23E-112	1.062161	0.612	0.06	5.41E-108	2
Esyt2	1.34E-45	1.060883	0.883	0.346	2.24E-41	2
Nes	2.43E-273	1.054415	0.718	0.032	4.06E-269	2
Parm1	0	1.052827	0.738	0.016	0	2
Itga1	3.69E-149	1.0505	0.845	0.092	6.18E-145	2
Fhl1	1.19E-187	1.049053	0.67	0.044	2.00E-183	2
Aebp1	1.29E-84	1.048698	0.767	0.112	2.17E-80	2
Mgp	3.18E-13	1.048635	0.34	0.113	5.32E-09	2
Gamt	3.72E-68	1.046589	0.796	0.183	6.22E-64	2
Ccnd2	2.61E-52	1.045848	0.893	0.253	4.37E-48	2
Id4	2.14E-257	1.04388	0.583	0.022	3.58E-253	2
Adamts4	9.53E-146	1.041679	0.67	0.057	1.60E-141	2
Pde1a	3.20E-241	1.037313	0.767	0.045	5.35E-237	2
Esam	0	1.032421	0.903	0.038	0	2
Serpinb6a	1.47E-41	1.031544	0.99	0.401	2.46E-37	2

Rsu1	3.12E-35	1.02972	0.961	0.595	5.22E-31	2
Nid1	1.14E-93	1.027538	0.767	0.111	1.91E-89	2
Tsc22d1	8.77E-44	1.023063	0.796	0.217	1.47E-39	2
Maged2	1.05E-99	1.021555	0.816	0.123	1.75E-95	2
Plpp3	7.10E-92	1.019687	0.835	0.128	1.19E-87	2
1500009L1	0	1.015375	0.738	0.022	0	2
Jag1	1.81E-149	1.013442	0.816	0.085	3.02E-145	2
Hacd1	4.97E-86	1.012608	0.816	0.16	8.32E-82	2
S100a6	2.46E-21	1.008123	0.961	0.845	4.11E-17	2
Eva1b	2.18E-62	1.007193	0.922	0.257	3.65E-58	2
Cfl2	1.39E-61	1.004643	0.854	0.244	2.33E-57	2
Hspa1a	1.79E-59	1.004451	0.757	0.165	2.99E-55	2
Oat	9.96E-49	1.002616	0.951	0.391	1.67E-44	2
Ddr2	1.29E-150	########	0.825	0.083	2.15E-146	2
Tgfb3	3.07E-122	########	0.563	0.048	5.13E-118	2
Ngf	1.27E-203	########	0.583	0.03	2.13E-199	2
Fbn1	9.93E-107	########	0.835	0.108	1.66E-102	2
Rbpms2	0	########	0.612	0.015	0	2
Rasd1	9.90E-162	########	0.621	0.043	1.66E-157	2
Phlda1	1.19E-25	########	0.786	0.343	1.99E-21	2
Postn	1.00E-67	########	0.612	0.094	1.67E-63	2
Sdc2	4.06E-139	########	0.806	0.088	6.80E-135	2
Cd248	3.66E-104	########	0.641	0.07	6.13E-100	2
Ednrb	1.88E-16	########	0.388	0.123	3.15E-12	2
C1s1	3.71E-152	########	0.709	0.059	6.20E-148	2
Cacna2d1	7.77E-221	########	0.777	0.052	1.30E-216	2
Atp5b	1.09E-35	########	0.99	0.842	1.83E-31	2
mt-Nd2	6.68E-22	########	1	0.891	1.12E-17	2
Crim1	1.92E-90	########	0.709	0.103	3.21E-86	2
Pdlim3	0	########	0.466	0.007	0	2
Ntn4	5.89E-181	########	0.534	0.028	9.85E-177	2
Cox4i2	0	########	0.68	0.015	0	2
Rps2	1.75E-28	########	1	0.93	2.92E-24	2
C1qtnf6	2.78E-92	########	0.709	0.099	4.66E-88	2
Arid5b	3.19E-43	########	0.893	0.352	5.33E-39	2
Nrarp	5.27E-157	########	0.65	0.049	8.82E-153	2
Olfr558	0	########	0.495	0.002	0	2
Myo1b	1.12E-99	########	0.767	0.115	1.87E-95	2
Ndufs5	1.85E-39	########	0.99	0.731	3.09E-35	2
Axl	2.69E-105	########	0.835	0.127	4.50E-101	2
Nnmt	1.53E-69	########	0.621	0.096	2.57E-65	2
Gadd45b	1.85E-15	########	0.893	0.586	3.10E-11	2
Lama5	1.03E-271	########	0.66	0.027	1.72E-267	2
Myo1c	2.37E-40	########	0.864	0.328	3.97E-36	2
Hspg2	2.56E-101	########	0.854	0.124	4.28E-97	2
Higd1b	0	########	0.447	0.006	0	2
Pdlim2	1.54E-45	########	0.835	0.287	2.57E-41	2
Ryk	2.37E-105	########	0.864	0.129	3.97E-101	2
Ilk	1.13E-32	########	0.883	0.49	1.89E-28	2
Prkar1a	7.55E-36	########	0.981	0.77	1.26E-31	2
Rasal2	1.46E-102	########	0.67	0.083	2.44E-98	2
Ppp1r14b	1.33E-19	########	0.903	0.556	2.23E-15	2

Smtn	1.44E-84	########	0.68	0.105	2.41E-80	2
Npy1r	0	########	0.553	0.006	0	2
Msrb3	8.98E-151	########	0.718	0.066	1.50E-146	2
Pde5a	8.13E-241	########	0.709	0.037	1.36E-236	2
Nupr1	3.38E-25	########	0.806	0.348	5.65E-21	2
Pln	0	########	0.214	0	0	2
Efemp2	2.25E-99	########	0.854	0.126	3.76E-95	2
Dst	3.46E-74	########	0.864	0.168	5.80E-70	2
Ddah2	8.38E-82	########	0.816	0.147	1.40E-77	2
Col5a1	2.56E-78	########	0.709	0.104	4.28E-74	2
Hmgn1	1.31E-34	########	0.951	0.409	2.19E-30	2
Serpine1	2.02E-45	########	0.563	0.112	3.38E-41	2
4-Sep	9.65E-106	########	0.447	0.034	1.62E-101	2
Mlf1	8.21E-231	########	0.718	0.041	1.37E-226	2
Zbtb20	1.24E-37	########	0.893	0.326	2.07E-33	2
Gpx8	2.62E-124	########	0.835	0.097	4.39E-120	2
mt-Atp6	4.94E-26	########	1	0.989	8.27E-22	2
Gnb4	1.06E-125	########	0.757	0.086	1.78E-121	2
Bcr	3.44E-69	########	0.699	0.134	5.75E-65	2
Gstm1	6.80E-21	########	0.718	0.298	1.14E-16	2
Myl12a	9.18E-26	########	0.942	0.842	1.54E-21	2
Cox6c	3.65E-33	########	1	0.913	6.11E-29	2
Arf4	6.18E-31	########	0.971	0.634	1.03E-26	2
Jun	3.88E-23	########	0.942	0.549	6.50E-19	2
Pdlim1	1.72E-53	########	0.874	0.255	2.87E-49	2
Nfib	2.91E-88	########	0.903	0.142	4.87E-84	2
Ppp1r12b	1.98E-81	########	0.602	0.083	3.32E-77	2
2200002D(	5.57E-96	########	0.553	0.056	9.32E-92	2
Ppp1r12a	5.13E-16	########	0.903	0.599	8.59E-12	2
Chchd2	2.41E-32	########	1	0.961	4.04E-28	2
Ppic	1.70E-75	########	0.816	0.142	2.84E-71	2
Stk38l	3.96E-123	########	0.66	0.067	6.62E-119	2
Wwtr1	1.38E-101	########	0.845	0.124	2.30E-97	2
Kcnk3	0	########	0.66	0.002	0	2
11-Sep	3.89E-31	########	0.893	0.415	6.50E-27	2
Ramp1	6.90E-18	########	0.718	0.35	1.15E-13	2
Dag1	3.99E-62	########	0.854	0.216	6.67E-58	2
Kitl	2.24E-101	########	0.67	0.079	3.76E-97	2
Pgrmc1	7.26E-47	########	0.903	0.322	1.21E-42	2
Ebf1	1.81E-91	########	0.942	0.162	3.02E-87	2
Parva	4.34E-98	########	0.777	0.115	7.26E-94	2
Cyb5a	1.07E-27	########	0.961	0.566	1.79E-23	2
Dlc1	2.23E-131	########	0.816	0.095	3.74E-127	2
Ndufb6	5.25E-30	########	0.922	0.595	8.79E-26	2
Ube2s	1.23E-19	########	0.961	0.691	2.06E-15	2
Npdc1	1.13E-102	########	0.854	0.122	1.89E-98	2
Ybx3	4.13E-36	########	0.951	0.421	6.90E-32	2
Rdh10	1.58E-35	########	0.485	0.112	2.64E-31	2
Ech1	1.00E-24	########	0.932	0.569	1.68E-20	2
Tnfrsf12a	4.66E-25	########	0.777	0.303	7.79E-21	2
Rgs16	4.51E-141	########	0.505	0.032	7.55E-137	2
Kcnj8	5.32E-157	########	0.447	0.022	8.90E-153	2

Slc7a2	1.74E-118	########	0.573	0.051	2.92E-114	2
Osmr	3.33E-120	########	0.796	0.099	5.57E-116	2
Metap2	2.36E-18	########	0.942	0.635	3.95E-14	2
Cygb	3.60E-56	########	0.485	0.071	6.03E-52	2
Itga5	3.96E-16	########	0.66	0.3	6.62E-12	2
Psmb5	2.02E-23	########	0.942	0.734	3.38E-19	2
Ndufb9	1.34E-31	########	0.99	0.827	2.24E-27	2
Vtn	1.75E-151	########	0.388	0.017	2.92E-147	2
Sulf1	2.34E-109	########	0.689	0.077	3.92E-105	2
Gadd45g	2.69E-19	########	0.631	0.249	4.50E-15	2
Nckap1	2.02E-110	########	0.854	0.122	3.37E-106	2
Ndufa12	1.62E-27	########	0.942	0.565	2.70E-23	2
Dmd	4.32E-302	########	0.709	0.028	7.23E-298	2
Col8a1	1.51E-119	########	0.515	0.039	2.52E-115	2
Morf4l2	2.82E-31	########	0.951	0.514	4.72E-27	2
Dad1	2.28E-27	########	0.981	0.736	3.81E-23	2
Arhgef12	4.76E-69	########	0.825	0.176	7.96E-65	2
Wbp5	6.33E-31	########	0.903	0.421	1.06E-26	2
Map3k7cl	0	########	0.524	0.001	0	2
Ktn1	1.18E-35	########	0.883	0.356	1.98E-31	2
Rab13	3.13E-62	########	0.748	0.17	5.24E-58	2
Gpx4	3.72E-16	########	0.981	0.86	6.22E-12	2
Pmepa1	5.96E-55	########	0.835	0.189	9.96E-51	2
Fkbp9	7.61E-84	########	0.816	0.136	1.27E-79	2
2310022B0	1.62E-89	########	0.738	0.117	2.71E-85	2
Ccdc107	4.99E-40	########	0.786	0.256	8.35E-36	2
Arhgef7	2.02E-43	########	0.757	0.221	3.38E-39	2
Bok	3.38E-88	########	0.66	0.088	5.65E-84	2
Fam129a	4.04E-17	########	0.845	0.46	6.76E-13	2
Ppp1r15a	3.94E-17	########	0.903	0.496	6.59E-13	2
Hdlbp	1.13E-26	########	0.932	0.596	1.89E-22	2
Cpe	5.18E-167	########	0.68	0.049	8.67E-163	2
Atpif1	4.51E-20	########	0.981	0.738	7.55E-16	2
Ndufa11	2.08E-26	########	0.951	0.681	3.48E-22	2
Wisp2	6.24E-34	########	0.379	0.069	1.04E-29	2
Hmgn3	5.56E-82	########	0.816	0.147	9.31E-78	2
Phlda3	3.03E-91	########	0.767	0.114	5.07E-87	2
Uqcc2	7.81E-25	########	0.942	0.534	1.31E-20	2
Anxa5	4.24E-22	########	0.981	0.679	7.09E-18	2
Calm2	4.08E-18	########	0.961	0.896	6.83E-14	2
Fosb	7.12E-20	########	0.922	0.462	1.19E-15	2
Synm	2.01E-101	########	0.485	0.042	3.36E-97	2
Tnc	7.60E-83	########	0.621	0.08	1.27E-78	2
Ndrg2	6.64E-164	########	0.67	0.05	1.11E-159	2
Epn2	2.90E-133	########	0.757	0.082	4.86E-129	2
Manf	8.22E-17	########	0.864	0.6	1.38E-12	2
Itga2b	3.24E-10	########	0.165	0.04	5.43E-06	2
Vasn	6.99E-96	########	0.738	0.1	1.17E-91	2
Snrpd2	4.72E-24	########	0.971	0.691	7.89E-20	2
Phldb2	2.61E-79	########	0.563	0.072	4.36E-75	2
Myof	2.78E-36	########	0.816	0.294	4.65E-32	2
Zfhx3	5.05E-29	########	0.806	0.322	8.45E-25	2

Slmap	8.67E-25	########	0.738	0.319	1.45E-20	2
Myom1	2.21E-287	########	0.485	0.012	3.69E-283	2
Copz2	1.78E-95	########	0.835	0.126	2.98E-91	2
Slit3	1.48E-111	########	0.66	0.069	2.47E-107	2
Kctd10	8.25E-41	########	0.845	0.314	1.38E-36	2
S1pr3	7.97E-118	########	0.563	0.049	1.33E-113	2
Gna11	1.10E-65	########	0.845	0.196	1.83E-61	2
Plpp1	6.92E-102	########	0.796	0.105	1.16E-97	2
Ildr2	9.48E-189	########	0.563	0.03	1.59E-184	2
Rheb	4.43E-30	########	0.951	0.618	7.41E-26	2
Ndufs2	1.33E-27	########	0.951	0.614	2.23E-23	2
Atf5	1.45E-57	########	0.718	0.156	2.43E-53	2
Arhgef17	3.85E-303	########	0.757	0.033	6.45E-299	2
Syne2	2.99E-37	########	0.757	0.239	5.00E-33	2
Gstm2	4.99E-93	########	0.68	0.089	8.36E-89	2
mt-Nd4	3.23E-19	########	1	0.961	5.41E-15	2
Atf3	3.03E-24	########	0.835	0.355	5.08E-20	2
Rasgrp2	4.77E-22	########	0.913	0.468	7.98E-18	2
Ttll7	4.43E-196	########	0.602	0.033	7.42E-192	2
Isyna1	2.90E-34	########	0.728	0.242	4.85E-30	2
Btbd3	2.65E-137	########	0.728	0.072	4.43E-133	2
Tceal8	1.50E-40	########	0.825	0.267	2.50E-36	2
Ddit4	4.85E-34	########	0.709	0.214	8.12E-30	2
Atp1b2	0	########	0.68	0.008	0	2
Rock1	3.29E-18	########	0.951	0.775	5.50E-14	2
Cobll1	2.59E-176	########	0.709	0.052	4.33E-172	2
Mast4	5.35E-83	########	0.786	0.128	8.96E-79	2
Hbegf	1.09E-50	########	0.699	0.152	1.82E-46	2
Ndufb2	1.29E-27	########	0.961	0.601	2.16E-23	2
Rabac1	2.60E-28	########	0.981	0.826	4.35E-24	2
Nrip2	0	########	0.398	0.001	0	2
Pdlim5	4.34E-23	########	0.835	0.402	7.25E-19	2
Ctnna1	6.60E-35	########	0.932	0.422	1.10E-30	2
Rbms3	1.29E-130	########	0.728	0.074	2.15E-126	2
Uqcr11	4.95E-24	########	0.971	0.786	8.28E-20	2
mt-Co3	6.81E-17	########	1	0.991	1.14E-12	2
Emp2	1.83E-126	########	0.796	0.092	3.05E-122	2
Ppp1cb	1.77E-21	########	0.961	0.607	2.96E-17	2
Col12a1	4.33E-97	########	0.689	0.083	7.24E-93	2
Dynlrb1	3.65E-29	########	0.981	0.677	6.10E-25	2
Luzp1	9.83E-51	########	0.845	0.234	1.65E-46	2
Cbx6	7.57E-82	########	0.621	0.085	1.27E-77	2
Timm17a	1.36E-31	########	0.864	0.404	2.28E-27	2
Prkacb	5.93E-29	########	0.854	0.388	9.93E-25	2
Ppib	1.81E-20	########	0.981	0.84	3.02E-16	2
Ifitm3	1.29E-13	########	1	0.794	2.16E-09	2
Tgfb2	8.38E-94	########	0.563	0.061	1.40E-89	2
mt-Nd3	7.68E-16	########	0.981	0.845	1.28E-11	2
Oxct1	3.41E-28	########	0.883	0.394	5.71E-24	2
Cox6a1	2.48E-23	########	0.99	0.833	4.16E-19	2
Maged1	3.70E-71	########	0.816	0.147	6.20E-67	2
Pmp22	7.31E-82	########	0.883	0.171	1.22E-77	2

Tmem47	2.97E-151	########	0.65	0.051	4.97E-147	2
2010111I0'	1.91E-31	########	0.748	0.261	3.20E-27	2
Edf1	9.95E-21	########	0.961	0.805	1.67E-16	2
Eif1ax	9.19E-25	########	0.893	0.541	1.54E-20	2
Tead1	3.02E-86	########	0.689	0.1	5.05E-82	2
Sod3	2.11E-82	########	0.68	0.089	3.54E-78	2
Ikbip	7.40E-61	########	0.806	0.183	1.24E-56	2
Prkar2b	1.40E-25	########	0.592	0.203	2.34E-21	2
Tagln2	1.15E-13	########	0.981	0.756	1.93E-09	2
Ppia	5.08E-22	########	1	0.903	8.51E-18	2
Jph2	0	########	0.592	0.009	0	2
Mrvi1	0	########	0.689	0.008	0	2
Pkig	3.75E-25	########	0.845	0.433	6.27E-21	2
Arhgap6	7.58E-151	########	0.689	0.058	1.27E-146	2
Tspan3	3.58E-38	########	0.874	0.302	5.99E-34	2
Id1	3.47E-21	########	0.641	0.257	5.81E-17	2
Fblim1	7.44E-77	########	0.718	0.12	1.24E-72	2
Sptbn1	8.14E-21	########	0.932	0.547	1.36E-16	2
8-Sep	2.94E-62	########	0.748	0.158	4.92E-58	2
Psmd7	2.07E-24	########	0.883	0.501	3.46E-20	2
Anxa3	4.32E-15	########	0.835	0.457	7.23E-11	2
Plec	3.88E-17	########	0.922	0.617	6.49E-13	2
Tuba1b	2.16E-14	########	0.922	0.598	3.62E-10	2
Nts	1.12E-34	########	0.126	0.008	1.88E-30	2
Uqcrb	2.90E-20	########	0.971	0.761	4.85E-16	2
Tubb6	9.68E-20	########	0.796	0.381	1.62E-15	2
Ndufv1	2.58E-27	########	0.883	0.452	4.31E-23	2
Kdelr2	1.53E-23	########	0.883	0.463	2.57E-19	2
Mrfap1	7.26E-23	########	0.961	0.7	1.21E-18	2
Dbi	3.21E-17	########	0.981	0.724	5.38E-13	2
Ndufab1	3.36E-23	########	0.971	0.591	5.62E-19	2
Hmgb1	1.56E-16	########	1	0.879	2.62E-12	2
Itga7	0	########	0.689	0.024	0	2
Cltb	3.22E-36	########	0.845	0.307	5.39E-32	2
Minos1	7.66E-23	########	0.971	0.769	1.28E-18	2
Ndufa5	3.74E-20	########	0.913	0.562	6.26E-16	2
Hspa5	2.24E-16	########	0.971	0.835	3.74E-12	2
Sema6d	2.75E-128	########	0.583	0.049	4.61E-124	2
Cope	2.11E-21	########	0.913	0.634	3.53E-17	2
Tspan17	3.16E-74	########	0.748	0.14	5.29E-70	2
Map4	6.45E-29	########	0.854	0.382	1.08E-24	2
Filip1	9.92E-128	########	0.466	0.03	1.66E-123	2
Tspan2	3.64E-82	########	0.631	0.088	6.09E-78	2
Dab2ip	3.51E-70	########	0.786	0.151	5.88E-66	2
Adra2a	0	########	0.466	0.009	0	2
Mrpl20	3.66E-19	########	0.913	0.692	6.13E-15	2
Anxa6	8.04E-28	########	0.854	0.407	1.34E-23	2
Fat1	3.68E-132	########	0.699	0.069	6.15E-128	2
Tbrg1	4.94E-25	########	0.883	0.464	8.27E-21	2
Oaz2	1.07E-31	########	0.816	0.308	1.78E-27	2
Lama4	4.56E-93	########	0.718	0.095	7.63E-89	2
Bsg	3.59E-21	########	0.961	0.704	6.01E-17	2

Kdelr3	2.55E-86	########	0.68	0.09	4.27E-82	2
Atp5d	1.22E-21	########	0.981	0.815	2.04E-17	2
Swi5	1.10E-21	########	0.942	0.691	1.85E-17	2
Egr1	9.59E-24	########	0.874	0.362	1.60E-19	2
Hspa1b	1.63E-49	########	0.641	0.134	2.73E-45	2
Gm10073	4.93E-22	########	0.942	0.559	8.25E-18	2
Rps27l	3.60E-17	########	0.932	0.742	6.02E-13	2
Ntrk2	5.39E-131	########	0.534	0.039	9.02E-127	2
Cdc42bpa	4.23E-89	########	0.699	0.102	7.08E-85	2
Fos	2.89E-13	########	0.932	0.591	4.84E-09	2
Arhgap42	5.19E-124	########	0.612	0.057	8.68E-120	2
Atp5o	7.19E-18	########	0.981	0.753	1.20E-13	2
Nr4a2	3.31E-15	########	0.689	0.304	5.55E-11	2
mt-Co2	1.59E-17	########	1	0.989	2.66E-13	2
Inpp5a	1.01E-61	########	0.718	0.146	1.69E-57	2
Ptprf	2.11E-133	########	0.67	0.06	3.54E-129	2
Ybx1	1.12E-16	########	1	0.91	1.87E-12	2
Nab1	1.15E-21	########	0.854	0.473	1.93E-17	2
Psmb6	4.03E-19	########	0.942	0.711	6.74E-15	2
Ptov1	1.21E-36	########	0.816	0.292	2.03E-32	2
Fundc2	2.09E-25	########	0.932	0.491	3.50E-21	2
Arhgef25	1.75E-145	########	0.68	0.058	2.92E-141	2
Pdcl3	2.85E-26	########	0.883	0.466	4.78E-22	2
Myadm	4.28E-23	########	0.854	0.437	7.16E-19	2
Sh3bgr	2.05E-299	########	0.379	0.006	3.42E-295	2
Impdh2	1.33E-24	########	0.913	0.41	2.23E-20	2
Rpl22l1	7.08E-16	########	1	0.804	1.18E-11	2
Tsen34	1.78E-30	########	0.825	0.343	2.97E-26	2
Utrn	6.13E-26	########	0.796	0.319	1.03E-21	2
Ccl19	4.50E-112	########	0.301	0.014	7.52E-108	2
2900026AC	8.06E-100	########	0.573	0.06	1.35E-95	2
Ywhaq	2.16E-18	########	0.932	0.632	3.62E-14	2
Ift20	6.09E-21	########	0.903	0.536	1.02E-16	2
Kcnab1	4.06E-202	########	0.553	0.026	6.79E-198	2
Rem1	1.84E-265	########	0.68	0.03	3.08E-261	2
Igfbp4	2.33E-25	########	0.854	0.354	3.90E-21	2
Stub1	1.38E-21	########	0.864	0.478	2.31E-17	2
Ndufv2	1.79E-17	########	0.903	0.631	3.00E-13	2
Ppp1r2	1.13E-22	########	0.951	0.636	1.88E-18	2
Fkbp10	2.94E-77	########	0.718	0.106	4.93E-73	2
Mxra7	9.63E-82	########	0.699	0.102	1.61E-77	2
Map1a	2.67E-167	########	0.68	0.051	4.46E-163	2
Ttc28	6.45E-65	########	0.631	0.107	1.08E-60	2
Mif	3.11E-15	########	0.981	0.673	5.20E-11	2
Cd200	6.92E-73	########	0.612	0.084	1.16E-68	2
Medag	9.68E-73	########	0.631	0.089	1.62E-68	2
Ubb	6.10E-19	########	1	0.984	1.02E-14	2
Comt	1.03E-24	########	0.883	0.419	1.73E-20	2
Dnajb1	8.42E-18	########	0.757	0.404	1.41E-13	2
Arhgap29	5.19E-81	########	0.68	0.097	8.69E-77	2
Romo1	3.05E-21	########	0.961	0.685	5.10E-17	2
Tubb2a	1.03E-22	########	0.816	0.337	1.72E-18	2

Pebp1	3.01E-19	########	0.942	0.617	5.04E-15	2
Mrps6	3.79E-40	########	0.689	0.193	6.35E-36	2
Samd4	3.25E-97	########	0.621	0.072	5.43E-93	2
Dmpk	5.93E-124	########	0.427	0.026	9.92E-120	2
Agpat3	5.56E-46	########	0.835	0.233	9.30E-42	2
Ssr4	1.28E-13	########	0.942	0.771	2.14E-09	2
Nap1l1	1.62E-13	########	0.922	0.668	2.71E-09	2
Lxn	1.87E-22	########	0.65	0.255	3.14E-18	2
Nfe2l1	5.28E-26	########	0.883	0.418	8.83E-22	2
Trim47	2.94E-62	########	0.699	0.137	4.91E-58	2
Dctn2	9.90E-20	########	0.903	0.571	1.66E-15	2
Loxl1	1.04E-66	########	0.612	0.087	1.73E-62	2
Ccl2	4.73E-14	########	0.689	0.305	7.92E-10	2
Atp5g1	2.24E-15	########	0.942	0.731	3.75E-11	2
Nfix	2.97E-32	########	0.835	0.296	4.96E-28	2
Psmd8	5.41E-10	########	0.893	0.733	9.05E-06	2
Tceb2	2.31E-21	########	0.99	0.909	3.86E-17	2
Rap2a	6.89E-13	########	0.65	0.351	1.15E-08	2
Atp1a2	8.10E-62	########	0.204	0.012	1.36E-57	2
Hsp90b1	3.69E-15	########	0.961	0.766	6.18E-11	2
Gtf2h5	3.65E-20	########	0.932	0.653	6.10E-16	2
2-Sep	9.55E-19	########	0.913	0.553	1.60E-14	2
Rgs7bp	3.56E-171	########	0.505	0.026	5.96E-167	2
Lbh	3.77E-13	########	0.699	0.349	6.31E-09	2
Tnks1bp1	9.47E-77	########	0.728	0.124	1.58E-72	2
Pam	8.25E-32	########	0.767	0.281	1.38E-27	2
Ndufa13	3.70E-18	########	1	0.881	6.19E-14	2
Drap1	2.87E-19	########	0.922	0.699	4.79E-15	2
Kank2	3.68E-88	########	0.66	0.089	6.16E-84	2
Fam92a	2.17E-61	########	0.786	0.173	3.63E-57	2
Ranbp1	1.61E-13	########	0.874	0.586	2.70E-09	2
Arl3	1.05E-35	########	0.796	0.276	1.75E-31	2
Mical2	1.53E-79	########	0.495	0.056	2.55E-75	2
Dnlz	1.70E-32	########	0.777	0.287	2.85E-28	2
Calr	1.71E-14	########	0.971	0.773	2.86E-10	2
Bmp1	4.96E-55	########	0.553	0.093	8.29E-51	2
Lmcd1	1.52E-98	########	0.495	0.044	2.54E-94	2
Cox8a	6.82E-23	########	1	0.954	1.14E-18	2
Mlf2	1.14E-21	########	0.874	0.472	1.92E-17	2
Galk1	7.30E-35	########	0.757	0.239	1.22E-30	2
Etfa	1.28E-20	########	0.883	0.481	2.14E-16	2
Fhl2	2.66E-111	########	0.641	0.066	4.45E-107	2
Cyp7b1	7.44E-132	########	0.524	0.037	1.25E-127	2
Cav2	1.16E-66	########	0.786	0.154	1.94E-62	2
Rcan2	1.65E-98	########	0.359	0.023	2.76E-94	2
Myocd	0	########	0.427	0.001	0	2
Nfia	6.00E-47	########	0.718	0.17	1.00E-42	2
Elovl5	3.73E-35	########	0.786	0.262	6.25E-31	2
Eif2s2	1.04E-14	########	0.961	0.766	1.73E-10	2
Cdc42ep5	2.58E-72	########	0.65	0.101	4.31E-68	2
Hras	4.34E-28	########	0.874	0.424	7.27E-24	2
Cox7b	4.84E-15	########	0.981	0.812	8.10E-11	2

Susd2	6.11E-274	########	0.544	0.017	1.02E-269	2
C1qtnf1	2.16E-138	########	0.612	0.05	3.61E-134	2
Eef1d	1.47E-14	########	0.981	0.732	2.45E-10	2
Rbfox2	6.59E-91	########	0.728	0.104	1.10E-86	2
Ndn	2.10E-101	########	0.67	0.077	3.51E-97	2
Actc1	1.49E-64	########	0.107	0.003	2.49E-60	2
mt-Nd4l	9.04E-13	########	0.961	0.749	1.51E-08	2
Tcp1	1.04E-15	########	0.864	0.575	1.74E-11	2
Fam96b	4.24E-22	########	0.835	0.42	7.09E-18	2
1810011O1	4.26E-80	########	0.621	0.083	7.13E-76	2
Flt1	6.06E-90	########	0.495	0.046	1.01E-85	2
Rwdd1	6.25E-16	########	0.903	0.606	1.05E-11	2
Crtap	2.50E-44	########	0.738	0.191	4.19E-40	2
Tnxb	1.97E-99	########	0.544	0.052	3.30E-95	2
Slc11a1	2.66E-16	########	0.728	0.337	4.46E-12	2
Apbb2	4.32E-91	########	0.718	0.098	7.23E-87	2
Angpt2	2.64E-154	########	0.612	0.043	4.42E-150	2
Pcdh1	7.79E-208	########	0.612	0.031	1.30E-203	2
Heyl	0	########	0.573	0.008	0	2
Mmp23	4.37E-52	########	0.476	0.072	7.31E-48	2
Pmm1	1.87E-46	########	0.767	0.206	3.12E-42	2
Fam162a	7.54E-26	########	0.854	0.399	1.26E-21	2
Bag3	4.60E-35	########	0.767	0.241	7.70E-31	2
Fdps	1.64E-14	########	0.66	0.3	2.75E-10	2
Sh3pxd2a	1.43E-41	########	0.738	0.187	2.39E-37	2
H2-M9	4.76E-140	########	0.107	0.001	7.96E-136	2
Hsp90ab1	1.64E-14	########	0.99	0.916	2.74E-10	2
Eif5a	6.32E-14	########	0.961	0.862	1.06E-09	2
Gsn	2.62E-17	########	1	0.705	4.39E-13	2
Pawr	4.62E-230	########	0.563	0.023	7.74E-226	2
Slc25a3	1.58E-16	########	0.971	0.867	2.64E-12	2
Mark1	6.76E-162	########	0.515	0.029	1.13E-157	2
Afap1	2.34E-64	########	0.621	0.107	3.91E-60	2
Sec13	8.08E-24	########	0.874	0.451	1.35E-19	2
Cox7a2	8.33E-15	########	0.981	0.869	1.39E-10	2
Fkbp7	1.39E-59	########	0.67	0.122	2.33E-55	2
Camk1	3.56E-26	########	0.816	0.327	5.95E-22	2
Idi1	1.19E-20	########	0.573	0.217	1.99E-16	2
Ran	1.19E-10	########	0.922	0.715	2.00E-06	2
Myo10	1.29E-58	########	0.631	0.115	2.15E-54	2
Pdcd5	9.31E-19	########	0.942	0.653	1.56E-14	2
Ndufc1	8.21E-16	########	0.961	0.669	1.37E-11	2
Psmc4	2.14E-20	########	0.922	0.54	3.58E-16	2
Ginm1	7.50E-31	########	0.757	0.277	1.25E-26	2
Thra	5.90E-39	########	0.689	0.192	9.87E-35	2
Rbbp7	1.52E-23	########	0.874	0.407	2.55E-19	2
Tspan4	1.34E-30	########	0.816	0.266	2.24E-26	2
Meox2	1.28E-75	########	0.563	0.072	2.14E-71	2
Eef1g	8.85E-13	########	0.981	0.803	1.48E-08	2
Eny2	8.29E-20	########	0.883	0.488	1.39E-15	2
Pgm5	5.31E-221	########	0.437	0.014	8.88E-217	2
Scn1b	1.49E-63	########	0.689	0.125	2.49E-59	2

Ube2m	7.90E-17	########	0.922	0.638	1.32E-12	2
Galnt16	5.99E-143	########	0.534	0.035	1.00E-138	2
Jund	1.01E-11	########	0.971	0.893	1.69E-07	2
Ebf2	6.27E-152	########	0.592	0.041	1.05E-147	2
Adcy6	2.43E-206	########	0.641	0.035	4.07E-202	2
Uqcr10	8.12E-15	########	0.961	0.792	1.36E-10	2
Lix1l	6.78E-80	########	0.689	0.105	1.14E-75	2
Tesc	2.42E-19	########	0.262	0.055	4.05E-15	2
Mxra8	2.78E-86	########	0.738	0.101	4.65E-82	2
Hcfc1r1	1.42E-08	########	0.816	0.578	########	2
Ccser2	1.29E-49	########	0.806	0.209	2.16E-45	2
Gpx7	2.57E-65	########	0.65	0.102	4.29E-61	2
Uqcrq	2.74E-15	########	0.961	0.835	4.59E-11	2
Ift27	5.15E-31	########	0.728	0.255	8.62E-27	2
Eprs	2.76E-16	########	0.845	0.453	4.62E-12	2
Krtcap2	1.81E-12	########	0.893	0.668	3.04E-08	2
Bmpr1a	8.84E-71	########	0.67	0.112	1.48E-66	2
Hmg20b	9.98E-24	########	0.874	0.424	1.67E-19	2
Erh	3.52E-13	########	0.961	0.714	5.88E-09	2
Trabd2b	5.58E-206	########	0.573	0.028	9.33E-202	2
Hspb7	3.37E-191	########	0.379	0.012	5.64E-187	2
Itgb5	1.88E-32	########	0.854	0.286	3.15E-28	2
Gstm5	7.88E-58	########	0.699	0.138	1.32E-53	2
Wdr83os	1.44E-17	########	0.903	0.596	2.41E-13	2
Pold4	3.14E-13	########	0.854	0.587	5.25E-09	2
Pigp	1.72E-34	########	0.777	0.26	2.89E-30	2
Cct7	1.30E-14	########	0.883	0.62	2.18E-10	2
4930523C0	4.94E-10	########	0.748	0.467	8.27E-06	2
Spop	9.74E-13	########	0.903	0.648	1.63E-08	2
Marveld1	2.59E-41	########	0.65	0.171	4.33E-37	2
Cttn	2.89E-70	########	0.709	0.119	4.84E-66	2
Cp	2.30E-74	########	0.456	0.049	3.85E-70	2
Hint1	6.04E-12	########	0.961	0.788	1.01E-07	2
Yif1b	1.27E-18	########	0.767	0.372	2.13E-14	2
Ndufb4	1.48E-15	########	0.932	0.712	2.48E-11	2
Tmbim1	5.41E-27	########	0.767	0.286	9.05E-23	2
Kif5b	2.13E-11	########	0.922	0.714	3.56E-07	2
Foxs1	3.67E-282	########	0.417	0.009	6.14E-278	2
Nbeal1	2.66E-23	########	0.689	0.273	4.45E-19	2
Atp5j2	5.19E-17	########	1	0.867	8.68E-13	2
Adcy5	3.07E-290	########	0.437	0.009	5.13E-286	2
Twist1	5.79E-86	########	0.621	0.075	9.69E-82	2
Peli2	1.03E-41	########	0.641	0.159	1.72E-37	2
Pcdh19	3.97E-77	########	0.544	0.066	6.65E-73	2
Tbcb	3.43E-17	########	0.913	0.642	5.74E-13	2
Ssr2	2.23E-16	########	0.883	0.506	3.73E-12	2
Mdh1	6.97E-15	########	0.893	0.554	1.17E-10	2
Cyc1	1.44E-15	########	0.835	0.518	2.41E-11	2
Colec11	0	########	0.262	0.001	0	2
Eif3i	1.11E-12	########	0.951	0.692	1.87E-08	2
Emc10	1.85E-17	########	0.854	0.44	3.09E-13	2
Tceb1	1.96E-15	########	0.932	0.709	3.28E-11	2

Acyp2	5.85E-82	########	0.621	0.084	9.78E-78	2
Psma3	2.10E-13	########	0.961	0.774	3.51E-09	2
Itpripl2	5.75E-21	########	0.777	0.348	9.62E-17	2
Etfb	8.42E-14	########	0.903	0.601	1.41E-09	2
Ctsk	1.11E-81	########	0.757	0.11	1.86E-77	2
Lin7a	0	########	0.437	0.007	0	2
Higd1a	2.01E-13	########	0.874	0.584	3.37E-09	2
Tusc5	0	########	0.524	0.003	0	2
Bin1	1.54E-15	########	0.777	0.391	2.57E-11	2
Spcs1	6.09E-14	########	0.893	0.681	1.02E-09	2
Itga8	1.93E-175	########	0.32	0.009	3.24E-171	2
Ptk2	1.71E-78	########	0.621	0.089	2.85E-74	2
Angptl2	7.98E-53	########	0.515	0.082	1.33E-48	2
Hipk3	3.07E-30	########	0.806	0.304	5.14E-26	2
Tcf15	3.06E-108	########	0.34	0.018	5.11E-104	2
Akap6	1.13E-219	########	0.32	0.007	1.89E-215	2
Mdh2	1.49E-14	########	0.942	0.724	2.49E-10	2
Prmt1	3.13E-20	########	0.874	0.43	5.23E-16	2
Usp2	1.46E-59	########	0.573	0.096	2.44E-55	2
Cisd1	3.71E-26	########	0.835	0.33	6.20E-22	2
Arl1	3.59E-17	########	0.874	0.512	6.01E-13	2
P3h3	8.96E-64	########	0.583	0.091	1.50E-59	2
Gnai1	1.14E-109	########	0.515	0.043	1.91E-105	2
Gatad1	1.26E-11	########	0.825	0.477	2.12E-07	2
Pfdn1	1.81E-17	########	0.835	0.468	3.03E-13	2
Dnajc3	2.79E-14	########	0.874	0.584	4.66E-10	2
Camk2g	1.06E-23	########	0.612	0.216	1.78E-19	2
Rcn2	1.58E-29	########	0.767	0.277	2.65E-25	2
Atp5g3	2.02E-13	########	0.942	0.744	3.38E-09	2
Dnpep	8.17E-26	########	0.767	0.312	1.37E-21	2
Tcaf1	1.22E-106	########	0.689	0.079	2.05E-102	2
Smarcd3	2.92E-185	########	0.534	0.027	4.89E-181	2
Itih5	3.26E-27	########	0.417	0.099	5.46E-23	2
Slc25a25	3.52E-45	########	0.631	0.143	5.89E-41	2
Wfs1	1.30E-79	########	0.485	0.054	2.17E-75	2
Adgra2	8.74E-88	########	0.573	0.067	1.46E-83	2
Dynlt3	9.12E-22	########	0.796	0.361	1.53E-17	2
Capn2	1.03E-18	########	0.835	0.377	1.73E-14	2
Fam69a	4.75E-33	########	0.835	0.294	7.94E-29	2
Cd59a	8.07E-79	########	0.476	0.051	1.35E-74	2
Cenpb	2.15E-18	########	0.864	0.461	3.60E-14	2
Dnajc10	1.48E-21	########	0.806	0.359	2.48E-17	2
Rbms2	4.87E-45	########	0.738	0.19	8.15E-41	2
Ssb	9.27E-14	########	0.942	0.692	1.55E-09	2
Mob2	1.60E-16	########	0.583	0.246	2.67E-12	2
Fstl3	2.38E-84	########	0.544	0.064	3.99E-80	2
Peak1	2.44E-31	########	0.786	0.271	4.08E-27	2
Pnkd	4.17E-22	########	0.786	0.366	6.99E-18	2
Fndc3b	2.35E-23	########	0.835	0.356	3.92E-19	2
Cct3	1.31E-17	########	0.913	0.505	2.20E-13	2
Pttg1ip	1.27E-11	########	0.796	0.425	2.12E-07	2
Eif4a1	3.29E-11	########	0.951	0.834	5.50E-07	2

Ddt	8.58E-20	########	0.835	0.4	1.44E-15	2
1110065P2	1.01E-39	########	0.796	0.236	1.68E-35	2
Ndufb8	3.17E-12	########	0.951	0.754	5.30E-08	2
Hsp90aa1	4.55E-11	########	0.971	0.806	7.62E-07	2
Capns1	1.07E-14	########	0.981	0.751	1.80E-10	2
Parp3	6.43E-39	########	0.709	0.194	1.08E-34	2
Mtch1	2.43E-15	########	0.913	0.591	4.07E-11	2
Gcnt2	6.13E-28	########	0.854	0.345	1.02E-23	2
Pla2g16	1.28E-33	########	0.709	0.208	2.14E-29	2
Atp8b1	1.08E-73	########	0.553	0.072	1.80E-69	2
Hsbp1	5.31E-14	########	0.922	0.66	8.88E-10	2
Dync1li2	3.83E-25	########	0.689	0.268	6.42E-21	2
Mrpl23	8.36E-15	########	0.874	0.569	1.40E-10	2
Gas1	1.09E-33	########	0.485	0.1	1.82E-29	2
Timm13	1.03E-12	########	0.961	0.694	1.72E-08	2
Tspan12	1.16E-179	########	0.573	0.032	1.93E-175	2
Cox5b	1.56E-12	########	0.971	0.863	2.61E-08	2
Trip6	5.85E-60	########	0.66	0.124	9.78E-56	2
Zc2hc1a	6.47E-83	########	0.553	0.068	1.08E-78	2
Suclg1	4.38E-17	########	0.893	0.531	7.33E-13	2
Ostc	7.51E-09	########	0.845	0.645	########	2
Sdhc	2.36E-18	########	0.845	0.449	3.95E-14	2
Gkap1	2.95E-49	########	0.602	0.126	4.93E-45	2
Kifap3	6.40E-39	########	0.641	0.17	1.07E-34	2
Cisd3	1.91E-51	########	0.709	0.158	3.20E-47	2
Rnf11	7.68E-21	########	0.816	0.386	1.29E-16	2
Rhob	9.62E-20	########	0.883	0.437	1.61E-15	2
Tbca	2.81E-14	########	0.913	0.718	4.69E-10	2
Endod1	2.71E-36	########	0.592	0.148	4.53E-32	2
Coq7	2.68E-34	########	0.748	0.235	4.48E-30	2
Tuba1c	4.58E-14	########	0.883	0.54	7.66E-10	2
Snta1	5.05E-71	########	0.65	0.103	8.46E-67	2
Wfdc1	3.36E-269	########	0.408	0.009	5.62E-265	2
Ubc	2.94E-12	########	0.981	0.95	4.92E-08	2
Inpp4b	3.07E-28	########	0.447	0.101	5.14E-24	2
Ptges3l	1.11E-112	########	0.592	0.057	1.85E-108	2
Emid1	1.75E-198	########	0.476	0.019	2.93E-194	2
Antxr1	5.26E-85	########	0.631	0.079	8.80E-81	2
Ano1	4.42E-191	########	0.408	0.014	7.40E-187	2
Shox2	6.62E-86	########	0.505	0.052	1.11E-81	2
Commd1	1.89E-14	########	0.874	0.56	3.15E-10	2
Cyp4b1	1.67E-121	########	0.33	0.015	2.80E-117	2
Sfr1	3.71E-14	########	0.951	0.703	6.21E-10	2
Hif1a	9.25E-12	########	0.903	0.639	1.55E-07	2
Sar1a	6.79E-17	########	0.874	0.515	1.14E-12	2
Ufm1	1.14E-17	########	0.883	0.516	1.90E-13	2
Pdia6	5.34E-10	########	0.874	0.607	8.94E-06	2
Hdgf	9.39E-15	########	0.893	0.572	1.57E-10	2
Ccdc3	1.39E-97	########	0.456	0.038	2.32E-93	2
Tubb3	4.74E-49	########	0.291	0.031	7.93E-45	2
St5	7.76E-82	########	0.505	0.056	1.30E-77	2
Slc6a17	3.96E-247	########	0.495	0.016	6.63E-243	2

Vkorc1	3.50E-25	########	0.728	0.272	5.86E-21	2
Cfdp1	2.38E-16	########	0.854	0.486	3.98E-12	2
2410015M2	4.35E-12	########	0.913	0.68	7.27E-08	2
Sik1	3.80E-15	########	0.748	0.35	6.35E-11	2
Tmed9	7.31E-12	########	0.922	0.67	1.22E-07	2
Adam9	1.96E-24	########	0.777	0.304	3.29E-20	2
Tns2	1.84E-85	########	0.544	0.061	3.08E-81	2
Angptl4	7.50E-26	########	0.534	0.148	1.25E-21	2
Ndufa7	3.04E-13	########	0.951	0.841	5.09E-09	2
Ndufaf2	1.44E-34	########	0.757	0.237	2.41E-30	2
Mrps28	4.58E-18	########	0.796	0.37	7.67E-14	2
Srp54b	2.66E-14	########	0.864	0.523	4.45E-10	2
1810037I17	1.33E-09	########	0.922	0.766	2.22E-05	2
Cox7c	2.44E-13	########	1	0.919	4.09E-09	2
Nucks1	1.40E-08	########	0.864	0.577	########	2
Coq10b	7.40E-17	########	0.835	0.442	1.24E-12	2
Acat1	4.01E-21	########	0.883	0.407	6.70E-17	2
Gstp1	1.08E-22	########	0.757	0.319	1.81E-18	2
Dctn6	1.07E-15	########	0.67	0.331	1.80E-11	2
Dtx3	8.30E-48	########	0.553	0.108	1.39E-43	2
B230219D2	2.04E-12	########	0.806	0.509	3.42E-08	2
Pop5	1.01E-17	########	0.806	0.401	1.68E-13	2
Unc45a	1.20E-35	########	0.602	0.156	2.01E-31	2
Ddb1	1.39E-18	########	0.845	0.42	2.32E-14	2
Ror1	7.85E-167	########	0.524	0.029	1.31E-162	2
Anapc5	1.72E-14	########	0.845	0.476	2.87E-10	2
Ddost	3.35E-12	########	0.854	0.545	5.60E-08	2
Sec61g	1.37E-09	########	0.942	0.865	2.29E-05	2
Eml1	2.74E-101	########	0.583	0.061	4.58E-97	2
Timp2	2.07E-17	########	0.961	0.546	3.47E-13	2
Srpx	1.16E-40	########	0.369	0.055	1.94E-36	2
Daam1	4.48E-34	########	0.612	0.173	7.50E-30	2
Ltbp1	5.11E-31	########	0.35	0.065	8.54E-27	2
Casp12	1.62E-101	########	0.583	0.06	2.71E-97	2
Cbx5	2.05E-20	########	0.709	0.272	3.43E-16	2
Daam2	7.89E-232	########	0.427	0.012	1.32E-227	2
Cacna1c	6.57E-217	########	0.553	0.024	1.10E-212	2
Pxdn	9.63E-71	########	0.612	0.089	1.61E-66	2
Fam104a	7.64E-15	########	0.922	0.644	1.28E-10	2
Zcrb1	4.17E-12	########	0.893	0.614	6.98E-08	2
Sox5	9.10E-188	########	0.544	0.027	1.52E-183	2
Txn1	8.50E-10	########	0.99	0.898	1.42E-05	2
Kdelr1	6.32E-13	########	0.854	0.559	1.06E-08	2
Il34	5.78E-189	########	0.408	0.014	9.67E-185	2
Ece1	3.44E-43	########	0.728	0.183	5.76E-39	2
Cfap36	9.98E-27	########	0.718	0.268	1.67E-22	2
Ccdc124	9.63E-20	########	0.903	0.47	1.61E-15	2
Pin1	1.18E-17	########	0.845	0.429	1.97E-13	2
Arhgap1	4.28E-21	########	0.767	0.35	7.16E-17	2
Pja2	2.92E-23	########	0.68	0.265	4.88E-19	2
Fkbp2	3.19E-12	########	0.903	0.562	5.34E-08	2
Lrrc58	6.72E-16	########	0.767	0.409	1.12E-11	2

Rnd3	4.76E-27	########	0.65	0.206	7.97E-23	2
Lims2	1.68E-111	########	0.417	0.027	2.82E-107	2
Mlec	2.09E-17	########	0.922	0.466	3.50E-13	2
Txndc17	1.07E-12	########	0.922	0.756	1.79E-08	2
Fyttd1	1.27E-22	########	0.757	0.309	2.12E-18	2
Smarcb1	2.50E-20	########	0.767	0.346	4.19E-16	2
Cnn2	7.83E-07	########	0.883	0.646	########	2
Pofut2	5.76E-27	########	0.806	0.305	9.64E-23	2
Pdia3	1.31E-09	########	0.942	0.753	2.19E-05	2
Banf1	1.83E-11	########	0.854	0.55	3.06E-07	2
Mrpl17	7.08E-15	########	0.845	0.462	1.18E-10	2
Uqcrh	1.53E-12	########	0.99	0.932	2.56E-08	2
Psmb2	1.99E-11	########	0.922	0.694	3.33E-07	2
Pcdh7	5.80E-16	########	0.32	0.092	9.70E-12	2
Vat1	2.09E-24	########	0.816	0.323	3.49E-20	2
Xirp1	0	########	0.252	0.001	0	2
Hspa9	6.96E-15	########	0.835	0.434	1.16E-10	2
Psma4	3.58E-11	########	0.903	0.693	5.99E-07	2
Tbx2	7.70E-228	########	0.388	0.01	1.29E-223	2
Dgcr6	7.88E-28	########	0.718	0.255	1.32E-23	2
Bmp2	4.55E-111	########	0.485	0.037	7.61E-107	2
Sec31a	3.32E-18	########	0.825	0.4	5.55E-14	2
Ccdc34	1.45E-12	########	0.641	0.281	2.42E-08	2
Gipc1	1.56E-22	########	0.748	0.305	2.62E-18	2
Mrps36	1.74E-16	########	0.816	0.42	2.91E-12	2
Nrep	1.40E-77	########	0.417	0.04	2.34E-73	2
Cpxm2	1.29E-88	########	0.35	0.024	2.15E-84	2
Acadvl	2.33E-24	########	0.786	0.314	3.90E-20	2
Ndufa2	5.91E-13	########	1	0.857	9.88E-09	2
Nr2f6	1.22E-37	########	0.689	0.191	2.03E-33	2
Twsg1	8.55E-54	########	0.67	0.137	1.43E-49	2
Uqcrfs1	4.40E-12	########	0.913	0.621	7.36E-08	2
Hspb6	4.64E-104	########	0.427	0.032	7.76E-100	2
Naf1	3.29E-34	########	0.592	0.157	5.50E-30	2
Cox4i1	1.37E-12	########	1	0.958	2.29E-08	2
Wasl	4.81E-28	########	0.699	0.237	8.05E-24	2
Dcbld2	4.27E-75	########	0.544	0.069	7.15E-71	2
Srm	9.85E-28	########	0.709	0.217	1.65E-23	2
Nptn	4.60E-12	########	0.893	0.613	7.70E-08	2
Mapre2	1.16E-14	########	0.767	0.407	1.95E-10	2
Emd	1.85E-15	########	0.864	0.497	3.10E-11	2
Lman1	3.82E-16	########	0.786	0.392	6.39E-12	2
Tspan9	2.60E-76	########	0.563	0.072	4.35E-72	2
Pros1	1.67E-35	########	0.748	0.213	2.79E-31	2
Mbnl2	3.87E-12	########	0.942	0.62	6.47E-08	2
Csnk1a1	7.20E-10	########	0.932	0.779	1.20E-05	2
1700020I1	1.41E-15	########	0.883	0.537	2.36E-11	2
Lrrc10b	0	########	0.301	0.003	0	2
Tmem38b	4.96E-20	########	0.757	0.331	8.29E-16	2
Ppp1r12c	1.05E-10	########	0.777	0.455	1.76E-06	2
Tnfsf12	7.10E-28	########	0.748	0.248	1.19E-23	2
Fdx1	1.26E-33	########	0.767	0.243	2.11E-29	2

Mmp14	3.71E-28	########	0.748	0.243	6.20E-24	2
Arl2	3.43E-34	########	0.65	0.187	5.73E-30	2
Thbd	7.06E-16	########	0.456	0.16	1.18E-11	2
Pam16	1.73E-20	########	0.796	0.352	2.90E-16	2
BC030336	1.29E-33	########	0.66	0.199	2.16E-29	2
Prdx1	2.29E-12	########	0.981	0.752	3.83E-08	2
Colgalt1	2.16E-14	########	0.796	0.411	3.61E-10	2
Rcn1	2.88E-29	########	0.602	0.17	4.82E-25	2
H1f0	1.93E-17	########	0.631	0.251	3.23E-13	2
1110001J0	8.10E-17	########	0.767	0.363	1.35E-12	2
Ndufb5	5.74E-12	########	0.99	0.725	9.60E-08	2
Ctps	1.39E-45	########	0.612	0.134	2.32E-41	2
Psmc3	5.12E-13	########	0.942	0.638	8.57E-09	2
Jam3	6.94E-114	########	0.553	0.049	1.16E-109	2
Fzd4	3.07E-73	########	0.534	0.067	5.14E-69	2
Sgcb	1.04E-52	########	0.65	0.133	1.74E-48	2
Arhgap5	1.80E-29	########	0.65	0.196	3.01E-25	2
Olfml2a	1.04E-95	########	0.291	0.015	1.74E-91	2
Ssbp4	3.36E-10	########	0.816	0.498	5.63E-06	2
Tyms	3.91E-14	########	0.524	0.206	6.54E-10	2
Sgce	9.12E-96	########	0.573	0.062	1.53E-91	2
Arhgap10	1.00E-28	########	0.689	0.224	1.68E-24	2
Ndufa8	6.43E-12	########	0.932	0.708	1.08E-07	2
Rsrc1	5.11E-13	########	0.748	0.384	8.54E-09	2
Pdha1	6.74E-16	########	0.806	0.451	1.13E-11	2
Psma5	3.88E-11	########	0.942	0.628	6.48E-07	2
Psmb4	1.10E-08	########	0.922	0.767	########	2
Ebna1bp2	1.25E-15	########	0.825	0.441	2.09E-11	2
Ndufa9	2.11E-17	########	0.864	0.45	3.54E-13	2
Slc2a4	0	########	0.369	0.002	0	2
Myo1d	2.02E-48	########	0.505	0.087	3.39E-44	2
Strn3	2.39E-13	########	0.806	0.47	3.99E-09	2
Plekhg2	6.14E-40	########	0.68	0.173	1.03E-35	2
Mir143hg	2.20E-154	########	0.427	0.02	3.69E-150	2
Atl3	9.40E-21	########	0.641	0.246	1.57E-16	2
Rtn4	5.17E-11	########	0.951	0.747	8.66E-07	2
Gnb1	4.75E-11	########	0.942	0.763	7.95E-07	2
Nudc	1.03E-14	########	0.942	0.534	1.72E-10	2
Cycs	1.86E-11	########	0.874	0.592	3.10E-07	2
Mrps24	1.47E-11	########	0.903	0.651	2.47E-07	2
Idh2	1.41E-17	########	0.777	0.351	2.36E-13	2
Psmd6	1.64E-12	########	0.825	0.543	2.75E-08	2
Phldb1	2.12E-53	########	0.553	0.096	3.55E-49	2
Bag1	6.23E-11	########	0.922	0.675	1.04E-06	2
Pkd1	1.33E-38	########	0.544	0.12	2.22E-34	2
A730049H(	2.39E-268	########	0.408	0.009	3.99E-264	2
Acp1	3.46E-16	########	0.854	0.477	5.79E-12	2
C1ra	4.88E-44	########	0.476	0.084	8.17E-40	2
Adrm1	1.24E-15	########	0.767	0.392	2.08E-11	2
Nhp2	6.37E-12	########	0.854	0.489	1.07E-07	2
Txndc5	3.68E-23	########	0.786	0.297	6.15E-19	2
Rab12	8.15E-28	########	0.767	0.272	1.36E-23	2

Gpc6	4.17E-57	########	0.379	0.045	6.98E-53	2
Lamb1	2.65E-55	########	0.573	0.094	4.44E-51	2
Plcb4	7.43E-56	########	0.534	0.086	1.24E-51	2
P3h4	9.19E-62	########	0.583	0.091	1.54E-57	2
Slc39a6	4.79E-35	########	0.65	0.177	8.02E-31	2
Msx1	1.45E-119	########	0.417	0.026	2.42E-115	2
Dbn1	4.15E-46	########	0.447	0.073	6.94E-42	2
Cdh2	8.57E-148	########	0.437	0.022	1.43E-143	2
Stip1	7.90E-18	########	0.777	0.366	1.32E-13	2
Timp1	7.51E-44	########	0.845	0.249	1.26E-39	2
Spry1	4.50E-91	########	0.505	0.05	7.54E-87	2
Tnfaip1	6.24E-28	########	0.66	0.209	1.04E-23	2
Sspn	1.32E-76	########	0.495	0.057	2.20E-72	2
Lzts2	1.33E-52	########	0.505	0.083	2.23E-48	2
Adgrl2	6.58E-42	########	0.573	0.12	1.10E-37	2
Armcx3	1.19E-31	########	0.709	0.215	1.99E-27	2
Sdhb	1.63E-10	########	0.942	0.663	2.72E-06	2
Atf4	1.45E-10	########	0.913	0.646	2.42E-06	2
Pmvk	2.38E-17	########	0.709	0.315	3.99E-13	2
Naca	5.72E-09	########	0.981	0.907	9.57E-05	2
Actr10	5.80E-13	########	0.864	0.529	9.70E-09	2
Gm10076	5.39E-08	########	0.99	0.888	########	2
Hspb8	2.29E-74	########	0.592	0.081	3.83E-70	2
Mgll	3.22E-15	########	0.379	0.126	5.38E-11	2
Txnl1	1.34E-11	########	0.893	0.584	2.23E-07	2
Nkd1	1.95E-305	########	0.466	0.01	3.27E-301	2
Yap1	2.91E-65	########	0.592	0.092	4.87E-61	2
Rpl14	6.22E-08	########	0.99	0.887	########	2
Prkg1	1.41E-276	########	0.485	0.013	2.35E-272	2
Adgrl1	8.57E-64	########	0.456	0.058	1.43E-59	2
Uba2	1.22E-12	########	0.796	0.429	2.04E-08	2
Mrpl54	7.29E-11	########	0.845	0.564	1.22E-06	2
Stom	2.48E-22	########	0.738	0.286	4.15E-18	2
Ddx1	9.16E-21	########	0.748	0.312	1.53E-16	2
Selk	5.80E-11	########	0.961	0.852	9.71E-07	2
Tln2	1.34E-54	########	0.573	0.097	2.25E-50	2
Dhrs4	1.42E-23	########	0.67	0.245	2.37E-19	2
Odc1	8.91E-12	########	0.777	0.41	1.49E-07	2
Pdap1	1.99E-09	########	0.893	0.623	3.33E-05	2
Bcas3	5.10E-17	########	0.379	0.115	8.54E-13	2
Med9	2.75E-27	########	0.699	0.244	4.61E-23	2
Runx1t1	2.77E-120	########	0.456	0.031	4.64E-116	2
Dlgap4	5.83E-17	########	0.718	0.34	9.75E-13	2
1700021F0	8.66E-37	########	0.68	0.193	1.45E-32	2
Rplp1	3.31E-10	########	1	0.975	5.54E-06	2
Casc4	6.94E-58	########	0.515	0.078	1.16E-53	2
Snrpb	1.99E-09	########	0.951	0.74	3.33E-05	2
Psmb7	1.33E-13	########	0.913	0.605	2.22E-09	2
Cmbl	6.82E-111	########	0.427	0.029	1.14E-106	2
Mrpl51	1.79E-11	########	0.874	0.523	3.00E-07	2
Ugp2	9.72E-09	########	0.757	0.512	########	2
Gm13861	8.20E-247	########	0.214	0.002	1.37E-242	2

Vcp	1.63E-09	########	0.932	0.724	2.73E-05	2
Park7	4.28E-10	########	0.951	0.728	7.16E-06	2
Cops6	6.93E-12	########	0.845	0.525	1.16E-07	2
Hmgn5	3.93E-15	########	0.738	0.333	6.58E-11	2
Polr2c	1.33E-15	########	0.854	0.473	2.23E-11	2
Psmb3	8.92E-09	########	0.932	0.786	########	2
Zbtb38	2.51E-17	########	0.631	0.273	4.21E-13	2
Bdnf	1.29E-180	########	0.33	0.009	2.16E-176	2
Nfatc4	2.27E-85	########	0.495	0.051	3.80E-81	2
N6amt2	2.50E-17	########	0.699	0.318	4.19E-13	2
Gspt1	1.70E-12	########	0.845	0.505	2.84E-08	2
Rasl12	1.04E-175	########	0.456	0.02	1.74E-171	2
Rrbp1	3.51E-08	########	0.951	0.787	########	2
Impad1	1.23E-21	########	0.718	0.289	2.06E-17	2
Pdia5	1.53E-47	########	0.534	0.101	2.57E-43	2
Ap1s1	3.44E-12	########	0.728	0.401	5.76E-08	2
Bmpr2	2.30E-18	########	0.748	0.321	3.85E-14	2
Psmc5	4.38E-13	########	0.864	0.556	7.33E-09	2
Mesdc2	3.19E-26	########	0.748	0.271	5.34E-22	2
Cxcl12	1.17E-31	########	0.524	0.118	1.96E-27	2
Ptpn9	2.64E-26	########	0.563	0.172	4.41E-22	2
Clstn1	1.84E-59	########	0.612	0.105	3.08E-55	2
Mef2c	1.89E-14	########	0.767	0.385	3.17E-10	2
Tmem109	7.77E-19	########	0.718	0.289	1.30E-14	2
Mrpl42	3.39E-14	########	0.854	0.462	5.67E-10	2
Rtn4rl1	4.26E-164	########	0.515	0.028	7.13E-160	2
Prdx2	2.28E-08	########	0.961	0.678	########	2
Mrps18a	1.72E-16	########	0.796	0.397	2.88E-12	2
Ssr3	2.25E-09	########	0.903	0.647	3.76E-05	2
Mrps34	8.97E-18	########	0.825	0.387	1.50E-13	2
Tulp4	2.32E-17	########	0.757	0.338	3.89E-13	2
Tusc3	3.03E-30	########	0.66	0.205	5.07E-26	2
Glo1	8.96E-19	########	0.796	0.362	1.50E-14	2
Pgf	2.09E-87	########	0.369	0.027	3.49E-83	2
Lap3	7.63E-25	########	0.748	0.275	1.28E-20	2
Chchd6	1.94E-47	########	0.612	0.129	3.24E-43	2
Psma2	8.49E-11	########	0.942	0.799	1.42E-06	2
Enpp2	1.84E-28	########	0.204	0.025	3.08E-24	2
Ndufb10	1.23E-08	########	0.942	0.746	########	2
Gucy1a2	0	########	0.485	0.005	0	2
Pmpcb	2.23E-20	########	0.786	0.345	3.74E-16	2
Eif4ebp1	1.54E-09	########	0.854	0.632	2.58E-05	2
Cyth3	4.26E-31	########	0.68	0.203	7.12E-27	2
Tbx3	3.26E-102	########	0.437	0.034	5.46E-98	2
Ate1	1.18E-17	########	0.728	0.341	1.98E-13	2
Sumo2	2.85E-08	########	0.932	0.782	########	2
Mapre1	6.12E-09	########	0.903	0.676	########	2
2-Mar	5.41E-13	########	0.777	0.402	9.05E-09	2
Dtymk	8.43E-13	########	0.767	0.375	1.41E-08	2
Trove2	3.91E-42	########	0.534	0.107	6.54E-38	2
Dclk1	2.04E-39	########	0.447	0.078	3.41E-35	2
Rpl12	2.21E-10	########	1	0.89	3.69E-06	2

Cox6b1	4.69E-10	########	0.971	0.911	7.85E-06	2
Tmed1	2.87E-31	########	0.612	0.17	4.81E-27	2
Kcnmb1	0	########	0.408	0.001	0	2
Camta1	5.22E-17	########	0.641	0.274	8.73E-13	2
Rpl7a	1.05E-07	########	0.99	0.92	########	2
Spry4	1.19E-117	########	0.437	0.029	1.98E-113	2
Frmd4a	1.00E-38	########	0.68	0.174	1.67E-34	2
Pth1r	6.03E-92	########	0.505	0.049	1.01E-87	2
Trp53inp2	2.91E-28	########	0.612	0.178	4.86E-24	2
Bola1	1.90E-17	########	0.68	0.288	3.19E-13	2
Rpl24	5.07E-08	########	0.99	0.961	########	2
1810022K0	2.43E-12	########	0.874	0.547	4.06E-08	2
Ndufb11	1.56E-10	########	0.981	0.826	2.62E-06	2
Vps29	4.23E-10	########	0.883	0.655	7.07E-06	2
Smim1	1.96E-92	########	0.544	0.056	3.27E-88	2
Bace1	2.86E-31	########	0.602	0.169	4.78E-27	2
Fh1	7.98E-18	########	0.786	0.363	1.33E-13	2
Ndufs7	8.88E-09	########	0.913	0.608	########	2
Ctdspl	9.22E-35	########	0.68	0.195	1.54E-30	2
Rbm3	5.61E-07	########	0.99	0.886	########	2
Armcx2	1.26E-57	########	0.524	0.081	2.11E-53	2
Mpc2	9.59E-10	########	0.913	0.722	1.61E-05	2
Ggt5	3.08E-14	########	0.437	0.159	5.16E-10	2
Snhg6	9.73E-16	########	0.728	0.342	1.63E-11	2
Rer1	1.09E-09	########	0.903	0.709	1.82E-05	2
2700029M0	2.00E-13	########	0.835	0.431	3.35E-09	2
Ppme1	6.35E-23	########	0.641	0.23	1.06E-18	2
Hadha	4.33E-12	########	0.874	0.531	7.24E-08	2
Atp9a	4.79E-109	########	0.544	0.048	8.01E-105	2
Fam132a	3.63E-37	########	0.67	0.181	6.08E-33	2
Mnd1	1.12E-21	########	0.311	0.072	1.88E-17	2
Mpc1	1.13E-09	########	0.961	0.719	1.88E-05	2
Mical1	1.54E-23	########	0.67	0.235	2.57E-19	2
Tfpi	8.54E-45	########	0.505	0.093	1.43E-40	2
Gtf2a2	8.93E-12	########	0.854	0.537	1.49E-07	2
P3h1	1.72E-47	########	0.563	0.105	2.89E-43	2
Spr	1.76E-17	########	0.68	0.285	2.95E-13	2
CR974586.	2.00E-160	########	0.233	0.005	3.35E-156	2
Sorbs1	2.28E-174	0.356084	0.408	0.016	3.81E-170	2
Cnnm2	3.23E-31	########	0.427	0.09	5.40E-27	2
Nov	3.18E-56	########	0.243	0.018	5.32E-52	2
Bola3	5.06E-15	########	0.738	0.348	8.46E-11	2
Rbm24	0	########	0.34	0.003	0	2
Kdelc2	1.29E-45	########	0.563	0.112	2.15E-41	2
Farp1	2.04E-67	########	0.495	0.065	3.41E-63	2
Bcap31	4.31E-10	########	0.913	0.639	7.21E-06	2
Ifngr2	1.98E-09	########	0.796	0.508	3.32E-05	2
Casq2	1.73E-96	########	0.184	0.006	2.89E-92	2
Kif1b	7.81E-15	########	0.777	0.396	1.31E-10	2
Cnbp	1.30E-09	########	0.961	0.707	2.17E-05	2
Ext2	3.63E-25	########	0.709	0.245	6.07E-21	2
Zfp771	4.93E-31	########	0.728	0.236	8.25E-27	2

Cmtm4	6.35E-47	########	0.495	0.089	1.06E-42	2
Sec24d	2.84E-30	########	0.612	0.173	4.74E-26	2
Ak3	5.37E-33	########	0.641	0.18	8.99E-29	2
Uap1	2.71E-18	########	0.68	0.288	4.54E-14	2
Chpf	5.01E-69	########	0.573	0.081	8.39E-65	2
Acadl	1.56E-13	########	0.854	0.491	2.61E-09	2
Cct5	1.52E-08	########	0.913	0.687	########	2
Dynll2	1.06E-16	########	0.718	0.312	1.77E-12	2
Prkrir	5.38E-20	########	0.66	0.251	9.01E-16	2
Lrrc59	2.75E-10	########	0.748	0.43	4.61E-06	2
Emc7	1.29E-10	########	0.864	0.586	2.16E-06	2
Nr4a3	1.25E-26	########	0.65	0.194	2.09E-22	2
Srsf3	1.60E-09	########	0.981	0.781	2.68E-05	2
Rhoq	2.68E-20	########	0.65	0.231	4.49E-16	2
Trappc6b	6.78E-12	########	0.806	0.515	1.13E-07	2
Birc2	2.95E-14	########	0.631	0.273	4.94E-10	2
Fst	4.13E-14	########	0.282	0.075	6.92E-10	2
P4hb	2.29E-10	########	0.932	0.644	3.83E-06	2
Smad7	9.15E-13	########	0.738	0.388	1.53E-08	2
Rab34	7.20E-47	########	0.592	0.112	1.20E-42	2
Ogdh	2.18E-10	########	0.777	0.464	3.65E-06	2
Dnaja1	4.73E-07	########	0.942	0.848	########	2
Eif1b	5.84E-09	########	0.903	0.619	9.78E-05	2
Cap2	0	########	0.379	0.003	0	2
Serf2	4.13E-08	########	1	0.969	########	2
Polr2f	3.35E-12	########	0.913	0.562	5.61E-08	2
Yif1a	2.08E-16	########	0.709	0.33	3.47E-12	2
Sptan1	8.93E-13	########	0.883	0.489	1.49E-08	2
Plat	1.72E-30	########	0.408	0.083	2.88E-26	2
Plce1	7.91E-87	########	0.427	0.038	1.32E-82	2
Endog	1.72E-29	########	0.583	0.165	2.87E-25	2
Stbd1	5.55E-118	########	0.359	0.019	9.29E-114	2
Med21	3.16E-15	########	0.806	0.418	5.29E-11	2
Ube2o	1.70E-13	########	0.369	0.125	2.85E-09	2
Mageh1	5.70E-74	########	0.515	0.063	9.54E-70	2
Uqcrc1	1.20E-09	########	0.874	0.543	2.01E-05	2
Limd1	1.24E-15	########	0.777	0.367	2.08E-11	2
Ugdh	3.77E-13	########	0.689	0.338	6.31E-09	2
Ndufc2	8.13E-09	########	0.932	0.677	########	2
Vdac3	2.50E-10	########	0.835	0.521	4.18E-06	2
Nme2	6.54E-21	########	0.689	0.264	1.09E-16	2
Timm8b	1.50E-13	########	0.942	0.559	2.50E-09	2
Fam114a1	4.86E-29	########	0.612	0.172	8.14E-25	2
Snrpd1	6.93E-08	########	0.845	0.57	########	2
Atp5j	1.77E-08	########	0.971	0.87	########	2
Slc25a5	2.68E-07	########	0.971	0.832	########	2
Ndufs3	9.69E-11	########	0.845	0.515	1.62E-06	2
Fam177a	2.16E-19	########	0.738	0.329	3.62E-15	2
Uqcrc2	6.42E-12	########	0.913	0.564	1.07E-07	2
Diablo	1.17E-22	########	0.718	0.274	1.96E-18	2
Anapc11	3.25E-11	########	0.845	0.504	5.44E-07	2
Bod1	3.61E-33	########	0.631	0.174	6.04E-29	2

Pura	3.82E-11	########	0.835	0.512	6.40E-07	2
Spry2	6.63E-35	########	0.534	0.125	1.11E-30	2
Scoc	6.05E-24	########	0.641	0.218	1.01E-19	2
Tmem123	4.71E-14	########	0.816	0.458	7.87E-10	2
Dut	2.58E-11	########	0.602	0.267	4.32E-07	2
Rdh5	2.47E-77	########	0.408	0.038	4.13E-73	2
Tjp1	1.97E-65	########	0.602	0.093	3.30E-61	2
Vimp	2.23E-10	########	0.893	0.582	3.74E-06	2
Hrct1	4.64E-182	########	0.456	0.019	7.76E-178	2
Mrpl28	3.65E-12	########	0.825	0.468	6.10E-08	2
Scara3	2.19E-42	########	0.369	0.055	3.66E-38	2
Immt	1.11E-11	########	0.806	0.459	1.86E-07	2
Cks1b	3.13E-12	########	0.592	0.249	5.23E-08	2
Psmd12	1.20E-09	########	0.835	0.533	2.00E-05	2
Lpcat3	5.39E-15	########	0.66	0.288	9.02E-11	2
Tmem205	3.95E-13	########	0.641	0.312	6.61E-09	2
Lrig1	3.07E-79	########	0.437	0.043	5.13E-75	2
Zbtb10	2.66E-30	########	0.369	0.072	4.45E-26	2
Serinc1	1.57E-10	########	0.854	0.557	2.62E-06	2
Map4k5	1.04E-42	########	0.602	0.133	1.74E-38	2
Vstm4	3.39E-132	########	0.427	0.024	5.68E-128	2
Ddx3y	1.36E-08	########	0.757	0.435	########	2
Pkia	1.85E-133	########	0.408	0.022	3.10E-129	2
Mrpl14	1.78E-08	########	0.854	0.586	########	2
Sfxn1	2.64E-14	########	0.67	0.313	4.41E-10	2
Fam120a	3.75E-12	########	0.835	0.462	6.28E-08	2
Psip1	7.44E-14	########	0.728	0.343	1.24E-09	2
Ppm1l	5.66E-62	########	0.359	0.036	9.48E-58	2
Tnfrsf1a	7.83E-09	########	0.913	0.642	########	2
Dnajc1	3.31E-15	########	0.767	0.371	5.54E-11	2
Siva1	6.00E-11	########	0.845	0.486	1.00E-06	2
Islr	4.82E-87	########	0.437	0.038	8.06E-83	2
Pvrl2	8.30E-54	########	0.476	0.071	1.39E-49	2
Pelo	8.71E-26	########	0.602	0.188	1.46E-21	2
Ctdsp2	8.37E-13	########	0.854	0.466	1.40E-08	2
Fam8a1	6.79E-31	########	0.621	0.179	1.14E-26	2
Heg1	4.68E-25	########	0.456	0.118	7.83E-21	2
Cdh13	2.01E-46	########	0.398	0.057	3.36E-42	2
Sec11a	3.81E-10	########	0.874	0.605	6.37E-06	2
Ahcyl1	4.90E-17	########	0.728	0.335	8.21E-13	2
Wls	9.45E-15	########	0.777	0.389	1.58E-10	2
Dtna	0	########	0.369	0.003	0	2
Rbm8a	4.17E-10	########	0.913	0.665	6.97E-06	2
Sod2	4.45E-13	########	0.922	0.606	7.45E-09	2
Mpdz	3.46E-55	########	0.495	0.075	5.79E-51	2
Abhd14a	4.67E-36	########	0.583	0.142	7.82E-32	2
Anxa7	6.45E-10	########	0.767	0.471	1.08E-05	2
Ak6	2.47E-15	########	0.728	0.351	4.13E-11	2
Nedd8	1.20E-08	########	0.971	0.845	########	2
Mrpl15	3.99E-13	########	0.816	0.43	6.68E-09	2
Psmd1	9.06E-13	########	0.854	0.46	1.52E-08	2
Smim11	3.20E-11	########	0.786	0.471	5.35E-07	2

Gm17501	3.50E-161	########	0.417	0.018	5.85E-157	2
Lrrc8a	1.03E-30	########	0.583	0.158	1.73E-26	2
Smim10l1	6.80E-20	########	0.738	0.304	1.14E-15	2
Zfp91	6.72E-10	########	0.913	0.628	1.12E-05	2
Tmem263	1.97E-29	########	0.621	0.174	3.30E-25	2
Psmc6	1.06E-09	########	0.874	0.574	1.78E-05	2
Oaf	4.89E-26	########	0.544	0.15	8.18E-22	2
Mrpl13	3.69E-16	########	0.796	0.376	6.17E-12	2
2610507B1	9.55E-08	########	0.699	0.39	########	2
Elovl1	2.65E-11	########	0.709	0.403	4.43E-07	2
Eci2	1.41E-14	########	0.738	0.364	2.37E-10	2
Eif4g2	9.37E-09	########	0.961	0.864	########	2
Ski	3.38E-13	########	0.767	0.382	5.65E-09	2
U2af1	5.29E-07	########	0.845	0.595	########	2
Rab18	4.66E-13	########	0.835	0.477	7.80E-09	2
Pkd2	2.92E-39	########	0.534	0.112	4.89E-35	2
Klhl23	5.76E-85	########	0.476	0.048	9.65E-81	2
Eif4g1	1.67E-08	########	0.806	0.538	########	2
Pbxip1	1.37E-07	########	0.767	0.489	########	2
Crtc3	6.53E-12	########	0.757	0.38	1.09E-07	2
Commd3	2.38E-11	########	0.816	0.469	3.99E-07	2
Cry2	8.95E-41	########	0.485	0.093	1.50E-36	2
Thbs3	1.37E-52	########	0.417	0.057	2.29E-48	2
R3hcc1	3.16E-37	########	0.524	0.118	5.29E-33	2
Sav1	7.34E-16	########	0.631	0.274	1.23E-11	2
Glrx2	1.11E-10	########	0.786	0.47	1.86E-06	2
Atp5a1	1.37E-07	########	0.951	0.794	########	2
1700025G(	5.69E-28	########	0.621	0.186	9.52E-24	2
Ncs1	3.79E-73	########	0.456	0.051	6.34E-69	2
Kirrel	4.37E-52	########	0.447	0.065	7.31E-48	2
Fez2	3.37E-22	########	0.612	0.209	5.64E-18	2
Ube2v2	5.29E-15	########	0.699	0.344	8.85E-11	2
Iah1	3.94E-12	########	0.728	0.375	6.59E-08	2
Lage3	8.35E-14	########	0.786	0.388	1.40E-09	2
Avpr1a	4.97E-91	########	0.33	0.021	8.31E-87	2
2310039H(	5.26E-17	########	0.68	0.289	8.80E-13	2
Praf2	5.76E-37	########	0.563	0.126	9.64E-33	2
Atp10a	8.58E-31	########	0.379	0.076	1.43E-26	2
Glrx3	3.70E-09	########	0.835	0.528	6.20E-05	2
Plekhh3	4.37E-71	########	0.417	0.044	7.31E-67	2
Enho	6.21E-87	########	0.35	0.025	1.04E-82	2
Arhgap35	1.43E-39	########	0.476	0.093	2.38E-35	2
Lsm4	1.32E-06	########	0.903	0.684	########	2
Acot9	2.44E-13	########	0.699	0.358	4.08E-09	2
Slirp	1.14E-10	########	0.835	0.497	1.90E-06	2
Slc50a1	7.43E-15	########	0.777	0.37	1.24E-10	2
Hnrnpab	5.21E-07	########	0.903	0.665	########	2
Pcmt1	4.45E-09	########	0.835	0.513	7.45E-05	2
Creb3l2	4.81E-31	########	0.553	0.14	8.05E-27	2
Bcar3	5.65E-42	########	0.388	0.06	9.45E-38	2
Itpr1	5.23E-19	########	0.592	0.21	8.74E-15	2
Nudt9	2.95E-18	########	0.631	0.256	4.93E-14	2

Mbd3	2.15E-10	########	0.748	0.401	3.59E-06	2
Alg14	1.69E-32	########	0.592	0.161	2.82E-28	2
Ntan1	4.04E-09	########	0.777	0.493	6.76E-05	2
Cct2	4.82E-08	########	0.922	0.677	########	2
Trak2	7.31E-14	########	0.583	0.25	1.22E-09	2
10-Sep	5.26E-41	########	0.563	0.123	8.81E-37	2
2700060E0	9.61E-08	########	0.951	0.685	########	2
Atp5k	2.59E-07	########	0.971	0.809	########	2
Pxdc1	3.02E-36	########	0.485	0.1	5.06E-32	2
Foxc1	4.86E-56	########	0.398	0.049	8.13E-52	2
Smap1	5.29E-10	########	0.864	0.603	8.85E-06	2
Atraid	4.71E-11	########	0.806	0.477	7.88E-07	2
Imp3	3.61E-09	########	0.835	0.495	6.04E-05	2
Rdx	4.40E-09	########	0.864	0.553	7.35E-05	2
Ptprn	9.81E-108	########	0.252	0.01	1.64E-103	2
Ccs	1.14E-17	########	0.699	0.297	1.91E-13	2
Eif6	2.75E-08	########	0.854	0.621	########	2
Mthfd2l	4.88E-37	########	0.534	0.119	8.16E-33	2
Synpo	1.05E-49	########	0.456	0.071	1.75E-45	2
Idh3g	4.07E-10	########	0.845	0.503	6.81E-06	2
Hars	2.92E-16	########	0.68	0.299	4.88E-12	2
Speg	7.03E-259	########	0.388	0.008	1.18E-254	2
Ict1	4.05E-12	########	0.835	0.463	6.77E-08	2
Tpd52l2	2.53E-11	########	0.767	0.425	4.23E-07	2
Rpn2	1.63E-07	########	0.845	0.564	########	2
Irs2	3.57E-11	########	0.563	0.263	5.98E-07	2
Gpm6b	3.56E-26	########	0.359	0.074	5.95E-22	2
Zfp664	1.06E-27	########	0.592	0.174	1.77E-23	2
Atp5l	4.45E-10	########	0.99	0.953	7.44E-06	2
Ndufa10	3.72E-10	########	0.864	0.52	6.23E-06	2
Ptpn11	8.10E-10	########	0.709	0.388	1.35E-05	2
Dzip1l	2.61E-127	########	0.456	0.029	4.36E-123	2
Fam124a	1.11E-143	########	0.379	0.017	1.85E-139	2
Ipo5	2.84E-14	########	0.67	0.28	4.75E-10	2
Tcf7l1	1.04E-82	########	0.485	0.05	1.74E-78	2
Cnpy2	1.21E-09	########	0.845	0.498	2.03E-05	2
Ccdc68	0	########	0.35	0.003	0	2
Il17rc	3.71E-56	########	0.495	0.075	6.21E-52	2
Setbp1	9.34E-53	########	0.466	0.068	1.56E-48	2
Guk1	1.58E-13	########	0.757	0.379	2.64E-09	2
Cops4	3.85E-12	########	0.757	0.413	6.44E-08	2
Mxd4	1.04E-11	########	0.796	0.431	1.74E-07	2
Tax1bp3	1.55E-15	########	0.709	0.322	2.60E-11	2
Srpr	4.12E-09	########	0.796	0.514	6.90E-05	2
Tmem63b	2.22E-22	########	0.515	0.161	3.72E-18	2
Rab10	4.40E-09	########	0.864	0.638	7.36E-05	2
Nol3	6.65E-83	########	0.495	0.052	1.11E-78	2
Mrps25	5.63E-14	########	0.748	0.371	9.42E-10	2
Dact3	2.13E-92	########	0.466	0.042	3.56E-88	2
Sema5a	3.61E-109	########	0.437	0.031	6.05E-105	2
Mrgprf	3.95E-150	########	0.427	0.021	6.62E-146	2
Actr1b	3.80E-22	########	0.66	0.236	6.36E-18	2

Trpc6	0	########	0.408	0	0	2
Gars	1.08E-14	########	0.718	0.341	1.81E-10	2
Snrnp27	2.82E-10	########	0.825	0.497	4.72E-06	2
Fgf1	6.41E-160	########	0.291	0.008	1.07E-155	2
Neo1	6.49E-52	########	0.427	0.061	1.09E-47	2
Fkbp11	4.63E-23	########	0.485	0.13	7.75E-19	2
Agbl3	8.80E-54	########	0.408	0.054	1.47E-49	2
Aga	1.34E-17	########	0.621	0.252	2.24E-13	2
Atp5f1	3.93E-08	########	0.981	0.883	########	2
Kank1	3.97E-52	########	0.301	0.03	6.64E-48	2
Cdr2l	8.88E-38	########	0.417	0.076	1.49E-33	2
4931406P1	8.40E-17	########	0.573	0.222	1.41E-12	2
Slc5a3	8.99E-23	########	0.583	0.187	1.50E-18	2
Plekha3	7.61E-28	########	0.67	0.21	1.27E-23	2
Micu3	2.17E-31	########	0.369	0.07	3.63E-27	2
Dusp8	3.01E-71	########	0.398	0.04	5.04E-67	2
Desi2	7.47E-15	########	0.709	0.334	1.25E-10	2
Slc4a3	3.51E-117	########	0.379	0.021	5.87E-113	2
Tmem159	2.36E-23	########	0.524	0.161	3.95E-19	2
Ndufs6	3.08E-08	########	0.932	0.691	########	2
Qsox1	1.41E-13	########	0.65	0.292	2.37E-09	2
I7Rn6	8.98E-14	########	0.728	0.354	1.50E-09	2
Nabp2	1.78E-11	########	0.68	0.355	2.98E-07	2
Psmd13	2.09E-08	########	0.883	0.598	########	2
Cct8	4.91E-08	########	0.893	0.658	########	2
Hspb11	6.88E-18	########	0.689	0.277	1.15E-13	2
Asph	1.46E-17	########	0.621	0.238	2.44E-13	2
Denr	1.83E-09	########	0.786	0.481	3.06E-05	2
Pkdcc	4.93E-51	########	0.505	0.08	8.25E-47	2
Grpel1	4.19E-10	########	0.835	0.531	7.00E-06	2
Tbl1x	1.56E-11	########	0.699	0.368	2.61E-07	2
Ecscr	1.28E-41	########	0.485	0.087	2.14E-37	2
Iscu	1.14E-08	########	0.883	0.607	########	2
Aamdc	1.79E-23	########	0.602	0.202	3.00E-19	2
Cyp20a1	1.47E-22	########	0.602	0.2	2.46E-18	2
Gas2l1	1.34E-18	########	0.544	0.183	2.25E-14	2
Trpc1	7.65E-159	########	0.456	0.023	1.28E-154	2
Purb	9.58E-07	########	0.913	0.708	########	2
Elof1	1.01E-09	########	0.806	0.489	1.68E-05	2
Atp5c1	2.14E-07	########	0.961	0.812	########	2
Nucb1	1.89E-09	########	0.854	0.498	3.16E-05	2
Kif3a	1.22E-32	########	0.563	0.142	2.04E-28	2
2210013O2	4.70E-15	########	0.583	0.243	7.86E-11	2
Rangrf	2.66E-43	########	0.553	0.111	4.44E-39	2
Kcne4	1.01E-56	########	0.34	0.035	1.69E-52	2
Appl1	2.27E-14	########	0.757	0.366	3.80E-10	2
Acads	2.37E-18	########	0.709	0.295	3.97E-14	2
Mrps17	2.56E-10	########	0.777	0.454	4.28E-06	2
Mrpl27	2.95E-12	########	0.738	0.385	4.93E-08	2
Tmem160	1.88E-06	########	0.922	0.678	########	2
Hsd17b10	5.18E-09	########	0.845	0.503	8.67E-05	2
Insig2	1.76E-23	########	0.612	0.204	2.95E-19	2

Chchd1	2.87E-07	########	0.835	0.541	########	2
Numbl	1.10E-54	########	0.524	0.083	1.84E-50	2
Btf3l4	2.65E-12	########	0.786	0.432	4.44E-08	2
Calm1	8.34E-07	########	0.981	0.98	########	2
Pla1a	2.61E-44	########	0.214	0.018	4.37E-40	2
Mfn2	6.72E-19	########	0.505	0.177	1.12E-14	2
Ppp1r1a	0	########	0.408	0.003	0	2
Ptprs	4.67E-24	########	0.534	0.156	7.81E-20	2
Chpt1	8.37E-20	########	0.573	0.206	1.40E-15	2
Mzt2	8.60E-18	########	0.544	0.2	1.44E-13	2
9530068E0	1.10E-09	########	0.786	0.489	1.84E-05	2
Jkamp	9.34E-17	########	0.65	0.278	1.56E-12	2
Siah2	4.62E-11	########	0.495	0.213	7.73E-07	2
Stoml2	1.08E-11	########	0.718	0.373	1.81E-07	2
Acadm	3.45E-13	########	0.777	0.392	5.77E-09	2
Glrx5	3.73E-12	########	0.845	0.465	6.25E-08	2
Tbx15	5.98E-63	########	0.456	0.056	1.00E-58	2
Ldb3	3.01E-251	########	0.262	0.003	5.04E-247	2
Itga9	1.71E-36	########	0.485	0.097	2.85E-32	2
Csgalnact1	4.85E-39	########	0.485	0.09	8.11E-35	2
Cdk4	4.81E-10	########	0.767	0.409	8.05E-06	2
Arl4a	7.24E-17	########	0.66	0.262	1.21E-12	2
Ephx3	3.17E-112	########	0.291	0.013	5.30E-108	2
Ube2j1	4.77E-12	########	0.728	0.387	7.98E-08	2
Svbp	5.59E-09	########	0.728	0.452	9.35E-05	2
Mrpl12	7.33E-11	########	0.816	0.448	1.23E-06	2
Tmem126a	5.53E-11	########	0.738	0.393	9.25E-07	2
Pla2r1	4.93E-189	########	0.379	0.012	8.26E-185	2
Sdpr	7.12E-50	########	0.408	0.055	1.19E-45	2
Susd5	0	########	0.233	0.001	0	2
Plcl1	3.76E-79	########	0.427	0.04	6.30E-75	2
Nhp2l1	9.99E-08	########	0.874	0.593	########	2
Gadd45gip	1.12E-11	########	0.748	0.386	1.87E-07	2
Ift74	2.66E-31	########	0.495	0.118	4.46E-27	2
6330403A0	0	########	0.243	0.001	0	2
Adam12	6.38E-31	########	0.369	0.068	1.07E-26	2
Tssc4	2.94E-17	########	0.573	0.222	4.91E-13	2
Sash1	3.23E-18	########	0.748	0.286	5.41E-14	2
Itpk1	3.88E-12	########	0.388	0.15	6.50E-08	2
Enpep	1.16E-60	########	0.233	0.016	1.95E-56	2
Has1	2.97E-22	########	0.33	0.071	4.97E-18	2
Mydgf	3.97E-08	########	0.816	0.514	########	2
Cox20	2.57E-09	########	0.816	0.479	4.29E-05	2
Thbs1	2.20E-11	########	0.777	0.389	3.68E-07	2
Ngfrap1	5.33E-15	########	0.631	0.255	8.92E-11	2
Tmem5	6.67E-14	########	0.631	0.276	1.12E-09	2
Pygb	1.13E-18	########	0.476	0.152	1.89E-14	2
Sema4c	2.63E-52	########	0.408	0.055	4.40E-48	2
Acadsb	1.53E-17	########	0.631	0.248	2.57E-13	2
Hadh	9.37E-20	########	0.709	0.273	1.57E-15	2
Dap3	8.31E-12	########	0.806	0.403	1.39E-07	2
Rora	6.32E-30	########	0.544	0.137	1.06E-25	2

Adgrf5	4.41E-59	########	0.34	0.033	7.38E-55	2
Ncl	8.45E-09	########	0.971	0.824	########	2
AW011738	1.34E-31	########	0.272	0.038	2.24E-27	2
Timm50	7.79E-12	########	0.621	0.286	1.30E-07	2
Ramp3	8.34E-12	########	0.165	0.036	1.39E-07	2
Snx7	8.30E-32	########	0.515	0.123	1.39E-27	2
Txn2	6.39E-09	########	0.825	0.539	0.000107	2
Erlec1	2.57E-11	########	0.709	0.369	4.30E-07	2
Pvrl3	4.00E-53	########	0.437	0.062	6.69E-49	2
Eif3l	9.28E-09	########	0.845	0.524	########	2
Copg1	6.92E-16	########	0.689	0.303	1.16E-11	2
Slc12a4	2.18E-29	########	0.476	0.115	3.65E-25	2
Zcchc24	1.16E-20	########	0.602	0.205	1.94E-16	2
Tbxa2r	1.88E-58	########	0.369	0.039	3.14E-54	2
Polr1d	2.68E-06	########	0.951	0.787	########	2
Tmco1	4.87E-08	########	0.883	0.586	########	2
Agrn	3.48E-60	########	0.398	0.045	5.82E-56	2
Pck2	1.05E-20	########	0.524	0.169	1.76E-16	2
Mrto4	1.60E-09	########	0.65	0.348	2.68E-05	2
Mrps7	2.29E-11	########	0.728	0.376	3.83E-07	2
Amotl1	6.65E-26	########	0.408	0.095	1.11E-21	2
Aimp1	1.48E-07	########	0.913	0.606	########	2
2310036O2	8.18E-07	########	0.913	0.772	########	2
Angpt1	1.18E-30	########	0.301	0.048	1.97E-26	2
Rabggtb	2.00E-19	########	0.68	0.253	3.34E-15	2
Ptges3	2.24E-07	########	0.874	0.641	########	2
Lims1	2.30E-08	########	0.864	0.582	########	2
Psmc2	1.50E-07	########	0.786	0.532	########	2
Mrpl55	3.56E-15	########	0.68	0.313	5.96E-11	2
Slc35f6	1.59E-20	########	0.689	0.257	2.67E-16	2
Pgp	1.64E-09	########	0.767	0.44	2.75E-05	2
Morf4l1	2.94E-07	########	0.951	0.898	########	2
Dgkb	2.69E-294	########	0.233	0.001	4.50E-290	2
Cdr2	1.17E-38	########	0.466	0.091	1.96E-34	2
Ly6k	7.55E-45	########	0.175	0.012	1.26E-40	2
Mnat1	3.73E-19	########	0.602	0.223	6.24E-15	2
Ptbp1	8.82E-10	########	0.864	0.509	1.48E-05	2
Serpini1	6.10E-45	########	0.262	0.026	1.02E-40	2
Socs5	5.20E-42	########	0.495	0.094	8.69E-38	2
Srf	5.08E-16	########	0.583	0.237	8.51E-12	2
Trp53i11	4.73E-15	########	0.291	0.078	7.91E-11	2
Hoxc9	1.23E-43	########	0.262	0.028	2.06E-39	2
Rit1	1.26E-14	########	0.689	0.318	2.10E-10	2
Copb1	3.17E-08	########	0.835	0.556	########	2
Pomgnt1	1.82E-31	########	0.427	0.092	3.05E-27	2
Sema3f	5.96E-48	########	0.359	0.046	9.98E-44	2
Copb2	5.40E-07	########	0.835	0.583	########	2
Mrps14	1.99E-07	########	0.893	0.649	########	2
Ubxn6	1.39E-09	########	0.728	0.404	2.32E-05	2
Vti1b	3.67E-11	########	0.825	0.495	6.14E-07	2
D10Wsu10	3.01E-11	########	0.515	0.232	5.03E-07	2
Man1a2	2.82E-11	########	0.767	0.411	4.72E-07	2

Fem1b	1.52E-12	########	0.515	0.216	2.55E-08	2
Ptprg	4.76E-50	########	0.417	0.06	7.97E-46	2
Tmed3	5.16E-11	########	0.883	0.523	8.64E-07	2
R3hdm2	1.09E-10	########	0.825	0.473	1.83E-06	2
Neurl1b	1.19E-72	########	0.32	0.025	1.99E-68	2
Rab6b	1.66E-34	########	0.359	0.06	2.79E-30	2
Naa38	1.44E-08	########	0.806	0.479	########	2
Mrps15	2.73E-10	########	0.854	0.483	4.57E-06	2
Aimp2	6.74E-20	########	0.612	0.218	1.13E-15	2
Cuedc2	1.80E-08	########	0.864	0.553	########	2
Cdc42bpb	1.52E-26	########	0.534	0.145	2.54E-22	2
Hspd1	3.75E-10	########	0.874	0.537	6.28E-06	2
Fdx1l	4.51E-12	########	0.66	0.31	7.54E-08	2
Glt8d1	4.00E-18	########	0.466	0.155	6.70E-14	2
Smim12	2.83E-15	########	0.689	0.317	4.73E-11	2
Erdr1	3.70E-07	########	0.689	0.416	########	2
Afap1l2	1.81E-118	########	0.311	0.014	3.03E-114	2
Psma6	2.59E-06	########	0.883	0.676	########	2
Pstk	9.30E-20	########	0.485	0.155	1.56E-15	2
Ubr1	3.02E-21	########	0.602	0.209	5.05E-17	2
Tanc1	6.73E-22	########	0.398	0.103	1.13E-17	2
Naalad2	1.37E-108	########	0.35	0.019	2.30E-104	2
Ccdc102a	1.50E-52	########	0.476	0.073	2.51E-48	2
Hspa13	9.90E-19	########	0.621	0.235	1.66E-14	2
Coa3	1.31E-07	########	0.874	0.565	########	2
Hspe1	1.31E-06	########	0.903	0.674	########	2
Golt1b	6.23E-18	########	0.621	0.239	1.04E-13	2
Gorasp2	5.55E-10	########	0.67	0.371	9.29E-06	2
2610524H(	1.46E-21	########	0.563	0.184	2.44E-17	2
Usmg5	1.40E-07	########	0.942	0.782	########	2
Hadhb	1.53E-09	########	0.738	0.42	2.57E-05	2
Zfp503	1.80E-27	########	0.427	0.099	3.00E-23	2
Nop58	4.67E-12	########	0.767	0.363	7.81E-08	2
Mmadhc	9.36E-11	########	0.65	0.346	1.57E-06	2
Slco3a1	1.06E-13	########	0.485	0.197	1.77E-09	2
Nicn1	2.35E-65	########	0.427	0.049	3.93E-61	2
Ccnd1	4.26E-13	########	0.456	0.166	7.12E-09	2
Snhg12	5.54E-14	########	0.602	0.252	9.27E-10	2
Dazap1	1.02E-08	########	0.689	0.403	########	2
Tmem100	3.00E-34	########	0.301	0.044	5.02E-30	2
Phpt1	4.86E-18	########	0.495	0.165	8.13E-14	2
Zeb1	7.42E-17	########	0.602	0.223	1.24E-12	2
Fuom	7.90E-15	########	0.476	0.177	1.32E-10	2
1110004F1	1.57E-08	########	0.913	0.582	########	2
Hotairm1	3.28E-39	########	0.447	0.08	5.49E-35	2
Adamts12	7.45E-66	########	0.34	0.031	1.25E-61	2
Pard3	3.21E-50	########	0.427	0.062	5.36E-46	2
Ergic3	3.39E-08	########	0.806	0.492	########	2
Ccdc50	3.60E-12	########	0.816	0.434	6.02E-08	2
Creb3l1	7.50E-20	########	0.379	0.1	1.25E-15	2
B4galt2	5.44E-51	########	0.398	0.054	9.11E-47	2
Klhdc2	5.83E-14	########	0.689	0.322	9.75E-10	2

Grb10	7.85E-41	########	0.524	0.098	1.31E-36	2
Yae1d1	1.67E-27	########	0.524	0.141	2.79E-23	2
Kif1c	3.39E-18	########	0.573	0.205	5.67E-14	2
Emc8	4.29E-11	########	0.68	0.345	7.18E-07	2
Cacnb3	2.31E-73	########	0.369	0.033	3.86E-69	2
Fam132b	4.52E-85	########	0.175	0.006	7.56E-81	2
Epb41l1	3.78E-31	########	0.485	0.111	6.32E-27	2
Mtss1l	1.40E-130	########	0.398	0.021	2.35E-126	2
Raph1	1.89E-14	########	0.65	0.291	3.15E-10	2
1110038F1	2.79E-11	########	0.592	0.286	4.67E-07	2
Sh3d19	8.08E-37	########	0.456	0.091	1.35E-32	2
Mrpl35	1.09E-11	########	0.728	0.372	1.82E-07	2
Sacs	2.71E-31	########	0.485	0.112	4.54E-27	2
Slc25a17	2.59E-14	########	0.583	0.251	4.34E-10	2
Ilkap	5.99E-13	########	0.757	0.378	1.00E-08	2
Slc39a1	5.68E-12	########	0.796	0.41	9.50E-08	2
Asap2	4.11E-22	########	0.359	0.087	6.88E-18	2
Ddit4l	4.45E-101	########	0.301	0.015	7.45E-97	2
Skp1a	4.16E-09	########	0.845	0.537	6.95E-05	2
Tmem237	1.86E-38	########	0.456	0.087	3.11E-34	2
Drg1	1.08E-12	########	0.689	0.337	1.81E-08	2
Phb2	2.30E-07	########	0.903	0.594	########	2
Ldha	3.34E-07	########	1	0.838	########	2
Tob2	1.52E-10	########	0.864	0.489	2.55E-06	2
Hoxc10	1.66E-51	########	0.34	0.039	2.79E-47	2
Spag7	3.97E-12	########	0.796	0.432	6.65E-08	2
Ufc1	7.38E-07	########	0.883	0.602	########	2
Tomm5	1.14E-07	########	0.845	0.555	########	2
Fabp4	0	5.735737	0.823	0.127	0	3
Ctla2a	0	4.674156	0.956	0.091	0	3
Aqp1	0	3.667096	0.703	0.035	0	3
Igfbp7	2.68E-297	3.635995	0.867	0.146	4.48E-293	3
Tm4sf1	0	3.448803	0.877	0.101	0	3
Egfl7	0	3.174106	0.934	0.053	0	3
Cdh5	0	3.074425	0.87	0.02	0	3
Plvap	0	2.944668	0.69	0.022	0	3
Gng11	0	2.916729	0.889	0.128	0	3
Tmem252	0	2.899958	0.731	0.004	0	3
Pecam1	0	2.858365	0.946	0.139	0	3
Gpihbp1	0	2.834254	0.57	0.004	0	3
Col4a1	0	2.779309	0.864	0.106	0	3
Cldn5	0	2.743769	0.595	0.003	0	3
Ecscr	0	2.702628	0.826	0.064	0	3
Ly6c1	7.20E-242	2.636403	0.709	0.108	1.20E-237	3
Cav1	5.94E-292	2.60915	0.832	0.134	9.95E-288	3
Ramp2	0	2.521858	0.816	0.06	0	3
Flt1	0	2.519649	0.734	0.026	0	3
Esam	0	2.504655	0.788	0.02	0	3
Rgs5	2.72E-113	2.385751	0.231	0.02	4.54E-109	3
Sdpr	0	2.319438	0.807	0.031	0	3
Eng	0	2.301217	0.851	0.1	0	3
Ptprb	0	2.282574	0.797	0.004	0	3

Ece1	1.99E-280	2.223167	0.848	0.165	3.33E-276	3
Adamts1	3.74E-294	2.212042	0.81	0.116	6.25E-290	3
Cd36	6.82E-193	2.201631	0.658	0.109	1.14E-188	3
Crip2	0	2.164105	0.861	0.119	0	3
Emcn	0	2.149911	0.81	0.006	0	3
Sparcl1	0	2.14232	0.642	0.042	0	3
Col4a2	0	2.139408	0.807	0.094	0	3
Adgrf5	0	2.12378	0.813	0.007	0	3
Pdlim1	6.90E-200	2.093194	0.842	0.24	1.15E-195	3
Cd34	0	2.082101	0.797	0.085	0	3
Prkcdbp	1.93E-269	2.052403	0.816	0.133	3.24E-265	3
Fkbp1a	1.74E-136	1.978944	0.921	0.546	2.91E-132	3
Timp3	0	1.9681	0.731	0.076	0	3
Ptrf	3.37E-294	1.94246	0.851	0.137	5.63E-290	3
Epas1	0	1.92641	0.778	0.049	0	3
Hspb1	5.09E-255	1.8889	0.769	0.119	8.52E-251	3
Sparc	3.55E-189	1.869592	0.864	0.189	5.94E-185	3
Tcf4	4.62E-144	1.866547	0.927	0.452	7.73E-140	3
Ly6a	9.15E-122	1.856433	0.778	0.247	1.53E-117	3
Sptbn1	1.78E-130	1.855627	0.927	0.537	2.97E-126	3
Selp	2.21E-220	1.854343	0.43	0.038	3.70E-216	3
Rasip1	0	1.809277	0.791	0.029	0	3
Id3	7.01E-132	1.800339	0.81	0.259	1.17E-127	3
Egr1	2.87E-60	1.772489	0.769	0.353	4.81E-56	3
Mgll	1.32E-265	1.762851	0.725	0.107	2.21E-261	3
Jun	7.24E-66	1.725475	0.921	0.54	1.21E-61	3
Myct1	0	1.720949	0.785	0.004	0	3
S1pr1	2.29E-256	1.717637	0.889	0.164	3.84E-252	3
Kitl	0	1.712747	0.709	0.063	0	3
Mast4	0	1.712286	0.807	0.111	0	3
Tspan7	0	1.70441	0.744	0.025	0	3
Jam2	0	1.704366	0.677	0.022	0	3
Kdr	0	1.700143	0.769	0.009	0	3
Tinagl1	7.28E-306	1.697961	0.744	0.096	1.22E-301	3
Iigp1	0	1.687665	0.554	0.033	0	3
Col18a1	3.05E-279	1.680101	0.646	0.073	5.10E-275	3
Nfib	1.29E-264	1.668349	0.813	0.127	2.17E-260	3
Slc9a3r2	0	1.661276	0.693	0.046	0	3
Ehd2	0	1.660444	0.782	0.109	0	3
S100a16	2.18E-276	1.64222	0.82	0.125	3.64E-272	3
Hes1	3.53E-141	1.63058	0.791	0.226	5.91E-137	3
2200002D0	0	1.621409	0.658	0.04	0	3
Cd81	5.32E-131	1.613995	0.883	0.358	8.90E-127	3
Ackr1	1.39E-273	1.597086	0.278	0.009	2.32E-269	3
Wwtr1	0	1.593466	0.813	0.107	0	3
Adgrl4	0	1.58544	0.75	0.02	0	3
Rhoc	5.52E-158	1.570574	0.772	0.207	9.23E-154	3
Id1	7.03E-116	1.553148	0.744	0.243	1.18E-111	3
Slco2a1	1.94E-252	1.518673	0.484	0.043	3.25E-248	3
Cxcl12	3.27E-103	1.509123	0.509	0.108	5.47E-99	3
Hspg2	8.91E-261	1.505004	0.744	0.11	1.49E-256	3
Icam2	2.01E-251	1.50439	0.785	0.136	3.37E-247	3

Cyyr1	0	1.481248	0.747	0.004	0	3
Apold1	0	1.477337	0.728	0.027	0	3
Mmrn2	0	1.470897	0.728	0.015	0	3
Ppp1r2	1.63E-105	1.466559	0.911	0.629	2.72E-101	3
Abcg2	0	1.44632	0.785	0.084	0	3
Pim3	1.30E-113	1.435088	0.756	0.274	2.18E-109	3
Sox4	2.34E-200	1.423835	0.687	0.12	3.91E-196	3
Plcb4	0	1.42121	0.665	0.07	0	3
Grrp1	0	1.42074	0.652	0.003	0	3
Adamts9	0	1.406236	0.696	0.046	0	3
Cd93	1.35E-107	1.396926	0.832	0.32	2.26E-103	3
1810011O1	7.14E-290	1.395127	0.646	0.069	1.19E-285	3
Aplnr	0	1.39219	0.513	0.009	0	3
F11r	3.87E-303	1.385755	0.804	0.117	6.48E-299	3
Fermt2	2.08E-276	1.358132	0.797	0.111	3.47E-272	3
Ier5l	5.68E-159	1.352449	0.614	0.116	9.50E-155	3
Tcf15	0	1.35096	0.424	0.007	0	3
Dst	2.11E-190	1.347689	0.769	0.154	3.53E-186	3
Nedd4	2.78E-154	1.341307	0.908	0.282	4.65E-150	3
Heg1	1.98E-268	1.33841	0.725	0.1	3.32E-264	3
Cyr61	2.15E-189	1.337907	0.636	0.1	3.60E-185	3
Aplp2	9.92E-91	1.333809	0.889	0.514	1.66E-86	3
Stmn2	0	1.331562	0.329	0.006	0	3
Ppic	3.19E-223	1.327003	0.747	0.127	5.33E-219	3
Tie1	0	1.326418	0.769	0.009	0	3
Fam167b	0	1.321777	0.509	0.005	0	3
Zbtb20	6.01E-116	1.321136	0.861	0.313	1.01E-111	3
Crim1	0	1.319019	0.741	0.087	0	3
Clic4	1.01E-104	1.313276	0.873	0.403	1.70E-100	3
Lrg1	6.08E-39	1.310937	0.696	0.403	1.02E-34	3
Cdh13	0	1.307049	0.699	0.037	0	3
Col15a1	0	1.304033	0.535	0.028	0	3
Podxl	0	1.284628	0.677	0.003	0	3
Ctla2b	9.22E-235	1.281677	0.68	0.095	1.54E-230	3
Cd200	0	1.281054	0.747	0.066	0	3
Arhgap31	5.63E-123	1.278303	0.782	0.29	9.43E-119	3
Scarb1	1.21E-83	1.268472	0.646	0.236	2.03E-79	3
Rbp1	7.16E-110	1.264818	0.449	0.083	1.20E-105	3
Prex2	0	1.259406	0.712	0.01	0	3
Cald1	3.64E-189	1.258041	0.769	0.136	6.09E-185	3
Tsc22d1	1.22E-134	1.257575	0.747	0.204	2.05E-130	3
Mcam	0	1.256824	0.684	0.043	0	3
Cav2	1.02E-212	1.244019	0.747	0.14	1.70E-208	3
Mllt4	5.84E-200	1.240212	0.741	0.146	9.78E-196	3
Ehd4	2.32E-57	1.236297	0.785	0.449	3.88E-53	3
Hbegf	1.01E-134	1.23492	0.63	0.141	1.68E-130	3
Tjp1	0	1.223063	0.706	0.076	0	3
Adgrg1	0	1.215878	0.712	0.043	0	3
Nts	7.01E-215	1.213164	0.177	0.003	1.17E-210	3
Rbpms	3.30E-218	1.209747	0.788	0.159	5.52E-214	3
4931406P1	2.36E-166	1.2037	0.778	0.205	3.95E-162	3
Tshz2	9.03E-301	1.200307	0.706	0.08	1.51E-296	3

Tuba1a	3.78E-64	1.189241	0.794	0.449	6.32E-60	3
Fbln2	4.63E-198	1.183381	0.718	0.112	7.75E-194	3
Tspan13	2.49E-83	1.18282	0.772	0.278	4.16E-79	3
Fbln5	1.20E-176	1.177859	0.481	0.062	2.01E-172	3
Luzp1	1.17E-161	1.177313	0.785	0.221	1.95E-157	3
Plk2	1.96E-121	1.177164	0.668	0.168	3.28E-117	3
Gnas	1.73E-103	1.170256	0.968	0.88	2.89E-99	3
Prnp	6.27E-131	1.169781	0.684	0.175	1.05E-126	3
Gimap6	1.25E-215	1.161265	0.858	0.15	2.09E-211	3
Arhgap29	0	1.150528	0.718	0.081	0	3
Ptms	1.09E-82	1.148887	0.88	0.489	1.83E-78	3
Csrp2	1.02E-84	1.136656	0.712	0.278	1.71E-80	3
C1qtnf9	0	1.135903	0.472	0.003	0	3
Ablim1	1.21E-127	1.133315	0.737	0.183	2.03E-123	3
Sgk1	4.54E-80	1.130763	0.82	0.383	7.59E-76	3
Cyb5r3	4.98E-91	1.117936	0.81	0.365	8.34E-87	3
Cd151	2.93E-170	1.108725	0.747	0.186	4.90E-166	3
Tmem88	1.52E-175	1.108152	0.712	0.15	2.54E-171	3
Lyve1	1.94E-12	1.104884	0.12	0.04	3.24E-08	3
Ppfibp1	7.76E-196	1.10237	0.747	0.154	1.30E-191	3
Ebf1	2.43E-179	1.100776	0.788	0.149	4.07E-175	3
Akap12	3.36E-250	1.099649	0.598	0.066	5.62E-246	3
Nr2f2	1.87E-287	1.097135	0.611	0.06	3.14E-283	3
Rhoj	7.32E-236	1.096697	0.709	0.103	1.23E-231	3
Cnn3	4.71E-209	1.095665	0.756	0.134	7.89E-205	3
Cd300lg	3.64E-139	1.094379	0.472	0.076	6.09E-135	3
Uaca	0	1.086271	0.668	0.063	0	3
Timp4	7.51E-258	1.080081	0.218	0.004	1.26E-253	3
Ctnnb1	7.71E-91	1.079196	0.911	0.606	1.29E-86	3
Vwf	0	1.078718	0.497	0.021	0	3
Btbd3	0	1.078649	0.652	0.058	0	3
Apbb2	3.42E-276	1.078305	0.674	0.084	5.73E-272	3
Hsp90ab1	1.41E-86	1.075488	0.994	0.914	2.35E-82	3
Tns1	4.92E-144	1.070162	0.712	0.166	8.23E-140	3
Sema6a	0	1.061158	0.646	0.016	0	3
Mgp	3.18E-71	1.056454	0.424	0.104	5.32E-67	3
Ybx1	3.04E-59	1.048442	0.987	0.909	5.09E-55	3
Ltbp4	0	1.047863	0.652	0.053	0	3
Dock9	0	1.043811	0.674	0.042	0	3
Rhob	3.89E-61	1.041553	0.794	0.43	6.51E-57	3
Sox17	0	1.03706	0.535	0.001	0	3
Lmna	8.17E-58	1.036497	0.829	0.438	1.37E-53	3
Vwa1	0	1.034275	0.535	0.02	0	3
Itga6	4.89E-137	1.030569	0.775	0.208	8.19E-133	3
Actn4	9.28E-77	1.018045	0.864	0.549	1.55E-72	3
Vamp5	1.66E-110	1.017588	0.728	0.248	2.77E-106	3
Itm2c	1.05E-62	1.01395	0.788	0.457	1.76E-58	3
Ier2	6.95E-25	1.008242	0.839	0.69	1.16E-20	3
Palmd	0	1.006385	0.604	0.029	0	3
Atf3	1.52E-65	1.002498	0.769	0.345	2.54E-61	3
Tek	0	1.002136	0.633	0.01	0	3
Ctnna1	9.94E-79	########	0.804	0.413	1.66E-74	3

Ndufa8	6.21E-56	########	0.861	0.705	1.04E-51	3
Emp1	1.04E-58	########	0.778	0.367	1.75E-54	3
Snrk	1.75E-89	########	0.766	0.298	2.93E-85	3
Sepw1	1.37E-67	########	0.934	0.664	2.28E-63	3
Itgb1	5.21E-62	########	0.911	0.641	8.72E-58	3
Myo10	2.19E-169	########	0.601	0.103	3.67E-165	3
Fosb	2.09E-41	########	0.842	0.453	3.50E-37	3
Vim	8.23E-36	########	0.968	0.897	1.38E-31	3
Sox7	0	########	0.639	0.01	0	3
Smad1	4.56E-109	########	0.649	0.185	7.63E-105	3
Igfbp3	1.14E-90	########	0.266	0.034	1.91E-86	3
S100a13	1.73E-65	########	0.911	0.767	2.89E-61	3
Csrp1	1.29E-106	########	0.759	0.291	2.15E-102	3
Bst2	2.01E-78	########	0.883	0.495	3.36E-74	3
Plxnd1	3.57E-111	########	0.703	0.227	5.98E-107	3
Calcrl	4.76E-189	########	0.696	0.129	7.96E-185	3
Sele	0	########	0.313	0.006	0	3
Npdc1	3.79E-210	########	0.696	0.11	6.34E-206	3
Scn1b	5.38E-153	########	0.598	0.114	9.00E-149	3
Serpinh1	1.57E-176	########	0.801	0.15	2.62E-172	3
Plpp1	1.68E-222	########	0.661	0.093	2.82E-218	3
Nid1	2.87E-221	########	0.684	0.098	4.81E-217	3
Nus1	6.73E-63	########	0.759	0.411	1.13E-58	3
Yes1	2.69E-293	########	0.652	0.071	4.51E-289	3
Plpp3	5.95E-184	########	0.693	0.115	9.96E-180	3
Mecom	0	########	0.611	0.011	0	3
Sox18	0	########	0.627	0.001	0	3
9430020K0	1.14E-283	########	0.56	0.051	1.92E-279	3
Ifitm3	8.13E-46	########	0.949	0.79	1.36E-41	3
Pdzd2	0	########	0.563	0.033	0	3
Slc25a4	2.67E-66	########	0.854	0.508	4.46E-62	3
Fmo2	0	########	0.329	0.011	0	3
Il6st	6.78E-76	########	0.722	0.277	1.14E-71	3
Mtch1	5.11E-64	########	0.845	0.585	8.54E-60	3
Tubb5	1.34E-29	########	0.87	0.684	2.24E-25	3
Kank3	0	########	0.652	0.053	0	3
Sult1a1	2.65E-189	########	0.386	0.034	4.43E-185	3
Ndrg1	1.03E-60	########	0.741	0.349	1.72E-56	3
Fos	4.32E-28	########	0.842	0.586	7.23E-24	3
Cdc42ep3	2.36E-53	########	0.753	0.404	3.95E-49	3
Mfge8	1.24E-201	########	0.747	0.135	2.08E-197	3
4-Sep	0	########	0.475	0.022	0	3
Bvht	0	########	0.633	0.049	0	3
Serpinb6a	1.59E-65	########	0.816	0.392	2.66E-61	3
Dlc1	9.15E-232	########	0.636	0.084	1.53E-227	3
Ddah2	9.41E-186	########	0.712	0.134	1.57E-181	3
Cracr2b	3.22E-306	########	0.639	0.064	5.38E-302	3
Bcam	0	########	0.601	0.039	0	3
Sema3f	0	########	0.582	0.03	0	3
Erg	0	########	0.68	0.062	0	3
Tpm1	8.09E-103	########	0.766	0.268	1.35E-98	3
Arhgef12	1.28E-147	########	0.699	0.164	2.14E-143	3

Mmrn1	1.33E-177	########	0.123	0.001	2.22E-173	3
Elk3	6.69E-91	########	0.794	0.334	1.12E-86	3
Ctnnbip1	3.75E-59	########	0.715	0.341	6.27E-55	3
Lrrc8c	2.53E-118	########	0.722	0.208	4.24E-114	3
Cd9	2.62E-55	########	0.892	0.537	4.38E-51	3
Fgd5	0	########	0.642	0.01	0	3
Ptma	4.61E-69	########	0.994	0.867	7.71E-65	3
Lims2	0	########	0.516	0.014	0	3
Clec14a	0	########	0.541	0.01	0	3
Tmem176b	1.82E-85	########	0.769	0.284	3.05E-81	3
Ppia	4.65E-56	########	0.991	0.901	7.79E-52	3
Gadd45g	5.97E-50	########	0.589	0.241	9.99E-46	3
Nos3	0	########	0.592	0.011	0	3
Pcdh17	0	########	0.538	0.015	0	3
Lmo1	2.10E-84	########	0.633	0.222	3.51E-80	3
Gja1	1.27E-104	########	0.684	0.187	2.13E-100	3
Utrn	3.86E-72	########	0.744	0.309	6.46E-68	3
Slfn5	5.48E-54	########	0.674	0.283	9.17E-50	3
Meox2	1.65E-242	########	0.557	0.06	2.76E-238	3
Afap1l1	0	########	0.579	0.019	0	3
Mprip	5.72E-96	########	0.782	0.312	9.57E-92	3
Sncg	0	########	0.522	0.027	0	3
Adgrl2	9.76E-186	########	0.633	0.107	1.63E-181	3
C130074G	0	########	0.579	0.005	0	3
Csf3	1.73E-113	########	0.31	0.037	2.90E-109	3
Fzd4	0	########	0.614	0.053	0	3
Procr	2.54E-163	########	0.541	0.084	4.24E-159	3
Flnb	4.37E-185	########	0.668	0.12	7.31E-181	3
mt-Atp6	1.29E-46	########	0.975	0.99	2.16E-42	3
Isg15	2.24E-28	########	0.592	0.296	3.75E-24	3
Rras	1.71E-78	########	0.712	0.298	2.86E-74	3
Ablim3	0	########	0.386	0.003	0	3
Efnb2	1.30E-215	########	0.494	0.052	2.18E-211	3
Clu	1.28E-202	########	0.392	0.033	2.15E-198	3
Ldb2	0	########	0.627	0.019	0	3
Tubb4b	9.72E-36	########	0.816	0.573	1.63E-31	3
Tpm4	1.63E-48	########	0.88	0.667	2.73E-44	3
Pcdh1	0	########	0.579	0.017	0	3
Dab2ip	1.79E-185	########	0.712	0.138	2.99E-181	3
Pxdn	1.30E-236	########	0.611	0.076	2.18E-232	3
She	0	########	0.617	0.004	0	3
Nrp1	7.02E-31	########	0.693	0.397	1.17E-26	3
Tspan9	0	########	0.617	0.057	0	3
Fscn1	1.04E-170	########	0.566	0.088	1.74E-166	3
Adcy4	0	########	0.62	0.011	0	3
Flt4	0	########	0.316	0.002	0	3
Pls3	5.80E-139	########	0.617	0.127	9.71E-135	3
Sptan1	1.98E-54	########	0.813	0.481	3.31E-50	3
Cyb5a	3.99E-60	########	0.861	0.56	6.68E-56	3
Jund	1.47E-21	########	0.94	0.893	2.46E-17	3
Robo4	0	########	0.589	0.001	0	3
Jup	5.12E-136	########	0.538	0.098	8.56E-132	3

Nckap1	2.64E-190	########	0.658	0.111	4.41E-186	3
Pdgfb	0	########	0.573	0.037	0	3
Tnfaip1	3.61E-112	########	0.665	0.197	6.05E-108	3
Edn1	0	########	0.392	0.007	0	3
Ppp1r15a	4.12E-39	########	0.823	0.489	6.89E-35	3
Ypel2	1.74E-264	########	0.604	0.065	2.91E-260	3
Wbp5	1.39E-62	########	0.801	0.413	2.33E-58	3
Spns2	0	########	0.62	0.013	0	3
Klf9	4.73E-76	########	0.693	0.241	7.91E-72	3
Smco4	1.27E-114	########	0.617	0.156	2.12E-110	3
Clic5	0	########	0.332	0.004	0	3
Rgcc	6.03E-08	########	0.547	0.418	########	3
Grasp	7.06E-231	########	0.639	0.085	1.18E-226	3
Ifi27	1.28E-54	########	0.801	0.474	2.14E-50	3
Pkp4	5.21E-207	########	0.636	0.097	8.71E-203	3
Dynll1	2.59E-52	########	0.93	0.842	4.33E-48	3
2900026A0	0	########	0.601	0.046	0	3
Cpne8	1.12E-215	########	0.639	0.094	1.87E-211	3
Rab11a	7.07E-48	########	0.858	0.664	1.18E-43	3
Map1b	1.71E-179	########	0.598	0.091	2.86E-175	3
Ybx3	5.56E-56	########	0.794	0.414	9.30E-52	3
Myl12a	3.42E-52	########	0.927	0.84	5.73E-48	3
Fryl	2.03E-44	########	0.668	0.32	3.39E-40	3
Gja4	1.96E-163	########	0.218	0.011	3.28E-159	3
Pvrl2	7.26E-256	########	0.56	0.057	1.21E-251	3
Adam15	8.69E-43	########	0.725	0.43	1.45E-38	3
Nfia	2.08E-121	########	0.658	0.158	3.48E-117	3
Bok	3.95E-183	########	0.554	0.077	6.61E-179	3
Bmpr2	4.88E-76	########	0.744	0.31	8.16E-72	3
Osmr	1.49E-156	########	0.557	0.09	2.50E-152	3
Mxd4	1.92E-42	########	0.759	0.424	3.21E-38	3
Tmem176a	8.02E-74	########	0.706	0.257	1.34E-69	3
Lrrc32	8.33E-226	########	0.551	0.061	1.39E-221	3
Mkl2	1.18E-186	########	0.617	0.102	1.97E-182	3
Ubc	2.52E-43	########	0.984	0.95	4.21E-39	3
Cdc42bpa	9.13E-185	########	0.592	0.091	1.53E-180	3
Prss23	1.09E-131	########	0.475	0.075	1.83E-127	3
mt-Cytb	4.23E-36	########	0.956	0.965	7.08E-32	3
Bcr	3.41E-114	########	0.551	0.126	5.71E-110	3
Pdlim4	4.67E-76	########	0.623	0.213	7.81E-72	3
Rps2	7.99E-29	########	0.997	0.928	1.34E-24	3
Npr1	0	########	0.566	0.017	0	3
Erh	2.70E-40	########	0.905	0.71	4.52E-36	3
mt-Nd1	3.63E-24	########	0.953	0.905	6.08E-20	3
Ccnd1	2.47E-62	########	0.503	0.157	4.12E-58	3
Tgfbr3	1.01E-108	########	0.557	0.119	1.68E-104	3
Nrp2	4.96E-77	########	0.693	0.248	8.30E-73	3
Car4	8.76E-298	########	0.358	0.015	1.46E-293	3
Tanc1	3.80E-195	########	0.595	0.088	6.36E-191	3
Trp53i11	8.55E-154	########	0.478	0.066	1.43E-149	3
Pkig	7.24E-59	########	0.775	0.425	1.21E-54	3
Selm	1.32E-103	########	0.747	0.229	2.22E-99	3

Cda	1.19E-266	########	0.513	0.044	1.99E-262	3
Mall	0	########	0.56	0.009	0	3
Arid5b	2.00E-47	########	0.684	0.346	3.35E-43	3
Dpysl3	5.39E-141	########	0.551	0.094	9.02E-137	3
Hmgb1	2.22E-44	########	0.968	0.878	3.71E-40	3
Slc43a3	1.47E-134	########	0.633	0.14	2.46E-130	3
Ndufc2	8.32E-50	########	0.886	0.672	1.39E-45	3
Adamts4	2.58E-231	########	0.497	0.048	4.32E-227	3
Ywhaq	4.93E-57	########	0.886	0.626	8.26E-53	3
Amotl1	3.93E-173	########	0.544	0.082	6.57E-169	3
Bnip2	4.99E-50	########	0.87	0.647	8.34E-46	3
Atp8b1	3.44E-210	########	0.522	0.062	5.75E-206	3
Efna1	0	########	0.557	0.028	0	3
Tmem2	2.68E-61	########	0.598	0.239	4.49E-57	3
Arhgef7	3.23E-91	########	0.646	0.212	5.41E-87	3
Pgf	3.26E-35	########	0.149	0.027	5.45E-31	3
Myo1b	3.74E-137	########	0.554	0.106	6.26E-133	3
Cltb	1.07E-68	########	0.706	0.299	1.79E-64	3
Plxna2	0	########	0.519	0.026	0	3
Thbd	1.25E-77	########	0.532	0.15	2.09E-73	3
Zfand5	1.11E-32	########	0.892	0.727	1.85E-28	3
Notch1	3.19E-59	########	0.712	0.335	5.33E-55	3
Junb	4.50E-16	########	0.911	0.852	7.54E-12	3
Fam65a	1.20E-160	########	0.611	0.115	2.01E-156	3
Nfe2l1	1.83E-50	########	0.759	0.411	3.06E-46	3
Spry1	6.59E-145	########	0.37	0.043	1.10E-140	3
Hyal2	2.19E-155	########	0.566	0.102	3.67E-151	3
Smagp	1.04E-114	########	0.614	0.144	1.74E-110	3
Abcb1a	2.70E-292	########	0.408	0.022	4.52E-288	3
Nes	2.66E-214	########	0.38	0.028	4.45E-210	3
Arl4a	8.75E-76	########	0.693	0.25	1.46E-71	3
Ralb	4.01E-44	########	0.756	0.521	6.71E-40	3
Upp1	2.05E-88	########	0.592	0.159	3.43E-84	3
Lpl	3.03E-16	########	0.335	0.167	5.06E-12	3
Avpi1	3.30E-57	########	0.617	0.243	5.53E-53	3
Spint2	3.66E-85	########	0.554	0.148	6.12E-81	3
Laptm4a	5.95E-40	########	0.886	0.635	9.96E-36	3
Arhgef15	0	########	0.535	0.002	0	3
2-Mar	9.20E-40	########	0.772	0.511	1.54E-35	3
Rbp7	0	########	0.253	0.004	0	3
Dnaja1	3.22E-46	########	0.934	0.846	5.39E-42	3
Prkch	2.68E-116	########	0.642	0.152	4.48E-112	3
Cxx1a	1.27E-185	########	0.557	0.082	2.12E-181	3
Gimap5	6.57E-211	########	0.636	0.08	1.10E-206	3
Bmp4	0	########	0.396	0.014	0	3
Rnd1	1.16E-106	########	0.538	0.121	1.95E-102	3
Dysf	0	########	0.544	0.015	0	3
Sertad1	2.32E-28	########	0.753	0.469	3.89E-24	3
Lrrc58	9.93E-54	########	0.763	0.4	1.66E-49	3
Tagln2	1.15E-32	########	0.911	0.752	1.92E-28	3
Eln	3.69E-120	########	0.383	0.053	6.17E-116	3
Sema7a	4.35E-219	########	0.367	0.026	7.28E-215	3

Plec	5.10E-30	########	0.797	0.614	8.53E-26	3
Hnrnph1	1.73E-33	########	0.858	0.602	2.89E-29	3
Fus	7.05E-24	########	0.908	0.802	1.18E-19	3
Socs2	5.17E-270	########	0.57	0.055	8.66E-266	3
Zfos1	3.07E-39	########	0.778	0.481	5.14E-35	3
Pcmtd1	1.49E-47	########	0.709	0.344	2.49E-43	3
Fam43a	1.11E-132	########	0.592	0.12	1.86E-128	3
Prdx4	4.53E-48	########	0.715	0.345	7.58E-44	3
Calu	7.95E-58	########	0.753	0.36	1.33E-53	3
Icam1	1.19E-36	########	0.611	0.312	2.00E-32	3
Prmt1	8.98E-37	########	0.725	0.425	1.50E-32	3
Msn	6.46E-36	########	0.946	0.916	1.08E-31	3
Chchd2	1.26E-51	########	0.984	0.961	2.10E-47	3
Ran	3.02E-23	########	0.864	0.712	5.06E-19	3
Ephb4	0	########	0.551	0.032	0	3
Rdx	3.24E-44	########	0.835	0.546	5.43E-40	3
Lmcd1	0	########	0.503	0.033	0	3
Tuba1b	3.35E-19	########	0.807	0.594	5.60E-15	3
Ipo11	1.37E-120	########	0.589	0.136	2.30E-116	3
Nfat5	2.28E-23	########	0.747	0.494	3.81E-19	3
Nxn	2.37E-77	########	0.665	0.242	3.96E-73	3
Lama4	1.22E-133	########	0.516	0.087	2.04E-129	3
Tgm2	1.15E-62	########	0.718	0.275	1.93E-58	3
Hnrnpa1	2.36E-33	########	0.854	0.678	3.95E-29	3
Eef1g	2.06E-33	########	0.924	0.8	3.45E-29	3
Rnf125	2.22E-158	########	0.576	0.097	3.71E-154	3
Ptprm	0	########	0.475	0.024	0	3
Entpd1	2.35E-39	########	0.668	0.352	3.94E-35	3
Ncl	3.79E-28	########	0.921	0.822	6.34E-24	3
Mest	0	########	0.415	0.018	0	3
Rock2	4.46E-38	########	0.797	0.516	7.46E-34	3
Ddit4	3.79E-81	########	0.655	0.203	6.33E-77	3
Ace	4.79E-208	########	0.544	0.063	8.01E-204	3
Fnbp1l	7.62E-137	########	0.614	0.13	1.27E-132	3
Rasgrp3	0	########	0.497	0.027	0	3
Dll4	0	########	0.396	0.002	0	3
N4bp3	0	########	0.506	0.033	0	3
Dnajb9	1.63E-34	########	0.649	0.328	2.72E-30	3
Dnm3	7.27E-271	########	0.453	0.032	1.22E-266	3
Ccdc85b	8.43E-90	########	0.627	0.191	1.41E-85	3
Itga1	2.47E-101	########	0.453	0.088	4.13E-97	3
Lmo7	0	########	0.487	0.026	0	3
Cd59a	5.39E-281	########	0.506	0.039	9.01E-277	3
Lamc1	8.83E-106	########	0.639	0.164	1.48E-101	3
Rexo2	3.27E-35	########	0.775	0.501	5.47E-31	3
Gnb1	8.48E-41	########	0.88	0.761	1.42E-36	3
Ets1	1.13E-70	########	0.854	0.371	1.89E-66	3
Mgat4b	1.18E-96	########	0.649	0.205	1.98E-92	3
Tmcc3	3.35E-110	########	0.459	0.085	5.60E-106	3
Tmem47	5.44E-285	########	0.519	0.041	9.10E-281	3
Unc45b	0	########	0.443	0.006	0	3
Il6	7.07E-84	########	0.437	0.092	1.18E-79	3

Rps6ka3	1.62E-51	########	0.737	0.39	2.72E-47	3
Sh3bp5	2.30E-39	########	0.712	0.367	3.85E-35	3
Parvb	8.94E-146	########	0.592	0.111	1.50E-141	3
Abcc9	9.08E-46	########	0.158	0.024	1.52E-41	3
Tspan6	7.15E-181	########	0.535	0.075	1.20E-176	3
Lima1	1.03E-58	########	0.652	0.253	1.73E-54	3
Map4	3.12E-48	########	0.722	0.375	5.23E-44	3
Gna11	1.16E-94	########	0.636	0.187	1.94E-90	3
Pla2g16	2.88E-83	########	0.649	0.198	4.83E-79	3
Epn2	1.64E-127	########	0.459	0.076	2.74E-123	3
Imp3	1.95E-44	########	0.788	0.488	3.26E-40	3
Plscr2	0	########	0.443	0.021	0	3
Eef2	5.86E-42	########	0.953	0.891	9.81E-38	3
Tspan4	2.68E-69	########	0.703	0.256	4.49E-65	3
Etl4	1.02E-291	########	0.43	0.025	1.70E-287	3
Meox1	0	########	0.449	0.009	0	3
Asap2	6.99E-198	########	0.551	0.073	1.17E-193	3
2700094K1	2.95E-17	########	0.665	0.455	4.94E-13	3
Rab13	7.13E-102	########	0.595	0.161	1.19E-97	3
Cdkn1c	3.69E-65	########	0.301	0.059	6.17E-61	3
Fli1	3.72E-37	########	0.785	0.467	6.23E-33	3
Atf4	1.05E-30	########	0.867	0.641	1.76E-26	3
15-Sep	2.21E-37	########	0.908	0.774	3.69E-33	3
Nop58	5.22E-44	########	0.722	0.354	8.73E-40	3
Tspan12	0	########	0.509	0.021	0	3
Hdac7	3.06E-111	########	0.627	0.157	5.12E-107	3
Rasal2	5.25E-184	########	0.535	0.074	8.78E-180	3
Dusp6	5.93E-54	########	0.623	0.239	9.92E-50	3
Stc1	4.57E-290	########	0.342	0.014	7.64E-286	3
Trim47	4.84E-105	########	0.544	0.128	8.11E-101	3
Npm1	4.08E-28	########	0.943	0.812	6.82E-24	3
Tulp4	7.34E-40	########	0.668	0.331	1.23E-35	3
Atox1	7.95E-26	########	0.93	0.858	1.33E-21	3
Nck1	1.36E-43	########	0.741	0.416	2.28E-39	3
Nav1	3.35E-56	########	0.623	0.245	5.61E-52	3
Ppp1r14b	1.76E-23	########	0.766	0.553	2.95E-19	3
Ccdc85a	0	########	0.272	0.001	0	3
Serpine1	3.94E-72	########	0.437	0.106	6.58E-68	3
Rab12	5.98E-74	########	0.693	0.263	1.00E-69	3
Magi1	0	########	0.478	0.024	0	3
Git1	1.17E-97	########	0.566	0.151	1.96E-93	3
Adarb1	3.94E-178	########	0.544	0.077	6.60E-174	3
Chp2	0	########	0.358	0.013	0	3
Synpo	2.41E-202	########	0.506	0.059	4.03E-198	3
Msrb3	6.48E-212	########	0.513	0.057	1.08E-207	3
Rgs16	2.40E-269	########	0.405	0.024	4.02E-265	3
Tmod3	4.15E-29	########	0.826	0.657	6.94E-25	3
Shank3	0	########	0.497	0.004	0	3
Bsg	2.73E-30	########	0.87	0.701	4.57E-26	3
mt-Co2	2.21E-22	########	0.965	0.99	3.69E-18	3
Hoxa7	0	########	0.57	0.044	0	3
Smim10l1	3.83E-59	########	0.684	0.295	6.40E-55	3

Eif2s2	1.93E-21	########	0.896	0.763	3.23E-17	3
Cfdp1	9.06E-37	########	0.747	0.481	1.52E-32	3
10-Sep	1.97E-134	########	0.56	0.112	3.29E-130	3
Pea15a	3.47E-73	########	0.69	0.25	5.81E-69	3
Uqcrb	2.60E-31	########	0.902	0.759	4.35E-27	3
Thrsp	0	########	0.218	0.001	0	3
Cdc42bpb	1.67E-116	########	0.576	0.133	2.79E-112	3
Mylip	3.95E-29	########	0.592	0.303	6.61E-25	3
Psmd7	1.18E-40	########	0.807	0.495	1.97E-36	3
Caskin2	0	########	0.522	0.032	0	3
mt-Nd2	2.18E-16	########	0.953	0.89	3.65E-12	3
Lamb1	1.65E-157	########	0.544	0.083	2.76E-153	3
Mbnl2	3.63E-29	########	0.835	0.616	6.07E-25	3
Pde2a	5.87E-49	########	0.598	0.241	9.81E-45	3
Topors	1.39E-33	########	0.687	0.375	2.32E-29	3
Bcl6b	0	########	0.478	0.004	0	3
Sema3g	8.38E-252	########	0.199	0.003	1.40E-247	3
Lrrc8a	2.23E-101	########	0.576	0.147	3.74E-97	3
Tyms	5.18E-55	########	0.551	0.197	8.67E-51	3
Sfr1	3.76E-34	########	0.873	0.699	6.30E-30	3
Clec1a	0	########	0.449	0.02	0	3
Apln	8.51E-62	########	0.193	0.027	1.42E-57	3
Galnt15	0	########	0.373	0.006	0	3
Cnbp	5.58E-32	########	0.896	0.703	9.34E-28	3
Calm1	4.95E-34	########	0.972	0.98	8.28E-30	3
Ushbp1	0	########	0.43	0.001	0	3
Klf7	1.94E-29	########	0.756	0.511	3.25E-25	3
Fry	6.92E-42	########	0.582	0.25	1.16E-37	3
mt-Nd3	6.88E-10	########	0.927	0.843	1.15E-05	3
Slc38a2	3.04E-28	########	0.873	0.662	5.09E-24	3
Hmgn1	1.04E-44	########	0.797	0.401	1.74E-40	3
Gnai2	2.72E-39	########	0.968	0.949	4.55E-35	3
Hmgn3	7.75E-128	########	0.627	0.138	1.30E-123	3
Zeb1	7.51E-79	########	0.655	0.212	1.26E-74	3
Prcp	3.15E-29	########	0.668	0.407	5.27E-25	3
Gnb4	3.68E-132	########	0.484	0.079	6.15E-128	3
Ktn1	1.56E-50	########	0.734	0.348	2.61E-46	3
Mustn1	1.79E-192	########	0.449	0.046	2.99E-188	3
Cmtm8	0	########	0.468	0.022	0	3
Sema3a	1.71E-54	########	0.133	0.014	2.85E-50	3
Klf4	3.26E-21	########	0.797	0.501	5.46E-17	3
Cdkn1a	6.52E-21	########	0.797	0.479	1.09E-16	3
Tsen34	1.82E-51	########	0.693	0.336	3.04E-47	3
Serinc3	1.77E-19	########	0.953	0.824	2.97E-15	3
Myo1c	2.94E-53	########	0.687	0.322	4.92E-49	3
Ppp1r11	2.45E-31	########	0.75	0.524	4.10E-27	3
Atp5j	2.54E-33	########	0.934	0.869	4.25E-29	3
Ccser2	3.17E-74	########	0.614	0.202	5.31E-70	3
Hmgn5	5.83E-37	########	0.642	0.326	9.75E-33	3
Nrarp	2.84E-230	########	0.468	0.041	4.75E-226	3
Ptprg	1.01E-162	########	0.421	0.05	1.69E-158	3
Eif4a1	1.02E-27	########	0.949	0.831	1.71E-23	3

Adipor2	3.71E-32	########	0.725	0.436	6.21E-28	3
2210013O2	1.57E-70	########	0.633	0.232	2.62E-66	3
Lpar6	8.45E-86	########	0.509	0.125	1.41E-81	3
Bace2	0	########	0.494	0.032	0	3
Ptpn14	4.78E-203	########	0.418	0.038	7.99E-199	3
Sntb2	1.65E-49	########	0.633	0.286	2.77E-45	3
Endod1	3.34E-79	########	0.509	0.14	5.59E-75	3
Ttc28	6.95E-150	########	0.566	0.096	1.16E-145	3
Ube2s	6.13E-22	########	0.835	0.689	1.03E-17	3
Cxcl10	1.79E-08	########	0.184	0.089	########	3
Cttn	4.05E-138	########	0.582	0.109	6.78E-134	3
Eva1a	0	########	0.491	0.012	0	3
Srsf2	8.07E-29	########	0.915	0.742	1.35E-24	3
Galnt1	3.66E-23	########	0.725	0.472	6.12E-19	3
Trim24	5.79E-67	########	0.557	0.181	9.69E-63	3
Itpr1	7.49E-60	########	0.585	0.201	1.25E-55	3
Acvrl1	5.48E-64	########	0.633	0.228	9.18E-60	3
Gem	6.62E-69	########	0.491	0.131	1.11E-64	3
Atp5b	5.63E-30	########	0.924	0.841	9.43E-26	3
Scd2	2.29E-58	########	0.668	0.258	3.83E-54	3
Cxcl9	6.53E-123	########	0.158	0.007	1.09E-118	3
Arap3	1.32E-110	########	0.592	0.134	2.21E-106	3
Parva	4.24E-133	########	0.573	0.106	7.10E-129	3
Anxa7	2.88E-32	########	0.725	0.465	4.82E-28	3
Tspan15	0	########	0.408	0.012	0	3
Bik	0	########	0.44	0.006	0	3
Krtcap2	4.31E-25	########	0.858	0.663	7.22E-21	3
Filip1	0	########	0.43	0.021	0	3
Rps17	5.07E-25	########	0.968	0.889	8.48E-21	3
Arhgap5	4.63E-68	########	0.589	0.187	7.74E-64	3
Trim37	6.32E-115	########	0.563	0.127	1.06E-110	3
Tcf7l2	4.75E-82	########	0.63	0.187	7.94E-78	3
Pkn2	7.10E-31	########	0.782	0.515	1.19E-26	3
Nhp2l1	2.48E-28	########	0.813	0.589	4.14E-24	3
Mcf2l	1.40E-93	########	0.269	0.035	2.34E-89	3
Eva1b	3.00E-52	########	0.633	0.251	5.02E-48	3
Hip1	6.57E-49	########	0.642	0.271	1.10E-44	3
Hint1	3.18E-20	########	0.934	0.784	5.32E-16	3
Tns2	7.84E-205	########	0.484	0.052	1.31E-200	3
Plscr3	9.20E-64	########	0.62	0.234	1.54E-59	3
Phactr2	5.27E-53	########	0.63	0.265	8.82E-49	3
Abhd17a	1.32E-27	########	0.709	0.501	2.21E-23	3
Slc12a2	1.22E-136	########	0.5	0.083	2.05E-132	3
4921524J1	4.71E-47	########	0.633	0.291	7.88E-43	3
Irf1	1.22E-10	########	0.646	0.456	2.04E-06	3
Hnrnpa0	1.88E-30	########	0.902	0.801	3.15E-26	3
Snhg6	1.33E-32	########	0.642	0.335	2.22E-28	3
Dock1	5.13E-60	########	0.541	0.186	8.58E-56	3
Sorbs2	0	########	0.456	0.011	0	3
Insr	3.37E-26	########	0.437	0.201	5.63E-22	3
Nasp	2.02E-26	########	0.756	0.472	3.38E-22	3
Clic1	7.92E-24	########	0.927	0.94	1.33E-19	3

Cd2ap	1.12E-36	########	0.737	0.391	1.87E-32	3
Rps3	3.05E-22	########	0.984	0.967	5.11E-18	3
Ranbp1	1.34E-22	########	0.804	0.581	2.24E-18	3
Hspa5	1.21E-22	########	0.943	0.833	2.03E-18	3
Dnajb1	3.43E-31	########	0.699	0.397	5.74E-27	3
Hras	4.12E-33	########	0.693	0.42	6.89E-29	3
Mef2c	1.15E-62	########	0.813	0.374	1.92E-58	3
Pgm5	0	########	0.335	0.007	0	3
Slc29a1	2.28E-59	########	0.547	0.19	3.81E-55	3
Serbp1	3.07E-27	########	0.937	0.802	5.14E-23	3
Phlda3	2.09E-85	########	0.475	0.108	3.49E-81	3
Cpe	8.70E-58	########	0.259	0.049	1.46E-53	3
Rai14	1.01E-105	########	0.528	0.114	1.69E-101	3
Ppp1r13b	3.69E-153	########	0.528	0.081	6.17E-149	3
Gm42418	1.67E-09	########	1	1	2.79E-05	3
Sash1	4.27E-48	########	0.671	0.278	7.15E-44	3
Cfl2	8.04E-69	########	0.652	0.236	1.35E-64	3
Dennd5b	1.22E-183	########	0.497	0.061	2.04E-179	3
Rpl4	1.24E-24	########	0.949	0.883	2.08E-20	3
Rwdd1	6.33E-25	########	0.797	0.603	1.06E-20	3
Fam198b	9.75E-138	########	0.462	0.07	1.63E-133	3
Ubb	1.32E-28	########	0.978	0.984	2.21E-24	3
Fam174b	2.47E-147	########	0.291	0.025	4.14E-143	3
Hmg20b	8.20E-35	########	0.709	0.419	1.37E-30	3
Rps6	4.16E-26	########	0.984	0.937	6.96E-22	3
Tubb2a	3.74E-32	########	0.642	0.332	6.26E-28	3
Pear1	7.39E-303	########	0.525	0.038	1.24E-298	3
Fam101b	6.12E-19	########	0.582	0.325	1.02E-14	3
Pros1	8.82E-58	########	0.589	0.205	1.48E-53	3
Ptk2	2.88E-137	########	0.491	0.081	4.82E-133	3
Ndufa12	4.24E-27	########	0.794	0.561	7.10E-23	3
Tmem158	5.14E-108	########	0.37	0.058	8.60E-104	3
Fez2	1.69E-54	########	0.551	0.201	2.83E-50	3
Ppm1f	5.84E-186	########	0.506	0.064	9.77E-182	3
7-Sep	9.29E-27	########	0.861	0.756	1.55E-22	3
Trp53	4.41E-37	########	0.722	0.428	7.37E-33	3
Rpl3	3.01E-22	########	0.987	0.864	5.03E-18	3
Gpr146	4.27E-50	########	0.576	0.221	7.14E-46	3
Xbp1	4.64E-20	########	0.778	0.55	7.76E-16	3
Prox1	3.68E-142	########	0.155	0.005	6.16E-138	3
Fblim1	3.32E-97	########	0.509	0.113	5.55E-93	3
Arhgef5	4.06E-295	########	0.481	0.032	6.80E-291	3
Bambi	1.30E-88	########	0.443	0.094	2.17E-84	3
Tmem59	1.70E-23	########	0.867	0.761	2.85E-19	3
Ccm2l	0	########	0.446	0.006	0	3
Anxa3	1.31E-24	########	0.715	0.452	2.19E-20	3
Hspa8	6.18E-25	########	0.984	0.943	1.03E-20	3
Slc50a1	1.84E-41	########	0.709	0.362	3.08E-37	3
Mpzl1	1.19E-144	########	0.491	0.079	1.98E-140	3
Galnt18	2.18E-292	########	0.408	0.022	3.64E-288	3
Sh3glb1	3.44E-25	########	0.93	0.838	5.76E-21	3
Tmem204	0	########	0.449	0.01	0	3

2-Sep	3.92E-34	########	0.829	0.547	6.56E-30	3
Wasf2	2.11E-22	########	0.848	0.716	3.53E-18	3
Angpt2	1.52E-126	########	0.335	0.039	2.55E-122	3
Cdk4	9.19E-29	########	0.677	0.403	1.54E-24	3
Vcam1	1.96E-26	########	0.329	0.124	3.29E-22	3
Cct7	1.88E-24	########	0.804	0.616	3.14E-20	3
Gls	9.58E-22	########	0.864	0.633	1.60E-17	3
Cxx1b	4.96E-149	########	0.491	0.075	8.29E-145	3
Grb10	5.15E-153	########	0.557	0.086	8.62E-149	3
Piezo1	7.49E-53	########	0.642	0.275	1.25E-48	3
Lamb2	2.57E-113	########	0.475	0.086	4.30E-109	3
H1f0	8.61E-49	########	0.604	0.242	1.44E-44	3
Wwc2	4.15E-84	########	0.522	0.14	6.94E-80	3
Hexim1	7.10E-20	########	0.69	0.415	1.19E-15	3
Arf4	2.32E-26	########	0.826	0.631	3.87E-22	3
Impdh2	5.01E-32	########	0.715	0.404	8.38E-28	3
Morf4l2	7.84E-34	########	0.804	0.509	1.31E-29	3
Eef1a1	2.25E-27	########	0.997	0.987	3.77E-23	3
Fmo1	2.89E-159	########	0.288	0.022	4.84E-155	3
Mfng	6.81E-107	########	0.481	0.092	1.14E-102	3
Psmc4	3.36E-24	########	0.737	0.537	5.63E-20	3
Wasl	1.12E-58	########	0.614	0.228	1.87E-54	3
Tpd52l2	9.63E-30	########	0.674	0.42	1.61E-25	3
2610305D1	0	########	0.411	0.01	0	3
Dad1	1.47E-21	########	0.88	0.733	2.45E-17	3
Itpkb	4.79E-27	########	0.661	0.358	8.02E-23	3
Itgb4	1.67E-153	########	0.326	0.029	2.80E-149	3
Map4k3	1.09E-70	########	0.408	0.097	1.83E-66	3
Kctd10	2.02E-41	########	0.639	0.308	3.37E-37	3
Pa2g4	1.02E-29	########	0.725	0.426	1.71E-25	3
Sbds	2.99E-26	########	0.722	0.438	5.01E-22	3
Pdlim7	6.04E-43	########	0.627	0.284	1.01E-38	3
Foxo1	1.24E-64	########	0.582	0.189	2.08E-60	3
mt-Nd4	5.30E-13	########	0.956	0.961	8.86E-09	3
Ccny	3.40E-45	########	0.639	0.294	5.69E-41	3
Cetn3	3.67E-26	########	0.759	0.51	6.14E-22	3
2010111I01	8.04E-49	########	0.614	0.254	1.35E-44	3
Mrpl17	3.01E-24	########	0.69	0.458	5.04E-20	3
Kit	4.46E-70	########	0.272	0.045	7.47E-66	3
Myc	4.00E-66	########	0.5	0.143	6.69E-62	3
Slc25a3	2.87E-26	########	0.946	0.866	4.80E-22	3
Socs3	2.13E-12	########	0.744	0.539	3.57E-08	3
Atp5g2	8.00E-21	########	0.946	0.812	1.34E-16	3
Eif3i	1.54E-22	########	0.835	0.689	2.58E-18	3
Slco3a1	8.15E-40	########	0.484	0.19	1.36E-35	3
Rell1	2.31E-28	########	0.617	0.348	3.86E-24	3
Atrx	2.66E-21	########	0.842	0.696	4.45E-17	3
Set	1.56E-25	########	0.886	0.709	2.60E-21	3
Gimap1	2.22E-118	########	0.734	0.156	3.71E-114	3
Rfk	1.47E-43	########	0.595	0.257	2.45E-39	3
Tceal8	1.29E-53	########	0.658	0.259	2.16E-49	3
Snrpd1	1.96E-20	########	0.778	0.566	3.28E-16	3

Auts2	1.58E-49	########	0.38	0.107	2.65E-45	3
Lama5	0	########	0.453	0.019	0	3
Smc1a	2.94E-25	########	0.807	0.598	4.92E-21	3
Psma3	8.78E-25	########	0.886	0.772	1.47E-20	3
Gm10073	1.48E-21	########	0.763	0.556	2.48E-17	3
Adam10	3.06E-25	########	0.753	0.519	5.13E-21	3
Adh1	3.61E-64	########	0.165	0.018	6.04E-60	3
Sod1	1.43E-33	########	0.797	0.507	2.39E-29	3
Park7	8.67E-20	########	0.864	0.726	1.45E-15	3
Tmem173	2.77E-36	########	0.642	0.314	4.64E-32	3
Eny2	7.87E-27	########	0.747	0.484	1.32E-22	3
Sertad2	8.18E-35	########	0.636	0.307	1.37E-30	3
Tspan2	1.51E-91	########	0.418	0.082	2.53E-87	3
Creb3l2	7.36E-91	########	0.525	0.13	1.23E-86	3
Gstt2	4.40E-113	########	0.453	0.08	7.36E-109	3
Chst7	1.08E-264	########	0.434	0.029	1.81E-260	3
Midn	8.29E-23	########	0.734	0.504	1.39E-18	3
Tmed9	8.10E-23	########	0.851	0.666	1.36E-18	3
Efnb1	4.27E-109	########	0.405	0.067	7.15E-105	3
Pnkd	7.30E-40	########	0.696	0.359	1.22E-35	3
Nova2	0	########	0.421	0.001	0	3
Tmem140	6.81E-47	########	0.411	0.128	1.14E-42	3
Hoxb7	0	########	0.443	0.009	0	3
Mylk	2.66E-251	########	0.484	0.039	4.44E-247	3
Ndufab1	4.21E-23	########	0.788	0.589	7.05E-19	3
Ctnnd1	4.07E-65	########	0.585	0.2	6.81E-61	3
8430408G2	0	########	0.218	0.001	0	3
Smad5	3.62E-73	########	0.528	0.15	6.05E-69	3
Limch1	3.66E-272	########	0.354	0.017	6.12E-268	3
Pkn3	0	########	0.475	0.023	0	3
Tcf12	4.68E-38	########	0.671	0.331	7.83E-34	3
Stox2	6.84E-173	########	0.405	0.042	1.14E-168	3
Tnfsf10	1.85E-209	########	0.361	0.025	3.09E-205	3
Ehd3	2.43E-137	########	0.32	0.032	4.06E-133	3
Kif5b	3.12E-19	########	0.835	0.712	5.22E-15	3
Fkbp9	1.45E-100	########	0.576	0.128	2.43E-96	3
Mapk3	3.89E-24	########	0.775	0.543	6.50E-20	3
Dach1	5.69E-48	########	0.367	0.106	9.52E-44	3
Lysmd2	1.28E-154	########	0.5	0.074	2.15E-150	3
Ndufs5	1.94E-24	########	0.858	0.729	3.25E-20	3
Cbx1	1.14E-26	########	0.725	0.441	1.90E-22	3
Mmp14	1.79E-43	########	0.611	0.236	3.00E-39	3
Snrpb	5.58E-20	########	0.87	0.738	9.34E-16	3
Tmem109	1.18E-42	########	0.627	0.282	1.98E-38	3
Cct3	1.18E-25	########	0.759	0.5	1.97E-21	3
Atp2b4	5.68E-57	########	0.551	0.182	9.51E-53	3
Stap2	0	########	0.462	0.021	0	3
Tfpi	7.87E-121	########	0.475	0.084	1.32E-116	3
Ddah1	7.12E-120	########	0.364	0.048	1.19E-115	3
Fmnl3	4.88E-74	########	0.503	0.138	8.17E-70	3
Gngt2	2.55E-46	########	0.801	0.42	4.27E-42	3
Ctnnal1	1.36E-169	########	0.342	0.03	2.28E-165	3

Fundc2	9.48E-28	########	0.744	0.487	1.59E-23	3
Rasd1	3.83E-125	########	0.335	0.04	6.41E-121	3
Prkar1a	1.57E-26	########	0.87	0.769	2.63E-22	3
Top1	9.21E-20	########	0.883	0.806	1.54E-15	3
Ndufa4	4.39E-14	########	0.915	0.829	7.34E-10	3
Stmn1	6.28E-21	########	0.589	0.315	1.05E-16	3
Yap1	1.51E-125	########	0.484	0.083	2.53E-121	3
Kcnj8	6.21E-48	########	0.155	0.022	1.04E-43	3
Fzd6	6.81E-306	########	0.405	0.02	1.14E-301	3
Cebpg	1.74E-32	########	0.718	0.42	2.91E-28	3
Itga5	2.40E-39	########	0.636	0.291	4.02E-35	3
Mxra7	6.14E-126	########	0.528	0.093	1.03E-121	3
Zfp326	1.21E-37	########	0.658	0.334	2.02E-33	3
Card10	3.33E-178	########	0.427	0.046	5.57E-174	3
Maged1	3.01E-97	########	0.604	0.139	5.04E-93	3
Bag3	6.75E-52	########	0.608	0.234	1.13E-47	3
Tax1bp3	6.91E-39	########	0.633	0.315	1.16E-34	3
Ssb	2.27E-22	########	0.87	0.688	3.79E-18	3
Anapc16	4.75E-28	########	0.744	0.48	7.95E-24	3
Prdx2	4.78E-21	########	0.861	0.674	7.99E-17	3
Pgrmc1	3.46E-42	########	0.684	0.316	5.79E-38	3
Sumo2	2.17E-22	########	0.88	0.78	3.64E-18	3
Csnk1a1	6.69E-23	########	0.896	0.777	1.12E-18	3
Cenpb	4.52E-26	########	0.728	0.456	7.57E-22	3
Knop1	9.80E-34	########	0.636	0.323	1.64E-29	3
Gpr182	0	########	0.358	0.001	0	3
Dtymk	1.03E-22	########	0.649	0.37	1.73E-18	3
Myo6	9.60E-98	########	0.386	0.067	1.61E-93	3
Cdr2l	3.66E-141	########	0.453	0.066	6.12E-137	3
Sypl	4.65E-31	########	0.81	0.519	7.79E-27	3
Slc30a4	1.14E-105	########	0.411	0.071	1.91E-101	3
Sugt1	1.11E-22	########	0.778	0.63	1.86E-18	3
Banf1	4.68E-19	########	0.75	0.547	7.83E-15	3
Tceb1	5.53E-19	########	0.845	0.706	9.26E-15	3
S100a10	8.82E-21	########	0.949	0.703	1.48E-16	3
Brd2	2.50E-16	########	0.823	0.666	4.19E-12	3
Psmb6	3.58E-18	########	0.829	0.709	6.00E-14	3
Cbx5	1.23E-39	########	0.608	0.265	2.06E-35	3
Tbrg1	7.02E-29	########	0.759	0.458	1.17E-24	3
Mrfap1	2.77E-22	########	0.864	0.697	4.64E-18	3
Bzw2	1.85E-44	########	0.598	0.257	3.10E-40	3
Enah	6.20E-49	########	0.373	0.103	1.04E-44	3
Srsf7	5.32E-24	########	0.763	0.491	8.91E-20	3
Smtn	1.60E-74	########	0.418	0.1	2.67E-70	3
Rps27l	2.56E-13	########	0.889	0.739	4.28E-09	3
Lmo2	2.06E-14	########	0.592	0.376	3.45E-10	3
Gbp7	9.58E-29	########	0.554	0.255	1.60E-24	3
Tmem238	1.44E-60	########	0.503	0.159	2.41E-56	3
Plekhg1	2.06E-105	########	0.434	0.079	3.44E-101	3
Mat2a	1.49E-21	########	0.832	0.583	2.49E-17	3
Cers4	1.57E-138	########	0.487	0.075	2.62E-134	3
Psmc6	1.02E-22	########	0.782	0.57	1.71E-18	3

Ncoa7	7.71E-34	########	0.544	0.249	1.29E-29	3
Taf1d	7.26E-27	########	0.737	0.456	1.21E-22	3
Plcg1	1.67E-126	########	0.497	0.086	2.79E-122	3
Hsp90aa1	6.54E-20	########	0.934	0.803	1.09E-15	3
Zcchc14	2.25E-134	########	0.443	0.067	3.77E-130	3
Ptp4a3	2.21E-21	########	0.478	0.248	3.70E-17	3
1700020I14	5.49E-25	########	0.785	0.532	9.19E-21	3
Plod1	1.21E-30	########	0.598	0.293	2.02E-26	3
Pfdn1	4.03E-22	########	0.696	0.464	6.75E-18	3
Tspan18	1.61E-293	########	0.408	0.022	2.69E-289	3
Taf7	2.18E-21	########	0.525	0.284	3.65E-17	3
Spop	2.91E-13	########	0.794	0.646	4.87E-09	3
Psmd1	3.14E-22	########	0.69	0.456	5.26E-18	3
2700060E0	1.45E-21	########	0.832	0.682	2.42E-17	3
Cds2	2.70E-45	########	0.655	0.297	4.51E-41	3
Rpl36a	4.22E-19	########	0.978	0.866	7.07E-15	3
Scoc	5.21E-59	########	0.585	0.209	8.72E-55	3
Zfp664	1.65E-58	########	0.509	0.167	2.75E-54	3
Gata2	0	########	0.446	0.012	0	3
Guk1	5.54E-28	########	0.649	0.373	9.27E-24	3
mt-Co3	6.97E-12	########	0.968	0.992	1.17E-07	3
Nhp2	1.07E-12	########	0.646	0.487	1.79E-08	3
Rpl14	2.29E-15	########	0.981	0.885	3.83E-11	3
Cdc42ep1	7.10E-163	########	0.348	0.033	1.19E-158	3
Pik3r3	3.88E-158	########	0.301	0.024	6.49E-154	3
Impad1	3.57E-36	########	0.601	0.282	5.97E-32	3
Macf1	1.65E-18	########	0.851	0.673	2.76E-14	3
Ppib	4.70E-18	########	0.896	0.84	7.87E-14	3
Ggta1	7.00E-40	########	0.576	0.249	1.17E-35	3
Tob1	1.47E-26	########	0.585	0.296	2.46E-22	3
Metap2	2.30E-20	########	0.816	0.632	3.85E-16	3
Aebp1	1.20E-54	########	0.421	0.109	2.00E-50	3
Rps20	9.00E-18	########	0.997	0.935	1.51E-13	3
Srsf3	2.08E-18	########	0.899	0.779	3.48E-14	3
Ttc3	3.78E-49	########	0.576	0.215	6.33E-45	3
Spaca6	4.70E-84	########	0.402	0.082	7.86E-80	3
Nbeal1	8.03E-39	########	0.601	0.265	1.34E-34	3
Eif4g2	5.56E-23	########	0.94	0.862	9.30E-19	3
Bcar1	1.27E-199	########	0.427	0.041	2.12E-195	3
Cox6c	1.70E-17	########	0.962	0.912	2.84E-13	3
Mical2	4.73E-87	########	0.32	0.051	7.91E-83	3
Cct2	2.25E-18	########	0.816	0.674	3.76E-14	3
St3gal6	4.66E-92	########	0.528	0.117	7.80E-88	3
Nol7	4.74E-19	########	0.823	0.659	7.94E-15	3
Swap70	1.93E-51	########	0.627	0.25	3.23E-47	3
Gm12216	1.63E-50	########	0.278	0.061	2.72E-46	3
Hnrnpab	1.96E-19	########	0.845	0.661	3.28E-15	3
Inhbb	6.77E-301	########	0.348	0.014	1.13E-296	3
Pcp4l1	3.14E-105	########	0.256	0.027	5.25E-101	3
Btf3	2.21E-20	########	0.956	0.918	3.70E-16	3
Lxn	1.09E-38	########	0.554	0.248	1.83E-34	3
Scarf1	0	########	0.462	0.025	0	3

Ugcg	1.01E-16	########	0.718	0.472	1.69E-12	3
Dnttip2	4.95E-25	########	0.712	0.457	8.29E-21	3
Aqp7	5.11E-301	########	0.187	0.001	8.55E-297	3
Clca3a1	8.68E-245	########	0.174	0.002	1.45E-240	3
Myof	1.21E-16	########	0.494	0.293	2.02E-12	3
Rapgef5	0	########	0.377	0.014	0	3
Myo1d	5.02E-106	########	0.44	0.079	8.41E-102	3
Rapgef3	0	########	0.386	0.015	0	3
Hspa12b	0	########	0.396	0.009	0	3
Smarca4	1.21E-14	########	0.627	0.421	2.03E-10	3
Ccnyl1	9.78E-72	########	0.446	0.114	1.64E-67	3
Spag9	1.52E-22	########	0.873	0.713	2.55E-18	3
Rps8	2.38E-14	########	0.997	0.964	3.98E-10	3
Tnfrsf10b	1.02E-267	########	0.392	0.023	1.70E-263	3
Paics	1.22E-28	########	0.706	0.388	2.04E-24	3
Svbp	2.22E-27	########	0.706	0.446	3.71E-23	3
Igfbp5	6.51E-10	########	0.193	0.09	1.09E-05	3
Timm17a	2.97E-26	########	0.668	0.4	4.96E-22	3
Hspa1a	2.52E-25	########	0.392	0.163	4.22E-21	3
Kcnq1ot1	6.81E-19	########	0.598	0.336	1.14E-14	3
Lhx6	0	########	0.361	0.001	0	3
Rasgrf2	7.34E-258	########	0.294	0.011	1.23E-253	3
Zfp644	4.14E-27	########	0.715	0.427	6.92E-23	3
Dock6	4.08E-266	########	0.405	0.025	6.83E-262	3
Cyth3	7.10E-39	########	0.503	0.198	1.19E-34	3
Ddx39	6.33E-19	########	0.636	0.399	1.06E-14	3
Lgals9	9.23E-18	########	0.728	0.501	1.54E-13	3
Elk4	2.09E-29	########	0.617	0.316	3.50E-25	3
Eif3e	7.10E-18	########	0.867	0.73	1.19E-13	3
Fkbp10	8.23E-96	########	0.5	0.098	1.38E-91	3
Mphosph8	1.61E-29	########	0.598	0.305	2.70E-25	3
Lnx1	3.67E-59	########	0.18	0.024	6.14E-55	3
Vtn	1.97E-34	########	0.117	0.017	3.30E-30	3
Sh3pxd2a	1.75E-43	########	0.513	0.182	2.93E-39	3
Glrx3	2.37E-21	########	0.769	0.523	3.96E-17	3
Rpl28	6.26E-15	########	0.987	0.963	1.05E-10	3
Tcp1	2.36E-19	########	0.775	0.571	3.94E-15	3
Clstn1	2.97E-84	########	0.446	0.099	4.97E-80	3
Rbfox2	1.69E-98	########	0.478	0.098	2.82E-94	3
Ywhae	2.62E-19	########	0.892	0.781	4.39E-15	3
Bola3	3.75E-25	########	0.636	0.342	6.27E-21	3
Glul	9.83E-08	########	0.652	0.478	########	3
Slc39a10	1.29E-62	########	0.478	0.138	2.16E-58	3
Ercc1	3.56E-31	########	0.443	0.187	5.95E-27	3
Nenf	5.95E-43	########	0.703	0.325	9.96E-39	3
Fam92a	3.26E-79	########	0.582	0.166	5.45E-75	3
Fyttd1	3.24E-34	########	0.62	0.303	5.42E-30	3
Rnf7	3.95E-24	########	0.807	0.621	6.61E-20	3
Ptprk	5.07E-237	########	0.389	0.027	8.49E-233	3
Tacc1	2.15E-17	########	0.759	0.521	3.59E-13	3
Grap	3.42E-73	########	0.462	0.108	5.72E-69	3
Arhgef28	1.98E-227	########	0.297	0.014	3.31E-223	3

Ech1	9.42E-16	########	0.763	0.566	1.58E-11	3
Car8	4.79E-163	########	0.155	0.004	8.01E-159	3
Erf	1.72E-44	########	0.528	0.199	2.87E-40	3
Cyp4b1	2.55E-187	########	0.237	0.011	4.27E-183	3
Mgat4a	1.47E-60	########	0.497	0.148	2.46E-56	3
Rbm3	2.40E-11	########	0.946	0.885	4.01E-07	3
Peak1	2.66E-43	########	0.633	0.264	4.44E-39	3
Tbca	1.43E-17	########	0.854	0.715	2.39E-13	3
Kcna5	0	########	0.231	0.002	0	3
Sema4c	1.97E-132	########	0.373	0.047	3.30E-128	3
Fam181b	0	########	0.269	0.001	0	3
Hspa2	3.32E-58	########	0.411	0.112	5.55E-54	3
Manf	3.14E-16	########	0.782	0.596	5.26E-12	3
Hivep2	1.79E-27	########	0.614	0.31	3.00E-23	3
Nop56	4.29E-24	########	0.668	0.37	7.18E-20	3
Erc1	1.76E-63	########	0.459	0.132	2.95E-59	3
Gm10076	6.19E-11	########	0.918	0.888	1.04E-06	3
Ilf2	2.32E-33	########	0.633	0.311	3.88E-29	3
Rlim	7.62E-19	########	0.661	0.426	1.27E-14	3
Cct5	9.81E-16	########	0.804	0.686	1.64E-11	3
Tes	5.17E-29	########	0.655	0.36	8.65E-25	3
Qk	6.95E-19	########	0.854	0.656	1.16E-14	3
Psmb2	9.52E-15	########	0.816	0.692	1.59E-10	3
Gltscr2	7.61E-19	########	0.867	0.703	1.27E-14	3
Hmgb3	4.53E-56	########	0.497	0.159	7.57E-52	3
Gopc	7.27E-42	########	0.491	0.185	1.22E-37	3
Klhl4	0	########	0.304	0	0	3
Cbfa2t3	6.75E-46	########	0.494	0.165	1.13E-41	3
Rpl24	6.14E-14	########	0.991	0.96	1.03E-09	3
Pdap1	1.38E-19	########	0.807	0.62	2.31E-15	3
Emc10	2.57E-27	########	0.703	0.435	4.31E-23	3
Cenpt	3.42E-89	########	0.418	0.087	5.71E-85	3
Cp	7.47E-25	########	0.18	0.049	1.25E-20	3
2310036O2	6.62E-20	########	0.883	0.77	1.11E-15	3
Pard6g	6.67E-17	########	0.158	0.052	1.12E-12	3
Gm13889	3.11E-114	########	0.285	0.03	5.20E-110	3
Plxna4	2.15E-226	########	0.297	0.014	3.60E-222	3
Nop10	3.06E-16	########	0.778	0.589	5.12E-12	3
G3bp1	2.79E-20	########	0.807	0.571	4.66E-16	3
Sema6d	7.13E-168	########	0.402	0.042	1.19E-163	3
Eef1b2	9.22E-14	########	0.959	0.925	1.54E-09	3
Mapre1	1.51E-16	########	0.813	0.674	2.52E-12	3
Raly	1.95E-17	########	0.782	0.637	3.27E-13	3
Pura	7.95E-18	########	0.763	0.507	1.33E-13	3
Gabarapl1	5.13E-43	########	0.566	0.224	8.58E-39	3
Hrct1	3.45E-279	########	0.326	0.013	5.78E-275	3
1110038F1	3.40E-29	########	0.566	0.279	5.69E-25	3
Olfml2a	4.90E-281	########	0.285	0.009	8.19E-277	3
Myzap	1.49E-284	########	0.323	0.012	2.50E-280	3
Il1r1	6.38E-62	########	0.427	0.107	1.07E-57	3
Psmc5	3.59E-13	########	0.722	0.554	6.00E-09	3
Gkap1	2.22E-50	########	0.405	0.121	3.72E-46	3

Mepce	1.13E-18	########	0.538	0.299	1.90E-14	3
Hnrnpa2b1	1.05E-15	########	0.946	0.898	1.75E-11	3
Pparg	2.26E-118	########	0.313	0.036	3.78E-114	3
Lrp5	8.15E-51	########	0.354	0.095	1.36E-46	3
Rpl31	3.08E-14	########	0.968	0.904	5.15E-10	3
Kif1c	1.29E-41	########	0.513	0.198	2.16E-37	3
Stard4	1.42E-79	########	0.516	0.136	2.38E-75	3
Cct4	3.23E-19	########	0.826	0.63	5.41E-15	3
Pitpnc1	1.80E-19	########	0.585	0.328	3.02E-15	3
Mlf2	4.68E-21	########	0.709	0.468	7.83E-17	3
Ndufv1	6.36E-24	########	0.699	0.448	1.06E-19	3
Exoc3l4	1.25E-162	########	0.354	0.034	2.09E-158	3
Snhg12	2.97E-33	########	0.547	0.245	4.97E-29	3
Strn3	3.90E-24	########	0.741	0.464	6.53E-20	3
Fam171a1	8.35E-189	########	0.411	0.04	1.40E-184	3
Minos1	2.76E-16	########	0.883	0.768	4.63E-12	3
Tnfrsf1a	3.64E-15	########	0.769	0.64	6.09E-11	3
Rpl29	2.57E-13	########	0.946	0.91	4.30E-09	3
Atraid	7.96E-23	########	0.734	0.472	1.33E-18	3
Ica1	2.93E-112	########	0.434	0.074	4.90E-108	3
Dctn2	1.10E-15	########	0.763	0.568	1.84E-11	3
Smarce1	2.36E-21	########	0.759	0.539	3.94E-17	3
Pnp	6.57E-16	########	0.832	0.656	1.10E-11	3
Uchl1	1.31E-160	########	0.345	0.032	2.20E-156	3
Msi2	2.19E-30	########	0.459	0.186	3.67E-26	3
Rcan1	4.66E-35	########	0.424	0.158	7.80E-31	3
Pigp	1.61E-35	########	0.573	0.255	2.69E-31	3
Thra	4.85E-39	########	0.481	0.187	8.12E-35	3
Qdpr	2.15E-44	########	0.601	0.256	3.59E-40	3
Cyb561	1.87E-198	########	0.396	0.034	3.13E-194	3
Lsm2	5.67E-18	########	0.589	0.362	9.48E-14	3
Psmc3	2.26E-16	########	0.791	0.636	3.79E-12	3
Fubp1	1.01E-20	########	0.75	0.516	1.69E-16	3
Nsg1	2.59E-50	########	0.294	0.067	4.33E-46	3
Rps15	5.96E-20	########	0.994	0.935	9.97E-16	3
Skp1a	3.39E-17	########	0.75	0.533	5.68E-13	3
Eef1d	4.77E-14	########	0.87	0.73	7.99E-10	3
Eif6	8.86E-13	########	0.747	0.62	1.48E-08	3
Ndufs2	1.08E-15	########	0.756	0.612	1.81E-11	3
Apc	2.11E-30	########	0.63	0.325	3.54E-26	3
Ackr3	8.66E-59	########	0.358	0.083	1.45E-54	3
Myadm	9.61E-17	########	0.677	0.433	1.61E-12	3
Rcn2	4.90E-31	########	0.582	0.272	8.19E-27	3
Smarcd1	5.18E-46	########	0.519	0.195	8.66E-42	3
Ssrp1	4.05E-20	########	0.696	0.445	6.78E-16	3
11-Sep	1.25E-25	########	0.718	0.409	2.10E-21	3
Phc2	1.02E-35	########	0.639	0.317	1.71E-31	3
Magoh	6.97E-18	########	0.801	0.611	1.17E-13	3
Zhx3	7.09E-90	########	0.421	0.085	1.19E-85	3
Fabp5	5.07E-25	########	0.807	0.556	8.48E-21	3
Uqcr11	2.07E-17	########	0.902	0.784	3.46E-13	3
Camta1	1.27E-31	########	0.566	0.267	2.12E-27	3

Mrps6	4.05E-49	########	0.522	0.186	6.77E-45	3
Stub1	3.90E-20	########	0.709	0.474	6.53E-16	3
Tjp2	3.73E-94	########	0.468	0.099	6.23E-90	3
Rabac1	3.53E-14	########	0.854	0.827	5.91E-10	3
BC028528	7.04E-22	########	0.601	0.344	1.18E-17	3
Tor1aip2	1.41E-30	########	0.642	0.338	2.36E-26	3
Arglu1	1.84E-14	########	0.797	0.611	3.07E-10	3
Eif4a2	4.17E-14	########	0.807	0.601	6.97E-10	3
Mrrf	4.73E-65	########	0.459	0.128	7.91E-61	3
Sbf2	6.23E-40	########	0.459	0.165	1.04E-35	3
Znrf1	1.46E-25	########	0.665	0.406	2.44E-21	3
1110001J0	1.04E-27	########	0.652	0.357	1.73E-23	3
Tmem44	0	########	0.307	0.005	0	3
Crybb3	3.00E-271	########	0.241	0.005	5.01E-267	3
Snrpd2	1.08E-09	########	0.788	0.691	1.81E-05	3
Ccnl1	9.36E-14	########	0.877	0.751	1.57E-09	3
Drap1	9.00E-17	########	0.848	0.696	1.51E-12	3
Snrpe	3.17E-12	########	0.88	0.775	5.31E-08	3
Sumo3	2.25E-20	########	0.706	0.457	3.76E-16	3
Dynlt3	1.78E-30	########	0.661	0.355	2.97E-26	3
Znrd1	1.57E-22	########	0.699	0.446	2.63E-18	3
Pqlc1	2.30E-24	########	0.592	0.342	3.85E-20	3
Nudt4	1.30E-16	########	0.731	0.511	2.17E-12	3
Snrpf	4.68E-11	########	0.858	0.703	7.83E-07	3
Lipe	1.17E-31	########	0.37	0.136	1.95E-27	3
Capns1	1.57E-15	########	0.848	0.75	2.63E-11	3
Vcp	3.51E-15	########	0.851	0.722	5.88E-11	3
Tgfb1i1	1.12E-112	########	0.43	0.071	1.88E-108	3
Ngfrap1	3.70E-34	########	0.563	0.248	6.18E-30	3
Smim11	1.84E-18	########	0.706	0.466	3.08E-14	3
Map4k4	1.81E-16	########	0.826	0.657	3.03E-12	3
Frmd4a	2.36E-52	########	0.503	0.168	3.95E-48	3
Aida	1.29E-28	########	0.589	0.294	2.16E-24	3
Nr1d1	1.33E-65	########	0.424	0.107	2.22E-61	3
Ddx3x	3.77E-11	########	0.861	0.713	6.31E-07	3
Hdlbp	1.18E-22	########	0.816	0.592	1.98E-18	3
Pak1ip1	2.30E-17	########	0.677	0.468	3.84E-13	3
Prdx1	1.12E-19	########	0.905	0.749	1.88E-15	3
Gspt1	2.79E-18	########	0.737	0.5	4.66E-14	3
Alg14	2.84E-64	########	0.506	0.154	4.75E-60	3
Mrpl2	3.60E-24	########	0.592	0.325	6.03E-20	3
Cops7a	3.20E-41	########	0.551	0.231	5.36E-37	3
Apol10b	1.01E-271	########	0.171	0.001	1.69E-267	3
Hint2	1.16E-28	########	0.646	0.355	1.95E-24	3
Ppp3ca	1.62E-10	########	0.82	0.663	2.71E-06	3
Adm	2.30E-54	########	0.256	0.05	3.85E-50	3
Slc5a3	1.13E-36	########	0.478	0.181	1.90E-32	3
Mras	8.27E-96	########	0.351	0.057	1.38E-91	3
Wdr83os	2.96E-17	########	0.772	0.593	4.95E-13	3
Acadl	3.36E-21	########	0.734	0.487	5.63E-17	3
Car2	3.46E-41	########	0.184	0.034	5.79E-37	3
B230219D2	7.22E-19	########	0.737	0.504	1.21E-14	3

Pdgfa	3.05E-36	########	0.351	0.116	5.10E-32	3
Atp9a	3.39E-142	########	0.367	0.042	5.68E-138	3
Igf2	3.36E-43	########	0.101	0.01	5.63E-39	3
Rassf1	6.94E-19	########	0.68	0.424	1.16E-14	3
Lman1	1.44E-30	########	0.712	0.385	2.40E-26	3
Psma5	4.90E-16	########	0.778	0.626	8.19E-12	3
Gjc1	8.70E-100	########	0.348	0.053	1.45E-95	3
Kdelr1	7.45E-16	########	0.75	0.555	1.25E-11	3
Rbbp7	8.34E-22	########	0.696	0.402	1.40E-17	3
Rpl7	9.91E-17	########	0.987	0.966	1.66E-12	3
Ywhah	1.76E-14	########	0.813	0.679	2.95E-10	3
Txndc17	9.98E-15	########	0.848	0.754	1.67E-10	3
Nxf1	1.44E-20	########	0.731	0.507	2.42E-16	3
Spata6	1.08E-47	########	0.525	0.19	1.80E-43	3
Sec62	1.62E-16	########	0.889	0.761	2.70E-12	3
Itga3	2.42E-269	########	0.377	0.019	4.05E-265	3
Cd320	1.47E-54	########	0.437	0.127	2.46E-50	3
Psma6	9.76E-13	########	0.797	0.674	1.63E-08	3
Wipi2	5.87E-28	########	0.566	0.284	9.82E-24	3
Itpripl1	8.76E-27	########	0.332	0.125	1.47E-22	3
Ifit1	3.55E-07	########	0.139	0.065	########	3
Amotl2	2.05E-53	########	0.37	0.096	3.43E-49	3
Eif5a	2.02E-09	########	0.908	0.862	3.38E-05	3
U2af1	3.48E-12	########	0.731	0.593	5.83E-08	3
Pxdc1	6.43E-78	########	0.424	0.093	1.08E-73	3
Lzts2	2.16E-92	########	0.405	0.076	3.62E-88	3
Eif3a	1.37E-13	########	0.873	0.738	2.29E-09	3
B4galt4	9.11E-145	########	0.37	0.042	1.53E-140	3
Reep3	6.88E-11	########	0.731	0.576	1.15E-06	3
Rpl41	1.78E-11	########	0.994	0.992	2.97E-07	3
Rapgef1	7.32E-28	########	0.528	0.254	1.22E-23	3
Mtmr11	2.18E-204	########	0.392	0.032	3.64E-200	3
Enpp4	4.72E-104	########	0.38	0.06	7.89E-100	3
Tmem255b	0	########	0.285	0.002	0	3
Snai1	3.08E-82	########	0.377	0.072	5.15E-78	3
Tra2b	1.50E-17	########	0.851	0.644	2.51E-13	3
Psmd3	5.07E-22	########	0.661	0.408	8.49E-18	3
Cnot6	6.23E-20	########	0.633	0.392	1.04E-15	3
Pdia5	2.28E-60	########	0.383	0.096	3.81E-56	3
Fam13c	8.43E-188	########	0.345	0.027	1.41E-183	3
Gmpr	5.87E-188	########	0.408	0.039	9.82E-184	3
Anapc5	7.21E-18	########	0.718	0.472	1.21E-13	3
H2afv	2.17E-15	########	0.807	0.606	3.63E-11	3
Notch4	9.88E-254	########	0.326	0.015	1.65E-249	3
Stoml2	2.01E-24	########	0.639	0.367	3.36E-20	3
Kif1b	3.40E-15	########	0.642	0.391	5.69E-11	3
Polr2c	9.12E-20	########	0.69	0.469	1.53E-15	3
Xiap	3.46E-12	########	0.722	0.548	5.80E-08	3
Erdr1	6.35E-20	########	0.671	0.409	1.06E-15	3
Tbcb	3.10E-16	########	0.794	0.639	5.19E-12	3
Hoxd8	7.00E-206	########	0.364	0.027	1.17E-201	3
Tacc2	4.61E-54	########	0.326	0.078	7.72E-50	3

Irf2bpl	5.05E-22	########	0.519	0.267	8.45E-18	3
Plekha3	4.56E-38	########	0.516	0.204	7.64E-34	3
Tal1	0	########	0.421	0.019	0	3
Senp6	5.13E-16	########	0.766	0.561	8.59E-12	3
Phldb2	2.63E-87	########	0.373	0.067	4.39E-83	3
Acyp2	2.51E-102	########	0.427	0.078	4.19E-98	3
Pomp	1.98E-11	########	0.883	0.814	3.32E-07	3
Glod4	7.96E-24	########	0.661	0.405	1.33E-19	3
Dync1li2	5.10E-26	########	0.544	0.262	8.54E-22	3
Lix1l	1.25E-85	########	0.459	0.099	2.09E-81	3
Rbm18	1.40E-30	########	0.497	0.225	2.34E-26	3
Dll1	9.97E-219	########	0.247	0.009	1.67E-214	3
Arhgap23	3.67E-50	########	0.427	0.134	6.14E-46	3
Pgm1	2.56E-32	########	0.573	0.279	4.28E-28	3
Nr1d2	8.39E-35	########	0.421	0.156	1.40E-30	3
Timeless	4.59E-48	########	0.358	0.101	7.68E-44	3
Cox7a2	2.43E-13	########	0.924	0.868	4.06E-09	3
Mical3	5.13E-99	########	0.396	0.07	8.58E-95	3
Bbx	3.14E-20	########	0.541	0.291	5.25E-16	3
Sfpq	4.18E-12	########	0.873	0.763	6.99E-08	3
Acer2	3.57E-200	########	0.297	0.017	5.97E-196	3
Ppp1r14a	1.40E-149	########	0.313	0.028	2.35E-145	3
Rasa1	1.75E-26	########	0.614	0.333	2.93E-22	3
Srsf10	1.04E-20	########	0.759	0.523	1.74E-16	3
Tcf7l1	1.48E-151	########	0.383	0.043	2.48E-147	3
Nxpe2	1.09E-291	########	0.326	0.012	1.83E-287	3
H2-Q4	2.99E-22	########	0.446	0.205	5.01E-18	3
Cnih1	1.11E-25	########	0.658	0.381	1.85E-21	3
Pde12	3.48E-24	########	0.383	0.166	5.82E-20	3
Nr2f6	7.35E-45	########	0.509	0.186	1.23E-40	3
Aig1	9.36E-40	########	0.472	0.174	1.57E-35	3
Vgll4	4.21E-32	########	0.623	0.309	7.04E-28	3
Tcaf1	7.13E-91	########	0.396	0.074	1.19E-86	3
Chst1	1.54E-122	########	0.304	0.032	2.58E-118	3
Dnpep	1.95E-27	########	0.585	0.308	3.26E-23	3
Crk	3.97E-17	########	0.696	0.483	6.64E-13	3
Plekhg5	5.35E-144	########	0.335	0.034	8.96E-140	3
Ccdc28b	8.75E-31	########	0.506	0.226	1.46E-26	3
Dusp23	1.57E-55	########	0.418	0.119	2.63E-51	3
Ing1	2.72E-17	########	0.661	0.414	4.56E-13	3
Leprot	6.31E-20	########	0.642	0.401	1.06E-15	3
Pnn	5.94E-13	########	0.715	0.504	9.95E-09	3
Ebf3	7.92E-198	########	0.386	0.032	1.32E-193	3
Coq10b	2.08E-21	########	0.699	0.437	3.47E-17	3
Adamts5	4.01E-19	########	0.234	0.083	6.71E-15	3
Taok2	3.37E-34	########	0.484	0.196	5.64E-30	3
Hmox2	5.82E-16	########	0.69	0.508	9.74E-12	3
Arl5b	9.05E-25	########	0.468	0.216	1.51E-20	3
Egln1	2.37E-12	########	0.614	0.388	3.96E-08	3
Zbtb10	2.73E-90	########	0.367	0.065	4.57E-86	3
Rpl22l1	4.29E-13	########	0.94	0.802	7.19E-09	3
Gtf2i	1.83E-31	########	0.608	0.292	3.06E-27	3

Trip10	1.32E-78	########	0.475	0.116	2.21E-74	3
Zfpm1	5.98E-126	########	0.418	0.059	1.00E-121	3
Vamp3	5.48E-18	########	0.674	0.44	9.17E-14	3
Rpl9	2.78E-15	########	0.994	0.975	4.65E-11	3
Wars	2.49E-42	########	0.513	0.197	4.17E-38	3
Arsa	1.94E-74	########	0.437	0.104	3.25E-70	3
Snrpc	7.38E-13	########	0.763	0.606	1.24E-08	3
Uqcrc2	4.49E-13	########	0.766	0.56	7.51E-09	3
Tmem30a	1.24E-14	########	0.753	0.59	2.07E-10	3
Epha4	1.81E-118	########	0.247	0.022	3.02E-114	3
Gemin7	1.37E-18	########	0.652	0.439	2.29E-14	3
Adrm1	2.20E-19	########	0.62	0.388	3.68E-15	3
Ipo7	2.99E-25	########	0.661	0.357	5.00E-21	3
Bax	1.99E-10	########	0.744	0.616	3.34E-06	3
Maoa	2.95E-73	########	0.358	0.074	4.94E-69	3
Pcdh19	3.96E-51	########	0.288	0.064	6.63E-47	3
Cdv3	5.19E-16	########	0.807	0.634	8.68E-12	3
Cdc37	5.36E-13	########	0.772	0.642	8.98E-09	3
Purb	8.02E-14	########	0.842	0.706	1.34E-09	3
Stx12	6.21E-22	########	0.557	0.316	1.04E-17	3
Syt15	0	########	0.234	0	0	3
Hs3st1	5.85E-149	########	0.329	0.031	9.79E-145	3
Gbp4	2.91E-74	########	0.301	0.049	4.87E-70	3
Ano6	6.56E-22	########	0.684	0.417	1.10E-17	3
Serpinb9	5.26E-96	########	0.373	0.061	8.81E-92	3
Ptbp1	8.87E-19	########	0.737	0.504	1.48E-14	3
Ube2m	4.76E-11	########	0.775	0.636	7.97E-07	3
Pdia3	8.14E-15	########	0.88	0.75	1.36E-10	3
Tnfaip8l1	3.84E-143	########	0.335	0.034	6.42E-139	3
Acox1	2.50E-31	########	0.503	0.219	4.18E-27	3
Klk8	1.05E-20	########	0.253	0.091	1.76E-16	3
Rpl5	2.23E-18	########	0.984	0.904	3.72E-14	3
Uqcc2	9.64E-17	########	0.785	0.53	1.61E-12	3
Swi5	3.77E-15	########	0.845	0.688	6.30E-11	3
Rsrc2	3.46E-15	########	0.835	0.669	5.79E-11	3
Tnks1bp1	2.89E-50	########	0.408	0.121	4.84E-46	3
Rps12	1.53E-10	########	0.994	0.982	2.55E-06	3
Plekhg2	1.67E-41	########	0.472	0.168	2.80E-37	3
Orc6	4.94E-27	########	0.491	0.229	8.27E-23	3
Rpl7a	2.02E-10	########	0.972	0.918	3.38E-06	3
Nufip2	1.17E-06	########	0.766	0.633	########	3
Rps5	2.79E-11	########	0.991	0.936	4.66E-07	3
Prkd2	6.70E-47	########	0.494	0.168	1.12E-42	3
Ddb1	1.33E-23	########	0.696	0.415	2.22E-19	3
Trove2	4.57E-54	########	0.383	0.102	7.64E-50	3
Cyb5b	2.51E-17	########	0.56	0.33	4.20E-13	3
Fyn	1.48E-37	########	0.611	0.256	2.48E-33	3
Dag1	5.34E-41	########	0.541	0.212	8.93E-37	3
Syne2	6.78E-24	########	0.491	0.236	1.13E-19	3
Ndufb6	1.34E-10	########	0.747	0.594	2.24E-06	3
B3gnt3	0	########	0.358	0.009	0	3
Eif5b	6.34E-13	########	0.823	0.637	1.06E-08	3

Capn2	2.04E-18	########	0.649	0.372	3.41E-14	3
Dynll2	3.20E-30	########	0.646	0.305	5.35E-26	3
Psmb4	2.48E-13	########	0.858	0.765	4.15E-09	3
Ddrgk1	2.07E-14	########	0.734	0.515	3.47E-10	3
Hey1	1.40E-158	########	0.222	0.012	2.35E-154	3
Kpnb1	1.25E-12	########	0.633	0.43	2.09E-08	3
Sh3glb2	2.19E-39	########	0.484	0.193	3.67E-35	3
Stk19	2.02E-23	########	0.611	0.341	3.37E-19	3
Ednrb	1.57E-41	########	0.386	0.116	2.63E-37	3
mt-Nd4l	2.86E-10	########	0.883	0.746	4.79E-06	3
Yrdc	3.23E-24	########	0.573	0.304	5.41E-20	3
Stx6	3.00E-32	########	0.538	0.245	5.02E-28	3
Txnl1	4.94E-14	########	0.75	0.581	8.27E-10	3
Bcl2l1	2.42E-26	########	0.459	0.204	4.04E-22	3
Sncaip	0	########	0.266	0.002	0	3
Gimap9	2.32E-67	########	0.544	0.149	3.89E-63	3
Cks1b	7.93E-15	########	0.449	0.246	1.33E-10	3
Ahctf1	5.41E-26	########	0.544	0.269	9.06E-22	3
Mpdz	3.84E-54	########	0.313	0.071	6.42E-50	3
Dpysl2	1.74E-24	########	0.614	0.321	2.92E-20	3
Ptgs1	8.99E-26	########	0.354	0.145	1.50E-21	3
Plpp2	2.01E-55	########	0.408	0.117	3.36E-51	3
Vat1	4.52E-30	########	0.636	0.318	7.57E-26	3
Copg1	7.49E-22	########	0.566	0.298	1.25E-17	3
Mrpl15	1.89E-17	########	0.658	0.426	3.17E-13	3
Traf7	4.04E-23	########	0.56	0.302	6.77E-19	3
Csgalnact1	3.66E-64	########	0.38	0.084	6.13E-60	3
Yif1b	7.50E-16	########	0.595	0.368	1.26E-11	3
Shroom4	0	########	0.345	0.004	0	3
Ginm1	2.60E-32	########	0.573	0.272	4.35E-28	3
Ebna1bp2	2.23E-13	########	0.639	0.438	3.73E-09	3
Kmt2a	1.48E-09	########	0.62	0.441	2.48E-05	3
Cox5a	1.49E-11	########	0.905	0.833	2.50E-07	3
Slc35b1	2.73E-20	########	0.62	0.355	4.57E-16	3
Pgm2l1	1.64E-47	########	0.389	0.114	2.74E-43	3
Zcchc17	5.30E-18	########	0.646	0.41	8.87E-14	3
Golgb1	1.65E-13	########	0.712	0.478	2.77E-09	3
Pcm1	1.25E-16	########	0.677	0.423	2.09E-12	3
Gbp9	1.66E-52	########	0.326	0.076	2.78E-48	3
Errfi1	1.42E-29	########	0.573	0.257	2.37E-25	3
Hotairm1	1.74E-57	########	0.329	0.075	2.92E-53	3
1500011K1	4.75E-26	########	0.589	0.305	7.95E-22	3
Jmjd6	5.65E-21	########	0.56	0.322	9.45E-17	3
Eci2	4.29E-27	########	0.639	0.358	7.17E-23	3
1810022K0	4.11E-10	########	0.728	0.544	6.88E-06	3
Ndufa5	1.38E-14	########	0.769	0.558	2.31E-10	3
Cnpy2	7.50E-18	########	0.734	0.494	1.26E-13	3
Hnrnpr	2.71E-14	########	0.696	0.463	4.54E-10	3
Arrb1	8.48E-35	########	0.494	0.199	1.42E-30	3
Plcb1	1.21E-28	########	0.38	0.147	2.02E-24	3
Ndufaf2	3.99E-28	########	0.5	0.234	6.67E-24	3
Cdc42ep2	4.19E-54	########	0.427	0.121	7.00E-50	3

Mtmr2	7.25E-38	########	0.446	0.167	1.21E-33	3
C1qbp	3.17E-19	########	0.658	0.401	5.30E-15	3
Sav1	9.95E-23	########	0.519	0.269	1.67E-18	3
Rps12-ps3	2.07E-12	########	0.737	0.54	3.46E-08	3
Pitpnm2	1.96E-142	########	0.37	0.042	3.28E-138	3
Dnajc10	1.39E-16	########	0.579	0.356	2.33E-12	3
Armcx4	1.08E-93	########	0.291	0.04	1.80E-89	3
Gatsl3	4.94E-74	########	0.335	0.064	8.26E-70	3
Mrpl55	4.78E-21	########	0.551	0.309	8.00E-17	3
Dtd1	2.55E-44	########	0.519	0.192	4.27E-40	3
Ddx24	8.88E-14	########	0.804	0.61	1.49E-09	3
Hk1	1.70E-24	########	0.598	0.324	2.85E-20	3
AU021092	0	########	0.266	0	0	3
Slc39a1	6.96E-17	########	0.655	0.406	1.17E-12	3
Rbm8a	4.55E-15	########	0.794	0.663	7.61E-11	3
Acot9	2.14E-20	########	0.592	0.353	3.58E-16	3
Ctgf	9.78E-73	########	0.316	0.056	1.64E-68	3
Fat4	4.80E-181	########	0.272	0.016	8.03E-177	3
Sh3bgrl	1.31E-20	########	0.861	0.604	2.20E-16	3
Esf1	8.58E-22	########	0.617	0.351	1.44E-17	3
Pam16	5.66E-22	########	0.614	0.348	9.47E-18	3
Zfp521	2.35E-128	########	0.335	0.039	3.92E-124	3
Snrpa1	2.55E-18	########	0.614	0.373	4.27E-14	3
AW112010	3.67E-15	########	0.509	0.302	6.13E-11	3
Mob2	2.00E-27	########	0.509	0.24	3.35E-23	3
Ssh1	2.33E-47	########	0.434	0.135	3.90E-43	3
Matr3	2.45E-12	########	0.756	0.568	4.11E-08	3
Tipin	1.15E-26	########	0.491	0.223	1.93E-22	3
Rassf9	1.08E-248	########	0.269	0.009	1.81E-244	3
Mesdc1	4.14E-27	########	0.437	0.198	6.92E-23	3
Suclg2	2.72E-26	########	0.503	0.232	4.55E-22	3
Abcb1b	6.06E-29	########	0.351	0.128	1.01E-24	3
Actl6a	1.94E-19	########	0.595	0.353	3.24E-15	3
Nudc	4.19E-15	########	0.75	0.531	7.00E-11	3
Cr1l	2.42E-14	########	0.718	0.543	4.05E-10	3
Rplp1	7.85E-15	########	0.997	0.974	1.31E-10	3
Ndufb5	3.03E-13	########	0.87	0.723	5.07E-09	3
Lpp	1.28E-33	########	0.617	0.274	2.13E-29	3
Stk25	1.84E-39	########	0.551	0.23	3.07E-35	3
Mydgf	1.82E-12	########	0.731	0.509	3.05E-08	3
Klf10	2.07E-20	########	0.576	0.314	3.46E-16	3
Nrip1	1.48E-22	########	0.585	0.298	2.48E-18	3
Eif4g1	2.44E-13	########	0.734	0.534	4.09E-09	3
Nsmce1	6.63E-18	########	0.687	0.453	1.11E-13	3
Rps18	7.28E-11	########	0.991	0.911	1.22E-06	3
Mtx2	1.80E-28	########	0.598	0.313	3.01E-24	3
Denr	6.48E-13	########	0.693	0.477	1.08E-08	3
Psma4	1.85E-12	########	0.835	0.69	3.09E-08	3
Mapk12	1.11E-161	########	0.32	0.027	1.86E-157	3
Fkbp7	3.28E-71	########	0.475	0.115	5.48E-67	3
Wtip	3.43E-77	########	0.421	0.093	5.74E-73	3
Angptl4	1.42E-33	########	0.405	0.143	2.38E-29	3

Oaz2	3.15E-25	########	0.576	0.304	5.28E-21	3
Thsd1	0	########	0.345	0.01	0	3
Tmem57	1.17E-25	########	0.525	0.252	1.96E-21	3
Rab5c	1.51E-10	########	0.82	0.741	2.52E-06	3
Zcrb1	7.78E-10	########	0.744	0.612	1.30E-05	3
Flywch1	1.68E-62	########	0.361	0.085	2.81E-58	3
Eif3c	5.72E-10	########	0.858	0.764	9.57E-06	3
Tsc22d2	3.45E-16	########	0.582	0.342	5.77E-12	3
Hdgf	2.00E-13	########	0.759	0.569	3.35E-09	3
Ctdsp2	2.57E-17	########	0.693	0.462	4.29E-13	3
Cdipt	2.93E-17	########	0.617	0.399	4.91E-13	3
Pttg1ip	2.11E-15	########	0.649	0.421	3.53E-11	3
Fam189a2	1.11E-209	########	0.285	0.014	1.86E-205	3
Hif1a	5.69E-15	########	0.826	0.635	9.52E-11	3
Pam	1.93E-26	########	0.56	0.277	3.23E-22	3
Slc25a25	1.40E-35	########	0.396	0.139	2.34E-31	3
Praf2	1.80E-53	########	0.424	0.121	3.00E-49	3
Gar1	2.56E-31	########	0.516	0.228	4.28E-27	3
Polr2h	8.04E-24	########	0.56	0.291	1.35E-19	3
Psmd12	3.06E-14	########	0.715	0.53	5.12E-10	3
Atp1b3	6.55E-10	########	0.82	0.709	1.10E-05	3
Rpl15	6.02E-13	########	0.921	0.826	1.01E-08	3
Ssbp3	8.60E-31	########	0.551	0.259	1.44E-26	3
Ak3	4.54E-40	########	0.468	0.175	7.60E-36	3
Sec14l1	6.26E-13	########	0.614	0.403	1.05E-08	3
Art3	1.05E-63	########	0.184	0.023	1.75E-59	3
Pde8a	2.65E-35	########	0.503	0.21	4.44E-31	3
Mrpl51	2.46E-13	0.271543	0.725	0.52	4.12E-09	3
Mrps24	2.87E-10	########	0.797	0.649	4.81E-06	3
Syncrip	7.17E-12	########	0.75	0.561	1.20E-07	3
Plscr4	1.12E-104	########	0.316	0.042	1.88E-100	3
Camk2n1	2.08E-59	########	0.323	0.071	3.49E-55	3
Ccdc59	2.30E-15	########	0.699	0.48	3.85E-11	3
Myl9	9.29E-39	########	0.209	0.045	1.55E-34	3
Ivns1abp	8.63E-19	########	0.551	0.311	1.44E-14	3
Prkrir	1.04E-33	########	0.557	0.245	1.73E-29	3
Ripk3	9.45E-20	########	0.459	0.244	1.58E-15	3
Psmb1	4.69E-12	########	0.877	0.836	7.86E-08	3
Lepr	1.14E-242	########	0.199	0.004	1.91E-238	3
Edf1	3.24E-10	########	0.832	0.806	5.42E-06	3
Hoxb4	2.38E-50	########	0.345	0.088	3.98E-46	3
Mrpl12	5.07E-17	########	0.677	0.443	8.48E-13	3
Pik3ip1	1.04E-22	########	0.332	0.129	1.75E-18	3
Cntln	7.20E-43	########	0.456	0.16	1.20E-38	3
Rgs3	2.26E-30	########	0.494	0.213	3.78E-26	3
Kif26a	0	########	0.313	0.005	0	3
Jag1	1.20E-42	########	0.316	0.086	2.00E-38	3
Synj2bp	1.04E-22	########	0.601	0.337	1.74E-18	3
Crtap	5.98E-43	########	0.509	0.186	1.00E-38	3
Tomm20	2.82E-12	########	0.87	0.755	4.72E-08	3
Rpl10a	4.61E-08	########	0.981	0.87	########	3
Csde1	6.15E-11	########	0.877	0.763	1.03E-06	3

Emc4	2.64E-25	########	0.589	0.312	4.42E-21	3
Hoxd9	2.05E-259	########	0.291	0.011	3.43E-255	3
Fbl	3.24E-14	########	0.715	0.486	5.43E-10	3
Pebp1	3.54E-13	########	0.801	0.614	5.92E-09	3
Ufc1	1.18E-10	########	0.759	0.599	1.97E-06	3
Higd1b	9.91E-91	########	0.127	0.007	1.66E-86	3
Huwe1	6.26E-13	########	0.706	0.494	1.05E-08	3
Spry4	1.91E-156	########	0.297	0.024	3.19E-152	3
Wsb2	2.02E-24	########	0.595	0.328	3.39E-20	3
Fdx1	8.27E-34	########	0.547	0.238	1.38E-29	3
Impdh1	4.66E-39	########	0.491	0.188	7.80E-35	3
Ski	3.03E-19	########	0.642	0.377	5.08E-15	3
Synm	6.16E-126	########	0.329	0.037	1.03E-121	3
Rps14	3.18E-12	########	0.997	0.984	5.32E-08	3
Phb2	2.25E-13	########	0.759	0.592	3.76E-09	3
1110004E0	2.90E-38	########	0.43	0.159	4.85E-34	3
Tgoln1	7.68E-15	########	0.782	0.581	1.29E-10	3
Ext1	4.23E-26	########	0.535	0.251	7.08E-22	3
Nbea	3.71E-136	########	0.342	0.038	6.21E-132	3
Ptov1	1.61E-33	########	0.63	0.286	2.69E-29	3
Ptges3	2.31E-11	########	0.81	0.638	3.86E-07	3
Pdcl3	1.67E-17	########	0.687	0.463	2.79E-13	3
Mrpl20	4.21E-09	########	0.794	0.691	7.05E-05	3
Myef2	1.29E-40	########	0.405	0.132	2.17E-36	3
Eif4b	1.05E-14	########	0.788	0.568	1.75E-10	3
Ube2r2	2.08E-13	########	0.715	0.544	3.48E-09	3
Tgfbr2	4.12E-15	########	0.661	0.403	6.90E-11	3
Polr2e	2.16E-15	########	0.731	0.489	3.62E-11	3
Psmb7	6.55E-13	########	0.756	0.603	1.10E-08	3
Smyd2	1.03E-43	########	0.418	0.135	1.73E-39	3
Tanc2	4.66E-24	########	0.278	0.099	7.81E-20	3
Pdcd5	1.11E-11	########	0.801	0.651	1.85E-07	3
Nucks1	2.86E-11	########	0.794	0.573	4.79E-07	3
Mrpl27	1.06E-19	########	0.636	0.38	1.78E-15	3
Snrpa	2.61E-18	########	0.595	0.361	4.37E-14	3
Pcna	1.85E-15	########	0.614	0.377	3.09E-11	3
Mageh1	1.31E-103	########	0.37	0.057	2.20E-99	3
Hspa1b	6.25E-25	########	0.345	0.132	1.05E-20	3
Fam212a	5.59E-66	########	0.31	0.061	9.35E-62	3
C1d	3.03E-14	########	0.699	0.501	5.08E-10	3
Nqo1	1.31E-70	########	0.259	0.042	2.18E-66	3
Gm26802	2.08E-41	########	0.171	0.03	3.49E-37	3
Dusp3	2.60E-14	########	0.582	0.35	4.34E-10	3
Btbd7	1.03E-31	########	0.544	0.254	1.72E-27	3
Pyurf	4.58E-35	########	0.497	0.205	7.67E-31	3
Mrps5	5.79E-26	########	0.576	0.281	9.68E-22	3
Stard13	9.30E-99	########	0.288	0.037	1.56E-94	3
Higd2a	5.98E-13	########	0.772	0.624	1.00E-08	3
Nfic	1.65E-11	########	0.668	0.484	2.77E-07	3
Mcc	1.68E-70	########	0.294	0.052	2.80E-66	3
Cobll1	2.58E-108	########	0.351	0.049	4.31E-104	3
Ndufb2	4.09E-12	########	0.759	0.6	6.84E-08	3

Cops5	1.44E-11	########	0.677	0.479	2.40E-07	3
Actn1	8.80E-16	########	0.703	0.449	1.47E-11	3
Ctps	1.66E-50	########	0.424	0.129	2.78E-46	3
Bod1	1.84E-46	########	0.484	0.168	3.08E-42	3
Tbl1x	2.72E-16	########	0.608	0.363	4.56E-12	3
Mtch2	3.86E-14	########	0.655	0.426	6.46E-10	3
Phactr4	1.79E-25	########	0.475	0.218	2.99E-21	3
Elp5	1.88E-28	########	0.528	0.253	3.15E-24	3
Glrx5	1.74E-14	########	0.68	0.461	2.92E-10	3
Cops6	6.83E-13	########	0.715	0.521	1.14E-08	3
Dnajc1	8.66E-21	########	0.652	0.365	1.45E-16	3
Pald1	1.62E-127	########	0.266	0.023	2.72E-123	3
Sptlc2	2.48E-11	########	0.684	0.517	4.16E-07	3
Hsph1	2.21E-18	########	0.418	0.201	3.71E-14	3
Nid2	3.98E-51	########	0.285	0.064	6.66E-47	3
Mib1	5.42E-21	########	0.551	0.301	9.07E-17	3
Cttnbp2nl	3.45E-23	########	0.449	0.201	5.77E-19	3
Noct	1.93E-19	########	0.516	0.286	3.22E-15	3
Tmem11	2.02E-14	########	0.516	0.306	3.38E-10	3
Golm1	1.05E-29	########	0.415	0.174	1.76E-25	3
Txndc12	4.87E-25	########	0.487	0.241	8.15E-21	3
Cyth2	4.01E-18	########	0.554	0.329	6.71E-14	3
Pitpnb	1.69E-17	########	0.604	0.363	2.82E-13	3
Tnfsf12	6.68E-31	########	0.551	0.243	1.12E-26	3
Plat	1.60E-58	########	0.342	0.077	2.67E-54	3
Sec63	1.26E-10	########	0.623	0.439	2.11E-06	3
Eif2b2	2.12E-20	########	0.544	0.309	3.55E-16	3
Gnl3	1.54E-18	########	0.576	0.312	2.57E-14	3
Akt1s1	5.56E-31	########	0.522	0.231	9.31E-27	3
Gbp2	2.27E-35	########	0.418	0.146	3.79E-31	3
Nrep	4.15E-89	########	0.272	0.036	6.94E-85	3
Ttll7	1.60E-117	########	0.288	0.031	2.67E-113	3
Pcdh12	0	########	0.272	0.001	0	3
Mrps7	4.55E-17	########	0.604	0.372	7.62E-13	3
Ttc9	1.04E-203	########	0.244	0.01	1.73E-199	3
Snrnp48	4.19E-18	########	0.506	0.274	7.00E-14	3
Map4k5	4.31E-44	########	0.402	0.128	7.21E-40	3
Vsig2	0	########	0.288	0.002	0	3
Sdcbp2	7.90E-193	########	0.247	0.011	1.32E-188	3
Srpk1	2.06E-16	########	0.658	0.425	3.45E-12	3
Dazap1	4.79E-17	########	0.633	0.398	8.02E-13	3
Eif1a	8.35E-18	########	0.722	0.464	1.40E-13	3
Tmem245	1.36E-38	########	0.453	0.165	2.27E-34	3
Psma2	3.08E-08	########	0.858	0.799	########	3
Nolc1	1.50E-22	########	0.566	0.287	2.51E-18	3
Rsu1	9.83E-12	########	0.772	0.592	1.65E-07	3
Cops2	7.07E-20	########	0.646	0.373	1.18E-15	3
Kctd12b	5.97E-65	########	0.253	0.042	9.98E-61	3
Hadhb	4.32E-15	########	0.639	0.416	7.23E-11	3
Cdc42ep5	1.06E-52	########	0.377	0.097	1.77E-48	3
Slc20a1	2.20E-21	########	0.478	0.24	3.69E-17	3
Cers5	1.75E-26	########	0.563	0.28	2.93E-22	3

Rpn2	8.96E-12	########	0.756	0.561	1.50E-07	3
Map3k11	1.38E-30	########	0.525	0.237	2.31E-26	3
Lats2	5.13E-12	########	0.522	0.323	8.59E-08	3
Adh5	2.04E-16	########	0.718	0.485	3.41E-12	3
Polr2f	1.10E-11	########	0.744	0.56	1.84E-07	3
Nucb1	1.72E-14	########	0.699	0.495	2.88E-10	3
Cfap36	3.12E-28	########	0.563	0.262	5.23E-24	3
Naca	8.90E-10	########	0.949	0.906	1.49E-05	3
Psmc1	5.27E-15	########	0.623	0.408	8.82E-11	3
Ccdc34	4.77E-17	########	0.509	0.277	7.97E-13	3
Cisd1	5.29E-20	########	0.595	0.326	8.85E-16	3
Fam63b	3.18E-12	########	0.623	0.415	5.32E-08	3
Dapk2	1.07E-30	########	0.19	0.046	1.79E-26	3
Mcm3	6.54E-17	########	0.449	0.23	1.09E-12	3
Tomm7	1.11E-06	########	0.924	0.869	########	3
Nop16	3.68E-26	########	0.472	0.209	6.16E-22	3
Oaf	2.28E-44	########	0.443	0.144	3.82E-40	3
Med19	1.59E-35	########	0.509	0.212	2.66E-31	3
Zfp639	4.63E-19	########	0.427	0.213	7.74E-15	3
Rabggtb	1.88E-30	########	0.535	0.248	3.15E-26	3
Mpz	0	7.325014	0.719	0.009	0	4
Pmp22	8.38E-62	5.766782	0.895	0.175	1.40E-57	4
Cryab	6.62E-147	4.870765	0.965	0.08	1.11E-142	4
Mbp	9.24E-20	4.511228	0.702	0.322	1.55E-15	4
Mal	0	4.204199	0.772	0.003	0	4
Plp1	0	4.064732	0.93	0.009	0	4
Apod	1.46E-79	4.054813	0.702	0.071	2.45E-75	4
Gatm	1.26E-57	4.05437	0.825	0.158	2.12E-53	4
Cd9	4.33E-34	3.406633	0.965	0.547	7.24E-30	4
Secisbp2l	1.48E-22	3.15494	0.754	0.328	2.47E-18	4
Pou3f1	1.57E-134	3.015871	0.754	0.048	2.62E-130	4
Gpm6b	1.24E-108	2.998988	0.807	0.072	2.07E-104	4
Limch1	1.06E-212	2.87253	0.702	0.024	1.78E-208	4
Scd2	8.56E-37	2.821814	0.842	0.269	1.43E-32	4
Egfl8	1.98E-225	2.81897	0.719	0.024	3.31E-221	4
Ncmap	0	2.804338	0.561	0.004	0	4
Cnp	5.40E-39	2.802419	0.86	0.267	9.03E-35	4
Prx	0	2.736792	0.632	0.007	0	4
Timp3	1.87E-110	2.720427	0.93	0.094	3.14E-106	4
Ndrg1	1.19E-16	2.706361	0.719	0.361	1.99E-12	4
Cadm4	0	2.694184	0.719	0.009	0	4
Tuba1a	5.04E-28	2.628608	0.895	0.458	8.44E-24	4
Fxyd3	3.32E-152	2.626041	0.702	0.034	5.55E-148	4
Qk	2.41E-27	2.597567	0.965	0.661	4.03E-23	4
Dbi	1.69E-25	2.595551	0.965	0.725	2.82E-21	4
Dag1	2.18E-48	2.574124	0.86	0.22	3.64E-44	4
Kcna1	0	2.539073	0.772	0.002	0	4
Arpc1a	1.38E-34	2.536386	0.965	0.561	2.32E-30	4
Fos	4.51E-15	2.505232	0.93	0.593	7.55E-11	4
Mt3	0	2.494419	0.544	0.003	0	4
Ogn	8.78E-34	2.465926	0.456	0.067	1.47E-29	4
Hbegf	1.06E-30	2.42963	0.649	0.155	1.78E-26	4

Sparc	3.08E-47	2.405348	0.947	0.209	5.15E-43	4
Fxyd1	3.40E-64	2.400525	0.667	0.079	5.69E-60	4
Pllp	0	2.388223	0.579	0.003	0	4
Mt1	1.76E-21	2.386651	0.965	0.55	2.95E-17	4
Drp2	0	2.376188	0.526	0.003	0	4
Prnp	2.68E-50	2.37572	0.86	0.189	4.48E-46	4
Ldhb	6.33E-64	2.366436	0.842	0.145	1.06E-59	4
Nr4a2	2.14E-16	2.35375	0.719	0.305	3.58E-12	4
Cd59a	7.64E-170	2.324646	0.86	0.05	1.28E-165	4
Fam178b	3.13E-247	2.311078	0.491	0.009	5.24E-243	4
Sncg	2.23E-68	2.308604	0.509	0.042	3.73E-64	4
Cldn19	0	2.287912	0.579	0.001	0	4
Pla2g16	1.92E-42	2.25004	0.825	0.21	3.21E-38	4
Crlf1	1.88E-78	2.221265	0.614	0.054	3.14E-74	4
Mt2	3.44E-36	2.20746	0.912	0.261	5.76E-32	4
S100b	6.71E-271	2.144353	0.614	0.013	1.12E-266	4
Ugt8a	0	2.115435	0.561	0.003	0	4
Art3	1.37E-282	2.045259	0.807	0.024	2.30E-278	4
Itgb8	2.31E-172	2.026582	0.667	0.028	3.86E-168	4
Plekhb1	0	2.00549	0.807	0.002	0	4
Dst	1.43E-58	1.973641	0.895	0.172	2.40E-54	4
Ckb	7.43E-25	1.973246	0.737	0.258	1.24E-20	4
Klf9	2.55E-26	1.959211	0.772	0.254	4.27E-22	4
Sema3b	5.38E-277	1.954496	0.702	0.018	9.00E-273	4
Mlip	0	1.950614	0.561	0.001	0	4
Hes1	1.21E-24	1.925102	0.772	0.242	2.02E-20	4
Gldn	0	1.920279	0.561	0.005	0	4
Matn2	3.66E-70	1.919252	0.614	0.062	6.13E-66	4
Crip2	1.51E-47	1.91003	0.772	0.141	2.52E-43	4
Nes	2.40E-135	1.890299	0.667	0.036	4.01E-131	4
Fxyd6	1.69E-54	1.887133	0.544	0.06	2.83E-50	4
Igfbp5	1.30E-26	1.88667	0.491	0.091	2.18E-22	4
Emp2	6.61E-62	1.88089	0.702	0.096	1.11E-57	4
Hmgcs1	1.87E-14	1.880141	0.632	0.268	3.13E-10	4
Gap43	5.33E-20	1.877295	0.175	0.017	8.91E-16	4
Gjc3	0	1.840003	0.632	0.001	0	4
Aatk	4.80E-90	1.834869	0.737	0.071	8.03E-86	4
Col28a1	0	1.821533	0.614	0.006	0	4
Fosb	2.48E-15	1.819256	0.877	0.465	4.15E-11	4
Lmna	1.86E-23	1.81373	0.912	0.449	3.11E-19	4
Lgi4	1.74E-204	1.80405	0.544	0.014	2.91E-200	4
Sbspon	0	1.788397	0.544	0.005	0	4
Utrn	9.60E-29	1.786212	0.842	0.321	1.61E-24	4
Pdgfa	1.41E-48	1.777949	0.702	0.12	2.36E-44	4
Rasal2	6.96E-47	1.777716	0.596	0.087	1.16E-42	4
Rgcc	1.49E-16	1.766308	0.825	0.42	2.49E-12	4
Cpe	2.75E-130	1.760696	0.772	0.052	4.60E-126	4
Cd81	3.36E-23	1.735467	0.825	0.374	5.62E-19	4
Mfap5	1.77E-15	1.73236	0.386	0.089	2.96E-11	4
Map1b	2.06E-54	1.726351	0.702	0.106	3.45E-50	4
Csrp1	4.49E-25	1.723332	0.789	0.305	7.52E-21	4
Vim	2.25E-15	1.720359	0.965	0.899	3.76E-11	4

Vwa1	1.27E-103	1.706879	0.579	0.035	2.12E-99	4
Cd63	2.39E-15	1.700562	0.895	0.588	4.00E-11	4
Megf9	3.29E-34	1.691299	0.772	0.211	5.51E-30	4
Cltb	4.24E-28	1.676075	0.825	0.31	7.09E-24	4
Sptbn1	2.72E-24	1.663662	0.912	0.549	4.56E-20	4
Mag	1.93E-173	1.657907	0.544	0.017	3.23E-169	4
Pcbp4	4.94E-100	1.652945	0.825	0.084	8.26E-96	4
Cdkn1a	2.79E-07	1.648554	0.702	0.489	########	4
Tppp3	1.82E-18	1.641207	0.579	0.181	3.04E-14	4
Clstn1	5.89E-41	1.639102	0.614	0.108	9.85E-37	4
Nr4a1	8.22E-11	1.630267	0.702	0.384	1.38E-06	4
Bcas1	0	1.629829	0.526	0.004	0	4
Cfl2	4.36E-28	1.62691	0.737	0.248	7.30E-24	4
Sfrp5	0	1.62224	0.526	0.003	0	4
Chl1	4.81E-44	1.611524	0.509	0.063	8.05E-40	4
Sdc4	6.91E-22	1.608973	0.912	0.386	1.16E-17	4
Plekha4	1.44E-257	1.599917	0.544	0.011	2.41E-253	4
Sema5a	2.15E-70	1.597532	0.456	0.033	3.60E-66	4
Tubb2b	1.26E-33	1.595529	0.509	0.086	2.11E-29	4
Egr2	6.17E-41	1.590492	0.579	0.092	1.03E-36	4
Ccser2	3.55E-23	1.588484	0.667	0.214	5.94E-19	4
Rnf7	4.10E-22	1.56107	0.895	0.626	6.86E-18	4
Dynlrb1	1.46E-20	1.556147	0.93	0.679	2.44E-16	4
Ank3	2.03E-131	1.552601	0.719	0.045	3.39E-127	4
Pmepa1	5.20E-16	1.546822	0.579	0.194	8.70E-12	4
Jun	7.14E-16	1.54564	0.912	0.551	1.19E-11	4
Hspa12a	5.46E-175	1.536162	0.614	0.023	9.13E-171	4
Atp1a2	4.14E-169	1.530056	0.439	0.011	6.93E-165	4
Pdlim4	8.76E-12	1.527254	0.526	0.225	1.47E-07	4
Phlda3	3.88E-46	1.525807	0.684	0.117	6.50E-42	4
Slc44a1	7.91E-33	1.519352	0.772	0.223	1.32E-28	4
Sirt2	2.37E-19	1.513372	0.825	0.488	3.97E-15	4
Selm	4.44E-28	1.510788	0.825	0.244	7.43E-24	4
Fkbp1a	2.27E-20	1.506789	0.895	0.557	3.81E-16	4
Atf3	5.04E-08	1.495351	0.649	0.359	########	4
Gpr37l1	0	1.493778	0.509	0.001	0	4
Stmn1	3.98E-18	1.493408	0.789	0.322	6.66E-14	4
Dynll1	2.02E-15	1.488306	0.947	0.844	3.38E-11	4
Phlda1	1.61E-17	1.481755	0.789	0.345	2.70E-13	4
Fermt2	1.17E-60	1.480383	0.86	0.131	1.95E-56	4
Ptn	6.04E-20	1.479454	0.298	0.045	1.01E-15	4
Tubb2a	1.42E-14	1.470247	0.684	0.341	2.37E-10	4
Mfap3l	2.00E-144	1.460691	0.614	0.028	3.35E-140	4
Lamb1	2.03E-54	1.449029	0.684	0.095	3.40E-50	4
Hs2st1	9.05E-31	1.443784	0.719	0.213	1.51E-26	4
Tnfrsf12a	1.32E-11	1.442798	0.614	0.306	2.21E-07	4
Gnai1	6.18E-88	1.438375	0.596	0.045	1.03E-83	4
Ptms	8.65E-20	1.436107	0.895	0.5	1.45E-15	4
Cmtm5	0	1.430929	0.544	0	0	4
Hspg2	4.63E-54	1.423845	0.789	0.129	7.75E-50	4
Ptprf	2.01E-66	1.421802	0.614	0.064	3.36E-62	4
Serpina3n	3.41E-23	1.420535	0.439	0.081	5.71E-19	4

Gfra1	4.01E-64	1.420305	0.456	0.036	6.71E-60	4
Nenf	8.49E-24	1.41949	0.807	0.336	1.42E-19	4
Map1lc3a	1.66E-18	1.419259	0.772	0.381	2.78E-14	4
Dhh	6.87E-268	1.419129	0.544	0.01	1.15E-263	4
Cdh19	0	1.419111	0.596	0.002	0	4
Tead1	7.05E-66	1.417416	0.754	0.103	1.18E-61	4
Gjb1	0	1.413167	0.368	0.001	0	4
Cnn3	8.42E-47	1.411106	0.789	0.153	1.41E-42	4
Rhoc	8.05E-19	1.408984	0.667	0.224	1.35E-14	4
Jam3	3.74E-94	1.392816	0.649	0.051	6.26E-90	4
Ctnnal1	6.33E-93	1.390812	0.561	0.038	1.06E-88	4
Dusp15	0	1.390657	0.474	0.001	0	4
Oaf	5.20E-19	1.387654	0.526	0.152	8.69E-15	4
Shc4	2.49E-230	1.384737	0.509	0.011	4.16E-226	4
Kif1a	0	1.383254	0.579	0.009	0	4
Pea15a	9.12E-18	1.38257	0.667	0.263	1.53E-13	4
Epb41l2	4.43E-11	1.379866	0.632	0.371	7.41E-07	4
Kcna2	0	1.376668	0.491	0.005	0	4
Gas2l3	2.52E-21	1.369159	0.649	0.216	4.21E-17	4
Tmod2	4.01E-129	1.367625	0.632	0.034	6.71E-125	4
Rhobtb3	1.78E-61	1.358463	0.596	0.067	2.98E-57	4
Tspan15	1.18E-255	1.350012	0.737	0.022	1.97E-251	4
Prdx1	1.79E-09	1.342397	0.93	0.754	3.00E-05	4
Phgdh	1.06E-27	1.337908	0.649	0.165	1.77E-23	4
Serpine2	9.71E-42	1.332105	0.632	0.105	1.62E-37	4
Fut8	2.15E-20	1.330775	0.614	0.198	3.60E-16	4
Col5a3	2.29E-58	1.330243	0.649	0.078	3.84E-54	4
Serinc5	3.95E-34	1.327068	0.561	0.104	6.61E-30	4
Col20a1	2.61E-40	1.324399	0.105	0.003	4.37E-36	4
Cyb5r3	9.22E-17	1.324117	0.772	0.378	1.54E-12	4
Setd8	2.05E-11	1.320673	0.719	0.502	3.42E-07	4
Col16a1	4.08E-33	1.316595	0.491	0.078	6.83E-29	4
Gmfb	8.12E-13	1.30087	0.719	0.438	1.36E-08	4
Col18a1	3.83E-19	1.29885	0.421	0.092	6.41E-15	4
Egr1	1.26E-09	1.293124	0.719	0.366	2.11E-05	4
Tspan17	1.05E-26	1.283943	0.579	0.145	1.76E-22	4
Itga6	1.65E-27	1.282208	0.754	0.225	2.76E-23	4
Tubb3	7.89E-30	1.277613	0.298	0.032	1.32E-25	4
Cyr61	3.17E-18	1.270408	0.474	0.117	5.30E-14	4
Col4a1	9.33E-35	1.266532	0.684	0.13	1.56E-30	4
Cab39l	3.63E-20	1.26589	0.667	0.247	6.07E-16	4
C1ql3	0	1.261626	0.404	0.003	0	4
Rarres2	1.14E-13	1.255458	0.386	0.094	1.91E-09	4
Deb1	1.31E-12	1.25484	0.579	0.28	2.19E-08	4
Gadd45b	4.62E-07	1.248954	0.772	0.588	########	4
Cadm3	3.75E-116	1.248581	0.491	0.022	6.27E-112	4
Fa2h	0	1.245326	0.439	0.002	0	4
Mapk8ip1	1.53E-119	1.243839	0.614	0.035	2.56E-115	4
Mras	6.96E-39	1.241317	0.474	0.065	1.17E-34	4
Cxcl14	5.11E-13	1.237218	0.439	0.13	8.54E-09	4
Reln	0	1.230893	0.579	0.005	0	4
Peli2	2.85E-23	1.227706	0.579	0.162	4.77E-19	4

Lgals1	3.44E-10	1.223737	0.965	0.657	5.76E-06	4
Hspb2	1.43E-81	1.220581	0.491	0.032	2.39E-77	4
Itih5	1.19E-30	1.21431	0.526	0.1	1.99E-26	4
Hcn1	0	1.213996	0.439	0.001	0	4
Foxd3	0	1.213648	0.474	0.001	0	4
Tjp1	4.53E-60	1.211371	0.702	0.095	7.58E-56	4
Sostdc1	4.14E-186	1.209097	0.404	0.008	6.93E-182	4
Pebp1	4.28E-13	1.205927	0.807	0.619	7.16E-09	4
Dmd	8.38E-143	1.200856	0.649	0.032	1.40E-138	4
Gulp1	1.19E-74	1.198484	0.579	0.051	1.99E-70	4
Cd151	9.26E-25	1.196946	0.667	0.203	1.55E-20	4
Malat1	9.66E-14	1.19506	1	0.993	1.62E-09	4
Prkcdbp	2.19E-28	1.192113	0.684	0.154	3.67E-24	4
Ybx3	5.11E-13	1.191992	0.754	0.425	8.55E-09	4
Sox10	0	1.188886	0.614	0.001	0	4
Erbb3	3.61E-89	1.184159	0.509	0.032	6.05E-85	4
Arhgap19	2.17E-13	1.178474	0.474	0.161	3.63E-09	4
Dynlt3	2.42E-19	1.15331	0.754	0.363	4.05E-15	4
Ivns1abp	3.09E-20	1.152349	0.754	0.317	5.18E-16	4
Plekha7	1.93E-88	1.151693	0.421	0.022	3.23E-84	4
Tmed10	5.50E-16	1.148418	0.912	0.788	9.20E-12	4
Auts2	7.00E-30	1.14697	0.561	0.114	1.17E-25	4
Frmd3	0	1.146034	0.421	0.001	0	4
Smtn	8.29E-24	1.143452	0.491	0.109	1.39E-19	4
Ctnna1	4.20E-15	1.143015	0.754	0.425	7.04E-11	4
Socs3	2.83E-09	1.139574	0.825	0.544	4.74E-05	4
Sgk1	4.08E-07	1.139563	0.649	0.397	########	4
15-Sep	2.27E-13	1.132982	0.86	0.778	3.81E-09	4
Scd1	1.02E-27	1.128865	0.544	0.109	1.71E-23	4
mt-Cytb	2.20E-20	1.126781	1	0.964	3.68E-16	4
Itm2a	5.74E-39	1.12649	0.561	0.086	9.60E-35	4
Camk2n1	1.14E-36	1.124443	0.509	0.077	1.90E-32	4
Col4a2	6.13E-36	1.113695	0.649	0.116	1.03E-31	4
Cadm1	2.28E-54	1.112179	0.316	0.02	3.81E-50	4
Arhgef10	5.78E-47	1.111308	0.474	0.053	9.68E-43	4
Tspan3	8.43E-17	1.108921	0.702	0.306	1.41E-12	4
Mthfd2l	1.81E-24	1.102575	0.526	0.122	3.03E-20	4
Gal3st1	1.76E-251	1.101436	0.491	0.009	2.95E-247	4
Pura	2.48E-12	1.096543	0.789	0.514	4.15E-08	4
Ppp1r9a	2.14E-156	1.093855	0.526	0.018	3.57E-152	4
Kras	4.56E-10	1.087927	0.789	0.603	7.64E-06	4
Lpar1	8.79E-22	1.086156	0.474	0.105	1.47E-17	4
Acyp2	4.39E-38	1.085308	0.544	0.088	7.35E-34	4
Map4	6.45E-09	1.079865	0.614	0.386	########	4
Ecscr	4.72E-21	1.078849	0.439	0.089	7.90E-17	4
Gas7	1.55E-07	1.074242	0.579	0.376	########	4
Evi5	3.08E-21	1.071774	0.702	0.258	5.15E-17	4
Cdkn1b	2.40E-08	1.067743	0.789	0.616	########	4
Osbpl1a	2.65E-22	1.067571	0.456	0.1	4.44E-18	4
Cuedc2	3.01E-09	1.066723	0.719	0.556	5.04E-05	4
Fth1	2.80E-07	1.064496	1	0.996	########	4
Zbtb20	8.40E-07	1.054195	0.579	0.331	########	4

Syngr1	1.84E-20	1.052029	0.491	0.119	3.08E-16	4
Snca	0	1.04072	0.439	0.001	0	4
Slc12a2	1.46E-24	1.034381	0.474	0.096	2.45E-20	4
S100a16	9.57E-19	1.034064	0.561	0.147	1.60E-14	4
Rtkn	3.01E-83	1.033355	0.509	0.035	5.04E-79	4
mt-Nd2	3.66E-16	1.033024	0.982	0.892	6.12E-12	4
Angptl4	2.01E-14	1.031908	0.491	0.15	3.37E-10	4
Plpp3	6.45E-33	1.031575	0.667	0.132	1.08E-28	4
Cyp2j6	2.99E-67	1.029774	0.579	0.057	5.00E-63	4
Fbln2	1.61E-12	1.029451	0.456	0.131	2.70E-08	4
Phldb1	7.76E-35	1.028648	0.561	0.099	1.30E-30	4
Eid1	1.67E-09	1.023695	0.772	0.634	2.80E-05	4
Mapk3	3.81E-09	1.020331	0.719	0.55	6.38E-05	4
Snta1	2.69E-32	1.020295	0.561	0.106	4.51E-28	4
Nfasc	7.86E-182	1.020106	0.474	0.012	1.32E-177	4
Ndfip2	5.48E-13	1.014456	0.702	0.401	9.16E-09	4
Sptan1	7.10E-11	1.00912	0.754	0.492	1.19E-06	4
Tm7sf3	6.43E-16	1.009004	0.596	0.232	1.08E-11	4
Bsg	4.51E-13	1.006702	0.86	0.706	7.54E-09	4
Pigp	7.95E-17	1.002158	0.649	0.264	1.33E-12	4
Prss23	4.46E-16	########	0.386	0.088	7.46E-12	4
Cav1	6.58E-27	########	0.667	0.156	1.10E-22	4
Kif21a	3.91E-32	########	0.421	0.06	6.55E-28	4
Otud7b	7.37E-15	########	0.491	0.159	1.23E-10	4
Pros1	1.91E-10	########	0.509	0.217	3.19E-06	4
Aspa	8.36E-164	########	0.439	0.012	1.40E-159	4
Purb	1.74E-06	########	0.807	0.71	########	4
Slit2	1.36E-65	########	0.386	0.025	2.28E-61	4
Itgav	1.98E-16	########	0.754	0.335	3.31E-12	4
Sema6d	1.74E-33	########	0.404	0.053	2.92E-29	4
Syt11	1.40E-39	########	0.544	0.081	2.35E-35	4
Kif1b	3.78E-12	########	0.684	0.398	6.33E-08	4
Ddah1	8.67E-25	########	0.368	0.058	1.45E-20	4
1810037I17	5.01E-10	########	0.877	0.767	8.39E-06	4
Chpt1	7.48E-08	########	0.439	0.209	########	4
Slc25a4	2.62E-13	########	0.842	0.518	4.38E-09	4
Apc	1.69E-09	########	0.596	0.334	2.83E-05	4
Sult1a1	3.95E-36	########	0.386	0.045	6.61E-32	4
Atp8a1	1.20E-07	########	0.596	0.375	########	4
Magi2	8.87E-62	########	0.456	0.037	1.48E-57	4
mt-Nd3	1.80E-12	########	0.965	0.846	3.02E-08	4
Fryl	1.54E-06	########	0.561	0.331	########	4
Bicd1	3.77E-155	########	0.439	0.012	6.31E-151	4
Lama4	4.68E-24	########	0.491	0.1	7.84E-20	4
Itgb4	1.31E-59	########	0.456	0.037	2.18E-55	4
Ywhaq	6.67E-11	########	0.789	0.635	1.12E-06	4
Prex2	5.05E-80	########	0.491	0.032	8.45E-76	4
Ppp1r14c	8.72E-74	########	0.439	0.027	1.46E-69	4
Slc3a2	1.71E-06	########	0.789	0.591	########	4
Hmgn1	1.43E-16	########	0.842	0.412	2.39E-12	4
Thra	2.66E-15	########	0.561	0.196	4.46E-11	4
Ybx1	2.11E-12	########	0.947	0.911	3.54E-08	4

Plekha1	2.23E-14	########	0.561	0.224	3.72E-10	4
Cav2	2.04E-22	########	0.579	0.159	3.41E-18	4
Mocs2	5.80E-15	########	0.702	0.361	9.71E-11	4
Ppm1l	4.22E-61	########	0.456	0.037	7.06E-57	4
Prkca	4.98E-16	########	0.474	0.138	8.34E-12	4
Adam10	6.49E-12	########	0.789	0.525	1.09E-07	4
Clic4	1.00E-12	########	0.772	0.417	1.67E-08	4
Ptov1	1.15E-14	########	0.667	0.296	1.92E-10	4
Arid5b	2.59E-08	########	0.632	0.356	########	4
Gamt	1.44E-13	########	0.509	0.188	2.41E-09	4
Arl3	7.09E-20	########	0.719	0.28	1.19E-15	4
Adam23	9.37E-81	########	0.298	0.011	1.57E-76	4
Slc7a2	1.36E-16	########	0.298	0.055	2.27E-12	4
Fez1	1.89E-72	########	0.474	0.034	3.16E-68	4
Paqr6	1.43E-176	########	0.368	0.007	2.39E-172	4
Tsc22d4	2.57E-07	########	0.842	0.758	########	4
Ubb	3.10E-16	########	1	0.984	5.19E-12	4
Flnb	3.99E-18	########	0.491	0.137	6.68E-14	4
Plxdc2	5.41E-26	########	0.509	0.098	9.05E-22	4
Kcnk1	2.61E-116	########	0.316	0.008	4.37E-112	4
Metrn	3.68E-14	########	0.491	0.168	6.15E-10	4
mt-Nd1	2.09E-15	########	0.982	0.906	3.49E-11	4
Tenm3	3.15E-61	########	0.439	0.035	5.28E-57	4
Mvb12b	4.57E-11	########	0.509	0.219	7.65E-07	4
Jund	2.74E-07	########	0.947	0.894	########	4
Ift43	1.37E-29	########	0.596	0.132	2.28E-25	4
Vamp5	1.22E-07	########	0.509	0.264	########	4
Sox2	0	########	0.421	0.001	0	4
Atp1b3	4.60E-08	########	0.842	0.712	########	4
Arhgef26	6.76E-207	########	0.474	0.01	1.13E-202	4
Bok	1.24E-15	########	0.386	0.093	2.08E-11	4
Gprc5b	4.50E-34	########	0.439	0.061	7.53E-30	4
Sh3d19	3.54E-38	########	0.561	0.092	5.92E-34	4
Sox4	5.29E-11	########	0.421	0.138	8.86E-07	4
Elovl1	1.61E-11	########	0.684	0.405	2.69E-07	4
Fam198b	1.67E-20	########	0.404	0.082	2.79E-16	4
Endod1	4.90E-16	########	0.491	0.151	8.20E-12	4
Mmp15	3.79E-123	########	0.456	0.018	6.35E-119	4
Lysmd2	3.22E-35	########	0.526	0.087	5.39E-31	4
Selk	2.40E-10	########	0.912	0.853	4.01E-06	4
Idi1	4.14E-07	########	0.456	0.219	########	4
Sorcs1	0	########	0.421	0.001	0	4
Ryk	2.20E-28	########	0.614	0.135	3.68E-24	4
Ptrf	1.33E-28	########	0.702	0.159	2.23E-24	4
Frmd4a	1.62E-09	########	0.439	0.178	2.71E-05	4
Papss1	9.28E-13	########	0.579	0.251	1.55E-08	4
Adgrg6	7.01E-72	########	0.526	0.042	1.17E-67	4
Casc4	1.90E-29	########	0.474	0.081	3.18E-25	4
Itpr3	7.11E-17	########	0.421	0.102	1.19E-12	4
Anxa5	1.36E-06	########	0.842	0.681	########	4
Ank	1.39E-22	########	0.491	0.111	2.32E-18	4
Dlc1	1.17E-20	########	0.456	0.101	1.96E-16	4

Itgb1	1.00E-12	########	0.93	0.649	1.68E-08	4
Nid1	2.28E-29	########	0.596	0.116	3.81E-25	4
Nek1	3.16E-18	########	0.404	0.09	5.30E-14	4
Ngfr	2.23E-138	########	0.263	0.004	3.73E-134	4
Npdc1	2.09E-23	########	0.561	0.128	3.49E-19	4
Nedd4	6.04E-13	########	0.702	0.302	1.01E-08	4
Tns3	6.39E-13	########	0.614	0.268	1.07E-08	4
Serinc1	4.97E-10	########	0.789	0.559	8.32E-06	4
Efhd1	1.86E-168	########	0.386	0.008	3.11E-164	4
Ndufa13	1.76E-07	########	0.877	0.882	########	4
Tmem176a	8.26E-14	########	0.684	0.27	1.38E-09	4
Arhgef12	2.24E-22	########	0.632	0.18	3.75E-18	4
Arl5a	5.38E-09	########	0.561	0.287	9.00E-05	4
Lhfp	1.25E-34	########	0.596	0.101	2.09E-30	4
Srp14	1.05E-09	########	0.86	0.791	1.76E-05	4
Hsbp1	2.80E-11	########	0.86	0.662	4.68E-07	4
Snx7	3.29E-25	########	0.544	0.125	5.50E-21	4
Nudt3	7.07E-08	########	0.544	0.336	########	4
Il6st	4.79E-08	########	0.579	0.291	########	4
Cers2	2.03E-08	########	0.702	0.543	########	4
Cttn	2.75E-23	########	0.526	0.123	4.61E-19	4
Bche	1.51E-71	########	0.123	0.002	2.53E-67	4
Tmem47	5.07E-28	########	0.386	0.056	8.49E-24	4
Wwtr1	2.72E-24	########	0.579	0.13	4.55E-20	4
Eif1	8.20E-12	########	1	0.99	1.37E-07	4
Capg	3.13E-07	########	0.825	0.673	########	4
Tax1bp3	1.12E-07	########	0.561	0.325	########	4
Sh3pxd2a	1.18E-12	########	0.526	0.192	1.98E-08	4
Mprip	1.11E-09	########	0.632	0.326	1.86E-05	4
Rab34	5.31E-29	########	0.561	0.115	8.88E-25	4
Dad1	2.21E-08	########	0.912	0.737	########	4
Fscn1	6.48E-26	########	0.509	0.102	1.08E-21	4
Pex5l	0	########	0.421	0.001	0	4
Zfp536	0	########	0.474	0.001	0	4
Bpgm	3.46E-09	########	0.298	0.088	5.78E-05	4
Lap3	1.83E-10	########	0.561	0.278	3.06E-06	4
Ywhae	7.13E-11	########	0.877	0.785	1.19E-06	4
Higd2a	7.24E-07	########	0.737	0.629	########	4
Pdlim7	2.01E-07	########	0.526	0.295	########	4
Plec	4.19E-08	########	0.754	0.62	########	4
Fnta	1.43E-06	########	0.561	0.377	########	4
Nov	6.86E-84	########	0.386	0.019	1.15E-79	4
Csmd1	2.18E-97	########	0.263	0.007	3.65E-93	4
Nhp2l1	1.02E-08	########	0.789	0.595	########	4
Usp6nl	9.32E-11	########	0.368	0.117	1.56E-06	4
Odc1	1.46E-13	########	0.807	0.412	2.45E-09	4
Pttg1ip	1.30E-10	########	0.719	0.428	2.18E-06	4
Nckap1	3.58E-18	########	0.491	0.129	6.00E-14	4
Dab2ip	1.62E-16	########	0.509	0.156	2.72E-12	4
Ado	1.74E-07	########	0.439	0.215	########	4
Atp1b2	1.91E-33	########	0.211	0.014	3.19E-29	4
Ptpdc1	3.88E-52	########	0.333	0.023	6.49E-48	4

Ndrg2	2.15E-23	########	0.351	0.056	3.60E-19	4
Hsp90ab1	8.73E-11	########	0.947	0.917	1.46E-06	4
Csnk1g3	9.56E-08	########	0.579	0.336	########	4
Syt4	1.18E-168	########	0.193	0.001	1.98E-164	4
Ggct	3.00E-10	########	0.474	0.19	5.02E-06	4
Psmd7	9.20E-09	########	0.737	0.504	########	4
3-Sep	1.75E-207	########	0.351	0.005	2.93E-203	4
Uchl1	3.68E-32	########	0.351	0.041	6.16E-28	4
Mtch1	2.36E-09	########	0.754	0.593	3.94E-05	4
Ptprs	2.66E-22	########	0.579	0.158	4.45E-18	4
Emid1	9.07E-25	########	0.228	0.023	1.52E-20	4
Nudt9	4.71E-13	########	0.579	0.258	7.88E-09	4
Atraid	6.12E-11	########	0.737	0.48	1.02E-06	4
Rnd3	1.55E-08	########	0.474	0.209	########	4
Col12a1	2.13E-15	########	0.386	0.088	3.56E-11	4
Tmem205	2.45E-06	########	0.509	0.314	########	4
Fgf1	6.06E-84	########	0.281	0.01	1.01E-79	4
Hist1h2bc	5.90E-09	########	0.649	0.369	9.88E-05	4
Pgrmc1	2.19E-16	########	0.737	0.326	3.67E-12	4
Srgap2	7.28E-07	########	0.579	0.354	########	4
1810011O1	2.14E-11	########	0.333	0.088	3.59E-07	4
Cfap100	0	########	0.298	0.001	0	4
Fam19a5	9.97E-130	########	0.351	0.009	1.67E-125	4
Nfia	5.77E-11	########	0.474	0.174	9.65E-07	4
Tprgl	1.70E-06	########	0.719	0.557	########	4
Scrn1	1.89E-135	########	0.421	0.013	3.16E-131	4
Nrcam	1.01E-206	########	0.368	0.006	1.68E-202	4
Csnk1a1	8.40E-09	########	0.912	0.78	########	4
Hdhd2	7.41E-09	########	0.456	0.206	########	4
Mgll	2.27E-21	########	0.526	0.126	3.80E-17	4
Mmgt2	2.53E-14	########	0.439	0.13	4.23E-10	4
Nrn1	5.65E-09	########	0.175	0.035	9.45E-05	4
Fyn	3.05E-10	########	0.596	0.267	5.11E-06	4
Tpm1	4.79E-09	########	0.561	0.284	8.01E-05	4
Ttc3	1.85E-14	########	0.596	0.226	3.09E-10	4
Gpc1	3.31E-12	########	0.404	0.13	5.54E-08	4
Deptor	2.93E-16	########	0.456	0.128	4.90E-12	4
Gabarapl1	1.08E-07	########	0.491	0.235	########	4
Laptm4a	1.29E-07	########	0.772	0.643	########	4
Mbnl2	5.99E-08	########	0.807	0.622	########	4
Stard13	5.67E-41	########	0.404	0.043	9.48E-37	4
Mapre3	9.34E-63	########	0.474	0.039	1.56E-58	4
Mpdz	2.77E-23	########	0.421	0.078	4.64E-19	4
Clcn3	5.29E-09	########	0.526	0.255	8.86E-05	4
Epb41l4b	2.24E-76	########	0.368	0.019	3.75E-72	4
Hsp90aa1	4.90E-07	########	0.895	0.807	########	4
Acadl	1.05E-06	########	0.667	0.494	########	4
Snx16	2.40E-12	########	0.368	0.102	4.02E-08	4
Tgfbr3	3.28E-22	########	0.561	0.132	5.50E-18	4
Ttc9	1.36E-117	########	0.421	0.016	2.28E-113	4
Rap1gap	3.51E-144	########	0.281	0.005	5.87E-140	4
Rabac1	1.22E-07	########	0.825	0.828	########	4

Nacc2	1.64E-11	########	0.386	0.12	2.74E-07	4
Rasgef1c	0	########	0.333	0.001	0	4
Calu	7.04E-10	########	0.684	0.372	1.18E-05	4
Utp11l	3.11E-08	########	0.649	0.418	########	4
Uqcc2	7.89E-08	########	0.772	0.538	########	4
Wasl	4.71E-12	########	0.561	0.24	7.88E-08	4
Olfml2b	2.94E-12	########	0.491	0.169	4.92E-08	4
Fbln5	5.04E-07	########	0.246	0.076	########	4
Amotl1	2.17E-16	########	0.404	0.097	3.63E-12	4
Stxbp6	9.38E-13	########	0.404	0.122	1.57E-08	4
Plxnb3	5.90E-274	########	0.333	0.003	9.87E-270	4
Spire1	1.82E-15	########	0.421	0.112	3.04E-11	4
Pmm1	7.81E-13	########	0.526	0.21	1.31E-08	4
Vat1l	0	########	0.404	0.002	0	4
2700060E0	9.44E-07	########	0.772	0.687	########	4
Gnas	4.45E-10	########	0.965	0.882	7.44E-06	4
Filip1	1.72E-39	########	0.351	0.033	2.88E-35	4
Nrbp2	4.39E-35	########	0.281	0.024	7.35E-31	4
Arl2	1.11E-08	########	0.439	0.191	########	4
Cdc37l1	2.55E-08	########	0.491	0.23	########	4
Josd2	9.76E-07	########	0.491	0.285	########	4
Asrgl1	9.03E-12	########	0.456	0.161	1.51E-07	4
Entpd2	1.30E-06	########	0.193	0.053	########	4
Myo9a	2.47E-06	########	0.456	0.225	########	4
Hspa2	1.08E-19	########	0.491	0.12	1.80E-15	4
Ccdc47	1.87E-09	########	0.719	0.447	3.12E-05	4
Agpat3	2.96E-08	########	0.491	0.239	########	4
Ctnnb1	1.94E-08	########	0.825	0.615	########	4
C4b	7.11E-09	########	0.281	0.079	########	4
Cd99l2	3.73E-14	########	0.351	0.085	6.23E-10	4
Ahnak2	4.25E-20	########	0.404	0.084	7.12E-16	4
Slc9a3r2	1.18E-14	########	0.316	0.067	1.98E-10	4
Spop	8.26E-07	########	0.789	0.65	########	4
Abca8a	1.91E-14	########	0.158	0.019	3.19E-10	4
2010111I0	9.01E-09	########	0.544	0.265	########	4
mt-Nd4	1.01E-09	########	0.982	0.961	1.69E-05	4
Abhd4	1.13E-20	########	0.491	0.117	1.89E-16	4
Fat1	1.41E-20	########	0.386	0.075	2.36E-16	4
Dctn2	1.26E-08	########	0.719	0.574	########	4
Myo1b	6.01E-14	########	0.421	0.12	1.01E-09	4
Cd200	1.71E-13	########	0.368	0.089	2.87E-09	4
L1cam	1.26E-10	########	0.298	0.078	2.11E-06	4
Ucn2	2.48E-119	########	0.175	0.002	4.15E-115	4
1110065P2	1.46E-11	########	0.561	0.241	2.45E-07	4
Sgce	1.54E-40	########	0.491	0.065	2.58E-36	4
mt-Nd4l	3.50E-10	########	0.912	0.75	5.85E-06	4
Syne2	3.53E-11	########	0.561	0.243	5.91E-07	4
Abca8b	6.11E-20	########	0.158	0.014	1.02E-15	4
Grb14	1.86E-29	########	0.281	0.029	3.11E-25	4
Tanc2	1.23E-18	########	0.439	0.103	2.06E-14	4
Git1	2.84E-15	########	0.509	0.164	4.76E-11	4
Tmem245	3.55E-11	########	0.474	0.173	5.93E-07	4

Bod1	1.03E-13	########	0.509	0.177	1.72E-09	4
Mrfap1	2.23E-06	########	0.772	0.702	########	4
Fgf5	6.52E-248	########	0.158	0	1.09E-243	4
Kif19a	3.26E-99	########	0.333	0.011	5.45E-95	4
Kif3a	1.48E-09	########	0.404	0.145	2.47E-05	4
P2rx7	7.57E-13	########	0.439	0.132	1.27E-08	4
Akap12	2.33E-12	########	0.333	0.084	3.91E-08	4
Timp2	3.01E-10	########	0.912	0.549	5.03E-06	4
Prrg3	3.50E-41	########	0.298	0.023	5.86E-37	4
Slc6a8	2.38E-06	########	0.368	0.162	########	4
Vamp2	1.07E-11	########	0.544	0.22	1.79E-07	4
Uaca	3.38E-16	########	0.368	0.083	5.66E-12	4
D430041D(	0	########	0.351	0.001	0	4
Cyp2j9	5.91E-126	########	0.316	0.008	9.89E-122	4
Prss12	4.04E-106	########	0.386	0.014	6.77E-102	4
Epdr1	1.40E-33	########	0.439	0.062	2.35E-29	4
Snhg18	1.84E-09	########	0.333	0.099	3.08E-05	4
Rasl11b	1.10E-13	########	0.263	0.052	1.84E-09	4
Me1	1.10E-21	########	0.351	0.06	1.84E-17	4
Mme	8.12E-50	########	0.246	0.013	1.36E-45	4
Nfib	1.04E-16	########	0.561	0.149	1.73E-12	4
Nav3	1.27E-37	########	0.351	0.036	2.12E-33	4
Fam107a	1.03E-152	########	0.263	0.004	1.73E-148	4
Adgrl3	1.63E-120	########	0.351	0.01	2.73E-116	4
Spg20	1.52E-10	########	0.386	0.125	2.54E-06	4
Cyp51	7.70E-07	########	0.439	0.207	########	4
Dock1	1.53E-07	########	0.421	0.197	########	4
Pink1	9.02E-07	########	0.509	0.278	########	4
Tmbim1	3.43E-10	########	0.596	0.29	5.74E-06	4
Cldnd1	1.00E-08	########	0.579	0.322	########	4
Rcn1	1.58E-14	########	0.544	0.172	2.65E-10	4
Cdh1	1.11E-28	########	0.316	0.036	1.86E-24	4
Tmem176b	7.07E-10	########	0.684	0.299	1.18E-05	4
Aif1l	7.22E-115	########	0.368	0.012	1.21E-110	4
Atp6v0e2	2.66E-171	########	0.333	0.006	4.45E-167	4
Sgcb	1.04E-10	########	0.404	0.137	1.75E-06	4
Itga7	1.99E-40	########	0.333	0.03	3.32E-36	4
Vkorc1	1.77E-06	########	0.526	0.275	########	4
Arhgap5	2.85E-08	########	0.456	0.2	########	4
Ndufa7	2.28E-06	########	0.895	0.842	########	4
Frmd6	2.68E-08	########	0.368	0.136	########	4
Gabarapl2	1.87E-06	########	0.772	0.706	########	4
Ndufb4	1.92E-06	########	0.737	0.715	########	4
Tsen34	1.04E-07	########	0.579	0.347	########	4
Emc7	5.82E-07	########	0.737	0.588	########	4
Plin3	4.93E-07	########	0.614	0.379	########	4
Sptb	1.50E-49	########	0.158	0.005	2.51E-45	4
Ahnak	6.91E-07	########	0.947	0.636	########	4
St6gal1	1.45E-09	########	0.386	0.133	2.43E-05	4
Myo18a	4.23E-10	########	0.509	0.218	7.08E-06	4
Maged1	6.58E-15	########	0.526	0.153	1.10E-10	4
Kcnmb4	3.32E-51	########	0.281	0.016	5.55E-47	4

Plpp1	5.45E-16	########	0.439	0.111	9.11E-12	4
Ahi1	5.42E-09	########	0.368	0.13	9.07E-05	4
Fads1	2.60E-11	########	0.404	0.128	4.34E-07	4
Ak3	4.50E-13	########	0.509	0.184	7.53E-09	4
Hmgcs2	2.48E-62	########	0.263	0.012	4.16E-58	4
Aebp1	9.89E-07	########	0.333	0.119	########	4
Gm42418	2.05E-06	########	1	1	########	4
Fmnl2	1.23E-13	########	0.474	0.151	2.06E-09	4
Gstm5	6.66E-12	########	0.439	0.143	1.11E-07	4
Rab6b	1.66E-10	########	0.263	0.062	2.77E-06	4
Arhgef40	1.47E-18	########	0.368	0.076	2.47E-14	4
Kctd11	1.54E-11	########	0.316	0.083	2.57E-07	4
Mboat2	3.18E-118	########	0.351	0.01	5.31E-114	4
Lamc1	4.10E-12	########	0.509	0.179	6.86E-08	4
Pgp	2.54E-06	########	0.649	0.442	########	4
Cfap36	6.77E-10	########	0.561	0.271	1.13E-05	4
Dock7	3.36E-07	########	0.421	0.195	########	4
Spon2	7.83E-25	########	0.193	0.016	1.31E-20	4
Cetn3	1.78E-06	########	0.649	0.519	########	4
Ppp2r3a	5.07E-08	########	0.298	0.096	########	4
Trim2	3.15E-07	########	0.228	0.066	########	4
mt-Atp6	1.91E-10	########	1	0.989	3.20E-06	4
Alg14	4.13E-08	########	0.404	0.165	########	4
Adamts5	2.18E-15	########	0.386	0.087	3.66E-11	4
Psmb6	1.38E-06	########	0.86	0.713	########	4
Tmem229a	2.68E-254	########	0.211	0.001	4.49E-250	4
Smco3	0	########	0.263	0.001	0	4
Swi5	5.76E-07	########	0.807	0.693	########	4
Nudc	3.35E-07	########	0.719	0.538	########	4
Elovl6	1.95E-10	########	0.333	0.098	3.26E-06	4
Lzts2	1.81E-15	########	0.368	0.086	3.03E-11	4
Ktn1	1.72E-07	########	0.614	0.361	########	4
Bcl7c	1.87E-06	########	0.667	0.432	########	4
Ddah2	3.90E-11	########	0.456	0.153	6.53E-07	4
Comt	1.84E-06	########	0.632	0.423	########	4
Sgcd	2.30E-50	########	0.351	0.027	3.85E-46	4
Tmem106b	8.54E-08	########	0.544	0.299	########	4
Tmem59	1.34E-06	########	0.772	0.765	########	4
Serf1	7.98E-09	########	0.404	0.158	########	4
Mast2	1.67E-12	########	0.421	0.136	2.80E-08	4
Lin7c	1.75E-06	########	0.596	0.375	########	4
Rap1gds1	2.15E-06	########	0.579	0.358	########	4
Insc	1.54E-293	########	0.386	0.004	2.57E-289	4
Ptk2	1.60E-15	########	0.386	0.094	2.68E-11	4
Ptpn13	5.26E-28	########	0.368	0.052	8.80E-24	4
Gpr155	2.48E-13	########	0.246	0.046	4.15E-09	4
Sorcs2	3.90E-11	########	0.246	0.054	6.53E-07	4
Sec62	1.23E-07	########	0.895	0.765	########	4
Glo1	1.69E-06	########	0.579	0.366	########	4
Amfr	1.51E-06	########	0.614	0.422	########	4
Copz2	8.11E-13	########	0.456	0.132	1.36E-08	4
Ctnna3	7.76E-152	########	0.246	0.003	1.30E-147	4

Plscr4	3.75E-23	########	0.333	0.05	6.27E-19	4
Abhd14a	1.99E-12	########	0.439	0.146	3.34E-08	4
Hepacam	0	########	0.211	0	0	4
Sccpdh	4.29E-18	########	0.386	0.083	7.18E-14	4
Nipal1	2.93E-22	########	0.246	0.029	4.90E-18	4
Tnxb	1.90E-13	########	0.281	0.056	3.18E-09	4
Ak5	2.80E-132	########	0.316	0.007	4.69E-128	4
Zfp651	4.65E-13	########	0.333	0.081	7.78E-09	4
Serpinh1	9.93E-27	########	0.754	0.169	1.66E-22	4
Parva	3.17E-14	########	0.439	0.121	5.30E-10	4
Fmn2	5.56E-156	########	0.263	0.004	9.30E-152	4
Lpin1	2.00E-10	########	0.298	0.078	3.34E-06	4
Rtn4	2.09E-06	########	0.825	0.749	########	4
9530059O1	1.82E-119	########	0.228	0.004	3.05E-115	4
Disp1	5.68E-23	########	0.333	0.05	9.51E-19	4
Nf1	1.99E-07	########	0.439	0.205	########	4
Ank2	5.62E-20	########	0.333	0.057	9.40E-16	4
Tulp4	6.98E-07	########	0.561	0.341	########	4
Map6	1.50E-35	########	0.368	0.042	2.51E-31	4
Plxnb1	2.10E-22	########	0.193	0.018	3.51E-18	4
Cisd1	1.56E-07	########	0.579	0.334	########	4
Cuedc1	5.24E-19	########	0.386	0.077	8.77E-15	4
Angptl2	7.79E-17	########	0.386	0.085	1.30E-12	4
Maged2	1.04E-12	########	0.439	0.129	1.73E-08	4
Tmem237	2.13E-20	########	0.421	0.089	3.57E-16	4
Zfp462	1.09E-21	########	0.298	0.043	1.82E-17	4
Ndnf	6.22E-11	########	0.14	0.02	1.04E-06	4
Nop16	9.66E-07	########	0.456	0.217	########	4
Zdhhc8	7.02E-09	########	0.333	0.111	########	4
Jkamp	3.55E-07	########	0.526	0.281	########	4
Col5a2	7.43E-24	########	0.596	0.123	1.24E-19	4
Hspa13	4.44E-07	########	0.474	0.238	########	4
Kank4	3.90E-171	########	0.228	0.002	6.53E-167	4
Sbf2	4.99E-08	########	0.421	0.174	########	4
Shroom2	2.27E-46	########	0.246	0.014	3.79E-42	4
Ankrd13b	5.27E-34	########	0.316	0.032	8.82E-30	4
Slc36a2	4.62E-110	########	0.246	0.005	7.73E-106	4
Galnt16	2.35E-18	########	0.263	0.04	3.93E-14	4
Hrsp12	7.04E-10	########	0.333	0.103	1.18E-05	4
Nr2f2	5.54E-22	########	0.421	0.078	9.27E-18	4
Drap1	1.47E-06	########	0.807	0.701	########	4
Pdzd2	1.16E-13	########	0.263	0.051	1.94E-09	4
Ptprz1	6.43E-19	########	0.14	0.011	1.08E-14	4
Pex2	2.38E-09	########	0.579	0.294	3.99E-05	4
Sema3g	2.15E-80	########	0.263	0.009	3.59E-76	4
Hmgn3	1.33E-10	########	0.439	0.153	2.23E-06	4
Arhgap24	3.80E-08	########	0.439	0.189	########	4
Ddhd1	8.77E-07	########	0.404	0.178	########	4
Dnaja4	5.43E-33	########	0.368	0.044	9.08E-29	4
Adcy1	5.11E-110	########	0.228	0.004	8.55E-106	4
Dnajb2	6.54E-15	########	0.404	0.103	1.09E-10	4
Igsf11	0	########	0.263	0	0	4

Nptx2	5.53E-104	########	0.211	0.004	9.25E-100	4
Polr3h	1.68E-07	########	0.316	0.116	########	4
Frzb	3.14E-209	########	0.228	0.002	5.26E-205	4
Mical3	2.45E-13	########	0.333	0.08	4.10E-09	4
Spry2	8.20E-09	########	0.368	0.128	########	4
Lama2	8.63E-19	########	0.281	0.043	1.44E-14	4
Pxdc1	1.30E-14	########	0.404	0.102	2.18E-10	4
Lrrc75a	3.48E-25	########	0.298	0.038	5.82E-21	4
Yae1d1	9.75E-09	########	0.386	0.144	########	4
Fxyd7	6.40E-23	########	0.105	0.005	1.07E-18	4
Sema4f	8.17E-11	########	0.14	0.02	1.37E-06	4
Chrdl1	2.11E-17	########	0.228	0.031	3.53E-13	4
Cry2	3.14E-07	########	0.281	0.096	########	4
Nkain2	0	########	0.333	0.001	0	4
Hmgb1	2.38E-07	########	0.947	0.88	########	4
Crabp2	1.52E-09	########	0.193	0.039	2.55E-05	4
Btc	1.85E-85	########	0.193	0.004	3.10E-81	4
Abca2	3.23E-10	########	0.298	0.08	5.40E-06	4
Ccdc13	9.72E-129	########	0.175	0.002	1.63E-124	4
Nfix	6.08E-07	########	0.579	0.3	########	4
Camk2b	2.03E-24	########	0.211	0.019	3.40E-20	4
Sorbs1	5.11E-72	########	0.351	0.018	8.55E-68	4
Akap11	2.41E-08	########	0.439	0.19	########	4
Fbxo7	8.26E-12	########	0.404	0.121	1.38E-07	4
Tcaf1	8.62E-11	########	0.316	0.084	1.44E-06	4
Plat	1.02E-10	########	0.316	0.085	1.71E-06	4
Zfp664	1.10E-07	########	0.421	0.177	########	4
Gid4	5.18E-07	########	0.386	0.164	########	4
Smap1	1.24E-06	########	0.754	0.605	########	4
Zcchc24	1.86E-07	########	0.456	0.208	########	4
Myo6	8.03E-16	########	0.351	0.077	1.34E-11	4
Dbn1	2.47E-16	########	0.351	0.075	4.14E-12	4
Fzd3	1.95E-64	########	0.263	0.011	3.26E-60	4
Arhgef17	3.14E-23	########	0.298	0.04	5.25E-19	4
Tom1l2	3.51E-16	########	0.333	0.069	5.87E-12	4
B4gat1	7.01E-07	########	0.333	0.134	########	4
Slc27a1	2.46E-07	########	0.298	0.103	########	4
Caskin2	1.20E-10	########	0.228	0.048	2.00E-06	4
Asap2	3.42E-09	########	0.298	0.089	5.72E-05	4
Kif3c	7.25E-43	########	0.298	0.022	1.21E-38	4
Zc2hc1a	7.56E-09	########	0.263	0.073	########	4
Acsl3	1.08E-07	########	0.368	0.147	########	4
Afap1l2	8.22E-48	########	0.263	0.016	1.38E-43	4
Ttyh1	9.81E-116	########	0.281	0.007	1.64E-111	4
Ptch1	2.81E-11	########	0.281	0.066	4.71E-07	4
Pcdh9	5.15E-69	########	0.246	0.009	8.61E-65	4
Wtip	3.06E-08	########	0.316	0.104	########	4
Tjp2	4.42E-11	########	0.368	0.111	7.40E-07	4
Tmcc3	2.47E-10	########	0.333	0.097	4.14E-06	4
Mmp17	2.40E-119	########	0.298	0.007	4.01E-115	4
Fkbp9	1.38E-16	########	0.526	0.142	2.32E-12	4
Pithd1	1.30E-07	########	0.421	0.186	########	4

Grik2	0	########	0.246	0	0	4
Ephb6	2.02E-22	########	0.193	0.018	3.39E-18	4
Chadl	1.61E-117	########	0.333	0.009	2.70E-113	4
Sema6c	2.31E-26	########	0.175	0.013	3.87E-22	4
Chp2	1.01E-10	########	0.158	0.025	1.69E-06	4
Maml3	5.55E-08	########	0.421	0.169	########	4
1300002E1	5.88E-07	########	0.333	0.131	########	4
Arl13b	1.43E-06	########	0.456	0.224	########	4
Hdgfrp3	3.03E-09	########	0.316	0.097	5.07E-05	4
Fads3	8.38E-13	########	0.368	0.097	1.40E-08	4
Camsap2	2.38E-07	########	0.386	0.161	########	4
Hadh	3.94E-07	########	0.526	0.277	########	4
Il34	9.38E-56	########	0.298	0.017	1.57E-51	4
Gpd1	1.87E-60	########	0.228	0.009	3.13E-56	4
Gm12688	0	########	0.263	0.001	0	4
Dusp18	4.92E-12	########	0.298	0.07	8.23E-08	4
Shisa4	5.05E-27	########	0.316	0.04	8.45E-23	4
Rimklb	2.21E-280	########	0.175	0	3.70E-276	4
Tln2	4.22E-07	########	0.298	0.102	########	4
Gkap1	5.65E-07	########	0.333	0.13	########	4
Ddr1	3.64E-29	########	0.386	0.053	6.10E-25	4
Cyp7b1	1.13E-19	########	0.281	0.042	1.89E-15	4
Mxra7	9.85E-15	########	0.421	0.107	1.65E-10	4
Ntm	2.19E-58	########	0.158	0.004	3.66E-54	4
Rdh5	3.25E-23	########	0.298	0.041	5.44E-19	4
Coprs	1.44E-12	########	0.351	0.091	2.40E-08	4
Sphk1	8.06E-07	########	0.281	0.096	########	4
Epn2	4.27E-09	########	0.298	0.088	7.14E-05	4
Fdft1	7.07E-07	########	0.333	0.137	########	4
Pam	1.11E-06	########	0.526	0.285	########	4
Dgcr6	5.55E-07	########	0.491	0.259	########	4
Tceal8	2.69E-06	########	0.526	0.272	########	4
Shroom3	8.53E-58	0.431971	0.281	0.014	1.43E-53	4
Tusc3	2.08E-06	########	0.421	0.209	########	4
Cmbl	9.43E-30	########	0.298	0.032	1.58E-25	4
Nid2	4.37E-12	########	0.298	0.07	7.31E-08	4
A330069K(	0	########	0.193	0	0	4
Rftn2	3.98E-16	########	0.298	0.056	6.66E-12	4
Shpk	4.81E-37	########	0.228	0.015	8.04E-33	4
Lrrc8b	1.37E-10	########	0.298	0.078	2.30E-06	4
Cyp39a1	7.75E-09	########	0.211	0.049	########	4
Osbpl5	7.12E-12	########	0.263	0.057	1.19E-07	4
Gna11	1.96E-06	########	0.421	0.202	########	4
B230118H(	1.10E-08	########	0.421	0.171	########	4
Clip3	7.32E-34	########	0.333	0.035	1.23E-29	4
Kcna6	6.27E-251	########	0.263	0.002	1.05E-246	4
Cdc42bpa	2.03E-12	########	0.386	0.107	3.39E-08	4
Btbd3	1.37E-10	########	0.298	0.078	2.29E-06	4
Ldlrad3	3.74E-07	########	0.298	0.105	########	4
Piezo2	5.54E-34	########	0.281	0.025	9.27E-30	4
Grik5	5.41E-28	########	0.193	0.014	9.04E-24	4
Bcl2l1	4.35E-07	########	0.456	0.211	########	4

Col14a1	2.83E-11	########	0.281	0.064	4.74E-07	4
Arhgap39	1.66E-16	########	0.386	0.086	2.77E-12	4
Dusp8	1.11E-14	########	0.246	0.042	1.87E-10	4
Smyd2	4.43E-08	########	0.368	0.144	########	4
Cers4	1.09E-07	########	0.281	0.089	########	4
Zfyve21	3.85E-08	########	0.351	0.13	########	4
Bmp1	1.31E-06	########	0.281	0.097	########	4
Ncald	6.37E-17	########	0.211	0.028	1.07E-12	4
Bmpr1a	2.52E-11	########	0.386	0.117	4.21E-07	4
Cystm1	7.54E-14	########	0.351	0.081	1.26E-09	4
Samd4	2.02E-12	########	0.316	0.077	3.38E-08	4
Tagln	1.25E-27	########	0.368	0.05	2.10E-23	4
Prima1	5.82E-81	########	0.105	0.001	9.73E-77	4
Mgat3	1.67E-154	########	0.281	0.004	2.79E-150	4
Ptpn21	1.76E-09	########	0.193	0.04	2.94E-05	4
Agrn	1.07E-12	########	0.246	0.048	1.80E-08	4
Spire2	1.99E-79	########	0.158	0.003	3.33E-75	4
Car11	7.85E-65	########	0.175	0.005	1.31E-60	4
Mboat1	3.37E-10	########	0.281	0.072	5.64E-06	4
Slc22a23	5.57E-89	########	0.298	0.01	9.32E-85	4
Ick	1.39E-08	########	0.281	0.082	########	4
Arnt2	4.53E-166	########	0.228	0.002	7.58E-162	4
Gpm6a	2.98E-71	########	0.193	0.005	4.98E-67	4
Stox2	7.98E-11	########	0.246	0.054	1.34E-06	4
Chn2	2.40E-19	########	0.298	0.047	4.02E-15	4
Dlgap1	7.67E-36	########	0.158	0.007	1.28E-31	4
Mcam	5.89E-11	########	0.281	0.065	9.86E-07	4
Evc2	4.44E-15	########	0.228	0.036	7.43E-11	4
Adamtsl5	1.10E-17	########	0.263	0.041	1.84E-13	4
Myl9	2.06E-22	########	0.333	0.049	3.44E-18	4
Fam132a	2.29E-06	########	0.404	0.185	########	4
Lims2	4.73E-24	########	0.263	0.03	7.91E-20	4
Mamld1	2.28E-36	########	0.228	0.015	3.81E-32	4
Crebl2	2.08E-08	########	0.263	0.072	########	4
Prrg4	2.60E-13	########	0.14	0.016	4.36E-09	4
Sox6	9.77E-46	########	0.193	0.009	1.63E-41	4
Tdrkh	3.17E-44	########	0.333	0.027	5.31E-40	4
Ak1	3.68E-08	########	0.263	0.076	########	4
Zcchc14	8.28E-08	########	0.263	0.079	0.001385	4
9430020K0	9.05E-07	########	0.228	0.068	########	4
Ppfibp2	5.10E-09	########	0.298	0.086	8.53E-05	4
Morn2	3.17E-11	########	0.298	0.076	5.31E-07	4
Nradd	1.18E-08	########	0.316	0.104	########	4
Fez2	1.74E-06	########	0.439	0.213	########	4
Map1a	6.83E-09	########	0.228	0.057	########	4
Epha5	8.97E-103	########	0.246	0.006	1.50E-98	4
Cdc42ep1	4.52E-19	########	0.281	0.043	7.57E-15	4
Laptm4b	1.58E-12	########	0.298	0.069	2.65E-08	4
Dmwd	3.42E-09	########	0.263	0.071	5.72E-05	4
Wbscr16	1.07E-07	########	0.263	0.08	########	4
Fgf2	4.00E-16	########	0.228	0.034	6.69E-12	4
Efna5	1.01E-06	########	0.14	0.031	########	4

Gdnf	3.48E-21	########	0.158	0.013	5.83E-17	4
Sorbs3	3.82E-10	########	0.193	0.038	6.40E-06	4
Sema4c	1.42E-08	########	0.228	0.058	########	4
Rnf122	7.89E-08	########	0.211	0.054	########	4
Mageh1	1.74E-14	########	0.316	0.067	2.91E-10	4
Zfhx4	4.09E-15	########	0.316	0.063	6.84E-11	4
Ltbp3	1.07E-07	########	0.281	0.088	########	4
Fign	6.80E-116	########	0.263	0.006	1.14E-111	4
Sorbs2	8.01E-10	########	0.158	0.026	1.34E-05	4
Cpeb1	2.42E-16	########	0.193	0.024	4.05E-12	4
Adamtsl1	7.86E-10	########	0.246	0.058	1.31E-05	4
Hoxb3os	2.12E-35	########	0.158	0.008	3.55E-31	4
Col27a1	1.26E-13	########	0.298	0.062	2.11E-09	4
Col15a1	1.15E-07	########	0.193	0.045	########	4
Arhgap32	2.27E-12	########	0.246	0.049	3.79E-08	4
Dennd2a	4.61E-12	########	0.298	0.072	7.72E-08	4
Gm2115	1.51E-56	########	0.123	0.003	2.52E-52	4
Fam212b	7.95E-33	########	0.193	0.012	1.33E-28	4
Nr2f1	4.07E-56	########	0.246	0.011	6.82E-52	4
Xrcc5	9.87E-10	########	0.298	0.083	1.65E-05	4
Col23a1	8.45E-20	########	0.211	0.024	1.41E-15	4
Slc6a1	4.92E-306	########	0.175	0	8.23E-302	4
Myh14	1.91E-49	########	0.14	0.004	3.19E-45	4
Eml1	1.19E-11	########	0.281	0.066	1.99E-07	4
Ankrd50	5.16E-08	########	0.316	0.107	########	4
Cdh2	4.00E-25	########	0.246	0.026	6.70E-21	4
Ehbp1	1.28E-09	########	0.193	0.039	2.14E-05	4
Rnf125	4.73E-07	########	0.316	0.113	########	4
Atp9a	5.28E-15	########	0.281	0.052	8.84E-11	4
Fgf7	1.73E-07	########	0.246	0.069	########	4
Mxra8	5.99E-07	########	0.316	0.107	########	4
Gm16638	2.32E-10	########	0.158	0.025	3.88E-06	4
2010320M1	2.65E-06	########	0.316	0.126	########	4
Arhgap42	4.87E-12	########	0.281	0.062	8.15E-08	4
Rxrg	2.14E-30	########	0.14	0.007	3.58E-26	4
Trim13	8.53E-14	########	0.281	0.055	1.43E-09	4
Slc22a17	9.74E-18	########	0.175	0.019	1.63E-13	4
Trove2	1.10E-10	########	0.368	0.11	1.85E-06	4
Slc35f1	1.32E-145	########	0.175	0.001	2.21E-141	4
Yap1	7.12E-08	########	0.298	0.096	########	4
Rhbdf1	2.93E-13	########	0.316	0.072	4.90E-09	4
Tnni1	8.90E-18	########	0.105	0.007	1.49E-13	4
Carns1	9.97E-12	########	0.193	0.032	1.67E-07	4
Stard10	5.59E-08	########	0.246	0.067	########	4
B3galt2	5.43E-25	########	0.158	0.011	9.08E-21	4
Fktn	7.58E-08	########	0.228	0.061	########	4
Ston1	1.04E-32	########	0.228	0.017	1.73E-28	4
Maoa	2.92E-11	########	0.316	0.083	4.88E-07	4
Ube2e2	4.17E-09	########	0.281	0.081	6.97E-05	4
Apbb1	3.05E-15	########	0.193	0.026	5.11E-11	4
Dok4	6.76E-09	########	0.158	0.029	########	4
Vit	2.18E-14	########	0.158	0.019	3.65E-10	4

Lox	7.17E-07	########	0.316	0.106	########	4
Tnfaip8l1	5.13E-10	########	0.211	0.044	8.59E-06	4
Afap1	2.82E-06	########	0.298	0.112	########	4
Nkd1	2.06E-39	########	0.228	0.014	3.44E-35	4
Lrig1	2.18E-06	########	0.175	0.047	########	4
Lgr4	7.42E-13	########	0.228	0.04	1.24E-08	4
Bace2	1.38E-07	########	0.193	0.048	########	4
Wbscr17	2.88E-13	########	0.228	0.039	4.82E-09	4
Cobll1	7.27E-15	########	0.298	0.058	1.22E-10	4
Tmprss5	0	########	0.193	0	0	4
Clip2	6.05E-10	########	0.281	0.072	1.01E-05	4
Lurap1	1.55E-32	########	0.123	0.005	2.59E-28	4
Per3	5.70E-08	########	0.228	0.06	########	4
Tmem117	7.01E-124	########	0.175	0.002	1.17E-119	4
Tmem14a	8.02E-14	########	0.193	0.028	1.34E-09	4
Csrnp2	9.32E-14	########	0.193	0.028	1.56E-09	4
Neb	4.21E-16	########	0.105	0.008	7.04E-12	4
Clcn6	7.41E-17	########	0.263	0.042	1.24E-12	4
Sema3e	1.55E-32	########	0.105	0.004	2.60E-28	4
Dock9	1.56E-07	########	0.228	0.063	########	4
Fam212a	1.03E-06	########	0.228	0.069	########	4
Gpx8	4.89E-08	########	0.333	0.104	########	4
Scube1	2.29E-123	########	0.158	0.001	3.84E-119	4
Crym	2.31E-107	########	0.14	0.001	3.87E-103	4
Kank2	6.84E-07	########	0.281	0.094	########	4
Gpx7	7.19E-10	########	0.368	0.107	1.20E-05	4
Frem1	1.71E-36	########	0.105	0.003	2.86E-32	4
Scn1b	2.64E-06	########	0.333	0.13	########	4
Gm15706	3.98E-10	########	0.211	0.043	6.67E-06	4
Tgfa	1.93E-11	########	0.14	0.018	3.24E-07	4
Hist3h2ba	1.08E-07	########	0.175	0.039	########	4
Spp1	1.34E-06	########	0.596	0.321	########	4
Rgmb	3.22E-09	########	0.246	0.061	5.38E-05	4
Srgap1	9.35E-12	########	0.228	0.045	1.56E-07	4
Thsd7a	2.02E-07	########	0.158	0.034	########	4
Tacc2	3.85E-07	########	0.263	0.086	0.006441	4
B9d1	6.91E-09	########	0.246	0.063	########	4
Ctsf	1.18E-07	########	0.175	0.04	########	4
Fstl3	4.04E-08	########	0.246	0.069	########	4
Kazn	1.12E-15	########	0.175	0.021	1.88E-11	4
Nkd2	3.03E-10	########	0.14	0.021	5.07E-06	4
Ntng1	8.27E-69	########	0.14	0.003	1.38E-64	4
Enpp4	1.47E-07	########	0.246	0.071	########	4
Sspn	1.28E-06	########	0.211	0.061	########	4
Dclk3	6.10E-110	########	0.123	0.001	1.02E-105	4
Aqp5	1.40E-20	########	0.105	0.006	2.35E-16	4
Asphd2	1.98E-92	########	0.175	0.003	3.31E-88	4
Tnnc1	1.19E-17	########	0.14	0.012	1.98E-13	4
Daam2	3.35E-10	########	0.123	0.016	5.61E-06	4
Grik3	1.37E-121	########	0.105	0	2.29E-117	4
Fam181b	1.25E-21	########	0.14	0.01	2.09E-17	4
Flrt3	7.83E-11	########	0.246	0.052	1.31E-06	4

Pkia	1.09E-15	########	0.193	0.025	1.82E-11	4
Krt14	6.76E-247	6.955109	1	0.173	1.13E-242	5
Lgals7	0	6.496668	1	0.116	0	5
Krt5	0	5.358095	0.939	0.05	0	5
Sfn	0	5.22337	1	0.103	0	5
Cst6	2.67E-74	5.054986	0.494	0.088	4.47E-70	5
Krt6a	0	4.998495	0.889	0.056	0	5
Krt16	0	4.663451	0.761	0.047	0	5
Krt17	0	4.629957	0.911	0.027	0	5
Krtdap	0	4.577215	0.894	0.038	0	5
Dmkn	1.93E-264	4.149971	1	0.154	3.23E-260	5
BC100530	1.73E-202	4.08043	0.717	0.081	2.89E-198	5
Krt15	0	3.808448	0.444	0.007	0	5
Perp	0	3.788447	0.994	0.024	0	5
Fxyd3	0	3.747089	0.994	0.018	0	5
Fabp5	5.54E-36	3.599074	0.889	0.559	9.26E-32	5
Aqp3	0	3.357251	0.939	0.015	0	5
Urah	0	3.31192	1	0.014	0	5
Krt79	4.93E-98	3.306899	0.278	0.022	8.25E-94	5
S100a14	0	3.275808	0.994	0.017	0	5
Dsp	0	3.229733	1	0.017	0	5
Ly6d	7.08E-196	3.211604	0.767	0.089	1.18E-191	5
Hspb1	1.97E-244	3.105935	0.939	0.126	3.30E-240	5
Defb6	4.33E-157	2.909858	0.161	0.003	7.25E-153	5
Sbsn	0	2.863925	0.972	0.077	0	5
2610528A1	0	2.701596	0.65	0.011	0	5
Serpinb5	0	2.692578	0.989	0.008	0	5
Col17a1	0	2.559285	0.861	0.009	0	5
Krt6b	1.29E-229	2.547934	0.328	0.011	2.15E-225	5
Sprr1b	0	2.485173	0.428	0.013	0	5
S100a16	1.54E-267	2.462422	0.994	0.132	2.58E-263	5
Gsta4	0	2.447557	0.644	0.017	0	5
Avpi1	3.64E-168	2.436477	0.972	0.242	6.09E-164	5
Anxa8	0	2.425018	0.978	0.039	0	5
Stfa1	2.14E-256	2.424272	0.628	0.043	3.57E-252	5
Apoc1	0	2.398341	0.9	0.057	0	5
Dsc3	0	2.369253	0.989	0.005	0	5
Mt2	4.43E-134	2.242773	0.972	0.251	7.41E-130	5
Hras	8.99E-137	2.151766	1	0.418	1.50E-132	5
Id1	1.29E-129	2.147288	0.922	0.247	2.15E-125	5
Jup	0	2.069265	1	0.095	0	5
Sdc1	1.72E-283	2.066876	0.972	0.117	2.87E-279	5
Stfa3	4.12E-274	2.021333	0.617	0.038	6.89E-270	5
Trim29	0	2.015742	0.983	0.006	0	5
Fam162a	5.76E-86	2.006583	0.939	0.394	9.64E-82	5
Tpm2	0	2.000896	0.983	0.096	0	5
Itga6	1.44E-135	1.977312	0.894	0.214	2.41E-131	5
Hmgn1	2.24E-124	1.967257	1	0.403	3.74E-120	5
Rab25	0	1.948218	0.933	0.004	0	5
Ppp1r14b	5.51E-99	1.905434	1	0.551	9.23E-95	5
Lypd3	0	1.88953	0.833	0.005	0	5
Mt1	2.14E-77	1.885171	0.994	0.544	3.58E-73	5

Ybx3	1.49E-119	1.851752	1	0.416	2.49E-115	5
Epcam	0	1.781082	0.906	0.011	0	5
Cd9	9.45E-98	1.768761	0.994	0.54	1.58E-93	5
Pkp1	0	1.763739	0.95	0.006	0	5
Gjb2	0	1.737645	0.45	0.007	0	5
Fgfbp1	0	1.733884	0.811	0.004	0	5
Mif	5.90E-84	1.711635	1	0.67	9.88E-80	5
Postn	5.28E-50	1.705669	0.433	0.093	8.83E-46	5
Txndc17	3.01E-92	1.698671	1	0.753	5.04E-88	5
Ppp1r14c	0	1.692339	0.961	0.011	0	5
Bok	0	1.687641	0.944	0.077	0	5
Phlda3	1.89E-278	1.670603	0.928	0.104	3.16E-274	5
Lmna	3.98E-90	1.656469	0.994	0.441	6.66E-86	5
Tubb4b	6.23E-63	1.653943	0.967	0.574	1.04E-58	5
Fam25c	0	1.65072	0.889	0.004	0	5
Ncl	3.74E-67	1.630533	1	0.822	6.25E-63	5
Ckmt1	0	1.626596	0.944	0.003	0	5
Npm1	1.59E-75	1.615399	0.994	0.813	2.66E-71	5
Ptn	0	1.601686	0.689	0.034	0	5
Wnt4	3.33E-232	1.598075	0.822	0.097	5.58E-228	5
Itgb4	0	1.592639	0.806	0.024	0	5
Scd1	2.00E-47	1.579542	0.467	0.105	3.35E-43	5
Sprr1a	9.76E-259	1.572644	0.244	0.004	1.63E-254	5
Crip2	1.48E-235	1.563025	0.978	0.128	2.48E-231	5
Areg	2.91E-302	1.552545	0.522	0.023	4.87E-298	5
Calm4	0	1.551489	0.444	0.005	0	5
Rps2	1.59E-86	1.549296	1	0.929	2.66E-82	5
Rbp1	1.66E-162	1.51047	0.683	0.084	2.78E-158	5
Dynll1	8.80E-74	1.481129	0.989	0.842	1.47E-69	5
Tacstd2	1.55E-206	1.475592	0.917	0.139	2.59E-202	5
Eif5a	1.16E-82	1.463138	1	0.861	1.94E-78	5
Lamc2	0	1.454529	0.683	0.023	0	5
Map1lc3a	3.31E-76	1.442144	0.917	0.372	5.54E-72	5
Anxa2	2.09E-60	1.439163	0.994	0.817	3.50E-56	5
Hsp90ab1	1.09E-80	1.411996	1	0.915	1.83E-76	5
Fam132a	3.88E-165	1.409069	0.889	0.172	6.49E-161	5
Nhp2	5.03E-93	1.396528	1	0.483	8.41E-89	5
Sox9	0	1.392671	0.706	0.042	0	5
Trp63	0	1.382831	0.939	0.005	0	5
Krt10	9.88E-159	1.372149	0.589	0.062	1.65E-154	5
Hspd1	7.74E-65	1.368462	0.961	0.532	1.30E-60	5
Glo1	1.41E-109	1.361788	0.961	0.355	2.36E-105	5
Spink5	3.22E-257	1.361478	0.311	0.008	5.39E-253	5
Dst	6.32E-127	1.354773	0.844	0.162	1.06E-122	5
Gpx2	0	1.349007	0.578	0.004	0	5
Nfib	1.85E-165	1.336888	0.906	0.135	3.10E-161	5
Ldha	2.07E-62	1.334851	0.994	0.837	3.46E-58	5
Atp5g1	1.16E-76	1.33272	1	0.727	1.94E-72	5
Higd1a	8.17E-51	1.332507	0.944	0.58	1.37E-46	5
Ptprf	0	1.314822	0.956	0.049	0	5
Eif6	6.46E-74	1.307869	0.972	0.617	1.08E-69	5
Pkp3	4.49E-297	1.305787	0.978	0.104	7.51E-293	5

Tpi1	1.65E-66	1.296998	0.989	0.638	2.76E-62	5
Cltb	1.97E-118	1.293514	0.956	0.3	3.29E-114	5
Ranbp1	8.94E-60	1.286097	0.989	0.581	1.50E-55	5
Serbp1	2.30E-76	1.284666	1	0.803	3.85E-72	5
Ndufa4	9.72E-73	1.281204	1	0.828	1.63E-68	5
Set	1.12E-62	1.279853	0.994	0.71	1.87E-58	5
Csta1	0	1.276458	0.506	0.003	0	5
Atp5b	1.06E-86	1.274199	1	0.841	1.78E-82	5
Eef1g	2.51E-74	1.252877	1	0.801	4.21E-70	5
Crabp2	8.87E-242	1.246683	0.517	0.03	1.48E-237	5
Ran	7.43E-60	1.243872	0.994	0.711	1.24E-55	5
Gja1	8.12E-108	1.243422	0.839	0.192	1.36E-103	5
Tuba4a	6.78E-62	1.237669	0.95	0.47	1.13E-57	5
Tmem45a	1.68E-131	1.235964	0.561	0.071	2.82E-127	5
Ptma	3.84E-59	1.232936	1	0.869	6.42E-55	5
Sprr2a3	0	1.230377	0.344	0.002	0	5
Jun	7.68E-41	1.226478	0.939	0.546	1.28E-36	5
Uqcr11	9.52E-82	1.224214	0.994	0.784	1.59E-77	5
Hmgb1	5.44E-56	1.219467	1	0.878	9.10E-52	5
Dstn	2.57E-61	1.214121	1	0.705	4.30E-57	5
Emp2	0	1.211773	0.922	0.083	0	5
Mrpl12	9.99E-82	1.209103	0.972	0.441	1.67E-77	5
Ggct	6.74E-97	1.204981	0.761	0.18	1.13E-92	5
Odc1	1.06E-39	1.201078	0.867	0.405	1.77E-35	5
Rpl14	1.64E-76	1.194531	1	0.886	2.75E-72	5
Eef1d	1.25E-71	1.185736	0.994	0.729	2.09E-67	5
Timm13	2.48E-71	1.185056	1	0.69	4.15E-67	5
Vdac1	1.85E-90	1.183914	0.983	0.547	3.09E-86	5
Atp1a1	5.65E-62	1.183639	0.978	0.63	9.45E-58	5
C1qbp	1.84E-81	1.182945	0.956	0.399	3.07E-77	5
Skp1a	1.93E-53	1.175614	0.961	0.532	3.23E-49	5
Psph	1.96E-149	1.174359	0.894	0.19	3.28E-145	5
Tmem238	9.76E-200	1.168291	0.922	0.156	1.63E-195	5
Pa2g4	3.82E-77	1.163605	0.944	0.426	6.39E-73	5
Erh	2.09E-60	1.160746	0.989	0.711	3.50E-56	5
Phb2	1.18E-78	1.158881	0.983	0.59	1.97E-74	5
Tagln2	4.37E-47	1.144717	0.994	0.753	7.32E-43	5
Phgdh	2.90E-164	1.14095	0.883	0.153	4.85E-160	5
Pdap1	2.18E-78	1.137178	1	0.619	3.65E-74	5
Cxadr	0	1.131317	0.872	0.014	0	5
Nme1	2.44E-53	1.125868	0.972	0.665	4.09E-49	5
Dsg3	0	1.122474	0.85	0.002	0	5
Dhcr24	8.17E-155	1.122082	0.683	0.088	1.37E-150	5
Slc25a5	2.39E-70	1.12093	1	0.83	4.00E-66	5
Idi1	5.38E-85	1.10316	0.772	0.209	9.00E-81	5
S100a6	1.24E-37	1.096849	1	0.843	2.08E-33	5
Hspe1	5.58E-56	1.095274	0.994	0.67	9.33E-52	5
Eif2s2	4.16E-65	1.088817	0.994	0.763	6.97E-61	5
S100a10	1.18E-40	1.087852	0.983	0.706	1.97E-36	5
Elovl6	2.67E-39	1.084632	0.4	0.093	4.47E-35	5
Polr2f	7.10E-76	1.084604	1	0.557	1.19E-71	5
Mgst3	2.42E-113	1.080357	0.922	0.261	4.05E-109	5

Ndufa4l2	3.90E-62	1.079727	0.378	0.062	6.53E-58	5
Tns4	0	1.078718	0.794	0.038	0	5
Snrpd2	4.19E-65	1.075814	0.983	0.688	7.01E-61	5
Polr1d	5.90E-71	1.070319	1	0.784	9.88E-67	5
Ppa1	1.19E-83	1.069253	0.889	0.289	1.99E-79	5
Tomm40	2.32E-86	1.069233	0.928	0.361	3.89E-82	5
Cyc1	3.99E-75	1.063936	0.967	0.513	6.68E-71	5
Gsto1	1.34E-93	1.060726	0.944	0.352	2.25E-89	5
Atp5o	9.80E-64	1.05908	0.983	0.751	1.64E-59	5
Serpinb6a	2.29E-64	1.057717	0.961	0.396	3.83E-60	5
Lmo1	2.91E-108	1.056081	0.878	0.224	4.87E-104	5
Uqcrc1	1.49E-80	1.054563	0.961	0.539	2.49E-76	5
Ppia	3.39E-65	1.053063	1	0.902	5.68E-61	5
Uqcc2	1.95E-69	1.051933	0.972	0.53	3.27E-65	5
Hmgcs1	1.47E-77	1.045687	0.833	0.259	2.46E-73	5
Lad1	0	1.041071	0.883	0.003	0	5
Rpl7a	4.22E-75	1.039596	1	0.919	7.07E-71	5
Hnrnpab	1.00E-56	1.033295	0.983	0.661	1.68E-52	5
Psmd7	1.25E-67	1.033198	0.944	0.497	2.09E-63	5
Atp5d	8.55E-74	1.032485	1	0.814	1.43E-69	5
Rbm3	2.07E-64	1.026403	1	0.884	3.46E-60	5
Nfix	2.69E-88	1.023902	0.933	0.289	4.51E-84	5
Dusp7	3.45E-110	1.023142	0.889	0.245	5.78E-106	5
Rpl3	6.05E-64	1.022985	1	0.866	1.01E-59	5
Defb1	0	1.022527	0.528	0.003	0	5
Fst	2.50E-85	1.020567	0.461	0.069	4.19E-81	5
Eif3i	8.47E-71	1.017756	0.994	0.688	1.42E-66	5
Spint2	9.35E-213	1.014401	0.967	0.146	1.57E-208	5
Lamb3	8.20E-293	1.014258	0.7	0.046	1.37E-288	5
Rpl11	8.31E-69	1.009456	1	0.951	1.39E-64	5
Rpl10a	1.17E-60	1.007191	1	0.872	1.96E-56	5
Palmd	0	########	0.817	0.034	0	5
Enah	5.10E-238	########	0.867	0.097	8.53E-234	5
Plek2	0	########	0.85	0.005	0	5
Capns2	0	########	0.672	0.007	0	5
Tomm20	3.40E-60	########	0.994	0.754	5.69E-56	5
Ube2s	3.90E-42	########	1	0.688	6.53E-38	5
Rbp2	0	########	0.456	0.002	0	5
Eif1ax	1.72E-74	########	0.972	0.536	2.88E-70	5
Anp32b	4.50E-39	########	0.983	0.739	7.53E-35	5
Ptms	3.45E-55	########	0.989	0.492	5.78E-51	5
Cdh1	0	########	0.883	0.021	0	5
Rps17	1.61E-66	########	1	0.889	2.69E-62	5
Serpinb2	8.79E-118	########	0.589	0.083	1.47E-113	5
Epgn	0	########	0.4	0.005	0	5
mt-Nd1	2.19E-43	########	0.989	0.905	3.67E-39	5
Eif4a1	3.39E-55	########	1	0.831	5.67E-51	5
Sfr1	9.47E-51	########	0.989	0.7	1.58E-46	5
Mllt4	5.09E-150	########	0.861	0.153	8.52E-146	5
Ly6g6c	4.02E-86	########	0.144	0.006	6.73E-82	5
Gas1	4.75E-171	########	0.75	0.091	7.94E-167	5
Iffo2	6.50E-195	########	0.878	0.126	1.09E-190	5

Rpl8	9.84E-64	########	1	0.948	1.65E-59	5
Net1	1.40E-234	########	0.894	0.112	2.34E-230	5
Kctd1	0	########	0.856	0.036	0	5
Ndufa12	7.35E-66	########	0.978	0.561	1.23E-61	5
Tomm5	1.53E-59	########	0.961	0.55	2.56E-55	5
Pvrl1	0	########	0.883	0.076	0	5
Tspan3	3.97E-48	########	0.772	0.299	6.64E-44	5
Btf3	1.09E-66	########	1	0.918	1.82E-62	5
Tmem147	3.79E-67	########	0.956	0.465	6.34E-63	5
Pgam1	6.26E-39	########	0.967	0.737	1.05E-34	5
2700094K1	1.47E-55	########	0.928	0.452	2.46E-51	5
Slc2a1	6.14E-71	########	0.822	0.262	1.03E-66	5
Rps18	1.99E-51	########	1	0.912	3.33E-47	5
Rpl36al	1.14E-57	########	1	0.839	1.90E-53	5
Mrpl51	5.28E-70	########	0.967	0.518	8.84E-66	5
Dynlt3	1.90E-98	########	0.978	0.353	3.18E-94	5
Tubb5	4.58E-31	########	0.95	0.685	7.66E-27	5
Fdps	4.16E-69	########	0.872	0.293	6.97E-65	5
Pycard	2.05E-39	########	0.989	0.688	3.44E-35	5
Tnfrsf12a	1.33E-59	########	0.844	0.297	2.22E-55	5
Tsen34	5.12E-89	########	0.939	0.336	8.56E-85	5
Bod1	9.47E-170	########	0.911	0.164	1.58E-165	5
Txn1	1.89E-35	########	1	0.897	3.16E-31	5
Plet1	0	########	0.633	0.01	0	5
Ddx39	1.39E-61	########	0.911	0.397	2.33E-57	5
Pof1b	0	########	0.7	0.002	0	5
Impdh2	2.76E-71	########	0.956	0.404	4.62E-67	5
Lama3	0	########	0.628	0.006	0	5
Atp5g3	2.78E-60	########	0.994	0.741	4.65E-56	5
Ndufb9	1.60E-66	########	0.994	0.825	2.68E-62	5
Nucks1	3.43E-45	########	0.972	0.572	5.74E-41	5
Prmt1	1.32E-63	########	0.939	0.425	2.22E-59	5
Il24	0	########	0.339	0.003	0	5
Pls3	3.45E-183	########	0.883	0.129	5.77E-179	5
Atf3	1.67E-26	########	0.722	0.353	2.80E-22	5
Ryk	7.97E-208	########	0.928	0.121	1.33E-203	5
Naca	5.18E-62	########	1	0.906	8.67E-58	5
Rpl4	6.89E-58	########	1	0.883	1.15E-53	5
Lyar	1.68E-61	########	0.85	0.326	2.81E-57	5
Pebp1	1.97E-57	########	1	0.613	3.30E-53	5
Atp5a1	3.77E-60	########	0.994	0.792	6.31E-56	5
Atpif1	3.26E-51	########	0.994	0.735	5.45E-47	5
Csnk1a1	6.84E-55	########	0.983	0.777	1.14E-50	5
Ccnd1	6.70E-109	########	0.778	0.157	1.12E-104	5
Irx5	0	########	0.811	0.016	0	5
Cks1b	1.70E-89	########	0.878	0.24	2.84E-85	5
Fam213a	5.53E-229	########	0.689	0.061	9.26E-225	5
Esrp1	0	########	0.906	0.003	0	5
Nop58	4.07E-62	########	0.9	0.356	6.81E-58	5
Snrpf	1.20E-47	########	0.994	0.703	2.01E-43	5
Ndufab1	4.20E-56	########	0.983	0.588	7.03E-52	5
Irf6	0	########	0.872	0.005	0	5

Cct2	1.44E-58	########	0.989	0.673	2.41E-54	5
Rps6	2.52E-55	########	1	0.937	4.21E-51	5
Itga3	0	########	0.611	0.02	0	5
Gcat	1.94E-263	########	0.828	0.083	3.24E-259	5
Fkbp4	2.55E-82	########	0.928	0.344	4.27E-78	5
Krt42	1.60E-174	########	0.122	0.001	2.68E-170	5
Calml3	0	########	0.428	0.003	0	5
Hes1	6.28E-75	########	0.861	0.233	1.05E-70	5
Cdv3	2.01E-52	########	0.989	0.633	3.36E-48	5
Cycs	4.43E-44	########	0.961	0.587	7.41E-40	5
Dctpp1	1.75E-67	########	0.883	0.33	2.93E-63	5
Rgs12	0	########	0.856	0.056	0	5
Gm10073	8.70E-59	########	0.983	0.555	1.46E-54	5
Snrpd1	6.22E-44	########	0.961	0.565	1.04E-39	5
Klk10	0	########	0.378	0.002	0	5
Lsm4	3.03E-55	########	0.983	0.681	5.08E-51	5
Uqcr10	6.32E-53	########	0.989	0.79	1.06E-48	5
Mrto4	5.07E-71	########	0.9	0.34	8.48E-67	5
Npm3	1.18E-68	########	0.922	0.399	1.97E-64	5
Chchd10	3.60E-148	########	0.828	0.135	6.02E-144	5
Ktn1	6.24E-74	########	0.944	0.35	1.04E-69	5
Rpl24	1.13E-60	########	1	0.96	1.89E-56	5
Bsg	8.98E-55	########	0.994	0.701	1.50E-50	5
Rpl13	5.11E-49	########	1	0.961	8.54E-45	5
Glrx5	5.22E-63	########	0.956	0.459	8.74E-59	5
Cnbp	4.31E-52	########	0.994	0.704	7.22E-48	5
Hint1	2.79E-43	########	1	0.785	4.67E-39	5
Rpl12	1.38E-46	########	1	0.889	2.30E-42	5
Klc3	0	########	0.8	0.008	0	5
Cct3	3.15E-58	########	0.972	0.5	5.26E-54	5
Gnai1	0	########	0.756	0.034	0	5
Tcp1	1.23E-53	########	0.972	0.57	2.07E-49	5
Clca3a2	0	########	0.3	0.002	0	5
Cct5	6.02E-53	########	0.989	0.684	1.01E-48	5
Rps27l	1.08E-47	########	0.983	0.739	1.80E-43	5
Pxdc1	1.79E-173	########	0.728	0.091	3.00E-169	5
Rpl18	3.89E-53	########	1	0.957	6.51E-49	5
Rps8	1.09E-57	########	1	0.964	1.82E-53	5
Hspa9	2.37E-58	########	0.944	0.428	3.97E-54	5
Eif3c	1.21E-49	########	0.983	0.763	2.02E-45	5
Rpl5	7.84E-53	########	1	0.904	1.31E-48	5
Akr1b8	1.52E-130	########	0.806	0.148	2.55E-126	5
Hdgf	1.69E-54	########	0.994	0.567	2.82E-50	5
Grpel1	1.60E-55	########	0.978	0.525	2.68E-51	5
Hbegf	9.54E-87	########	0.706	0.147	1.60E-82	5
Chchd2	8.68E-63	########	1	0.961	1.45E-58	5
Mrfap1	6.06E-53	########	0.994	0.697	1.01E-48	5
Rps15	1.30E-56	########	1	0.936	2.17E-52	5
Ndufs6	1.96E-53	########	0.978	0.688	3.27E-49	5
Hnrnpa3	1.17E-35	########	1	0.832	1.96E-31	5
Lgalsl	5.47E-171	########	0.728	0.091	9.16E-167	5
Ly6a	5.42E-55	########	0.794	0.255	9.08E-51	5

Bcam	0	########	0.8	0.044	0	5
Aars	2.13E-72	########	0.939	0.369	3.56E-68	5
Sult2b1	0	########	0.45	0.009	0	5
Naa50	3.76E-57	########	0.972	0.503	6.29E-53	5
Car12	0	########	0.828	0.017	0	5
Rpl22l1	1.59E-42	########	1	0.803	2.66E-38	5
Cox7b	7.28E-47	########	0.989	0.811	1.22E-42	5
Rsl1d1	1.07E-60	########	0.939	0.408	1.79E-56	5
Prdx1	8.90E-51	########	1	0.75	1.49E-46	5
Rps3	1.24E-47	########	1	0.967	2.08E-43	5
Rpl7	7.16E-56	########	1	0.966	1.20E-51	5
Lima1	4.54E-85	########	0.883	0.255	7.60E-81	5
Dtymk	1.44E-54	########	0.883	0.37	2.41E-50	5
Uchl3	3.77E-70	########	0.906	0.346	6.32E-66	5
Pmepa1	1.41E-90	########	0.822	0.183	2.37E-86	5
Rpl28	1.12E-46	########	1	0.963	1.87E-42	5
Ebp	2.54E-60	########	0.967	0.523	4.25E-56	5
Ndufa5	1.25E-52	########	0.978	0.557	2.10E-48	5
Wnt10a	0	########	0.717	0.003	0	5
Rpl32	4.96E-44	########	1	0.936	8.29E-40	5
Cct7	1.44E-52	########	0.983	0.616	2.41E-48	5
Llph	3.70E-48	########	0.972	0.651	6.20E-44	5
Mrpl20	1.65E-48	########	0.978	0.688	2.76E-44	5
Rpl29	5.94E-47	########	1	0.909	9.94E-43	5
Ndufv1	1.91E-69	########	0.967	0.446	3.19E-65	5
Sdc4	8.56E-54	########	0.972	0.377	1.43E-49	5
Gm10076	1.92E-45	########	0.994	0.887	3.20E-41	5
Uqcrq	5.48E-45	########	0.989	0.834	9.17E-41	5
Rpl41	1.64E-57	########	1	0.992	2.74E-53	5
Sprr2h	6.41E-250	########	0.217	0.003	1.07E-245	5
Psmd12	9.19E-56	########	0.961	0.528	1.54E-51	5
Birc5	3.02E-59	########	0.661	0.172	5.04E-55	5
Cox7a2	2.56E-54	########	1	0.867	4.29E-50	5
Barx2	0	########	0.728	0.011	0	5
Lap3	2.94E-84	########	0.906	0.267	4.92E-80	5
Tnfrsf18	1.84E-229	########	0.856	0.094	3.08E-225	5
Got2	1.24E-60	########	0.928	0.441	2.08E-56	5
Gnb2l1	3.06E-45	########	1	0.922	5.13E-41	5
Rpl22	8.89E-44	########	1	0.92	1.49E-39	5
Msmo1	2.15E-52	########	0.767	0.267	3.60E-48	5
Mdh2	1.70E-47	########	0.983	0.721	2.84E-43	5
Mrps7	1.91E-74	########	0.939	0.369	3.19E-70	5
Hspa1b	2.03E-195	########	0.894	0.124	3.39E-191	5
Psmb3	5.00E-43	########	0.983	0.784	8.37E-39	5
Psat1	5.55E-139	########	0.811	0.154	9.29E-135	5
Gltp	4.17E-16	########	0.844	0.518	6.97E-12	5
Plec	3.86E-27	########	0.933	0.614	6.47E-23	5
Denr	1.62E-59	########	0.967	0.475	2.70E-55	5
Gspt1	3.44E-45	########	0.939	0.5	5.76E-41	5
Irx3	0	########	0.8	0.024	0	5
Tomm22	3.86E-50	########	0.994	0.751	6.46E-46	5
Ptges	4.89E-153	########	0.656	0.084	8.18E-149	5

Cav2	5.66E-152	########	0.856	0.147	9.48E-148	5
Psmb6	1.56E-47	########	0.983	0.708	2.60E-43	5
Acbd6	1.35E-49	########	0.95	0.431	2.26E-45	5
Mrps26	8.20E-70	########	0.944	0.4	1.37E-65	5
Cd109	3.90E-235	########	0.783	0.079	6.52E-231	5
Bcl7c	8.27E-65	########	0.956	0.423	1.38E-60	5
Wdr61	1.63E-71	########	0.911	0.373	2.72E-67	5
Acaa2	1.64E-48	########	0.922	0.443	2.75E-44	5
Apex1	2.30E-90	########	0.861	0.24	3.84E-86	5
Ccdc34	5.67E-67	########	0.861	0.274	9.49E-63	5
Slc25a3	7.22E-47	########	1	0.866	1.21E-42	5
Timm8b	1.85E-53	########	0.972	0.555	3.09E-49	5
Adamts1	3.67E-99	########	0.722	0.129	6.14E-95	5
Rplp1	1.65E-43	########	1	0.975	2.77E-39	5
Stard10	0	########	0.883	0.051	0	5
Atp2a2	6.57E-43	########	0.933	0.485	1.10E-38	5
Cdc20	2.00E-69	########	0.572	0.126	3.35E-65	5
Rps26	4.62E-41	########	1	0.948	7.72E-37	5
Ndufb10	4.49E-48	########	0.989	0.743	7.51E-44	5
Cox5b	1.86E-44	########	0.989	0.862	3.12E-40	5
Rps5	4.47E-41	########	1	0.937	7.48E-37	5
Rplp0	1.14E-36	########	1	0.964	1.91E-32	5
Gadd45gip	1.67E-58	########	0.9	0.38	2.79E-54	5
Psmc5	1.75E-50	########	0.939	0.552	2.93E-46	5
Luzp1	2.77E-101	########	0.906	0.227	4.63E-97	5
Acp1	5.61E-61	########	0.944	0.472	9.39E-57	5
Uqcrb	5.55E-44	########	0.989	0.759	9.29E-40	5
Csrp1	7.80E-74	########	0.872	0.296	1.31E-69	5
Eif3g	3.22E-53	########	0.972	0.543	5.39E-49	5
Rpl31	2.93E-49	########	1	0.905	4.90E-45	5
Ndufa11	2.38E-43	########	0.967	0.678	3.98E-39	5
Bag1	2.75E-42	########	0.961	0.672	4.60E-38	5
Blmh	2.37E-47	########	0.861	0.374	3.97E-43	5
Hadh	2.66E-92	########	0.917	0.265	4.45E-88	5
Mrpl42	4.41E-55	########	0.939	0.457	7.38E-51	5
Psmb5	1.02E-40	########	0.972	0.732	1.70E-36	5
Eef1b2	5.07E-38	########	1	0.925	8.48E-34	5
Mrps34	1.55E-68	########	0.939	0.381	2.59E-64	5
Cdca8	1.03E-71	########	0.7	0.169	1.72E-67	5
Dbi	2.12E-17	########	0.978	0.722	3.54E-13	5
Nasp	9.81E-42	########	0.922	0.473	1.64E-37	5
Rpl6	2.21E-42	########	1	0.941	3.70E-38	5
Cacybp	2.61E-40	########	0.933	0.522	4.38E-36	5
Pin1	6.93E-59	########	0.939	0.423	1.16E-54	5
Cct4	2.59E-45	########	0.967	0.63	4.34E-41	5
Rps12	1.67E-43	########	1	0.982	2.80E-39	5
Ndufb6	9.31E-49	########	0.956	0.592	1.56E-44	5
Srm	8.89E-84	########	0.828	0.21	1.49E-79	5
Mpzl2	0	########	0.717	0.004	0	5
Ptges3	1.93E-38	########	0.967	0.637	3.23E-34	5
Rpl13a	5.04E-43	########	1	0.962	8.43E-39	5
Bzw1	2.16E-35	########	0.978	0.764	3.61E-31	5

Snrpa1	2.16E-58	########	0.883	0.371	3.61E-54	5
Atp5g2	8.45E-41	########	0.994	0.813	1.41E-36	5
Rps3a1	2.75E-43	########	1	0.968	4.61E-39	5
Prdx2	2.40E-36	########	0.989	0.674	4.02E-32	5
Ndufc2	3.87E-41	########	0.983	0.673	6.48E-37	5
Celsr2	0	########	0.728	0.006	0	5
Ndufb8	5.49E-42	########	0.989	0.752	9.19E-38	5
2410015M2	3.00E-46	########	0.983	0.677	5.02E-42	5
Cyp51	1.15E-62	########	0.711	0.198	1.92E-58	5
Arpp19	9.05E-45	########	1	0.743	1.52E-40	5
Timm17a	1.37E-58	########	0.933	0.399	2.30E-54	5
Eef2	1.48E-41	########	1	0.891	2.47E-37	5
Naa10	9.09E-63	########	0.928	0.394	1.52E-58	5
Cox5a	1.22E-39	########	0.989	0.833	2.05E-35	5
Car13	3.24E-253	########	0.778	0.074	5.42E-249	5
Klf5	8.17E-211	########	0.772	0.087	1.37E-206	5
Ebna1bp2	2.88E-46	########	0.911	0.436	4.82E-42	5
Gjb3	0	########	0.689	0.034	0	5
Atp1b3	3.45E-34	########	0.983	0.707	5.77E-30	5
Banf1	5.14E-43	########	0.956	0.545	8.60E-39	5
Nolc1	9.97E-55	########	0.794	0.286	1.67E-50	5
Nedd4	2.25E-67	########	0.944	0.292	3.77E-63	5
Nradd	8.65E-196	########	0.767	0.092	1.45E-191	5
Serinc2	0	########	0.794	0.009	0	5
Cers4	9.42E-18	########	0.278	0.086	1.58E-13	5
Gnl3	7.55E-63	########	0.894	0.309	1.26E-58	5
Imp3	1.32E-48	########	0.944	0.489	2.21E-44	5
Moxd1	0	########	0.683	0.012	0	5
Hr	0	########	0.806	0.016	0	5
Kif21a	1.23E-289	########	0.706	0.049	2.06E-285	5
Ndufs7	4.09E-45	########	0.961	0.604	6.84E-41	5
Nhp2l1	1.85E-38	########	0.967	0.589	3.10E-34	5
Pard6g	0	########	0.783	0.04	0	5
Mrpl17	2.80E-50	########	0.939	0.456	4.69E-46	5
Psmb2	1.34E-39	########	0.994	0.691	2.24E-35	5
mt-Cytb	2.88E-26	########	0.994	0.964	4.81E-22	5
Eprs	1.05E-48	########	0.95	0.447	1.76E-44	5
Fos	1.13E-20	########	0.917	0.589	1.90E-16	5
Rpl36	1.99E-36	########	1	0.906	3.32E-32	5
Sf3b5	2.55E-40	########	0.983	0.686	4.26E-36	5
Ssr2	3.40E-46	########	0.944	0.502	5.68E-42	5
Psapl1	1.62E-228	########	0.211	0.003	2.72E-224	5
Snrpe	6.14E-36	########	0.994	0.774	1.03E-31	5
Cct8	3.08E-39	########	0.978	0.655	5.16E-35	5
Srsf3	4.19E-30	########	0.983	0.779	7.01E-26	5
Bcl11b	2.45E-266	########	0.878	0.075	4.09E-262	5
Eif3b	4.72E-51	########	0.944	0.423	7.90E-47	5
Nrarp	0	########	0.711	0.042	0	5
Nckap1	1.97E-180	########	0.856	0.116	3.30E-176	5
Ube2c	5.64E-37	########	0.6	0.198	9.44E-33	5
Aimp1	4.20E-44	########	0.961	0.602	7.03E-40	5
Zfp593	1.60E-81	########	0.844	0.246	2.68E-77	5

Psmc6	3.03E-52	########	0.983	0.569	5.07E-48	5
Cyb5r3	1.48E-48	########	0.906	0.37	2.48E-44	5
Exosc7	8.39E-80	########	0.861	0.273	1.40E-75	5
Cenpf	2.84E-39	########	0.461	0.123	4.75E-35	5
Fbl	8.95E-40	########	0.894	0.486	1.50E-35	5
Rrm2	7.45E-87	########	0.711	0.149	1.25E-82	5
Pdcd5	7.34E-38	########	0.983	0.649	1.23E-33	5
Actn4	4.58E-44	########	0.972	0.551	7.66E-40	5
Il17rc	0	########	0.817	0.065	0	5
1810011O1	6.33E-155	########	0.65	0.078	1.06E-150	5
Pafah1b3	6.83E-117	########	0.922	0.213	1.14E-112	5
Ube2m	6.36E-42	########	0.972	0.635	1.06E-37	5
Rdx	2.49E-40	########	0.961	0.548	4.16E-36	5
Gapdh	1.58E-25	########	1	0.943	2.64E-21	5
Tecr	1.47E-30	########	0.944	0.593	2.45E-26	5
Plpp2	1.73E-216	########	0.883	0.111	2.90E-212	5
Nudc	1.68E-37	########	0.956	0.531	2.80E-33	5
Pqlc1	7.30E-65	########	0.894	0.34	1.22E-60	5
4631405K0	0	########	0.717	0.002	0	5
Htra1	4.60E-119	########	0.617	0.09	7.69E-115	5
Glrx3	3.00E-44	########	0.956	0.523	5.03E-40	5
Ndufa10	8.46E-48	########	0.956	0.515	1.41E-43	5
Gstm5	2.59E-102	########	0.706	0.133	4.33E-98	5
Psma3	9.47E-34	########	0.967	0.772	1.58E-29	5
Atp5j2	1.13E-40	########	1	0.866	1.90E-36	5
Cox6b1	2.54E-42	########	1	0.91	4.25E-38	5
Mast4	3.26E-162	########	0.839	0.121	5.45E-158	5
mt-Atp6	1.82E-23	########	0.994	0.989	3.04E-19	5
Atp5k	3.82E-39	########	0.978	0.807	6.39E-35	5
Larp1b	1.19E-83	########	0.889	0.275	1.98E-79	5
Pfdn1	4.38E-50	########	0.933	0.463	7.33E-46	5
Ndufb5	6.17E-38	########	0.989	0.723	1.03E-33	5
Mrpl57	3.71E-40	########	0.95	0.583	6.21E-36	5
Hsp90aa1	2.79E-23	########	0.978	0.804	4.67E-19	5
Sssca1	2.90E-71	########	0.861	0.277	4.86E-67	5
Eif4g1	4.26E-34	########	0.933	0.533	7.12E-30	5
Atp5c1	5.92E-36	########	0.994	0.81	9.91E-32	5
Rpl9	9.68E-36	########	1	0.975	1.62E-31	5
mt-Nd2	5.74E-22	########	0.989	0.89	9.61E-18	5
Etfa	2.62E-50	########	0.956	0.476	4.39E-46	5
Rpl27a	1.36E-35	########	1	0.978	2.28E-31	5
Efnb1	2.01E-249	########	0.744	0.065	3.36E-245	5
Tfap2a	0	########	0.806	0.004	0	5
Pkm	9.94E-26	########	0.983	0.885	1.66E-21	5
Cldn1	7.65E-290	########	0.567	0.029	1.28E-285	5
Id3	4.61E-28	########	0.678	0.27	7.72E-24	5
Stmn1	1.53E-53	########	0.906	0.313	2.56E-49	5
Rps4x	8.96E-34	0.622042	1	0.938	1.50E-29	5
Rps20	1.12E-32	########	1	0.935	1.87E-28	5
Zfp706	1.12E-40	########	0.967	0.631	1.88E-36	5
Hist1h2bc	1.84E-46	########	0.889	0.36	3.08E-42	5
Metap2	2.30E-35	########	0.972	0.631	3.85E-31	5

Fermt1	0	########	0.744	0.002	0	5
Rpl15	1.08E-34	########	1	0.826	1.80E-30	5
Krtcap3	0	########	0.783	0.037	0	5
Gipc1	2.25E-79	########	0.9	0.298	3.77E-75	5
Eif5b	1.76E-32	########	0.967	0.637	2.95E-28	5
Srsf7	5.38E-30	########	0.878	0.493	9.01E-26	5
Limk2	2.50E-189	########	0.85	0.121	4.19E-185	5
Col18a1	3.15E-177	########	0.717	0.081	5.28E-173	5
Uqcrfs1	3.19E-39	########	0.956	0.618	5.33E-35	5
Rpl19	6.74E-36	########	1	0.963	1.13E-31	5
1110004E0	5.47E-127	########	0.806	0.156	9.16E-123	5
Ddr1	0	########	0.694	0.041	0	5
F11r	5.53E-165	########	0.878	0.126	9.25E-161	5
Idh3a	1.45E-80	########	0.856	0.256	2.43E-76	5
Mdh1	1.70E-38	########	0.961	0.55	2.85E-34	5
Hsd17b12	7.85E-39	########	0.917	0.426	1.31E-34	5
Hspa4l	2.66E-85	########	0.872	0.244	4.45E-81	5
Asns	4.40E-123	########	0.789	0.154	7.37E-119	5
Psmd6	1.09E-43	########	0.956	0.538	1.82E-39	5
Oxct1	6.46E-44	########	0.906	0.389	1.08E-39	5
Rbm8a	1.22E-38	########	0.967	0.661	2.05E-34	5
Actn1	2.86E-32	########	0.933	0.448	4.79E-28	5
Ndufc1	2.14E-35	########	0.978	0.666	3.58E-31	5
Psma5	6.72E-35	########	0.972	0.624	1.13E-30	5
Ywhae	4.00E-34	########	0.994	0.781	6.70E-30	5
Vdr	1.98E-259	########	0.711	0.055	3.30E-255	5
Pmm1	3.32E-74	########	0.744	0.201	5.56E-70	5
Mybbp1a	1.26E-49	########	0.844	0.325	2.11E-45	5
Rps6ka4	3.83E-63	########	0.761	0.241	6.40E-59	5
Cdh3	0	########	0.728	0.005	0	5
Rpsa	7.75E-28	########	1	0.934	1.30E-23	5
Psma4	1.27E-36	########	0.978	0.689	2.13E-32	5
Cd151	6.31E-110	########	0.867	0.192	1.06E-105	5
Ctnnb1	2.72E-30	########	0.967	0.609	4.56E-26	5
Ola1	1.64E-51	########	0.928	0.456	2.74E-47	5
Sqle	2.90E-148	########	0.733	0.107	4.86E-144	5
Lsm2	8.35E-48	########	0.867	0.36	1.40E-43	5
Fam83g	0	########	0.728	0.008	0	5
Stoml2	1.41E-56	########	0.883	0.366	2.35E-52	5
Ptpn13	0	########	0.806	0.038	0	5
Cks2	3.74E-30	########	0.828	0.397	6.26E-26	5
Nap1l1	9.09E-28	########	0.983	0.664	1.52E-23	5
Cox7c	2.70E-37	########	1	0.919	4.51E-33	5
Tbrg1	5.55E-40	########	0.933	0.459	9.28E-36	5
Ndufs3	9.03E-45	########	0.956	0.51	1.51E-40	5
Rpl23a	4.85E-37	########	1	0.909	8.11E-33	5
Rpl36a	3.28E-32	########	1	0.867	5.49E-28	5
Ndufs2	2.35E-43	########	0.978	0.61	3.93E-39	5
Naa20	6.08E-59	########	0.906	0.366	1.02E-54	5
Eif2s1	1.13E-38	########	0.922	0.484	1.89E-34	5
Ezr	8.92E-32	########	0.956	0.583	1.49E-27	5
Mrps24	5.21E-35	########	0.956	0.648	8.72E-31	5

Elof1	3.11E-46	########	0.933	0.484	5.20E-42	5
Sgk1	2.83E-22	########	0.8	0.39	4.73E-18	5
Sdr16c6	2.56E-192	########	0.2	0.004	4.28E-188	5
Syncrip	3.12E-34	########	0.939	0.56	5.22E-30	5
Atp5j	6.49E-39	########	1	0.869	1.09E-34	5
Tk1	2.56E-90	########	0.717	0.154	4.28E-86	5
Arhgef5	0	########	0.8	0.033	0	5
Psmb7	2.06E-43	########	0.972	0.601	3.45E-39	5
Pop5	3.29E-54	########	0.922	0.395	5.50E-50	5
Mrpl21	9.74E-46	########	0.928	0.459	1.63E-41	5
Nop56	8.64E-44	########	0.872	0.371	1.45E-39	5
Eif3l	7.90E-43	########	0.933	0.519	1.32E-38	5
Casz1	2.15E-124	########	0.639	0.094	3.60E-120	5
Prnp	6.54E-104	########	0.844	0.18	1.09E-99	5
Dpy30	3.12E-58	########	0.933	0.386	5.21E-54	5
Zak	3.96E-110	########	0.844	0.191	6.63E-106	5
Polr2j	6.04E-38	########	0.961	0.591	1.01E-33	5
Polr2e	3.94E-45	########	0.956	0.488	6.59E-41	5
Cdca3	1.13E-66	########	0.694	0.173	1.88E-62	5
Strap	3.94E-42	########	0.911	0.48	6.59E-38	5
Mrps14	3.04E-36	########	0.967	0.645	5.09E-32	5
Atp5f1	2.08E-37	########	1	0.882	3.47E-33	5
Cysrt1	0	########	0.528	0.001	0	5
Fkbp3	1.48E-39	########	0.933	0.473	2.48E-35	5
Cox4i1	5.84E-35	########	1	0.957	9.77E-31	5
Phf5a	1.32E-40	########	0.933	0.496	2.22E-36	5
Nrg1	4.15E-71	########	0.5	0.095	6.94E-67	5
Spc24	2.25E-77	########	0.739	0.173	3.76E-73	5
Psmd3	4.84E-51	########	0.922	0.407	8.10E-47	5
Mrpl28	2.57E-45	########	0.928	0.463	4.30E-41	5
U2af1	1.29E-31	########	0.95	0.591	2.16E-27	5
Cenpa	2.81E-31	########	0.689	0.289	4.71E-27	5
Rwdd1	5.84E-33	########	0.967	0.602	9.78E-29	5
Psmd1	1.87E-37	########	0.894	0.455	3.13E-33	5
Rnf126	3.24E-77	########	0.817	0.244	5.42E-73	5
Psma2	3.36E-33	########	0.989	0.797	5.63E-29	5
Spr	5.39E-77	########	0.9	0.276	9.03E-73	5
Pi15	2.58E-114	########	0.428	0.045	4.31E-110	5
Rrm1	1.87E-53	########	0.756	0.239	3.13E-49	5
Mrpl13	9.34E-58	########	0.917	0.369	1.56E-53	5
Myof	2.31E-44	########	0.778	0.29	3.86E-40	5
Rps12-ps3	1.46E-40	########	0.95	0.539	2.44E-36	5
Adgrl2	1.19E-87	########	0.617	0.115	2.00E-83	5
Hspa4	1.09E-27	########	0.939	0.592	1.82E-23	5
Knop1	5.00E-57	########	0.889	0.323	8.36E-53	5
Hoxa7	0	########	0.739	0.049	0	5
Cdcp1	0	########	0.733	0.002	0	5
Magoh	1.55E-34	########	0.961	0.611	2.59E-30	5
Smc2	2.52E-39	########	0.694	0.244	4.21E-35	5
Ndufb2	2.27E-35	########	0.972	0.598	3.80E-31	5
Ppl	0	########	0.667	0.011	0	5
Rps13	5.82E-33	########	1	0.974	9.73E-29	5

Prelid1	1.88E-28	########	0.989	0.799	3.15E-24	5
Cox8a	9.38E-36	########	1	0.954	1.57E-31	5
Eif1ad	6.48E-55	########	0.867	0.345	1.08E-50	5
Bola1	4.35E-61	########	0.822	0.282	7.28E-57	5
Pcdh7	9.09E-141	########	0.644	0.083	1.52E-136	5
Ctnna1	5.71E-47	########	0.939	0.417	9.55E-43	5
Pole3	6.62E-69	########	0.856	0.275	1.11E-64	5
Ccl27a	1.84E-137	########	0.494	0.051	3.07E-133	5
Ift43	1.53E-156	########	0.817	0.121	2.56E-152	5
Fdx1	9.77E-93	########	0.889	0.236	1.63E-88	5
Sfrp1	3.12E-160	########	0.561	0.053	5.22E-156	5
Cnih4	9.29E-33	########	0.961	0.622	1.55E-28	5
Rps10	5.00E-32	########	1	0.983	8.37E-28	5
Emc4	4.65E-68	########	0.894	0.31	7.78E-64	5
Endou	0	########	0.389	0.01	0	5
Cdk4	1.34E-39	########	0.894	0.403	2.24E-35	5
Mrpl54	2.52E-33	########	0.933	0.56	4.21E-29	5
Srp72	1.52E-38	########	0.95	0.533	2.54E-34	5
Hdlbp	8.70E-36	########	0.972	0.592	1.46E-31	5
Cpe	2.20E-306	########	0.706	0.043	3.68E-302	5
Nudt5	2.98E-61	########	0.828	0.277	4.99E-57	5
Jag1	1.22E-186	########	0.728	0.081	2.04E-182	5
Park7	3.09E-29	########	0.994	0.725	5.16E-25	5
Pgap2	7.13E-62	########	0.922	0.349	1.19E-57	5
Tfap2b	0	########	0.578	0.002	0	5
Yrdc	9.07E-62	########	0.867	0.302	1.52E-57	5
Rangap1	1.70E-56	########	0.822	0.285	2.84E-52	5
Dgcr6	1.58E-82	########	0.883	0.247	2.64E-78	5
Efnb2	1.15E-130	########	0.522	0.059	1.92E-126	5
Mrpl35	3.19E-53	########	0.894	0.365	5.34E-49	5
Eif3d	1.54E-36	########	0.928	0.516	2.58E-32	5
Snrpb	2.05E-26	########	0.978	0.738	3.42E-22	5
Irx2	0	########	0.672	0.01	0	5
Eif3m	2.27E-32	########	0.983	0.708	3.80E-28	5
Psmc3	3.37E-33	########	0.956	0.635	5.63E-29	5
Mrpl18	3.23E-27	########	0.922	0.559	5.40E-23	5
Aplp2	2.14E-26	########	0.944	0.519	3.59E-22	5
Dkc1	3.14E-66	########	0.8	0.237	5.25E-62	5
Sostdc1	0	########	0.328	0.004	0	5
Tacc2	6.86E-253	########	0.783	0.072	1.15E-248	5
Rcc2	4.09E-50	########	0.889	0.364	6.85E-46	5
Tbca	1.94E-31	########	0.983	0.715	3.24E-27	5
Yap1	4.11E-224	########	0.794	0.083	6.88E-220	5
Sema3c	3.49E-241	########	0.667	0.052	5.83E-237	5
Plp2	3.25E-30	########	0.944	0.542	5.43E-26	5
Timm50	6.96E-67	########	0.844	0.278	1.16E-62	5
Wdr18	3.25E-73	########	0.811	0.24	5.44E-69	5
Snrpd3	8.14E-26	########	0.944	0.667	1.36E-21	5
Gar1	5.70E-68	########	0.783	0.227	9.54E-64	5
Dut	6.07E-42	########	0.739	0.261	1.02E-37	5
Ndufb4	6.59E-34	########	0.994	0.709	1.10E-29	5
Ccnb1	2.71E-67	########	0.544	0.115	4.53E-63	5

1810037I17	2.36E-13	########	0.961	0.764	3.94E-09	5
Ptprz1	0	########	0.489	0.002	0	5
Ywhaq	7.12E-30	########	0.961	0.629	1.19E-25	5
Capg	1.02E-24	########	0.972	0.667	1.71E-20	5
Rnd3	1.68E-80	########	0.811	0.199	2.81E-76	5
Eif4g2	3.31E-24	########	0.994	0.862	5.55E-20	5
Emg1	8.15E-31	########	0.928	0.561	1.36E-26	5
Golim4	3.20E-38	########	0.789	0.311	5.36E-34	5
Ube2v2	9.09E-64	########	0.917	0.336	1.52E-59	5
Tmem97	1.11E-60	########	0.689	0.189	1.87E-56	5
Dnph1	4.92E-185	########	0.661	0.071	8.24E-181	5
Eif3a	2.80E-21	########	0.978	0.738	4.69E-17	5
Cisd1	3.09E-43	########	0.856	0.325	5.16E-39	5
Hnrnpa0	9.80E-23	########	0.989	0.801	1.64E-18	5
Gclm	2.36E-39	########	0.9	0.397	3.95E-35	5
Eny2	4.26E-38	########	0.95	0.483	7.12E-34	5
Myl12a	6.51E-26	########	0.983	0.84	1.09E-21	5
Eef1a1	4.89E-25	########	1	0.987	8.19E-21	5
Slc25a39	2.67E-48	########	0.883	0.376	4.46E-44	5
Brix1	1.33E-61	########	0.872	0.296	2.22E-57	5
Minos1	1.49E-28	########	0.994	0.767	2.50E-24	5
Cbr3	9.04E-102	########	0.75	0.147	1.51E-97	5
Gm16136	0	########	0.6	0	0	5
Reep4	2.84E-111	########	0.822	0.174	4.75E-107	5
Arl4a	2.97E-55	########	0.806	0.255	4.97E-51	5
Eif3k	5.92E-29	########	1	0.854	9.91E-25	5
Hagh	2.53E-54	########	0.861	0.336	4.24E-50	5
Sdhd	3.78E-30	########	0.894	0.548	6.32E-26	5
Mlf2	7.08E-42	########	0.933	0.467	1.19E-37	5
Anln	1.12E-64	########	0.572	0.13	1.87E-60	5
Ssb	3.74E-24	########	0.972	0.689	6.26E-20	5
Dnajc2	5.57E-39	########	0.9	0.412	9.31E-35	5
Teddm3	0	########	0.289	0.001	0	5
Ybx1	1.12E-23	########	1	0.91	1.87E-19	5
Txnl4a	1.19E-49	########	0.878	0.357	2.00E-45	5
Rnh1	1.19E-19	########	0.978	0.687	1.99E-15	5
Psmb4	1.15E-28	########	0.978	0.764	1.92E-24	5
Tfap2c	0	########	0.694	0.002	0	5
Mki67	5.08E-32	########	0.617	0.219	8.50E-28	5
Tuba1c	9.09E-25	########	0.917	0.536	1.52E-20	5
Fosl1	6.57E-79	########	0.611	0.125	1.10E-74	5
Cdk1	1.30E-39	########	0.533	0.156	2.18E-35	5
Morf4l2	3.53E-33	########	0.944	0.511	5.90E-29	5
Ak2	5.48E-28	########	0.9	0.533	9.17E-24	5
Syne2	3.72E-65	########	0.806	0.233	6.22E-61	5
Mrps25	1.85E-48	########	0.872	0.365	3.09E-44	5
Rpl26	5.85E-25	########	1	0.968	9.78E-21	5
Adrb2	3.66E-20	########	0.611	0.276	6.12E-16	5
Rps23	9.17E-29	########	1	0.977	1.53E-24	5
Cldnd1	8.86E-62	########	0.889	0.312	1.48E-57	5
Sumo2	3.48E-23	########	0.978	0.779	5.82E-19	5
2010111I01	1.69E-65	########	0.839	0.255	2.82E-61	5

Cpne8	2.38E-172	########	0.783	0.1	3.99E-168	5
1810022K0	1.22E-31	########	0.944	0.542	2.04E-27	5
Ndufv2	1.36E-29	########	0.978	0.627	2.28E-25	5
Aldoa	5.68E-18	########	0.989	0.867	9.50E-14	5
Arhgef12	3.75E-108	########	0.844	0.169	6.27E-104	5
Prc1	1.71E-41	########	0.494	0.131	2.86E-37	5
Aldh3a1	0	########	0.394	0.002	0	5
Slc27a3	3.59E-181	########	0.589	0.056	6.00E-177	5
Cd81	9.28E-49	########	0.95	0.365	1.55E-44	5
Pfdn6	2.26E-28	########	0.922	0.551	3.78E-24	5
Nt5dc2	2.54E-41	########	0.767	0.279	4.25E-37	5
Cdk2ap1	8.67E-50	########	0.85	0.346	1.45E-45	5
Ces2g	0	########	0.594	0.018	0	5
Reep5	7.39E-13	########	0.994	0.815	1.24E-08	5
Dazap1	5.38E-50	########	0.917	0.395	9.00E-46	5
Emc6	1.21E-33	########	0.917	0.503	2.03E-29	5
Nop10	2.01E-27	########	0.961	0.589	3.37E-23	5
Phb	2.17E-62	########	0.844	0.283	3.64E-58	5
Alyref	1.76E-31	########	0.9	0.471	2.95E-27	5
Dsg1a	0	########	0.272	0.001	0	5
Fscn1	3.38E-107	########	0.611	0.094	5.65E-103	5
Ngfrap1	7.21E-59	########	0.794	0.248	1.21E-54	5
Arhgap5	4.29E-97	########	0.844	0.188	7.17E-93	5
Qars	1.07E-55	########	0.911	0.359	1.80E-51	5
Dnajb1	1.14E-43	########	0.906	0.397	1.91E-39	5
Lrrc59	2.26E-31	########	0.856	0.424	3.79E-27	5
Cd3eap	4.04E-79	########	0.739	0.186	6.75E-75	5
Elovl7	7.25E-177	########	0.633	0.063	1.21E-172	5
Psmc4	2.03E-33	########	0.922	0.537	3.40E-29	5
Srsf2	3.69E-26	########	0.978	0.743	6.18E-22	5
Txn2	4.32E-33	########	0.928	0.535	7.23E-29	5
Pinlyp	0	########	0.311	0.001	0	5
Cenpw	2.66E-72	########	0.717	0.178	4.46E-68	5
Ammecr1	4.05E-90	########	0.722	0.159	6.78E-86	5
H2afx	2.03E-42	########	0.722	0.25	3.40E-38	5
Eci2	4.28E-41	########	0.839	0.358	7.15E-37	5
Ormdl3	2.39E-93	########	0.756	0.163	4.00E-89	5
Bcas2	6.71E-34	########	0.944	0.549	1.12E-29	5
Slc25a17	1.81E-78	########	0.85	0.242	3.03E-74	5
Ifrd2	2.86E-132	########	0.706	0.11	4.78E-128	5
Lsm3	4.51E-29	########	0.833	0.434	7.54E-25	5
Akr1c18	4.35E-180	########	0.172	0.003	7.28E-176	5
Snrpg	1.16E-23	########	0.989	0.698	1.94E-19	5
Fgfr3	0	########	0.511	0.01	0	5
Smap1	5.48E-34	########	0.972	0.598	9.17E-30	5
Fyttd1	1.17E-55	########	0.867	0.303	1.95E-51	5
Tpd52l2	1.38E-43	########	0.906	0.419	2.30E-39	5
Rps25	3.49E-26	########	1	0.966	5.85E-22	5
Itgb1	6.99E-08	########	0.9	0.645	########	5
Acadl	6.37E-23	########	0.894	0.487	1.07E-18	5
Ahnak2	1.87E-117	########	0.567	0.076	3.14E-113	5
Gm5416	1.57E-211	########	0.306	0.01	2.63E-207	5

Anxa7	1.96E-38	########	0.922	0.465	3.29E-34	5
Prpf19	4.14E-53	########	0.822	0.303	6.92E-49	5
Cttn	5.06E-137	########	0.756	0.113	8.46E-133	5
H2afj	2.24E-21	########	0.983	0.851	3.74E-17	5
Sigmar1	6.08E-72	########	0.811	0.231	1.02E-67	5
Top2a	1.88E-37	########	0.561	0.17	3.15E-33	5
Uqcrc2	1.18E-33	########	0.95	0.56	1.98E-29	5
Slco2a1	5.10E-186	########	0.567	0.048	8.54E-182	5
Mapk6	4.81E-37	########	0.878	0.384	8.05E-33	5
Nxn	6.61E-66	########	0.822	0.246	1.11E-61	5
Stom	1.37E-56	########	0.822	0.28	2.30E-52	5
Cast	2.00E-29	########	0.939	0.537	3.35E-25	5
H1f0	1.35E-44	########	0.733	0.245	2.27E-40	5
Nop16	1.52E-67	########	0.744	0.207	2.54E-63	5
Mvk	9.88E-82	########	0.678	0.153	1.65E-77	5
Fam167a	0	########	0.556	0.012	0	5
Rps11	1.17E-24	########	1	0.965	1.97E-20	5
Palld	2.22E-118	########	0.661	0.101	3.71E-114	5
Cdk6	3.74E-62	########	0.772	0.219	6.26E-58	5
Eif5	1.29E-23	########	0.983	0.837	2.17E-19	5
Sars	1.07E-43	########	0.933	0.437	1.80E-39	5
Tmed9	3.99E-27	########	0.983	0.666	6.68E-23	5
Flnb	6.71E-104	########	0.706	0.127	1.12E-99	5
Arf5	1.12E-28	########	1	0.894	1.88E-24	5
Fam83h	0	########	0.694	0.002	0	5
Mrps16	4.33E-30	########	0.956	0.555	7.25E-26	5
Grhpr	1.04E-97	########	0.794	0.178	1.74E-93	5
Cfdp1	1.99E-34	########	0.917	0.482	3.32E-30	5
Thoc7	1.54E-37	########	0.95	0.466	2.58E-33	5
Sh3pxd2a	3.62E-76	########	0.767	0.182	6.06E-72	5
Mrpl30	1.39E-28	########	0.983	0.669	2.32E-24	5
Dek	5.37E-20	########	0.967	0.667	8.98E-16	5
Eif4ebp1	1.48E-23	########	0.928	0.628	2.47E-19	5
Abcf1	1.52E-34	########	0.933	0.518	2.55E-30	5
Nol7	1.34E-27	########	0.967	0.659	2.25E-23	5
Ftsj3	2.44E-59	########	0.756	0.228	4.08E-55	5
Aimp2	1.42E-68	########	0.75	0.212	2.38E-64	5
Hmgcr	1.43E-22	########	0.678	0.305	2.39E-18	5
Dag1	1.14E-72	########	0.794	0.212	1.91E-68	5
Psma7	8.55E-23	########	0.994	0.82	1.43E-18	5
Immt	2.84E-32	########	0.889	0.454	4.75E-28	5
Fam84a	0	########	0.4	0.002	0	5
Rpl21	1.82E-25	########	1	0.949	3.04E-21	5
Mrps5	8.57E-55	########	0.811	0.281	1.43E-50	5
Plch2	0	########	0.667	0.002	0	5
Scel	0	########	0.367	0.004	0	5
Ggh	5.15E-21	########	0.889	0.49	8.61E-17	5
Hells	3.96E-52	########	0.578	0.15	6.63E-48	5
Gata3	0	########	0.611	0.029	0	5
Purb	3.04E-23	########	0.972	0.705	5.09E-19	5
Nip7	8.65E-58	########	0.828	0.282	1.45E-53	5
Mrpl55	7.78E-55	########	0.833	0.307	1.30E-50	5

Il13ra1	4.95E-45	########	0.928	0.373	8.29E-41	5
H2afz	1.06E-15	########	0.994	0.914	1.77E-11	5
Nsun2	3.57E-55	########	0.811	0.275	5.97E-51	5
Hnrnpa1	4.90E-21	########	0.978	0.678	8.20E-17	5
Lsm5	2.11E-27	########	0.889	0.528	3.53E-23	5
Dcun1d5	5.12E-31	########	0.883	0.484	8.57E-27	5
Pum3	3.83E-49	########	0.783	0.286	6.40E-45	5
Ctdspl	1.26E-90	########	0.794	0.188	2.10E-86	5
Coq7	1.15E-67	########	0.789	0.23	1.92E-63	5
Myo1b	5.01E-110	########	0.667	0.111	8.39E-106	5
Elovl4	1.22E-77	########	0.228	0.019	2.04E-73	5
Egln1	2.43E-33	########	0.828	0.387	4.07E-29	5
Ube2i	9.86E-26	########	0.989	0.749	1.65E-21	5
Fhl2	4.81E-194	########	0.644	0.061	8.05E-190	5
Ssna1	6.10E-35	########	0.906	0.45	1.02E-30	5
Akt1s1	3.96E-84	########	0.861	0.229	6.62E-80	5
Ap1s1	3.38E-37	########	0.878	0.395	5.65E-33	5
Dohh	2.22E-55	########	0.839	0.3	3.71E-51	5
Samm50	1.23E-36	########	0.933	0.483	2.05E-32	5
Rbbp7	1.84E-33	########	0.883	0.402	3.08E-29	5
Ehf	0	########	0.394	0.003	0	5
Klf4	1.14E-20	########	0.961	0.502	1.90E-16	5
Ass1	1.45E-92	########	0.656	0.121	2.43E-88	5
Mgll	2.11E-12	########	0.322	0.125	3.53E-08	5
Itpr3	3.19E-183	########	0.772	0.09	5.34E-179	5
Rpl17	1.83E-26	########	1	0.983	3.05E-22	5
Ndufs5	2.59E-26	########	0.983	0.728	4.33E-22	5
Ddx21	6.27E-23	########	0.894	0.502	1.05E-18	5
Pak1ip1	9.82E-34	########	0.883	0.467	1.64E-29	5
Romo1	3.02E-26	########	0.983	0.682	5.05E-22	5
Cops5	2.88E-36	########	0.917	0.477	4.81E-32	5
Nudt2	2.78E-82	########	0.794	0.205	4.65E-78	5
Clpp	2.68E-59	########	0.806	0.259	4.49E-55	5
Slc25a4	1.26E-31	########	0.972	0.511	2.11E-27	5
Ssrp1	1.09E-29	########	0.883	0.445	1.83E-25	5
Tmem54	0	########	0.644	0.002	0	5
Aasdhppt	5.02E-73	########	0.789	0.219	8.41E-69	5
Pmvk	5.90E-43	########	0.806	0.31	9.88E-39	5
Tgfa	0	########	0.528	0.009	0	5
Shmt2	3.69E-105	########	0.772	0.155	6.17E-101	5
Pdlim1	2.92E-60	########	0.817	0.25	4.88E-56	5
Ece1	9.51E-96	########	0.817	0.176	1.59E-91	5
Fnbp1l	4.16E-118	########	0.767	0.134	6.97E-114	5
Psmd4	1.11E-25	########	0.961	0.7	1.86E-21	5
Erdr1	4.29E-36	########	0.911	0.409	7.18E-32	5
Aadac	0	########	0.317	0.001	0	5
Kank1	0	########	0.583	0.022	0	5
Dynll2	1.21E-45	########	0.828	0.306	2.03E-41	5
Bzw2	3.15E-56	########	0.817	0.258	5.27E-52	5
Rps14	6.06E-21	########	1	0.984	1.01E-16	5
Sptssa	1.92E-26	########	0.944	0.571	3.22E-22	5
Rab18	6.42E-32	########	0.917	0.472	1.07E-27	5

Rcn1	1.75E-103	########	0.822	0.161	2.93E-99	5
Myo10	4.37E-152	########	0.767	0.108	7.32E-148	5
Fam83b	0	########	0.622	0.001	0	5
Wdr74	1.12E-74	########	0.767	0.201	1.87E-70	5
Mbd3	1.42E-38	########	0.889	0.395	2.38E-34	5
Ier2	5.04E-20	########	0.956	0.69	8.43E-16	5
Ormdl2	2.65E-57	########	0.867	0.304	4.44E-53	5
1500011K1	2.29E-57	########	0.894	0.303	3.84E-53	5
Eif4b	6.27E-26	########	0.956	0.568	1.05E-21	5
Hddc2	4.00E-81	########	0.789	0.201	6.70E-77	5
Txnl1	3.06E-29	########	0.972	0.579	5.12E-25	5
Abce1	8.90E-37	########	0.789	0.325	1.49E-32	5
Cenpb	1.70E-33	########	0.922	0.456	2.84E-29	5
Cyb5a	2.13E-30	########	0.956	0.563	3.56E-26	5
Rnf7	6.80E-26	########	0.944	0.621	1.14E-21	5
Psma6	1.60E-23	########	0.967	0.672	2.67E-19	5
Tpm4	6.28E-20	########	0.983	0.668	1.05E-15	5
Dusp22	3.78E-80	########	0.761	0.178	6.32E-76	5
Sepw1	2.63E-22	########	0.978	0.668	4.40E-18	5
Rab38	0	########	0.717	0.028	0	5
Ddx27	1.87E-50	########	0.794	0.28	3.12E-46	5
Nudt21	9.83E-30	########	0.933	0.499	1.64E-25	5
Prkrir	3.11E-61	########	0.8	0.245	5.21E-57	5
Chl1	3.82E-270	########	0.728	0.052	6.38E-266	5
Wdr43	5.29E-49	########	0.867	0.315	8.85E-45	5
Suclg2	7.66E-71	########	0.817	0.229	1.28E-66	5
Taf10	2.67E-18	########	0.967	0.745	4.47E-14	5
Gtf2h5	3.96E-26	########	0.961	0.65	6.62E-22	5
Ppid	3.06E-41	########	0.789	0.307	5.11E-37	5
Amd1	4.30E-37	########	0.772	0.34	7.20E-33	5
Ndufa8	2.19E-29	########	0.978	0.705	3.66E-25	5
Dusp6	8.28E-20	########	0.578	0.246	1.38E-15	5
Nqo1	1.39E-270	########	0.611	0.038	2.33E-266	5
Shmt1	7.93E-194	########	0.606	0.056	1.33E-189	5
Evpl	0	########	0.578	0.003	0	5
Prkcdbp	1.12E-103	########	0.817	0.144	1.88E-99	5
Rrs1	1.04E-60	########	0.7	0.194	1.74E-56	5
Sgta	9.77E-46	########	0.894	0.372	1.63E-41	5
Mettl1	1.35E-82	########	0.767	0.18	2.25E-78	5
Prkar2a	5.93E-53	########	0.772	0.262	9.92E-49	5
Etv4	0	########	0.633	0.011	0	5
Ubxn1	2.48E-26	########	0.983	0.75	4.15E-22	5
Ddb1	4.20E-36	########	0.928	0.414	7.02E-32	5
Tipin	6.29E-45	########	0.689	0.224	1.05E-40	5
Ndufa9	4.27E-35	########	0.906	0.446	7.14E-31	5
Eif3e	6.85E-21	########	0.994	0.729	1.15E-16	5
Acot1	5.20E-68	########	0.478	0.089	8.71E-64	5
Mrps17	1.00E-33	########	0.894	0.449	1.68E-29	5
Mrpl52	1.38E-18	########	0.994	0.708	2.31E-14	5
Adgra3	3.89E-121	########	0.433	0.045	6.51E-117	5
Rac3	9.00E-154	########	0.644	0.076	1.51E-149	5
Arap2	7.57E-115	########	0.778	0.133	1.27E-110	5

Rrp15	5.07E-68	########	0.75	0.202	8.49E-64	5
Fat1	8.99E-201	########	0.672	0.064	1.50E-196	5
Mtx2	2.69E-49	########	0.839	0.312	4.50E-45	5
Eif4e2	1.22E-27	########	0.933	0.595	2.04E-23	5
Rpl35	2.52E-20	0.422836	1	0.905	4.22E-16	5
Rps19bp1	7.44E-51	########	0.822	0.296	1.24E-46	5
Cops7a	4.75E-70	########	0.811	0.231	7.96E-66	5
Crb3	0	########	0.55	0.003	0	5
Ssr4	2.07E-21	########	0.994	0.768	3.46E-17	5
Ctnnal1	9.35E-204	########	0.483	0.032	1.56E-199	5
Ccdc58	4.91E-77	########	0.75	0.189	8.21E-73	5
Polr2h	2.29E-52	########	0.828	0.289	3.83E-48	5
Psmc2	3.77E-26	########	0.894	0.528	6.30E-22	5
Poldip2	9.67E-76	########	0.794	0.206	1.62E-71	5
Bmpr1a	3.52E-124	########	0.694	0.107	5.89E-120	5
Cox6c	6.34E-22	########	1	0.912	1.06E-17	5
Cd2ap	1.32E-39	########	0.922	0.393	2.21E-35	5
Krt1	1.83E-134	########	0.106	0.001	3.06E-130	5
Med19	8.21E-77	########	0.789	0.211	1.37E-72	5
Ifi202b	0	########	0.394	0.002	0	5
Pkp4	5.17E-140	########	0.722	0.104	8.65E-136	5
Acsl3	1.43E-82	########	0.661	0.137	2.39E-78	5
Pou3f1	8.23E-198	########	0.55	0.043	1.38E-193	5
Fasn	1.61E-80	########	0.644	0.131	2.69E-76	5
Pdcd6	1.59E-28	########	0.961	0.66	2.66E-24	5
Tyms	7.40E-57	########	0.722	0.199	1.24E-52	5
Mrpl41	8.73E-39	########	0.878	0.385	1.46E-34	5
Rpl23	1.71E-21	########	1	0.984	2.87E-17	5
Stra13	6.86E-26	########	0.95	0.552	1.15E-21	5
Ptgfrn	3.48E-87	########	0.656	0.122	5.83E-83	5
Nifk	3.29E-58	########	0.822	0.257	5.51E-54	5
Mrps28	2.96E-37	########	0.867	0.365	4.96E-33	5
Psmd2	3.56E-26	########	0.928	0.528	5.96E-22	5
Camsap2	9.71E-75	########	0.672	0.152	1.63E-70	5
Fbln1	2.84E-216	########	0.744	0.07	4.75E-212	5
Mrps12	5.35E-34	########	0.917	0.449	8.96E-30	5
Ubl4a	7.98E-43	########	0.85	0.347	1.33E-38	5
Prep	3.93E-85	########	0.722	0.165	6.57E-81	5
Jagn1	1.79E-57	########	0.806	0.256	2.99E-53	5
Igsf8	5.14E-59	########	0.783	0.232	8.61E-55	5
Cops6	2.21E-25	########	0.889	0.521	3.70E-21	5
Smo	1.55E-176	########	0.694	0.079	2.59E-172	5
2310039H0	1.58E-49	########	0.828	0.283	2.65E-45	5
mt-Co2	2.36E-12	########	0.989	0.989	3.95E-08	5
1110004F1	3.22E-24	########	0.961	0.578	5.39E-20	5
Ptrf	6.48E-107	########	0.822	0.149	1.08E-102	5
Eef1e1	2.94E-61	########	0.767	0.227	4.93E-57	5
Csnk2a2	3.57E-45	########	0.867	0.349	5.98E-41	5
Tceb2	1.21E-23	########	1	0.908	2.03E-19	5
Erbb3	0	########	0.661	0.022	0	5
Aldh3b2	0	########	0.311	0.004	0	5
Ppp2r3a	8.93E-110	########	0.583	0.087	1.49E-105	5

Eif3j1	9.83E-28	########	0.939	0.521	1.65E-23	5
Dph3	3.21E-25	########	0.917	0.565	5.37E-21	5
Tnks1bp1	1.05E-119	########	0.717	0.119	1.76E-115	5
Zfp326	4.81E-47	########	0.889	0.334	8.04E-43	5
Gjb4	0	########	0.461	0.008	0	5
Nhsl1	4.82E-297	########	0.522	0.024	8.07E-293	5
Hmgn5	3.14E-36	########	0.822	0.328	5.26E-32	5
Flrt3	1.24E-177	########	0.533	0.043	2.07E-173	5
Papss1	2.43E-57	########	0.778	0.242	4.07E-53	5
Cox6a1	3.59E-21	########	1	0.832	6.00E-17	5
Tceal8	7.30E-52	########	0.828	0.262	1.22E-47	5
Rps16	1.81E-22	########	1	0.989	3.03E-18	5
Gmnn	6.02E-40	########	0.7	0.242	1.01E-35	5
Ide	6.90E-49	########	0.728	0.236	1.15E-44	5
Sdr16c5	0	########	0.478	0.001	0	5
Efna5	0	########	0.656	0.019	0	5
Tpm1	2.65E-55	########	0.822	0.275	4.44E-51	5
Ptbp1	2.04E-28	########	0.922	0.504	3.41E-24	5
Mrpl36	5.23E-28	########	0.911	0.488	8.74E-24	5
Gpr87	0	########	0.644	0	0	5
Fam46b	0	########	0.539	0.008	0	5
Psmd11	2.99E-25	########	0.917	0.543	5.00E-21	5
Rab34	5.27E-126	########	0.717	0.105	8.82E-122	5
Adrm1	2.50E-35	########	0.856	0.387	4.18E-31	5
Zfp91	4.18E-22	########	0.933	0.625	6.99E-18	5
Ctnnd1	2.31E-82	########	0.822	0.201	3.87E-78	5
Naa38	4.04E-30	########	0.9	0.474	6.76E-26	5
Pfdn4	6.24E-36	########	0.789	0.336	1.04E-31	5
Pdha1	3.23E-29	########	0.894	0.446	5.40E-25	5
Arl6ip4	2.23E-36	########	0.911	0.424	3.73E-32	5
Ccdc124	1.09E-25	########	0.867	0.467	1.82E-21	5
Aig1	2.56E-88	########	0.783	0.172	4.28E-84	5
Adgrg1	5.91E-270	########	0.733	0.053	9.89E-266	5
Sh2d5	1.82E-139	########	0.422	0.036	3.05E-135	5
Serpinb11	2.51E-185	########	0.117	0	4.19E-181	5
Rpl30	8.07E-25	########	1	0.976	1.35E-20	5
Tars	5.96E-54	########	0.811	0.256	9.97E-50	5
Acad9	2.63E-102	########	0.617	0.103	4.40E-98	5
Rab10	2.56E-21	########	0.956	0.634	4.28E-17	5
Wdr83os	2.96E-25	########	0.967	0.592	4.95E-21	5
Vps36	6.13E-27	########	0.933	0.512	1.03E-22	5
Mycbp	9.03E-48	########	0.789	0.281	1.51E-43	5
Cs	2.87E-31	########	0.856	0.415	4.80E-27	5
Fam136a	2.53E-81	########	0.706	0.159	4.23E-77	5
Timm10	7.25E-46	########	0.728	0.245	1.21E-41	5
Slirp	7.18E-28	########	0.917	0.492	1.20E-23	5
Hoxa9	0	########	0.617	0.033	0	5
Vcp	4.01E-19	########	0.983	0.721	6.70E-15	5
Tenm2	0	########	0.556	0.004	0	5
Tmem79	0	########	0.622	0.019	0	5
Gmps	1.53E-37	########	0.872	0.37	2.55E-33	5
Slc25a48	0	########	0.539	0.001	0	5

Larp1	4.41E-32	########	0.856	0.398	7.38E-28	5
Atic	8.33E-38	########	0.667	0.24	1.39E-33	5
Fdft1	9.12E-61	########	0.556	0.129	1.53E-56	5
Polr3g	4.72E-222	########	0.644	0.054	7.90E-218	5
Utp14b	3.89E-47	########	0.456	0.104	6.51E-43	5
Ank	2.09E-43	0.38849	0.444	0.107	3.50E-39	5
Pno1	9.59E-58	########	0.761	0.228	1.60E-53	5
Rangrf	7.47E-107	########	0.633	0.106	1.25E-102	5
Esf1	3.83E-38	########	0.85	0.351	6.41E-34	5
Ccnb2	1.42E-46	########	0.617	0.173	2.37E-42	5
Lancl1	3.45E-105	########	0.656	0.114	5.78E-101	5
Mrpl32	1.31E-35	########	0.9	0.423	2.19E-31	5
2200002D(	4.00E-176	########	0.578	0.051	6.70E-172	5
Cwh43	0	########	0.478	0.001	0	5
0610012G(	3.09E-23	########	0.894	0.542	5.17E-19	5
Ahcyl2	4.72E-47	########	0.794	0.274	7.90E-43	5
Igsf3	3.53E-243	########	0.628	0.046	5.90E-239	5
Fat2	0	########	0.628	0.001	0	5
Trip6	7.83E-117	########	0.717	0.118	1.31E-112	5
Naa15	3.24E-34	########	0.878	0.405	5.43E-30	5
Lage3	1.68E-39	########	0.894	0.382	2.80E-35	5
Pcyox1	2.22E-51	########	0.756	0.245	3.71E-47	5
Impad1	3.52E-47	########	0.822	0.283	5.89E-43	5
Ccna2	4.90E-53	########	0.556	0.134	8.19E-49	5
Pam16	9.82E-45	########	0.906	0.346	1.64E-40	5
Efna1	2.86E-292	########	0.622	0.035	4.79E-288	5
Cadm1	0	########	0.556	0.011	0	5
Smarcc1	7.87E-34	########	0.828	0.345	1.32E-29	5
Mrps18b	9.56E-61	########	0.744	0.212	1.60E-56	5
Psmb1	9.98E-19	########	0.978	0.834	1.67E-14	5
Polr2i	2.29E-30	########	0.9	0.469	3.83E-26	5
Ruvbl1	1.69E-35	########	0.744	0.3	2.83E-31	5
Hebp2	1.08E-176	########	0.483	0.038	1.82E-172	5
Pak6	0	########	0.589	0.001	0	5
Dynlrb1	2.61E-22	########	0.967	0.675	4.36E-18	5
Sae1	9.45E-45	########	0.833	0.299	1.58E-40	5
Eif1a	3.54E-26	########	0.894	0.465	5.92E-22	5
Ruvbl2	2.96E-46	########	0.722	0.244	4.96E-42	5
Ddx46	4.74E-23	########	0.917	0.551	7.92E-19	5
Cinp	2.20E-78	########	0.744	0.185	3.68E-74	5
Zfp750	0	########	0.411	0.001	0	5
Setd8	1.30E-24	########	0.889	0.495	2.18E-20	5
Stub1	2.88E-28	########	0.922	0.473	4.82E-24	5
Anp32e	2.76E-20	########	0.861	0.464	4.61E-16	5
Hacd2	5.16E-18	########	0.756	0.36	8.64E-14	5
Clic3	0	########	0.294	0.001	0	5
Snai2	8.56E-266	########	0.506	0.025	1.43E-261	5
2700060E(	9.81E-19	########	0.967	0.682	1.64E-14	5
Ovol1	0	########	0.478	0.001	0	5
Mrpl15	1.30E-29	########	0.883	0.425	2.17E-25	5
Gltscr2	1.19E-18	########	0.978	0.703	1.99E-14	5
Tenm4	0	########	0.561	0.005	0	5

Ppil1	1.96E-60	########	0.767	0.228	3.28E-56	5
Mpp7	1.50E-97	########	0.672	0.126	2.50E-93	5
Cpox	3.22E-99	########	0.683	0.129	5.39E-95	5
Phlda1	3.92E-13	########	0.661	0.341	6.56E-09	5
Pank1	7.14E-94	########	0.356	0.037	1.19E-89	5
Cxcl14	3.87E-135	########	0.778	0.118	6.48E-131	5
Mtdh	4.10E-16	########	0.983	0.654	6.86E-12	5
Pawr	0	########	0.639	0.017	0	5
Thop1	6.31E-115	########	0.683	0.113	1.06E-110	5
Atp6v0a4	1.88E-252	########	0.283	0.006	3.15E-248	5
Scap	2.58E-113	########	0.772	0.143	4.32E-109	5
Gars	1.06E-37	########	0.85	0.335	1.78E-33	5
Unc45a	3.42E-91	########	0.728	0.149	5.72E-87	5
Slc6a8	1.20E-95	########	0.761	0.15	2.01E-91	5
Gpatch4	1.69E-64	########	0.689	0.174	2.83E-60	5
Mpc2	2.74E-16	########	0.972	0.719	4.58E-12	5
Ubap2	1.48E-49	########	0.733	0.24	2.48E-45	5
Gtpbp4	6.01E-34	########	0.828	0.359	1.01E-29	5
Acpp	2.61E-81	########	0.572	0.103	4.37E-77	5
Pard3	5.66E-244	########	0.678	0.053	9.47E-240	5
BC003965	2.38E-54	########	0.794	0.263	3.98E-50	5
Amotl1	2.38E-157	########	0.7	0.086	3.99E-153	5
Ddx1	9.25E-47	########	0.861	0.306	1.55E-42	5
Letm1	4.30E-47	########	0.806	0.297	7.19E-43	5
Txnrd3	5.30E-285	########	0.633	0.038	8.87E-281	5
Cep170b	0	########	0.656	0.028	0	5
Pfkp	3.42E-38	########	0.883	0.38	5.72E-34	5
Tle1	1.67E-68	########	0.55	0.116	2.80E-64	5
Mak16	6.53E-37	########	0.783	0.312	1.09E-32	5
Taf1d	4.66E-29	########	0.933	0.457	7.79E-25	5
Lsm8	1.28E-28	########	0.883	0.423	2.14E-24	5
Bmpr2	6.18E-41	########	0.861	0.315	1.03E-36	5
Timm9	1.05E-66	########	0.694	0.176	1.76E-62	5
Dnajc21	4.96E-38	########	0.811	0.33	8.30E-34	5
Mrpl27	1.59E-34	########	0.861	0.379	2.66E-30	5
Hat1	1.41E-33	########	0.767	0.31	2.36E-29	5
Fkbp2	4.18E-20	########	0.961	0.557	7.00E-16	5
Epha4	1.71E-268	########	0.472	0.021	2.85E-264	5
Slc7a5	3.23E-91	########	0.661	0.127	5.40E-87	5
Echs1	3.11E-32	########	0.872	0.383	5.21E-28	5
Hmmr	2.97E-41	########	0.472	0.12	4.97E-37	5
Cenpe	5.88E-25	########	0.417	0.139	9.83E-21	5
Tubb6	1.93E-18	########	0.756	0.378	3.22E-14	5
Rps27a	1.99E-18	########	1	0.989	3.33E-14	5
Ube2n	3.21E-20	########	0.95	0.638	5.37E-16	5
Glod4	1.01E-33	########	0.878	0.405	1.70E-29	5
Ppp1r13l	0	########	0.633	0.023	0	5
Bnc1	0	########	0.528	0	0	5
Etl4	3.20E-283	########	0.561	0.029	5.35E-279	5
Suclg1	4.52E-24	########	0.9	0.527	7.57E-20	5
Canx	3.14E-13	########	0.967	0.671	5.26E-09	5
Cdc42ep5	5.64E-112	########	0.639	0.096	9.44E-108	5

Cetn3	5.74E-27	########	0.961	0.51	9.61E-23	5
Rpl7l1	4.01E-25	########	0.872	0.448	6.70E-21	5
Gna11	3.81E-65	########	0.739	0.192	6.37E-61	5
Exosc8	2.24E-30	########	0.806	0.354	3.75E-26	5
Cdc34	8.63E-29	########	0.839	0.412	1.44E-24	5
Pgrmc2	3.26E-43	########	0.778	0.275	5.46E-39	5
Tpx2	3.36E-38	########	0.511	0.144	5.62E-34	5
Fam96b	9.90E-31	########	0.883	0.416	1.66E-26	5
Grwd1	1.33E-99	########	0.572	0.092	2.23E-95	5
Shtn1	4.51E-81	########	0.783	0.167	7.55E-77	5
Chchd3	2.31E-36	########	0.867	0.375	3.86E-32	5
Noc2l	2.56E-36	########	0.728	0.281	4.29E-32	5
Srsf9	4.87E-19	########	0.922	0.599	8.14E-15	5
Me1	3.30E-60	########	0.356	0.056	5.53E-56	5
Hook1	0	########	0.6	0.024	0	5
Lhx2	1.05E-294	########	0.283	0.005	1.76E-290	5
Timm22	2.89E-45	########	0.822	0.307	4.83E-41	5
Adh5	2.51E-23	########	0.878	0.485	4.20E-19	5
Guk1	4.62E-32	########	0.839	0.374	7.73E-28	5
Slc16a1	7.74E-46	########	0.583	0.163	1.30E-41	5
Zc3h15	4.86E-22	########	0.939	0.559	8.13E-18	5
Dennd2c	1.34E-276	########	0.533	0.027	2.24E-272	5
Bccip	4.02E-35	########	0.822	0.354	6.73E-31	5
Tmem65	1.78E-51	########	0.817	0.266	2.98E-47	5
Ap1m2	0	########	0.644	0.006	0	5
Hspa8	6.94E-12	########	0.994	0.943	1.16E-07	5
Ajuba	0	########	0.572	0.02	0	5
Vdac2	1.13E-15	########	0.967	0.758	1.89E-11	5
Adtrp	2.74E-170	########	0.444	0.031	4.58E-166	5
Eif2s3x	3.69E-34	########	0.822	0.354	6.17E-30	5
Cenpm	2.32E-69	########	0.589	0.129	3.88E-65	5
Ilf2	2.90E-36	########	0.8	0.313	4.85E-32	5
Map2k2	4.12E-21	########	0.933	0.623	6.90E-17	5
Pdgfa	8.63E-109	########	0.683	0.112	1.44E-104	5
Mark3	3.10E-29	########	0.8	0.371	5.18E-25	5
Mrps22	4.02E-77	########	0.717	0.172	6.73E-73	5
Pgrmc1	9.76E-41	########	0.85	0.318	1.63E-36	5
Eif3h	8.43E-17	########	0.989	0.8	1.41E-12	5
Uba2	4.01E-27	########	0.861	0.424	6.72E-23	5
Ptrh2	3.86E-67	########	0.778	0.211	6.46E-63	5
Mrpl2	8.84E-38	########	0.8	0.325	1.48E-33	5
Tmem109	6.82E-34	########	0.772	0.284	1.14E-29	5
Srp19	3.72E-19	########	0.939	0.581	6.22E-15	5
E130012A1	9.12E-215	########	0.567	0.042	1.53E-210	5
Has3	7.68E-292	########	0.311	0.006	1.28E-287	5
Mrps10	1.62E-48	########	0.761	0.253	2.72E-44	5
Fdx1l	2.41E-44	########	0.861	0.303	4.04E-40	5
Atp5h	1.65E-15	########	1	0.91	2.75E-11	5
Exosc4	2.83E-43	########	0.839	0.321	4.74E-39	5
Rars	2.85E-30	########	0.839	0.387	4.77E-26	5
Cluh	9.74E-81	########	0.689	0.151	1.63E-76	5
Edf1	1.08E-16	########	0.983	0.803	1.81E-12	5

Cnih1	6.77E-36	########	0.889	0.38	1.13E-31	5
Lig1	2.98E-39	########	0.644	0.206	4.99E-35	5
Emc8	1.41E-33	########	0.794	0.34	2.36E-29	5
Tmem63b	1.50E-91	########	0.75	0.153	2.51E-87	5
Wbp5	2.10E-24	########	0.867	0.418	3.52E-20	5
Smc1a	3.86E-14	########	0.911	0.599	6.46E-10	5
Kcnk7	0	########	0.544	0.007	0	5
Fgfr2	0	########	0.511	0.016	0	5
Map7	0	########	0.617	0.03	0	5
Tiam1	1.14E-40	########	0.722	0.249	1.91E-36	5
Rad23b	9.19E-28	########	0.889	0.44	1.54E-23	5
Mark4	1.67E-67	########	0.717	0.184	2.80E-63	5
1110001J0	3.42E-32	########	0.817	0.358	5.72E-28	5
Fam92a	1.02E-66	########	0.694	0.17	1.71E-62	5
Orc6	2.88E-50	########	0.739	0.228	4.81E-46	5
Nxt1	3.01E-46	########	0.794	0.271	5.04E-42	5
Ptgr1	1.71E-28	########	0.65	0.254	2.86E-24	5
Macf1	9.31E-10	########	0.906	0.675	1.56E-05	5
Manf	8.26E-17	########	0.933	0.596	1.38E-12	5
Tulp4	7.89E-36	########	0.844	0.332	1.32E-31	5
Rps15a	1.26E-19	########	1	0.952	2.11E-15	5
Eif4a3	2.73E-21	########	0.894	0.497	4.56E-17	5
Usmg5	3.42E-16	########	0.967	0.781	5.72E-12	5
Rps19	1.95E-14	########	1	0.944	3.26E-10	5
Chchd7	7.60E-39	########	0.839	0.329	1.27E-34	5
Srp9	1.07E-16	########	1	0.803	1.80E-12	5
Trmt6	1.45E-53	########	0.761	0.232	2.42E-49	5
Fjx1	2.54E-187	########	0.467	0.032	4.25E-183	5
Smc3	8.76E-20	########	0.917	0.529	1.47E-15	5
Swi5	4.98E-19	########	0.983	0.688	8.34E-15	5
Nenf	3.77E-48	########	0.933	0.327	6.31E-44	5
Pbk	9.31E-51	########	0.478	0.11	1.56E-46	5
Erf	4.04E-62	########	0.739	0.199	6.76E-58	5
Stfa2	1.76E-101	########	0.333	0.03	2.94E-97	5
Ipo5	3.24E-44	########	0.8	0.274	5.42E-40	5
Yif1a	4.46E-38	########	0.839	0.324	7.47E-34	5
9130401M(	8.11E-39	########	0.739	0.273	1.36E-34	5
Srsf6	7.92E-20	########	0.922	0.579	1.33E-15	5
Sltm	7.42E-24	########	0.917	0.498	1.24E-19	5
Cds1	9.60E-137	########	0.55	0.06	1.61E-132	5
Trp53i11	1.18E-155	########	0.639	0.069	1.97E-151	5
Rps7	8.55E-20	########	1	0.948	1.43E-15	5
Cbr1	1.46E-50	########	0.661	0.184	2.44E-46	5
Sumo3	3.73E-26	########	0.911	0.457	6.25E-22	5
Supt16	1.34E-19	########	0.9	0.525	2.25E-15	5
Tomm70a	3.56E-29	########	0.783	0.353	5.96E-25	5
Mtx1	8.42E-47	########	0.822	0.292	1.41E-42	5
Bmp7	0	########	0.467	0.005	0	5
2010107E(	5.06E-16	########	0.989	0.814	8.47E-12	5
Hspb8	3.71E-115	########	0.572	0.077	6.21E-111	5
Tspan9	5.32E-215	########	0.706	0.064	8.90E-211	5
Nedd8	5.98E-16	########	0.983	0.844	1.00E-11	5

Cyb561	2.25E-276	########	0.606	0.035	3.76E-272	5
Nfe2l1	7.48E-23	########	0.844	0.414	1.25E-18	5
Daam1	8.05E-82	########	0.744	0.166	1.35E-77	5
Dkk3	2.43E-117	########	0.389	0.036	4.06E-113	5
Mydgf	9.01E-23	########	0.928	0.509	1.51E-18	5
Slc25a13	1.13E-124	########	0.611	0.083	1.89E-120	5
Nr2f6	6.72E-70	########	0.744	0.186	1.12E-65	5
Endod1	7.66E-70	########	0.644	0.143	1.28E-65	5
Foxc1	6.65E-111	########	0.422	0.046	1.11E-106	5
Chchd1	2.75E-19	########	0.911	0.537	4.60E-15	5
Dlk2	0	########	0.372	0.002	0	5
Sar1b	4.18E-24	########	0.883	0.472	7.00E-20	5
Pycr2	3.14E-39	########	0.739	0.275	5.25E-35	5
Vangl1	2.22E-207	########	0.572	0.045	3.71E-203	5
Gm16286	2.07E-23	########	0.939	0.541	3.46E-19	5
Hsdl2	2.30E-49	########	0.633	0.182	3.85E-45	5
Nubp1	7.14E-28	########	0.872	0.412	1.20E-23	5
mt-Nd3	2.76E-09	########	0.989	0.843	4.62E-05	5
Tacc3	1.13E-43	########	0.583	0.167	1.90E-39	5
Myo18a	1.17E-59	########	0.744	0.209	1.96E-55	5
Ripk4	0	########	0.572	0.001	0	5
Pfkl	4.78E-32	########	0.65	0.256	7.99E-28	5
Smim7	1.54E-29	########	0.9	0.447	2.57E-25	5
Mrpl14	6.17E-22	########	0.933	0.582	1.03E-17	5
Snrpa	5.30E-34	########	0.85	0.359	8.87E-30	5
H3f3b	1.19E-14	########	0.994	0.99	2.00E-10	5
AI661453	0	########	0.589	0.005	0	5
Psmc1	2.78E-29	########	0.867	0.406	4.65E-25	5
Srsf1	4.16E-23	########	0.889	0.458	6.96E-19	5
Stip1	8.33E-28	########	0.789	0.362	1.39E-23	5
Abhd17a	3.66E-23	########	0.9	0.5	6.13E-19	5
Rpl18a	2.67E-13	########	1	0.978	4.47E-09	5
Hist1h2ap	7.30E-19	########	0.556	0.243	1.22E-14	5
Nup43	6.24E-98	########	0.65	0.118	1.04E-93	5
Zfos1	1.53E-18	########	0.889	0.484	2.56E-14	5
Fzd6	0	########	0.55	0.023	0	5
Slc25a1	6.69E-37	########	0.572	0.176	1.12E-32	5
Epb41l4b	0	########	0.522	0.011	0	5
Tmigd1	0	########	0.356	0.006	0	5
Rxra	5.41E-82	########	0.678	0.139	9.05E-78	5
Aim1	2.80E-70	########	0.772	0.181	4.69E-66	5
Clock	1.66E-52	########	0.8	0.252	2.77E-48	5
Mrpl9	2.90E-44	########	0.844	0.31	4.86E-40	5
Siva1	1.78E-19	########	0.878	0.482	2.98E-15	5
Rpl37	1.28E-16	########	1	0.982	2.14E-12	5
Nudt1	7.13E-82	########	0.694	0.152	1.19E-77	5
Mapk13	1.95E-70	########	0.85	0.204	3.26E-66	5
Cspg4	1.81E-69	########	0.294	0.035	3.03E-65	5
Rrp1	6.61E-22	########	0.917	0.537	1.11E-17	5
Mpp6	9.32E-31	########	0.811	0.341	1.56E-26	5
F3	1.77E-43	########	0.344	0.068	2.97E-39	5
Hdac2	2.06E-44	########	0.817	0.284	3.45E-40	5

Pcbp1	2.39E-15	########	0.983	0.743	4.01E-11	5
Ociad2	0	########	0.583	0.022	0	5
Hadha	4.17E-18	########	0.889	0.528	6.99E-14	5
Nrip1	2.67E-34	########	0.778	0.299	4.47E-30	5
Ehd2	1.09E-103	########	0.717	0.121	1.83E-99	5
Fam120a	4.94E-20	########	0.856	0.458	8.27E-16	5
Pigf	2.38E-88	########	0.683	0.14	3.98E-84	5
Mrpl4	4.26E-28	########	0.856	0.408	7.13E-24	5
Lsm12	1.09E-18	0.318401	0.933	0.607	1.83E-14	5
Xrcc5	4.11E-163	########	0.65	0.073	6.88E-159	5
Paics	7.35E-27	########	0.861	0.389	1.23E-22	5
Sf3a3	1.91E-31	########	0.767	0.325	3.20E-27	5
Plxnb1	0	########	0.528	0.008	0	5
Pla2g2f	0	########	0.317	0.001	0	5
Dctn2	2.28E-21	########	0.928	0.567	3.81E-17	5
Ube2q2	2.16E-40	########	0.806	0.305	3.62E-36	5
Prom2	0	########	0.467	0.001	0	5
Hist1h4i	2.05E-39	########	0.656	0.221	3.43E-35	5
Abcf2	1.37E-40	########	0.711	0.255	2.30E-36	5
Snhg6	5.76E-35	########	0.844	0.336	9.63E-31	5
Tmpo	1.63E-17	########	0.844	0.456	2.73E-13	5
Lars	4.21E-31	########	0.839	0.365	7.04E-27	5
Hmbs	4.59E-43	########	0.794	0.279	7.67E-39	5
Auts2	8.97E-64	########	0.539	0.108	1.50E-59	5
Cmc2	2.31E-35	########	0.767	0.294	3.86E-31	5
Atad3a	8.52E-62	########	0.606	0.145	1.43E-57	5
Cdc123	1.14E-25	########	0.839	0.418	1.91E-21	5
Twf1	2.06E-25	########	0.828	0.395	3.45E-21	5
Lsr	1.03E-122	########	0.678	0.095	1.73E-118	5
Dnaja2	3.25E-18	########	0.928	0.622	5.43E-14	5
Ube2e1	1.32E-29	########	0.889	0.424	2.22E-25	5
Pigp	6.90E-50	########	0.8	0.255	1.15E-45	5
0610007P1	6.21E-32	########	0.789	0.329	1.04E-27	5
Chit1	2.70E-267	########	0.406	0.015	4.52E-263	5
Nppb	4.50E-280	########	0.172	0.001	7.52E-276	5
Yes1	7.07E-168	########	0.694	0.079	1.18E-163	5
Ttc39b	3.20E-46	########	0.794	0.263	5.35E-42	5
Rnaseh2a	1.10E-58	########	0.75	0.216	1.84E-54	5
Lsm7	4.06E-56	########	0.767	0.228	6.80E-52	5
Eif4h	2.57E-17	########	0.956	0.644	4.31E-13	5
Isoc1	2.82E-54	########	0.694	0.199	4.73E-50	5
Kpnb1	1.97E-24	########	0.867	0.429	3.29E-20	5
Ndufaf2	2.64E-51	########	0.761	0.233	4.42E-47	5
Capn2	1.12E-22	########	0.817	0.373	1.87E-18	5
Rala	1.35E-19	########	0.939	0.625	2.26E-15	5
Grhl1	0	########	0.444	0.004	0	5
Snrpc	2.43E-19	########	0.928	0.606	4.06E-15	5
Chmp2b	8.75E-37	########	0.878	0.367	1.46E-32	5
Rplp2	1.28E-18	########	1	0.972	2.14E-14	5
Pusl1	1.62E-77	########	0.65	0.14	2.71E-73	5
Echdc2	3.66E-276	########	0.539	0.028	6.12E-272	5
Mrpl43	5.50E-20	########	0.956	0.591	9.20E-16	5

Tinagl1	2.31E-39	########	0.444	0.112	3.87E-35	5
Acat1	2.30E-24	########	0.861	0.403	3.84E-20	5
Agrn	8.02E-153	########	0.472	0.04	1.34E-148	5
Cops2	1.22E-29	########	0.839	0.373	2.05E-25	5
Tdg	1.25E-44	########	0.733	0.253	2.10E-40	5
Commd1	4.58E-18	########	0.933	0.556	7.66E-14	5
Eps8l2	0	########	0.567	0.005	0	5
Pus1	4.95E-77	########	0.75	0.174	8.29E-73	5
Dnlz	4.50E-45	########	0.828	0.282	7.54E-41	5
St13	2.13E-16	########	0.928	0.606	3.56E-12	5
Usp10	1.14E-62	########	0.672	0.172	1.90E-58	5
Lrrc1	1.63E-210	########	0.533	0.037	2.73E-206	5
Tsfm	1.00E-48	########	0.761	0.246	1.67E-44	5
Slc38a2	3.08E-12	########	0.967	0.663	5.15E-08	5
C77080	0	########	0.583	0.02	0	5
Prlr	2.06E-193	########	0.194	0.003	3.45E-189	5
Tbx1	2.93E-302	########	0.3	0.005	4.90E-298	5
Smco4	4.01E-84	########	0.744	0.161	6.71E-80	5
Hsd17b7	2.56E-74	########	0.489	0.084	4.29E-70	5
Ndufaf4	2.17E-48	########	0.661	0.193	3.64E-44	5
Ppib	5.98E-16	########	0.989	0.839	1.00E-11	5
Igfbp3	1.78E-21	########	0.183	0.04	2.97E-17	5
Lgr4	1.13E-165	########	0.444	0.033	1.89E-161	5
Psmg1	3.40E-56	########	0.772	0.224	5.69E-52	5
Cbx5	4.03E-30	########	0.7	0.268	6.74E-26	5
Tuba1b	6.56E-10	########	0.867	0.596	1.10E-05	5
Ebpl	3.23E-52	0.304036	0.772	0.229	5.40E-48	5
Rhod	1.05E-262	########	0.55	0.031	1.76E-258	5
Vma21	1.35E-27	########	0.911	0.437	2.25E-23	5
Cebpzos	2.48E-39	########	0.822	0.311	4.15E-35	5
Sac3d1	2.48E-97	########	0.683	0.126	4.15E-93	5
Ralbp1	2.13E-09	########	0.95	0.657	3.56E-05	5
Psmd9	3.86E-35	########	0.811	0.334	6.45E-31	5
2810004N2	1.79E-39	########	0.772	0.281	3.00E-35	5
Atp5l	5.25E-15	########	0.994	0.952	8.78E-11	5
Carnmt1	8.89E-53	########	0.783	0.241	1.49E-48	5
Ppih	1.09E-41	########	0.728	0.249	1.82E-37	5
Rexo2	2.12E-19	########	0.917	0.502	3.54E-15	5
Aco2	3.81E-24	########	0.894	0.469	6.38E-20	5
Pomp	2.08E-14	########	0.994	0.813	3.47E-10	5
Tmem234	6.92E-17	########	0.972	0.75	1.16E-12	5
Reep3	1.38E-15	########	0.889	0.576	2.31E-11	5
Fam81a	0	########	0.417	0.005	0	5
Polr3k	1.35E-40	########	0.817	0.301	2.25E-36	5
Hnrnpm	2.48E-14	########	0.978	0.722	4.15E-10	5
Lman2	7.74E-15	########	0.928	0.658	1.29E-10	5
Ywhaz	1.10E-12	########	0.983	0.9	1.84E-08	5
Cisd3	1.93E-68	########	0.661	0.154	3.23E-64	5
Uchl5	2.79E-28	########	0.817	0.372	4.67E-24	5
Ezh2	1.75E-40	########	0.706	0.236	2.94E-36	5
Cenpv	9.34E-110	########	0.594	0.089	1.56E-105	5
Psmd14	9.81E-21	########	0.906	0.503	1.64E-16	5

Il17re	0	########	0.544	0.003	0	5
Pgls	2.23E-14	########	0.978	0.715	3.72E-10	5
Sptbn2	0	########	0.417	0.004	0	5
Itgb8	2.80E-208	########	0.433	0.024	4.68E-204	5
Dhrs4	1.21E-50	########	0.761	0.239	2.02E-46	5
Pex16	6.71E-57	########	0.694	0.188	1.12E-52	5
Cmtm4	6.40E-145	########	0.65	0.082	1.07E-140	5
Nop14	1.50E-40	########	0.739	0.253	2.52E-36	5
Sh3rf1	6.18E-140	########	0.506	0.051	1.03E-135	5
Serpinb3b	0	########	0.306	0	0	5
Galnt18	9.25E-248	########	0.5	0.026	1.55E-243	5
Clstn1	3.33E-69	########	0.528	0.102	5.58E-65	5
Itpkc	3.13E-80	########	0.506	0.084	5.24E-76	5
Uhrf1	1.52E-48	########	0.517	0.129	2.55E-44	5
2610528J1	0	########	0.489	0.001	0	5
Mrps36	1.42E-25	########	0.883	0.415	2.38E-21	5
Tmprss4	0	########	0.261	0.003	0	5
Mecr	1.60E-69	########	0.672	0.16	2.68E-65	5
Eno1	1.28E-09	########	0.967	0.797	2.14E-05	5
Wbscr22	9.46E-39	########	0.756	0.284	1.58E-34	5
Ndufb11	1.38E-11	########	0.989	0.824	2.30E-07	5
Gpsm2	3.83E-85	########	0.561	0.098	6.41E-81	5
Pdzk1ip1	2.36E-242	########	0.222	0.003	3.94E-238	5
Lpcat3	6.71E-24	########	0.672	0.285	1.12E-19	5
Sytl1	2.85E-192	########	0.522	0.039	4.77E-188	5
2-Mar	1.51E-20	########	0.956	0.511	2.52E-16	5
Chchd6	2.70E-86	########	0.639	0.124	4.51E-82	5
Ccar1	2.82E-21	########	0.889	0.469	4.71E-17	5
Cblc	0	########	0.55	0.001	0	5
Mrps35	1.11E-51	########	0.778	0.244	1.86E-47	5
Ckap2	4.18E-41	########	0.411	0.1	6.99E-37	5
Egfr	8.41E-143	########	0.694	0.087	1.41E-138	5
Mphosph1(	1.66E-33	########	0.811	0.317	2.77E-29	5
Tcerg1	3.86E-33	########	0.772	0.309	6.46E-29	5
Smarca5	2.15E-10	########	0.922	0.666	3.59E-06	5
Gm8730	5.33E-37	########	0.833	0.323	8.92E-33	5
Psma1	1.29E-14	########	0.956	0.686	2.16E-10	5
Nme2	7.33E-50	########	0.828	0.257	1.23E-45	5
Prkci	3.86E-93	########	0.617	0.107	6.46E-89	5
1110008P1	7.57E-13	########	0.8	0.47	1.27E-08	5
Shisa2	3.08E-115	########	0.178	0.007	5.15E-111	5
Rfc3	6.98E-38	########	0.606	0.195	1.17E-33	5
Pfdn2	1.58E-16	########	0.883	0.549	2.65E-12	5
Wdr46	4.39E-54	########	0.656	0.18	7.35E-50	5
Hprt	3.85E-17	########	0.9	0.542	6.44E-13	5
Ier5l	2.11E-76	########	0.617	0.124	3.53E-72	5
Mrpl11	7.59E-31	########	0.856	0.369	1.27E-26	5
Cav1	1.71E-85	########	0.744	0.147	2.87E-81	5
Dap3	8.37E-25	########	0.833	0.399	1.40E-20	5
Degs1	1.21E-15	########	0.939	0.595	2.03E-11	5
Esrp2	0	########	0.489	0.001	0	5
Tbc1d10a	6.93E-68	########	0.7	0.169	1.16E-63	5

Fam104a	1.56E-15	########	0.944	0.641	2.60E-11	5
Bdh1	0	########	0.583	0.021	0	5
Pacsin3	2.90E-224	########	0.528	0.034	4.86E-220	5
Grhl2	0	########	0.483	0.001	0	5
Celsr1	3.04E-278	########	0.5	0.023	5.09E-274	5
Nars	4.85E-15	########	0.911	0.607	8.11E-11	5
Eif2b1	1.23E-57	########	0.706	0.198	2.06E-53	5
Usp14	1.45E-26	########	0.806	0.365	2.43E-22	5
Nsfl1c	1.14E-35	########	0.85	0.34	1.91E-31	5
Mrpl40	1.29E-31	########	0.817	0.343	2.16E-27	5
Tanc2	8.33E-84	########	0.561	0.096	1.39E-79	5
Ak6	1.77E-32	########	0.839	0.345	2.95E-28	5
Slc38a4	9.11E-54	########	0.211	0.023	1.52E-49	5
Tfg	2.54E-29	########	0.833	0.372	4.25E-25	5
Prss12	0	########	0.428	0.008	0	5
Huwe1	7.24E-20	########	0.894	0.494	1.21E-15	5
Csnk2b	4.25E-16	########	0.956	0.662	7.12E-12	5
Il33	8.43E-13	########	0.2	0.063	1.41E-08	5
Polr2d	2.82E-51	########	0.711	0.216	4.72E-47	5
Galnt3	1.62E-187	########	0.483	0.034	2.71E-183	5
Fundc2	2.93E-21	########	0.944	0.487	4.91E-17	5
Notch1	6.68E-25	########	0.761	0.34	1.12E-20	5
1700021F0	4.93E-56	########	0.683	0.189	8.25E-52	5
4833423E2	0	########	0.4	0	0	5
Uck2	1.13E-35	########	0.761	0.287	1.89E-31	5
Rnps1	9.22E-24	########	0.828	0.402	1.54E-19	5
Rarg	2.46E-82	########	0.728	0.154	4.12E-78	5
Sucla2	3.56E-34	########	0.822	0.329	5.96E-30	5
Mtch2	2.15E-24	########	0.85	0.425	3.60E-20	5
Bop1	1.64E-53	########	0.717	0.202	2.74E-49	5
Rrp9	6.71E-54	########	0.589	0.152	1.12E-49	5
Ipo7	2.08E-25	########	0.794	0.359	3.47E-21	5
Erc1	2.32E-86	########	0.672	0.133	3.88E-82	5
Mrpl3	5.10E-44	########	0.8	0.279	8.54E-40	5
Sumo1	5.96E-15	########	0.978	0.719	9.97E-11	5
Eif3f	4.71E-12	########	0.989	0.861	7.89E-08	5
Tceb1	5.42E-15	########	0.972	0.706	9.07E-11	5
Gart	2.28E-38	########	0.678	0.235	3.82E-34	5
Adh7	1.71E-78	########	0.222	0.018	2.87E-74	5
Calr	1.40E-14	########	0.983	0.771	2.34E-10	5
Pgp	1.17E-22	########	0.867	0.435	1.96E-18	5
Ttll12	6.03E-107	########	0.594	0.09	1.01E-102	5
Mrpl22	5.00E-39	########	0.817	0.301	8.36E-35	5
Eif2b5	3.78E-48	########	0.728	0.233	6.33E-44	5
Bicd2	1.89E-41	########	0.656	0.207	3.17E-37	5
Aebp2	1.51E-31	########	0.794	0.329	2.53E-27	5
Bcap31	5.73E-13	########	0.944	0.636	9.59E-09	5
Cyb5b	6.06E-25	########	0.733	0.33	1.01E-20	5
2810417H1	4.56E-44	########	0.639	0.179	7.63E-40	5
Yae1d1	7.37E-95	########	0.711	0.134	1.23E-90	5
Trap1	2.92E-52	########	0.728	0.215	4.89E-48	5
Ldlrap1	9.90E-33	########	0.767	0.316	1.66E-28	5

A430005L1	7.58E-42	########	0.717	0.243	1.27E-37	5
Epha2	1.12E-133	########	0.467	0.045	1.87E-129	5
Smim13	1.01E-51	########	0.683	0.196	1.69E-47	5
Erbb2	8.30E-273	########	0.528	0.027	1.39E-268	5
Prelid2	2.48E-55	########	0.378	0.068	4.15E-51	5
Ptprs	2.40E-41	########	0.544	0.152	4.02E-37	5
Tardbp	8.58E-18	########	0.878	0.495	1.43E-13	5
Mrpl23	3.72E-16	########	0.9	0.565	6.22E-12	5
Pla2g12a	1.42E-54	########	0.606	0.155	2.37E-50	5
Ssfa2	4.75E-33	########	0.744	0.272	7.96E-29	5
Jtb	1.21E-19	########	0.917	0.524	2.03E-15	5
Tarbp2	9.04E-64	########	0.694	0.175	1.51E-59	5
Cntfr	1.10E-191	########	0.306	0.012	1.83E-187	5
Rabggtb	3.71E-45	########	0.756	0.248	6.21E-41	5
Kif5b	5.28E-10	########	0.944	0.711	8.84E-06	5
Ltb4r2	0	########	0.539	0.004	0	5
Uqcrh	2.38E-12	########	1	0.931	3.99E-08	5
Klk13	9.78E-258	########	0.172	0.001	1.64E-253	5
2610001J0	8.22E-24	########	0.878	0.442	1.38E-19	5
Ap1ar	6.83E-56	########	0.678	0.184	1.14E-51	5
Ndufs8	1.05E-16	########	0.933	0.575	1.75E-12	5
Dus1l	4.76E-47	########	0.672	0.208	7.96E-43	5
Hsd17b10	4.67E-20	########	0.928	0.498	7.82E-16	5
Smc4	1.23E-15	########	0.806	0.443	2.06E-11	5
Zcrb1	2.08E-13	########	0.911	0.611	3.48E-09	5
Hnrnpk	1.63E-11	########	0.994	0.869	2.73E-07	5
Slmo2	1.33E-27	########	0.794	0.359	2.23E-23	5
Sppl3	8.80E-34	########	0.789	0.314	1.47E-29	5
Camta1	4.11E-37	########	0.739	0.268	6.87E-33	5
Srsf10	1.16E-19	########	0.944	0.522	1.93E-15	5
Ept1	3.05E-61	########	0.65	0.158	5.10E-57	5
Gtf2a2	2.04E-17	########	0.9	0.533	3.42E-13	5
Prdx6	5.43E-15	########	0.961	0.728	9.08E-11	5
Elovl1	3.13E-16	########	0.75	0.399	5.23E-12	5
Prim1	1.55E-49	########	0.556	0.143	2.60E-45	5
Fam57a	1.37E-153	########	0.511	0.048	2.29E-149	5
Chmp4c	0	########	0.472	0.003	0	5
Hn1l	6.49E-62	########	0.694	0.179	1.09E-57	5
Uba52	1.98E-15	########	0.989	0.835	3.32E-11	5
Pvrl4	3.90E-103	########	0.339	0.031	6.53E-99	5
Ubac1	1.17E-67	########	0.717	0.179	1.96E-63	5
Tax1bp3	8.41E-36	########	0.822	0.316	1.41E-31	5
Cdkn3	6.50E-54	########	0.594	0.146	1.09E-49	5
Naa25	1.58E-50	########	0.628	0.172	2.64E-46	5
Porcn	5.36E-163	########	0.394	0.026	8.97E-159	5
Pir	8.67E-101	########	0.472	0.062	1.45E-96	5
Rrp7a	5.33E-38	########	0.756	0.27	8.91E-34	5
Grsf1	3.04E-37	########	0.75	0.275	5.08E-33	5
Gcsh	6.79E-39	########	0.806	0.293	1.14E-34	5
Tmem14c	3.01E-12	########	0.989	0.765	5.04E-08	5
Ssbp1	1.06E-19	########	0.872	0.464	1.78E-15	5
Mmadhc	2.92E-33	########	0.833	0.34	4.89E-29	5

Mcm5	7.53E-44	########	0.644	0.187	1.26E-39	5
Mcm7	1.47E-34	########	0.644	0.219	2.45E-30	5
Nr2c2ap	3.78E-57	########	0.728	0.202	6.32E-53	5
Dhrs7b	2.98E-44	########	0.694	0.22	4.98E-40	5
Rtkn	1.32E-278	########	0.539	0.027	2.21E-274	5
Kti12	1.46E-62	########	0.661	0.163	2.44E-58	5
Tcf12	5.59E-30	########	0.806	0.333	9.36E-26	5
Wtip	2.41E-100	########	0.6	0.095	4.04E-96	5
Ubtf	9.48E-20	########	0.883	0.473	1.59E-15	5
Iars2	5.33E-48	########	0.733	0.228	8.91E-44	5
Dsc2	0	########	0.317	0.001	0	5
Il1rl2	1.74E-173	########	0.572	0.053	2.92E-169	5
Tmem57	5.46E-39	########	0.728	0.252	9.14E-35	5
Kdelr1	4.33E-20	########	0.972	0.554	7.25E-16	5
Tmem33	2.19E-24	########	0.839	0.407	3.67E-20	5
Smyd2	1.61E-71	########	0.633	0.135	2.69E-67	5
Duoxa1	0	########	0.489	0.011	0	5
Rbm17	1.72E-20	########	0.828	0.43	2.87E-16	5
Itpa	1.31E-31	########	0.728	0.292	2.19E-27	5
Hypk	4.70E-22	########	0.867	0.47	7.86E-18	5
Nudt19	4.58E-40	########	0.65	0.207	7.66E-36	5
Top2b	6.39E-18	########	0.928	0.568	1.07E-13	5
Zdhhc5	1.06E-33	########	0.767	0.298	1.78E-29	5
Alad	3.44E-65	########	0.583	0.127	5.75E-61	5
Oaz1	1.07E-12	########	1	0.969	1.79E-08	5
Stard5	5.58E-36	########	0.55	0.168	9.33E-32	5
H13	4.16E-14	########	0.95	0.6	6.96E-10	5
Acadvl	6.60E-30	########	0.767	0.311	1.10E-25	5
Nono	1.14E-15	########	0.939	0.578	1.90E-11	5
Serpinb10	2.08E-72	########	0.472	0.078	3.48E-68	5
Cfap20	1.90E-35	########	0.756	0.283	3.18E-31	5
Tnfaip1	1.15E-57	########	0.756	0.203	1.92E-53	5
Ssx2ip	1.93E-54	########	0.456	0.098	3.22E-50	5
Polr1c	1.99E-41	########	0.778	0.266	3.34E-37	5
Ddx18	2.60E-28	########	0.833	0.357	4.34E-24	5
Igfbp2	1.03E-231	########	0.222	0.004	1.73E-227	5
Nsmce1	4.32E-18	########	0.817	0.454	7.22E-14	5
Dera	2.00E-32	########	0.8	0.332	3.35E-28	5
Ppm1g	8.73E-20	########	0.822	0.429	1.46E-15	5
St14	0	########	0.494	0.015	0	5
Rcc1	2.94E-68	########	0.617	0.137	4.93E-64	5
Mrpl50	6.38E-50	########	0.756	0.233	1.07E-45	5
Gps1	5.09E-31	########	0.794	0.319	8.51E-27	5
Mtap	1.57E-34	########	0.544	0.174	2.63E-30	5
Pold2	4.14E-51	########	0.55	0.14	6.92E-47	5
Gpaa1	4.14E-43	########	0.756	0.255	6.93E-39	5
Surf2	4.04E-65	########	0.683	0.167	6.76E-61	5
Pmf1	1.68E-38	########	0.739	0.263	2.82E-34	5
Cnpy2	9.66E-20	########	0.961	0.493	1.62E-15	5
Wasl	1.21E-47	########	0.756	0.231	2.02E-43	5
N6amt2	1.31E-29	########	0.772	0.314	2.19E-25	5
Tmem11	5.23E-36	########	0.794	0.304	8.74E-32	5

Sf3b6	2.67E-13	########	0.95	0.734	4.47E-09	5
Nudcd2	8.51E-30	########	0.811	0.329	1.42E-25	5
Sox15	0	########	0.439	0.001	0	5
Fer	4.77E-89	########	0.611	0.11	7.98E-85	5
Dlat	1.78E-44	########	0.7	0.223	2.98E-40	5
Wdr12	1.41E-37	########	0.65	0.217	2.36E-33	5
Anapc5	1.04E-18	########	0.911	0.472	1.74E-14	5
Fam60a	7.65E-59	########	0.633	0.156	1.28E-54	5
Naf1	4.73E-59	########	0.622	0.152	7.92E-55	5
G3bp1	1.50E-13	########	0.906	0.572	2.51E-09	5
C1d	2.43E-18	########	0.889	0.5	4.06E-14	5
Cad	1.07E-67	########	0.561	0.116	1.79E-63	5
Kars	2.65E-39	########	0.789	0.276	4.43E-35	5
Myo1e	7.13E-67	########	0.739	0.174	1.19E-62	5
Cbx3	3.99E-13	########	0.911	0.561	6.67E-09	5
Rfc4	7.47E-43	########	0.672	0.204	1.25E-38	5
Rpp25l	2.55E-34	########	0.717	0.267	4.27E-30	5
Hmgb3	3.40E-51	########	0.617	0.162	5.68E-47	5
Aprt	3.50E-10	########	0.967	0.736	5.86E-06	5
Gnl1	8.52E-42	########	0.739	0.246	1.42E-37	5
Bag4	1.24E-64	########	0.594	0.134	2.08E-60	5
Ict1	4.50E-20	########	0.889	0.458	7.53E-16	5
Vsnl1	0	########	0.428	0.002	0	5
Dusp14	3.57E-242	########	0.489	0.026	5.97E-238	5
Ube2a	4.64E-16	########	0.939	0.597	7.76E-12	5
Ppie	3.21E-40	########	0.706	0.237	5.37E-36	5
Ccdc59	4.59E-21	########	0.917	0.479	7.68E-17	5
Dapl1	2.69E-60	########	0.328	0.045	4.49E-56	5
Smim10l1	6.71E-30	########	0.744	0.3	1.12E-25	5
Tmem208	5.76E-17	########	0.939	0.559	9.64E-13	5
Clip4	1.06E-192	########	0.383	0.02	1.78E-188	5
Ptov1	1.52E-37	########	0.822	0.288	2.54E-33	5
Ak3	1.51E-36	########	0.567	0.178	2.52E-32	5
Ppan	5.71E-53	########	0.572	0.142	9.55E-49	5
Kif1c	3.15E-50	########	0.7	0.199	5.27E-46	5
Hdac1	9.39E-19	########	0.856	0.441	1.57E-14	5
Hars	1.31E-33	########	0.767	0.294	2.19E-29	5
Pon2	1.35E-21	########	0.894	0.453	2.26E-17	5
Abracl	6.21E-08	########	0.967	0.807	########	5
Ppdpf	1.82E-24	########	0.9	0.457	3.05E-20	5
Hspa1a	4.51E-75	########	0.728	0.16	7.55E-71	5
Sec11a	5.32E-14	########	0.933	0.601	8.90E-10	5
Rbmxl1	8.70E-24	########	0.867	0.404	1.46E-19	5
Get4	5.08E-44	########	0.772	0.26	8.50E-40	5
Phlda2	0	########	0.383	0.001	0	5
Aamp	2.45E-17	########	0.961	0.575	4.10E-13	5
Dtd1	4.36E-55	########	0.706	0.193	7.30E-51	5
Phyh	1.53E-19	########	0.789	0.397	2.57E-15	5
Zfp771	4.73E-41	########	0.711	0.232	7.92E-37	5
Gm42835	3.71E-173	########	0.172	0.003	6.21E-169	5
Mcm3	2.36E-22	########	0.572	0.231	3.94E-18	5
Epb41l4aos	1.98E-34	########	0.7	0.251	3.32E-30	5

Rce1	5.59E-51	########	0.733	0.216	9.35E-47	5
Hyal2	4.72E-81	########	0.589	0.109	7.89E-77	5
Fam114a2	2.59E-31	########	0.817	0.345	4.33E-27	5
Col7a1	0	########	0.339	0.007	0	5
Tufm	1.63E-58	########	0.706	0.187	2.73E-54	5
Anapc15	7.65E-32	########	0.75	0.298	1.28E-27	5
Bcl2l13	4.57E-54	########	0.611	0.157	7.64E-50	5
Dnajc19	4.85E-16	########	0.917	0.532	8.11E-12	5
Duox1	0	########	0.461	0.008	0	5
Efna3	0	########	0.344	0.002	0	5
Ccdc47	1.26E-17	########	0.833	0.441	2.10E-13	5
Plxna1	6.44E-81	########	0.572	0.102	1.08E-76	5
Retnlg	0	7.027208	0.928	0.129	0	6
S100a9	0	6.364169	0.984	0.457	0	6
S100a8	0	6.094122	0.987	0.489	0	6
Ngp	0	5.799484	0.656	0.068	0	6
Lcn2	0	5.162702	0.924	0.214	0	6
Camp	0	5.100973	0.55	0.094	0	6
Ltf	0	4.998485	0.487	0.028	0	6
Wfdc21	0	4.478568	0.93	0.169	0	6
Pglyrp1	0	3.734826	0.918	0.193	0	6
Gm5483	0	3.712264	0.314	0.02	0	6
Mmp9	0	3.478402	0.843	0.094	0	6
Ifitm6	0	3.462958	0.828	0.257	0	6
Stfa2l1	0	3.446249	0.398	0.014	0	6
Mmp8	0	3.233069	0.655	0.156	0	6
Cxcr2	0	3.185956	0.886	0.036	0	6
G0s2	0	3.183591	0.764	0.087	0	6
Slpi	0	3.095126	0.95	0.291	0	6
Ifitm1	1.75E-274	3.037799	0.609	0.267	2.93E-270	6
Acod1	0	3.029959	0.353	0.03	0	6
Il1r2	0	2.774752	0.79	0.184	0	6
Ly6g	0	2.750264	0.73	0.011	0	6
Hdc	0	2.664785	0.92	0.072	0	6
Asprv1	0	2.653514	0.584	0.019	0	6
Csf3r	0	2.649888	0.874	0.214	0	6
Anxa1	0	2.608441	0.898	0.672	0	6
Mxd1	0	2.554062	0.935	0.331	0	6
Lrg1	0	2.496654	0.923	0.253	0	6
Cd177	0	2.495187	0.68	0.101	0	6
Hp	0	2.470898	0.951	0.334	0	6
S100a11	0	2.272507	0.995	0.9	0	6
Il1b	4.22E-108	2.26858	0.48	0.284	7.07E-104	6
Grina	0	2.156859	0.901	0.511	0	6
Slc7a11	3.10E-114	2.144197	0.396	0.189	5.19E-110	6
Pygl	0	2.119019	0.885	0.333	0	6
F630028O1	0	2.060972	0.834	0.171	0	6
Clec4d	2.42E-202	2.060414	0.631	0.355	4.06E-198	6
Chil1	0	2.033704	0.788	0.019	0	6
Clec4e	0	2.03227	0.803	0.343	0	6
Mcemp1	0	2.017254	0.909	0.251	0	6
Slfn4	0	1.997388	0.743	0.06	0	6

Msrb1	0	1.967551	0.972	0.68	0	6
Sorl1	0	1.956084	0.933	0.365	0	6
Hmgn2	0	1.90709	0.774	0.5	0	6
Trem1	0	1.906471	0.628	0.153	0	6
Hcar2	3.93E-276	1.892198	0.296	0.039	6.58E-272	6
Fpr2	0	1.816379	0.794	0.105	0	6
Gsr	0	1.813677	0.969	0.5	0	6
Cd24a	0	1.813015	0.879	0.23	0	6
Cd33	0	1.811911	0.817	0.165	0	6
Wfdc17	3.78E-40	1.783162	0.61	0.575	6.33E-36	6
Prdx5	0	1.778243	0.981	0.832	0	6
Dhrs7	0	1.726665	0.885	0.484	0	6
Cxcl3	1.84E-15	1.704404	0.146	0.094	3.08E-11	6
Cxcl2	2.12E-07	1.687282	0.324	0.466	########	6
Nlrp3	4.37E-22	1.685745	0.384	0.329	7.31E-18	6
Srgn	2.40E-51	1.672139	0.789	0.844	4.02E-47	6
Chil3	5.13E-64	1.660876	0.516	0.377	8.59E-60	6
Tmcc1	0	1.64539	0.843	0.372	0	6
Il1f9	0	1.641726	0.632	0.031	0	6
Ncf1	0	1.635347	0.824	0.417	0	6
Lmnb1	1.35E-266	1.629123	0.786	0.449	2.26E-262	6
Lcp1	0	1.625324	0.983	0.756	0	6
Lyst	0	1.619993	0.853	0.299	0	6
Samsn1	0	1.61705	0.904	0.38	0	6
Hmgb2	0	1.612918	0.955	0.779	0	6
Serpinb1a	3.39E-168	1.603505	0.513	0.272	5.67E-164	6
Rdh12	0	1.601567	0.706	0.063	0	6
Trem3	0	1.595656	0.807	0.177	0	6
S100a6	5.03E-265	1.588964	0.946	0.815	8.41E-261	6
Adpgk	0	1.581807	0.598	0.18	0	6
AA467197	0	1.577752	0.633	0.08	0	6
Cd300lf	0	1.569379	0.827	0.195	0	6
Stfa1	2.34E-55	1.56622	0.122	0.034	3.91E-51	6
Fpr1	0	1.547187	0.615	0.045	0	6
Slfn1	0	1.544135	0.762	0.251	0	6
Trim30b	0	1.536442	0.622	0.088	0	6
Prr13	0	1.531674	0.869	0.656	0	6
Slc2a3	0	1.52595	0.718	0.06	0	6
Slc16a3	0	1.500925	0.839	0.337	0	6
Rac2	0	1.490158	0.94	0.677	0	6
Vasp	0	1.481948	0.917	0.668	0	6
Fcnb	1.34E-130	1.4519	0.267	0.077	2.24E-126	6
Plek	6.44E-130	1.451743	0.849	0.56	1.08E-125	6
Tnfaip2	4.21E-300	1.449765	0.854	0.459	7.05E-296	6
Dgat1	0	1.447875	0.778	0.202	0	6
Ccr1	0	1.445707	0.85	0.388	0	6
Stfa2	2.42E-88	1.431316	0.105	0.014	4.05E-84	6
Gda	0	1.43115	0.927	0.563	0	6
Glrx	0	1.422355	0.839	0.395	0	6
Cebpe	0	1.407444	0.439	0.056	0	6
Pilra	0	1.403431	0.844	0.291	0	6
Syne1	0	1.401969	0.6	0.135	0	6

Fam101b	0	1.394889	0.725	0.21	0	6
R3hdm4	0	1.383553	0.815	0.456	0	6
Sgms2	0	1.370698	0.699	0.265	0	6
Lilr4b	0	1.369368	0.811	0.356	0	6
Padi4	0	1.365963	0.691	0.062	0	6
Fbxl5	5.15E-303	1.365054	0.779	0.432	8.62E-299	6
Arg2	0	1.350919	0.508	0.142	0	6
Dhrs9	0	1.322781	0.574	0.035	0	6
Taldo1	0	1.320649	0.956	0.86	0	6
Mrgpra2b	0	1.304059	0.611	0.021	0	6
2810474O1	6.84E-150	1.292356	0.816	0.604	1.14E-145	6
Alox5ap	0	1.287132	0.973	0.604	0	6
Tnf	2.09E-06	1.271323	0.354	0.311	########	6
Selplg	6.26E-229	1.264555	0.866	0.568	1.05E-224	6
Zyx	0	1.248375	0.911	0.654	0	6
Prok2	0	1.236608	0.315	0.004	0	6
Gadd45a	0	1.231964	0.615	0.199	0	6
Il1rap	3.09E-179	1.225973	0.418	0.16	5.16E-175	6
Aldh2	3.27E-206	1.225179	0.749	0.667	5.47E-202	6
Igsf6	0	1.223422	0.883	0.444	0	6
Txn1	0	1.220939	0.967	0.877	0	6
Ltb4r1	0	1.213363	0.731	0.3	0	6
Slc40a1	0	1.206803	0.617	0.036	0	6
Cd52	0	1.204704	0.986	0.786	0	6
Arhgdib	0	1.20195	0.93	0.868	0	6
Gpsm3	0	1.19323	0.892	0.62	0	6
Itgb2l	0	1.190692	0.524	0.014	0	6
Cd9	0	1.18771	0.944	0.425	0	6
Lbr	0	1.185359	0.835	0.503	0	6
Ncf2	0	1.178675	0.921	0.549	0	6
Actn1	0	1.174098	0.822	0.343	0	6
Entpd1	3.58E-148	1.173506	0.567	0.299	5.99E-144	6
Nudt4	0	1.172887	0.843	0.416	0	6
Pgd	0	1.165491	0.842	0.582	0	6
Ostf1	0	1.161144	0.967	0.859	0	6
Sell	0	1.159159	0.877	0.357	0	6
Upp1	0	1.155303	0.464	0.084	0	6
Stx11	0	1.147703	0.674	0.198	0	6
Cybb	2.57E-80	1.13914	0.634	0.515	4.30E-76	6
Hist1h2ap	4.22E-36	1.126335	0.346	0.219	7.06E-32	6
B430306N(	0	1.109857	0.7	0.111	0	6
Mapk13	0	1.109103	0.637	0.085	0	6
Dstn	3.58E-194	1.108705	0.788	0.687	5.99E-190	6
Siglece	0	1.108073	0.708	0.108	0	6
Il1rn	4.37E-120	1.10546	0.412	0.177	7.31E-116	6
Pnkp	0	1.103572	0.716	0.335	0	6
Antxr2	2.81E-159	1.10193	0.64	0.357	4.69E-155	6
Glipr2	0	1.093305	0.792	0.337	0	6
Gmfg	0	1.087344	0.95	0.723	0	6
Cdk2ap2	2.90E-185	1.082097	0.866	0.719	4.86E-181	6
BC100530	2.09E-19	1.080934	0.14	0.079	3.50E-15	6
Tkt	6.35E-235	1.079066	0.795	0.748	1.06E-230	6

Rnf149	4.56E-52	1.074457	0.782	0.655	7.64E-48	6
Mgst1	1.27E-270	1.065973	0.766	0.443	2.13E-266	6
Il1a	1.61E-08	1.06161	0.103	0.071	########	6
Adam8	4.37E-299	1.058226	0.704	0.276	7.30E-295	6
Gcnt2	2.24E-192	1.056689	0.579	0.279	3.75E-188	6
Anxa11	0	1.053949	0.809	0.453	0	6
Lilrb4a	1.03E-264	1.047552	0.857	0.453	1.73E-260	6
Apobr	0	1.041352	0.738	0.256	0	6
Adipor1	5.34E-273	1.040937	0.884	0.639	8.93E-269	6
H2-Q10	2.73E-152	1.036273	0.299	0.089	4.57E-148	6
Pirb	0	1.033753	0.882	0.508	0	6
Igf1r	0	1.02425	0.691	0.298	0	6
Ceacam10	0	1.021797	0.472	0.006	0	6
Ogfrl1	0	1.019457	0.796	0.387	0	6
Itgam	0	1.014216	0.894	0.511	0	6
Atxn10	0	1.012842	0.783	0.472	0	6
Ncf4	0	1.009166	0.865	0.532	0	6
Il18rap	0	1.009083	0.666	0.104	0	6
Abtb1	0	1.007052	0.684	0.163	0	6
Ifitm2	1.12E-167	1.006807	0.979	0.777	1.88E-163	6
Fgr	0	1.000665	0.797	0.321	0	6
Ckap4	6.49E-220	########	0.717	0.483	1.09E-215	6
Plp2	0	########	0.81	0.469	0	6
Mettl9	3.94E-288	########	0.73	0.48	6.60E-284	6
Map1lc3b	6.04E-117	########	0.96	0.869	1.01E-112	6
G6pdx	1.11E-301	########	0.671	0.355	1.86E-297	6
Itgb2	0	########	0.898	0.566	0	6
2310001H1	0	########	0.682	0.226	0	6
Msra	0	########	0.705	0.215	0	6
Clec5a	0	########	0.647	0.186	0	6
Cyp4f18	8.75E-152	########	0.581	0.319	1.46E-147	6
Gpi1	0	########	0.889	0.824	0	6
Iqgap1	1.85E-253	########	0.973	0.895	3.09E-249	6
Dmxl2	0	########	0.599	0.199	0	6
Tyrobp	8.33E-241	########	0.988	0.669	1.39E-236	6
Stk17b	2.25E-140	########	0.869	0.701	3.76E-136	6
Spi1	6.92E-303	########	0.925	0.571	1.16E-298	6
Tpd52	1.34E-87	########	0.847	0.653	2.24E-83	6
Ankrd33b	4.84E-272	########	0.412	0.098	8.10E-268	6
Cap1	0	########	0.881	0.699	0	6
C5ar1	1.22E-297	########	0.854	0.369	2.04E-293	6
6430548M(	0	########	0.63	0.172	0	6
Cnn2	1.15E-300	########	0.824	0.593	1.92E-296	6
Myh9	1.54E-301	########	0.91	0.819	2.57E-297	6
Mcl1	2.91E-41	########	0.978	0.939	4.87E-37	6
Tmem154	0	########	0.629	0.116	0	6
Ly6c2	8.48E-269	########	0.711	0.281	1.42E-264	6
Lpcat2	0	########	0.708	0.27	0	6
Rasa2	5.50E-179	########	0.604	0.315	9.20E-175	6
St3gal5	0	########	0.534	0.164	0	6
Slfn2	1.23E-169	########	0.965	0.744	2.06E-165	6
Ptprc	1.37E-191	########	0.964	0.752	2.29E-187	6

Nfam1	0	########	0.719	0.281	0	6
Rnf144a	0	########	0.621	0.138	0	6
Arrb2	2.89E-283	0.888397	0.753	0.453	4.84E-279	6
Cpne3	2.38E-127	########	0.61	0.478	3.98E-123	6
Nfkbia	1.41E-16	########	0.871	0.859	2.36E-12	6
Gpcpd1	4.34E-99	########	0.603	0.398	7.27E-95	6
Amica1	9.00E-111	########	0.371	0.167	1.51E-106	6
Bmx	0	########	0.489	0.074	0	6
Rab11fip1	1.15E-188	########	0.556	0.232	1.93E-184	6
Mgst2	0	########	0.515	0.128	0	6
Alox5	0	########	0.625	0.088	0	6
Litaf	4.19E-127	########	0.868	0.65	7.02E-123	6
Pilrb2	0	########	0.669	0.157	0	6
Cd300ld	1.84E-125	########	0.565	0.321	3.07E-121	6
C3	1.22E-234	########	0.657	0.326	2.05E-230	6
Ccpg1	0	########	0.582	0.179	0	6
Fam65b	0	########	0.761	0.283	0	6
Sri	7.38E-269	########	0.853	0.803	1.23E-264	6
Plaur	6.90E-172	########	0.824	0.516	1.15E-167	6
Cpd	6.08E-190	########	0.686	0.383	1.02E-185	6
Plbd1	8.12E-234	########	0.758	0.39	1.36E-229	6
Pbx1	1.10E-297	########	0.661	0.265	1.84E-293	6
Svil	2.64E-227	########	0.766	0.483	4.42E-223	6
Lsp1	1.12E-206	########	0.925	0.655	1.87E-202	6
Hsd11b1	0	########	0.635	0.211	0	6
Cd44	2.46E-109	########	0.955	0.737	4.12E-105	6
Rasgrp4	0	########	0.622	0.17	0	6
Cdkn2d	4.92E-238	########	0.737	0.461	8.24E-234	6
Sema4a	2.51E-274	########	0.697	0.3	4.20E-270	6
Txnip	4.28E-104	########	0.823	0.592	7.16E-100	6
Mirt1	0	########	0.51	0.082	0	6
Gk	6.74E-226	########	0.452	0.149	1.13E-221	6
Hk3	0	########	0.639	0.189	0	6
Sp140	1.58E-272	########	0.704	0.35	2.65E-268	6
Pag1	0	########	0.674	0.243	0	6
Vsir	1.88E-243	########	0.843	0.566	3.15E-239	6
Rin3	0	########	0.707	0.294	0	6
Cyb5r4	1.34E-276	########	0.72	0.429	2.24E-272	6
Ccnd3	1.55E-232	########	0.798	0.6	2.59E-228	6
Unc119	1.04E-302	########	0.705	0.31	1.73E-298	6
Nfkbiz	1.48E-06	########	0.452	0.553	########	6
Cyfip2	0	########	0.638	0.223	0	6
Mtus1	2.90E-290	########	0.657	0.267	4.86E-286	6
Samd9l	1.67E-205	########	0.674	0.353	2.80E-201	6
Myo1f	1.06E-283	########	0.781	0.392	1.78E-279	6
Alas1	2.45E-201	########	0.644	0.352	4.11E-197	6
Pram1	0	########	0.582	0.07	0	6
Ceacam1	4.24E-293	########	0.56	0.191	7.09E-289	6
Stfa3	4.81E-44	########	0.107	0.032	8.05E-40	6
Skap2	1.90E-230	########	0.876	0.63	3.17E-226	6
Lrrk2	0	########	0.552	0.138	0	6
Pi16	0	########	0.642	0.165	0	6

Cmtm6	1.95E-200	########	0.713	0.423	3.26E-196	6
Hck	3.82E-291	########	0.756	0.365	6.39E-287	6
Bst1	4.75E-139	########	0.548	0.303	7.94E-135	6
Lta4h	6.06E-47	########	0.458	0.398	1.01E-42	6
Rcsd1	6.47E-280	########	0.739	0.366	1.08E-275	6
Pten	2.45E-147	########	0.84	0.688	4.09E-143	6
Snap23	4.02E-89	########	0.692	0.537	6.72E-85	6
Nadk	3.65E-204	########	0.776	0.512	6.11E-200	6
Atg3	5.02E-174	########	0.824	0.617	8.39E-170	6
Gm26740	2.55E-226	########	0.684	0.295	4.27E-222	6
Pla2g7	3.23E-44	########	0.547	0.442	5.41E-40	6
Nfe2	0	########	0.589	0.111	0	6
Myl12b	6.11E-273	########	0.924	0.855	1.02E-268	6
Plscr1	4.05E-230	########	0.566	0.245	6.77E-226	6
N4bp1	3.85E-131	########	0.743	0.492	6.44E-127	6
Ddx6	3.72E-51	########	0.846	0.77	6.23E-47	6
Steap4	9.98E-54	########	0.225	0.106	1.67E-49	6
Kdm7a	3.14E-146	########	0.795	0.569	5.26E-142	6
Ndel1	6.78E-62	########	0.681	0.536	1.13E-57	6
Ptafr	6.20E-76	########	0.596	0.362	1.04E-71	6
Cpne2	3.63E-263	########	0.639	0.286	6.07E-259	6
Csf2rb	1.37E-187	########	0.79	0.445	2.30E-183	6
Cd84	1.04E-96	########	0.663	0.437	1.75E-92	6
Pfkfb4	0	########	0.579	0.165	0	6
Capn1	2.78E-230	########	0.656	0.341	4.66E-226	6
Flna	4.18E-182	########	0.83	0.738	6.99E-178	6
Sun2	4.95E-266	########	0.661	0.309	8.28E-262	6
Sirpb1b	2.61E-114	########	0.325	0.131	4.37E-110	6
Hcst	9.84E-278	########	0.84	0.467	1.65E-273	6
Trpm2	0	########	0.384	0.052	0	6
Hcls1	3.62E-201	########	0.871	0.611	6.05E-197	6
7-Mar	1.72E-50	########	0.726	0.628	2.87E-46	6
Hdac4	3.46E-154	########	0.487	0.228	5.80E-150	6
Snx20	2.80E-105	########	0.711	0.434	4.69E-101	6
Smox	3.02E-42	########	0.355	0.245	5.05E-38	6
Pxn	2.67E-172	########	0.742	0.473	4.47E-168	6
B230208H1	0	########	0.479	0.039	0	6
AB124611	1.15E-167	########	0.803	0.505	1.92E-163	6
Hist1h1e	3.31E-53	########	0.541	0.363	5.53E-49	6
A530064D0	0	########	0.408	0.055	0	6
Coro1a	3.20E-202	########	0.973	0.768	5.36E-198	6
Sqrdl	1.82E-283	########	0.661	0.294	3.04E-279	6
Cd300lb	1.73E-223	########	0.492	0.169	2.90E-219	6
Emilin2	9.29E-57	########	0.664	0.535	1.55E-52	6
Fam129a	3.71E-181	########	0.697	0.391	6.21E-177	6
Fam107b	4.08E-148	########	0.737	0.489	6.83E-144	6
Hjurp	3.56E-224	########	0.592	0.274	5.95E-220	6
Slc22a15	0	########	0.528	0.127	0	6
Gm14548	2.92E-216	########	0.404	0.118	4.88E-212	6
Lmo4	1.54E-142	########	0.688	0.547	2.58E-138	6
Zcchc6	3.96E-96	########	0.741	0.573	6.62E-92	6
Cebpb	2.90E-20	########	0.925	0.755	4.84E-16	6

Rhog	2.77E-168	########	0.824	0.61	4.63E-164	6
Orm1	1.56E-293	########	0.277	0.025	2.62E-289	6
Arid3a	1.03E-292	########	0.607	0.225	1.72E-288	6
Agpat2	1.80E-177	########	0.487	0.228	3.02E-173	6
Diaph1	7.58E-120	########	0.876	0.71	1.27E-115	6
Tarm1	3.64E-266	########	0.445	0.114	6.09E-262	6
Oasl2	3.99E-47	########	0.286	0.164	6.67E-43	6
Qsox1	8.70E-207	########	0.529	0.222	1.46E-202	6
Sfxn5	4.41E-166	########	0.345	0.111	7.38E-162	6
Rnf11	7.35E-124	########	0.58	0.332	1.23E-119	6
Pbxip1	2.68E-138	########	0.673	0.435	4.48E-134	6
Fgd4	1.49E-245	########	0.629	0.253	2.49E-241	6
Xpc	9.55E-268	########	0.531	0.175	1.60E-263	6
Iqsec1	1.05E-177	########	0.556	0.26	1.76E-173	6
4932438A1	1.76E-91	########	0.642	0.45	2.94E-87	6
Atp11b	3.15E-152	########	0.745	0.51	5.27E-148	6
Ankrd22	0	########	0.432	0.012	0	6
D1Ertd622e	2.31E-117	########	0.636	0.391	3.86E-113	6
Arpc5	6.34E-208	########	0.939	0.905	1.06E-203	6
Capza1	1.75E-180	########	0.86	0.732	2.93E-176	6
4833407H1	8.17E-219	########	0.28	0.046	1.37E-214	6
Lasp1	3.93E-152	########	0.729	0.516	6.57E-148	6
Mefv	1.72E-18	########	0.347	0.267	2.87E-14	6
Klhdc4	4.01E-136	########	0.467	0.235	6.71E-132	6
Mrpl33	8.49E-144	########	0.933	0.848	1.42E-139	6
Sepn1	2.22E-57	########	0.365	0.229	3.71E-53	6
Fam32a	2.05E-121	########	0.749	0.549	3.42E-117	6
Lilra6	7.27E-165	########	0.334	0.099	1.22E-160	6
Tlr13	3.11E-60	########	0.422	0.266	5.21E-56	6
Clec12a	6.18E-143	########	0.633	0.356	1.03E-138	6
Ypel3	1.04E-87	########	0.729	0.56	1.73E-83	6
Gca	0	########	0.423	0.052	0	6
Il17ra	5.20E-122	########	0.689	0.454	8.69E-118	6
Rab27a	0	########	0.58	0.17	0	6
Ube2b	5.41E-107	########	0.885	0.782	9.05E-103	6
Fes	1.02E-182	########	0.726	0.429	1.71E-178	6
Pira2	2.09E-134	########	0.528	0.26	3.50E-130	6
Crispld2	0	########	0.489	0.111	0	6
Fmnl1	1.66E-144	########	0.743	0.435	2.79E-140	6
Syk	7.15E-114	########	0.792	0.513	1.20E-109	6
Cep19	0	########	0.499	0.108	0	6
Gm17619	0	########	0.267	0.01	0	6
Dfna5	4.09E-305	########	0.541	0.169	6.85E-301	6
Picalm	7.35E-23	########	0.839	0.786	1.23E-18	6
Marcksl1	2.28E-11	########	0.552	0.464	3.81E-07	6
Cmip	2.64E-94	########	0.863	0.689	4.41E-90	6
Snx18	2.11E-105	########	0.698	0.473	3.53E-101	6
1600010M(	4.42E-153	########	0.382	0.138	7.40E-149	6
Fam134b	1.69E-86	########	0.498	0.284	2.83E-82	6
Srpk2	8.15E-143	########	0.705	0.479	1.36E-138	6
Hipk1	4.30E-136	########	0.753	0.591	7.20E-132	6
Hist1h4d	7.36E-08	########	0.365	0.312	########	6

Tnfaip3	8.60E-25	########	0.232	0.413	1.44E-20	6
Scrg1	0	########	0.394	0.01	0	6
Mctp1	1.37E-282	########	0.542	0.17	2.29E-278	6
Tsc22d4	4.07E-145	########	0.822	0.739	6.80E-141	6
Itgal	2.20E-220	########	0.682	0.287	3.68E-216	6
Max	1.08E-141	########	0.674	0.432	1.80E-137	6
Ppp2r5a	2.00E-143	########	0.79	0.646	3.34E-139	6
Fth1	3.84E-22	########	0.998	0.995	6.43E-18	6
Ly75	0	########	0.416	0.052	0	6
Rlf	3.03E-120	########	0.536	0.298	5.06E-116	6
Rab3d	5.21E-239	########	0.534	0.208	8.71E-235	6
Nlrp12	0	########	0.286	0.007	0	6
Treml2	1.79E-272	########	0.553	0.17	2.99E-268	6
Ptpre	1.48E-150	########	0.694	0.425	2.48E-146	6
Rgs18	1.76E-229	########	0.555	0.212	2.94E-225	6
Abca13	0	########	0.396	0.029	0	6
Chd7	5.56E-73	########	0.58	0.369	9.30E-69	6
Trp53inp1	7.61E-175	########	0.456	0.166	1.27E-170	6
Ipcef1	0	########	0.541	0.112	0	6
Fgl2	2.37E-08	########	0.369	0.349	########	6
Carhsp1	2.87E-120	########	0.609	0.363	4.81E-116	6
Abhd5	7.17E-115	########	0.493	0.3	1.20E-110	6
Hist1h2bc	2.41E-38	########	0.466	0.341	4.04E-34	6
Pstpip1	6.08E-149	########	0.713	0.424	1.02E-144	6
Usp32	4.84E-99	########	0.425	0.224	8.10E-95	6
Oas3	4.94E-159	########	0.355	0.112	8.27E-155	6
Tmsb4x	2.62E-241	########	0.998	0.997	4.38E-237	6
Sephs2	3.52E-99	########	0.51	0.293	5.90E-95	6
Hist1h3c	2.26E-18	########	0.149	0.089	3.78E-14	6
Rassf5	4.86E-136	########	0.641	0.353	8.14E-132	6
Il16	1.69E-204	########	0.624	0.272	2.83E-200	6
Slc35a5	6.43E-185	########	0.479	0.185	1.08E-180	6
Gnai2	2.81E-194	########	0.967	0.944	4.70E-190	6
Baz2b	3.36E-80	########	0.7	0.544	5.63E-76	6
Fmo5	0	########	0.45	0.066	0	6
Fas	1.54E-32	########	0.329	0.234	2.57E-28	6
Rab8b	3.04E-50	########	0.798	0.668	5.09E-46	6
1700047M1	0	########	0.361	0.007	0	6
Megf9	1.34E-286	########	0.478	0.132	2.24E-282	6
Themis2	4.08E-80	########	0.384	0.198	6.83E-76	6
9830107B1	0	########	0.389	0.008	0	6
Dgat2	1.54E-294	########	0.462	0.112	2.58E-290	6
Gm5150	1.10E-28	########	0.297	0.208	1.84E-24	6
Sh2d3c	4.37E-180	########	0.432	0.145	7.31E-176	6
Nhsl2	2.96E-194	########	0.488	0.189	4.95E-190	6
Klhl2	2.72E-135	########	0.409	0.177	4.56E-131	6
Flot1	3.24E-109	########	0.694	0.497	5.41E-105	6
Glipr1	1.09E-95	########	0.572	0.336	1.82E-91	6
F5	2.44E-274	########	0.422	0.092	4.09E-270	6
Apbb1ip	6.63E-88	########	0.849	0.669	1.11E-83	6
Hiatl1	1.09E-126	########	0.613	0.37	1.83E-122	6
Neat1	1.81E-14	########	0.94	0.846	3.03E-10	6

Chp1	2.43E-102	########	0.621	0.41	4.07E-98	6
Osbpl9	1.19E-65	########	0.725	0.573	1.99E-61	6
Atp6v1g1	3.31E-84	########	0.927	0.844	5.53E-80	6
Asb7	1.71E-239	########	0.412	0.11	2.87E-235	6
Triobp	1.25E-192	########	0.55	0.256	2.09E-188	6
Ppm1m	2.87E-143	########	0.527	0.3	4.80E-139	6
Cd53	4.71E-47	########	0.937	0.725	7.88E-43	6
Msl1	1.86E-96	########	0.644	0.458	3.11E-92	6
Ccdc88b	3.17E-228	########	0.497	0.16	5.30E-224	6
Pkm	9.60E-142	########	0.977	0.859	1.61E-137	6
Adam19	2.13E-79	########	0.293	0.134	3.56E-75	6
Slc27a4	5.97E-238	########	0.429	0.124	9.98E-234	6
Rassf3	1.88E-106	########	0.61	0.362	3.15E-102	6
Tirap	3.84E-219	########	0.429	0.133	6.43E-215	6
Cfap43	4.06E-150	########	0.451	0.186	6.79E-146	6
Atp6v1e1	6.03E-86	########	0.852	0.759	1.01E-81	6
Actr3	1.28E-158	########	0.951	0.924	2.14E-154	6
Fxyd5	1.32E-24	########	0.944	0.873	2.21E-20	6
Ccdc125	2.52E-289	########	0.477	0.12	4.22E-285	6
Dok3	4.79E-167	########	0.635	0.322	8.02E-163	6
Aldoa	1.61E-124	########	0.913	0.856	2.69E-120	6
Slc31a2	2.40E-157	########	0.537	0.269	4.02E-153	6
Phf20l1	1.62E-102	########	0.654	0.475	2.71E-98	6
Slc15a3	7.21E-28	########	0.478	0.385	1.21E-23	6
Tmem71	1.09E-143	########	0.502	0.22	1.83E-139	6
Arpc3	2.04E-130	########	0.975	0.95	3.41E-126	6
Fam111a	5.94E-102	########	0.699	0.481	9.94E-98	6
Xdh	2.50E-125	########	0.643	0.389	4.18E-121	6
Tspo	9.41E-116	0.486605	0.955	0.92	1.58E-111	6
Il13ra1	3.85E-07	########	0.373	0.388	########	6
Anxa3	4.31E-58	########	0.546	0.434	7.21E-54	6
Aldh3b1	7.56E-141	########	0.613	0.358	1.27E-136	6
Arhgap25	1.43E-159	########	0.528	0.227	2.40E-155	6
Adgrg3	0	########	0.422	0.057	0	6
Klf7	2.90E-63	########	0.655	0.477	4.85E-59	6
Rab6a	2.31E-100	########	0.686	0.501	3.86E-96	6
Trim12c	2.72E-101	########	0.55	0.306	4.56E-97	6
Mgam	0	########	0.356	0.014	0	6
Acsl1	1.29E-153	########	0.464	0.208	2.16E-149	6
Ebi3	4.76E-296	########	0.479	0.12	7.97E-292	6
Atp8b4	3.81E-136	########	0.592	0.297	6.38E-132	6
Xylt1	2.09E-69	########	0.395	0.223	3.49E-65	6
Tnrc6b	9.34E-100	########	0.691	0.484	1.56E-95	6
Card19	1.13E-35	########	0.797	0.65	1.90E-31	6
Dennd4a	2.36E-27	########	0.517	0.408	3.96E-23	6
Gnai3	9.01E-94	########	0.767	0.61	1.51E-89	6
Tmem40	0	########	0.44	0.044	0	6
Csf2ra	1.44E-122	########	0.746	0.459	2.41E-118	6
Rhov	4.42E-53	########	0.209	0.091	7.39E-49	6
Vamp5	1.32E-216	########	0.52	0.185	2.20E-212	6
AI467606	1.02E-178	########	0.568	0.232	1.71E-174	6
Kctd12	4.94E-47	########	0.74	0.569	8.27E-43	6

Cmah	3.19E-193	########	0.445	0.135	5.34E-189	6
Bcl2l11	3.55E-12	########	0.31	0.446	5.95E-08	6
Arhgap9	2.30E-126	########	0.687	0.41	3.85E-122	6
Cln3	1.11E-91	########	0.472	0.263	1.86E-87	6
Ehd1	7.80E-58	########	0.796	0.575	1.30E-53	6
Gsap	1.04E-89	########	0.429	0.218	1.74E-85	6
Mov10	3.25E-117	########	0.268	0.087	5.44E-113	6
Rab7	1.53E-26	########	0.875	0.781	2.57E-22	6
Tnfrsf23	2.00E-12	########	0.169	0.122	3.34E-08	6
Ppp1r18	3.16E-97	########	0.793	0.649	5.30E-93	6
Golim4	4.93E-74	########	0.443	0.283	8.24E-70	6
Fcgr3	1.60E-113	0.456402	0.874	0.513	2.67E-109	6
Casp4	2.94E-18	########	0.509	0.427	4.91E-14	6
1110008F1	2.02E-111	########	0.857	0.756	3.39E-107	6
Fgd3	2.81E-188	########	0.407	0.122	4.70E-184	6
Raf1	3.57E-69	########	0.58	0.411	5.97E-65	6
4930438A0	0	########	0.366	0.017	0	6
Shfm1	1.62E-159	########	0.976	0.948	2.72E-155	6
Snx10	4.54E-33	########	0.533	0.416	7.60E-29	6
Eif4ebp1	2.36E-44	########	0.754	0.597	3.95E-40	6
Mbd2	8.44E-87	########	0.789	0.67	1.41E-82	6
Gmip	1.18E-135	########	0.578	0.307	1.98E-131	6
Rinl	1.22E-200	########	0.568	0.23	2.05E-196	6
Ppp1r3d	6.03E-305	########	0.277	0.022	1.01E-300	6
Nbeal2	2.70E-232	########	0.418	0.11	4.52E-228	6
Ampd3	1.08E-151	########	0.457	0.191	1.80E-147	6
Cotl1	1.32E-105	########	0.938	0.803	2.21E-101	6
Myadm	3.20E-12	########	0.463	0.435	5.35E-08	6
Tacstd2	7.76E-278	########	0.4	0.077	1.30E-273	6
Acvrl1	1.23E-38	########	0.321	0.217	2.05E-34	6
Arap3	1.83E-305	########	0.401	0.071	3.05E-301	6
Slc2a6	1.18E-78	########	0.24	0.095	1.98E-74	6
Tuba4a	7.60E-46	########	0.578	0.449	1.27E-41	6
Ppp1r2	3.22E-108	########	0.776	0.596	5.39E-104	6
Notch2	1.97E-32	########	0.515	0.418	3.30E-28	6
Nin	1.16E-81	########	0.59	0.421	1.95E-77	6
Riok3	6.39E-83	########	0.768	0.616	1.07E-78	6
Neurl3	1.32E-38	########	0.532	0.384	2.21E-34	6
Hacd4	8.65E-63	########	0.628	0.452	1.45E-58	6
Samhd1	7.33E-43	########	0.818	0.756	1.23E-38	6
Gadd45b	2.47E-26	########	0.496	0.619	4.14E-22	6
Ppp1r3b	1.92E-216	########	0.269	0.042	3.20E-212	6
Ikbkap	4.95E-110	########	0.403	0.193	8.28E-106	6
Actg1	7.95E-75	########	0.983	0.976	1.33E-70	6
Lims1	1.26E-50	########	0.644	0.567	2.11E-46	6
Ssh2	1.88E-39	########	0.711	0.576	3.15E-35	6
Mapk3	1.03E-102	########	0.678	0.511	1.72E-98	6
Suco	2.62E-16	########	0.438	0.367	4.39E-12	6
Rnd1	5.99E-147	########	0.299	0.085	1.00E-142	6
Rnasel	9.15E-78	########	0.523	0.309	1.53E-73	6
Pdzd8	7.62E-92	########	0.571	0.377	1.28E-87	6
Rab44	0	########	0.419	0.062	0	6

Pet100	3.77E-75	########	0.768	0.67	6.31E-71	6
Pnrc1	7.44E-07	########	0.812	0.837	########	6
Nedd9	2.09E-72	########	0.483	0.29	3.49E-68	6
Numb	5.89E-134	########	0.443	0.199	9.86E-130	6
Fam122a	5.67E-119	########	0.46	0.227	9.49E-115	6
Osgin1	1.85E-125	########	0.224	0.055	3.09E-121	6
Pde2a	1.76E-214	########	0.506	0.174	2.94E-210	6
Kctd20	3.95E-110	########	0.582	0.36	6.62E-106	6
Mboat7	3.11E-91	########	0.483	0.278	5.20E-87	6
Npepps	1.64E-71	########	0.576	0.428	2.74E-67	6
Dennd5a	2.95E-53	########	0.594	0.447	4.94E-49	6
Xpo6	1.27E-36	########	0.454	0.346	2.12E-32	6
Cdc42ep3	5.90E-117	########	0.617	0.353	9.88E-113	6
Irak3	5.37E-60	########	0.449	0.275	8.98E-56	6
Dock8	3.47E-93	########	0.677	0.457	5.80E-89	6
Rtp4	1.44E-13	########	0.257	0.202	2.41E-09	6
Map4k2	1.03E-211	########	0.486	0.16	1.73E-207	6
Bcl10	3.62E-39	########	0.783	0.682	6.06E-35	6
Ube2h	6.50E-32	########	0.596	0.505	1.09E-27	6
Pak2	7.83E-87	########	0.847	0.809	1.31E-82	6
Ncor1	2.31E-53	########	0.803	0.76	3.86E-49	6
St3gal6	2.31E-36	########	0.204	0.109	3.87E-32	6
Slco4c1	0	########	0.353	0.01	0	6
Adrbk1	1.06E-69	########	0.765	0.635	1.77E-65	6
Gpr84	3.24E-147	########	0.293	0.076	5.42E-143	6
Atp2c1	8.64E-104	########	0.599	0.384	1.45E-99	6
Mafg	1.44E-78	########	0.641	0.486	2.41E-74	6
Isg20	3.04E-50	########	0.284	0.153	5.08E-46	6
Pdlim7	8.30E-98	########	0.472	0.24	1.39E-93	6
Gng12	9.32E-53	########	0.708	0.534	1.56E-48	6
Sla	2.03E-108	########	0.654	0.372	3.39E-104	6
Stxbp2	9.38E-119	########	0.597	0.34	1.57E-114	6
Bin3	2.19E-103	########	0.571	0.352	3.66E-99	6
Orai1	7.45E-84	########	0.681	0.481	1.25E-79	6
Mmp25	0	########	0.328	0.01	0	6
I830077J02	5.02E-156	########	0.453	0.172	8.39E-152	6
Basp1	4.37E-08	########	0.273	0.37	########	6
Stk38	4.24E-71	########	0.709	0.559	7.10E-67	6
Pilrb1	3.72E-219	########	0.413	0.108	6.23E-215	6
Itgam.1	1.70E-193	########	0.268	0.048	2.84E-189	6
Dedd2	7.27E-68	########	0.384	0.209	1.22E-63	6
Btg2	2.52E-17	########	0.773	0.713	4.21E-13	6
E2f2	9.19E-209	########	0.441	0.134	1.54E-204	6
Vps4b	4.67E-25	########	0.671	0.596	7.81E-21	6
Atrn	5.38E-127	########	0.427	0.193	9.01E-123	6
Por	3.21E-31	########	0.613	0.504	5.37E-27	6
Zdhhc18	3.08E-114	########	0.483	0.238	5.16E-110	6
Rb1cc1	6.72E-31	########	0.674	0.577	1.12E-26	6
Cers6	3.07E-44	########	0.54	0.392	5.13E-40	6
Flot2	3.88E-93	########	0.529	0.337	6.50E-89	6
Dock5	2.39E-177	########	0.44	0.154	4.01E-173	6
Klra2	2.01E-65	########	0.307	0.149	3.37E-61	6

Rab32	6.80E-34	########	0.505	0.372	1.14E-29	6
Braf	3.60E-38	########	0.466	0.34	6.03E-34	6
Tmem38b	2.19E-80	########	0.496	0.285	3.67E-76	6
Tmbim6	3.87E-83	########	0.92	0.872	6.47E-79	6
Clip1	2.10E-56	########	0.569	0.417	3.51E-52	6
Pbx2	2.10E-108	########	0.504	0.275	3.51E-104	6
Cds2	3.81E-90	########	0.469	0.26	6.37E-86	6
Tcp11l2	1.83E-68	########	0.574	0.401	3.06E-64	6
Arhgap30	4.50E-72	########	0.762	0.558	7.52E-68	6
Trim30a	9.60E-25	########	0.57	0.458	1.61E-20	6
Ddx60	7.72E-66	########	0.245	0.103	1.29E-61	6
Ggt5	2.93E-94	########	0.3	0.119	4.90E-90	6
Ogt	1.22E-29	########	0.625	0.521	2.04E-25	6
Cd82	2.65E-95	########	0.517	0.281	4.44E-91	6
Plxnc1	3.21E-175	########	0.395	0.123	5.37E-171	6
Anxa2	5.69E-72	########	0.942	0.782	9.52E-68	6
Ap3s1	1.94E-64	########	0.646	0.543	3.25E-60	6
Degs1	1.89E-46	########	0.662	0.582	3.16E-42	6
Adgre5	1.62E-20	########	0.692	0.566	2.71E-16	6
Lrrfip1	5.89E-67	########	0.818	0.682	9.86E-63	6
Hist1h2ab	2.95E-17	########	0.134	0.078	4.94E-13	6
Rhou	7.80E-153	########	0.384	0.144	1.30E-148	6
Acpp	0	########	0.338	0.041	0	6
Cdc42se1	1.57E-38	########	0.731	0.644	2.63E-34	6
Dck	1.21E-42	########	0.43	0.296	2.03E-38	6
Gcnt1	1.22E-38	########	0.25	0.143	2.04E-34	6
Gm20406	1.19E-247	########	0.185	0.005	1.98E-243	6
Hectd1	3.79E-18	########	0.693	0.624	6.35E-14	6
Hist1h1c	1.43E-23	########	0.505	0.412	2.40E-19	6
Jdp2	1.12E-61	########	0.53	0.336	1.87E-57	6
Mctp2	1.20E-184	########	0.295	0.062	2.00E-180	6
Arfgef1	5.31E-49	########	0.579	0.475	8.88E-45	6
Tecr	6.07E-34	########	0.602	0.6	1.01E-29	6
Ero1l	1.32E-30	########	0.427	0.343	2.21E-26	6
Dgkg	8.63E-189	########	0.331	0.081	1.44E-184	6
Dach1	0	########	0.358	0.039	0	6
Prkcb	1.28E-108	########	0.568	0.302	2.14E-104	6
Slc9a3r1	6.09E-53	########	0.605	0.434	1.02E-48	6
Atp11a	1.21E-54	########	0.377	0.23	2.03E-50	6
Mark2	1.11E-64	########	0.64	0.481	1.85E-60	6
Klra17	2.19E-264	########	0.222	0.012	3.67E-260	6
Slc25a24	3.66E-89	########	0.487	0.292	6.13E-85	6
Cib2	1.26E-204	########	0.415	0.123	2.10E-200	6
Asnsd1	3.58E-71	########	0.557	0.387	5.98E-67	6
Bcl6	5.08E-80	########	0.367	0.184	8.51E-76	6
Rab24	3.94E-63	########	0.637	0.502	6.59E-59	6
Akna	6.31E-105	########	0.539	0.273	1.06E-100	6
Cd101	2.05E-106	########	0.179	0.04	3.44E-102	6
Atg7	4.86E-69	########	0.45	0.267	8.13E-65	6
Sesn2	3.59E-46	########	0.244	0.13	6.00E-42	6
Cass4	2.96E-20	########	0.125	0.068	4.95E-16	6
Ezr	5.32E-45	########	0.71	0.553	8.90E-41	6

Tst	1.95E-225	########	0.361	0.087	3.27E-221	6
Ralgapa1	5.16E-82	########	0.423	0.228	8.64E-78	6
Lipg	1.77E-262	########	0.234	0.017	2.97E-258	6
Spata13	2.15E-20	########	0.28	0.193	3.60E-16	6
Scp2	1.16E-15	########	0.726	0.844	1.94E-11	6
Mrgpra2a	0	########	0.253	0.009	0	6
Atxn7l1	1.02E-59	########	0.462	0.284	1.70E-55	6
Arpc1b	1.67E-97	########	0.974	0.955	2.80E-93	6
Sat1	5.75E-41	########	0.933	0.839	9.62E-37	6
Twf2	3.13E-61	########	0.672	0.555	5.23E-57	6
Pfn1	3.87E-130	########	0.987	0.981	6.48E-126	6
Lars2	3.88E-09	########	0.984	0.975	6.50E-05	6
Sos2	1.17E-82	########	0.426	0.23	1.95E-78	6
Rap2c	5.66E-42	########	0.608	0.474	9.46E-38	6
Bnip3l	6.53E-62	########	0.78	0.696	1.09E-57	6
Rgs19	3.27E-65	########	0.592	0.44	5.48E-61	6
Fcgr4	6.96E-99	########	0.522	0.261	1.17E-94	6
Gdpd3	1.93E-89	########	0.347	0.155	3.23E-85	6
Chst11	1.35E-48	########	0.235	0.116	2.26E-44	6
Gripap1	6.63E-44	########	0.526	0.384	1.11E-39	6
Nabp1	1.65E-09	########	0.431	0.383	2.77E-05	6
Agpat9	3.79E-108	########	0.225	0.061	6.34E-104	6
Hiat1	3.97E-54	########	0.497	0.345	6.65E-50	6
Myd88	1.96E-22	########	0.533	0.45	3.28E-18	6
Asxl1	3.69E-64	########	0.463	0.292	6.17E-60	6
Sort1	4.60E-139	########	0.438	0.179	7.70E-135	6
Preb	1.42E-16	########	0.415	0.348	2.37E-12	6
Ethe1	8.94E-40	########	0.532	0.451	1.50E-35	6
Tnfrsf1a	1.00E-21	########	0.736	0.616	1.68E-17	6
Mtmr3	1.17E-74	########	0.54	0.348	1.96E-70	6
H3f3a	5.80E-105	########	0.996	0.986	9.70E-101	6
Rgs3	1.16E-41	########	0.316	0.194	1.93E-37	6
Tnfsf14	5.75E-83	########	0.232	0.081	9.63E-79	6
Fcer1g	2.69E-39	########	0.985	0.659	4.49E-35	6
Mtmr6	1.30E-35	########	0.492	0.368	2.18E-31	6
Hmha1	9.73E-86	########	0.763	0.504	1.63E-81	6
Zdhhc3	1.60E-59	########	0.531	0.369	2.67E-55	6
Ptpn6	1.46E-67	########	0.766	0.556	2.44E-63	6
Rgs2	7.52E-50	########	0.704	0.491	1.26E-45	6
Prkcd	5.90E-59	########	0.722	0.532	9.87E-55	6
Ggt1	5.59E-174	########	0.166	0.014	9.35E-170	6
Scnn1a	1.64E-135	########	0.23	0.052	2.75E-131	6
Syne2	3.03E-89	########	0.395	0.197	5.07E-85	6
Tbc1d14	8.43E-17	########	0.358	0.291	1.41E-12	6
Ppp1r12a	1.20E-53	########	0.675	0.58	2.01E-49	6
Fam63a	4.19E-44	########	0.487	0.341	7.01E-40	6
Ccdc180	7.74E-137	########	0.238	0.056	1.29E-132	6
Fry	4.74E-103	########	0.44	0.205	7.93E-99	6
Gapdh	6.69E-81	########	0.961	0.939	1.12E-76	6
Abr	8.55E-25	########	0.448	0.337	1.43E-20	6
Hsd17b11	2.36E-44	########	0.554	0.401	3.94E-40	6
Tln1	8.18E-65	########	0.871	0.866	1.37E-60	6

Clec4a2	2.93E-80	########	0.716	0.462	4.91E-76	6
Gpr27	2.70E-196	########	0.21	0.025	4.52E-192	6
Fam21	1.44E-29	########	0.619	0.498	2.40E-25	6
Tbc1d8	1.91E-183	########	0.38	0.11	3.20E-179	6
Inpp1	4.41E-70	########	0.35	0.176	7.38E-66	6
Snrk	4.24E-72	########	0.458	0.269	7.10E-68	6
Tlr4	1.99E-23	########	0.258	0.17	3.32E-19	6
Rps6ka1	1.11E-73	########	0.54	0.345	1.86E-69	6
Nt5c2	7.08E-50	########	0.375	0.228	1.18E-45	6
Trim12a	2.35E-74	########	0.556	0.329	3.92E-70	6
Gm19705	4.98E-233	########	0.289	0.045	8.34E-229	6
Nusap1	6.48E-10	########	0.144	0.101	1.08E-05	6
Mical1	1.68E-76	########	0.378	0.196	2.82E-72	6
Bcl2a1b	4.70E-10	########	0.159	0.238	7.87E-06	6
Gyg	2.80E-82	########	0.593	0.376	4.69E-78	6
Akap13	5.58E-20	########	0.844	0.81	9.34E-16	6
Larp4b	3.51E-14	########	0.59	0.548	5.88E-10	6
Hlx	3.01E-112	########	0.328	0.123	5.04E-108	6
Stap1	6.87E-139	########	0.568	0.261	1.15E-134	6
Lcp2	1.71E-38	########	0.605	0.424	2.86E-34	6
Ankrd13a	5.55E-43	########	0.591	0.457	9.29E-39	6
Tcn2	1.35E-57	########	0.62	0.427	2.26E-53	6
1810058I24	4.93E-27	########	0.776	0.724	8.26E-23	6
Pip5k1b	1.23E-278	########	0.286	0.031	2.06E-274	6
Ypel5	1.02E-40	########	0.551	0.409	1.70E-36	6
Nod1	1.04E-69	########	0.37	0.191	1.74E-65	6
E030030I0	3.02E-54	########	0.22	0.098	5.06E-50	6
Rnf167	6.52E-59	########	0.54	0.356	1.09E-54	6
RP23-6I17.	1.07E-08	########	0.134	0.095	########	6
Oaz2	1.06E-31	########	0.398	0.288	1.77E-27	6
Arl11	7.26E-54	########	0.45	0.262	1.21E-49	6
Chst12	9.19E-84	########	0.458	0.257	1.54E-79	6
Acap1	4.27E-190	########	0.448	0.134	7.14E-186	6
Aqp9	5.92E-133	########	0.166	0.024	9.91E-129	6
Vcl	2.53E-65	########	0.498	0.331	4.24E-61	6
Cyba	1.48E-58	########	0.967	0.924	2.48E-54	6
Inpp4a	3.17E-108	########	0.422	0.192	5.30E-104	6
Mapk14	3.58E-32	########	0.563	0.447	5.99E-28	6
Arhgap4	3.72E-107	########	0.489	0.236	6.22E-103	6
Map3k3	1.97E-38	########	0.397	0.268	3.30E-34	6
Fam160a2	2.31E-132	########	0.357	0.13	3.87E-128	6
Azin1	3.45E-32	########	0.657	0.542	5.77E-28	6
Dennd3	3.63E-165	########	0.203	0.029	6.07E-161	6
Sema4d	5.47E-14	########	0.428	0.335	9.15E-10	6
Dennd1c	6.48E-109	########	0.366	0.144	1.08E-104	6
Prr5l	4.53E-33	########	0.135	0.06	7.57E-29	6
Arl2bp	1.15E-38	########	0.433	0.307	1.92E-34	6
Ptpn1	1.52E-23	########	0.837	0.748	2.54E-19	6
Lyn	2.87E-50	########	0.887	0.639	4.80E-46	6
Tgm2	7.28E-15	########	0.198	0.321	1.22E-10	6
Tmem216	2.58E-111	########	0.273	0.093	4.32E-107	6
4732465J0	9.70E-146	########	0.126	0.008	1.62E-141	6

Kdm5b	8.76E-36	########	0.412	0.287	1.47E-31	6
Lrrfip2	3.15E-35	########	0.57	0.46	5.28E-31	6
Arpc2	1.02E-73	########	0.973	0.959	1.71E-69	6
Mid1ip1	2.95E-66	########	0.43	0.256	4.93E-62	6
Borcs6	2.22E-31	########	0.438	0.319	3.72E-27	6
Zfp608	1.05E-49	########	0.359	0.205	1.76E-45	6
Ltb	1.59E-231	########	0.676	0.22	2.66E-227	6
Palm	4.28E-98	########	0.368	0.168	7.16E-94	6
Amn1	9.37E-40	########	0.315	0.188	1.57E-35	6
Dopey2	3.09E-53	########	0.27	0.136	5.16E-49	6
Cdc42ep2	6.67E-23	########	0.188	0.114	1.12E-18	6
Kcnj2	1.03E-91	########	0.194	0.054	1.73E-87	6
Cdkn1b	6.31E-29	########	0.697	0.591	1.06E-24	6
Tax1bp1	1.79E-16	########	0.878	0.842	2.99E-12	6
S1pr4	1.81E-157	########	0.446	0.155	3.03E-153	6
Cldn15	1.89E-217	########	0.324	0.064	3.16E-213	6
Fam217b	6.15E-135	########	0.281	0.081	1.03E-130	6
Stim2	6.09E-34	########	0.425	0.299	1.02E-29	6
Olr1	3.03E-52	########	0.17	0.062	5.07E-48	6
Kif21b	1.61E-126	########	0.441	0.177	2.70E-122	6
Abcd2	1.49E-115	########	0.392	0.16	2.49E-111	6
Parvg	8.74E-125	########	0.424	0.169	1.46E-120	6
Hbp1	3.65E-13	########	0.543	0.489	6.11E-09	6
Cbl	2.50E-29	########	0.697	0.606	4.19E-25	6
Setd8	9.96E-23	########	0.554	0.487	1.67E-18	6
4933408B1	1.36E-217	########	0.224	0.024	2.27E-213	6
Tle3	3.57E-17	########	0.462	0.382	5.97E-13	6
Pnpla7	3.16E-56	########	0.378	0.214	5.28E-52	6
Rfwd2	2.28E-32	########	0.582	0.467	3.82E-28	6
Gpr141	1.75E-115	########	0.469	0.192	2.92E-111	6
Svip	1.19E-123	########	0.35	0.124	1.99E-119	6
Was	1.40E-43	########	0.63	0.464	2.34E-39	6
Gclm	7.77E-34	########	0.504	0.377	1.30E-29	6
P2ry13	9.31E-82	########	0.145	0.033	1.56E-77	6
Map3k5	8.80E-35	########	0.304	0.187	1.47E-30	6
Tpm3	2.32E-43	########	0.922	0.927	3.89E-39	6
Slc25a37	5.92E-32	########	0.361	0.243	9.90E-28	6
Tnfaip8	3.17E-37	########	0.734	0.64	5.30E-33	6
Smap2	7.30E-34	########	0.684	0.581	1.22E-29	6
Clec2d	1.59E-14	########	0.614	0.516	2.67E-10	6
1700020L2	2.58E-148	########	0.254	0.057	4.32E-144	6
Slc28a2	8.58E-84	########	0.378	0.17	1.44E-79	6
Stk26	3.15E-87	########	0.385	0.175	5.28E-83	6
Wbp1l	1.99E-08	########	0.376	0.338	########	6
Ppp1r42	0	########	0.265	0.006	0	6
Ankrd44	6.13E-38	########	0.619	0.452	1.03E-33	6
Hist1h2be	1.27E-52	########	0.231	0.106	2.13E-48	6
Optn	1.14E-158	########	0.247	0.052	1.90E-154	6
Plcl2	4.54E-70	########	0.43	0.234	7.59E-66	6
Nt5c3	4.24E-64	########	0.455	0.284	7.09E-60	6
Agap1	2.92E-86	########	0.355	0.166	4.89E-82	6
Armc7	4.75E-71	########	0.233	0.09	7.95E-67	6

Tnfsf13b	2.50E-201	########	0.251	0.038	4.18E-197	6
Ffar2	1.07E-206	########	0.255	0.037	1.80E-202	6
Atp1a3	3.99E-46	########	0.331	0.184	6.68E-42	6
Map2k4	1.84E-29	########	0.438	0.321	3.09E-25	6
Hacd2	7.34E-44	########	0.47	0.336	1.23E-39	6
Lnpep	1.00E-12	########	0.442	0.379	1.67E-08	6
Cox17	1.69E-24	########	0.729	0.664	2.83E-20	6
Cd74	0	4.18614	0.598	0.098	0	7
Pf4	0	4.127571	0.508	0.138	0	7
Apoe	0	3.969101	0.807	0.332	0	7
C1qb	0	3.655825	0.463	0.073	0	7
Ctss	0	3.434377	0.965	0.235	0	7
C1qa	0	3.342201	0.424	0.056	0	7
H2-Ab1	0	3.329888	0.395	0.075	0	7
H2-Aa	0	3.290191	0.361	0.051	0	7
H2-Eb1	1.25E-274	3.206644	0.329	0.05	2.10E-270	7
Lgmn	0	3.115538	0.807	0.254	0	7
Ms4a6c	0	2.958266	0.845	0.093	0	7
Sepp1	0	2.919884	0.767	0.418	0	7
Ctsb	0	2.848167	0.897	0.69	0	7
C1qc	0	2.771312	0.428	0.042	0	7
Mrc1	0	2.733997	0.475	0.036	0	7
Ifi30	0	2.702932	0.906	0.282	0	7
Psap	0	2.618434	0.937	0.787	0	7
Ctsc	0	2.609363	0.894	0.326	0	7
Ccl9	0	2.484445	0.808	0.134	0	7
F13a1	0	2.448894	0.695	0.066	0	7
Mafb	0	2.364196	0.693	0.161	0	7
Ccr2	0	2.323367	0.668	0.085	0	7
Aif1	0	2.314534	0.67	0.042	0	7
Ccl8	1.08E-42	2.14337	0.19	0.099	1.82E-38	7
Dab2	0	2.135843	0.544	0.157	0	7
Ms4a7	0	2.082515	0.487	0.02	0	7
AI607873	0	2.078486	0.793	0.11	0	7
Ms4a6d	0	2.0709	0.762	0.059	0	7
Grn	0	2.028674	0.848	0.572	0	7
Hmox1	3.74E-294	2.00575	0.638	0.336	6.26E-290	7
Arg1	1.26E-199	1.936012	0.219	0.019	2.10E-195	7
Trem2	0	1.912335	0.673	0.036	0	7
Cd79a	5.38E-81	1.893476	0.118	0.02	8.99E-77	7
Csf1r	0	1.886025	0.808	0.127	0	7
Ctsl	0	1.87741	0.781	0.425	0	7
S100a4	0	1.865975	0.836	0.294	0	7
Cd68	0	1.865813	0.817	0.206	0	7
Fcgr2b	0	1.849217	0.77	0.186	0	7
Lamp1	0	1.82574	0.9	0.679	0	7
Lyz2	0	1.747549	0.892	0.641	0	7
Tgfbi	0	1.695165	0.83	0.503	0	7
Npc2	0	1.636387	0.914	0.76	0	7
Ftl1	0	1.632284	0.992	0.99	0	7
Clec4a3	0	1.624901	0.705	0.033	0	7
Zeb2	0	1.619085	0.889	0.39	0	7

Ms4a4c	0	1.613912	0.583	0.064	0	7
Ctsz	0	1.596136	0.926	0.576	0	7
Maf	0	1.568321	0.547	0.148	0	7
Ctsa	0	1.538068	0.843	0.392	0	7
Mpeg1	0	1.535803	0.797	0.229	0	7
Ly86	0	1.526751	0.741	0.045	0	7
Plac8	7.81E-100	1.525898	0.572	0.449	1.31E-95	7
Ctsd	2.63E-151	1.525054	0.841	0.861	4.40E-147	7
Lgals3	0	1.517071	0.872	0.681	0	7
Fcgr1	0	1.504826	0.62	0.025	0	7
C3ar1	0	1.503638	0.504	0.025	0	7
Ctsh	0	1.502374	0.898	0.478	0	7
Cfp	0	1.495672	0.761	0.369	0	7
AF251705	0	1.494534	0.716	0.205	0	7
Pld4	0	1.487046	0.748	0.049	0	7
Gngt2	0	1.474552	0.694	0.214	0	7
Cd79b	6.45E-111	1.471729	0.201	0.051	1.08E-106	7
Unc93b1	0	1.468609	0.878	0.412	0	7
Pltp	0	1.456445	0.445	0.077	0	7
Adgre1	0	1.42314	0.633	0.051	0	7
Cstb	0	1.40966	0.864	0.632	0	7
Clec4a1	0	1.406085	0.683	0.029	0	7
Ier3	1.06E-171	1.401993	0.687	0.523	1.77E-167	7
Ly6e	0	1.389025	0.935	0.715	0	7
Abca1	2.72E-306	1.387616	0.552	0.221	4.54E-302	7
H2-DMa	0	1.381628	0.653	0.116	0	7
Snx2	0	1.376688	0.766	0.414	0	7
Ahnak	0	1.367534	0.889	0.427	0	7
Ccl6	0	1.364639	0.799	0.332	0	7
Cst3	0	1.361624	0.946	0.856	0	7
Plin2	0	1.354788	0.807	0.541	0	7
Pid1	0	1.348709	0.688	0.083	0	7
Hexa	0	1.348225	0.799	0.492	0	7
Fn1	0	1.306252	0.599	0.201	0	7
Gm2a	0	1.296788	0.857	0.377	0	7
Clta	0	1.257826	0.942	0.844	0	7
Stab1	1.08E-301	1.248496	0.399	0.085	1.80E-297	7
Snx5	0	1.244486	0.783	0.375	0	7
Laptm5	0	1.241679	0.962	0.701	0	7
Napsa	1.05E-264	1.238285	0.63	0.345	1.76E-260	7
Cdkn1a	0	1.212711	0.709	0.306	0	7
Msr1	0	1.212444	0.551	0.019	0	7
Spp1	3.05E-154	1.210103	0.453	0.213	5.11E-150	7
Ifi27l2a	0	1.201337	0.736	0.354	0	7
Ehd4	0	1.187076	0.718	0.245	0	7
Klf4	0	1.180487	0.77	0.293	0	7
Thbs1	1.50E-208	1.163937	0.55	0.261	2.51E-204	7
Akr1a1	0	1.16356	0.867	0.614	0	7
Lst1	0	1.160328	0.804	0.489	0	7
Sdc3	0	1.151882	0.498	0.096	0	7
Sirpa	0	1.132818	0.784	0.485	0	7
Rgs10	0	1.128784	0.736	0.323	0	7

Cltc	1.62E-301	1.118982	0.85	0.694	2.72E-297	7
Cxcl16	0	1.109091	0.437	0.1	0	7
Fcer1g	0	1.106897	0.88	0.617	0	7
Atp2b1	0	1.093077	0.885	0.638	0	7
Ifi204	0	1.08909	0.632	0.175	0	7
Gm26917	1.45E-42	1.081499	0.585	0.492	2.42E-38	7
Erp29	0	1.078829	0.915	0.644	0	7
Ms4a6b	0	1.076472	0.764	0.222	0	7
Pycard	0	1.073279	0.828	0.58	0	7
Cndp2	0	1.066486	0.662	0.273	0	7
Rel	0	1.064022	0.764	0.422	0	7
BC005537	0	1.06031	0.839	0.635	0	7
Pou2f2	0	1.05857	0.419	0.051	0	7
Mef2c	8.34E-305	1.040816	0.586	0.224	1.39E-300	7
H2-DMb1	0	1.037441	0.42	0.061	0	7
Ms4a1	6.98E-102	1.033053	0.1	0.003	1.17E-97	7
Rassf4	0	1.031214	0.578	0.07	0	7
Itm2b	2.12E-300	1.031078	0.972	0.965	3.54E-296	7
Ucp2	0	1.01352	0.938	0.712	0	7
Crip1	1.06E-303	1.010289	0.92	0.554	1.78E-299	7
Anxa5	0	1.002672	0.845	0.545	0	7
Rab7b	0	1.002327	0.518	0.12	0	7
Mfsd1	0	########	0.673	0.32	0	7
Tob2	0	########	0.671	0.344	0	7
Ccl7	2.15E-60	########	0.292	0.157	3.59E-56	7
Ptpn18	0	########	0.928	0.629	0	7
Tgfb1	0	########	0.83	0.475	0	7
Bri3	0	########	0.865	0.702	0	7
Pitpna	0	########	0.892	0.778	0	7
B2m	0	########	0.988	0.957	0	7
Efhd2	0	########	0.836	0.637	0	7
S100a10	0	########	0.919	0.537	0	7
Asah1	0	########	0.762	0.438	0	7
Cbr2	4.79E-184	########	0.244	0.04	8.01E-180	7
Nrp1	2.59E-205	########	0.559	0.279	4.33E-201	7
Folr2	3.80E-303	########	0.28	0.01	6.36E-299	7
Fcrls	6.27E-257	########	0.242	0.009	1.05E-252	7
Cd163	3.59E-124	########	0.139	0.011	6.01E-120	7
Smpdl3a	2.58E-157	########	0.671	0.497	4.32E-153	7
Slc43a2	0	########	0.616	0.143	0	7
Cd38	0	########	0.5	0.137	0	7
Atox1	0	########	0.907	0.82	0	7
Lrp1	0	########	0.744	0.223	0	7
Prdx1	1.39E-292	########	0.92	0.615	2.33E-288	7
Gusb	0	########	0.705	0.352	0	7
Sh3bgrl	0	########	0.798	0.457	0	7
Cd93	0	########	0.57	0.142	0	7
Sdc4	1.62E-220	########	0.562	0.244	2.71E-216	7
Cyfip1	0	########	0.743	0.498	0	7
Atp6v0b	0	########	0.93	0.813	0	7
Serinc3	0	########	0.91	0.76	0	7
Gdi2	0	########	0.903	0.728	0	7

Hspa8	0	########	0.985	0.91	0	7
Creg1	3.49E-300	########	0.78	0.569	5.84E-296	7
Cyba	0	########	0.972	0.903	0	7
Fam96a	0	########	0.738	0.511	0	7
Capza2	0	########	0.925	0.851	0	7
Eps8	0	########	0.591	0.185	0	7
Gpx1	0	########	0.95	0.839	0	7
Blvrb	7.30E-233	########	0.641	0.364	1.22E-228	7
Ccl2	7.39E-142	########	0.444	0.195	1.24E-137	7
Rnh1	7.66E-290	########	0.806	0.597	1.28E-285	7
Rbpj	5.54E-247	########	0.706	0.493	9.27E-243	7
Irf5	0	########	0.607	0.065	0	7
Fos	3.09E-209	########	0.769	0.448	5.17E-205	7
Plekho1	0	########	0.65	0.144	0	7
Lyn	0	########	0.888	0.539	0	7
Ninj1	2.26E-298	########	0.688	0.376	3.78E-294	7
Ccl12	9.01E-179	########	0.18	0.009	1.51E-174	7
Lpl	1.48E-34	########	0.223	0.132	2.48E-30	7
Atp13a2	0	########	0.598	0.167	0	7
Jarid2	0	########	0.648	0.307	0	7
Glul	4.71E-216	########	0.624	0.367	7.87E-212	7
Ccr5	0	########	0.45	0.044	0	7
Eif4e	1.89E-221	########	0.672	0.419	3.16E-217	7
Fcgrt	1.98E-111	########	0.402	0.214	3.31E-107	7
Ap2a2	8.34E-233	########	0.67	0.447	1.40E-228	7
Rap1b	0	########	0.92	0.878	0	7
Vcan	7.54E-117	########	0.399	0.18	1.26E-112	7
Emp3	3.26E-307	########	0.914	0.782	5.45E-303	7
Naaa	0	########	0.487	0.136	0	7
Clec10a	5.01E-306	########	0.317	0.025	8.38E-302	7
Plxnb2	0	########	0.605	0.204	0	7
Nrros	0	########	0.642	0.237	0	7
Atp6v1a	2.22E-234	########	0.688	0.489	3.72E-230	7
Aprt	2.68E-190	########	0.822	0.672	4.49E-186	7
Dusp3	0	########	0.59	0.163	0	7
Ap2s1	3.26E-283	########	0.831	0.664	5.45E-279	7
Sdcbp	1.66E-274	########	0.885	0.813	2.78E-270	7
Camk2d	0	########	0.648	0.308	0	7
Metrnl	0	########	0.645	0.279	0	7
Sgpl1	0	########	0.646	0.301	0	7
Ednrb	1.34E-132	########	0.218	0.048	2.25E-128	7
Zfp703	4.70E-242	########	0.535	0.246	7.86E-238	7
Clec4a2	1.74E-219	########	0.713	0.363	2.92E-215	7
Tmem106a	0	########	0.489	0.059	0	7
Cd48	0	########	0.708	0.206	0	7
Hfe	0	########	0.471	0.107	0	7
Cd302	0	########	0.653	0.212	0	7
Rilpl2	1.03E-263	########	0.654	0.374	1.72E-259	7
Gna13	3.60E-248	########	0.727	0.524	6.02E-244	7
Prcp	0	########	0.648	0.221	0	7
Ebf1	1.63E-20	########	0.12	0.215	2.73E-16	7
Ccdc109b	0	########	0.575	0.182	0	7

Rab5c	2.80E-271	########	0.82	0.679	4.69E-267	7
Hexb	6.16E-189	########	0.598	0.387	1.03E-184	7
Tgif1	1.74E-281	########	0.624	0.29	2.92E-277	7
Cd83	0	########	0.306	0.012	0	7
Gpnmb	6.90E-91	########	0.16	0.038	1.15E-86	7
M6pr	0	########	0.717	0.434	0	7
Fcgr3	1.44E-236	########	0.766	0.459	2.41E-232	7
Cd300a	0	########	0.635	0.274	0	7
Abhd12	0	########	0.551	0.232	0	7
Dpep2	0	########	0.463	0.029	0	7
Nrp2	1.36E-172	########	0.394	0.154	2.27E-168	7
Mef2a	2.41E-262	########	0.754	0.531	4.03E-258	7
Mdm2	1.67E-169	########	0.641	0.468	2.79E-165	7
Nxpe5	0	########	0.355	0.012	0	7
F10	6.54E-225	########	0.393	0.114	1.09E-220	7
Ifngr1	8.75E-231	########	0.821	0.657	1.46E-226	7
Apobec1	0	########	0.555	0.142	0	7
Pyhin1	2.80E-260	########	0.473	0.133	4.68E-256	7
Dusp2	3.92E-219	########	0.506	0.201	6.57E-215	7
P2ry6	0	########	0.465	0.038	0	7
Glud1	1.78E-271	########	0.814	0.64	2.98E-267	7
Cln8	0	########	0.495	0.088	0	7
Nipa2	2.78E-288	########	0.678	0.412	4.66E-284	7
mt-Co3	4.97E-302	########	0.999	0.985	8.31E-298	7
Fabp5	1.37E-105	########	0.656	0.488	2.28E-101	7
Wwp1	3.71E-81	########	0.404	0.281	6.21E-77	7
Rab1a	1.48E-205	########	0.759	0.573	2.47E-201	7
Gns	1.31E-259	########	0.569	0.272	2.18E-255	7
Fam49b	4.83E-277	########	0.888	0.676	8.08E-273	7
Cxcr4	1.01E-207	########	0.665	0.39	1.68E-203	7
Rab3il1	2.48E-264	########	0.377	0.091	4.14E-260	7
Man2b1	8.78E-264	########	0.733	0.486	1.47E-259	7
Capg	1.98E-173	########	0.773	0.59	3.30E-169	7
Lat2	0	########	0.577	0.101	0	7
mt-Co2	0	########	0.998	0.982	0	7
Evi2a	0	########	0.683	0.313	0	7
Ifitm3	2.66E-76	########	0.849	0.752	4.46E-72	7
Jmjd1c	3.58E-171	########	0.752	0.599	5.99E-167	7
mt-Co1	0	########	0.998	0.99	0	7
Wsb1	3.99E-218	########	0.756	0.528	6.67E-214	7
Rnf130	6.90E-271	########	0.822	0.594	1.15E-266	7
Cd86	0	########	0.45	0.046	0	7
Bmp2k	2.74E-249	########	0.59	0.306	4.58E-245	7
Soat1	1.29E-249	########	0.678	0.431	2.16E-245	7
Lipa	8.90E-195	########	0.55	0.304	1.49E-190	7
Zfp36l1	4.61E-128	########	0.769	0.551	7.71E-124	7
Tpp1	1.25E-230	########	0.553	0.289	2.09E-226	7
Lpxn	0	########	0.498	0.049	0	7
Wnk1	1.07E-165	########	0.83	0.735	1.79E-161	7
Acer3	0	########	0.571	0.243	0	7
Sirpb1c	7.52E-271	########	0.468	0.119	1.26E-266	7
Cox4i1	0	########	0.974	0.945	0	7

Dbi	2.47E-292	########	0.875	0.602	4.13E-288	7
Ap1b1	6.98E-171	########	0.432	0.209	1.17E-166	7
Sqstm1	5.01E-172	########	0.719	0.523	8.38E-168	7
Trf	1.50E-216	########	0.437	0.149	2.51E-212	7
Man1a	2.52E-189	########	0.575	0.308	4.21E-185	7
Atp1a1	9.29E-243	########	0.805	0.496	1.55E-238	7
Tiparp	1.01E-145	########	0.64	0.444	1.70E-141	7
AI662270	0	########	0.675	0.269	0	7
Tnfsf9	1.02E-134	########	0.263	0.079	1.71E-130	7
Il10	6.57E-161	########	0.172	0.011	1.10E-156	7
Zfp36l2	4.32E-189	########	0.883	0.764	7.23E-185	7
Atp6v1b2	1.60E-229	########	0.724	0.525	2.68E-225	7
Dnase2a	0	########	0.504	0.133	0	7
Necap2	3.94E-290	########	0.68	0.421	6.59E-286	7
1-Mar	1.83E-218	########	0.417	0.134	3.07E-214	7
Zfp36	1.01E-189	########	0.866	0.707	1.69E-185	7
Arhgap17	1.09E-268	########	0.659	0.361	1.83E-264	7
Slc6a6	3.36E-218	########	0.764	0.56	5.62E-214	7
H2-K1	0	########	0.979	0.838	0	7
Junb	2.98E-179	########	0.919	0.799	4.99E-175	7
Bank1	7.79E-58	########	0.143	0.049	1.30E-53	7
BC028528	5.57E-207	########	0.493	0.235	9.32E-203	7
Tcirg1	2.94E-272	########	0.56	0.269	4.92E-268	7
Rnpep	1.80E-235	########	0.664	0.425	3.01E-231	7
Smdt1	8.56E-245	########	0.874	0.761	1.43E-240	7
Ap2m1	1.99E-112	########	0.704	0.577	3.33E-108	7
Cotl1	6.51E-216	########	0.912	0.771	1.09E-211	7
Pnp	1.45E-180	########	0.771	0.571	2.42E-176	7
Ifrd1	3.42E-102	########	0.688	0.582	5.73E-98	7
Snx3	4.55E-170	########	0.799	0.676	7.61E-166	7
Syngr2	1.20E-238	########	0.688	0.441	2.01E-234	7
Mdfic	1.99E-285	########	0.487	0.165	3.34E-281	7
Scpep1	0	########	0.544	0.186	0	7
5031439G(	1.01E-307	########	0.625	0.288	1.70E-303	7
Mrpl52	1.74E-224	########	0.87	0.581	2.92E-220	7
Slc3a2	1.43E-162	########	0.703	0.498	2.40E-158	7
Got1	1.55E-120	########	0.461	0.301	2.59E-116	7
mt-Nd5	1.01E-202	########	0.964	0.803	1.69E-198	7
Tep1	0	########	0.482	0.139	0	7
Mmp19	2.91E-194	########	0.364	0.115	4.87E-190	7
Emp1	3.49E-128	########	0.52	0.265	5.83E-124	7
Bax	8.91E-254	########	0.771	0.494	1.49E-249	7
Ubl3	5.02E-229	########	0.777	0.575	8.40E-225	7
Prdx2	1.36E-242	########	0.859	0.53	2.27E-238	7
Tlr2	1.70E-222	########	0.553	0.234	2.85E-218	7
Nr4a2	9.40E-138	########	0.432	0.204	1.57E-133	7
Psmb10	1.97E-248	########	0.735	0.469	3.29E-244	7
Stx7	3.38E-244	########	0.692	0.464	5.66E-240	7
Susd3	0	########	0.47	0.106	0	7
Gm6377	0	########	0.328	0.017	0	7
Rgl1	5.07E-157	########	0.355	0.141	8.48E-153	7
H3f3a	2.65E-286	########	0.987	0.99	4.43E-282	7

Cysltr1	0	########	0.411	0.031	0	7
Sh3bgrl3	3.06E-258	########	0.973	0.964	5.11E-254	7
Fam105a	7.64E-294	########	0.587	0.215	1.28E-289	7
Pla2g7	4.73E-262	########	0.7	0.271	7.91E-258	7
Adam15	1.54E-243	########	0.596	0.309	2.57E-239	7
Ptprj	3.13E-262	########	0.6	0.286	5.23E-258	7
Uvrag	1.82E-237	########	0.637	0.37	3.04E-233	7
Mtss1	4.47E-171	########	0.351	0.125	7.47E-167	7
Ikbkb	1.48E-197	########	0.637	0.401	2.48E-193	7
mt-Nd4	1.01E-197	########	0.995	0.933	1.68E-193	7
P2rx4	8.40E-283	########	0.487	0.17	1.41E-278	7
Dazap2	2.22E-228	########	0.878	0.772	3.71E-224	7
Mndal	5.29E-142	########	0.523	0.265	8.86E-138	7
Calm1	1.35E-249	########	0.982	0.978	2.26E-245	7
Qk	4.44E-221	########	0.77	0.574	7.43E-217	7
Slc38a1	7.99E-284	########	0.539	0.187	1.34E-279	7
Cebpb	7.67E-217	########	0.866	0.737	1.28E-212	7
Tnfrsf13b	4.31E-297	########	0.445	0.112	7.20E-293	7
Rpl10	3.60E-203	########	0.973	0.934	6.03E-199	7
Map4k4	1.93E-93	########	0.747	0.592	3.23E-89	7
Filip1l	3.57E-165	########	0.49	0.233	5.97E-161	7
Atp6ap1	1.25E-216	########	0.731	0.534	2.08E-212	7
Myo5a	5.97E-132	########	0.541	0.368	9.98E-128	7
Etv3	8.34E-154	########	0.424	0.198	1.39E-149	7
Tmem256	2.21E-207	########	0.799	0.56	3.69E-203	7
Cfh	2.29E-146	########	0.311	0.1	3.83E-142	7
Clec4n	2.10E-267	########	0.338	0.046	3.51E-263	7
Dusp5	4.93E-169	########	0.47	0.212	8.26E-165	7
Slc25a5	1.40E-208	########	0.904	0.774	2.34E-204	7
Cerk	1.94E-224	########	0.498	0.219	3.24E-220	7
Cx3cr1	8.41E-227	########	0.245	0.019	1.41E-222	7
Tmem14c	7.36E-191	########	0.836	0.713	1.23E-186	7
Rtn4	1.89E-160	########	0.832	0.679	3.16E-156	7
Sod1	3.55E-166	########	0.64	0.414	5.94E-162	7
Rap2b	9.36E-212	########	0.577	0.318	1.57E-207	7
Npc1	2.23E-206	########	0.498	0.229	3.73E-202	7
Fnip2	3.95E-212	########	0.429	0.156	6.62E-208	7
Fam213b	1.92E-198	########	0.356	0.113	3.22E-194	7
Ptp4a2	1.07E-194	########	0.87	0.728	1.80E-190	7
Psmb8	3.81E-194	########	0.858	0.678	6.37E-190	7
Tmem86a	3.72E-255	########	0.342	0.066	6.23E-251	7
Ecm1	7.79E-164	########	0.531	0.252	1.30E-159	7
Vamp8	6.49E-221	########	0.877	0.798	1.09E-216	7
Cd180	0	########	0.394	0.026	0	7
H2-DMb2	1.13E-90	########	0.126	0.019	1.89E-86	7
Coro1b	8.55E-202	########	0.656	0.461	1.43E-197	7
Pomp	3.76E-212	########	0.87	0.771	6.28E-208	7
Arhgdia	5.73E-196	########	0.864	0.72	9.59E-192	7
Bach1	2.97E-150	########	0.692	0.512	4.97E-146	7
Irf8	0	########	0.615	0.103	0	7
Skil	1.28E-117	########	0.596	0.421	2.14E-113	7
Dusp1	1.58E-89	########	0.689	0.515	2.64E-85	7

Abl2	9.82E-152	########	0.427	0.209	1.64E-147	7
Acp2	2.97E-217	########	0.389	0.127	4.97E-213	7
Sppl2a	1.43E-197	########	0.704	0.51	2.40E-193	7
Atp6ap2	5.14E-186	########	0.716	0.511	8.59E-182	7
Blvra	4.19E-209	########	0.531	0.289	7.01E-205	7
Epn1	3.79E-198	########	0.674	0.472	6.34E-194	7
Trim25	6.18E-187	########	0.589	0.327	1.03E-182	7
Sash1	3.09E-119	########	0.396	0.203	5.17E-115	7
Myo1g	1.28E-146	########	0.491	0.267	2.14E-142	7
Scamp2	1.36E-159	########	0.662	0.495	2.28E-155	7
Slc7a8	6.53E-283	########	0.342	0.052	1.09E-278	7
Man2a1	3.47E-135	########	0.668	0.473	5.81E-131	7
Snx6	7.55E-60	########	0.638	0.587	1.26E-55	7
Slfn5	1.07E-154	########	0.437	0.179	1.80E-150	7
Atf3	6.03E-102	########	0.48	0.26	1.01E-97	7
Ndufb8	4.02E-198	########	0.849	0.679	6.73E-194	7
Sap30	6.05E-165	########	0.497	0.272	1.01E-160	7
Sat1	4.56E-131	########	0.891	0.837	7.62E-127	7
Dock10	7.70E-235	########	0.629	0.29	1.29E-230	7
Serp1	8.77E-169	########	0.87	0.72	1.47E-164	7
Kdm6b	5.91E-84	########	0.702	0.613	9.88E-80	7
Tppp3	1.09E-68	########	0.257	0.123	1.82E-64	7
Gltp	8.35E-168	########	0.631	0.436	1.40E-163	7
Capzb	6.88E-235	########	0.918	0.875	1.15E-230	7
Tmem37	7.71E-227	########	0.291	0.044	1.29E-222	7
Ly6d	1.18E-33	########	0.145	0.068	1.98E-29	7
Dhrs3	2.65E-140	########	0.421	0.214	4.44E-136	7
Hn1	1.69E-182	########	0.822	0.626	2.83E-178	7
Sowahc	3.44E-148	########	0.364	0.155	5.76E-144	7
Tm6sf1	5.71E-184	########	0.712	0.436	9.56E-180	7
Atp6v1f	4.81E-235	########	0.876	0.81	8.05E-231	7
Arhgef10l	0	########	0.408	0.052	0	7
Fam174a	3.24E-190	########	0.594	0.36	5.43E-186	7
Zbtb7a	1.44E-147	########	0.703	0.545	2.40E-143	7
Mat2a	1.50E-133	########	0.71	0.492	2.50E-129	7
Mnda	4.98E-167	########	0.332	0.106	8.33E-163	7
Olfm1	1.35E-210	########	0.378	0.118	2.26E-206	7
Ap1s2	5.63E-113	########	0.578	0.413	9.42E-109	7
Trps1	1.23E-204	########	0.632	0.332	2.06E-200	7
Slc25a3	2.33E-188	########	0.932	0.815	3.89E-184	7
Lamtor1	5.39E-165	########	0.768	0.651	9.02E-161	7
Zbp1	1.70E-150	########	0.442	0.198	2.84E-146	7
Psma7	1.42E-137	########	0.878	0.778	2.38E-133	7
Pepd	1.06E-212	########	0.494	0.224	1.77E-208	7
Pip4k2a	7.22E-184	########	0.602	0.354	1.21E-179	7
Inpp5d	1.07E-222	########	0.665	0.353	1.79E-218	7
Nceh1	1.98E-144	########	0.449	0.237	3.32E-140	7
Zfand5	4.14E-125	########	0.803	0.674	6.93E-121	7
Fermt3	1.77E-190	########	0.732	0.429	2.97E-186	7
Zc3h12a	1.93E-146	########	0.461	0.236	3.23E-142	7
Rnase4	1.08E-124	########	0.456	0.222	1.80E-120	7
Npl	9.72E-111	########	0.2	0.052	1.63E-106	7

Ckb	5.48E-131	########	0.378	0.162	9.17E-127	7
Ccdc50	4.04E-163	########	0.567	0.329	6.76E-159	7
Frmd4b	1.10E-195	########	0.32	0.082	1.85E-191	7
Itgb5	7.18E-152	########	0.421	0.183	1.20E-147	7
Pcbp2	4.46E-167	########	0.897	0.785	7.47E-163	7
Lmo2	1.55E-195	########	0.536	0.254	2.59E-191	7
Rap1a	5.21E-160	########	0.85	0.761	8.71E-156	7
Rasgef1b	2.14E-238	########	0.286	0.04	3.58E-234	7
Ptgs2	1.14E-39	########	0.298	0.184	1.90E-35	7
Rgs2	2.40E-98	########	0.632	0.467	4.01E-94	7
Tor1aip1	1.43E-136	########	0.735	0.603	2.39E-132	7
Rnase6	2.02E-280	########	0.322	0.035	3.38E-276	7
Snx8	2.92E-229	########	0.33	0.075	4.89E-225	7
Vwa5a	6.52E-180	########	0.472	0.227	1.09E-175	7
Gnpda1	3.51E-220	########	0.45	0.169	5.88E-216	7
Anxa4	4.26E-165	########	0.429	0.203	7.14E-161	7
Adam17	4.07E-198	########	0.568	0.316	6.80E-194	7
mt-Nd1	3.80E-141	########	0.993	0.834	6.36E-137	7
Cmpk1	4.36E-159	########	0.768	0.629	7.30E-155	7
Gm42418	2.23E-108	########	1	1	3.73E-104	7
Lamp2	7.21E-195	########	0.856	0.744	1.21E-190	7
Rbm3	2.37E-154	########	0.943	0.839	3.97E-150	7
Foxp1	2.82E-75	########	0.756	0.589	4.72E-71	7
Ralgps2	1.93E-25	########	0.204	0.135	3.22E-21	7
Pold4	3.28E-127	########	0.683	0.511	5.48E-123	7
Ggh	1.46E-165	########	0.605	0.409	2.44E-161	7
Ptpro	0	########	0.349	0.009	0	7
Psmd8	8.28E-162	########	0.81	0.672	1.38E-157	7
Serpinb8	4.51E-290	########	0.322	0.037	7.54E-286	7
Tifa	1.23E-271	########	0.417	0.11	2.05E-267	7
Wdr26	3.38E-145	########	0.79	0.687	5.66E-141	7
Lhfpl2	1.89E-59	########	0.266	0.153	3.16E-55	7
Gch1	3.17E-176	########	0.446	0.188	5.31E-172	7
Fem1c	2.36E-167	########	0.563	0.325	3.95E-163	7
Canx	7.90E-134	########	0.797	0.576	1.32E-129	7
Grb2	4.86E-185	########	0.86	0.745	8.14E-181	7
Fchsd2	1.18E-46	########	0.289	0.185	1.97E-42	7
Il18	1.02E-275	########	0.359	0.064	1.71E-271	7
Gas7	1.16E-126	########	0.481	0.29	1.94E-122	7
Hilpda	1.73E-74	########	0.4	0.253	2.90E-70	7
Nsf	9.35E-179	########	0.545	0.311	1.56E-174	7
Cebpa	6.66E-213	########	0.427	0.156	1.11E-208	7
Ntpcr	8.99E-156	########	0.454	0.247	1.50E-151	7
Tmem261	1.71E-151	########	0.583	0.381	2.86E-147	7
Rbm47	5.76E-243	########	0.485	0.156	9.64E-239	7
Zmiz1	4.52E-142	########	0.637	0.441	7.57E-138	7
Parp14	2.29E-123	########	0.494	0.265	3.83E-119	7
mt-Nd3	6.90E-130	########	0.973	0.74	1.15E-125	7
Aplp2	1.29E-181	########	0.668	0.409	2.15E-177	7
Actb	3.54E-216	########	1	0.999	5.93E-212	7
Tlr7	4.17E-291	########	0.363	0.057	6.97E-287	7
Parp1	7.41E-170	########	0.493	0.246	1.24E-165	7

Vim	4.51E-164	########	0.94	0.865	7.54E-160	7
Brk1	1.43E-200	########	0.862	0.791	2.39E-196	7
Tyrobp	8.14E-136	########	0.898	0.617	1.36E-131	7
Rps9	5.84E-249	########	0.997	0.995	9.76E-245	7
Map3k1	8.47E-159	########	0.495	0.254	1.42E-154	7
Bin1	8.51E-117	########	0.503	0.305	1.42E-112	7
Abi1	2.63E-135	########	0.716	0.59	4.40E-131	7
Ccdc88a	4.43E-114	########	0.472	0.278	7.41E-110	7
Atp6v1h	9.14E-120	########	0.495	0.321	1.53E-115	7
Pkig	1.77E-159	########	0.573	0.324	2.95E-155	7
Lrpap1	5.91E-171	########	0.581	0.347	9.90E-167	7
Tmem160	8.86E-167	########	0.783	0.595	1.48E-162	7
Mpp1	4.45E-113	########	0.623	0.484	7.45E-109	7
Fam129b	4.19E-160	########	0.432	0.199	7.01E-156	7
Odc1	3.83E-38	########	0.47	0.367	6.40E-34	7
Ptpn6	7.21E-131	########	0.75	0.485	1.21E-126	7
Lgals3bp	7.18E-108	########	0.37	0.187	1.20E-103	7
Gla	2.41E-210	########	0.482	0.2	4.03E-206	7
Tpt1	5.31E-275	########	0.999	0.998	8.89E-271	7
Eif4a1	2.98E-159	########	0.921	0.762	4.99E-155	7
Dnajb14	1.05E-114	########	0.491	0.329	1.76E-110	7
Ifi203	2.63E-99	########	0.482	0.256	4.40E-95	7
Cyth4	7.24E-217	########	0.694	0.371	1.21E-212	7
Plekho2	3.50E-183	########	0.565	0.312	5.85E-179	7
Fnip1	1.66E-119	########	0.566	0.394	2.78E-115	7
Syngr1	1.23E-60	########	0.178	0.073	2.05E-56	7
Camk1d	6.50E-242	########	0.457	0.135	1.09E-237	7
Atf6	4.49E-176	########	0.499	0.261	7.51E-172	7
Ms4a8a	0	########	0.287	0.006	0	7
Rpl36al	2.92E-145	########	0.965	0.739	4.88E-141	7
Eif3k	5.09E-148	########	0.905	0.817	8.51E-144	7
Arpc2	3.96E-196	########	0.969	0.956	6.63E-192	7
Itga4	6.13E-139	########	0.617	0.361	1.03E-134	7
Cfl1	7.02E-223	########	0.98	0.969	1.17E-218	7
Fau	2.70E-171	########	0.99	0.995	4.51E-167	7
Fam46a	9.41E-97	########	0.585	0.382	1.57E-92	7
Eif3a	1.68E-130	########	0.849	0.654	2.81E-126	7
Hint1	3.31E-157	########	0.923	0.677	5.54E-153	7
Pim1	1.42E-144	########	0.81	0.698	2.38E-140	7
Gab1	4.39E-162	########	0.316	0.102	7.35E-158	7
Ywhae	3.22E-141	########	0.877	0.708	5.39E-137	7
Tmem176b	1.21E-35	########	0.361	0.251	2.03E-31	7
Idh1	5.57E-91	########	0.486	0.354	9.33E-87	7
Nckap1l	3.73E-189	########	0.624	0.335	6.24E-185	7
Slc29a3	2.36E-201	########	0.396	0.14	3.95E-197	7
Ppt1	1.85E-155	########	0.679	0.5	3.09E-151	7
Map2k3	1.42E-89	########	0.642	0.528	2.38E-85	7
mt-Atp6	6.78E-119	########	0.998	0.982	1.13E-114	7
Eif3f	3.99E-139	########	0.919	0.817	6.68E-135	7
Gls	5.96E-102	########	0.731	0.566	9.97E-98	7
Gas2l3	1.39E-153	########	0.334	0.122	2.32E-149	7
Samhd1	3.42E-121	########	0.852	0.703	5.72E-117	7

Slc11a1	1.31E-218	########	0.52	0.191	2.19E-214	7
Aup1	4.89E-151	########	0.606	0.422	8.19E-147	7
Lactb	1.15E-145	########	0.479	0.281	1.92E-141	7
Cox5a	2.72E-144	########	0.906	0.777	4.55E-140	7
Glmp	1.00E-136	########	0.582	0.41	1.68E-132	7
Rbbp6	2.36E-107	########	0.717	0.568	3.94E-103	7
Atf4	6.09E-103	########	0.75	0.563	1.02E-98	7
H2-Ob	2.30E-61	########	0.109	0.025	3.84E-57	7
Trappc2l	1.20E-130	########	0.666	0.518	2.00E-126	7
Il6ra	2.45E-148	########	0.624	0.378	4.10E-144	7
Ndufa6	1.33E-126	########	0.863	0.784	2.22E-122	7
Taok3	3.02E-133	########	0.613	0.425	5.05E-129	7
Arl8b	1.15E-124	########	0.548	0.375	1.92E-120	7
Ube2l3	1.57E-145	########	0.826	0.726	2.62E-141	7
Adssl1	6.99E-148	########	0.548	0.294	1.17E-143	7
Ptpn1	1.61E-112	########	0.803	0.74	2.69E-108	7
Slc15a3	2.79E-196	########	0.591	0.251	4.68E-192	7
Uap1l1	3.99E-107	########	0.382	0.218	6.68E-103	7
Nptn	1.44E-128	########	0.707	0.539	2.42E-124	7
Pld3	1.30E-79	########	0.372	0.228	2.17E-75	7
Cd2ap	5.31E-131	########	0.529	0.297	8.89E-127	7
Tnfrsf11a	2.66E-274	########	0.282	0.022	4.45E-270	7
Ifnar2	4.98E-142	########	0.683	0.524	8.33E-138	7
Amdhd2	2.21E-120	########	0.456	0.274	3.70E-116	7
Casp8	2.56E-156	########	0.581	0.363	4.29E-152	7
Tbxas1	2.45E-237	########	0.31	0.053	4.10E-233	7
Il10ra	8.56E-282	########	0.44	0.095	1.43E-277	7
Hmox2	1.37E-126	########	0.602	0.44	2.29E-122	7
D8Ertd738e	5.65E-144	########	0.881	0.841	9.45E-140	7
Dok2	3.68E-144	########	0.394	0.166	6.15E-140	7
Spty2d1	6.71E-93	########	0.562	0.417	1.12E-88	7
Pi4k2a	4.05E-152	########	0.463	0.249	6.78E-148	7
Cox6b1	6.10E-182	########	0.942	0.885	1.02E-177	7
Rab14	5.59E-126	########	0.81	0.726	9.35E-122	7
Tnfrsf1b	4.64E-229	########	0.576	0.244	7.76E-225	7
Snx1	6.72E-130	########	0.55	0.383	1.12E-125	7
App	7.72E-102	########	0.752	0.638	1.29E-97	7
B4galt6	2.76E-136	########	0.397	0.189	4.62E-132	7
Bscl2	1.99E-145	########	0.524	0.32	3.33E-141	7
Raph1	1.06E-113	########	0.399	0.208	1.77E-109	7
Blnk	1.48E-201	########	0.246	0.029	2.47E-197	7
Cux1	5.76E-147	########	0.719	0.538	9.65E-143	7
B3gnt5	8.79E-146	########	0.267	0.069	1.47E-141	7
Camk1	2.88E-113	########	0.433	0.247	4.81E-109	7
B4galt5	7.86E-156	########	0.421	0.194	1.32E-151	7
Ppig	2.04E-97	########	0.747	0.613	3.42E-93	7
Tifab	8.02E-239	########	0.278	0.032	1.34E-234	7
Arl4c	3.36E-153	########	0.595	0.296	5.62E-149	7
Spag9	7.36E-136	########	0.806	0.644	1.23E-131	7
Pdxk	3.75E-186	0.414753	0.365	0.127	6.27E-182	7
Dse	1.24E-137	########	0.388	0.169	2.07E-133	7
Lifr	3.21E-139	########	0.327	0.122	5.37E-135	7

Arhgap18	5.66E-128	########	0.417	0.21	9.47E-124	7
Atp5a1	4.23E-131	########	0.888	0.718	7.08E-127	7
Slc37a2	2.64E-253	########	0.278	0.026	4.41E-249	7
Marcks	3.04E-115	########	0.775	0.554	5.09E-111	7
Fndc3a	5.74E-107	########	0.514	0.348	9.60E-103	7
Vps26a	1.14E-129	########	0.54	0.364	1.91E-125	7
Gm15987	1.58E-183	########	0.207	0.02	2.64E-179	7
Vat1	6.10E-43	########	0.382	0.285	1.02E-38	7
Tpd52	1.30E-272	########	0.871	0.555	2.17E-268	7
Scarb2	3.35E-138	########	0.598	0.387	5.60E-134	7
Stxbp3	2.18E-104	########	0.511	0.347	3.65E-100	7
Shtn1	8.58E-249	########	0.326	0.056	1.44E-244	7
Bcl2a1b	9.54E-164	########	0.353	0.105	1.60E-159	7
Cd36	1.62E-92	########	0.209	0.061	2.70E-88	7
Srsf9	2.22E-118	########	0.697	0.529	3.71E-114	7
Kxd1	3.27E-118	########	0.59	0.424	5.47E-114	7
Ndufs8	7.65E-110	########	0.668	0.51	1.28E-105	7
Sertad1	1.06E-94	########	0.566	0.406	1.77E-90	7
Basp1	1.92E-98	########	0.454	0.255	3.22E-94	7
Csrnp1	2.47E-102	########	0.489	0.298	4.14E-98	7
Prkcd	4.19E-120	########	0.682	0.489	7.02E-116	7
Epb41l2	6.77E-134	########	0.497	0.268	1.13E-129	7
Atp5c1	1.03E-116	########	0.868	0.767	1.72E-112	7
Ms4a4a	0	########	0.301	0.006	0	7
Zswim6	1.58E-135	########	0.473	0.249	2.64E-131	7
Gm9733	2.95E-59	########	0.283	0.155	4.94E-55	7
Fyb	7.14E-108	########	0.783	0.589	1.20E-103	7
Edem1	9.19E-110	########	0.597	0.458	1.54E-105	7
Mapre2	8.60E-117	########	0.507	0.33	1.44E-112	7
Tuba1c	2.78E-83	########	0.628	0.473	4.66E-79	7
Gng2	2.50E-145	########	0.685	0.448	4.18E-141	7
Clcn5	1.42E-110	########	0.306	0.134	2.37E-106	7
Vps35	1.02E-93	########	0.648	0.541	1.70E-89	7
Renbp	8.88E-208	########	0.329	0.085	1.49E-203	7
Runx1	1.33E-129	########	0.701	0.458	2.22E-125	7
Atpif1	1.36E-114	########	0.841	0.656	2.28E-110	7
Ndufc2	8.44E-125	########	0.794	0.583	1.41E-120	7
Atp6v0d1	1.98E-124	########	0.699	0.563	3.30E-120	7
Mapkapk2	8.44E-134	########	0.709	0.568	1.41E-129	7
Rab20	8.32E-111	########	0.454	0.26	1.39E-106	7
Snx30	2.71E-185	########	0.296	0.072	4.53E-181	7
Nr4a1	1.52E-137	########	0.53	0.264	2.54E-133	7
Tbc1d9	8.62E-272	########	0.29	0.027	1.44E-267	7
Ppp2ca	8.67E-97	########	0.824	0.745	1.45E-92	7
Rab11fip5	5.99E-129	########	0.307	0.12	1.00E-124	7
Cdc42	1.75E-219	########	0.971	0.959	2.92E-215	7
Zfp706	1.46E-106	########	0.727	0.562	2.44E-102	7
Cttnbp2nl	2.33E-169	########	0.33	0.109	3.90E-165	7
Adam9	2.42E-131	########	0.423	0.214	4.05E-127	7
Atp5d	2.79E-127	########	0.884	0.761	4.66E-123	7
Tns3	4.68E-142	########	0.384	0.175	7.82E-138	7
Mob1a	1.42E-104	########	0.722	0.62	2.38E-100	7

Atp6v1c1	1.37E-100	########	0.49	0.341	2.29E-96	7
Arl5c	3.36E-154	########	0.324	0.095	5.62E-150	7
Apobec3	1.95E-61	########	0.506	0.364	3.26E-57	7
Hopx	4.92E-34	########	0.336	0.245	8.24E-30	7
Pcbp1	2.78E-107	########	0.841	0.669	4.65E-103	7
Cmtm7	1.93E-174	########	0.774	0.59	3.23E-170	7
Atp5e	8.38E-179	########	0.96	0.924	1.40E-174	7
Zfp91	1.92E-99	########	0.723	0.554	3.21E-95	7
Nedd8	1.37E-130	########	0.884	0.814	2.29E-126	7
H2afy	1.09E-122	########	0.831	0.74	1.82E-118	7
Rragc	9.38E-82	########	0.506	0.389	1.57E-77	7
Hpse	6.32E-208	########	0.317	0.076	1.06E-203	7
Scand1	1.02E-121	########	0.879	0.849	1.70E-117	7
Tapbp	4.55E-106	########	0.697	0.551	7.61E-102	7
2610001J0	3.36E-112	########	0.54	0.376	5.63E-108	7
Ubash3b	4.05E-157	########	0.424	0.188	6.77E-153	7
Dcxr	1.77E-173	########	0.373	0.14	2.96E-169	7
Akr1b3	2.28E-116	########	0.617	0.405	3.82E-112	7
Fcgr4	4.27E-72	########	0.415	0.247	7.15E-68	7
Cytip	3.00E-44	########	0.637	0.532	5.02E-40	7
Dnajc13	9.02E-81	########	0.481	0.352	1.51E-76	7
Cox7a2l	3.94E-119	########	0.905	0.844	6.60E-115	7
Abi3	1.34E-148	########	0.29	0.084	2.24E-144	7
Ear2	7.16E-32	########	0.142	0.07	1.20E-27	7
Lair1	2.96E-143	########	0.365	0.142	4.95E-139	7
Gnb2l1	3.34E-102	########	0.99	0.867	5.58E-98	7
Frrs1	2.48E-127	########	0.437	0.248	4.15E-123	7
Tmem8	1.19E-70	########	0.223	0.098	1.99E-66	7
Stard8	5.20E-85	########	0.244	0.103	8.71E-81	7
Rpl26	1.84E-111	########	0.989	0.951	3.09E-107	7
Tmem189	8.61E-125	########	0.555	0.364	1.44E-120	7
Ube2f	1.70E-114	########	0.565	0.412	2.85E-110	7
Adgre4	8.43E-136	########	0.137	0.006	1.41E-131	7
Acp5	8.32E-38	########	0.303	0.191	1.39E-33	7
Il4ra	1.17E-52	########	0.578	0.455	1.96E-48	7
Twf1	1.95E-62	########	0.475	0.344	3.26E-58	7
Ccnl1	7.82E-121	########	0.825	0.697	1.31E-116	7
Adora2b	1.28E-212	########	0.281	0.046	2.15E-208	7
Arpc4	2.94E-110	########	0.84	0.764	4.91E-106	7
G3bp2	7.85E-100	########	0.707	0.582	1.31E-95	7
1700017B0	1.76E-112	########	0.39	0.201	2.94E-108	7
Havcr2	1.53E-221	########	0.232	0.016	2.55E-217	7
Tmem219	2.18E-112	########	0.552	0.39	3.65E-108	7
Dnase1l1	5.78E-202	########	0.373	0.121	9.68E-198	7
Zeb2os	5.11E-278	########	0.344	0.053	8.56E-274	7
Itm2c	1.87E-114	########	0.596	0.361	3.12E-110	7
Hprt	3.53E-97	########	0.633	0.478	5.91E-93	7
Mertk	2.92E-185	########	0.237	0.035	4.89E-181	7
Klf6	2.70E-54	########	0.846	0.812	4.52E-50	7
Csf2ra	4.21E-112	########	0.655	0.421	7.05E-108	7
Ppp1r10	2.90E-75	########	0.65	0.543	4.85E-71	7
Fosb	5.19E-111	########	0.612	0.346	8.69E-107	7

Gcsh	4.41E-85	########	0.393	0.227	7.38E-81	7
Tmed5	8.92E-107	########	0.736	0.582	1.49E-102	7
Daglb	1.50E-160	########	0.393	0.169	2.52E-156	7
Irf2bp2	1.90E-67	########	0.661	0.559	3.17E-63	7
mt-Nd2	4.20E-95	########	0.993	0.807	7.02E-91	7
Marcksl1	7.95E-88	########	0.561	0.421	1.33E-83	7
Ddi2	7.92E-97	########	0.483	0.324	1.33E-92	7
Use1	1.94E-102	########	0.79	0.721	3.25E-98	7
Rpl35a	1.71E-160	########	0.992	0.971	2.86E-156	7
Arap1	3.72E-127	########	0.509	0.293	6.22E-123	7
Rps27l	1.36E-120	########	0.86	0.647	2.28E-116	7
Atp5g2	2.05E-117	########	0.926	0.724	3.43E-113	7
Traf1	1.87E-112	########	0.354	0.15	3.12E-108	7
Rnf213	2.88E-75	########	0.446	0.291	4.83E-71	7
Arl8a	2.05E-101	########	0.486	0.327	3.44E-97	7
Lrrc25	1.47E-234	########	0.489	0.161	2.46E-230	7
Gpr183	8.06E-142	########	0.36	0.12	1.35E-137	7
Agpat4	3.17E-108	########	0.411	0.214	5.30E-104	7
Snrpb2	9.42E-96	########	0.608	0.466	1.58E-91	7
Rsrp1	1.47E-128	########	0.949	0.921	2.46E-124	7
Sfpq	1.91E-76	########	0.825	0.717	3.19E-72	7
Gabarap	4.01E-140	########	0.952	0.937	6.72E-136	7
Chchd10	1.54E-07	########	0.168	0.133	########	7
Ssfa2	2.36E-149	########	0.406	0.176	3.95E-145	7
Actr2	1.24E-111	########	0.903	0.851	2.08E-107	7
Phlda1	6.29E-25	########	0.389	0.313	1.05E-20	7
Gna12	1.39E-69	########	0.347	0.222	2.32E-65	7
Rps29	4.16E-276	########	0.994	0.989	6.95E-272	7
Rac1	3.24E-131	########	0.883	0.857	5.42E-127	7
Hpgds	9.35E-168	########	0.2	0.022	1.56E-163	7
Capn2	8.15E-139	########	0.518	0.267	1.36E-134	7
Tubb6	8.07E-134	########	0.539	0.256	1.35E-129	7
Cxcl10	1.48E-14	########	0.117	0.072	2.48E-10	7
Eef1a1	9.86E-90	########	0.998	0.978	1.65E-85	7
Ndufb5	5.07E-111	########	0.825	0.647	8.49E-107	7
Psmb4	5.71E-109	########	0.849	0.701	9.56E-105	7
Lamtor5	4.86E-112	########	0.706	0.578	8.13E-108	7
Ahr	8.80E-111	########	0.303	0.123	1.47E-106	7
H3f3b	2.64E-149	########	0.992	0.989	4.41E-145	7
Tmem51	1.62E-203	########	0.294	0.06	2.71E-199	7
Slc31a1	3.28E-87	########	0.422	0.278	5.48E-83	7
Tmem50a	8.02E-104	########	0.862	0.824	1.34E-99	7
Abcg1	8.71E-87	########	0.334	0.165	1.46E-82	7
Slc48a1	5.13E-35	########	0.371	0.296	8.59E-31	7
Rps25	2.49E-120	########	0.985	0.952	4.16E-116	7
Dmxl1	1.01E-60	########	0.415	0.293	1.68E-56	7
Stard3nl	4.45E-128	########	0.475	0.282	7.45E-124	7
Cmklr1	1.52E-227	########	0.271	0.034	2.55E-223	7
Ten1	1.45E-109	########	0.532	0.369	2.43E-105	7
Ppp1r15a	2.03E-113	########	0.628	0.393	3.40E-109	7
Spred1	3.80E-91	########	0.377	0.21	6.36E-87	7
Mef2d	1.87E-103	########	0.508	0.326	3.13E-99	7

0610012G0	2.05E-90	########	0.634	0.477	3.42E-86	7
Cbfa2t3	4.84E-119	########	0.276	0.093	8.10E-115	7
Cmtm3	1.65E-121	########	0.378	0.18	2.76E-117	7
Coro1c	1.05E-95	########	0.533	0.375	1.75E-91	7
Nfkb1	1.99E-80	########	0.716	0.585	3.34E-76	7
Spi1	1.26E-132	########	0.851	0.491	2.10E-128	7
Runx3	1.91E-149	########	0.434	0.175	3.20E-145	7
Xrn2	1.63E-108	########	0.719	0.54	2.72E-104	7
Calm2	8.91E-82	########	0.929	0.87	1.49E-77	7
Fkbp2	3.62E-93	########	0.682	0.468	6.06E-89	7
Hsd17b12	3.05E-103	########	0.531	0.356	5.10E-99	7
Hsp90aa1	6.94E-89	########	0.903	0.728	1.16E-84	7
Epsti1	6.38E-216	########	0.636	0.263	1.07E-211	7
Tmem55b	1.25E-101	########	0.433	0.268	2.08E-97	7
Tmed10	3.73E-104	########	0.847	0.739	6.24E-100	7
Acaa1a	2.04E-99	########	0.577	0.44	3.41E-95	7
Adap2	4.16E-245	########	0.264	0.023	6.96E-241	7
Brd2	1.25E-90	########	0.747	0.608	2.10E-86	7
Eps15	2.60E-66	########	0.523	0.419	4.35E-62	7
Hexim1	3.89E-66	########	0.498	0.363	6.51E-62	7
Rest	1.43E-66	########	0.531	0.413	2.39E-62	7
Atp5l	5.26E-159	########	0.955	0.951	8.80E-155	7
Dram2	2.17E-122	########	0.441	0.248	3.64E-118	7
Cela1	5.42E-119	########	0.197	0.045	9.07E-115	7
Tab2	5.36E-90	########	0.643	0.515	8.96E-86	7
Cwc15	9.41E-94	########	0.748	0.643	1.57E-89	7
Siglecg	5.63E-08	########	0.11	0.083	########	7
Sh3glb1	1.22E-94	########	0.897	0.794	2.04E-90	7
2010107E0	1.07E-108	########	0.875	0.77	1.78E-104	7
Nus1	1.31E-71	########	0.506	0.354	2.19E-67	7
Rhoa	2.54E-136	########	0.954	0.933	4.25E-132	7
Pkib	3.18E-233	########	0.295	0.041	5.32E-229	7
Osm	5.54E-100	########	0.303	0.125	9.28E-96	7
Sptssa	3.64E-79	########	0.657	0.512	6.10E-75	7
Man1c1	1.05E-99	########	0.327	0.166	1.76E-95	7
Sub1	3.32E-120	########	0.924	0.901	5.56E-116	7
Fam49a	1.54E-137	########	0.289	0.089	2.57E-133	7
Reep5	2.37E-147	########	0.899	0.751	3.97E-143	7
Synj1	4.12E-116	########	0.543	0.348	6.89E-112	7
Ranbp2	1.37E-67	########	0.572	0.448	2.29E-63	7
Gnb1	8.63E-84	########	0.823	0.716	1.44E-79	7
Irf7	1.58E-47	########	0.311	0.184	2.64E-43	7
Srgap2	2.14E-96	########	0.442	0.283	3.59E-92	7
Slc16a10	9.40E-208	########	0.309	0.066	1.57E-203	7
Nuak2	1.67E-152	########	0.351	0.126	2.79E-148	7
Syk	3.22E-158	########	0.737	0.449	5.39E-154	7
Limd1	4.87E-102	########	0.472	0.287	8.16E-98	7
Trafd1	1.31E-85	########	0.421	0.264	2.19E-81	7
H2-M3	5.42E-160	########	0.403	0.158	9.07E-156	7
Pgap1	1.29E-63	########	0.18	0.069	2.16E-59	7
Osbpl8	2.18E-42	########	0.479	0.398	3.65E-38	7
Dync1i2	4.05E-67	########	0.737	0.672	6.78E-63	7

Cd72	2.06E-51	########	0.141	0.05	3.44E-47	7
Hacd4	2.59E-94	########	0.602	0.404	4.34E-90	7
Nufip1	2.83E-88	########	0.351	0.195	4.73E-84	7
Ube2a	1.08E-82	########	0.664	0.553	1.81E-78	7
Trappc1	4.38E-80	########	0.559	0.442	7.32E-76	7
Plbd2	3.05E-119	########	0.385	0.199	5.10E-115	7
Sdhb	3.99E-85	########	0.719	0.622	6.68E-81	7
Osgep	1.20E-102	########	0.498	0.324	2.00E-98	7
Usf2	1.72E-88	########	0.646	0.539	2.87E-84	7
Atp6v0e	2.41E-125	########	0.903	0.872	4.03E-121	7
Ctage5	1.75E-68	########	0.671	0.599	2.92E-64	7
Rgs1	2.01E-113	########	0.206	0.048	3.37E-109	7
Gpr137b	2.19E-156	########	0.346	0.116	3.67E-152	7
Txnrd1	2.72E-49	########	0.592	0.504	4.55E-45	7
Comt	3.20E-87	########	0.52	0.343	5.36E-83	7
Ybx1	4.60E-144	########	0.96	0.871	7.70E-140	7
Cnih4	1.38E-77	########	0.702	0.567	2.30E-73	7
mt-Atp8	9.84E-78	########	0.806	0.674	1.65E-73	7
Tfec	4.37E-196	########	0.274	0.051	7.31E-192	7
Ik	4.39E-88	########	0.645	0.529	7.34E-84	7
Kctd12	7.63E-109	########	0.709	0.527	1.28E-104	7
Slc35f6	1.94E-134	########	0.372	0.169	3.25E-130	7
Plbd1	2.23E-74	########	0.604	0.373	3.73E-70	7
Plcg2	7.22E-98	########	0.479	0.292	1.21E-93	7
Emb	3.65E-52	########	0.773	0.711	6.11E-48	7
Eif5b	1.11E-82	########	0.744	0.559	1.86E-78	7
Etv6	1.30E-93	########	0.447	0.285	2.18E-89	7
Mapk6	1.18E-70	########	0.472	0.329	1.97E-66	7
Sdf2l1	1.61E-105	########	0.471	0.277	2.70E-101	7
Vapa	1.77E-87	########	0.827	0.743	2.96E-83	7
Clic1	1.12E-107	########	0.946	0.935	1.88E-103	7
Ly96	2.91E-140	########	0.42	0.198	4.87E-136	7
Ubc	1.43E-126	########	0.964	0.939	2.38E-122	7
Fgd2	6.96E-200	########	0.261	0.04	1.16E-195	7
Prpf40a	4.78E-79	########	0.741	0.633	7.99E-75	7
Pgls	6.73E-99	########	0.818	0.638	1.13E-94	7
H2-D1	3.73E-108	########	0.983	0.964	6.24E-104	7
Tm2d2	4.59E-86	########	0.599	0.459	7.69E-82	7
Rasa4	2.05E-174	########	0.286	0.068	3.43E-170	7
Btf3	6.17E-105	########	0.956	0.888	1.03E-100	7
Psmb1	6.34E-95	########	0.895	0.788	1.06E-90	7
AI413582	9.23E-121	########	0.496	0.301	1.54E-116	7
Plekhb2	1.08E-132	########	0.346	0.142	1.81E-128	7
Magt1	3.09E-73	########	0.531	0.392	5.17E-69	7
Uqcrq	2.41E-106	########	0.892	0.79	4.03E-102	7
Sgk3	2.31E-87	########	0.491	0.331	3.87E-83	7
Rps26	1.43E-105	0.325935	0.99	0.915	2.39E-101	7
Ndufc1	7.79E-83	########	0.766	0.593	1.30E-78	7
Rps19	3.63E-111	########	0.993	0.906	6.07E-107	7
Nubp1	7.22E-92	########	0.512	0.345	1.21E-87	7
Mrpl54	4.82E-87	########	0.673	0.478	8.07E-83	7
Hspa5	6.92E-102	########	0.914	0.772	1.16E-97	7

Tomm20	1.29E-84	########	0.856	0.678	2.17E-80	7
Ssbp4	6.71E-88	########	0.582	0.435	1.12E-83	7
Rpl7a	3.31E-83	########	0.97	0.878	5.54E-79	7
Dennd1a	2.31E-108	########	0.343	0.164	3.87E-104	7
Malt1	4.52E-69	########	0.362	0.216	7.57E-65	7
Lamtor3	1.73E-80	########	0.568	0.447	2.89E-76	7
Sys1	1.25E-78	########	0.731	0.65	2.09E-74	7
Eif3h	3.10E-80	########	0.87	0.748	5.19E-76	7
Naa50	4.08E-59	########	0.599	0.44	6.83E-55	7
Hsd17b4	2.78E-99	########	0.435	0.271	4.66E-95	7
mt-Nd4l	1.67E-84	########	0.863	0.656	2.79E-80	7
Polr2l	4.16E-20	########	0.35	0.298	6.97E-16	7
Cers2	3.11E-76	########	0.605	0.492	5.21E-72	7
Rnf150	1.81E-94	########	0.241	0.093	3.03E-90	7
Rnf19b	3.77E-57	########	0.522	0.405	6.31E-53	7
Commd4	3.46E-89	########	0.545	0.397	5.80E-85	7
mt-Cytb	2.18E-68	########	0.994	0.939	3.64E-64	7
Iqgap2	3.05E-61	########	0.472	0.342	5.10E-57	7
Slc38a2	5.88E-70	########	0.739	0.611	9.85E-66	7
Card19	4.75E-110	########	0.746	0.634	7.95E-106	7
Aph1a	8.99E-82	########	0.552	0.416	1.50E-77	7
Adrbk2	1.32E-121	########	0.191	0.039	2.22E-117	7
Csk	3.51E-69	########	0.598	0.491	5.88E-65	7
Arl6ip1	6.06E-78	########	0.839	0.755	1.01E-73	7
Fkbp15	3.97E-71	########	0.438	0.301	6.64E-67	7
2310022A1	2.76E-159	########	0.3	0.091	4.62E-155	7
Slc9a9	6.94E-118	########	0.277	0.094	1.16E-113	7
Cflar	2.41E-61	########	0.661	0.588	4.03E-57	7
Nfe2l2	7.62E-65	########	0.744	0.68	1.27E-60	7
Picalm	9.13E-123	########	0.857	0.748	1.53E-118	7
Nfkbie	2.47E-121	########	0.36	0.154	4.12E-117	7
Emilin2	3.19E-151	########	0.721	0.435	5.34E-147	7
Nmt1	3.17E-62	########	0.62	0.528	5.31E-58	7
Prpf38b	1.69E-61	########	0.662	0.566	2.83E-57	7
Gas6	1.36E-23	########	0.227	0.156	2.28E-19	7
Stom	1.01E-95	########	0.379	0.217	1.69E-91	7
Ano6	2.43E-53	########	0.48	0.381	4.06E-49	7
Ddrgk1	7.56E-85	########	0.623	0.438	1.26E-80	7
Smagp	4.35E-130	########	0.262	0.074	7.28E-126	7
Atp5g3	1.33E-77	########	0.839	0.668	2.23E-73	7
Gpr132	3.55E-148	########	0.321	0.094	5.93E-144	7
Nfkbib	1.64E-84	########	0.488	0.341	2.75E-80	7
Tbcb	5.31E-71	########	0.727	0.576	8.88E-67	7
Fam102b	1.08E-73	########	0.388	0.232	1.81E-69	7
Il10rb	1.57E-104	########	0.613	0.44	2.63E-100	7
Dpysl2	4.27E-93	########	0.431	0.248	7.15E-89	7
Itgav	1.14E-39	########	0.394	0.29	1.91E-35	7
Bcl2a1d	8.21E-131	########	0.182	0.027	1.37E-126	7
Nsa2	1.27E-100	########	0.884	0.745	2.12E-96	7
Polr1d	2.19E-91	########	0.854	0.734	3.66E-87	7
Tnip3	3.60E-135	########	0.138	0.006	6.02E-131	7
Mfsd11	1.56E-76	########	0.319	0.181	2.61E-72	7

Rps11	3.24E-93	########	0.988	0.947	5.43E-89	7
Nfat5	3.02E-45	########	0.557	0.458	5.05E-41	7
Map7d1	2.16E-64	########	0.585	0.469	3.61E-60	7
Pin4	5.66E-69	########	0.627	0.521	9.47E-65	7
Vps29	7.66E-66	########	0.713	0.611	1.28E-61	7
Dek	8.48E-59	########	0.777	0.584	1.42E-54	7
Ddx3x	1.40E-69	########	0.77	0.675	2.34E-65	7
Fuca1	1.02E-84	########	0.713	0.611	1.71E-80	7
Mtpn	1.84E-60	########	0.742	0.658	3.07E-56	7
Eif4g3	5.86E-61	########	0.584	0.49	9.80E-57	7
Zfand2a	1.33E-113	########	0.275	0.101	2.22E-109	7
Cacul1	1.51E-80	########	0.376	0.226	2.53E-76	7
C1galt1c1	3.41E-39	########	0.289	0.201	5.71E-35	7
Ifnar1	5.36E-86	########	0.504	0.348	8.96E-82	7
Slc7a7	5.92E-167	########	0.291	0.079	9.91E-163	7
Gpr35	3.22E-220	########	0.374	0.083	5.39E-216	7
Tpp2	1.10E-65	########	0.571	0.451	1.84E-61	7
Snrpb	5.69E-75	########	0.845	0.657	9.52E-71	7
Elk3	3.25E-97	########	0.455	0.262	5.43E-93	7
Cox7b	2.14E-83	########	0.875	0.763	3.59E-79	7
Tnfaip8l2	6.11E-65	########	0.57	0.411	1.02E-60	7
Arpc1b	1.68E-106	########	0.961	0.958	2.81E-102	7
Arpc3	8.01E-117	########	0.958	0.954	1.34E-112	7
Wdfy4	3.14E-95	########	0.348	0.168	5.25E-91	7
Ubap2l	4.31E-52	########	0.702	0.624	7.21E-48	7
Lmbrd1	1.50E-70	########	0.458	0.318	2.52E-66	7
Spg21	2.01E-76	########	0.58	0.467	3.36E-72	7
Gm17056	1.58E-63	########	0.263	0.134	2.65E-59	7
Ccdc115	4.54E-71	########	0.447	0.319	7.60E-67	7
Ptbp3	3.18E-73	########	0.895	0.847	5.31E-69	7
Psme1	1.56E-93	########	0.849	0.767	2.61E-89	7
Uqcrh	6.83E-94	########	0.95	0.918	1.14E-89	7
Plekhm2	1.15E-99	########	0.32	0.152	1.93E-95	7
Wasf2	3.46E-67	########	0.755	0.692	5.79E-63	7
Pon2	1.05E-67	########	0.524	0.409	1.76E-63	7
Mospd2	3.37E-84	########	0.379	0.229	5.63E-80	7
Myo1e	1.59E-40	########	0.236	0.143	2.65E-36	7
Ist1	2.41E-73	########	0.539	0.415	4.03E-69	7
Srsf2	6.51E-53	########	0.816	0.691	1.09E-48	7
Ifngr2	2.58E-78	########	0.587	0.449	4.32E-74	7
Otulin	3.24E-61	########	0.527	0.414	5.41E-57	7
Piezo1	5.37E-90	########	0.379	0.211	8.99E-86	7
Tnfsf12	1.82E-97	########	0.35	0.173	3.05E-93	7
Vdac2	3.03E-71	########	0.81	0.721	5.07E-67	7
Camkk2	1.32E-48	########	0.379	0.284	2.20E-44	7
Pabpc1	1.60E-64	########	0.948	0.907	2.68E-60	7
Cuta	6.86E-74	########	0.647	0.51	1.15E-69	7
Rps14	1.49E-98	########	0.993	0.977	2.50E-94	7
Ndufa2	3.16E-90	########	0.9	0.824	5.29E-86	7
Usp8	2.97E-59	########	0.486	0.385	4.98E-55	7
Casp1	5.13E-86	########	0.379	0.214	8.59E-82	7
Rp2	3.50E-77	########	0.423	0.277	5.86E-73	7

Fosl2	4.89E-68	########	0.64	0.497	8.18E-64	7
Pla2g15	1.95E-115	########	0.271	0.1	3.26E-111	7
Cd53	6.88E-140	########	0.907	0.665	1.15E-135	7
Lamtor4	7.93E-57	########	0.82	0.72	1.33E-52	7
Arf2	7.21E-103	########	0.367	0.188	1.21E-98	7
Tmem123	3.26E-38	########	0.506	0.426	5.46E-34	7
Hif1a	5.43E-40	########	0.713	0.581	9.09E-36	7
Ndufb10	3.14E-78	########	0.821	0.687	5.25E-74	7
Cpt1a	9.38E-81	########	0.423	0.271	1.57E-76	7
Hnrnph1	4.68E-47	########	0.675	0.557	7.83E-43	7
Hnrnpk	2.42E-68	########	0.91	0.838	4.05E-64	7
Oaz1	1.15E-125	########	0.977	0.963	1.93E-121	7
Rala	1.32E-32	########	0.665	0.603	2.21E-28	7
Itsn1	3.84E-49	########	0.254	0.147	6.42E-45	7
Arhgap26	4.73E-83	########	0.339	0.172	7.91E-79	7
Pdcd6ip	8.37E-51	########	0.666	0.599	1.40E-46	7
Bola2	5.63E-76	########	0.766	0.592	9.41E-72	7
Psma1	1.55E-67	########	0.746	0.646	2.59E-63	7
Cited2	1.44E-28	########	0.627	0.559	2.40E-24	7
Pirb	2.42E-121	########	0.79	0.435	4.06E-117	7
Ltc4s	6.42E-61	########	0.107	0.024	1.07E-56	7
AI839979	1.44E-67	########	0.286	0.151	2.41E-63	7
Socs3	2.24E-53	########	0.627	0.478	3.75E-49	7
Mif4gd	1.39E-62	########	0.361	0.232	2.32E-58	7
Hgsnat	8.37E-36	########	0.381	0.296	1.40E-31	7
Mrpl30	1.01E-68	########	0.745	0.616	1.69E-64	7
Myof	3.19E-87	########	0.403	0.213	5.34E-83	7
Ywhag	2.83E-60	########	0.586	0.472	4.74E-56	7
Slk	1.88E-38	########	0.645	0.587	3.15E-34	7
Lrrc8d	7.06E-69	########	0.419	0.276	1.18E-64	7
Uqcr11	1.12E-96	########	0.866	0.723	1.88E-92	7
Gpr65	1.04E-74	########	0.435	0.264	1.74E-70	7
Atp5h	6.04E-84	########	0.934	0.893	1.01E-79	7
Btk	4.32E-103	########	0.44	0.234	7.22E-99	7
Ggta1	4.67E-84	########	0.344	0.19	7.81E-80	7
Psmg4	7.71E-82	########	0.549	0.39	1.29E-77	7
Sirt2	1.98E-69	########	0.554	0.437	3.31E-65	7
Dnajc7	3.53E-16	########	0.545	0.521	5.90E-12	7
Sde2	6.05E-68	########	0.622	0.511	1.01E-63	7
Rassf1	3.20E-67	########	0.51	0.369	5.36E-63	7
AI504432	1.34E-119	########	0.22	0.054	2.24E-115	7
Glg1	5.32E-59	########	0.589	0.484	8.91E-55	7
Dr1	2.40E-51	########	0.395	0.297	4.01E-47	7
Dnajc15	6.81E-108	########	0.638	0.464	1.14E-103	7
Tor3a	8.79E-81	########	0.289	0.14	1.47E-76	7
Cyp4f16	2.38E-122	########	0.272	0.096	3.99E-118	7
Nt5c	2.66E-81	########	0.664	0.502	4.46E-77	7
Tet2	6.19E-86	########	0.505	0.336	1.04E-81	7
Sh3bp1	8.77E-81	########	0.409	0.249	1.47E-76	7
Adss	6.97E-44	########	0.564	0.486	1.17E-39	7
Naga	9.03E-74	########	0.317	0.176	1.51E-69	7
Capns1	5.46E-60	########	0.804	0.711	9.14E-56	7

Uqcrfs1	6.66E-62	########	0.687	0.572	1.11E-57	7
Cib1	1.19E-46	########	0.608	0.552	2.00E-42	7
Milr1	6.78E-77	########	0.497	0.321	1.13E-72	7
Cuedc2	1.13E-67	########	0.623	0.501	1.89E-63	7
Ccl24	1.78E-105	########	0.105	0.004	2.97E-101	7
Adam10	1.60E-52	########	0.573	0.489	2.68E-48	7
Gnaq	2.17E-36	########	0.467	0.401	3.63E-32	7
Tmem173	2.53E-82	########	0.419	0.247	4.23E-78	7
Sik1	1.11E-63	########	0.431	0.29	1.85E-59	7
Esd	1.14E-55	########	0.819	0.764	1.91E-51	7
Luc7l3	3.45E-54	########	0.665	0.55	5.77E-50	7
G3bp1	1.07E-45	########	0.657	0.513	1.80E-41	7
Cnpy3	3.89E-68	########	0.434	0.309	6.52E-64	7
Lacc1	1.70E-149	########	0.24	0.054	2.85E-145	7
Alcam	1.24E-122	########	0.396	0.171	2.07E-118	7
Ppp1r21	4.52E-68	########	0.345	0.21	7.57E-64	7
Tet3	3.56E-49	########	0.48	0.377	5.96E-45	7
Fxyd5	1.79E-60	########	0.888	0.892	2.99E-56	7
Abca9	6.92E-121	########	0.205	0.048	1.16E-116	7
Fh1	7.17E-42	########	0.423	0.322	1.20E-37	7
Tmem176a	7.17E-18	########	0.313	0.239	1.20E-13	7
Baiap2	4.26E-41	########	0.327	0.226	7.12E-37	7
Srsf6	2.38E-49	########	0.652	0.53	3.98E-45	7
Sestd1	1.10E-90	########	0.281	0.129	1.84E-86	7
Mxi1	1.08E-28	########	0.493	0.415	1.81E-24	7
Baz1a	9.29E-60	########	0.705	0.576	1.55E-55	7
Slc15a4	1.75E-81	########	0.297	0.151	2.93E-77	7
Exoc5	2.74E-36	########	0.477	0.407	4.59E-32	7
Btla	8.04E-43	########	0.119	0.042	1.34E-38	7
Ddhd1	2.02E-105	########	0.269	0.104	3.38E-101	7
Cybb	3.39E-207	########	0.783	0.342	5.67E-203	7
Abcc3	7.56E-132	########	0.195	0.037	1.26E-127	7
Psmb6	1.35E-71	########	0.796	0.644	2.26E-67	7
Vcp	1.19E-53	########	0.794	0.67	2.00E-49	7
Sec11c	9.71E-49	########	0.787	0.717	1.62E-44	7
Slc39a1	2.85E-75	########	0.511	0.333	4.77E-71	7
Tfe3	6.76E-70	########	0.461	0.326	1.13E-65	7
Aldh9a1	7.58E-66	########	0.355	0.228	1.27E-61	7
Rb1	1.97E-60	########	0.391	0.261	3.30E-56	7
Ndufb3	5.58E-63	########	0.771	0.668	9.33E-59	7
Ddx54	1.16E-53	########	0.467	0.353	1.94E-49	7
Ophn1	1.33E-81	########	0.221	0.087	2.23E-77	7
Gpr141	1.65E-20	########	0.292	0.231	2.76E-16	7
Lyl1	1.10E-79	########	0.29	0.141	1.83E-75	7
Phf11b	2.99E-91	########	0.233	0.077	5.00E-87	7
Tmem243	6.65E-44	########	0.448	0.335	1.11E-39	7
Rin2	1.30E-66	########	0.332	0.191	2.17E-62	7
Itgb7	3.23E-87	########	0.385	0.183	5.40E-83	7
Anapc13	7.38E-50	########	0.664	0.573	1.23E-45	7
Rapgef2	4.84E-38	########	0.354	0.262	8.10E-34	7
Bri3bp	4.36E-35	########	0.322	0.242	7.30E-31	7
Nfil3	1.35E-55	########	0.47	0.345	2.27E-51	7

Gm10076	5.14E-82	########	0.952	0.835	8.61E-78	7
Pik3r5	1.83E-92	########	0.414	0.219	3.06E-88	7
Colgalt1	4.66E-62	########	0.505	0.34	7.80E-58	7
Vma21	8.50E-57	########	0.512	0.391	1.42E-52	7
Phf23	1.15E-50	########	0.441	0.339	1.93E-46	7
Guca1a	3.07E-117	########	0.279	0.105	5.13E-113	7
Tmsb10	1.70E-54	########	0.956	0.82	2.85E-50	7
Rpl37a	6.52E-115	########	0.996	0.973	1.09E-110	7
Pfdn5	3.25E-85	########	0.945	0.934	5.43E-81	7
Timm10b	1.82E-53	########	0.764	0.712	3.04E-49	7
Myeov2	4.61E-55	########	0.797	0.741	7.72E-51	7
Serinc1	5.86E-54	########	0.625	0.505	9.81E-50	7
Mrpl23	1.24E-61	########	0.656	0.502	2.08E-57	7
Fxr1	1.40E-52	########	0.615	0.515	2.34E-48	7
Elmo2	4.22E-74	########	0.32	0.18	7.07E-70	7
Eid1	6.83E-41	########	0.68	0.597	1.14E-36	7
Gba	5.66E-68	########	0.373	0.241	9.47E-64	7
Cd44	6.43E-97	########	0.852	0.735	1.08E-92	7
Plod1	7.30E-60	########	0.377	0.243	1.22E-55	7
Crlf2	4.64E-70	########	0.664	0.545	7.76E-66	7
Nampt	9.14E-58	########	0.522	0.405	1.53E-53	7
Commd8	8.32E-56	########	0.552	0.463	1.39E-51	7
Bnip2	7.88E-56	########	0.71	0.608	1.32E-51	7
Osbpl11	2.44E-46	########	0.346	0.242	4.08E-42	7
Aftph	2.11E-53	########	0.447	0.324	3.53E-49	7
Atp13a3	6.83E-52	########	0.51	0.402	1.14E-47	7
Lgals8	2.66E-50	########	0.409	0.302	4.45E-46	7
Rps5	1.34E-53	########	0.989	0.896	2.24E-49	7
Atp5j2	1.69E-65	########	0.899	0.843	2.84E-61	7
Rpl22	6.31E-62	########	0.984	0.868	1.05E-57	7
Cd40	2.92E-154	########	0.178	0.017	4.88E-150	7
Rpl28	3.61E-53	########	0.986	0.945	6.03E-49	7
Pcf11	5.28E-55	########	0.578	0.468	8.84E-51	7
Rpn2	2.92E-64	########	0.669	0.482	4.89E-60	7
Tap1	6.81E-56	########	0.456	0.325	1.14E-51	7
Stx4a	1.44E-50	########	0.481	0.379	2.40E-46	7
Rreb1	6.55E-57	########	0.352	0.23	1.10E-52	7
Lamtor2	2.30E-79	########	0.814	0.703	3.84E-75	7
Nfkbid	1.59E-137	########	0.408	0.172	2.66E-133	7
Psma5	1.69E-63	########	0.716	0.56	2.82E-59	7
Mknk2	3.34E-66	########	0.614	0.499	5.58E-62	7
St13	3.25E-51	########	0.688	0.548	5.44E-47	7
Psmb9	6.76E-50	########	0.642	0.516	1.13E-45	7
Rara	1.15E-97	########	0.543	0.336	1.92E-93	7
Rpl19	3.45E-56	########	0.989	0.942	5.77E-52	7
Eif3m	1.13E-61	########	0.797	0.643	1.90E-57	7
Slc27a1	1.36E-107	########	0.181	0.04	2.27E-103	7
Tspan31	8.59E-82	########	0.451	0.283	1.44E-77	7
Csde1	1.44E-51	########	0.812	0.729	2.41E-47	7
Ccdc86	8.57E-47	########	0.339	0.238	1.43E-42	7
Specc1	2.46E-55	########	0.314	0.198	4.12E-51	7
Cat	1.84E-56	########	0.361	0.241	3.07E-52	7

Ppp1r9b	1.04E-60	########	0.432	0.3	1.74E-56	7
Coro1a	1.12E-35	########	0.934	0.719	1.87E-31	7
Sdhc	4.84E-52	########	0.515	0.402	8.10E-48	7
Tmbim1	4.98E-61	########	0.366	0.229	8.33E-57	7
Rab11a	3.24E-42	########	0.7	0.646	5.42E-38	7
Rbm25	1.72E-50	########	0.827	0.73	2.88E-46	7
Pcyt1a	1.87E-28	########	0.392	0.334	3.13E-24	7
Sh2b3	1.03E-73	########	0.396	0.242	1.72E-69	7
Atp6v0a1	9.91E-26	########	0.336	0.269	1.66E-21	7
Cenpb	5.00E-46	########	0.529	0.411	8.36E-42	7
Sulf2	7.74E-73	########	0.279	0.133	1.29E-68	7
Xbp1	2.29E-39	########	0.612	0.513	3.83E-35	7
Nufip2	6.03E-40	########	0.682	0.601	1.01E-35	7
Hps3	9.13E-56	########	0.345	0.217	1.53E-51	7
Fcho2	2.29E-46	########	0.539	0.445	3.83E-42	7
Prdm1	1.04E-95	########	0.177	0.042	1.74E-91	7
Mkrn1	5.43E-55	########	0.705	0.628	9.09E-51	7
Szrd1	3.60E-48	########	0.474	0.382	6.02E-44	7
Plcb1	1.39E-72	########	0.225	0.096	2.33E-68	7
Slc25a28	1.49E-46	########	0.38	0.287	2.50E-42	7
Plxnd1	2.32E-86	########	0.334	0.168	3.88E-82	7
Rpl34	2.99E-74	########	0.988	0.971	5.00E-70	7
Leprot	5.96E-56	########	0.473	0.356	9.97E-52	7
Chka	1.04E-34	########	0.385	0.302	1.74E-30	7
Stard3	2.78E-62	########	0.375	0.251	4.66E-58	7
Eif1a	1.75E-45	########	0.549	0.41	2.93E-41	7
Asap1	6.28E-34	########	0.587	0.531	1.05E-29	7
Uhrf2	6.98E-31	########	0.454	0.386	1.17E-26	7
Entpd7	8.34E-52	########	0.301	0.192	1.40E-47	7
Flcn	1.01E-97	########	0.266	0.105	1.69E-93	7
Nme1	3.98E-101	########	0.826	0.541	6.66E-97	7
Chmp4b	2.89E-66	########	0.826	0.804	4.83E-62	7
Nfkb2	5.35E-65	########	0.343	0.207	8.96E-61	7
2700060E0	1.27E-61	########	0.778	0.611	2.13E-57	7
Swap70	5.48E-36	########	0.315	0.219	9.17E-32	7
Ccdc71l	7.08E-47	########	0.362	0.253	1.18E-42	7
Ndufa1	1.82E-54	########	0.826	0.767	3.04E-50	7
Morf4l1	2.75E-64	########	0.918	0.883	4.60E-60	7
St6galnac4	3.85E-49	########	0.291	0.185	6.44E-45	7
Ccl5	0	6.558856	0.926	0.025	0	8
Gzma	0	6.206805	0.69	0.004	0	8
AW112010	2.46E-192	4.345162	0.942	0.292	4.12E-188	8
Nkg7	0	4.304313	0.926	0.105	0	8
Klrk1	0	3.453515	0.839	0.023	0	8
Klre1	0	3.445802	0.773	0.004	0	8
Klrd1	0	3.102769	0.868	0.063	0	8
Klrb1c	0	3.049352	0.789	0.003	0	8
Ms4a4b	5.90E-257	2.902233	0.938	0.159	9.88E-253	8
Ncr1	0	2.893824	0.57	0.002	0	8
Klra4	0	2.795419	0.355	0.002	0	8
Klra8	0	2.708973	0.401	0.001	0	8
Gzmb	0	2.658188	0.612	0.003	0	8

H2-Q7	2.24E-226	2.514869	0.975	0.218	3.75E-222	8
Gimap4	7.39E-256	2.458022	0.826	0.119	1.24E-251	8
Klra9	0	2.416915	0.376	0.001	0	8
Klrc2	0	2.3971	0.777	0.003	0	8
Ptprcap	5.49E-215	2.329771	0.897	0.174	9.18E-211	8
Ctsw	0	2.3244	0.81	0.036	0	8
Id2	1.65E-110	2.304767	0.905	0.462	2.77E-106	8
Ccnd2	2.41E-151	2.284076	0.855	0.244	4.04E-147	8
Txk	0	2.197034	0.831	0.088	0	8
Il2rb	0	2.182092	0.818	0.016	0	8
Klra7	0	2.164575	0.43	0.002	0	8
Gimap3	4.25E-237	2.015691	0.785	0.104	7.10E-233	8
Serpinb6b	0	1.985258	0.649	0.024	0	8
Bcl2	1.41E-161	1.973003	0.793	0.175	2.37E-157	8
Serpinb9	5.04E-302	1.961958	0.653	0.056	8.44E-298	8
Hcst	1.58E-105	1.912771	0.93	0.546	2.65E-101	8
Prf1	0	1.868915	0.574	0.01	0	8
1-Sep	2.62E-142	1.853045	0.864	0.268	4.38E-138	8
Gimap1	4.61E-198	1.769856	0.864	0.157	7.71E-194	8
Lck	3.69E-304	1.743611	0.798	0.082	6.18E-300	8
H2-K1	8.74E-117	1.728621	0.992	0.9	1.46E-112	8
Klri2	0	1.725902	0.471	0.003	0	8
Cd2	2.20E-247	1.670963	0.798	0.102	3.68E-243	8
Gimap6	1.27E-181	1.652303	0.835	0.157	2.12E-177	8
Pdcd4	3.70E-83	1.625996	0.86	0.454	6.19E-79	8
Klrc1	0	1.607052	0.413	0.002	0	8
Stat4	4.32E-147	1.583614	0.727	0.165	7.24E-143	8
Skap1	2.48E-275	1.58256	0.764	0.08	4.15E-271	8
Itga4	1.36E-49	1.562379	0.748	0.471	2.27E-45	8
Epsti1	1.83E-66	1.549474	0.785	0.424	3.06E-62	8
Klrb1a	0	1.540135	0.442	0.002	0	8
Ets1	1.30E-88	1.533684	0.868	0.375	2.17E-84	8
Ctla2a	2.02E-206	1.531128	0.744	0.105	3.39E-202	8
Ugcg	1.62E-74	1.458035	0.818	0.472	2.72E-70	8
Jak1	8.74E-85	1.457928	0.959	0.82	1.46E-80	8
Gimap9	1.32E-151	1.433106	0.723	0.148	2.20E-147	8
Il18r1	3.36E-165	1.402564	0.558	0.077	5.62E-161	8
Eomes	0	1.39228	0.463	0.006	0	8
Klra3	1.39E-195	1.386208	0.19	0.004	2.32E-191	8
Klrb1f	2.61E-237	1.377133	0.496	0.039	4.37E-233	8
Ptpn22	5.23E-95	1.368695	0.702	0.233	8.76E-91	8
Spn	4.65E-71	1.350466	0.612	0.208	7.77E-67	8
Xcl1	0	1.349212	0.355	0.004	0	8
Vps37b	6.80E-23	1.339978	0.541	0.346	1.14E-18	8
Irf8	6.13E-31	1.334274	0.591	0.33	1.03E-26	8
Fyn	1.74E-93	1.328098	0.731	0.256	2.90E-89	8
Tmsb10	1.49E-73	1.323295	0.992	0.88	2.49E-69	8
Ptprc	1.12E-73	1.315269	0.983	0.798	1.87E-69	8
Cd3g	1.75E-37	1.308697	0.314	0.082	2.92E-33	8
Il18rap	3.75E-90	1.293773	0.707	0.225	6.28E-86	8
Sytl3	0	1.284454	0.533	0.014	0	8
H2-Q6	1.99E-242	1.27168	0.653	0.068	3.32E-238	8

Ifngr1	4.35E-55	1.266465	0.909	0.727	7.27E-51	8
Il2rg	6.08E-69	1.264223	0.756	0.349	1.02E-64	8
Mbnl1	1.79E-66	1.26168	0.934	0.799	3.00E-62	8
Gimap5	1.64E-156	1.254337	0.587	0.086	2.75E-152	8
Gimap8	3.55E-183	1.236087	0.595	0.075	5.94E-179	8
Gm19585	0	1.226544	0.525	0.023	0	8
Arap2	8.63E-106	1.22258	0.591	0.134	1.44E-101	8
Pla2g16	2.38E-94	1.221382	0.665	0.201	3.99E-90	8
Arsb	6.16E-82	1.217528	0.545	0.154	1.03E-77	8
Sh2d2a	0	1.215816	0.541	0.023	0	8
Pik3r1	4.20E-55	1.214597	0.773	0.494	7.03E-51	8
Samd3	0	1.208562	0.479	0.005	0	8
Cd3d	6.10E-30	1.183883	0.322	0.101	1.02E-25	8
Prkcq	4.11E-214	1.183742	0.599	0.062	6.88E-210	8
Cma1	0	1.182081	0.397	0.005	0	8
Gimap7	2.39E-173	1.178478	0.463	0.045	4.00E-169	8
Cst7	1.00E-160	1.162329	0.558	0.077	1.68E-156	8
Camk2n1	5.85E-94	1.154546	0.409	0.07	9.78E-90	8
Rinl	1.13E-62	1.152812	0.674	0.301	1.89E-58	8
Cd48	3.82E-49	1.136521	0.752	0.427	6.38E-45	8
PISD	5.42E-32	1.128677	0.607	0.364	9.06E-28	8
Shisa5	3.23E-49	1.121953	0.872	0.746	5.41E-45	8
Atp1b1	1.13E-115	1.116315	0.504	0.084	1.89E-111	8
Sh2d1a	1.73E-233	1.113489	0.521	0.042	2.89E-229	8
Runx3	3.89E-57	1.113293	0.661	0.283	6.51E-53	8
Anxa6	1.52E-57	1.108365	0.731	0.404	2.55E-53	8
Dok2	2.70E-58	1.09772	0.632	0.26	4.52E-54	8
Ikzf3	4.79E-203	1.097495	0.574	0.061	8.02E-199	8
Cd7	4.93E-170	1.094673	0.471	0.047	8.25E-166	8
Chd3	2.17E-53	1.082214	0.636	0.28	3.63E-49	8
Zcchc11	9.65E-39	1.078586	0.702	0.458	1.62E-34	8
Tnfrsf18	3.68E-80	1.078351	0.459	0.1	6.15E-76	8
Osbpl3	8.48E-124	1.076554	0.475	0.074	1.42E-119	8
Tbx21	0	1.073402	0.434	0.003	0	8
Chsy1	1.54E-47	1.071818	0.541	0.229	2.57E-43	8
Lfng	5.41E-54	1.067615	0.674	0.321	9.06E-50	8
Prex1	1.06E-37	1.065176	0.665	0.405	1.77E-33	8
Itgal	7.54E-51	1.064523	0.727	0.372	1.26E-46	8
Dock2	2.82E-51	1.059644	0.847	0.608	4.72E-47	8
Hsd11b1	6.01E-50	1.051674	0.657	0.303	1.01E-45	8
Ywhaq	1.36E-43	1.043341	0.831	0.63	2.27E-39	8
6-Sep	3.35E-54	1.035983	0.57	0.225	5.60E-50	8
S100a10	1.37E-50	1.025933	0.971	0.705	2.29E-46	8
S1pr5	1.18E-211	1.015469	0.364	0.021	1.98E-207	8
Selplg	9.02E-57	1.007134	0.917	0.632	1.51E-52	8
Adgre5	1.10E-39	1.0012	0.822	0.59	1.84E-35	8
Ablim1	1.54E-72	1.000629	0.645	0.19	2.58E-68	8
Cd247	9.15E-165	########	0.533	0.064	1.53E-160	8
Khdc1a	0	########	0.256	0.001	0	8
Satb1	2.59E-52	########	0.612	0.235	4.33E-48	8
Sp100	1.25E-39	########	0.802	0.588	2.09E-35	8
Malat1	2.13E-61	########	1	0.993	3.57E-57	8

Vgll4	1.01E-26	########	0.525	0.314	1.68E-22	8
Itgb1	9.13E-36	########	0.839	0.645	1.53E-31	8
Fasl	0	########	0.376	0.004	0	8
Ptpn18	1.84E-52	########	0.942	0.761	3.08E-48	8
Psmb8	6.87E-49	########	0.926	0.756	1.15E-44	8
Ikzf1	8.09E-36	########	0.698	0.46	1.35E-31	8
11-Sep	1.35E-34	########	0.657	0.413	2.25E-30	8
Rps27	1.25E-68	########	1	0.977	2.09E-64	8
Arl4c	1.21E-32	########	0.719	0.425	2.02E-28	8
Ankrd44	1.60E-35	########	0.719	0.486	2.67E-31	8
B4galnt1	1.49E-44	########	0.682	0.384	2.49E-40	8
Rpl27	1.01E-54	########	0.971	0.874	1.69E-50	8
Hmha1	9.14E-37	########	0.785	0.56	1.53E-32	8
Dnajc15	1.64E-26	########	0.686	0.54	2.75E-22	8
Clec2d	4.91E-27	0.906087	0.711	0.535	8.21E-23	8
Car2	4.36E-143	########	0.347	0.031	7.29E-139	8
H2-D1	1.68E-63	########	1	0.972	2.80E-59	8
Ctla2b	6.18E-59	########	0.426	0.107	1.03E-54	8
B2m	7.95E-61	########	0.996	0.971	1.33E-56	8
Sub1	1.00E-53	########	0.963	0.91	1.68E-49	8
Klrg1	0	########	0.248	0.001	0	8
Itgb7	8.70E-40	########	0.603	0.266	1.46E-35	8
Bin2	4.98E-38	########	0.74	0.485	8.34E-34	8
Ccnd3	1.34E-34	########	0.835	0.643	2.25E-30	8
Lgals1	3.39E-34	########	0.95	0.651	5.67E-30	8
Rsbn1l	7.68E-24	########	0.665	0.547	1.29E-19	8
Peak1	3.93E-32	########	0.529	0.27	6.58E-28	8
Gm4070	7.67E-74	########	0.442	0.1	1.28E-69	8
Styk1	7.04E-298	########	0.277	0.006	1.18E-293	8
Ifi47	8.76E-53	########	0.591	0.226	1.47E-48	8
Il7r	4.67E-13	########	0.285	0.131	7.81E-09	8
Cd27	1.35E-61	########	0.438	0.11	2.26E-57	8
Tbc1d10c	1.17E-46	########	0.537	0.206	1.95E-42	8
Ifi203	1.06E-36	########	0.678	0.351	1.78E-32	8
Gpr171	6.33E-110	########	0.426	0.061	1.06E-105	8
Prpf4b	9.77E-31	########	0.715	0.549	1.64E-26	8
Cd226	2.05E-110	########	0.31	0.032	3.43E-106	8
Arhgef1	1.59E-30	########	0.785	0.648	2.67E-26	8
Ifitm10	1.59E-125	########	0.368	0.041	2.65E-121	8
Tspan32	4.52E-23	########	0.471	0.259	7.56E-19	8
Rpl38	1.56E-58	########	0.996	0.961	2.61E-54	8
Psme1	2.62E-36	########	0.872	0.803	4.38E-32	8
AY036118	6.78E-49	########	1	0.978	1.14E-44	8
Gata3	1.84E-119	########	0.326	0.033	3.08E-115	8
1700025G(	1.30E-34	########	0.438	0.184	2.17E-30	8
Klra1	3.15E-105	########	0.103	0.002	5.26E-101	8
Il12rb2	0	########	0.351	0.006	0	8
Klhl6	4.66E-25	########	0.554	0.338	7.80E-21	8
Xrn2	1.97E-27	########	0.748	0.618	3.29E-23	8
Klf2	2.73E-21	########	0.818	0.773	4.57E-17	8
Hopx	6.09E-19	########	0.483	0.281	1.02E-14	8
Tnik	2.86E-86	########	0.38	0.062	4.79E-82	8

H2afz	5.60E-31	########	0.992	0.914	9.36E-27	8
Rpl13a	5.43E-58	########	0.996	0.962	9.09E-54	8
Esyt1	1.13E-27	########	0.64	0.45	1.88E-23	8
Rac2	6.75E-38	########	0.946	0.735	1.13E-33	8
Arl6ip5	5.10E-26	########	0.579	0.38	8.54E-22	8
Slamf7	2.87E-97	########	0.388	0.057	4.81E-93	8
Aes	4.26E-29	########	0.826	0.74	7.13E-25	8
Rpl36	2.13E-49	########	0.992	0.905	3.56E-45	8
Borcs7	1.73E-12	########	0.417	0.3	2.89E-08	8
Rabgap1l	5.06E-33	########	0.545	0.272	8.47E-29	8
H2-Q4	1.75E-45	########	0.533	0.205	2.92E-41	8
Pitpnc1	4.07E-26	########	0.562	0.331	6.81E-22	8
Lat	1.37E-65	########	0.388	0.077	2.29E-61	8
Zap70	6.15E-164	########	0.38	0.032	1.03E-159	8
Set	8.56E-33	########	0.88	0.711	1.43E-28	8
Rbm39	4.01E-45	########	0.959	0.919	6.71E-41	8
Cd82	5.19E-23	########	0.533	0.332	8.69E-19	8
1700020I14	1.68E-21	########	0.657	0.538	2.80E-17	8
Lax1	1.17E-145	########	0.372	0.035	1.96E-141	8
Atp11b	6.31E-20	########	0.682	0.564	1.06E-15	8
Tes	7.98E-25	########	0.579	0.365	1.34E-20	8
Itgb2	7.66E-26	########	0.835	0.641	1.28E-21	8
Cd160	2.64E-296	########	0.277	0.006	4.42E-292	8
Zfpm1	1.62E-76	########	0.36	0.064	2.71E-72	8
Thy1	5.96E-23	########	0.36	0.145	9.97E-19	8
Coro2a	3.23E-41	########	0.43	0.149	5.40E-37	8
Ppp1r12a	1.61E-21	########	0.702	0.6	2.70E-17	8
Hist1h1e	6.95E-27	########	0.657	0.399	1.16E-22	8
Pfn1	3.37E-46	########	0.983	0.983	5.65E-42	8
Cnot6l	2.42E-18	########	0.612	0.482	4.05E-14	8
Pde7a	6.26E-40	########	0.434	0.156	1.05E-35	8
Lpin1	6.40E-50	########	0.318	0.073	1.07E-45	8
Ahnak	1.64E-26	########	0.913	0.631	2.75E-22	8
Trim12a	5.38E-21	########	0.57	0.378	8.99E-17	8
P2ry10	1.48E-45	########	0.281	0.058	2.48E-41	8
Gem	7.20E-28	########	0.368	0.138	1.21E-23	8
Rgs1	5.54E-21	########	0.31	0.115	9.27E-17	8
Dock10	3.56E-25	########	0.657	0.439	5.95E-21	8
Serpina3g	5.32E-89	########	0.289	0.035	8.90E-85	8
Arl4d	3.72E-80	########	0.335	0.053	6.22E-76	8
Cd28	7.10E-55	########	0.322	0.064	1.19E-50	8
Itk	3.74E-66	########	0.397	0.081	6.27E-62	8
Fam189b	4.90E-42	########	0.393	0.121	8.20E-38	8
Ogt	6.08E-24	########	0.711	0.542	1.02E-19	8
Serbp1	6.99E-28	########	0.938	0.803	1.17E-23	8
Rps21	7.93E-50	########	0.996	0.974	1.33E-45	8
Jakmip1	2.69E-92	########	0.393	0.06	4.50E-88	8
Fubp1	3.39E-21	########	0.661	0.521	5.67E-17	8
Klrb1b	1.01E-104	########	0.19	0.011	1.69E-100	8
Atp1b3	2.30E-22	########	0.822	0.709	3.84E-18	8
As3mt	2.28E-20	########	0.343	0.162	3.81E-16	8
Taf15	1.76E-22	########	0.579	0.404	2.95E-18	8

Snrpg	5.91E-30	########	0.864	0.7	9.89E-26	8
Rasal3	6.43E-45	########	0.413	0.127	1.08E-40	8
Fnbp1	1.69E-18	########	0.616	0.51	2.82E-14	8
Agpat3	3.62E-26	########	0.459	0.234	6.05E-22	8
Cetn2	3.50E-15	########	0.525	0.414	5.85E-11	8
Atp2a3	3.71E-22	########	0.417	0.215	6.21E-18	8
Traf3ip3	5.76E-25	########	0.545	0.313	9.63E-21	8
Rbl2	2.94E-17	########	0.405	0.238	4.92E-13	8
Cd3e	7.75E-26	########	0.252	0.072	1.30E-21	8
Ttc14	1.86E-17	########	0.599	0.484	3.12E-13	8
Macf1	1.64E-22	########	0.764	0.677	2.74E-18	8
Ly9	1.13E-39	########	0.434	0.155	1.90E-35	8
Mndal	5.25E-27	########	0.653	0.376	8.79E-23	8
Zcchc7	4.13E-15	########	0.558	0.435	6.91E-11	8
Usp48	1.16E-16	########	0.438	0.282	1.95E-12	8
Cxcr6	1.28E-83	########	0.161	0.01	2.15E-79	8
Akna	9.49E-24	########	0.55	0.331	1.59E-19	8
Acap1	1.61E-29	########	0.45	0.203	2.69E-25	8
Apobec3	6.31E-23	########	0.624	0.423	1.06E-18	8
Leprotl1	8.91E-21	########	0.69	0.624	1.49E-16	8
Prrc2c	5.78E-19	########	0.826	0.79	9.67E-15	8
Sumo2	7.79E-31	########	0.909	0.78	1.30E-26	8
Cxcr3	1.33E-123	########	0.273	0.021	2.22E-119	8
Mxd4	3.95E-12	########	0.533	0.433	6.60E-08	8
Prrc2b	5.43E-19	########	0.483	0.309	9.09E-15	8
Ikzf2	2.82E-18	########	0.165	0.046	4.72E-14	8
Rps24	3.59E-50	########	0.996	0.974	6.01E-46	8
Tecpr1	1.02E-33	########	0.438	0.175	1.71E-29	8
Itga2	2.63E-144	########	0.289	0.02	4.40E-140	8
Gpr65	3.66E-20	########	0.529	0.337	6.12E-16	8
Lrrk1	4.63E-22	########	0.376	0.179	7.74E-18	8
Prkacb	4.20E-16	########	0.529	0.389	7.04E-12	8
Xrn1	3.13E-15	########	0.475	0.339	5.23E-11	8
Sike1	1.05E-10	########	0.442	0.347	1.76E-06	8
D16Ertd47.	6.28E-29	########	0.38	0.154	1.05E-24	8
Arhgef18	1.78E-22	########	0.401	0.194	2.98E-18	8
Hnrnpa3	3.22E-25	########	0.909	0.834	5.39E-21	8
Fryl	3.89E-18	########	0.492	0.328	6.51E-14	8
Dusp11	1.05E-16	########	0.711	0.679	1.76E-12	8
Evl	5.77E-17	########	0.508	0.341	9.65E-13	8
Spata13	2.31E-26	########	0.45	0.208	3.87E-22	8
Ptpn4	1.32E-58	########	0.322	0.065	2.22E-54	8
Srrm2	2.08E-29	########	0.897	0.842	3.47E-25	8
Psmb10	1.80E-19	########	0.711	0.587	3.01E-15	8
Znrf2	1.03E-12	########	0.492	0.379	1.73E-08	8
Pogk	4.22E-44	########	0.293	0.07	7.07E-40	8
Dusp2	4.95E-19	########	0.583	0.334	8.29E-15	8
Tespa1	9.62E-110	########	0.36	0.043	1.61E-105	8
Mllt3	6.09E-33	########	0.343	0.114	1.02E-28	8
Tmsb4x	2.19E-36	########	1	0.997	3.66E-32	8
Sp110	3.34E-20	########	0.574	0.406	5.58E-16	8
Sytl2	1.79E-121	########	0.289	0.025	2.99E-117	8

Top2b	3.46E-11	########	0.599	0.575	5.80E-07	8
Tra2b	4.93E-17	########	0.715	0.65	8.25E-13	8
Uba52	3.29E-31	########	0.942	0.835	5.51E-27	8
Fam53b	4.65E-18	########	0.409	0.235	7.78E-14	8
Tma7	2.00E-30	########	0.938	0.876	3.34E-26	8
Dnaja1	1.73E-17	########	0.913	0.848	2.89E-13	8
Cd52	2.28E-23	########	0.988	0.83	3.81E-19	8
Arhgap15	2.26E-15	########	0.645	0.494	3.79E-11	8
Nup210	1.46E-32	########	0.384	0.141	2.45E-28	8
Dnajc1	1.43E-10	########	0.471	0.373	2.39E-06	8
Gramd3	4.18E-13	########	0.285	0.141	6.99E-09	8
Fgl2	1.55E-12	########	0.5	0.349	2.60E-08	8
Spry2	2.27E-18	########	0.289	0.125	3.80E-14	8
Eif4a2	1.70E-21	########	0.727	0.605	2.84E-17	8
S1pr4	1.02E-21	########	0.43	0.219	1.71E-17	8
Fkbp3	2.33E-12	########	0.579	0.48	3.91E-08	8
Rtf1	6.12E-19	########	0.653	0.551	1.02E-14	8
Cyfip2	5.16E-20	########	0.55	0.316	8.63E-16	8
Cnn2	6.80E-18	########	0.769	0.645	1.14E-13	8
Pole4	1.26E-11	########	0.496	0.407	2.12E-07	8
Adora2a	4.58E-76	########	0.26	0.033	7.67E-72	8
Sms	1.43E-19	########	0.504	0.317	2.40E-15	8
Dad1	3.78E-21	########	0.864	0.735	6.33E-17	8
Stk10	4.66E-18	########	0.558	0.377	7.79E-14	8
Whsc1l1	3.77E-15	########	0.748	0.722	6.31E-11	8
Rnaset2b	1.66E-11	########	0.384	0.263	2.78E-07	8
Nsmaf	2.29E-19	########	0.326	0.149	3.83E-15	8
Otulin	7.86E-14	########	0.558	0.463	1.32E-09	8
Zfp36l2	7.39E-16	########	0.86	0.818	1.24E-11	8
Cox17	8.98E-18	########	0.731	0.678	1.50E-13	8
Cd47	5.66E-25	########	0.942	0.898	9.47E-21	8
Gbp4	2.97E-85	########	0.343	0.05	4.97E-81	8
Rpl39	8.70E-36	########	0.996	0.959	1.46E-31	8
Tmem37	5.30E-21	########	0.355	0.152	8.86E-17	8
Cd244	1.78E-21	########	0.405	0.187	2.99E-17	8
Nktr	5.32E-08	########	0.603	0.611	########	8
Aqr	3.63E-09	########	0.417	0.332	6.08E-05	8
Arpc5l	6.25E-19	########	0.624	0.51	1.05E-14	8
Nop10	8.01E-17	########	0.719	0.593	1.34E-12	8
Arglu1	3.96E-18	########	0.686	0.616	6.62E-14	8
Abracl	4.30E-19	########	0.876	0.809	7.19E-15	8
Krit1	4.61E-08	########	0.5	0.446	########	8
Ptp4a3	1.39E-23	########	0.479	0.25	2.33E-19	8
Fam204a	1.11E-08	########	0.426	0.351	########	8
Padi2	7.29E-52	########	0.322	0.07	1.22E-47	8
Pfkp	1.76E-12	########	0.496	0.388	2.94E-08	8
Kmt2a	2.22E-10	########	0.521	0.445	3.71E-06	8
Gbp7	1.04E-19	########	0.463	0.26	1.74E-15	8
Ptpn7	1.08E-13	########	0.364	0.213	1.81E-09	8
Mir142hg	9.33E-17	########	0.347	0.171	1.56E-12	8
4930453N2	8.11E-12	########	0.43	0.316	1.36E-07	8
Atrx	1.46E-13	########	0.736	0.7	2.45E-09	8

S100a13	4.75E-17	########	0.843	0.77	7.95E-13	8
Txnip	1.72E-23	########	0.81	0.643	2.88E-19	8
mt-Nd2	6.77E-34	########	1	0.889	1.13E-29	8
Gabpb2	7.07E-09	########	0.417	0.332	########	8
Tapbpl	1.77E-17	########	0.331	0.161	2.96E-13	8
Gnas	5.34E-27	########	0.959	0.881	8.93E-23	8
Aak1	5.04E-15	########	0.397	0.241	8.43E-11	8
Zbtb1	2.96E-06	########	0.376	0.308	########	8
Rhof	7.98E-35	########	0.318	0.093	1.34E-30	8
Rps15a	7.25E-29	########	1	0.952	1.21E-24	8
Nabp1	1.87E-14	########	0.545	0.39	3.13E-10	8
Rpl21	7.73E-33	########	0.996	0.948	1.29E-28	8
Gabarapl2	5.01E-10	########	0.674	0.708	8.39E-06	8
Tpst2	2.14E-12	########	0.554	0.465	3.58E-08	8
Gnptg	6.86E-13	########	0.36	0.222	1.15E-08	8
Pnisr	2.48E-12	########	0.607	0.545	4.15E-08	8
Kcnab2	1.49E-17	########	0.372	0.201	2.49E-13	8
Prkch	1.21E-17	########	0.339	0.165	2.02E-13	8
Sell	1.28E-11	########	0.632	0.477	2.14E-07	8
Psip1	2.58E-13	########	0.488	0.343	4.31E-09	8
Myl6	3.84E-32	########	0.996	0.977	6.43E-28	8
Ankrd11	8.73E-11	########	0.764	0.771	1.46E-06	8
Rps23	4.18E-35	########	1	0.976	6.99E-31	8
Nck1	1.74E-13	########	0.537	0.425	2.91E-09	8
Hypk	6.23E-09	########	0.521	0.477	########	8
Psme2	1.16E-10	########	0.711	0.737	1.94E-06	8
Stk24	5.22E-12	########	0.574	0.518	8.74E-08	8
Plekha5	6.09E-35	########	0.293	0.083	1.02E-30	8
Rpl5	7.11E-31	########	0.988	0.904	1.19E-26	8
Krtcap2	1.30E-15	########	0.793	0.667	2.18E-11	8
Kcnj8	1.23E-56	########	0.186	0.022	2.06E-52	8
Hn1	8.59E-20	########	0.798	0.713	1.44E-15	8
Nt5c	2.78E-10	########	0.62	0.575	4.66E-06	8
Rpl34	3.44E-35	########	0.992	0.978	5.75E-31	8
Dennd1b	1.34E-13	########	0.463	0.324	2.24E-09	8
Prkca	9.75E-28	########	0.355	0.134	1.63E-23	8
Gm4955	8.38E-23	########	0.273	0.09	1.40E-18	8
Rhoh	1.47E-24	########	0.475	0.224	2.45E-20	8
Bptf	7.19E-12	########	0.657	0.641	1.20E-07	8
Snrpf	1.16E-22	########	0.884	0.704	1.94E-18	8
Cd69	1.82E-08	########	0.285	0.16	########	8
Pear1	2.69E-55	########	0.273	0.049	4.51E-51	8
Rpl15	9.15E-23	########	0.95	0.826	1.53E-18	8
Gse1	2.61E-17	########	0.347	0.176	4.37E-13	8
Pkp3	8.06E-21	########	0.302	0.117	1.35E-16	8
Rpl23a	1.16E-23	########	0.95	0.91	1.94E-19	8
Zfp512	1.20E-20	########	0.289	0.115	2.00E-16	8
Birc2	2.12E-07	########	0.368	0.274	########	8
Nmi	1.30E-10	########	0.45	0.346	2.17E-06	8
Kbtbd11	1.77E-27	########	0.273	0.081	2.97E-23	8
Rplp2	3.88E-35	########	0.996	0.971	6.48E-31	8
Kansl1	1.97E-07	########	0.517	0.475	########	8

H2-T22	2.64E-12	########	0.512	0.396	4.41E-08	8
Pde3b	3.13E-12	########	0.322	0.18	5.24E-08	8
Gm11713	2.68E-20	########	0.289	0.116	4.49E-16	8
Tsc22d4	3.83E-13	########	0.798	0.758	6.40E-09	8
Scimp	2.05E-44	########	0.223	0.04	3.44E-40	8
Elf1	2.95E-11	########	0.628	0.588	4.94E-07	8
Syf2	1.75E-14	########	0.74	0.703	2.92E-10	8
Rab37	3.01E-56	########	0.285	0.052	5.04E-52	8
Pnn	8.62E-12	########	0.587	0.51	1.44E-07	8
Sipa1	2.54E-07	########	0.496	0.447	########	8
Rpl27a	1.35E-29	########	1	0.978	2.26E-25	8
Ndufa4	4.76E-22	########	0.942	0.829	7.96E-18	8
St8sia4	1.19E-15	########	0.57	0.381	1.99E-11	8
Hnrnpa0	1.34E-20	########	0.86	0.803	2.24E-16	8
Gm12216	7.27E-45	########	0.285	0.063	1.22E-40	8
Cbx3	5.98E-14	########	0.669	0.565	1.00E-09	8
Rps7	3.40E-28	0.51754	0.996	0.947	5.69E-24	8
Ppp3cc	1.54E-61	########	0.289	0.049	2.57E-57	8
Cblb	1.15E-13	########	0.372	0.219	1.93E-09	8
Irf2bpl	1.60E-14	########	0.43	0.271	2.68E-10	8
Rasgrp1	3.63E-49	########	0.331	0.073	6.08E-45	8
Fam65b	1.80E-16	########	0.599	0.392	3.01E-12	8
A430078G2	1.65E-16	########	0.318	0.148	2.77E-12	8
Rnf138	1.06E-07	########	0.368	0.279	########	8
Zdhhc18	5.45E-13	########	0.434	0.293	9.11E-09	8
Rpl37a	1.73E-34	########	1	0.983	2.90E-30	8
Lbh	1.28E-10	########	0.467	0.35	2.14E-06	8
Polr2m	3.13E-07	########	0.455	0.402	########	8
Bcl11b	2.45E-18	########	0.248	0.087	4.09E-14	8
Icos	2.81E-21	########	0.103	0.018	4.70E-17	8
Sesn3	1.42E-22	########	0.368	0.159	2.38E-18	8
Pura	1.22E-07	########	0.554	0.515	########	8
Pydc3	2.99E-33	########	0.252	0.061	5.00E-29	8
Fam105a	9.17E-17	########	0.562	0.38	1.53E-12	8
Slc50a1	7.26E-09	########	0.455	0.372	########	8
Clnk	0	########	0.244	0.002	0	8
Mylip	2.56E-08	########	0.421	0.31	########	8
Tcf7	3.57E-45	########	0.372	0.09	5.98E-41	8
Gprin3	5.46E-60	########	0.207	0.026	9.14E-56	8
Gramd4	1.05E-17	########	0.298	0.131	1.76E-13	8
Lars2	6.68E-25	########	0.979	0.978	1.12E-20	8
Cmc1	4.08E-07	########	0.463	0.42	########	8
Dpm3	9.32E-10	########	0.628	0.6	1.56E-05	8
Samd9l	1.16E-11	########	0.562	0.427	1.94E-07	8
Cr1l	8.25E-10	########	0.587	0.549	1.38E-05	8
Anp32a	4.46E-16	########	0.826	0.777	7.46E-12	8
Tram1	2.58E-11	########	0.678	0.641	4.32E-07	8
Ppil2	4.11E-08	########	0.45	0.389	########	8
Ncor1	1.07E-18	########	0.831	0.768	1.79E-14	8
H2-T23	9.57E-14	########	0.781	0.682	1.60E-09	8
Gm11808	7.40E-09	########	0.579	0.546	########	8
Ndufa3	5.14E-13	########	0.793	0.812	8.59E-09	8

Socs1	7.28E-12	########	0.298	0.16	1.22E-07	8
Pik3cd	4.89E-08	########	0.5	0.423	########	8
Wipf1	7.24E-13	########	0.661	0.601	1.21E-08	8
Ctcf	2.56E-10	########	0.579	0.531	4.28E-06	8
Gpr174	1.07E-69	########	0.273	0.037	1.79E-65	8
Rpl30	3.76E-30	########	1	0.976	6.28E-26	8
Srsf3	4.11E-12	########	0.826	0.782	6.88E-08	8
Rplp1	4.05E-25	########	0.996	0.974	6.78E-21	8
Herpud1	1.07E-06	########	0.5	0.455	########	8
Sash3	5.37E-10	########	0.413	0.3	8.99E-06	8
Phf3	3.20E-07	########	0.599	0.619	########	8
Psmb9	1.09E-09	########	0.616	0.572	1.83E-05	8
Nfatc3	1.97E-07	########	0.38	0.295	########	8
Ddx21	1.38E-08	########	0.566	0.509	########	8
Grap	2.93E-32	########	0.355	0.114	4.90E-28	8
St3gal6	9.41E-34	########	0.38	0.125	1.57E-29	8
Arf6	8.39E-09	########	0.62	0.613	########	8
Lrrc8c	8.21E-12	########	0.364	0.222	1.37E-07	8
Luc7l2	5.18E-13	########	0.777	0.766	8.67E-09	8
Kpna4	7.34E-14	########	0.661	0.614	1.23E-09	8
Rnaseh2c	6.25E-11	########	0.694	0.626	1.05E-06	8
Tmem163	3.26E-90	########	0.252	0.025	5.45E-86	8
Pdcd10	2.15E-11	########	0.727	0.742	3.60E-07	8
Pde2a	1.54E-07	########	0.347	0.251	########	8
Atp5h	1.75E-18	########	0.946	0.911	2.93E-14	8
Hnrnpf	4.49E-18	########	0.946	0.889	7.52E-14	8
Gsap	2.97E-08	########	0.368	0.266	########	8
mt-Nd3	2.14E-24	########	0.971	0.843	3.58E-20	8
Sec62	1.93E-15	########	0.802	0.764	3.22E-11	8
Tmem243	1.17E-09	########	0.479	0.384	1.95E-05	8
Gm6483	3.21E-08	########	0.335	0.231	########	8
Vezf1	2.08E-06	########	0.438	0.39	########	8
Atp5e	6.92E-22	########	0.975	0.94	1.16E-17	8
Rps27rt	3.97E-08	########	0.467	0.381	########	8
Crip1	4.68E-18	########	0.955	0.715	7.83E-14	8
Npm3	3.06E-07	########	0.475	0.408	########	8
Idnk	3.46E-10	########	0.459	0.364	5.79E-06	8
Utrn	5.57E-08	########	0.421	0.321	########	8
Rps13	1.76E-26	########	1	0.974	2.94E-22	8
mt-Nd4l	3.47E-14	########	0.847	0.748	5.81E-10	8
Sh2d3c	1.98E-18	########	0.405	0.209	3.32E-14	8
Rpl19	2.19E-23	########	1	0.962	3.66E-19	8
F2r	4.00E-25	########	0.215	0.06	6.69E-21	8
Rps14	2.86E-24	########	0.992	0.984	4.78E-20	8
Rras2	6.45E-22	########	0.285	0.106	1.08E-17	8
Usp3	2.66E-07	########	0.5	0.461	########	8
Nol7	1.07E-09	########	0.702	0.664	1.79E-05	8
Cnbp	4.96E-12	########	0.773	0.708	8.30E-08	8
Rps11	4.22E-22	########	0.996	0.965	7.06E-18	8
Serinc3	2.21E-13	########	0.884	0.827	3.69E-09	8
Cd37	8.75E-12	########	0.579	0.458	1.46E-07	8
Aim1	3.42E-07	########	0.285	0.19	########	8

Csk	8.82E-12	########	0.599	0.539	1.48E-07	8
Stk4	1.60E-11	########	0.632	0.557	2.67E-07	8
Malt1	7.35E-07	########	0.376	0.28	########	8
Nudcd3	5.37E-08	########	0.45	0.389	########	8
Crebzf	1.38E-07	########	0.331	0.238	########	8
Plekhj1	7.79E-09	########	0.558	0.529	########	8
Kcnq1ot1	2.25E-06	########	0.438	0.342	########	8
Cecr5	1.26E-51	########	0.248	0.043	2.11E-47	8
Ssh1	8.11E-12	########	0.273	0.142	1.36E-07	8
Sepw1	4.26E-18	########	0.868	0.668	7.14E-14	8
Kmt2e	1.86E-06	########	0.678	0.716	########	8
Rpl12	6.48E-19	########	0.992	0.888	1.08E-14	8
Nlrc3	3.04E-45	########	0.26	0.053	5.09E-41	8
Ppp1r16b	3.89E-30	########	0.26	0.069	6.50E-26	8
Haus3	6.57E-07	########	0.269	0.176	########	8
Acot7	6.73E-08	########	0.31	0.213	########	8
Sema4a	1.30E-11	########	0.521	0.392	2.18E-07	8
Usmg5	1.22E-12	########	0.814	0.784	2.05E-08	8
Limd2	4.85E-10	########	0.711	0.66	8.11E-06	8
Tnfrsf9	6.61E-51	########	0.132	0.012	1.11E-46	8
Tln1	9.44E-12	########	0.868	0.867	1.58E-07	8
Tpm4	2.68E-12	########	0.785	0.671	4.49E-08	8
Stag1	1.63E-08	########	0.525	0.47	########	8
Rsrc2	8.23E-08	########	0.649	0.676	########	8
Apbb1ip	2.19E-10	########	0.769	0.711	3.66E-06	8
Thrap3	2.94E-09	########	0.653	0.621	4.92E-05	8
Il2ra	2.70E-56	########	0.103	0.006	4.51E-52	8
Jade2	1.29E-26	########	0.252	0.075	2.16E-22	8
N4bp2l2	2.00E-06	########	0.496	0.467	########	8
Hs3st3b1	2.14E-51	########	0.194	0.027	3.58E-47	8
Ldb1	7.17E-08	########	0.36	0.27	########	8
Tonsl	1.05E-08	########	0.669	0.689	########	8
Gm26740	3.37E-07	########	0.488	0.386	########	8
Tm6sf1	3.53E-07	########	0.62	0.561	########	8
Ict1	6.27E-07	########	0.504	0.466	########	8
Smc5	3.15E-07	########	0.331	0.239	########	8
Sla2	1.25E-77	########	0.24	0.026	2.08E-73	8
Ddx5	8.30E-16	########	0.992	0.983	1.39E-11	8
Mmgt2	7.85E-14	########	0.269	0.129	1.31E-09	8
Ube2i	1.39E-12	########	0.756	0.754	2.32E-08	8
Purb	7.55E-08	########	0.686	0.711	########	8
Fcho1	4.58E-15	########	0.198	0.073	7.66E-11	8
Rnaset2a	2.33E-06	########	0.364	0.29	########	8
Dennd1c	1.77E-10	########	0.326	0.194	2.96E-06	8
Arpp19	6.29E-11	########	0.756	0.748	1.05E-06	8
Vps13a	2.48E-06	########	0.302	0.218	########	8
Ttc39b	2.36E-07	########	0.368	0.271	########	8
Esco1	1.51E-07	########	0.409	0.323	########	8
Rnf187	3.04E-09	########	0.529	0.468	5.08E-05	8
Mdh1	1.94E-08	########	0.595	0.557	########	8
Trmt112	4.10E-10	########	0.665	0.623	6.85E-06	8
Sp4	6.21E-18	########	0.285	0.12	1.04E-13	8

Rpl9	1.77E-20	########	0.992	0.975	2.96E-16	8
Ptp4a2	5.07E-09	########	0.793	0.793	8.49E-05	8
Ppia	5.92E-20	########	0.996	0.902	9.91E-16	8
Hnrnpa2b1	9.72E-12	########	0.909	0.9	1.63E-07	8
Lat2	3.54E-07	########	0.417	0.316	########	8
Eef1d	3.14E-13	########	0.86	0.731	5.26E-09	8
Smad3	2.38E-09	########	0.244	0.135	3.98E-05	8
Adamts14	1.34E-75	########	0.194	0.018	2.25E-71	8
1810022K0	3.02E-08	########	0.595	0.549	########	8
Gbp8	4.36E-79	0.411382	0.236	0.025	7.30E-75	8
Lsm5	1.17E-06	########	0.57	0.534	########	8
Zcchc18	2.06E-101	########	0.207	0.014	3.45E-97	8
BC030336	1.32E-06	########	0.285	0.202	########	8
Saraf	6.06E-07	########	0.545	0.512	########	8
Stk26	2.14E-09	########	0.343	0.222	3.59E-05	8
Btg2	2.83E-06	########	0.715	0.728	########	8
Crim1	1.22E-12	########	0.24	0.107	2.04E-08	8
Rps29	6.68E-29	########	1	0.991	1.12E-24	8
Rps18	1.41E-14	########	1	0.911	2.36E-10	8
Cox8a	1.82E-20	########	0.979	0.954	3.04E-16	8
Tox	1.16E-49	########	0.207	0.03	1.94E-45	8
Cuedc1	2.37E-14	########	0.198	0.076	3.97E-10	8
Rps15	6.17E-19	########	0.992	0.936	1.03E-14	8
Rpl17	3.56E-19	########	0.996	0.983	5.96E-15	8
Rpl37	1.62E-22	########	0.988	0.982	2.71E-18	8
Rab19	8.72E-54	########	0.227	0.034	1.46E-49	8
Fam117a	3.71E-08	########	0.277	0.171	########	8
Tnk2	4.83E-18	########	0.227	0.083	8.09E-14	8
Celf2	8.22E-09	########	0.744	0.72	########	8
Ipcef1	5.40E-09	########	0.339	0.212	9.04E-05	8
Med30	1.68E-07	########	0.488	0.444	########	8
Ddx24	7.75E-07	########	0.624	0.617	########	8
Fam60a	3.20E-08	########	0.269	0.163	########	8
Raly	1.45E-08	########	0.661	0.642	########	8
Rps3a1	9.95E-17	########	0.996	0.968	1.66E-12	8
Sptbn1	1.03E-08	########	0.616	0.55	########	8
Emp3	1.39E-09	########	0.93	0.84	2.33E-05	8
Itgax	1.61E-29	########	0.198	0.044	2.69E-25	8
Tagln2	7.76E-10	########	0.876	0.755	1.30E-05	8
Kif21b	1.24E-07	########	0.343	0.237	########	8
Adamts10	1.09E-09	########	0.227	0.117	1.82E-05	8
Ifng	4.50E-200	########	0.136	0.001	7.53E-196	8
AU020206	1.39E-10	########	0.231	0.114	2.32E-06	8
Cers4	2.73E-16	########	0.227	0.086	4.56E-12	8
Gm37387	1.81E-50	########	0.178	0.022	3.03E-46	8
Rpl23	8.02E-16	########	1	0.984	1.34E-11	8
Dnajc8	7.93E-08	########	0.678	0.709	########	8
Rgs3	4.32E-09	########	0.339	0.22	7.22E-05	8
Eef1a1	9.92E-16	########	1	0.987	1.66E-11	8
Rpl10	1.10E-13	########	0.992	0.951	1.84E-09	8
Phf11b	7.40E-16	########	0.314	0.144	1.24E-11	8
Snrpe	9.78E-12	########	0.855	0.777	1.64E-07	8

Son	6.20E-13	########	0.921	0.933	1.04E-08	8
Calm2	1.29E-12	########	0.93	0.896	2.15E-08	8
Rps16	1.31E-18	########	1	0.989	2.20E-14	8
1300002E1	1.04E-09	########	0.244	0.13	1.75E-05	8
Pdgfb	1.69E-20	########	0.186	0.052	2.83E-16	8
Rbx1	8.86E-08	########	0.814	0.829	########	8
Lpcat4	2.53E-18	########	0.231	0.084	4.23E-14	8
Rpl22l1	4.10E-11	########	0.921	0.803	6.86E-07	8
Atp2b4	8.80E-09	########	0.31	0.192	########	8
Mfsd4	8.01E-08	########	0.302	0.193	########	8
Rabac1	3.97E-07	########	0.818	0.828	########	8
Trim26	2.44E-06	########	0.306	0.219	########	8
Rpl13	2.81E-12	########	1	0.961	4.69E-08	8
Rab6b	5.02E-15	########	0.178	0.06	8.40E-11	8
Mmd	2.81E-07	########	0.314	0.217	########	8
Sbno1	2.87E-06	########	0.612	0.63	########	8
Fam102a	1.17E-13	########	0.269	0.123	1.96E-09	8
Rpl22	1.30E-14	########	0.983	0.92	2.17E-10	8
Vopp1	3.22E-11	########	0.194	0.085	5.38E-07	8
Gm43647	1.05E-166	########	0.149	0.003	1.75E-162	8
Dapk2	6.26E-19	########	0.169	0.048	1.05E-14	8
Mcm6	2.85E-06	########	0.318	0.224	########	8
Ak3	2.16E-06	########	0.269	0.183	########	8
Sec11a	3.45E-07	########	0.624	0.608	########	8
Chn2	4.96E-35	########	0.211	0.044	8.30E-31	8
Tpr	1.19E-06	########	0.781	0.788	########	8
Snrnp70	1.23E-07	########	0.612	0.608	########	8
4930486L2	1.81E-79	########	0.174	0.013	3.02E-75	8
Rpl18a	1.82E-15	########	0.992	0.978	3.05E-11	8
Ndufa13	6.22E-14	########	0.901	0.882	1.04E-09	8
Mirt1	6.98E-11	########	0.326	0.181	1.17E-06	8
Sh3bgrl3	4.54E-11	########	0.983	0.968	7.60E-07	8
Myo6	1.65E-10	########	0.178	0.076	2.76E-06	8
Rps3	4.50E-14	########	0.988	0.967	7.53E-10	8
Ubald2	2.77E-07	########	0.587	0.566	########	8
2310036O2	3.41E-07	########	0.727	0.775	########	8
mt-Atp8	2.76E-09	########	0.756	0.734	4.61E-05	8
Tpt1	7.54E-20	########	1	0.998	1.26E-15	8
Acp5	9.15E-12	########	0.401	0.238	1.53E-07	8
Lef1	2.98E-48	########	0.372	0.082	4.98E-44	8
Gvin1	2.53E-28	########	0.174	0.036	4.23E-24	8
Cdca7l	3.77E-10	########	0.215	0.105	6.31E-06	8
Def6	6.47E-07	########	0.384	0.301	########	8
Surf6	4.00E-09	########	0.211	0.107	6.69E-05	8
Pglyrp2	1.62E-74	########	0.223	0.024	2.71E-70	8
Cox7b	1.99E-08	########	0.868	0.813	########	8
Lppos	1.02E-10	########	0.231	0.115	1.71E-06	8
Taf10	1.60E-07	########	0.752	0.749	########	8
Eif3m	3.20E-07	########	0.723	0.713	########	8
Rcsd1	1.08E-06	########	0.545	0.453	########	8
Zfp945	2.80E-08	########	0.215	0.115	########	8
Myl12a	1.36E-06	########	0.86	0.843	########	8

Abcb1a	1.05E-21	########	0.145	0.032	1.76E-17	8
Runx2	6.22E-09	########	0.248	0.134	########	8
Atp5j2	1.38E-11	########	0.926	0.867	2.31E-07	8
Nsd1	1.96E-06	########	0.545	0.535	########	8
Cox7c	2.37E-12	########	0.959	0.919	3.97E-08	8
2510002D2	4.85E-08	########	0.256	0.153	########	8
Ccr5	1.54E-09	########	0.38	0.225	2.58E-05	8
Uqcrh	2.42E-11	########	0.959	0.932	4.05E-07	8
Rpl32	2.94E-09	########	0.992	0.936	4.93E-05	8
Sf3b2	2.84E-07	########	0.744	0.774	########	8
Ncl	5.95E-12	########	0.917	0.823	9.95E-08	8
Cd72	5.63E-16	########	0.236	0.088	9.43E-12	8
Il27ra	8.48E-29	########	0.19	0.041	1.42E-24	8
Arl6ip1	2.33E-07	########	0.777	0.794	########	8
Tomm7	8.89E-09	########	0.855	0.872	########	8
4833403J1	2.02E-54	########	0.174	0.02	3.38E-50	8
Rpl14	1.87E-12	########	0.988	0.886	3.12E-08	8
Rps10	1.02E-14	########	0.996	0.983	1.70E-10	8
Zfhx2	2.24E-22	########	0.182	0.047	3.74E-18	8
Tmc8	1.78E-11	########	0.19	0.08	2.98E-07	8
Higd1a	6.41E-07	########	0.632	0.586	########	8
Cd200r4	1.91E-43	########	0.182	0.027	3.19E-39	8
Dek	5.69E-07	########	0.723	0.671	########	8
Rpl36a	2.49E-11	########	0.988	0.866	4.17E-07	8
Pak2	2.93E-06	########	0.793	0.818	########	8
Gm20069	3.05E-86	########	0.178	0.012	5.10E-82	8
Hmgb1	8.16E-16	########	0.938	0.879	1.36E-11	8
Wdr95	1.78E-57	########	0.107	0.007	2.98E-53	8
Ezr	3.16E-08	########	0.653	0.589	########	8
Gm42418	1.53E-17	########	1	1	2.57E-13	8
Ptma	2.97E-13	########	1	0.868	4.97E-09	8
Syne3	4.04E-07	########	0.194	0.107	########	8
Acss2	9.79E-14	########	0.207	0.081	1.64E-09	8
Qrfp	1.40E-115	########	0.136	0.004	2.34E-111	8
Enpp4	1.70E-11	########	0.174	0.069	2.84E-07	8
Mfng	5.60E-13	########	0.236	0.102	9.36E-09	8
A930024E0	1.46E-42	########	0.136	0.016	2.44E-38	8
Card11	3.55E-33	########	0.198	0.04	5.94E-29	8
Pabpc1	1.61E-07	########	0.938	0.925	########	8
Herc3	5.87E-13	########	0.207	0.084	9.82E-09	8
Sigirr	3.31E-16	########	0.174	0.055	5.53E-12	8
Atp8b2	2.81E-07	########	0.194	0.105	########	8
Cd96	4.19E-34	########	0.174	0.03	7.01E-30	8
Abca2	1.41E-09	########	0.178	0.079	2.36E-05	8
Gm14029	3.61E-129	########	0.145	0.004	6.05E-125	8
Rpl6	4.36E-10	########	1	0.941	7.29E-06	8
Nrarp	3.93E-14	########	0.161	0.053	6.58E-10	8
Nlrc5	1.37E-07	########	0.149	0.067	########	8
Sesn1	5.46E-07	########	0.285	0.18	########	8
Rpl8	1.08E-09	########	0.996	0.948	1.81E-05	8
Eif3e	7.97E-08	########	0.793	0.733	########	8
Rpl11	2.22E-09	########	1	0.95	3.71E-05	8

Slc25a53	8.02E-20	########	0.153	0.038	1.34E-15	8
Baiap3	5.21E-80	########	0.145	0.009	8.73E-76	8
Osbpl5	4.56E-10	########	0.145	0.056	7.64E-06	8
Sf3b6	1.00E-06	########	0.731	0.739	########	8
Jund	2.03E-06	########	0.946	0.893	########	8
Rnf157	6.72E-15	########	0.157	0.049	1.13E-10	8
Psmb3	1.45E-06	########	0.798	0.788	########	8
Cobll1	5.95E-11	########	0.153	0.057	9.96E-07	8
Rps28	1.30E-13	########	0.992	0.937	2.17E-09	8
Paip2b	3.37E-08	########	0.194	0.098	########	8
Rps5	9.65E-08	########	0.992	0.937	########	8
Rpl29	1.61E-06	########	0.955	0.91	########	8
Ppm1j	1.09E-53	########	0.136	0.012	1.82E-49	8
Pyhin1	5.72E-07	########	0.417	0.285	########	8
Cbx7	1.91E-09	########	0.145	0.056	3.19E-05	8
Hist1h4d	1.83E-19	########	0.545	0.318	3.06E-15	8
Plscr4	3.51E-08	########	0.124	0.05	########	8
Itm2a	3.17E-13	########	0.215	0.085	5.30E-09	8
mt-Nd5	7.78E-10	########	0.938	0.875	1.30E-05	8
Laptm5	4.20E-11	########	0.971	0.816	7.03E-07	8
Kcnip3	4.14E-27	########	0.132	0.023	6.93E-23	8
Eif1	1.26E-11	########	0.992	0.99	2.11E-07	8
Tpm3	2.25E-07	########	0.921	0.926	########	8
Tex9	2.43E-12	########	0.136	0.044	4.07E-08	8
Nck2	9.09E-13	########	0.145	0.048	1.52E-08	8
Trim14	1.04E-13	########	0.178	0.063	1.74E-09	8
Xlr4b	2.83E-19	########	0.186	0.054	4.74E-15	8
Pim2	2.64E-12	########	0.145	0.048	4.41E-08	8
Rps6	4.34E-08	########	0.996	0.937	########	8
Rpl4	1.22E-06	########	0.971	0.883	########	8
Btbd11	3.95E-21	########	0.149	0.035	6.60E-17	8
Pydc4	4.38E-07	########	0.161	0.077	########	8
Dtx1	5.17E-12	########	0.116	0.033	8.66E-08	8
D8Ertd82e	1.71E-06	########	0.145	0.071	########	8
Gpr68	3.04E-17	########	0.116	0.026	5.09E-13	8
Sidt1	5.92E-21	########	0.145	0.032	9.91E-17	8
Gm5547	1.65E-09	########	0.165	0.07	2.76E-05	8
Mterf1a	1.22E-07	########	0.149	0.067	########	8
Bst2	7.83E-52	4.453575	1	0.505	1.31E-47	9
Siglech	0	4.244213	0.882	0.002	0	9
Cox6a2	0	4.203635	0.912	0.012	0	9
Irf8	8.19E-53	3.898909	0.941	0.333	1.37E-48	9
Ly6d	6.67E-110	3.471407	0.882	0.097	1.12E-105	9
Tcf4	7.63E-53	3.260474	1	0.465	1.28E-48	9
Ccr9	0	3.097181	0.912	0.019	0	9
Rnase6	4.17E-95	2.99355	0.971	0.16	6.98E-91	9
Atp1b1	4.90E-139	2.647738	0.926	0.089	8.19E-135	9
Cd7	3.59E-226	2.644824	0.941	0.052	6.01E-222	9
Runx2	4.03E-92	2.561228	0.882	0.131	6.73E-88	9
Bcl11a	5.65E-93	2.528085	0.853	0.117	9.45E-89	9
Dnajc7	9.42E-48	2.345147	1	0.528	1.58E-43	9
Lair1	1.78E-68	2.277876	0.971	0.238	2.98E-64	9

St8sia4	1.74E-51	2.252225	1	0.382	2.91E-47	9
D13Ertd608	0	2.239968	0.691	0	0	9
Upb1	0	2.174136	0.824	0.019	0	9
Plac8	3.16E-38	2.155416	1	0.501	5.29E-34	9
Cd209d	2.75E-246	2.122792	0.456	0.009	4.60E-242	9
Tspan13	1.65E-59	2.068541	1	0.29	2.75E-55	9
Smim5	3.83E-121	2.024695	0.779	0.071	6.40E-117	9
Rpl31	3.45E-44	1.992721	1	0.906	5.77E-40	9
Nucb2	3.75E-47	1.968742	0.941	0.374	6.27E-43	9
Ccnd1	4.35E-53	1.957538	0.779	0.165	7.28E-49	9
Mpeg1	1.08E-39	1.914684	1	0.485	1.80E-35	9
Pafah1b3	1.39E-63	1.870703	0.912	0.222	2.33E-59	9
Kmo	1.08E-205	1.851106	0.824	0.044	1.81E-201	9
Rell1	6.97E-40	1.849572	0.882	0.354	1.17E-35	9
Pacsin1	0	1.821161	0.912	0.018	0	9
Pgls	5.61E-38	1.793853	0.985	0.719	9.38E-34	9
Mbnl1	1.34E-36	1.78515	1	0.801	2.25E-32	9
Ppfia4	1.17E-104	1.759147	0.897	0.114	1.96E-100	9
Mef2c	4.10E-45	1.747774	0.985	0.385	6.86E-41	9
Amica1	2.12E-57	1.738678	0.912	0.211	3.55E-53	9
P2ry14	2.60E-204	1.70362	0.75	0.035	4.36E-200	9
Dntt	0	1.701112	0.721	0.005	0	9
Blnk	2.08E-82	1.699992	0.853	0.123	3.48E-78	9
Bmyc	9.65E-63	1.692193	0.912	0.218	1.61E-58	9
Fyn	6.62E-44	1.689095	0.882	0.264	1.11E-39	9
Ly6c2	2.08E-28	1.680696	0.956	0.38	3.48E-24	9
Sell	4.49E-34	1.668955	0.985	0.478	7.52E-30	9
Ctsh	9.59E-35	1.666243	1	0.668	1.60E-30	9
Rpgrip1	3.86E-47	1.661404	0.838	0.238	6.45E-43	9
Cd300c	0	1.658958	0.75	0.004	0	9
Lifr	1.80E-56	1.657685	0.882	0.211	3.01E-52	9
Klrd1	2.13E-131	1.640521	0.912	0.078	3.57E-127	9
Irf2bp2	3.84E-35	1.610874	0.971	0.603	6.42E-31	9
Pld4	1.85E-36	1.600524	0.941	0.365	3.10E-32	9
Ly86	4.87E-41	1.589329	0.985	0.358	8.14E-37	9
Tmem229b	1.04E-124	1.588965	0.853	0.084	1.74E-120	9
Mvb12a	4.59E-44	1.573894	0.971	0.435	7.67E-40	9
Ly6e	3.90E-33	1.564171	1	0.814	6.53E-29	9
Ramp1	1.07E-39	1.55108	0.941	0.35	1.80E-35	9
Rnd3	2.44E-30	1.539456	0.676	0.208	4.08E-26	9
Cd47	9.37E-37	1.531706	1	0.899	1.57E-32	9
H2afy	7.95E-29	1.504619	0.985	0.78	1.33E-24	9
Tubgcp5	6.81E-49	1.481634	0.706	0.148	1.14E-44	9
Tsc22d1	5.98E-43	1.478005	0.809	0.219	1.00E-38	9
Spib	3.46E-214	1.471782	0.75	0.031	5.79E-210	9
Tex2	4.65E-101	1.471302	0.897	0.121	7.79E-97	9
H2-T23	1.71E-33	1.47058	1	0.682	2.87E-29	9
Gnas	1.17E-33	1.464391	1	0.882	1.97E-29	9
Prkca	2.00E-65	1.461552	0.794	0.135	3.35E-61	9
Ptms	4.25E-33	1.459874	0.985	0.499	7.11E-29	9
Lag3	0	1.441307	0.691	0.011	0	9
Bloc1s2	1.30E-39	1.429035	0.941	0.408	2.17E-35	9

Fcrla	6.24E-155	1.424793	0.691	0.037	1.04E-150	9
Rabgap1l	1.48E-47	1.418521	0.926	0.275	2.48E-43	9
Ptprcap	5.68E-52	1.416502	0.912	0.188	9.51E-48	9
Tifa	8.04E-25	1.41014	0.691	0.247	1.35E-20	9
Mctp2	5.76E-68	1.37861	0.75	0.113	9.64E-64	9
Fgfr1op2	4.48E-28	1.377464	0.956	0.688	7.50E-24	9
Smim14	5.60E-33	1.372945	0.971	0.623	9.36E-29	9
Kctd12	1.43E-25	1.36652	0.956	0.608	2.39E-21	9
Tagln2	9.06E-26	1.356836	1	0.756	1.52E-21	9
H2-DMa	1.90E-31	1.342146	0.941	0.358	3.18E-27	9
1-Sep	9.31E-37	1.333986	0.882	0.28	1.56E-32	9
Sms	2.31E-30	1.318394	0.809	0.319	3.86E-26	9
Dap	1.19E-28	1.316479	0.912	0.542	1.99E-24	9
Abhd17b	9.92E-40	1.304215	0.853	0.277	1.66E-35	9
Lrp8	4.69E-62	1.296632	0.559	0.068	7.84E-58	9
Unc93b1	2.27E-26	1.295471	0.956	0.623	3.80E-22	9
Gpr171	1.19E-100	1.291296	0.721	0.066	2.00E-96	9
Klk1	0	1.289935	0.309	0	0	9
Lefty1	1.15E-151	1.287137	0.721	0.046	1.93E-147	9
Cyth4	2.99E-22	1.28323	0.868	0.516	5.00E-18	9
Tnfrsf13b	3.68E-24	1.277849	0.691	0.261	6.16E-20	9
Net1	1.79E-44	1.276121	0.632	0.124	2.99E-40	9
Fyb	1.88E-24	1.275098	0.956	0.676	3.15E-20	9
Syngr2	1.35E-28	1.272535	0.941	0.551	2.26E-24	9
Herpud1	2.12E-28	1.271771	0.912	0.453	3.54E-24	9
Atp2a1	0	1.262622	0.632	0.005	0	9
Spns3	9.42E-154	1.259588	0.721	0.045	1.58E-149	9
Paqr5	0	1.244757	0.779	0.011	0	9
Psmb8	5.06E-29	1.233184	1	0.759	8.46E-25	9
Fdps	1.41E-21	1.216868	0.721	0.301	2.37E-17	9
Sh3bgr	0	1.211624	0.471	0.007	0	9
Dirc2	3.32E-30	1.206023	0.765	0.27	5.55E-26	9
Clec10a	8.60E-48	1.205902	0.765	0.154	1.44E-43	9
Ptprs	9.09E-39	1.201413	0.662	0.157	1.52E-34	9
Hsp90b1	5.87E-24	1.189508	1	0.766	9.83E-20	9
Apobec3	2.42E-26	1.188617	0.882	0.425	4.05E-22	9
Serinc3	1.54E-22	1.184869	0.971	0.828	2.57E-18	9
Pltp	2.80E-33	1.18123	0.853	0.241	4.69E-29	9
Klra17	3.02E-65	1.179699	0.544	0.059	5.05E-61	9
Lsp1	3.42E-19	1.179526	0.941	0.718	5.72E-15	9
Rnf187	2.74E-20	1.175462	0.838	0.467	4.58E-16	9
H13	1.15E-27	1.172588	0.971	0.604	1.92E-23	9
Pkib	6.53E-45	1.168645	0.721	0.153	1.09E-40	9
Snx5	8.46E-21	1.166513	0.882	0.559	1.42E-16	9
Cd209a	7.48E-159	1.163173	0.456	0.015	1.25E-154	9
Coro2a	3.41E-40	1.155843	0.676	0.153	5.71E-36	9
Selplg	3.80E-25	1.153513	0.985	0.637	6.35E-21	9
Cd164	2.48E-27	1.152574	0.926	0.552	4.14E-23	9
Rpl10	3.81E-35	1.146792	1	0.951	6.37E-31	9
Gm5547	8.62E-78	1.141939	0.632	0.068	1.44E-73	9
Cd180	3.37E-36	1.141543	0.721	0.19	5.64E-32	9
Psme1	2.46E-25	1.139376	0.971	0.803	4.12E-21	9

Khk	4.39E-22	1.137562	0.618	0.225	7.34E-18	9
Itpr1	1.39E-46	1.137103	0.824	0.21	2.32E-42	9
Hmgn3	1.01E-53	1.135895	0.765	0.15	1.69E-49	9
Cxxc5	3.68E-72	1.129693	0.706	0.095	6.15E-68	9
Serp1	2.33E-24	1.127941	0.985	0.787	3.90E-20	9
Arhgap17	7.46E-18	1.126584	0.794	0.495	1.25E-13	9
Sec61b	7.22E-27	1.125708	1	0.894	1.21E-22	9
Spcs2	4.25E-27	1.118901	0.971	0.725	7.11E-23	9
Gria3	1.81E-88	1.112592	0.647	0.064	3.04E-84	9
Trappc5	8.28E-23	1.111145	0.794	0.417	1.39E-18	9
Tbc1d8	2.39E-43	1.107631	0.735	0.171	3.99E-39	9
Srgap3	3.77E-121	1.101123	0.618	0.041	6.30E-117	9
AI662270	1.55E-19	1.096706	0.824	0.452	2.59E-15	9
Scimp	6.28E-82	1.092739	0.515	0.041	1.05E-77	9
Hmgb1	5.40E-18	1.091928	1	0.88	9.04E-14	9
Mtdh	4.38E-23	1.091895	0.971	0.659	7.33E-19	9
Plekhm3	1.98E-31	1.089964	0.676	0.192	3.31E-27	9
Slc44a2	2.62E-33	1.089724	0.809	0.283	4.39E-29	9
Flt3	1.61E-143	1.085128	0.588	0.03	2.69E-139	9
Scpep1	1.21E-26	1.082499	0.809	0.347	2.03E-22	9
Xbp1	2.01E-22	1.0798	0.941	0.555	3.37E-18	9
Il7r	9.62E-61	1.07726	0.838	0.13	1.61E-56	9
Sema4b	3.50E-77	1.074792	0.691	0.084	5.86E-73	9
Card11	2.32E-166	1.07256	0.721	0.039	3.88E-162	9
Sub1	1.34E-16	1.06538	1	0.911	2.24E-12	9
H2-Q7	5.30E-21	1.06252	0.706	0.235	8.86E-17	9
Hs3st1	4.61E-132	1.05796	0.618	0.037	7.71E-128	9
Uvrag	1.15E-20	1.057538	0.838	0.49	1.92E-16	9
Cbfa2t3	4.60E-27	1.057475	0.618	0.173	7.69E-23	9
Slamf9	2.44E-67	1.045375	0.5	0.048	4.08E-63	9
Tyrobp	2.67E-19	1.044761	1	0.743	4.48E-15	9
Csf2rb2	4.24E-32	1.042857	0.721	0.205	7.09E-28	9
Cnp	3.54E-36	1.042575	0.838	0.266	5.93E-32	9
Abhd17a	4.19E-29	1.038397	0.941	0.505	7.02E-25	9
Magt1	6.59E-22	1.024758	0.838	0.453	1.10E-17	9
Sik1	7.04E-23	1.021194	0.794	0.351	1.18E-18	9
Tmed3	7.83E-24	1.018419	0.926	0.524	1.31E-19	9
Manf	2.01E-21	1.011663	1	0.6	3.37E-17	9
Hivep3	3.02E-32	1.011379	0.529	0.111	5.05E-28	9
Tcf12	3.23E-26	1.007281	0.809	0.339	5.41E-22	9
9930111J2	1.87E-19	1.006051	0.779	0.366	3.13E-15	9
Ikzf1	4.54E-21	1.00263	0.853	0.463	7.60E-17	9
Sec11c	2.05E-21	########	0.971	0.747	3.43E-17	9
Ybx3	3.41E-26	########	0.941	0.424	5.71E-22	9
Rps6ka1	8.63E-23	########	0.809	0.388	1.44E-18	9
Lgals1	2.38E-17	########	0.985	0.657	3.98E-13	9
Eepd1	6.98E-31	########	0.632	0.164	1.17E-26	9
Rps11	7.84E-27	########	1	0.965	1.31E-22	9
Sla2	6.65E-129	########	0.544	0.028	1.11E-124	9
Ppia	7.57E-24	########	1	0.904	1.27E-19	9
Bcl7c	4.65E-30	########	0.941	0.43	7.78E-26	9
Smc6	1.79E-09	########	0.735	0.492	2.99E-05	9

Apc	9.45E-20	########	0.721	0.333	1.58E-15	9
Ech1	3.36E-16	########	0.809	0.572	5.62E-12	9
Tfrc	2.99E-26	########	0.647	0.198	5.00E-22	9
Pkig	4.72E-18	########	0.794	0.435	7.90E-14	9
P2ry10	5.86E-32	########	0.412	0.061	9.81E-28	9
Krtcap2	2.02E-20	########	0.985	0.668	3.39E-16	9
Tcf7l2	1.29E-16	########	0.544	0.2	2.16E-12	9
Arhgap5	3.17E-30	########	0.676	0.198	5.30E-26	9
Mzb1	9.49E-45	########	0.382	0.038	1.59E-40	9
Rexo2	1.19E-15	########	0.824	0.508	1.98E-11	9
Ifnar2	1.06E-16	########	0.853	0.595	1.78E-12	9
Vimp	5.49E-16	########	0.897	0.583	9.19E-12	9
Nptn	1.03E-20	########	0.926	0.613	1.72E-16	9
Slc38a1	4.84E-21	########	0.779	0.345	8.10E-17	9
Sec61g	1.39E-22	########	1	0.865	2.33E-18	9
Idi1	1.21E-16	########	0.559	0.218	2.02E-12	9
Gltp	1.00E-18	########	0.868	0.522	1.67E-14	9
Arhgap27os	1.06E-128	########	0.544	0.029	1.77E-124	9
Tmem59	2.12E-17	########	0.926	0.764	3.55E-13	9
Rab33b	2.77E-39	########	0.632	0.136	4.63E-35	9
Ptp4a3	2.24E-23	########	0.706	0.253	3.75E-19	9
Eml4	6.09E-21	########	0.735	0.325	1.02E-16	9
Fam174a	8.09E-19	########	0.838	0.464	1.35E-14	9
Ccdc88a	7.21E-14	########	0.691	0.364	1.21E-09	9
Pde7a	8.59E-30	########	0.618	0.16	1.44E-25	9
Cd2ap	6.59E-16	########	0.75	0.401	1.10E-11	9
Chdh	9.68E-122	########	0.588	0.037	1.62E-117	9
Orai3	1.16E-26	########	0.662	0.214	1.93E-22	9
Fmnl2	1.21E-38	########	0.662	0.149	2.02E-34	9
Atp6v1d	1.38E-12	########	0.853	0.598	2.31E-08	9
Psmb9	2.65E-16	########	0.853	0.571	4.44E-12	9
Napsa	1.85E-19	########	0.971	0.471	3.10E-15	9
Hnrnpa1	1.46E-16	########	0.926	0.682	2.44E-12	9
Ifnar1	1.37E-13	########	0.721	0.417	2.29E-09	9
Dctn6	3.91E-20	########	0.706	0.332	6.54E-16	9
Arhgef6	3.36E-18	########	0.765	0.385	5.62E-14	9
Tram1	2.73E-16	########	0.882	0.641	4.57E-12	9
Cybb	3.87E-16	########	0.956	0.54	6.47E-12	9
Pmvk	2.06E-22	########	0.735	0.317	3.45E-18	9
Stx7	4.97E-19	########	0.897	0.566	8.32E-15	9
Bmp2k	2.71E-14	########	0.765	0.434	4.54E-10	9
Grn	1.06E-18	########	1	0.696	1.77E-14	9
Calcoco1	3.29E-54	########	0.647	0.101	5.50E-50	9
Pdzd4	1.18E-133	########	0.515	0.025	1.97E-129	9
Cd200	2.69E-31	########	0.485	0.087	4.49E-27	9
11-Sep	8.61E-13	########	0.721	0.418	1.44E-08	9
Rpl36al	1.66E-18	########	1	0.841	2.78E-14	9
Clec2d	1.37E-13	########	0.824	0.537	2.29E-09	9
Rgs18	1.06E-15	########	0.647	0.292	1.78E-11	9
Dbnl	5.51E-13	########	0.824	0.591	9.22E-09	9
Psap	1.70E-18	########	1	0.854	2.84E-14	9
Rap1a	4.89E-19	########	0.971	0.8	8.18E-15	9

Ptpn6	5.55E-13	########	0.882	0.604	9.29E-09	9
Nceh1	7.10E-19	########	0.75	0.331	1.19E-14	9
Cirbp	6.42E-14	########	0.721	0.406	1.07E-09	9
Elf1	6.94E-15	########	0.853	0.587	1.16E-10	9
Stat2	5.85E-26	########	0.559	0.145	9.79E-22	9
Itga4	7.06E-17	########	0.912	0.475	1.18E-12	9
Ppdpf	2.63E-15	########	0.794	0.463	4.40E-11	9
Cacna1e	6.88E-260	########	0.5	0.01	1.15E-255	9
Dock10	8.17E-17	########	0.824	0.442	1.37E-12	9
Msmo1	2.68E-09	########	0.515	0.275	4.48E-05	9
Inpp4a	4.71E-14	########	0.559	0.245	7.88E-10	9
Clec12a	1.99E-13	0.794006	0.779	0.419	3.32E-09	9
Ivns1abp	8.08E-19	########	0.706	0.317	1.35E-14	9
Arl5c	6.26E-24	########	0.647	0.196	1.05E-19	9
Spint2	4.34E-32	########	0.647	0.159	7.27E-28	9
Cd48	3.46E-17	########	0.897	0.432	5.79E-13	9
Tmem258	2.06E-15	########	0.971	0.75	3.44E-11	9
Sema4d	3.64E-24	########	0.853	0.353	6.08E-20	9
Map3k1	9.84E-18	########	0.765	0.361	1.65E-13	9
mt-Nd4	9.85E-17	########	1	0.961	1.65E-12	9
Npc1	1.10E-15	########	0.721	0.349	1.84E-11	9
Rftn1	1.16E-15	########	0.559	0.21	1.93E-11	9
Syne2	3.28E-11	########	0.529	0.243	5.48E-07	9
Pkp3	1.45E-30	########	0.559	0.118	2.43E-26	9
Akap9	7.89E-13	########	0.735	0.432	1.32E-08	9
Hes6	7.76E-15	########	0.544	0.223	1.30E-10	9
Ppp1r14b	5.50E-17	########	0.971	0.557	9.20E-13	9
Ldhb	8.63E-22	########	0.515	0.146	1.44E-17	9
BC035044	1.41E-35	########	0.426	0.063	2.36E-31	9
Rfwd2	9.88E-15	########	0.794	0.492	1.65E-10	9
Med10	4.82E-17	########	0.765	0.423	8.06E-13	9
mt-Nd5	4.00E-17	########	1	0.876	6.69E-13	9
Klk1b27	0	########	0.206	0	0	9
Hoxa7	1.51E-58	########	0.515	0.059	2.53E-54	9
Txndc5	1.42E-13	########	0.647	0.3	2.38E-09	9
Stk4	2.43E-14	########	0.824	0.557	4.06E-10	9
Frmd4a	3.62E-24	########	0.588	0.177	6.06E-20	9
Man2a2	1.67E-20	########	0.618	0.222	2.80E-16	9
Jakmip1	1.59E-27	########	0.397	0.066	2.67E-23	9
Hp1bp3	6.60E-13	########	0.794	0.554	1.10E-08	9
Cd74	3.39E-14	########	0.721	0.324	5.67E-10	9
H2-M3	1.25E-19	########	0.676	0.267	2.10E-15	9
Sptssa	8.20E-14	########	0.838	0.576	1.37E-09	9
Them6	1.54E-29	########	0.471	0.093	2.58E-25	9
Cldnd1	1.49E-15	########	0.662	0.321	2.50E-11	9
Lmo2	7.97E-19	########	0.794	0.38	1.33E-14	9
Pnn	3.43E-10	########	0.75	0.51	5.74E-06	9
Oaz1	4.80E-22	########	1	0.969	8.04E-18	9
Luc7l2	2.71E-13	########	0.897	0.765	4.54E-09	9
6-Sep	3.66E-20	########	0.647	0.231	6.13E-16	9
0610007P1	5.18E-13	########	0.618	0.337	8.66E-09	9
Cyp51	1.22E-08	########	0.441	0.207	########	9

Rassf4	8.55E-18	########	0.75	0.299	1.43E-13	9
Snw1	2.20E-13	########	0.794	0.53	3.68E-09	9
Gna15	1.90E-16	########	0.471	0.159	3.18E-12	9
Rac2	8.78E-13	########	0.971	0.739	1.47E-08	9
Ogt	8.48E-12	########	0.794	0.544	1.42E-07	9
Tmem173	5.82E-17	########	0.706	0.323	9.75E-13	9
Iqgap2	3.76E-12	########	0.706	0.399	6.30E-08	9
Mndal	5.32E-14	########	0.765	0.38	8.90E-10	9
Anp32e	2.71E-11	########	0.779	0.47	4.54E-07	9
Ctsl	3.31E-15	########	0.971	0.585	5.53E-11	9
Kynu	4.44E-115	########	0.485	0.025	7.42E-111	9
Hmgcr	8.64E-11	########	0.574	0.311	1.45E-06	9
Ppm1h	7.52E-14	########	0.574	0.251	1.26E-09	9
Sp140	7.45E-11	########	0.735	0.433	1.25E-06	9
Fndc3a	2.93E-15	########	0.779	0.421	4.90E-11	9
H2-K1	1.64E-11	########	1	0.902	2.75E-07	9
Hhex	6.03E-12	########	0.574	0.28	1.01E-07	9
Ifi203	2.82E-07	########	0.632	0.358	########	9
Map4k1	3.34E-16	########	0.5	0.166	5.59E-12	9
Tbxa2r	3.91E-84	########	0.515	0.039	6.54E-80	9
Sp110	3.75E-09	########	0.662	0.409	6.27E-05	9
Sumo2	2.92E-12	########	0.956	0.782	4.88E-08	9
Slc29a3	2.40E-20	########	0.676	0.254	4.02E-16	9
Gns	1.97E-11	########	0.706	0.406	3.30E-07	9
Runx3	1.28E-09	########	0.588	0.291	2.14E-05	9
Spn	1.22E-18	########	0.618	0.216	2.05E-14	9
Sec24d	1.24E-23	########	0.588	0.175	2.08E-19	9
Aff3	9.09E-38	########	0.471	0.07	1.52E-33	9
Tnfaip8	8.65E-12	########	0.897	0.661	1.45E-07	9
Ddr1	2.56E-48	########	0.441	0.052	4.28E-44	9
Phactr2	7.60E-18	########	0.647	0.275	1.27E-13	9
Limd1	1.77E-12	########	0.676	0.369	2.96E-08	9
Ubxn4	3.04E-10	########	0.824	0.638	5.08E-06	9
L3mbtl3	8.76E-25	########	0.529	0.137	1.47E-20	9
Ctcf	3.85E-10	########	0.75	0.531	6.43E-06	9
Sepw1	1.24E-12	########	0.985	0.671	2.08E-08	9
Sdc4	9.74E-12	########	0.809	0.386	1.63E-07	9
Trim30a	3.98E-09	########	0.721	0.483	6.65E-05	9
Mgat1	1.06E-13	########	0.721	0.399	1.77E-09	9
Rgs10	2.78E-09	########	0.794	0.51	4.65E-05	9
Cdc42se2	2.06E-12	########	0.735	0.435	3.44E-08	9
Ptma	6.29E-12	########	1	0.871	1.05E-07	9
Bcr	9.61E-19	########	0.471	0.138	1.61E-14	9
Pip4k2a	1.08E-10	########	0.735	0.465	1.80E-06	9
Hnrnpa3	6.14E-12	########	0.985	0.835	1.03E-07	9
Setd2	7.65E-09	########	0.676	0.476	########	9
Atp13a2	1.27E-13	########	0.735	0.361	2.13E-09	9
Bzw1	3.80E-08	########	0.853	0.768	########	9
Srebf2	7.56E-12	########	0.574	0.29	1.27E-07	9
Npc2	3.07E-12	########	1	0.829	5.14E-08	9
Eif3f	1.22E-14	########	0.985	0.862	2.04E-10	9
Cops4	1.47E-12	########	0.706	0.414	2.46E-08	9

Nsmaf	5.86E-18	########	0.485	0.152	9.80E-14	9
Psme2	6.15E-11	########	0.882	0.736	1.03E-06	9
Rpl13a	2.36E-17	########	1	0.963	3.94E-13	9
Prr5	2.41E-33	########	0.441	0.073	4.04E-29	9
Snx9	3.96E-14	########	0.632	0.305	6.63E-10	9
Gapt	1.08E-22	########	0.544	0.154	1.81E-18	9
Fam3c	5.73E-17	########	0.706	0.322	9.59E-13	9
Scand1	2.28E-14	########	0.971	0.862	3.81E-10	9
Ubn1	1.40E-12	########	0.779	0.516	2.34E-08	9
PISD	2.29E-14	########	0.721	0.368	3.84E-10	9
H3f3b	1.28E-16	########	1	0.99	2.15E-12	9
Cmah	8.20E-15	########	0.574	0.206	1.37E-10	9
mt-Nd3	5.43E-13	########	1	0.845	9.08E-09	9
Emp3	1.18E-10	########	0.985	0.842	1.98E-06	9
Ctsz	2.87E-11	########	0.971	0.734	4.80E-07	9
Pik3ip1	2.35E-27	########	0.574	0.133	3.94E-23	9
Epsti1	7.23E-12	########	0.765	0.431	1.21E-07	9
Gpr137b	1.06E-12	########	0.544	0.219	1.78E-08	9
Ccdc162	1.20E-195	########	0.294	0.004	2.01E-191	9
Psmb10	7.61E-10	########	0.809	0.589	1.27E-05	9
Xkrx	2.79E-181	########	0.412	0.01	4.67E-177	9
mt-Nd2	1.44E-14	########	1	0.892	2.41E-10	9
Emc3	4.21E-13	########	0.721	0.431	7.04E-09	9
Anapc5	1.48E-10	########	0.75	0.479	2.48E-06	9
Ywhae	5.46E-11	########	0.956	0.784	9.13E-07	9
Jade1	2.69E-12	########	0.441	0.174	4.50E-08	9
Prcp	7.94E-11	########	0.721	0.414	1.33E-06	9
Rps26	2.29E-14	########	1	0.949	3.83E-10	9
H2-Aa	8.51E-15	########	0.574	0.19	1.42E-10	9
Trp53i11	7.46E-36	########	0.485	0.077	1.25E-31	9
Phgdh	1.95E-19	########	0.559	0.165	3.27E-15	9
Cdkn2d	6.32E-10	########	0.824	0.525	1.06E-05	9
Atxn7l3b	2.06E-10	########	0.824	0.635	3.45E-06	9
Irf1	2.03E-11	########	0.779	0.461	3.39E-07	9
Rnaset2b	2.39E-16	########	0.618	0.263	4.01E-12	9
Cbl	3.91E-10	########	0.824	0.627	6.54E-06	9
mt-Nd4l	4.51E-11	########	0.956	0.749	7.54E-07	9
Rps14	1.48E-16	########	1	0.984	2.48E-12	9
Padi2	1.43E-43	########	0.515	0.074	2.39E-39	9
Tifab	7.33E-18	########	0.485	0.142	1.23E-13	9
Rbm39	1.43E-15	########	1	0.919	2.39E-11	9
Tmem147	2.48E-11	########	0.765	0.473	4.15E-07	9
Dpm3	1.42E-11	########	0.838	0.598	2.38E-07	9
Senp6	1.69E-09	########	0.735	0.567	2.83E-05	9
Lrrc16a	1.95E-45	########	0.441	0.054	3.27E-41	9
Tmem163	6.11E-68	########	0.397	0.028	1.02E-63	9
Etnk1	6.86E-10	########	0.647	0.391	1.15E-05	9
Psmg4	9.69E-12	########	0.735	0.461	1.62E-07	9
Foxp1	5.93E-12	########	0.912	0.664	9.93E-08	9
Zc3h12c	8.48E-17	########	0.368	0.093	1.42E-12	9
Nsa2	1.65E-10	########	0.926	0.808	2.76E-06	9
Fam178a	7.54E-09	########	0.5	0.263	########	9

Acbd5	1.94E-11	########	0.662	0.377	3.24E-07	9
Inafm2	7.40E-28	########	0.485	0.105	1.24E-23	9
Chst15	2.06E-29	########	0.559	0.125	3.45E-25	9
Slc41a2	2.28E-35	########	0.5	0.09	3.81E-31	9
Sqle	1.34E-16	########	0.412	0.118	2.24E-12	9
Scap	2.02E-15	########	0.456	0.154	3.38E-11	9
Rps28	4.85E-15	########	1	0.938	8.11E-11	9
Cnot6l	8.75E-09	########	0.706	0.484	########	9
Rpl30	5.15E-18	########	1	0.976	8.61E-14	9
Tubb2b	5.55E-19	########	0.382	0.087	9.28E-15	9
Stambpl1	5.90E-15	########	0.485	0.171	9.88E-11	9
Me2	3.04E-10	########	0.632	0.367	5.08E-06	9
Rpl26	6.38E-14	########	1	0.968	1.07E-09	9
Wasf2	6.81E-09	########	0.838	0.72	########	9
Dcaf8	1.58E-13	########	0.721	0.407	2.65E-09	9
Ucp2	8.16E-11	########	0.956	0.815	1.37E-06	9
Hnrnpr	8.05E-11	########	0.735	0.47	1.35E-06	9
Sox4	7.77E-09	########	0.368	0.138	########	9
Tomm7	2.90E-14	########	0.971	0.871	4.84E-10	9
Pdcd4	4.82E-12	########	0.809	0.463	8.06E-08	9
Epha2	2.76E-46	########	0.426	0.05	4.61E-42	9
Imp3	4.27E-09	########	0.75	0.497	7.15E-05	9
Fkbp2	7.54E-11	########	0.926	0.563	1.26E-06	9
Dpp4	5.50E-28	########	0.485	0.094	9.20E-24	9
Gm14964	3.82E-185	########	0.294	0.005	6.40E-181	9
Cxcr3	2.06E-60	########	0.353	0.026	3.45E-56	9
Zmiz2	2.44E-14	########	0.574	0.242	4.09E-10	9
B2m	1.75E-10	########	1	0.971	2.93E-06	9
Nr2c2ap	3.09E-13	########	0.515	0.21	5.18E-09	9
Arhgap30	2.94E-09	########	0.824	0.605	4.92E-05	9
Irf7	3.47E-12	########	0.574	0.24	5.81E-08	9
Slc25a53	1.24E-73	########	0.471	0.038	2.07E-69	9
Trmt112	1.65E-10	########	0.853	0.623	2.76E-06	9
Tacc1	3.35E-11	########	0.809	0.527	5.60E-07	9
Cdh5	2.41E-66	########	0.515	0.047	4.03E-62	9
Gm5617	1.08E-09	########	0.529	0.28	1.81E-05	9
Krit1	6.41E-10	########	0.691	0.445	1.07E-05	9
Bod1l	1.53E-06	########	0.676	0.544	########	9
Galk1	4.64E-13	########	0.574	0.242	7.77E-09	9
Traf4	2.98E-18	########	0.309	0.063	4.98E-14	9
Ttc14	1.71E-12	########	0.779	0.484	2.86E-08	9
Ppm1m	8.65E-09	########	0.603	0.353	########	9
Ubl3	3.74E-10	########	0.882	0.666	6.26E-06	9
Zfp706	3.95E-10	########	0.838	0.636	6.61E-06	9
Tmem160	4.78E-10	########	0.897	0.679	8.01E-06	9
Haus3	1.44E-08	########	0.397	0.177	########	9
2410015M2	3.21E-10	########	0.912	0.681	5.37E-06	9
Stx16	1.62E-09	########	0.588	0.34	2.71E-05	9
Prrc2c	2.45E-10	########	0.956	0.79	4.09E-06	9
Hivep1	1.01E-10	########	0.559	0.291	1.70E-06	9
Zmynd8	2.51E-10	########	0.676	0.398	4.20E-06	9
Jtb	6.11E-08	########	0.735	0.53	########	9

Cdc14b	9.79E-29	########	0.368	0.058	1.64E-24	9
Rai1	4.54E-17	########	0.456	0.139	7.60E-13	9
Dnajc3	2.07E-10	########	0.868	0.585	3.47E-06	9
Rpl37	2.35E-12	########	1	0.982	3.93E-08	9
Cdh1	1.61E-54	########	0.397	0.035	2.69E-50	9
Prss30	1.14E-249	########	0.338	0.004	1.91E-245	9
Spcs3	8.96E-11	########	0.647	0.382	1.50E-06	9
mt-Co3	8.42E-16	########	1	0.991	1.41E-11	9
Fam117a	9.96E-17	########	0.515	0.171	1.67E-12	9
Srsf3	1.19E-07	########	0.956	0.782	########	9
Tmed9	8.73E-10	########	0.882	0.671	1.46E-05	9
Rab6b	2.24E-39	########	0.441	0.061	3.75E-35	9
mt-Co1	9.43E-19	########	1	0.993	1.58E-14	9
Smdt1	3.81E-11	########	0.985	0.812	6.38E-07	9
R3hdm4	1.49E-08	########	0.765	0.54	########	9
Pml	1.89E-18	########	0.5	0.152	3.17E-14	9
H2-DMb1	3.60E-18	########	0.647	0.222	6.02E-14	9
Rcsd1	1.58E-09	########	0.75	0.453	2.65E-05	9
Ccr5	1.02E-08	########	0.5	0.228	########	9
Rps13	1.82E-14	########	1	0.975	3.05E-10	9
Rrbp1	7.54E-11	########	0.985	0.787	1.26E-06	9
Trim25	5.56E-07	########	0.662	0.445	########	9
Cmtm7	6.56E-10	########	0.897	0.672	1.10E-05	9
Fubp1	4.40E-09	########	0.735	0.523	7.37E-05	9
Dek	1.18E-07	########	0.912	0.671	########	9
Exosc5	1.73E-10	########	0.632	0.36	2.89E-06	9
Snrpd2	3.51E-09	########	0.926	0.693	5.87E-05	9
Ift57	3.07E-16	########	0.5	0.177	5.14E-12	9
Tgfbr1	1.30E-10	########	0.603	0.317	2.17E-06	9
Eps15	1.89E-07	########	0.647	0.465	########	9
Pla2g16	2.21E-16	########	0.588	0.211	3.70E-12	9
Dapp1	2.55E-11	########	0.662	0.351	4.27E-07	9
Rpl35a	4.66E-15	########	1	0.98	7.80E-11	9
Celf2	1.23E-07	########	0.853	0.72	########	9
Nono	1.80E-08	########	0.765	0.584	########	9
Zfp644	6.56E-08	########	0.647	0.436	########	9
Tomm20	8.46E-08	########	0.971	0.758	########	9
Phf14	5.87E-07	########	0.632	0.458	########	9
Uggt1	7.59E-08	########	0.588	0.388	########	9
2700060E0	6.11E-09	########	0.868	0.686	########	9
Churc1	5.51E-10	########	0.765	0.532	9.23E-06	9
Cetn2	7.57E-10	########	0.662	0.415	1.27E-05	9
Rcc2	6.70E-08	########	0.588	0.373	########	9
Mycl	1.34E-162	########	0.353	0.008	2.25E-158	9
Rpl11	1.97E-10	########	1	0.951	3.30E-06	9
Rps25	5.28E-14	########	1	0.967	8.83E-10	9
Abcf1	1.36E-09	########	0.794	0.525	2.27E-05	9
Fkbp4	1.35E-07	########	0.588	0.354	########	9
Arpc5l	6.44E-10	########	0.735	0.512	1.08E-05	9
Lpgat1	2.88E-06	########	0.529	0.355	########	9
Edem2	8.67E-09	########	0.559	0.334	########	9
Prpf4b	2.42E-11	########	0.809	0.552	4.05E-07	9

Fgr	2.75E-07	########	0.691	0.433	########	9
Rpl18a	7.88E-12	########	1	0.978	1.32E-07	9
Gltscr2	6.69E-09	########	0.897	0.707	########	9
Pmepa1	1.27E-09	########	0.5	0.194	2.13E-05	9
Ap1ar	3.60E-10	########	0.441	0.192	6.03E-06	9
Pole4	2.27E-11	########	0.706	0.407	3.79E-07	9
Glrx3	5.18E-10	########	0.824	0.53	8.67E-06	9
Tmed2	7.90E-09	########	0.912	0.783	########	9
Eif3e	7.60E-09	########	0.941	0.733	########	9
Prr13	1.98E-11	########	0.956	0.705	3.32E-07	9
Pir	5.02E-15	########	0.294	0.068	8.40E-11	9
Gm26778	3.64E-27	########	0.441	0.086	6.09E-23	9
Pip5k1c	5.14E-08	########	0.529	0.294	########	9
Hn1	3.59E-08	########	0.897	0.714	########	9
Ube2e1	5.01E-09	########	0.662	0.431	8.37E-05	9
C1galt1	4.11E-10	########	0.618	0.348	6.87E-06	9
Saraf	7.36E-10	########	0.75	0.511	1.23E-05	9
Gm2a	2.76E-09	########	0.897	0.594	4.61E-05	9
Rpl28	4.12E-10	########	1	0.963	6.89E-06	9
Rpl21	3.02E-12	########	1	0.949	5.06E-08	9
Asxl2	2.83E-07	########	0.544	0.347	########	9
Itgax	2.72E-35	########	0.368	0.045	4.54E-31	9
Cnbp	8.71E-08	########	0.926	0.709	########	9
Selt	8.86E-10	########	0.794	0.594	1.48E-05	9
Nktr	3.35E-10	########	0.853	0.609	5.61E-06	9
Cib1	2.51E-08	########	0.779	0.576	########	9
Itgb7	3.51E-14	########	0.691	0.272	5.87E-10	9
Ppil4	1.90E-08	########	0.632	0.426	########	9
Hnrnpf	2.56E-10	########	0.985	0.89	4.28E-06	9
Nudt16	2.72E-17	########	0.412	0.11	4.55E-13	9
mt-Co2	4.11E-16	########	1	0.989	6.87E-12	9
Rbbp6	4.52E-07	########	0.838	0.634	########	9
Rps27	8.45E-19	########	1	0.977	1.41E-14	9
Strbp	3.65E-14	########	0.5	0.179	6.10E-10	9
Bptf	1.89E-07	########	0.794	0.64	########	9
Reep5	2.42E-09	########	0.985	0.817	4.06E-05	9
Laptm5	8.79E-10	########	1	0.819	1.47E-05	9
Ppp1r11	4.03E-10	########	0.779	0.53	6.74E-06	9
Pcyt2	1.99E-16	########	0.441	0.134	3.33E-12	9
Lat2	5.04E-12	########	0.676	0.316	8.43E-08	9
Vars	2.69E-11	########	0.618	0.31	4.51E-07	9
Rfc1	6.10E-07	########	0.618	0.432	########	9
Zcchc24	6.45E-07	########	0.412	0.208	########	9
Nudt19	6.03E-12	########	0.5	0.213	1.01E-07	9
Clec9a	3.86E-147	########	0.265	0.005	6.46E-143	9
Evi5	3.31E-08	########	0.485	0.259	########	9
Ctso	1.95E-08	########	0.397	0.172	########	9
Gm26759	1.09E-29	########	0.324	0.044	1.82E-25	9
Hnrnpul1	1.84E-09	########	0.765	0.56	3.08E-05	9
Tapbp	9.36E-09	########	0.838	0.616	########	9
Srsf11	5.00E-10	########	0.853	0.663	8.36E-06	9
Fam69a	5.20E-08	########	0.529	0.299	########	9

Malat1	7.34E-09	########	1	0.993	########	9
Ufc1	6.84E-09	########	0.882	0.603	########	9
Havcr1	0	########	0.353	0	0	9
mt-Atp6	7.08E-14	########	1	0.989	1.18E-09	9
Snx18	1.62E-06	########	0.691	0.526	########	9
Nol7	1.10E-08	########	0.882	0.663	########	9
Ube3a	4.07E-08	########	0.75	0.556	########	9
Luc7l3	6.07E-08	########	0.809	0.601	########	9
Cope	8.44E-09	########	0.868	0.635	########	9
Pura	1.03E-06	########	0.676	0.515	########	9
Bex6	1.07E-17	########	0.221	0.034	1.79E-13	9
Trim44	1.86E-10	########	0.529	0.247	3.11E-06	9
Tmem221	7.69E-159	########	0.353	0.009	1.29E-154	9
Rnf122	3.05E-32	########	0.368	0.053	5.11E-28	9
Zdhhc13	1.09E-21	########	0.426	0.1	1.82E-17	9
Serinc5	2.92E-09	########	0.309	0.106	4.89E-05	9
Tmem55b	2.89E-08	########	0.574	0.341	########	9
Commd3	5.07E-08	########	0.691	0.472	########	9
Insig1	5.76E-08	########	0.456	0.226	########	9
Thrap3	1.34E-07	########	0.794	0.62	########	9
Hibadh	2.89E-08	########	0.559	0.342	########	9
Trim24	1.49E-12	########	0.485	0.192	2.49E-08	9
Gpr146	3.08E-12	########	0.544	0.231	5.16E-08	9
Macf1	2.32E-07	########	0.824	0.678	########	9
Commd6	1.05E-08	########	0.706	0.463	########	9
Nudcd3	1.84E-08	########	0.632	0.388	########	9
Gng2	4.32E-08	########	0.809	0.554	########	9
Tnfaip8l2	3.94E-07	########	0.765	0.482	########	9
Rps3a1	4.59E-11	########	1	0.968	7.68E-07	9
Mettl23	1.91E-07	########	0.691	0.508	########	9
Zfp398	1.14E-16	########	0.397	0.104	1.90E-12	9
Cd8a	5.91E-23	########	0.265	0.035	9.90E-19	9
Man1a2	3.56E-09	########	0.676	0.413	5.95E-05	9
Cdip1	7.82E-12	########	0.309	0.09	1.31E-07	9
Atp5g2	2.23E-07	########	0.985	0.815	########	9
Gna11	6.38E-09	########	0.441	0.202	########	9
Sf1	3.07E-07	########	0.721	0.535	########	9
Rpl39	7.25E-12	########	1	0.959	1.21E-07	9
Slc25a12	3.70E-09	########	0.485	0.245	6.19E-05	9
Fryl	2.93E-06	########	0.515	0.331	########	9
Rpl3	5.70E-08	########	1	0.868	########	9
Rpl19	1.06E-09	########	1	0.963	1.77E-05	9
I830077J02	6.01E-11	########	0.529	0.237	1.01E-06	9
Nup210	2.39E-13	########	0.441	0.146	3.99E-09	9
Rps20	7.39E-10	########	1	0.936	1.24E-05	9
Rpl36	3.92E-10	########	1	0.907	6.57E-06	9
Lman2l	2.22E-10	########	0.456	0.197	3.72E-06	9
Mri1	4.09E-10	########	0.5	0.231	6.84E-06	9
Msi2	5.76E-10	########	0.456	0.194	9.64E-06	9
Zdhhc14	5.40E-22	########	0.412	0.089	9.03E-18	9
Ero1lb	1.86E-10	########	0.397	0.147	3.11E-06	9
Klf13	1.21E-06	########	0.926	0.761	########	9

Mrpl18	7.89E-07	########	0.765	0.565	########	9
Fbl	1.47E-09	########	0.779	0.492	2.46E-05	9
Clcn5	1.10E-11	########	0.515	0.21	1.83E-07	9
Nme7	2.78E-15	########	0.382	0.109	4.66E-11	9
Fgd2	2.94E-11	########	0.397	0.139	4.92E-07	9
Rps4x	4.88E-09	########	1	0.939	8.17E-05	9
1110059E2	1.11E-09	########	0.515	0.258	1.86E-05	9
Gne	3.42E-23	########	0.382	0.077	5.71E-19	9
Nop10	1.85E-07	########	0.853	0.594	########	9
Gnptab	7.37E-08	########	0.426	0.207	########	9
Ssr1	5.45E-07	########	0.765	0.583	########	9
Clec2i	1.04E-07	########	0.338	0.131	########	9
Arglu1	2.01E-07	########	0.75	0.617	########	9
Hmgn1	5.97E-10	########	0.794	0.412	9.99E-06	9
Taok3	1.52E-08	########	0.735	0.509	########	9
Filip1l	5.46E-08	########	0.632	0.348	########	9
Ptprf	2.31E-20	########	0.338	0.065	3.86E-16	9
Hspe1	1.07E-08	########	0.956	0.674	########	9
Mtpn	2.52E-06	########	0.824	0.695	########	9
Srp14	1.86E-08	########	0.912	0.79	########	9
Plekho1	1.57E-07	########	0.647	0.373	########	9
Ccdc107	2.51E-07	########	0.471	0.261	########	9
Swi5	1.13E-08	########	0.912	0.692	########	9
Bri3bp	2.75E-09	########	0.559	0.277	4.60E-05	9
Syf2	5.76E-08	########	0.824	0.703	########	9
Tlr7	2.03E-10	########	0.485	0.195	3.40E-06	9
Arid1b	6.29E-08	########	0.529	0.3	########	9
mt-Atp8	5.07E-07	########	0.868	0.733	########	9
Bbx	3.51E-09	########	0.559	0.298	5.87E-05	9
Spin1	1.06E-08	########	0.559	0.317	########	9
Asah1	1.69E-06	########	0.779	0.585	########	9
Rbm15b	5.08E-12	########	0.426	0.159	8.49E-08	9
Hist3h2a	2.43E-08	########	0.397	0.17	########	9
Zfp280d	1.71E-10	########	0.441	0.177	2.86E-06	9
Rbx1	1.12E-08	########	0.971	0.827	########	9
Mpnd	5.64E-08	########	0.515	0.291	########	9
Rilpl1	2.03E-19	########	0.324	0.066	3.40E-15	9
Cox14	1.16E-07	########	0.853	0.662	########	9
Mlec	1.09E-08	########	0.75	0.47	########	9
Srrm2	1.08E-07	########	0.941	0.843	########	9
Hnrnpa0	6.53E-08	########	0.956	0.803	########	9
Mrps34	4.47E-08	########	0.647	0.39	########	9
Rsbn1l	6.35E-09	########	0.75	0.548	########	9
Cd4	3.30E-29	########	0.309	0.038	5.52E-25	9
Galnt11	7.78E-12	########	0.559	0.247	1.30E-07	9
Hadhb	4.79E-09	########	0.676	0.422	8.01E-05	9
Tia1	2.73E-06	########	0.5	0.318	########	9
Ifi47	1.02E-09	########	0.529	0.234	1.71E-05	9
Larp1	1.55E-07	########	0.618	0.406	########	9
Card6	1.07E-16	########	0.426	0.111	1.79E-12	9
Brd3	5.83E-07	########	0.574	0.366	########	9
Zfp638	2.64E-06	########	0.632	0.474	########	9

Sc5d	2.02E-10	########	0.441	0.181	3.38E-06	9
Emg1	3.20E-08	########	0.794	0.566	########	9
Gnb4	4.00E-12	########	0.324	0.092	6.70E-08	9
Hvcn1	1.97E-12	########	0.485	0.18	3.30E-08	9
Hnrnpa2b1	7.54E-08	########	0.985	0.899	########	9
R3hdm1	1.11E-08	########	0.647	0.396	########	9
Cct5	4.62E-07	########	0.882	0.689	########	9
Git2	1.99E-09	########	0.779	0.528	3.32E-05	9
mt-Nd1	1.03E-09	########	1	0.906	1.73E-05	9
Chd2	2.72E-06	########	0.691	0.502	########	9
Cyfip2	2.37E-07	########	0.603	0.32	########	9
Hnrnpl	2.25E-07	########	0.809	0.593	########	9
Rhoh	5.43E-11	########	0.559	0.229	9.08E-07	9
Ndufb11	4.79E-07	########	0.941	0.827	########	9
Rpl4	3.22E-08	########	1	0.885	########	9
Gpr137b-ps	2.22E-14	########	0.382	0.106	3.71E-10	9
Rufy1	2.95E-06	########	0.412	0.229	########	9
Tns1	4.46E-10	########	0.456	0.184	7.47E-06	9
Bcas2	1.28E-09	########	0.824	0.555	2.15E-05	9
Tcea1	2.91E-06	########	0.721	0.573	########	9
Zfp62	1.41E-07	########	0.471	0.248	########	9
Slfn8	7.94E-09	########	0.441	0.186	########	9
Pqlc3	1.31E-06	########	0.456	0.26	########	9
Rps9	1.29E-12	########	1	0.996	2.15E-08	9
4931406C0	2.62E-07	########	0.353	0.159	########	9
Eif3h	4.63E-07	########	0.971	0.803	########	9
Adk	5.33E-08	########	0.485	0.263	########	9
Eif3d	1.01E-06	########	0.735	0.523	########	9
Sys1	1.05E-06	########	0.853	0.686	########	9
Setd7	1.89E-08	########	0.485	0.242	########	9
Clic4	1.94E-06	########	0.706	0.417	########	9
Aaed1	2.32E-17	########	0.441	0.128	3.87E-13	9
Pigp	1.28E-08	########	0.515	0.264	########	9
Ubn2	1.16E-09	########	0.676	0.409	1.94E-05	9
Fgfr1op	2.49E-08	########	0.382	0.168	########	9
Rab3ip	1.98E-11	########	0.324	0.098	3.32E-07	9
Zfp260	2.91E-08	########	0.412	0.183	########	9
Nek6	4.47E-09	########	0.412	0.175	7.48E-05	9
Sigmar1	1.23E-06	########	0.441	0.241	########	9
Osgep	7.48E-07	########	0.603	0.402	########	9
2010107E0	2.07E-07	########	0.985	0.817	########	9
Mbd3	1.90E-06	########	0.618	0.403	########	9
Dhcr24	2.49E-10	########	0.309	0.098	4.16E-06	9
Rbm26	7.83E-09	########	0.647	0.401	########	9
Dr1	2.67E-06	########	0.529	0.341	########	9
Ahnak	1.32E-06	########	0.985	0.636	########	9
Coro7	2.11E-08	########	0.5	0.263	########	9
Atp6v0e	1.74E-07	########	1	0.885	########	9
Atp5sl	9.33E-14	########	0.456	0.153	1.56E-09	9
Erp29	1.52E-06	########	0.956	0.766	########	9
Gpr155	2.10E-31	########	0.338	0.045	3.51E-27	9
Pebp1	4.86E-08	########	0.882	0.619	########	9

Cox8a	6.23E-10	########	1	0.954	1.04E-05	9
Trim12a	8.49E-07	########	0.618	0.381	########	9
Ccdc47	1.64E-07	########	0.662	0.447	########	9
Snrpb	3.32E-07	########	0.926	0.741	########	9
Il21r	4.89E-09	########	0.412	0.164	8.19E-05	9
Dad1	1.21E-06	########	0.956	0.737	########	9
Slco3a1	2.71E-06	########	0.382	0.199	########	9
Smim24	1.75E-15	########	0.338	0.084	2.93E-11	9
Rab11fip2	2.08E-07	########	0.397	0.186	########	9
Atp5c1	1.99E-07	########	0.956	0.812	########	9
Gtpbp2	5.36E-09	########	0.559	0.299	8.96E-05	9
Matr3	7.51E-08	########	0.794	0.573	########	9
Minos1	1.69E-08	########	0.971	0.77	########	9
Rab3gap1	1.20E-06	########	0.471	0.276	########	9
Cstf2	2.75E-07	########	0.353	0.16	########	9
Kctd14	4.52E-62	########	0.338	0.023	7.57E-58	9
Abhd6	1.49E-50	########	0.338	0.029	2.50E-46	9
Acin1	1.16E-06	########	0.838	0.673	########	9
Alkbh1	6.90E-11	########	0.485	0.205	1.16E-06	9
Rps6	3.68E-07	########	1	0.938	########	9
Ccnt2	4.99E-08	########	0.529	0.289	########	9
Ppif	2.02E-14	########	0.368	0.105	3.38E-10	9
Ngfrap1	2.73E-06	########	0.471	0.258	########	9
Ndufa7	3.85E-07	########	0.941	0.842	########	9
Phlpp2	3.81E-15	########	0.309	0.073	6.37E-11	9
Rpl27	1.84E-09	########	0.985	0.876	3.08E-05	9
Tmem192	6.81E-08	########	0.485	0.263	########	9
P2ry13	9.86E-17	########	0.294	0.058	1.65E-12	9
Hpse	1.61E-10	########	0.456	0.184	2.70E-06	9
Mrfap1	9.63E-07	########	0.897	0.701	########	9
Dpy19l1	2.77E-08	########	0.5	0.263	########	9
Atraid	1.20E-07	########	0.706	0.48	########	9
Pnck	2.27E-112	########	0.235	0.005	3.80E-108	9
Rpl29	4.61E-07	########	1	0.91	########	9
Slc7a5	4.08E-09	########	0.353	0.136	6.83E-05	9
Cd72	3.24E-21	########	0.426	0.089	5.42E-17	9
5430427O1	5.51E-16	########	0.397	0.104	9.21E-12	9
Top2b	1.26E-06	########	0.75	0.574	########	9
Fyco1	6.07E-09	########	0.397	0.165	########	9
Pdia3	7.90E-07	########	0.971	0.753	########	9
Il12a	7.55E-22	########	0.191	0.021	1.26E-17	9
Atp1a1	1.58E-06	########	0.912	0.635	########	9
Tnni2	5.51E-25	########	0.382	0.068	9.22E-21	9
Lrba	4.75E-10	########	0.324	0.104	7.94E-06	9
Lrrk1	7.03E-08	########	0.397	0.183	########	9
Slc15a4	5.89E-08	########	0.441	0.216	########	9
Rpl17	2.62E-10	########	1	0.984	4.38E-06	9
Rpl34	3.63E-08	########	1	0.978	########	9
Adam19	4.96E-10	########	0.441	0.17	8.29E-06	9
Uqcr11	2.83E-06	########	0.985	0.787	########	9
Parp3	2.99E-06	########	0.382	0.199	########	9
Rpl24	3.97E-07	########	1	0.961	########	9

Ptprc	7.30E-07	########	0.985	0.801	########	9
mt-Cytb	4.53E-09	########	1	0.964	7.58E-05	9
Sdf4	1.08E-06	########	0.794	0.649	########	9
Phf6	2.00E-06	########	0.382	0.196	########	9
Gm21762	0	########	0.221	0	0	9
Mtss1	8.60E-10	########	0.529	0.226	1.44E-05	9
Kdm2b	3.34E-08	########	0.426	0.197	########	9
Blmh	1.28E-07	########	0.618	0.382	########	9
Zfp746	3.55E-07	########	0.338	0.147	########	9
Dnajc9	8.08E-07	########	0.544	0.295	########	9
Fus	8.43E-07	########	0.912	0.805	########	9
Morf4l1	2.27E-07	########	0.985	0.898	########	9
Mdm4	1.92E-06	########	0.515	0.317	########	9
Klhl42	9.65E-18	########	0.279	0.052	1.61E-13	9
Ercc5	3.19E-10	########	0.368	0.132	5.34E-06	9
Kbtbd11	3.98E-16	########	0.368	0.084	6.66E-12	9
Rfx7	2.70E-08	########	0.485	0.246	########	9
Coq2	1.39E-08	########	0.456	0.221	########	9
Scd2	1.70E-06	########	0.485	0.271	########	9
Robo3	4.03E-206	########	0.191	0.001	6.74E-202	9
Inip	1.28E-07	########	0.368	0.159	########	9
Phc1	1.61E-11	########	0.265	0.069	2.69E-07	9
Ankmy2	7.48E-18	########	0.382	0.094	1.25E-13	9
Mtmr1	7.55E-07	########	0.353	0.163	########	9
Lbh	8.40E-07	########	0.588	0.352	########	9
Sil1	1.19E-07	########	0.338	0.143	########	9
Zfp422	8.50E-08	########	0.368	0.163	########	9
Pycr2	1.78E-06	########	0.485	0.283	########	9
Ptch1	2.57E-10	########	0.25	0.066	4.30E-06	9
Ablim1	1.86E-06	########	0.441	0.201	########	9
Rpl9	4.35E-07	########	1	0.976	########	9
Timm10	1.95E-06	########	0.456	0.254	########	9
Srsf9	2.62E-06	########	0.765	0.605	########	9
Trappc4	1.88E-06	########	0.75	0.563	########	9
Polr3gl	1.01E-06	########	0.397	0.202	########	9
Atp5e	5.75E-07	########	0.985	0.94	########	9
Rps24	2.34E-12	########	1	0.974	3.91E-08	9
Calhm2	1.03E-07	########	0.324	0.126	########	9
Nxpe3	2.19E-25	########	0.338	0.052	3.67E-21	9
Atp5h	1.25E-06	########	1	0.911	########	9
Rpl27a	1.78E-06	########	1	0.979	########	9
Phc3	1.01E-07	########	0.441	0.209	########	9
Kdm4a	3.02E-07	########	0.397	0.19	########	9
Msh5	9.79E-32	########	0.279	0.031	1.64E-27	9
Cfap97	4.49E-07	########	0.397	0.192	########	9
Fau	2.53E-08	########	1	0.993	########	9
Ncf1	1.21E-07	########	0.853	0.512	########	9
Ghitm	1.12E-06	########	0.794	0.587	########	9
Rpl5	1.54E-07	########	1	0.906	########	9
Rps15a	1.40E-08	########	1	0.953	########	9
Ndufa13	3.79E-07	########	1	0.881	########	9
Plxdc1	4.67E-46	########	0.294	0.024	7.81E-42	9

Serbp1	2.11E-06	########	0.985	0.806	########	9
B3gnt8	2.99E-13	########	0.441	0.147	5.01E-09	9
Sgcb	1.03E-10	########	0.382	0.137	1.72E-06	9
Fbxl13	3.04E-98	########	0.25	0.007	5.09E-94	9
Siglecg	2.94E-16	########	0.382	0.093	4.92E-12	9
Slamf6	2.50E-17	########	0.471	0.117	4.19E-13	9
Pdia6	1.10E-07	########	0.912	0.608	########	9
C2cd5	1.68E-06	########	0.368	0.178	########	9
Marf1	4.64E-07	########	0.456	0.236	########	9
Stoml1	1.68E-17	########	0.353	0.081	2.81E-13	9
Abca5	6.99E-17	########	0.221	0.036	1.17E-12	9
BC147527	6.99E-29	########	0.279	0.033	1.17E-24	9
Nudt3	2.21E-07	########	0.559	0.336	########	9
Rtn4rl1	3.12E-21	########	0.235	0.032	5.22E-17	9
Dtx2	6.94E-07	########	0.353	0.163	########	9
Alyref2	9.09E-11	########	0.294	0.085	1.52E-06	9
Snx29	3.67E-13	########	0.353	0.097	6.14E-09	9
Gpcpd1	7.05E-08	########	0.721	0.445	########	9
Ifi44	2.05E-14	########	0.132	0.015	3.43E-10	9
Terf2	4.43E-10	########	0.397	0.152	7.41E-06	9
Asph	2.76E-06	########	0.441	0.241	########	9
Cyb561a3	5.46E-33	########	0.206	0.016	9.13E-29	9
H2-Oa	2.38E-18	########	0.309	0.056	3.98E-14	9
Pgam2	2.59E-53	########	0.132	0.004	4.33E-49	9
Hist3h2ba	5.16E-22	########	0.265	0.038	8.63E-18	9
Cluap1	7.74E-08	########	0.338	0.14	########	9
Mob3b	7.24E-12	########	0.294	0.079	1.21E-07	9
Slc27a1	8.08E-12	########	0.353	0.103	1.35E-07	9
Tmem106c	8.07E-09	########	0.324	0.118	########	9
Gm10138	3.44E-10	########	0.279	0.08	5.75E-06	9
Ppm1e	4.64E-82	########	0.235	0.008	7.76E-78	9
Fads3	9.61E-08	########	0.279	0.097	########	9
Cd37	1.18E-07	########	0.721	0.46	########	9
AY036118	2.64E-06	########	1	0.978	########	9
Rps29	1.40E-08	########	1	0.991	########	9
Ppp6r1	9.19E-07	########	0.603	0.385	########	9
Slco4a1	4.40E-19	########	0.206	0.028	7.36E-15	9
Tspyl4	1.70E-21	########	0.294	0.048	2.85E-17	9
Snrnp25	2.92E-06	########	0.5	0.301	########	9
Cds1	1.78E-14	########	0.294	0.068	2.98E-10	9
Cdk6	1.24E-06	########	0.456	0.229	########	9
Slf1	1.03E-07	########	0.338	0.139	########	9
Prkag2	3.28E-07	########	0.353	0.152	########	9
Snn	9.97E-20	########	0.294	0.05	1.67E-15	9
St8sia1	7.03E-40	########	0.265	0.021	1.18E-35	9
Tlr12	2.39E-76	########	0.206	0.006	3.99E-72	9
Cd8b1	4.19E-17	########	0.265	0.044	7.02E-13	9
Lynx1	9.55E-17	########	0.235	0.04	1.60E-12	9
Rubcn	1.16E-07	########	0.279	0.099	########	9
Sesn3	3.85E-08	########	0.397	0.163	########	9
Grm8	0	########	0.191	0	0	9
Gm5914	5.17E-11	########	0.265	0.068	8.66E-07	9

Pde4a	2.19E-10	########	0.25	0.067	3.67E-06	9
Zcchc18	1.29E-28	########	0.206	0.018	2.16E-24	9
Gbp4	7.10E-15	########	0.279	0.056	1.19E-10	9
C130026I2	8.02E-26	########	0.25	0.03	1.34E-21	9
Atp6ap2	1.43E-06	########	0.838	0.603	########	9
Matk	1.56E-07	########	0.294	0.108	########	9
Slc12a9	8.76E-07	########	0.382	0.179	########	9
Kdm1b	3.60E-08	########	0.221	0.064	########	9
Slc25a4	5.19E-08	########	0.868	0.517	########	9
Rnf167	2.83E-06	########	0.632	0.398	########	9
Cd244	1.39E-06	########	0.426	0.191	########	9
Gpr18	1.19E-13	########	0.309	0.071	2.00E-09	9
Dnmt3a	7.74E-07	########	0.426	0.208	########	9
Zfp865	6.54E-08	########	0.309	0.116	########	9
Itgb3	1.07E-08	########	0.235	0.067	########	9
Ikzf2	1.79E-23	########	0.309	0.047	2.99E-19	9
Zfp810	1.24E-16	########	0.191	0.027	2.07E-12	9
Trit1	1.05E-09	########	0.235	0.062	1.76E-05	9
Erbb3	6.31E-23	########	0.25	0.033	1.06E-18	9
1700097N0	2.87E-08	########	0.206	0.055	########	9
5031414D1	7.94E-11	########	0.235	0.056	1.33E-06	9
Cobll1	2.76E-10	########	0.235	0.058	4.61E-06	9
Tmem218	1.95E-06	########	0.235	0.086	########	9
N4bp3	1.13E-09	########	0.206	0.049	1.89E-05	9
S100pbp	3.14E-08	########	0.324	0.119	########	9
RP23-144N	0	########	0.191	0	0	9
Eef2k	4.98E-08	########	0.338	0.133	########	9
Rnf157	3.54E-14	########	0.25	0.051	5.93E-10	9
Fam234b	1.17E-07	########	0.162	0.039	########	9
H2-Ab1	1.03E-11	########	0.574	0.219	1.72E-07	9
Edrf1	2.50E-06	########	0.279	0.114	########	9
Dyrk3	5.94E-17	########	0.235	0.039	9.94E-13	9
Rhobtb2	6.61E-09	########	0.206	0.053	########	9
Nipal3	2.11E-07	########	0.235	0.074	########	9
Ctnnd2	1.81E-44	########	0.191	0.01	3.02E-40	9
Ralgps2	2.76E-09	########	0.426	0.164	4.62E-05	9
Adnp	1.68E-06	########	0.294	0.121	########	9
Wfs1	6.38E-07	########	0.191	0.058	########	9
Tmem194b	2.52E-10	########	0.206	0.046	4.22E-06	9
Tmigd3	4.59E-91	########	0.162	0.003	7.68E-87	9
Rpl12	2.06E-07	########	1	0.89	########	9
Apba1	2.81E-09	########	0.206	0.051	4.70E-05	9
Tespa1	5.04E-15	########	0.265	0.05	8.44E-11	9
Pecam1	5.48E-15	########	0.529	0.165	9.17E-11	9
Cadm1	7.74E-16	########	0.162	0.021	1.29E-11	9
Mcpt8	0	5.267602	0.762	0.01	0	10
Prss34	0	5.242186	0.413	0.001	0	10
Cpa3	0	4.873585	0.873	0.004	0	10
Ms4a2	0	3.760953	0.937	0.005	0	10
Ccl3	2.66E-60	3.604844	0.857	0.176	4.44E-56	10
Gata2	0	3.587461	0.968	0.021	0	10
Cyp11a1	0	3.497083	0.952	0.002	0	10

Hdc	7.79E-58	3.346844	1	0.271	1.30E-53	10
Cd200r3	0	3.322755	0.921	0.003	0	10
Rgs1	1.96E-32	3.319662	0.571	0.117	3.28E-28	10
Fcer1a	0	2.984404	0.825	0	0	10
Ifitm1	9.25E-35	2.815987	0.905	0.345	1.55E-30	10
Gm12840	7.44E-14	2.766842	0.413	0.13	1.25E-09	10
Csrp3	0	2.736655	0.73	0.001	0	10
Ccl4	1.06E-42	2.719972	0.746	0.156	1.78E-38	10
Hgf	3.61E-101	2.59987	0.794	0.085	6.04E-97	10
Txnip	3.20E-25	2.523973	0.921	0.646	5.35E-21	10
Tbc1d4	4.18E-96	2.50344	0.841	0.101	7.00E-92	10
Ccl9	8.06E-26	2.457034	0.889	0.44	1.35E-21	10
Ccl6	6.03E-16	2.456757	0.778	0.544	1.01E-11	10
Gpr183	1.46E-40	2.449171	0.81	0.226	2.44E-36	10
Cd7	9.38E-118	2.430724	0.714	0.054	1.57E-113	10
Sytl3	0	2.412607	0.81	0.023	0	10
Gzmb	2.19E-15	2.408249	0.159	0.019	3.67E-11	10
Nedd4	3.10E-42	2.357817	0.921	0.3	5.19E-38	10
Csf1	5.22E-75	2.357588	0.841	0.127	8.74E-71	10
Tmem71	9.09E-32	2.27984	0.794	0.284	1.52E-27	10
Csf2rb	6.32E-36	2.138069	0.968	0.525	1.06E-31	10
Lat2	5.00E-42	2.11797	0.905	0.314	8.37E-38	10
Slc18a2	0	2.07739	0.73	0.015	0	10
Lilr4b	8.92E-26	2.06304	0.873	0.462	1.49E-21	10
Cebpa	3.05E-34	2.038496	0.794	0.276	5.10E-30	10
Tax1bp1	3.66E-25	1.986136	0.968	0.85	6.12E-21	10
Tec	1.02E-39	1.949215	0.762	0.21	1.71E-35	10
Il6	5.07E-48	1.944726	0.635	0.1	8.48E-44	10
Il1rl1	1.75E-109	1.940421	0.746	0.061	2.92E-105	10
Jak2	2.01E-27	1.90028	0.794	0.345	3.37E-23	10
Il18r1	1.12E-65	1.899944	0.667	0.086	1.87E-61	10
Dapp1	1.90E-40	1.882338	0.905	0.35	3.18E-36	10
Padi2	4.36E-120	1.873336	0.81	0.072	7.30E-116	10
Tnfaip8	1.97E-26	1.847319	0.921	0.661	3.30E-22	10
Cyp4f18	1.63E-25	1.838615	0.825	0.379	2.72E-21	10
Furin	1.98E-13	1.823755	0.778	0.502	3.31E-09	10
Itga2b	1.40E-202	1.822476	0.778	0.036	2.35E-198	10
Cdh1	6.77E-235	1.816766	0.81	0.033	1.13E-230	10
Il18rap	5.56E-36	1.810352	0.794	0.235	9.31E-32	10
Neurl3	1.19E-21	1.805313	0.825	0.417	2.00E-17	10
Cd63	1.12E-30	1.788523	0.984	0.587	1.88E-26	10
Os9	3.97E-31	1.775815	0.921	0.567	6.65E-27	10
Mpc2	6.63E-19	1.769169	0.873	0.723	1.11E-14	10
Itk	6.05E-53	1.767804	0.619	0.086	1.01E-48	10
Gm20186	7.02E-11	1.747541	0.286	0.076	1.17E-06	10
Ikzf2	4.40E-153	1.733171	0.746	0.044	7.36E-149	10
Il4	0	1.658406	0.619	0.003	0	10
Csf2rb2	4.47E-30	1.650129	0.698	0.205	7.48E-26	10
Hist1h1c	1.03E-12	1.631298	0.683	0.432	1.72E-08	10
Klhl6	1.46E-27	1.614837	0.81	0.341	2.44E-23	10
Plac8	8.95E-19	1.61457	0.889	0.502	1.50E-14	10
Fxyd5	1.98E-26	1.61367	1	0.889	3.31E-22	10

Neat1	9.44E-13	1.606387	0.984	0.868	1.58E-08	10
Igf1r	6.88E-26	1.600434	0.81	0.389	1.15E-21	10
Serpinb1a	3.07E-08	1.583257	0.603	0.328	########	10
Rab44	2.16E-53	1.582133	0.746	0.143	3.61E-49	10
Gstm1	7.61E-21	1.552406	0.714	0.3	1.27E-16	10
Hacd4	1.70E-21	1.545676	0.857	0.492	2.85E-17	10
Itgb7	1.93E-29	1.541293	0.794	0.272	3.22E-25	10
Hmha1	5.18E-19	1.53912	0.873	0.564	8.67E-15	10
Cd9	1.27E-22	1.50963	0.952	0.547	2.12E-18	10
Stx3	1.48E-39	1.497967	0.667	0.148	2.47E-35	10
P2ry14	3.30E-126	1.483021	0.619	0.037	5.52E-122	10
Ncor1	3.02E-23	1.458788	0.952	0.769	5.05E-19	10
Rnf130	4.45E-26	1.458411	0.937	0.696	7.45E-22	10
Alox5	1.65E-32	1.454808	0.73	0.213	2.77E-28	10
Ctsg	1.92E-20	1.440509	0.333	0.058	3.21E-16	10
Fam174b	1.50E-151	1.434248	0.619	0.03	2.51E-147	10
Ets1	4.17E-23	1.421764	0.857	0.385	6.97E-19	10
Osm	2.06E-17	1.42147	0.571	0.204	3.44E-13	10
Hs3st1	1.67E-44	1.417483	0.381	0.039	2.79E-40	10
Plp2	1.23E-15	1.408671	0.794	0.549	2.06E-11	10
Prkacb	8.59E-24	1.4074	0.81	0.39	1.44E-19	10
Aqp9	2.27E-77	1.406794	0.587	0.055	3.80E-73	10
Syne1	1.81E-21	1.388312	0.698	0.243	3.04E-17	10
1-Sep	1.75E-26	1.382027	0.778	0.281	2.93E-22	10
Fyb	9.87E-21	1.381364	0.952	0.676	1.65E-16	10
Rgs2	5.84E-11	1.379366	0.762	0.541	9.77E-07	10
Plgrkt	4.24E-19	1.37578	0.794	0.498	7.10E-15	10
Cd69	8.47E-20	1.359369	0.556	0.161	1.42E-15	10
Herc1	1.64E-22	1.35321	0.825	0.459	2.74E-18	10
Rfc2	1.56E-21	1.352992	0.762	0.411	2.61E-17	10
Hist1h1e	5.11E-10	1.346717	0.667	0.404	8.55E-06	10
Pfkp	9.68E-14	1.332849	0.683	0.389	1.62E-09	10
St8sia4	6.08E-14	1.332267	0.714	0.384	1.02E-09	10
Fam107b	1.27E-18	1.329323	0.825	0.546	2.13E-14	10
Runx1	4.18E-20	1.323088	0.873	0.567	6.99E-16	10
Lmo4	5.19E-10	1.322329	0.762	0.58	8.68E-06	10
Atp1b1	6.50E-39	1.31083	0.556	0.093	1.09E-34	10
H3f3b	1.14E-16	1.294381	1	0.99	1.91E-12	10
Fbxo9	1.25E-20	1.289734	0.651	0.27	2.10E-16	10
Alox15	0	1.280058	0.524	0.004	0	10
Adk	4.33E-18	1.278104	0.619	0.262	7.25E-14	10
Emilin2	2.66E-18	1.275525	0.905	0.564	4.46E-14	10
Srgn	1.87E-19	1.267639	0.984	0.83	3.13E-15	10
Clint1	4.78E-18	1.266384	0.841	0.634	7.99E-14	10
Atp11b	2.73E-16	1.266233	0.825	0.565	4.57E-12	10
Il7r	1.26E-24	1.264964	0.571	0.132	2.11E-20	10
Slc7a8	3.59E-27	1.264548	0.651	0.181	6.00E-23	10
Myh9	1.23E-15	1.263077	0.921	0.841	2.06E-11	10
Alox5ap	3.08E-17	1.248279	0.937	0.69	5.16E-13	10
Fam65b	8.24E-21	1.241365	0.857	0.395	1.38E-16	10
Kcnj2	2.89E-34	1.235929	0.492	0.085	4.83E-30	10
Galnt6	2.27E-33	1.231127	0.667	0.168	3.80E-29	10

Id1	1.43E-15	1.223853	0.619	0.259	2.40E-11	10
Rbfa	3.19E-14	1.223798	0.73	0.51	5.33E-10	10
Cd200r1	1.79E-28	1.219344	0.556	0.132	3.00E-24	10
Gimap1	8.92E-32	1.204611	0.73	0.172	1.49E-27	10
Rin3	8.47E-21	1.202088	0.794	0.39	1.42E-16	10
H2-T23	2.35E-17	1.197929	0.937	0.683	3.93E-13	10
Oxr1	4.83E-12	1.187729	0.587	0.311	8.08E-08	10
Arhgap15	2.15E-18	1.186799	0.857	0.496	3.60E-14	10
Hcst	3.63E-14	1.184954	0.825	0.555	6.08E-10	10
Ssh2	6.75E-10	1.180891	0.778	0.607	1.13E-05	10
Rps6ka3	6.49E-09	1.174837	0.603	0.401	########	10
Mapk14	2.25E-12	1.173752	0.714	0.473	3.76E-08	10
Rgs18	1.19E-13	1.173661	0.603	0.292	2.00E-09	10
Hk2	1.23E-13	1.17121	0.698	0.424	2.06E-09	10
Gm20342	1.26E-21	1.171018	0.556	0.173	2.10E-17	10
Crlf3	4.49E-16	1.169248	0.794	0.512	7.51E-12	10
Srsf5	9.61E-17	1.169103	0.968	0.874	1.61E-12	10
Hip1r	1.79E-28	1.159497	0.571	0.139	2.99E-24	10
Spn	3.05E-20	1.157621	0.619	0.216	5.11E-16	10
L1cam	5.40E-38	1.153532	0.492	0.076	9.04E-34	10
AY036118	1.63E-10	1.150818	0.984	0.979	2.73E-06	10
Tgoln1	1.80E-10	1.141417	0.778	0.587	3.01E-06	10
Ept1	1.87E-17	1.139661	0.508	0.165	3.13E-13	10
Asph	1.28E-19	1.138694	0.619	0.24	2.14E-15	10
Stk17b	2.59E-11	1.136073	0.889	0.74	4.34E-07	10
Nt5c3	2.16E-15	1.132604	0.635	0.323	3.61E-11	10
Arhgef1	7.06E-15	1.132364	0.841	0.65	1.18E-10	10
Cnn2	8.45E-13	1.130549	0.825	0.647	1.41E-08	10
11-Sep	1.58E-16	1.129551	0.746	0.418	2.65E-12	10
Pyurf	5.85E-16	1.128163	0.54	0.213	9.79E-12	10
Prkd3	2.49E-15	1.125217	0.635	0.293	4.17E-11	10
2310001H1	9.37E-15	1.124249	0.683	0.333	1.57E-10	10
Ncf1	1.36E-11	1.122385	0.81	0.512	2.28E-07	10
Mapkapk3	9.39E-12	1.120991	0.571	0.305	1.57E-07	10
Satb1	1.25E-18	1.112533	0.667	0.242	2.09E-14	10
Bri3bp	7.28E-18	1.11114	0.651	0.276	1.22E-13	10
Slc41a3	1.48E-31	1.11067	0.54	0.116	2.47E-27	10
Grm6	0	1.105696	0.365	0.001	0	10
Rpl38	1.28E-20	1.104166	0.984	0.962	2.14E-16	10
Taldo1	4.26E-15	1.093524	0.921	0.883	7.13E-11	10
Lilrb4a	3.24E-12	1.091019	0.81	0.548	5.42E-08	10
Ptms	2.88E-12	1.084921	0.762	0.501	4.83E-08	10
Gcnt1	3.34E-23	1.083733	0.587	0.166	5.59E-19	10
Ptger3	0	1.081039	0.524	0.008	0	10
Fam46a	2.15E-10	1.079575	0.714	0.473	3.60E-06	10
Limd2	9.16E-11	1.079318	0.825	0.661	1.53E-06	10
Srpk1	2.16E-13	1.074826	0.698	0.431	3.62E-09	10
Paxbp1	3.76E-19	1.063345	0.683	0.303	6.30E-15	10
Trf	1.54E-15	1.055146	0.667	0.278	2.58E-11	10
Rsrp1	7.94E-17	1.04672	0.968	0.934	1.33E-12	10
Add1	7.75E-11	1.046166	0.698	0.516	1.30E-06	10
Ssbp2	8.80E-16	1.045376	0.54	0.2	1.47E-11	10

Ppp3ca	2.60E-13	1.041148	0.825	0.667	4.36E-09	10
Tmem64	1.81E-36	1.040824	0.587	0.113	3.02E-32	10
Ccng2	2.92E-12	1.03905	0.556	0.253	4.89E-08	10
Orai2	3.55E-13	1.037482	0.571	0.26	5.94E-09	10
Rb1cc1	1.35E-11	1.034933	0.762	0.6	2.27E-07	10
Swap70	3.33E-16	1.026586	0.619	0.26	5.58E-12	10
Leprotl1	2.11E-13	1.020066	0.825	0.624	3.52E-09	10
C3ar1	4.34E-18	1.019895	0.698	0.241	7.26E-14	10
Ankrd12	2.44E-09	1.017055	0.73	0.558	4.08E-05	10
Myo1d	1.98E-40	1.016195	0.54	0.089	3.31E-36	10
Itm2c	2.79E-12	1.015275	0.762	0.466	4.67E-08	10
Adgrg3	3.40E-24	1.010504	0.54	0.142	5.69E-20	10
Ier2	3.03E-07	1.008464	0.794	0.695	########	10
Ptpre	1.48E-10	1.004205	0.714	0.488	2.48E-06	10
Matk	1.06E-30	########	0.524	0.107	1.78E-26	10
Man2b1	8.23E-12	########	0.794	0.598	1.38E-07	10
Npl	1.36E-20	########	0.476	0.117	2.27E-16	10
Tagln2	6.08E-15	########	0.952	0.757	1.02E-10	10
Calm2	9.91E-14	########	0.952	0.896	1.66E-09	10
Samsn1	1.86E-19	########	0.905	0.503	3.11E-15	10
Mettl7a1	1.32E-28	########	0.54	0.123	2.20E-24	10
Eef2k	2.53E-31	########	0.571	0.131	4.22E-27	10
Rab37	9.49E-53	########	0.492	0.055	1.59E-48	10
Chst15	9.14E-27	########	0.54	0.125	1.53E-22	10
Il3ra	3.47E-13	########	0.508	0.216	5.81E-09	10
Akap13	1.42E-14	########	0.968	0.817	2.38E-10	10
Gpr34	2.35E-39	########	0.444	0.059	3.94E-35	10
Clec2d	7.45E-09	########	0.746	0.538	########	10
E2f8	2.34E-20	########	0.46	0.119	3.91E-16	10
Sub1	1.20E-09	########	0.968	0.911	2.01E-05	10
Gimap5	3.23E-31	########	0.524	0.097	5.41E-27	10
Atxn7	2.15E-12	########	0.556	0.255	3.60E-08	10
Acss2	9.67E-33	########	0.476	0.082	1.62E-28	10
Anxa6	7.11E-11	########	0.683	0.411	1.19E-06	10
Klf7	4.48E-08	########	0.714	0.519	########	10
Gmfg	1.76E-13	########	0.921	0.777	2.94E-09	10
Gpx4	4.95E-13	########	0.921	0.861	8.28E-09	10
Cst7	2.52E-31	########	0.492	0.088	4.21E-27	10
Nqo2	1.06E-06	########	0.429	0.243	########	10
Ero1l	4.62E-09	########	0.587	0.361	7.72E-05	10
Tsc22d3	1.10E-08	########	0.778	0.609	########	10
Cuedc1	7.96E-31	########	0.444	0.077	1.33E-26	10
Adgre5	1.70E-09	########	0.825	0.594	2.84E-05	10
Jak1	6.84E-10	########	0.857	0.824	1.14E-05	10
Rasal3	2.50E-19	########	0.492	0.133	4.18E-15	10
Emb	1.25E-10	########	0.921	0.738	2.08E-06	10
Smim3	2.66E-06	########	0.476	0.294	########	10
Nhsl2	4.88E-10	########	0.54	0.259	8.17E-06	10
Chn2	1.64E-48	########	0.429	0.046	2.75E-44	10
Cd84	4.69E-09	########	0.73	0.489	7.85E-05	10
Faah	3.75E-44	########	0.333	0.03	6.27E-40	10
Skap1	2.40E-25	########	0.492	0.096	4.01E-21	10

Irak3	1.72E-09	########	0.556	0.315	2.87E-05	10
Tprgl	1.22E-06	########	0.635	0.558	########	10
Osbpl3	1.24E-19	########	0.381	0.083	2.08E-15	10
D16Ertd472	8.40E-10	########	0.397	0.158	1.41E-05	10
Smap1	5.47E-08	########	0.714	0.605	########	10
Tln1	5.08E-12	########	0.905	0.867	8.50E-08	10
Cd244	1.08E-18	########	0.587	0.19	1.81E-14	10
Aff4	1.87E-08	########	0.667	0.485	########	10
Rps23	2.44E-17	########	1	0.977	4.09E-13	10
Serinc3	1.81E-12	########	0.921	0.828	3.02E-08	10
4833407H1	4.58E-21	########	0.444	0.099	7.66E-17	10
Tbc1d10c	3.20E-16	########	0.587	0.212	5.35E-12	10
Cetn2	1.14E-11	########	0.667	0.415	1.91E-07	10
Gabbr1	1.02E-20	########	0.381	0.08	1.71E-16	10
Tyrobp	5.91E-13	########	0.968	0.744	9.89E-09	10
Mbnl1	9.34E-11	########	0.905	0.802	1.56E-06	10
Myl12b	2.74E-12	########	0.952	0.871	4.58E-08	10
Trim12c	3.59E-09	########	0.603	0.363	6.00E-05	10
Pcmt1	7.07E-09	########	0.667	0.516	########	10
Phf20l1	1.51E-09	########	0.73	0.516	2.53E-05	10
Tmsb4x	1.55E-12	########	1	0.997	2.59E-08	10
Gna15	1.08E-14	########	0.46	0.159	1.80E-10	10
Nucb1	1.94E-07	########	0.619	0.501	########	10
Fut8	1.55E-07	########	0.397	0.199	########	10
Cnst	5.90E-20	########	0.476	0.129	9.88E-16	10
Add3	6.15E-10	########	0.73	0.542	1.03E-05	10
Mboat1	2.04E-31	########	0.429	0.07	3.41E-27	10
Whsc1l1	9.51E-09	########	0.841	0.722	########	10
Slc6a4	7.74E-144	########	0.222	0.003	1.29E-139	10
Atp2a3	1.68E-09	########	0.476	0.219	2.80E-05	10
Tacstd2	3.48E-18	########	0.508	0.152	5.82E-14	10
S100a10	1.03E-09	########	0.968	0.71	1.73E-05	10
Adora2b	1.89E-13	########	0.444	0.152	3.16E-09	10
Lgalsl	6.06E-24	########	0.46	0.101	1.01E-19	10
Fam204a	5.70E-07	########	0.54	0.352	########	10
Rbm39	1.60E-14	########	0.968	0.92	2.68E-10	10
Ubb	8.36E-10	########	0.984	0.984	1.40E-05	10
Arhgap4	2.37E-09	########	0.54	0.295	3.96E-05	10
1700020I14	5.95E-07	########	0.667	0.541	########	10
Sfxn1	1.64E-08	########	0.524	0.315	########	10
Fes	1.37E-07	########	0.683	0.499	########	10
Osbpl5	6.54E-37	########	0.413	0.056	1.09E-32	10
Heatr5a	2.07E-17	########	0.556	0.204	3.46E-13	10
Tespa1	1.22E-50	########	0.46	0.049	2.04E-46	10
Rps11	8.13E-16	########	0.984	0.966	1.36E-11	10
Adck4	2.64E-11	########	0.444	0.181	4.42E-07	10
Plek	1.24E-06	########	0.825	0.628	########	10
Plcb2	4.03E-14	########	0.476	0.172	6.75E-10	10
Lbr	6.66E-09	########	0.778	0.582	########	10
Slc2a3	9.97E-17	########	0.635	0.215	1.67E-12	10
Ankrd13a	7.29E-09	########	0.683	0.488	########	10
Btk	2.51E-07	########	0.524	0.327	########	10

Cd53	2.84E-10	########	0.921	0.775	4.75E-06	10
Unc13d	5.66E-17	########	0.524	0.173	9.47E-13	10
Wrn	1.23E-10	########	0.54	0.275	2.05E-06	10
Itga2	1.41E-56	########	0.349	0.025	2.36E-52	10
Glipr1	3.78E-08	########	0.619	0.391	########	10
Gab2	8.57E-15	########	0.524	0.195	1.43E-10	10
Ppp2r5a	2.09E-06	########	0.746	0.68	########	10
Inpp5b	1.24E-19	########	0.508	0.149	2.08E-15	10
Arpc5	6.40E-09	########	0.968	0.913	########	10
Vopp1	5.25E-21	########	0.397	0.085	8.78E-17	10
Stap1	2.89E-06	########	0.54	0.334	########	10
Rab4a	1.02E-37	########	0.333	0.035	1.71E-33	10
Nin	3.04E-07	########	0.635	0.461	########	10
Dock10	7.46E-11	########	0.698	0.443	1.25E-06	10
P2rx1	1.31E-44	########	0.381	0.039	2.19E-40	10
Rnf167	5.47E-07	########	0.571	0.399	########	10
Fli1	9.94E-09	########	0.683	0.477	########	10
Rac2	2.54E-12	########	0.952	0.739	4.26E-08	10
Ccnd2	2.02E-08	########	0.524	0.259	########	10
Rbl2	2.09E-11	########	0.524	0.24	3.49E-07	10
Ndrg1	1.26E-06	########	0.571	0.362	########	10
Vasp	1.11E-07	########	0.825	0.727	########	10
Acap1	3.51E-11	########	0.492	0.207	5.87E-07	10
Anp32a	2.69E-11	########	0.841	0.778	4.50E-07	10
Eif3j1	9.06E-09	########	0.698	0.528	########	10
Tecpr1	8.67E-10	########	0.429	0.18	1.45E-05	10
Scp2	1.27E-07	########	0.81	0.816	########	10
Ywhaz	1.33E-08	########	0.952	0.901	########	10
Zfp704	1.45E-17	########	0.413	0.108	2.42E-13	10
Ly6e	6.18E-09	########	0.905	0.815	########	10
Igsf6	8.06E-07	########	0.746	0.548	########	10
Rcn1	1.69E-13	########	0.492	0.173	2.83E-09	10
Arap3	3.00E-16	########	0.476	0.148	5.01E-12	10
Fam117a	2.65E-15	########	0.492	0.172	4.43E-11	10
Hvcn1	1.54E-11	########	0.46	0.18	2.58E-07	10
Atp8b4	2.07E-07	########	0.571	0.366	########	10
Adgrg1	2.70E-25	########	0.381	0.065	4.52E-21	10
Gnb4	1.11E-07	########	0.27	0.093	########	10
Camk1	2.92E-08	########	0.556	0.331	########	10
Tcp11l2	1.36E-06	########	0.619	0.442	########	10
Chd3	2.15E-08	########	0.54	0.288	########	10
Spry2	2.45E-06	########	0.302	0.128	########	10
Cd200r4	6.45E-45	########	0.333	0.029	1.08E-40	10
Stat4	1.54E-11	########	0.476	0.178	2.57E-07	10
P2rx4	3.62E-08	########	0.54	0.313	########	10
Ddx26b	1.88E-07	########	0.46	0.241	########	10
Tacc1	7.74E-07	########	0.667	0.528	########	10
Fam129a	8.78E-07	########	0.619	0.463	########	10
Nfe2	9.86E-09	########	0.476	0.224	########	10
P2ry10	1.26E-13	########	0.286	0.062	2.11E-09	10
Tmem156	2.90E-10	########	0.365	0.127	4.85E-06	10
Cd47	3.92E-08	########	0.873	0.9	########	10

Dpp4	8.99E-16	########	0.381	0.095	1.50E-11	10
Gltscr2	6.96E-07	########	0.794	0.708	########	10
Prkca	2.14E-14	########	0.444	0.138	3.58E-10	10
Tusc1	1.33E-06	########	0.333	0.152	########	10
Ngly1	7.44E-10	########	0.508	0.258	1.24E-05	10
Swt1	4.84E-08	########	0.54	0.321	########	10
Trpm4	2.19E-24	########	0.302	0.044	3.67E-20	10
Pfdn5	3.02E-12	########	0.937	0.939	5.06E-08	10
Rapsn	5.40E-36	########	0.365	0.044	9.03E-32	10
Rnase12	1.97E-268	########	0.317	0.003	3.30E-264	10
Sos2	5.79E-07	########	0.476	0.275	########	10
Inpp4b	5.52E-14	########	0.381	0.103	9.23E-10	10
Metap2	1.49E-06	########	0.714	0.638	########	10
Tnik	2.22E-32	########	0.444	0.068	3.71E-28	10
Cxcr4	1.51E-06	########	0.714	0.515	########	10
Sh3kbp1	7.20E-07	########	0.698	0.573	########	10
Vezf1	6.66E-07	########	0.556	0.39	########	10
Stk10	2.19E-09	########	0.651	0.38	3.67E-05	10
Ankrd44	1.21E-06	########	0.635	0.491	########	10
Pnpla7	3.51E-07	########	0.46	0.252	########	10
Rabac1	1.12E-08	########	0.857	0.827	########	10
Tbc1d9b	3.27E-09	########	0.46	0.221	5.46E-05	10
Flnb	4.21E-08	########	0.349	0.138	########	10
Ubn1	3.05E-07	########	0.651	0.517	########	10
Rpia	3.47E-11	########	0.492	0.213	5.81E-07	10
Pecam1	2.82E-17	########	0.54	0.165	4.72E-13	10
Btnl9	9.17E-44	########	0.349	0.033	1.53E-39	10
Klhl24	2.58E-06	########	0.508	0.309	########	10
Mob3a	1.94E-06	########	0.492	0.296	########	10
Fau	8.21E-15	########	1	0.993	1.37E-10	10
Ptpn7	2.18E-06	########	0.413	0.215	########	10
Zc3hav1	3.90E-07	########	0.698	0.556	########	10
Ptp4a2	1.29E-07	########	0.825	0.793	########	10
Atp1a3	3.60E-11	########	0.508	0.217	6.03E-07	10
Mir142hg	1.54E-09	########	0.429	0.174	2.57E-05	10
Tmem123	4.38E-10	########	0.698	0.461	7.33E-06	10
Srrm2	1.17E-09	########	0.905	0.843	1.95E-05	10
Uqcrh	3.98E-13	########	0.968	0.932	6.66E-09	10
Rpl39	1.81E-11	########	0.984	0.96	3.02E-07	10
Ptpn18	2.38E-08	########	0.921	0.765	########	10
Ikbkb	6.38E-08	########	0.714	0.507	########	10
Clec2i	2.69E-10	########	0.381	0.131	4.49E-06	10
Ndufa6	7.58E-09	########	0.889	0.82	########	10
Cdyl2	5.19E-09	########	0.254	0.074	8.68E-05	10
S1pr4	1.15E-09	########	0.492	0.223	1.93E-05	10
Esyt1	1.13E-07	########	0.651	0.454	########	10
Dctn6	1.34E-07	########	0.54	0.334	########	10
Ogt	2.02E-06	########	0.683	0.545	########	10
Xpc	1.91E-06	########	0.476	0.259	########	10
Rnf43	9.13E-77	########	0.27	0.011	1.53E-72	10
Pdzd4	1.56E-40	########	0.302	0.027	2.61E-36	10
AI467606	9.38E-08	########	0.54	0.311	########	10

P2ry1	8.11E-20	########	0.302	0.053	1.36E-15	10
Tnfaip8l2	1.23E-07	########	0.698	0.482	########	10
Hectd1	1.17E-06	########	0.73	0.64	########	10
Ep300	8.87E-07	########	0.714	0.571	########	10
Traf3ip3	2.08E-07	########	0.556	0.317	########	10
Gse1	9.81E-09	########	0.413	0.179	########	10
1810058I24	2.61E-06	########	0.794	0.736	########	10
Lcp1	4.95E-10	########	0.984	0.809	8.28E-06	10
Poln	8.84E-126	########	0.286	0.007	1.48E-121	10
Wdr95	3.05E-79	########	0.238	0.008	5.10E-75	10
Prrc2c	1.02E-08	########	0.889	0.79	########	10
Rpl37	6.69E-11	########	0.984	0.982	1.12E-06	10
Tal1	2.14E-47	########	0.349	0.031	3.58E-43	10
Malat1	7.97E-11	########	1	0.993	1.33E-06	10
Rps27	8.40E-13	########	0.968	0.977	1.41E-08	10
Fer	2.84E-08	########	0.317	0.119	########	10
Hmgb1	7.62E-08	########	0.905	0.881	########	10
Gsr	1.54E-07	########	0.889	0.611	########	10
Def6	1.68E-06	########	0.492	0.302	########	10
Il15	1.66E-12	########	0.365	0.107	2.77E-08	10
Klf5	6.61E-07	########	0.27	0.099	########	10
Itgb1	4.83E-07	########	0.857	0.649	########	10
Iqgap1	1.11E-08	########	0.984	0.913	########	10
Rpl37a	4.45E-12	########	1	0.983	7.44E-08	10
Zcchc7	1.14E-06	########	0.635	0.436	########	10
Rora	2.43E-06	########	0.317	0.14	########	10
Rab19	7.03E-38	########	0.349	0.037	1.18E-33	10
Supt3	7.14E-07	########	0.333	0.147	########	10
Pmm2	4.90E-09	########	0.524	0.279	8.20E-05	10
Prkcq	3.15E-21	########	0.397	0.075	5.26E-17	10
Sirt3	5.00E-13	########	0.317	0.084	8.36E-09	10
Prr13	2.56E-08	########	0.873	0.706	########	10
Inf2	2.44E-07	########	0.365	0.164	########	10
Hist1h2ap	1.43E-06	########	0.476	0.248	########	10
Tomm7	5.00E-08	########	0.889	0.871	########	10
Pak2	2.59E-06	########	0.841	0.817	########	10
Rpl10	2.06E-07	########	0.968	0.952	########	10
Tulp3	1.32E-13	########	0.27	0.059	2.21E-09	10
8-Mar	1.43E-08	########	0.397	0.17	########	10
Pdcd1lg2	1.42E-42	########	0.254	0.017	2.38E-38	10
Rpl34	2.04E-11	########	0.984	0.979	3.41E-07	10
Gm26541	6.86E-11	########	0.27	0.071	1.15E-06	10
Abca2	6.32E-08	########	0.254	0.081	########	10
Susd1	3.87E-10	########	0.27	0.075	6.48E-06	10
Grina	9.27E-09	########	0.857	0.603	########	10
Cxcr2	1.77E-15	########	0.698	0.237	2.96E-11	10
H2-Oa	2.11E-25	########	0.365	0.056	3.53E-21	10
Cd226	3.44E-44	########	0.381	0.037	5.76E-40	10
Rpl27	3.68E-07	########	0.937	0.876	########	10
Slc24a3	1.98E-24	########	0.19	0.018	3.31E-20	10
Sytl1	1.33E-22	########	0.302	0.047	2.23E-18	10
Fut7	1.60E-26	########	0.286	0.036	2.67E-22	10

Rpl36	1.50E-09	########	0.984	0.907	2.52E-05	10
Ctse	1.16E-14	########	0.27	0.054	1.93E-10	10
Selplg	6.33E-08	########	0.873	0.638	########	10
Angpt1	7.52E-19	########	0.286	0.049	1.26E-14	10
Tmcc3	5.07E-07	########	0.27	0.097	########	10
Trmt112	2.04E-07	########	0.762	0.623	########	10
Samd13	2.11E-132	########	0.238	0.004	3.52E-128	10
H2afj	4.58E-07	########	0.873	0.853	########	10
Runx2	7.61E-08	########	0.349	0.136	########	10
Gm21994	1.33E-37	########	0.222	0.015	2.22E-33	10
RP23-152F	0	########	0.222	0	0	10
Hist1h2be	4.30E-08	########	0.349	0.135	########	10
Acvr1c	4.60E-230	########	0.19	0.001	7.70E-226	10
Far2	1.07E-45	########	0.302	0.024	1.79E-41	10
Pcmtd2	8.86E-08	########	0.46	0.224	########	10
Chst13	1.59E-27	########	0.333	0.048	2.65E-23	10
Gimap9	4.50E-10	########	0.444	0.161	7.53E-06	10
Jakmip1	1.31E-13	########	0.302	0.067	2.19E-09	10
Zfp120	2.30E-06	########	0.349	0.162	########	10
Naprt	3.20E-08	########	0.286	0.099	########	10
Gm26526	6.24E-07	########	0.381	0.173	########	10
Trim39	2.41E-08	########	0.302	0.104	########	10
Eif1	1.35E-10	########	1	0.99	2.26E-06	10
Pafah2	3.18E-13	########	0.254	0.054	5.31E-09	10
Gm26809	3.50E-15	########	0.175	0.023	5.86E-11	10
Rpl23a	7.52E-08	########	0.968	0.911	########	10
Rapgef3	4.19E-25	########	0.238	0.027	7.01E-21	10
Nipal3	3.48E-10	########	0.27	0.074	5.82E-06	10
Htr1b	5.14E-92	########	0.206	0.005	8.61E-88	10
Ino80dos	8.27E-18	########	0.286	0.051	1.38E-13	10
Rpl15	2.82E-06	########	0.937	0.828	########	10
Itm2b	3.01E-10	########	1	0.968	5.04E-06	10
Ddx25	6.33E-49	########	0.19	0.008	1.06E-44	10
Smim24	1.45E-12	########	0.317	0.084	2.42E-08	10
Lrba	1.87E-09	########	0.317	0.105	3.14E-05	10
Treml2	3.64E-07	########	0.492	0.26	########	10
Pabpc1	2.88E-06	########	1	0.925	########	10
Mirt1	1.62E-06	########	0.397	0.184	########	10
Ppcdc	6.98E-08	########	0.286	0.099	########	10
Lag3	4.06E-39	########	0.222	0.015	6.80E-35	10
Sytl2	3.11E-13	########	0.19	0.031	5.20E-09	10
Gm28187	1.67E-24	########	0.175	0.015	2.79E-20	10
Adora3	2.90E-07	########	0.159	0.038	########	10
D430036J1	1.14E-304	########	0.159	0	1.90E-300	10
Dgkg	5.29E-07	########	0.333	0.14	########	10
Rom1	1.75E-09	########	0.254	0.071	2.93E-05	10
Kcne3	3.82E-38	########	0.222	0.015	6.39E-34	10
Rnf125	5.86E-08	########	0.317	0.113	########	10
Cables1	3.29E-10	########	0.19	0.04	5.51E-06	10
C530050E1	2.41E-07	########	0.222	0.066	########	10
Rgs12	4.82E-08	########	0.238	0.071	########	10
Prkag2	1.97E-06	########	0.333	0.152	########	10

Lat	6.00E-12	########	0.333	0.084	1.00E-07	10
Rpl17	5.79E-08	########	1	0.984	########	10
Pkp3	2.27E-08	########	0.349	0.12	########	10
Alox8	7.93E-141	########	0.175	0.002	1.33E-136	10
Rps6ka5	1.71E-07	########	0.27	0.093	########	10
Slain1	1.41E-19	########	0.206	0.025	2.36E-15	10
Arhgap6	1.87E-10	########	0.254	0.064	3.12E-06	10
Acss1	2.09E-09	########	0.238	0.061	3.50E-05	10
Siglecf	1.93E-21	########	0.206	0.023	3.22E-17	10
Ccdc62	6.24E-14	########	0.159	0.021	1.04E-09	10
Enpp4	5.23E-08	########	0.238	0.071	########	10
Otub2	3.59E-38	########	0.159	0.008	6.01E-34	10
Rps24	1.83E-08	########	0.984	0.974	########	10
Dtnb	1.22E-08	########	0.254	0.075	########	10
Itgb3	2.48E-07	########	0.222	0.067	########	10
C030034L1	2.13E-38	########	0.175	0.009	3.57E-34	10
Fam189b	2.72E-06	########	0.317	0.127	########	10
4833403J1	6.65E-08	########	0.127	0.023	########	10
Gm16124	6.55E-07	########	0.175	0.046	########	10
Gpsm1	3.00E-08	########	0.19	0.047	########	10
Plekhg5	2.00E-07	########	0.175	0.044	########	10
St8sia6	3.01E-22	########	0.222	0.025	5.03E-18	10
St6galnac3	8.80E-22	########	0.19	0.019	1.47E-17	10
Crip1	5.40E-07	########	0.968	0.72	########	10
Tet1	7.99E-24	########	0.206	0.021	1.34E-19	10
Kcnq5	8.93E-17	########	0.111	0.009	1.49E-12	10
Ccr3	3.76E-31	########	0.175	0.012	6.29E-27	10
Qrfp	1.92E-20	########	0.111	0.007	3.21E-16	10
Gnaz	3.25E-16	########	0.143	0.015	5.43E-12	10
Jazf1	3.71E-18	########	0.127	0.011	6.20E-14	10
Sdsl	1.30E-21	########	0.111	0.007	2.18E-17	10
Tctn1	6.31E-09	########	0.175	0.038	########	10
Agap2	3.13E-08	########	0.175	0.04	########	10
Gm37233	1.76E-07	########	0.175	0.043	########	10

Table S2 Subcluster-specific DEGs across day 3 neutrophil subtypes.

gene	p_val	avg_log2F(	pct.1	pct.2	p_val_adj	cluster
Hist1h1b	2.65E-234	4.50558	0.699	0.045	4.44E-230	1
Prtn3	2.66E-118	3.87632	0.624	0.128	4.45E-114	1
Hist1h2ap	2.12E-131	3.841791	0.755	0.252	3.55E-127	1
Elane	5.29E-108	3.793017	0.417	0.041	8.86E-104	1
Ptma	1.07E-184	3.081831	0.947	0.391	1.79E-180	1
Hist1h2ae	5.46E-211	2.970677	0.687	0.06	9.13E-207	1
Hist1h4d	2.07E-115	2.792333	0.742	0.279	3.46E-111	1
Fcnb	2.53E-130	2.653426	0.699	0.167	4.24E-126	1
Stmn1	2.09E-202	2.651237	0.765	0.114	3.50E-198	1
Rps18	8.89E-146	2.574983	0.942	0.615	1.49E-141	1
Top2a	2.53E-246	2.563817	0.742	0.049	4.23E-242	1
Ppia	2.26E-158	2.548636	0.942	0.578	3.79E-154	1
Rps2	1.11E-132	2.543372	0.957	0.7	1.86E-128	1
Hist1h1e	2.14E-74	2.543144	0.76	0.491	3.58E-70	1
Rpl3	1.95E-162	2.426982	0.922	0.389	3.26E-158	1
Rps20	2.78E-146	2.416921	0.952	0.713	4.65E-142	1
Rpsa	1.85E-144	2.405808	0.957	0.692	3.10E-140	1
Rpl10a	2.13E-146	2.363986	0.909	0.431	3.56E-142	1
Rpl32	3.16E-134	2.36092	0.942	0.734	5.29E-130	1
Hist1h3c	6.70E-244	2.359348	0.677	0.028	1.12E-239	1
Mki67	2.95E-194	2.257244	0.755	0.107	4.94E-190	1
2810417H1	9.69E-234	2.245218	0.742	0.063	1.62E-229	1
Rpl12	1.06E-137	2.21826	0.939	0.51	1.78E-133	1
Rps8	3.84E-142	2.186664	0.977	0.836	6.43E-138	1
Hist2h2ac	4.44E-225	2.182909	0.654	0.034	7.43E-221	1
Rpl13	1.12E-133	2.159235	0.962	0.836	1.88E-129	1
Rpl36a	5.45E-144	2.146855	0.907	0.439	9.11E-140	1
Rps19	1.27E-137	2.143604	0.957	0.754	2.12E-133	1
H2afz	3.65E-115	2.135052	0.934	0.836	6.11E-111	1
Rps5	1.33E-140	2.129312	0.949	0.728	2.22E-136	1
Anp32b	1.14E-165	2.12807	0.876	0.321	1.91E-161	1
Rpl14	5.39E-138	2.083149	0.939	0.507	9.02E-134	1
mt-Nd1	7.35E-102	2.078837	0.937	0.575	1.23E-97	1
Rps28	3.10E-139	2.070918	0.952	0.73	5.19E-135	1
Tubb5	5.82E-211	2.069376	0.828	0.14	9.73E-207	1
Rps17	2.34E-146	2.020277	0.939	0.548	3.91E-142	1
Rpl36	1.10E-136	2.000217	0.944	0.602	1.84E-132	1
Rpl35	9.33E-134	1.990394	0.932	0.611	1.56E-129	1
mt-Nd2	1.76E-85	1.968173	0.929	0.501	2.95E-81	1
Tuba1b	3.94E-252	1.957994	0.836	0.083	6.59E-248	1
Dek	3.12E-187	1.946857	0.861	0.216	5.21E-183	1
Rps4x	1.90E-127	1.929196	0.937	0.741	3.18E-123	1
Hist1h2ab	9.36E-258	1.905747	0.659	0.013	1.57E-253	1
Rps15a	8.96E-131	1.902812	0.952	0.814	1.50E-126	1
Hmgb1	2.12E-132	1.902211	0.932	0.685	3.54E-128	1
Rplp1	1.99E-112	1.899059	0.972	0.891	3.32E-108	1
Rpl8	8.83E-127	1.898827	0.955	0.786	1.48E-122	1
Chil3	2.92E-70	1.884947	0.808	0.449	4.88E-66	1
Rpl39	6.14E-128	1.882426	0.947	0.857	1.03E-123	1

Rpl36al	1.01E-157	1.870321	0.904	0.335	1.70E-153	1
Rps26	3.97E-127	1.862546	0.952	0.786	6.65E-123	1
Rpl19	2.59E-132	1.853963	0.965	0.851	4.33E-128	1
Hmgn2	8.69E-63	1.852928	0.851	0.756	1.45E-58	1
mt-Cytb	3.87E-88	1.835264	0.965	0.854	6.47E-84	1
Hist1h4h	1.39E-205	1.834018	0.694	0.066	2.33E-201	1
Rpl28	4.72E-138	1.825272	0.967	0.861	7.90E-134	1
Rpl22	1.16E-123	1.813974	0.952	0.659	1.94E-119	1
Rps3a1	1.45E-122	1.813601	0.96	0.871	2.43E-118	1
Rps23	5.56E-125	1.813285	0.975	0.912	9.30E-121	1
Rpl11	1.12E-118	1.79974	0.952	0.812	1.87E-114	1
mt-Atp6	4.03E-106	1.795911	0.97	0.966	6.74E-102	1
Rplp0	7.01E-112	1.772561	0.96	0.871	1.17E-107	1
Rps7	7.99E-126	1.771693	0.949	0.817	1.34E-121	1
Mt1	7.28E-115	1.760761	0.573	0.086	1.22E-110	1
Smc4	6.04E-144	1.750738	0.803	0.252	1.01E-139	1
Rpl18	2.74E-122	1.745209	0.957	0.837	4.59E-118	1
Rpl6	5.22E-127	1.744547	0.942	0.771	8.73E-123	1
Mpo	1.99E-81	1.740549	0.301	0.024	3.34E-77	1
Ran	2.74E-175	1.730033	0.859	0.232	4.59E-171	1
Ube2c	4.57E-179	1.704174	0.682	0.075	7.65E-175	1
Rpl38	7.43E-134	1.695957	0.957	0.845	1.24E-129	1
Dbi	1.50E-162	1.690814	0.866	0.254	2.51E-158	1
Ms4a3	9.73E-153	1.67829	0.639	0.087	1.63E-148	1
Rps6	7.07E-119	1.670227	0.952	0.749	1.18E-114	1
Nusap1	6.37E-228	1.662266	0.654	0.027	1.07E-223	1
Crip1	4.23E-42	1.657948	0.308	0.07	7.08E-38	1
Rps12	4.97E-124	1.657014	0.982	0.944	8.32E-120	1
mt-Co3	5.54E-114	1.655441	0.982	0.973	9.27E-110	1
Rpl22l1	4.76E-156	1.637671	0.899	0.294	7.97E-152	1
Rpl23	1.12E-127	1.634861	0.982	0.945	1.87E-123	1
Npm1	7.40E-170	1.629213	0.886	0.218	1.24E-165	1
Rps15	1.07E-126	1.619784	0.942	0.728	1.79E-122	1
Rpl26	2.25E-117	1.613205	0.97	0.88	3.77E-113	1
Gnb2l1	8.67E-111	1.611189	0.937	0.661	1.45E-106	1
H2afx	6.59E-227	1.595553	0.73	0.055	1.10E-222	1
H2afv	7.56E-134	1.594277	0.828	0.331	1.27E-129	1
Rplp2	5.53E-135	1.59358	0.98	0.877	9.25E-131	1
Hint1	4.50E-187	1.593518	0.909	0.225	7.53E-183	1
Rrm2	2.63E-258	1.593293	0.715	0.03	4.39E-254	1
Rpl21	1.55E-124	1.592439	0.949	0.817	2.60E-120	1
Birc5	7.53E-215	1.582906	0.725	0.062	1.26E-210	1
Rpl27a	5.39E-99	1.579471	0.972	0.919	9.02E-95	1
Rps29	1.15E-138	1.578173	0.987	0.975	1.92E-134	1
Smc2	4.28E-248	1.571226	0.74	0.041	7.16E-244	1
Lgals1	2.56E-56	1.566444	0.366	0.071	4.28E-52	1
Rps24	1.91E-122	1.565209	0.962	0.894	3.19E-118	1
Rps21	1.76E-117	1.561492	0.972	0.902	2.94E-113	1
Rps3	3.50E-122	1.553235	0.967	0.89	5.86E-118	1
Rps11	3.21E-115	1.550252	0.965	0.869	5.37E-111	1
Rpl5	3.48E-115	1.547829	0.939	0.587	5.82E-111	1
mt-Nd3	8.65E-102	1.523584	0.876	0.343	1.45E-97	1

Ube2s	4.67E-124	1.516962	0.871	0.395	7.82E-120	1
Ndufa4	1.39E-132	1.512892	0.891	0.46	2.32E-128	1
Rpl35a	5.17E-136	1.511648	0.972	0.93	8.66E-132	1
Atp5g2	1.16E-149	1.511464	0.886	0.345	1.94E-145	1
Plac8	5.34E-40	1.507213	0.826	0.518	8.94E-36	1
Rpl41	8.79E-107	1.504071	0.982	0.973	1.47E-102	1
mt-Nd4	8.71E-70	1.484828	0.967	0.837	1.46E-65	1
Rpl13a	1.81E-102	1.483884	0.965	0.846	3.03E-98	1
Naca	4.13E-118	1.472454	0.927	0.645	6.91E-114	1
Eef1a1	6.83E-106	1.46848	0.98	0.946	1.14E-101	1
Snrpe	1.02E-161	1.459128	0.876	0.291	1.71E-157	1
Serbp1	8.22E-150	1.457297	0.889	0.354	1.38E-145	1
Hist1h1d	2.02E-233	1.448321	0.679	0.035	3.37E-229	1
Snrpd1	1.32E-244	1.443079	0.808	0.081	2.21E-240	1
Sumo2	1.85E-127	1.431755	0.866	0.435	3.09E-123	1
Nucks1	9.68E-197	1.428929	0.803	0.12	1.62E-192	1
Prc1	3.71E-249	1.424138	0.687	0.023	6.21E-245	1
Rpl37a	4.16E-103	1.419009	0.965	0.936	6.96E-99	1
Dut	1.70E-244	1.418401	0.662	0.02	2.85E-240	1
Rpl18a	4.20E-116	1.418242	0.977	0.929	7.02E-112	1
Rps10	7.48E-113	1.408967	0.97	0.947	1.25E-108	1
Rpl7a	5.22E-102	1.408632	0.929	0.697	8.73E-98	1
S100a4	2.18E-29	1.408513	0.197	0.041	3.65E-25	1
Cdca8	1.88E-222	1.399727	0.707	0.046	3.15E-218	1
Rpl29	5.16E-106	1.392792	0.919	0.699	8.63E-102	1
Serpinb1a	5.17E-60	1.375249	0.783	0.45	8.65E-56	1
Rps16	5.78E-121	1.372585	0.977	0.973	9.68E-117	1
Ctsg	1.19E-82	1.367839	0.295	0.02	1.99E-78	1
Rpl24	6.10E-92	1.362066	0.947	0.845	1.02E-87	1
Rpl30	2.86E-89	1.361912	0.965	0.925	4.79E-85	1
mt-Co2	6.31E-90	1.361597	0.977	0.965	1.06E-85	1
Rpl15	3.30E-121	1.35424	0.889	0.404	5.53E-117	1
Hnrnpa3	1.01E-113	1.347255	0.896	0.522	1.69E-109	1
Pgp	1.69E-126	1.327103	0.783	0.273	2.82E-122	1
Rpl34	6.94E-105	1.323667	0.975	0.932	1.16E-100	1
Set	6.88E-174	1.308389	0.856	0.194	1.15E-169	1
Nap1l1	3.73E-151	1.307473	0.841	0.251	6.24E-147	1
Rpl9	2.71E-94	1.307283	0.97	0.936	4.54E-90	1
Hmgb2	1.83E-53	1.306734	0.96	0.954	3.07E-49	1
Hist1h3e	2.18E-247	1.306413	0.652	0.016	3.65E-243	1
Rpl31	1.64E-103	1.302858	0.922	0.644	2.75E-99	1
Rpl10	4.38E-77	1.302368	0.949	0.844	7.33E-73	1
Cebpe	8.66E-65	1.299082	0.71	0.377	1.45E-60	1
Tmem256	8.81E-115	1.298524	0.826	0.316	1.47E-110	1
Cdk1	3.02E-256	1.295485	0.732	0.032	5.06E-252	1
Rps13	1.18E-99	1.294694	0.97	0.92	1.98E-95	1
Cbx3	4.18E-161	1.289934	0.826	0.221	6.99E-157	1
Rpl4	3.16E-100	1.289351	0.907	0.563	5.28E-96	1
Tuba4a	9.78E-83	1.283074	0.831	0.519	1.64E-78	1
Eef1b2	2.28E-74	1.276238	0.929	0.739	3.82E-70	1
Rpl7	8.16E-100	1.254477	0.949	0.876	1.36E-95	1
Erh	3.82E-173	1.254167	0.861	0.205	6.39E-169	1

Hist1h2bj	5.92E-232	1.252576	0.629	0.02	9.90E-228	1
Rps14	2.18E-84	1.237659	0.975	0.947	3.65E-80	1
Alyref	1.61E-192	1.231717	0.801	0.133	2.70E-188	1
Srsf3	2.84E-120	1.230117	0.886	0.42	4.74E-116	1
Tagln2	1.71E-116	1.222192	0.896	0.362	2.86E-112	1
Ybx1	4.57E-108	1.220293	0.934	0.721	7.64E-104	1
Hnrnpab	4.18E-165	1.217503	0.843	0.197	7.00E-161	1
Atad2	3.85E-227	1.215538	0.692	0.044	6.45E-223	1
Ranbp1	3.72E-243	1.215496	0.811	0.073	6.23E-239	1
Camp	4.31E-34	1.208849	0.73	0.508	7.22E-30	1
Tmed3	1.16E-116	1.206692	0.79	0.279	1.94E-112	1
Anp32e	1.97E-206	1.206469	0.778	0.096	3.30E-202	1
Snrpf	1.86E-212	1.205777	0.843	0.123	3.11E-208	1
Nkg7	1.00E-150	1.205457	0.543	0.046	1.68E-146	1
Rps25	1.86E-91	1.200052	0.955	0.888	3.12E-87	1
Uqcrb	9.29E-114	1.190907	0.891	0.429	1.55E-109	1
Hsp90ab1	6.58E-52	1.189122	0.924	0.656	1.10E-47	1
Rpl23a	4.31E-102	1.179361	0.927	0.691	7.21E-98	1
Kif11	2.54E-235	1.171193	0.669	0.027	4.25E-231	1
Hnrnpa2b1	8.39E-102	1.170107	0.927	0.71	1.40E-97	1
Hnrnpd	6.45E-111	1.166746	0.869	0.445	1.08E-106	1
Eif5a	1.02E-96	1.158256	0.922	0.693	1.71E-92	1
Cox5a	3.46E-107	1.15524	0.899	0.591	5.79E-103	1
Ssr4	2.30E-84	1.154871	0.841	0.389	3.85E-80	1
Rbm3	2.07E-98	1.153441	0.932	0.686	3.46E-94	1
Atp5g1	1.31E-131	1.152026	0.838	0.233	2.20E-127	1
Atpif1	2.50E-96	1.150898	0.891	0.507	4.18E-92	1
S100a10	7.09E-33	1.148716	0.225	0.048	1.19E-28	1
Lig1	3.50E-246	1.146384	0.707	0.033	5.85E-242	1
Rps27l	2.84E-130	1.143566	0.881	0.312	4.75E-126	1
Etfb	4.42E-135	1.141531	0.821	0.236	7.39E-131	1
Tubb4b	7.89E-92	1.140475	0.833	0.401	1.32E-87	1
Snrpg	2.99E-162	1.133771	0.848	0.216	5.01E-158	1
1810037I17	6.19E-82	1.131884	0.894	0.662	1.04E-77	1
Gmnn	5.75E-234	1.131735	0.753	0.061	9.63E-230	1
Gm10076	4.87E-72	1.130053	0.917	0.612	8.14E-68	1
Hspa8	3.69E-62	1.118031	0.955	0.784	6.18E-58	1
Smc6	2.09E-201	1.114529	0.783	0.106	3.50E-197	1
Ncl	2.23E-95	1.100253	0.894	0.41	3.73E-91	1
Aprt	3.13E-88	1.099562	0.884	0.535	5.24E-84	1
Snrpb	4.93E-146	1.097344	0.886	0.299	8.26E-142	1
Dnmt1	6.33E-184	1.086608	0.747	0.114	1.06E-179	1
mt-Nd5	1.83E-62	1.083367	0.907	0.54	3.06E-58	1
Spc24	9.53E-240	1.080629	0.72	0.044	1.59E-235	1
Rpl17	6.31E-75	1.079112	0.967	0.952	1.06E-70	1
Tmsb10	5.09E-22	1.075874	0.869	0.562	8.51E-18	1
Smc1a	4.62E-106	1.073403	0.828	0.384	7.74E-102	1
Cenpf	7.22E-156	1.067541	0.578	0.053	1.21E-151	1
mt-Nd4l	2.32E-116	1.064799	0.783	0.216	3.88E-112	1
Cdca3	1.75E-211	1.057815	0.705	0.054	2.93E-207	1
Hsp90b1	1.24E-97	1.051858	0.851	0.304	2.07E-93	1
Fbxo5	2.79E-268	1.049406	0.679	0.012	4.67E-264	1

Cenpa	3.01E-112	1.043132	0.682	0.16	5.03E-108	1
Hnrnpf	1.31E-102	1.039967	0.939	0.734	2.19E-98	1
2700029M(	1.23E-170	1.034122	0.75	0.135	2.06E-166	1
Hist1h1c	3.01E-60	1.029992	0.788	0.441	5.04E-56	1
Banf1	6.60E-198	1.029705	0.801	0.118	1.10E-193	1
Dnajc9	7.18E-243	1.014164	0.75	0.051	1.20E-238	1
Rrm1	3.56E-255	1.01089	0.737	0.037	5.95E-251	1
Pgls	2.01E-101	1.010112	0.846	0.382	3.36E-97	1
Mrpl18	6.64E-122	1.008377	0.813	0.31	1.11E-117	1
Cks2	3.36E-94	1.007411	0.823	0.316	5.62E-90	1
Tpx2	7.08E-208	1.000153	0.659	0.038	1.18E-203	1
Krtcap2	1.88E-165	########	0.74	0.112	3.14E-161	1
Sptssa	6.17E-164	########	0.788	0.165	1.03E-159	1
Tyms	9.40E-265	########	0.697	0.017	1.57E-260	1
Rbbp4	1.51E-130	########	0.831	0.272	2.53E-126	1
Cox7c	3.55E-93	########	0.912	0.761	5.93E-89	1
Dtymk	1.04E-187	########	0.717	0.086	1.74E-183	1
Siva1	3.82E-130	########	0.775	0.205	6.39E-126	1
Slbp	9.34E-127	########	0.788	0.221	1.56E-122	1
Ccdc34	6.44E-220	########	0.732	0.059	1.08E-215	1
Rnaseh2c	8.89E-79	########	0.831	0.496	1.49E-74	1
Cox6c	3.09E-83	########	0.922	0.757	5.17E-79	1
Spc25	1.65E-235	########	0.657	0.023	2.75E-231	1
Smarca5	1.19E-110	########	0.854	0.334	1.99E-106	1
Prdx1	6.21E-91	########	0.689	0.179	1.04E-86	1
Atp5o	3.30E-102	########	0.856	0.385	5.52E-98	1
Fkbp2	1.02E-176	########	0.801	0.145	1.71E-172	1
Cks1b	5.37E-234	########	0.727	0.045	8.98E-230	1
Snrpd2	4.64E-140	########	0.864	0.24	7.76E-136	1
Tpr	3.90E-80	########	0.866	0.526	6.53E-76	1
Cox7a2	1.51E-92	########	0.929	0.732	2.53E-88	1
Btf3	1.77E-67	########	0.924	0.749	2.97E-63	1
Lamtor2	1.76E-82	########	0.866	0.493	2.95E-78	1
Hist2h2bb.'	1.68E-206	########	0.591	0.023	2.81E-202	1
Casc5	3.87E-222	########	0.664	0.032	6.48E-218	1
Lsm5	3.97E-145	########	0.793	0.186	6.65E-141	1
Eef1d	1.35E-135	########	0.838	0.204	2.26E-131	1
Hsp90aa1	4.57E-90	########	0.914	0.439	7.65E-86	1
Bola2	3.76E-141	########	0.79	0.184	6.28E-137	1
Lsm6	4.17E-136	########	0.821	0.24	6.98E-132	1
Ccna2	7.86E-232	########	0.677	0.03	1.31E-227	1
Mcm7	1.20E-243	########	0.712	0.034	2.01E-239	1
Lta4h	1.77E-61	########	0.788	0.382	2.96E-57	1
Nasp	4.79E-207	########	0.795	0.085	8.01E-203	1
Hmmr	9.50E-169	########	0.588	0.043	1.59E-164	1
Hist1h3i	2.34E-243	########	0.591	0.003	3.91E-239	1
Gnas	1.81E-78	########	0.902	0.663	3.02E-74	1
Rpl27	1.64E-60	########	0.899	0.64	2.75E-56	1
Asf1b	4.00E-259	########	0.702	0.024	6.70E-255	1
Srsf7	2.02E-151	########	0.788	0.164	3.38E-147	1
Ssrp1	8.34E-212	########	0.78	0.091	1.39E-207	1
Cenpe	1.05E-181	########	0.639	0.052	1.76E-177	1

Bub3	4.59E-125	########	0.79	0.23	7.68E-121	1
Eif2s2	1.37E-92	########	0.879	0.398	2.29E-88	1
Mt2	4.41E-14	########	0.101	0.022	7.38E-10	1
Nudc	3.93E-216	########	0.788	0.085	6.58E-212	1
Ndufc2	1.72E-131	########	0.821	0.229	2.88E-127	1
Usp1	1.13E-172	########	0.747	0.116	1.88E-168	1
Eif3i	5.94E-141	########	0.77	0.161	9.94E-137	1
Pdia3	9.45E-74	########	0.859	0.411	1.58E-69	1
Igfbp4	5.54E-166	########	0.561	0.037	9.27E-162	1
Ncapd3	6.12E-236	########	0.72	0.044	1.02E-231	1
Eef1g	7.71E-54	########	0.778	0.324	1.29E-49	1
Atp5a1	3.12E-86	########	0.869	0.429	5.22E-82	1
Incenp	2.59E-187	########	0.707	0.079	4.33E-183	1
Gstm1	4.64E-144	########	0.52	0.042	7.77E-140	1
Atp5h	8.91E-87	########	0.934	0.774	1.49E-82	1
Nme1	2.13E-132	########	0.636	0.092	3.56E-128	1
Ngp	1.40E-17	########	0.747	0.636	2.35E-13	1
Atp5j2	1.60E-76	########	0.899	0.674	2.68E-72	1
Sec61g	2.57E-49	########	0.891	0.629	4.29E-45	1
Tipin	8.13E-260	########	0.694	0.019	1.36E-255	1
Scp2	3.20E-53	########	0.899	0.687	5.36E-49	1
Lsm4	5.91E-104	########	0.848	0.306	9.89E-100	1
2700094K1	1.66E-181	########	0.674	0.068	2.77E-177	1
Esco2	1.50E-255	########	0.639	0.008	2.52E-251	1
Atp5c1	7.16E-81	########	0.907	0.557	1.20E-76	1
Tacc3	7.49E-216	########	0.702	0.048	1.25E-211	1
Anapc5	1.06E-140	########	0.775	0.179	1.77E-136	1
Rps27a	1.09E-43	########	0.982	0.97	1.83E-39	1
Tmpo	3.75E-76	########	0.793	0.367	6.27E-72	1
Hn1	3.79E-103	########	0.871	0.346	6.34E-99	1
Park7	3.07E-99	########	0.861	0.332	5.14E-95	1
Ezh2	2.76E-221	########	0.677	0.038	4.62E-217	1
Arl6ip1	8.75E-36	########	0.869	0.681	1.46E-31	1
Ndufb5	9.63E-106	########	0.836	0.308	1.61E-101	1
Rpl37	4.18E-44	########	0.962	0.956	6.99E-40	1
Ndufv3	9.55E-67	########	0.859	0.503	1.60E-62	1
Cd63	4.29E-34	########	0.871	0.701	7.17E-30	1
Topbp1	2.82E-207	########	0.74	0.077	4.71E-203	1
Kif15	6.89E-211	########	0.641	0.034	1.15E-206	1
Atp5e	2.54E-62	########	0.944	0.838	4.25E-58	1
U2af1	6.57E-99	########	0.823	0.326	1.10E-94	1
Cycs	2.75E-115	########	0.801	0.228	4.60E-111	1
mt-Co1	2.93E-42	########	0.987	0.985	4.91E-38	1
Cox7b	1.98E-63	########	0.874	0.508	3.31E-59	1
Emp3	3.40E-61	########	0.912	0.643	5.69E-57	1
Timm13	1.33E-117	########	0.828	0.235	2.23E-113	1
Ppp1r14b	3.95E-81	########	0.46	0.078	6.60E-77	1
Eif3a	1.44E-106	########	0.884	0.327	2.41E-102	1
Eif3c	1.60E-84	########	0.871	0.429	2.68E-80	1
Hspe1	1.87E-116	########	0.46	0.041	3.13E-112	1
Mcm6	7.94E-195	########	0.583	0.026	1.33E-190	1
Nol7	5.37E-125	########	0.813	0.219	8.98E-121	1

Orm1	9.64E-78	########	0.664	0.189	1.61E-73	1
Atp5j	1.87E-72	########	0.904	0.726	3.13E-68	1
Eif3h	1.73E-77	########	0.879	0.468	2.89E-73	1
Ndufc1	1.86E-103	########	0.818	0.276	3.11E-99	1
Atp5g3	3.76E-92	########	0.841	0.362	6.29E-88	1
Hnrnpa1	4.05E-152	########	0.808	0.157	6.78E-148	1
Hist1h2bn	5.14E-233	########	0.583	0.006	8.61E-229	1
Pbk	1.09E-268	########	0.669	0.009	1.82E-264	1
Metap2	1.11E-110	########	0.818	0.272	1.85E-106	1
mt-Atp8	5.28E-73	########	0.811	0.334	8.83E-69	1
Hist1h2af	1.19E-235	########	0.586	0.005	1.98E-231	1
Hdgf	7.10E-149	########	0.806	0.172	1.19E-144	1
Slc25a5	2.32E-61	########	0.907	0.577	3.88E-57	1
Lockd	2.74E-231	########	0.684	0.034	4.59E-227	1
Dmkn	8.12E-63	########	0.245	0.019	1.36E-58	1
Cbx5	1.18E-247	########	0.715	0.032	1.97E-243	1
H2afy	2.23E-52	########	0.861	0.595	3.73E-48	1
Mcm5	3.30E-225	########	0.669	0.033	5.52E-221	1
Cct2	1.12E-122	########	0.838	0.237	1.87E-118	1
Mrpl57	7.78E-107	########	0.808	0.279	1.30E-102	1
Dad1	2.32E-95	########	0.73	0.202	3.89E-91	1
Smc3	3.12E-106	########	0.801	0.269	5.22E-102	1
Reep5	7.86E-73	########	0.907	0.609	1.31E-68	1
Ccnb2	5.18E-153	########	0.667	0.078	8.67E-149	1
Plk4	2.02E-235	########	0.717	0.041	3.38E-231	1
Ssb	7.09E-105	########	0.854	0.313	1.19E-100	1
Pnp	2.30E-77	########	0.866	0.44	3.86E-73	1
Tk1	2.09E-234	########	0.649	0.022	3.50E-230	1
E2f8	5.77E-209	########	0.659	0.042	9.66E-205	1
Ndufb3	5.41E-70	########	0.851	0.454	9.05E-66	1
Mcm3	1.10E-225	########	0.641	0.022	1.84E-221	1
Ifi30	1.71E-76	########	0.429	0.071	2.86E-72	1
Plk1	1.34E-202	########	0.596	0.025	2.24E-198	1
Calm2	3.24E-56	########	0.939	0.813	5.41E-52	1
Hist1h2bm	2.10E-235	########	0.578	0.004	3.52E-231	1
Eif4a1	4.39E-57	########	0.919	0.505	7.35E-53	1
Stra13	2.55E-127	########	0.793	0.215	4.27E-123	1
Mrpl51	5.77E-111	########	0.773	0.25	9.65E-107	1
Ywhae	8.40E-74	########	0.884	0.49	1.41E-69	1
Lsm2	2.19E-195	########	0.753	0.08	3.67E-191	1
Cox5b	8.84E-66	########	0.937	0.749	1.48E-61	1
Ndufb10	7.18E-82	########	0.866	0.425	1.20E-77	1
Rdx	2.29E-99	########	0.798	0.283	3.83E-95	1
Sec61b	1.77E-33	########	0.917	0.772	2.97E-29	1
Hnrnpa0	2.86E-79	########	0.876	0.464	4.79E-75	1
Mylpf	1.33E-19	########	0.149	0.032	2.23E-15	1
Dpy30	5.97E-179	########	0.747	0.107	9.99E-175	1
Ms4a6c	3.39E-36	########	0.162	0.018	5.67E-32	1
Cdca7	1.48E-136	########	0.664	0.11	2.48E-132	1
1700020L2	4.03E-118	########	0.682	0.156	6.74E-114	1
Nudt21	2.17E-174	########	0.783	0.131	3.64E-170	1
Hist1h1a	3.10E-197	########	0.505	0.006	5.19E-193	1

Uqcr11	7.21E-56	########	0.874	0.499	1.21E-51	1
15-Sep	7.42E-76	########	0.886	0.475	1.24E-71	1
Ndufab1	1.15E-135	########	0.768	0.17	1.93E-131	1
Pcna	3.46E-165	########	0.646	0.063	5.79E-161	1
Hist1h2be	1.76E-106	########	0.644	0.136	2.95E-102	1
Cdc25a	1.20E-163	########	0.672	0.085	2.00E-159	1
Cdkn3	8.34E-152	########	0.641	0.074	1.40E-147	1
Racgap1	9.26E-206	########	0.629	0.03	1.55E-201	1
Tpm1	4.13E-36	########	0.167	0.019	6.91E-32	1
Lsm3	1.60E-137	########	0.763	0.165	2.67E-133	1
Ndufa5	1.55E-150	########	0.758	0.144	2.59E-146	1
Uba52	1.78E-50	########	0.874	0.519	2.99E-46	1
Lsm8	8.62E-103	########	0.788	0.277	1.44E-98	1
Smdt1	8.92E-57	########	0.884	0.613	1.49E-52	1
Aurkb	1.32E-250	########	0.659	0.015	2.21E-246	1
Eif3e	1.63E-102	########	0.742	0.213	2.73E-98	1
Gatm	1.03E-150	########	0.629	0.081	1.72E-146	1
Ndufa11	1.62E-91	########	0.838	0.341	2.72E-87	1
Ssna1	5.04E-102	########	0.78	0.273	8.44E-98	1
G3bp1	5.56E-164	########	0.737	0.103	9.30E-160	1
Cdv3	7.61E-87	########	0.826	0.346	1.27E-82	1
S100a1	2.43E-126	########	0.694	0.14	4.06E-122	1
Whsc1	8.72E-198	########	0.71	0.065	1.46E-193	1
Mrps14	5.13E-91	########	0.813	0.311	8.59E-87	1
Lmna	1.06E-15	########	0.134	0.034	1.77E-11	1
Ddx39b	1.12E-130	########	0.788	0.187	1.88E-126	1
Suz12	1.92E-122	########	0.788	0.197	3.20E-118	1
Eef2	5.70E-42	########	0.907	0.651	9.54E-38	1
Lgmn	4.05E-25	########	0.144	0.023	6.77E-21	1
Ahnak	3.10E-14	########	0.197	0.074	5.18E-10	1
Napsa	1.08E-40	########	0.836	0.537	1.81E-36	1
Atp5d	9.34E-59	########	0.879	0.526	1.56E-54	1
Npc2	6.24E-31	########	0.838	0.514	1.04E-26	1
Ckap5	4.39E-132	########	0.677	0.12	7.34E-128	1
Hprt	1.38E-129	########	0.785	0.197	2.31E-125	1
Rpa3	1.64E-203	########	0.684	0.055	2.74E-199	1
Ddost	8.69E-143	########	0.73	0.132	1.45E-138	1
Pdia6	1.73E-90	########	0.667	0.163	2.89E-86	1
Gpx4	3.55E-49	########	0.932	0.782	5.94E-45	1
Acadl	2.08E-88	########	0.803	0.32	3.48E-84	1
Psmb2	1.46E-81	########	0.851	0.389	2.45E-77	1
Ybx3	2.36E-159	########	0.551	0.038	3.95E-155	1
Arpp19	1.04E-66	########	0.874	0.609	1.74E-62	1
Atp5b	8.60E-54	########	0.912	0.606	1.44E-49	1
Pcbp1	2.70E-77	########	0.871	0.437	4.51E-73	1
Ctcf	3.66E-82	########	0.795	0.332	6.13E-78	1
Hist1h2ak	2.66E-224	########	0.573	0.008	4.44E-220	1
Hat1	4.26E-204	########	0.679	0.048	7.13E-200	1
Hmgb3	2.02E-218	########	0.667	0.036	3.38E-214	1
Psmb1	1.32E-61	########	0.896	0.566	2.21E-57	1
Dbf4	1.91E-219	########	0.674	0.037	3.20E-215	1
Clec12a	6.67E-29	########	0.795	0.595	1.12E-24	1

Vdac3	1.03E-121	########	0.755	0.197	1.72E-117	1
Ywhah	1.45E-57	########	0.854	0.546	2.42E-53	1
Kdelr2	1.14E-132	########	0.742	0.151	1.90E-128	1
Clspn	4.86E-241	########	0.657	0.019	8.13E-237	1
Srsf2	7.53E-75	########	0.861	0.45	1.26E-70	1
Ywhaq	2.99E-108	########	0.823	0.257	5.00E-104	1
Psmc3	9.36E-90	########	0.831	0.34	1.57E-85	1
Spcs2	5.57E-66	########	0.831	0.382	9.33E-62	1
AW112010	7.06E-73	########	0.283	0.023	1.18E-68	1
Edf1	1.36E-62	########	0.886	0.593	2.28E-58	1
Actn4	7.22E-95	########	0.826	0.311	1.21E-90	1
Nsa2	5.87E-57	########	0.861	0.455	9.83E-53	1
Lamp1	8.61E-53	########	0.894	0.547	1.44E-48	1
Tcp1	4.15E-154	########	0.735	0.124	6.94E-150	1
Hist1h2bb	4.15E-216	########	0.553	0.008	6.94E-212	1
Nop10	5.34E-122	########	0.634	0.103	8.94E-118	1
Cox6a1	1.89E-59	########	0.904	0.642	3.17E-55	1
Uqcr10	3.56E-54	########	0.896	0.629	5.96E-50	1
Rbbp7	9.62E-201	########	0.707	0.06	1.61E-196	1
Pnn	1.22E-96	########	0.722	0.207	2.04E-92	1
Eif3k	2.00E-51	########	0.907	0.63	3.35E-47	1
Ndufb8	2.20E-61	########	0.869	0.502	3.69E-57	1
Nsmce4a	1.18E-115	########	0.74	0.19	1.97E-111	1
Hist1h3f	3.11E-220	########	0.566	0.008	5.21E-216	1
Sgol2a	2.81E-229	########	0.644	0.021	4.70E-225	1
Sptbn1	2.50E-70	########	0.77	0.305	4.18E-66	1
Eri1	6.46E-202	########	0.702	0.058	1.08E-197	1
Srsf9	3.65E-95	########	0.813	0.308	6.10E-91	1
Atp5k	2.40E-52	########	0.889	0.61	4.01E-48	1
Ppib	3.93E-41	########	0.886	0.575	6.58E-37	1
Tomm20	8.41E-83	########	0.846	0.311	1.41E-78	1
Rfc4	3.34E-217	########	0.631	0.027	5.59E-213	1
Ap3s1	2.38E-44	########	0.838	0.602	3.98E-40	1
Polr1d	1.97E-60	########	0.884	0.488	3.30E-56	1
Prdx2	4.10E-86	########	0.588	0.131	6.86E-82	1
Cct8	1.58E-92	########	0.803	0.263	2.65E-88	1
Swi5	3.24E-71	########	0.838	0.356	5.42E-67	1
2010107E0	1.12E-54	########	0.889	0.556	1.88E-50	1
Mbnl1	5.51E-56	########	0.851	0.411	9.22E-52	1
Hells	1.33E-158	########	0.465	0.016	2.23E-154	1
Eny2	1.73E-188	########	0.679	0.057	2.90E-184	1
Rfc1	5.88E-83	########	0.755	0.281	9.84E-79	1
Gpx1	1.57E-43	########	0.942	0.885	2.63E-39	1
Thoc7	3.17E-124	########	0.76	0.18	5.30E-120	1
Ndufv2	3.36E-88	########	0.831	0.344	5.63E-84	1
Ccr2	1.68E-34	########	0.144	0.014	2.81E-30	1
Cct5	2.74E-99	########	0.806	0.256	4.59E-95	1
Cybb	2.92E-20	########	0.838	0.587	4.89E-16	1
Romo1	9.69E-73	########	0.816	0.349	1.62E-68	1
Cmc2	4.40E-175	########	0.707	0.081	7.37E-171	1
Vdac1	8.41E-86	########	0.798	0.3	1.41E-81	1
Hspd1	4.26E-102	########	0.611	0.121	7.13E-98	1

Hspa14	6.86E-119	########	0.76	0.19	1.15E-114	1
Fkbp3	3.54E-171	########	0.654	0.063	5.93E-167	1
Cox4i1	1.36E-52	########	0.96	0.883	2.28E-48	1
Exosc8	7.10E-218	########	0.689	0.042	1.19E-213	1
Pdap1	1.08E-78	########	0.833	0.326	1.80E-74	1
Ndc80	1.25E-226	########	0.624	0.019	2.09E-222	1
Tma7	1.24E-44	########	0.909	0.734	2.08E-40	1
Eif3f	2.98E-41	########	0.886	0.623	4.99E-37	1
Baz1b	8.48E-72	########	0.788	0.334	1.42E-67	1
Aes	7.30E-50	########	0.843	0.587	1.22E-45	1
Hist1h3b	3.56E-235	########	0.583	0.005	5.95E-231	1
Abracl	9.66E-44	########	0.884	0.646	1.62E-39	1
Ptges3	1.49E-95	########	0.813	0.266	2.49E-91	1
Dlgap5	3.06E-188	########	0.621	0.04	5.11E-184	1
Ndufs6	6.26E-67	########	0.856	0.42	1.05E-62	1
Mdh1	5.09E-103	########	0.732	0.204	8.51E-99	1
Scaf11	1.69E-80	########	0.831	0.337	2.82E-76	1
Mafb	3.27E-30	########	0.119	0.01	5.47E-26	1
Syncrip	5.13E-144	########	0.737	0.124	8.58E-140	1
Taf10	2.32E-69	########	0.879	0.465	3.88E-65	1
Mtdh	4.37E-85	########	0.806	0.284	7.31E-81	1
Kif20b	1.62E-178	########	0.566	0.03	2.70E-174	1
Gm26917	3.89E-74	########	0.689	0.21	6.51E-70	1
Hnrnpr	3.77E-149	########	0.707	0.111	6.31E-145	1
Orc6	5.61E-187	########	0.641	0.05	9.39E-183	1
Ndufb7	4.43E-47	########	0.909	0.711	7.41E-43	1
Mat2a	6.46E-166	########	0.732	0.088	1.08E-161	1
Mcm4	8.68E-153	########	0.593	0.057	1.45E-148	1
Manf	2.73E-122	########	0.735	0.151	4.57E-118	1
Canx	3.50E-90	########	0.803	0.252	5.85E-86	1
Med21	1.13E-96	########	0.742	0.219	1.90E-92	1
Baz1a	9.24E-50	########	0.859	0.535	1.55E-45	1
Abhd5	1.30E-45	########	0.745	0.435	2.18E-41	1
Ndufb2	9.47E-83	########	0.763	0.275	1.58E-78	1
C3	4.57E-30	########	0.818	0.62	7.65E-26	1
Hmgn5	7.73E-166	########	0.667	0.074	1.29E-161	1
Xrn2	3.73E-124	########	0.732	0.147	6.25E-120	1
Caprin1	6.54E-101	########	0.818	0.266	1.09E-96	1
Neil3	5.21E-174	########	0.657	0.062	8.72E-170	1
Mrpl42	2.03E-165	########	0.667	0.075	3.40E-161	1
Sarnp	2.12E-92	########	0.788	0.265	3.54E-88	1
Psma2	1.14E-51	########	0.876	0.593	1.91E-47	1
Eif1ax	2.78E-103	########	0.763	0.213	4.66E-99	1
Rad21	4.00E-55	########	0.773	0.376	6.70E-51	1
Psma7	6.54E-39	########	0.894	0.601	1.09E-34	1
Ccne2	8.75E-156	########	0.409	0.005	1.46E-151	1
Nono	3.23E-89	########	0.793	0.287	5.40E-85	1
Cbx1	7.77E-118	########	0.74	0.174	1.30E-113	1
Rnaseh2b	5.19E-205	########	0.687	0.051	8.69E-201	1
Cox8a	2.07E-59	########	0.967	0.919	3.46E-55	1
Pcbp2	1.98E-54	########	0.912	0.587	3.31E-50	1
Sgol1	3.95E-223	########	0.596	0.014	6.62E-219	1

Rnf7	3.96E-83	########	0.793	0.296	6.63E-79	1
Ndufb11	2.49E-45	########	0.879	0.661	4.16E-41	1
Anapc11	2.52E-109	########	0.76	0.203	4.22E-105	1
Pebp1	6.07E-85	########	0.652	0.162	1.02E-80	1
Itgb1	1.35E-106	########	0.753	0.175	2.25E-102	1
Dstn	7.93E-26	########	0.879	0.767	1.33E-21	1
Bzw1	1.64E-54	########	0.886	0.535	2.74E-50	1
Ndufa2	1.40E-42	########	0.914	0.658	2.34E-38	1
Hist1h4c	1.51E-141	########	0.528	0.045	2.53E-137	1
Eif3m	4.89E-80	########	0.763	0.262	8.19E-76	1
Cdkn2c	2.21E-215	########	0.609	0.02	3.69E-211	1
Commd1	6.03E-70	########	0.801	0.356	1.01E-65	1
Lbp	1.07E-60	########	0.697	0.265	1.79E-56	1
Psma3	9.07E-58	########	0.866	0.465	1.52E-53	1
Zmpste24	6.53E-49	########	0.684	0.258	1.09E-44	1
P4hb	1.06E-74	########	0.806	0.285	1.77E-70	1
Cit	2.14E-198	########	0.624	0.035	3.58E-194	1
Pbrm1	3.84E-66	########	0.79	0.327	6.43E-62	1
Supt16	2.55E-94	########	0.768	0.236	4.26E-90	1
Knstrn	1.11E-174	########	0.621	0.049	1.85E-170	1
Anln	1.15E-178	########	0.641	0.056	1.93E-174	1
Ndufs7	3.14E-73	########	0.803	0.309	5.26E-69	1
Zfp706	6.44E-78	########	0.77	0.259	1.08E-73	1
Ak2	6.27E-50	########	0.813	0.419	1.05E-45	1
Chchd2	6.64E-44	########	0.96	0.898	1.11E-39	1
Ppp1cc	9.15E-42	########	0.851	0.646	1.53E-37	1
Larp1b	2.09E-118	########	0.717	0.154	3.50E-114	1
Mlec	3.99E-63	########	0.778	0.341	6.68E-59	1
Agpat2	6.46E-38	########	0.74	0.428	1.08E-33	1
Maz	1.60E-142	########	0.747	0.133	2.67E-138	1
Cops6	1.07E-113	########	0.77	0.202	1.80E-109	1
Ilf2	1.31E-197	########	0.689	0.057	2.20E-193	1
Mrps24	1.10E-88	########	0.803	0.263	1.83E-84	1
Mpp6	4.89E-184	########	0.679	0.06	8.18E-180	1
Psmb5	2.59E-44	########	0.861	0.529	4.34E-40	1
Pde4d	1.53E-87	########	0.684	0.184	2.57E-83	1
Mrpl23	2.27E-98	########	0.598	0.115	3.80E-94	1
Bcl7c	4.23E-126	########	0.699	0.132	7.08E-122	1
Me2	1.73E-76	########	0.742	0.275	2.89E-72	1
Tsn	5.20E-104	########	0.765	0.211	8.69E-100	1
Snrpd3	3.97E-65	########	0.836	0.389	6.64E-61	1
Psmb6	5.66E-63	########	0.826	0.38	9.47E-59	1
Ska1	4.81E-216	########	0.616	0.022	8.05E-212	1
Stag1	2.07E-98	########	0.758	0.211	3.47E-94	1
Cd81	1.17E-69	########	0.717	0.252	1.96E-65	1
Sh3bgrl	1.38E-89	########	0.712	0.198	2.31E-85	1
Calr	4.07E-25	########	0.699	0.366	6.82E-21	1
Ddx39	2.46E-136	########	0.715	0.119	4.11E-132	1
Cdc20	1.07E-178	########	0.588	0.033	1.79E-174	1
Cenpw	5.21E-191	########	0.619	0.039	8.71E-187	1
Sumo3	1.03E-120	########	0.712	0.149	1.73E-116	1
Utp3	6.25E-107	########	0.773	0.215	1.05E-102	1

Hist1h2an	1.13E-230	########	0.558	0.002	1.90E-226	1
Cenph	4.24E-214	########	0.614	0.023	7.10E-210	1
Psma5	1.07E-87	########	0.785	0.254	1.79E-83	1
Sepw1	2.78E-38	########	0.237	0.042	4.66E-34	1
Zfp91	1.00E-85	########	0.803	0.276	1.67E-81	1
Nhp2l1	2.00E-125	########	0.78	0.165	3.34E-121	1
Cct7	9.48E-75	########	0.765	0.291	1.59E-70	1
Cyc1	9.83E-93	########	0.74	0.206	1.64E-88	1
Psma4	1.90E-69	########	0.816	0.335	3.19E-65	1
Eif2s1	3.64E-148	########	0.694	0.102	6.09E-144	1
Dynll1	4.30E-42	########	0.924	0.583	7.19E-38	1
Rps27	7.95E-17	########	0.975	0.958	1.33E-12	1
Sf3b5	5.64E-70	########	0.854	0.371	9.44E-66	1
Prss57	4.21E-93	########	0.53	0.097	7.05E-89	1
Psip1	1.03E-126	########	0.73	0.131	1.72E-122	1
Prim1	3.38E-231	########	0.591	0.008	5.66E-227	1
2810428I1!	3.84E-109	########	0.694	0.156	6.43E-105	1
Ncapg	1.76E-215	########	0.624	0.024	2.95E-211	1
Grpel1	3.38E-90	########	0.755	0.238	5.65E-86	1
Cox6b1	1.62E-39	########	0.932	0.805	2.71E-35	1
Smarcc1	1.23E-226	########	0.672	0.031	2.05E-222	1
Rpn2	3.92E-123	########	0.682	0.125	6.56E-119	1
Stub1	4.89E-112	########	0.753	0.187	8.19E-108	1
2700060E0	6.78E-84	########	0.78	0.255	1.13E-79	1
Tomm5	5.86E-90	########	0.742	0.216	9.80E-86	1
Psmb4	6.58E-48	########	0.869	0.483	1.10E-43	1
Emg1	1.15E-103	########	0.697	0.165	1.92E-99	1
Nedd4	1.41E-63	########	0.687	0.243	2.37E-59	1
Tuba1c	2.09E-65	########	0.811	0.327	3.50E-61	1
Dpm3	1.99E-90	########	0.758	0.229	3.33E-86	1
Irf2bp2	4.13E-60	########	0.848	0.408	6.90E-56	1
Kif4	6.59E-199	########	0.581	0.023	1.10E-194	1
Ero1l	9.30E-59	########	0.788	0.344	1.56E-54	1
2410015M2	1.57E-64	########	0.79	0.337	2.62E-60	1
Nsmce1	2.70E-101	########	0.775	0.215	4.51E-97	1
Rad51ap1	3.16E-255	########	0.649	0.01	5.28E-251	1
Ncapd2	4.93E-195	########	0.611	0.034	8.25E-191	1
Rbx1	1.24E-43	########	0.904	0.673	2.07E-39	1
Prpf4b	2.17E-83	########	0.753	0.237	3.64E-79	1
Ppm1g	1.37E-114	########	0.727	0.165	2.29E-110	1
Raly	1.73E-64	########	0.859	0.428	2.89E-60	1
Uchl5	1.51E-107	########	0.768	0.188	2.53E-103	1
Ndufa6	7.71E-42	########	0.886	0.586	1.29E-37	1
Srrm1	5.85E-54	########	0.823	0.417	9.78E-50	1
Cdc37	3.39E-71	########	0.798	0.321	5.67E-67	1
Srsf10	6.01E-113	########	0.76	0.178	1.01E-108	1
Elof1	3.25E-103	########	0.722	0.188	5.44E-99	1
Clta	8.60E-40	########	0.914	0.753	1.44E-35	1
Tbrg1	1.42E-58	########	0.735	0.308	2.37E-54	1
Ndufs8	1.39E-82	########	0.75	0.243	2.33E-78	1
Ccnb1	4.14E-146	########	0.51	0.034	6.92E-142	1
Arhgap19	1.39E-96	########	0.604	0.13	2.32E-92	1

Cdk4	2.70E-165	########	0.482	0.016	4.51E-161	1
Ppig	2.80E-58	########	0.828	0.385	4.68E-54	1
Csrp1	4.12E-200	########	0.654	0.043	6.89E-196	1
Srsf1	4.01E-101	########	0.755	0.203	6.70E-97	1
Cetn3	4.79E-99	########	0.765	0.214	8.02E-95	1
Gca	5.43E-42	########	0.702	0.359	9.09E-38	1
Rif1	1.57E-202	########	0.571	0.017	2.63E-198	1
Ptpn18	1.39E-27	########	0.874	0.634	2.32E-23	1
Ppil1	6.32E-177	########	0.662	0.066	1.06E-172	1
Bag1	6.69E-56	########	0.821	0.398	1.12E-51	1
Ccnf	9.82E-228	########	0.588	0.009	1.64E-223	1
Nuf2	6.79E-203	########	0.596	0.024	1.14E-198	1
Mrpl15	5.15E-150	########	0.677	0.097	8.62E-146	1
Mis18bp1	1.51E-190	########	0.553	0.02	2.53E-186	1
Hnrnpul2	8.33E-49	########	0.808	0.453	1.39E-44	1
Mrps18c	1.64E-56	########	0.833	0.434	2.74E-52	1
Naa50	2.84E-145	########	0.712	0.108	4.75E-141	1
Anapc13	1.55E-58	########	0.823	0.373	2.60E-54	1
Paics	5.03E-182	########	0.538	0.022	8.41E-178	1
Pole4	2.25E-158	########	0.657	0.078	3.77E-154	1
Sae1	1.80E-187	########	0.682	0.061	3.00E-183	1
Mapre1	8.02E-53	########	0.884	0.511	1.34E-48	1
Taf15	3.89E-105	########	0.689	0.154	6.52E-101	1
Fubp1	2.06E-114	########	0.598	0.095	3.45E-110	1
Ncapg2	1.06E-195	########	0.626	0.036	1.78E-191	1
Mrps17	5.42E-139	########	0.725	0.127	9.07E-135	1
Tecr	4.35E-32	########	0.826	0.551	7.28E-28	1
Nhp2	2.62E-126	########	0.523	0.053	4.39E-122	1
Tbca	5.47E-58	########	0.833	0.382	9.16E-54	1
Bcas2	1.62E-118	########	0.747	0.172	2.72E-114	1
Phb2	2.41E-76	########	0.682	0.203	4.03E-72	1
Eif5b	1.19E-85	########	0.753	0.215	1.99E-81	1
Bub1b	1.35E-194	########	0.586	0.026	2.25E-190	1
Minos1	3.42E-44	########	0.884	0.544	5.73E-40	1
Hist2h2aa1	1.95E-88	########	0.601	0.131	3.26E-84	1
Rpia	1.08E-112	########	0.684	0.143	1.81E-108	1
Trmt112	1.04E-92	########	0.75	0.215	1.74E-88	1
Gspt1	1.49E-142	########	0.682	0.097	2.49E-138	1
Chchd1	4.25E-118	########	0.72	0.153	7.11E-114	1
Rfc3	2.43E-186	########	0.54	0.019	4.07E-182	1
Oxct1	2.97E-162	########	0.646	0.068	4.97E-158	1
Nt5c	6.26E-114	########	0.624	0.111	1.05E-109	1
Pdcd5	1.49E-71	########	0.79	0.299	2.49E-67	1
Mrps16	3.13E-84	########	0.773	0.258	5.24E-80	1
Oaz1	3.54E-46	########	0.96	0.933	5.92E-42	1
Dnajc3	1.83E-81	########	0.76	0.245	3.06E-77	1
Fam173a	4.67E-77	########	0.778	0.286	7.81E-73	1
Mrpl28	6.40E-133	########	0.674	0.115	1.07E-128	1
Uqcrc1	3.77E-74	########	0.717	0.236	6.30E-70	1
Tmem160	1.23E-57	########	0.747	0.302	2.05E-53	1
Tmco1	4.23E-76	########	0.753	0.259	7.08E-72	1
Syne2	9.46E-45	########	0.705	0.324	1.58E-40	1

Ssbp1	7.04E-94	########	0.715	0.189	1.18E-89	1
Vdac2	8.90E-39	########	0.869	0.619	1.49E-34	1
Rbmxl1	1.34E-159	########	0.636	0.062	2.25E-155	1
Cacybp	7.33E-108	########	0.722	0.167	1.23E-103	1
Ube2m	1.84E-52	########	0.848	0.479	3.08E-48	1
Ethe1	7.03E-33	########	0.77	0.477	1.18E-28	1
Fdps	5.41E-65	########	0.631	0.198	9.04E-61	1
Dtnbp1	1.08E-101	########	0.715	0.185	1.80E-97	1
Dock10	5.36E-159	########	0.657	0.071	8.96E-155	1
Tmx4	1.53E-42	########	0.735	0.377	2.57E-38	1
Dnajc2	1.83E-131	########	0.664	0.103	3.06E-127	1
Kif22	9.93E-198	########	0.576	0.021	1.66E-193	1
Tra2b	2.40E-78	########	0.816	0.288	4.01E-74	1
Tomm7	1.75E-34	########	0.924	0.72	2.92E-30	1
Mrfap1	1.39E-58	########	0.838	0.418	2.32E-54	1
Aurka	8.13E-178	########	0.561	0.028	1.36E-173	1
Uqcrq	1.67E-32	########	0.914	0.66	2.79E-28	1
Cited2	2.06E-35	########	0.851	0.536	3.44E-31	1
Ube2i	4.11E-45	########	0.866	0.549	6.87E-41	1
Arhgap11a	2.11E-88	########	0.639	0.16	3.53E-84	1
Trim59	3.00E-189	########	0.593	0.03	5.02E-185	1
Dhx9	1.03E-118	########	0.679	0.134	1.73E-114	1
Slc25a3	1.00E-33	########	0.917	0.628	1.67E-29	1
Sfxn1	3.15E-151	########	0.588	0.059	5.28E-147	1
Nxt1	1.26E-148	########	0.684	0.093	2.11E-144	1
Pa2g4	1.42E-200	########	0.609	0.027	2.38E-196	1
Rwdd1	1.49E-77	########	0.75	0.244	2.50E-73	1
Rbl1	1.58E-201	########	0.593	0.024	2.65E-197	1
Eif4g1	4.22E-80	########	0.682	0.19	7.06E-76	1
Tmed9	1.18E-62	########	0.778	0.303	1.97E-58	1
Bsg	8.04E-54	########	0.846	0.4	1.35E-49	1
Hexb	6.82E-77	########	0.753	0.252	1.14E-72	1
Hnrnpk	5.30E-42	########	0.917	0.725	8.87E-38	1
Hvcn1	7.39E-77	########	0.649	0.182	1.24E-72	1
Cd47	8.24E-34	########	0.929	0.798	1.38E-29	1
Mtch1	6.36E-58	########	0.758	0.328	1.06E-53	1
Mrps33	4.80E-76	########	0.783	0.274	8.03E-72	1
Ncam1	1.85E-56	########	0.571	0.192	3.10E-52	1
Dhx15	6.86E-104	########	0.763	0.199	1.15E-99	1
Fen1	2.19E-194	########	0.598	0.03	3.67E-190	1
Thrap3	3.60E-66	########	0.813	0.337	6.02E-62	1
Pycard	8.02E-37	########	0.801	0.437	1.34E-32	1
Aars	2.89E-207	########	0.649	0.035	4.83E-203	1
Luc7l3	1.26E-62	########	0.732	0.273	2.12E-58	1
Rangap1	1.77E-198	########	0.644	0.038	2.96E-194	1
Wdr76	1.14E-200	########	0.576	0.02	1.91E-196	1
Uba2	7.66E-103	########	0.735	0.188	1.28E-98	1
Eif3j1	4.43E-108	########	0.697	0.146	7.41E-104	1
Srp9	2.72E-37	########	0.886	0.596	4.56E-33	1
Uhrf1	9.86E-206	########	0.568	0.015	1.65E-201	1
Mrpl52	3.67E-47	########	0.561	0.179	6.15E-43	1
Erp29	3.55E-38	########	0.821	0.457	5.94E-34	1

Sdf2l1	7.26E-137	########	0.442	0.023	1.21E-132	1
Glrx3	3.47E-118	########	0.596	0.092	5.80E-114	1
Psmd14	1.43E-77	########	0.775	0.273	2.40E-73	1
Eif6	3.16E-62	########	0.79	0.321	5.29E-58	1
Csrp2	4.36E-153	########	0.508	0.029	7.30E-149	1
Ict1	3.39E-132	########	0.694	0.124	5.67E-128	1
Hnrnpm	3.88E-48	########	0.869	0.488	6.49E-44	1
Rcc2	5.26E-194	########	0.609	0.033	8.81E-190	1
Rer1	7.43E-46	########	0.866	0.52	1.24E-41	1
Magoh	7.71E-58	########	0.828	0.406	1.29E-53	1
Setd8	2.89E-39	########	0.821	0.492	4.83E-35	1
Tcea1	1.86E-70	########	0.77	0.286	3.12E-66	1
Ndufa12	6.64E-87	########	0.677	0.184	1.11E-82	1
Ms4a6b	1.57E-39	########	0.149	0.011	2.63E-35	1
Snrnp40	1.44E-152	########	0.697	0.097	2.41E-148	1
Mrpl12	3.83E-165	########	0.523	0.026	6.41E-161	1
Cisd2	5.81E-75	########	0.76	0.246	9.73E-71	1
Gm11808	2.30E-72	########	0.73	0.243	3.85E-68	1
Cdca2	1.24E-205	########	0.545	0.01	2.08E-201	1
Cmtm7	7.47E-25	########	0.866	0.622	1.25E-20	1
Eif4e	2.03E-120	########	0.667	0.112	3.40E-116	1
H2-Ab1	3.52E-19	########	0.114	0.019	5.90E-15	1
Immt	3.01E-123	########	0.667	0.119	5.03E-119	1
Yeats4	3.65E-55	########	0.783	0.365	6.11E-51	1
Uqcc2	2.48E-99	########	0.497	0.07	4.15E-95	1
1110004F1	1.64E-70	########	0.697	0.231	2.75E-66	1
Snrpa1	1.06E-155	########	0.614	0.064	1.77E-151	1
Mrpl17	2.86E-94	########	0.657	0.145	4.78E-90	1
Brd8	1.00E-65	########	0.715	0.252	1.68E-61	1
Eif3g	1.74E-82	########	0.571	0.122	2.91E-78	1
Rad50	1.68E-164	########	0.601	0.049	2.80E-160	1
Fundc2	1.45E-137	########	0.596	0.073	2.42E-133	1
Prpf40a	8.60E-43	########	0.846	0.462	1.44E-38	1
Lamtor4	3.36E-25	########	0.874	0.701	5.62E-21	1
Mthfd2	3.78E-127	########	0.609	0.088	6.32E-123	1
Cntrl	4.91E-44	########	0.76	0.361	8.22E-40	1
Nfyb	1.27E-70	########	0.707	0.233	2.12E-66	1
Matr3	6.41E-91	########	0.763	0.216	1.07E-86	1
Spn	4.02E-85	########	0.636	0.16	6.73E-81	1
Commd3	1.41E-114	########	0.697	0.143	2.35E-110	1
Psmd13	4.29E-52	########	0.818	0.4	7.18E-48	1
Stk16	3.63E-62	########	0.76	0.313	6.08E-58	1
Ube2e3	9.53E-128	########	0.682	0.113	1.59E-123	1
Aurkaip1	6.84E-39	########	0.859	0.544	1.15E-34	1
Srp19	4.04E-61	########	0.783	0.324	6.76E-57	1
Ncaph	6.86E-210	########	0.558	0.012	1.15E-205	1
Pds5a	2.58E-81	########	0.732	0.226	4.31E-77	1
Nupr1	2.50E-11	########	0.101	0.027	4.18E-07	1
Uchl3	1.76E-134	########	0.604	0.078	2.94E-130	1
Mrps12	2.24E-97	########	0.682	0.159	3.75E-93	1
Psmc2	4.47E-71	########	0.765	0.291	7.48E-67	1
Psmc4	5.65E-87	########	0.77	0.242	9.45E-83	1

Mrps15	9.93E-66	########	0.725	0.259	1.66E-61	1
Gltp	9.42E-89	########	0.77	0.222	1.58E-84	1
Mrpl21	1.97E-118	########	0.593	0.091	3.29E-114	1
Tgfb1	7.73E-69	########	0.783	0.286	1.29E-64	1
Trappc2l	1.23E-62	########	0.795	0.337	2.06E-58	1
Tmem258	5.79E-36	########	0.866	0.532	9.69E-32	1
Akr1a1	4.46E-27	########	0.71	0.363	7.47E-23	1
Gusb	8.23E-77	########	0.629	0.164	1.38E-72	1
Txnl1	7.61E-72	########	0.823	0.331	1.27E-67	1
Ebp	8.28E-69	########	0.747	0.252	1.39E-64	1
Abcf1	8.35E-91	########	0.682	0.161	1.40E-86	1
Ppp1ca	3.59E-37	########	0.904	0.802	6.00E-33	1
Arhgdib	8.43E-21	########	0.944	0.926	1.41E-16	1
Dazap1	1.99E-91	########	0.692	0.175	3.34E-87	1
Mcee	2.37E-56	########	0.705	0.284	3.97E-52	1
Ap2s1	6.28E-39	########	0.869	0.553	1.05E-34	1
Sec11c	2.95E-29	########	0.866	0.68	4.94E-25	1
Pin1	1.23E-88	########	0.674	0.171	2.05E-84	1
Psmc6	3.12E-62	########	0.77	0.312	5.21E-58	1
Ddrgk1	9.06E-146	########	0.604	0.063	1.52E-141	1
F13a1	2.29E-37	########	0.169	0.019	3.83E-33	1
Cct4	6.63E-60	########	0.699	0.248	1.11E-55	1
Ada	8.17E-73	########	0.674	0.205	1.37E-68	1
Srrt	5.22E-98	########	0.682	0.164	8.73E-94	1
Srpk1	3.41E-89	########	0.697	0.192	5.70E-85	1
Id2	1.38E-29	########	0.73	0.383	2.31E-25	1
Ewsr1	3.04E-70	########	0.813	0.307	5.09E-66	1
Fkbp1a	2.14E-104	########	0.422	0.038	3.58E-100	1
Bub1	4.66E-191	########	0.568	0.024	7.79E-187	1
Snrnp70	3.34E-61	########	0.801	0.345	5.60E-57	1
Slc43a3	3.41E-178	########	0.563	0.028	5.70E-174	1
Ndufs5	1.75E-34	########	0.856	0.568	2.92E-30	1
Kif23	2.41E-69	########	0.619	0.168	4.04E-65	1
Eif4a3	1.11E-97	########	0.717	0.182	1.85E-93	1
Psmd6	5.37E-60	########	0.725	0.277	8.99E-56	1
Cuta	2.57E-74	########	0.717	0.225	4.29E-70	1
Nelfe	1.94E-135	########	0.636	0.086	3.25E-131	1
Rfc5	4.18E-189	########	0.598	0.033	6.99E-185	1
Mad2l1	1.25E-179	########	0.598	0.038	2.09E-175	1
Shcbp1	5.92E-205	########	0.609	0.025	9.90E-201	1
Ndufa10	1.13E-70	########	0.636	0.182	1.89E-66	1
Arl6ip4	1.63E-96	########	0.677	0.159	2.72E-92	1
Polr2m	4.13E-135	########	0.674	0.106	6.92E-131	1
Chaf1a	9.47E-205	########	0.563	0.015	1.58E-200	1
Ndufs2	2.52E-50	########	0.783	0.34	4.22E-46	1
Dnajc19	1.66E-95	########	0.649	0.147	2.78E-91	1
Rnps1	1.92E-108	########	0.677	0.138	3.21E-104	1
Lnp	1.08E-57	########	0.689	0.269	1.80E-53	1
Ckap2l	4.00E-159	########	0.591	0.046	6.69E-155	1
Slc25a4	7.57E-52	########	0.197	0.015	1.27E-47	1
Mgst2	1.08E-21	########	0.722	0.468	1.81E-17	1
Sepp1	3.36E-09	########	0.389	0.212	5.61E-05	1

Ndufb6	1.58E-52	########	0.783	0.337	2.65E-48	1
Klf4	1.36E-27	########	0.146	0.021	2.28E-23	1
Bax	2.86E-86	########	0.619	0.139	4.79E-82	1
Eprs	1.94E-140	########	0.533	0.045	3.24E-136	1
Atp5f1	1.05E-32	########	0.929	0.788	1.76E-28	1
Ndufb4	1.94E-39	########	0.831	0.45	3.24E-35	1
Rap1a	1.18E-31	########	0.894	0.676	1.98E-27	1
Fam96a	2.73E-52	########	0.803	0.383	4.57E-48	1
Kcnq1ot1	5.17E-83	########	0.311	0.023	8.65E-79	1
Pcm1	4.85E-91	########	0.659	0.16	8.12E-87	1
Mrpl20	4.52E-36	########	0.821	0.474	7.55E-32	1
Hdac1	7.46E-71	########	0.732	0.252	1.25E-66	1
Mrps26	2.81E-173	########	0.611	0.045	4.70E-169	1
Cdk2ap1	5.72E-128	########	0.677	0.11	9.57E-124	1
Polr2f	5.14E-84	########	0.636	0.147	8.60E-80	1
Nucb1	2.80E-83	########	0.705	0.203	4.68E-79	1
Itm2b	6.29E-20	########	0.97	0.944	1.05E-15	1
Cops5	2.11E-74	########	0.732	0.232	3.52E-70	1
Tceb2	1.65E-32	########	0.944	0.84	2.76E-28	1
Xpo1	4.98E-115	########	0.697	0.129	8.33E-111	1
Ap2a2	7.66E-59	########	0.785	0.3	1.28E-54	1
Cdc45	2.85E-182	########	0.588	0.033	4.76E-178	1
Cmpk1	1.37E-37	########	0.841	0.529	2.29E-33	1
Psmc5	1.10E-69	########	0.689	0.224	1.84E-65	1
U2surp	5.21E-85	########	0.72	0.205	8.71E-81	1
Mrpl30	5.20E-57	########	0.778	0.307	8.70E-53	1
Tpt1	4.19E-22	########	0.992	0.997	7.02E-18	1
Cnbp	3.48E-57	########	0.775	0.281	5.82E-53	1
Lage3	2.85E-137	########	0.606	0.076	4.77E-133	1
Ubl4a	6.33E-97	########	0.657	0.145	1.06E-92	1
Mcm2	9.80E-170	########	0.48	0.013	1.64E-165	1
7-Sep	1.12E-34	########	0.861	0.641	1.87E-30	1
Tpi1	5.66E-41	########	0.841	0.481	9.47E-37	1
Rad51	5.66E-204	########	0.513	0.004	9.46E-200	1
Nrm	1.30E-170	########	0.591	0.039	2.18E-166	1
Wdr83os	3.94E-57	########	0.702	0.262	6.59E-53	1
Ebna1bp2	2.91E-82	########	0.702	0.2	4.86E-78	1
Tmem14c	1.64E-33	########	0.886	0.618	2.74E-29	1
Ssbp4	1.50E-97	########	0.699	0.169	2.51E-93	1
Uqcrh	1.32E-20	########	0.942	0.814	2.21E-16	1
Srsf4	6.63E-106	########	0.662	0.135	1.11E-101	1
Psma6	1.32E-38	########	0.838	0.454	2.20E-34	1
Pold3	1.82E-142	########	0.588	0.062	3.05E-138	1
Faf1	3.87E-103	########	0.629	0.125	6.47E-99	1
Ctdspl2	2.56E-122	########	0.614	0.091	4.29E-118	1
Ssr2	3.67E-76	########	0.586	0.145	6.14E-72	1
Etfa	1.66E-95	########	0.644	0.142	2.77E-91	1
Syngr2	1.76E-58	########	0.76	0.299	2.95E-54	1
Depdc1a	5.71E-164	########	0.503	0.022	9.56E-160	1
Psmd2	3.21E-58	########	0.715	0.265	5.37E-54	1
Coa3	1.92E-48	########	0.768	0.352	3.22E-44	1
Id1	1.38E-57	########	0.689	0.237	2.30E-53	1

Naa38	3.93E-87	########	0.619	0.142	6.57E-83	1
Rasgrp2	3.22E-27	########	0.803	0.555	5.38E-23	1
Ptgr1	1.29E-91	########	0.583	0.121	2.15E-87	1
Hjurp	1.19E-22	########	0.765	0.552	1.98E-18	1
Cenpk	4.32E-206	########	0.545	0.01	7.23E-202	1
Atp1a1	2.51E-60	########	0.515	0.124	4.20E-56	1
Arglu1	7.66E-47	########	0.78	0.36	1.28E-42	1
Ift27	2.84E-152	########	0.533	0.037	4.76E-148	1
Med30	1.84E-63	########	0.742	0.275	3.08E-59	1
Eif3b	1.84E-134	########	0.535	0.051	3.07E-130	1
Ptbp1	1.71E-101	########	0.619	0.117	2.87E-97	1
Polr2j	2.14E-48	########	0.775	0.366	3.58E-44	1
Srsf6	3.93E-72	########	0.74	0.239	6.58E-68	1
Actl6a	2.55E-156	########	0.611	0.059	4.27E-152	1
Hmbs	4.44E-148	########	0.601	0.064	7.43E-144	1
Sap30	1.15E-63	########	0.682	0.228	1.93E-59	1
Cct3	9.15E-99	########	0.467	0.059	1.53E-94	1
Gm16286	3.16E-71	########	0.74	0.251	5.28E-67	1
Pcbd2	3.21E-80	########	0.727	0.216	5.37E-76	1
Dcun1d5	2.01E-72	########	0.73	0.236	3.37E-68	1
Usp14	1.65E-140	########	0.586	0.066	2.76E-136	1
Mxd3	5.47E-186	########	0.485	0.007	9.16E-182	1
Cep57	1.05E-164	########	0.614	0.052	1.76E-160	1
Cul1	2.88E-72	########	0.687	0.201	4.82E-68	1
Rgs10	3.12E-113	########	0.412	0.029	5.22E-109	1
Stt3b	1.33E-94	########	0.672	0.157	2.23E-90	1
Sfpq	1.62E-36	########	0.881	0.532	2.71E-32	1
Ube2t	6.89E-224	########	0.563	0.006	1.15E-219	1
Uqcrfs1	2.33E-52	########	0.758	0.32	3.90E-48	1
Cuedc2	1.06E-44	########	0.76	0.361	1.78E-40	1
Gpx3	1.19E-74	########	0.268	0.017	2.00E-70	1
Rsl1d1	2.07E-127	########	0.455	0.031	3.46E-123	1
Clec4a2	4.53E-20	########	0.848	0.685	7.58E-16	1
Rtf1	1.76E-59	########	0.735	0.257	2.95E-55	1
1810022K0	1.16E-83	########	0.609	0.143	1.93E-79	1
Psmd7	1.09E-85	########	0.616	0.142	1.83E-81	1
Cenpc1	2.06E-145	########	0.543	0.046	3.45E-141	1
Erdr1	7.80E-121	########	0.447	0.033	1.31E-116	1
G2e3	1.64E-115	########	0.53	0.067	2.75E-111	1
Sf3b2	4.24E-25	########	0.879	0.697	7.09E-21	1
Cox20	3.12E-61	########	0.73	0.263	5.22E-57	1
Bcap31	1.11E-40	########	0.808	0.415	1.85E-36	1
Psmb8	2.07E-20	########	0.836	0.559	3.46E-16	1
Pola1	1.01E-174	########	0.467	0.008	1.69E-170	1
Fam3c	3.62E-134	########	0.573	0.066	6.05E-130	1
Smarca4	1.50E-90	########	0.659	0.152	2.51E-86	1
Upf3b	3.96E-105	########	0.601	0.106	6.62E-101	1
Ndufa1	3.61E-24	########	0.889	0.623	6.04E-20	1
Phf5a	2.70E-78	########	0.747	0.232	4.52E-74	1
Sec11a	5.95E-65	########	0.722	0.25	9.95E-61	1
Skp1a	3.95E-60	########	0.705	0.253	6.60E-56	1
Hp1bp3	7.15E-35	########	0.758	0.398	1.20E-30	1

Glrx5	3.06E-113	########	0.551	0.074	5.12E-109	1
Nubp1	9.72E-146	########	0.548	0.047	1.63E-141	1
Fkbp4	1.00E-166	########	0.492	0.017	1.67E-162	1
Fignl1	6.87E-220	########	0.553	0.005	1.15E-215	1
Aimp1	6.26E-58	########	0.697	0.254	1.05E-53	1
Spcs1	1.02E-38	########	0.816	0.42	1.71E-34	1
Galnt1	1.38E-87	########	0.692	0.177	2.31E-83	1
Tardbp	3.24E-67	########	0.702	0.223	5.42E-63	1
Rb1	1.09E-68	########	0.652	0.197	1.82E-64	1
Anapc15	3.80E-103	########	0.626	0.123	6.36E-99	1
Ola1	1.76E-94	########	0.578	0.114	2.94E-90	1
Gtf2h5	4.74E-48	########	0.798	0.366	7.94E-44	1
Haus4	4.27E-172	########	0.558	0.031	7.15E-168	1
Snx2	4.40E-55	########	0.611	0.193	7.36E-51	1
Polr2g	1.79E-69	########	0.747	0.257	2.99E-65	1
Lmo1	6.08E-43	########	0.631	0.249	1.02E-38	1
Pfdn1	1.18E-79	########	0.598	0.147	1.98E-75	1
Emc7	1.60E-63	########	0.712	0.243	2.68E-59	1
Snx5	2.11E-97	########	0.447	0.053	3.53E-93	1
Hsd17b10	5.45E-53	########	0.75	0.306	9.12E-49	1
mt-Nd6	1.10E-69	########	0.48	0.1	1.83E-65	1
Larp1	5.16E-122	########	0.583	0.08	8.64E-118	1
Ly6c2	3.87E-10	########	0.801	0.691	6.48E-06	1
Dtl	1.40E-144	########	0.419	0.013	2.34E-140	1
Pfdn6	8.85E-61	########	0.745	0.266	1.48E-56	1
Nudt3	2.99E-99	########	0.626	0.127	5.00E-95	1
Hras	1.40E-87	########	0.631	0.138	2.34E-83	1
Ssr1	1.37E-45	########	0.758	0.348	2.29E-41	1
Psmd12	8.68E-67	########	0.737	0.256	1.45E-62	1
AI607873	1.25E-16	########	0.126	0.028	2.09E-12	1
Pabpc1	6.07E-20	########	0.949	0.816	1.02E-15	1
Rps12-ps3	1.35E-79	########	0.654	0.174	2.26E-75	1
Samm50	1.11E-97	########	0.667	0.148	1.86E-93	1
Ptpn7	5.58E-82	########	0.664	0.178	9.33E-78	1
Capns1	4.76E-25	########	0.889	0.641	7.96E-21	1
Tmed2	1.65E-30	########	0.894	0.608	2.76E-26	1
Mrps36	9.73E-116	########	0.535	0.069	1.63E-111	1
Ube2k	3.54E-58	########	0.798	0.329	5.92E-54	1
Glud1	4.68E-27	########	0.846	0.497	7.84E-23	1
Cnih1	7.23E-151	########	0.576	0.049	1.21E-146	1
0610007P1	5.53E-133	########	0.558	0.062	9.25E-129	1
Sf3b6	1.17E-41	########	0.881	0.55	1.96E-37	1
Mrpl13	2.18E-136	########	0.586	0.067	3.64E-132	1
Hnrnpul1	8.53E-32	########	0.818	0.476	1.43E-27	1
Acp1	7.64E-64	########	0.669	0.221	1.28E-59	1
Apitd1	3.20E-194	########	0.53	0.012	5.36E-190	1
Emc6	1.29E-76	########	0.604	0.15	2.17E-72	1
Tm7sf3	3.60E-127	########	0.561	0.068	6.03E-123	1
Nin	5.17E-18	########	0.798	0.543	8.66E-14	1
Calm3	2.50E-24	########	0.889	0.732	4.19E-20	1
Polr2i	1.14E-87	########	0.596	0.125	1.91E-83	1
Kpna2	2.17E-142	########	0.508	0.037	3.63E-138	1

Hirip3	1.04E-182	########	0.535	0.019	1.75E-178	1
Mbd3	4.11E-106	########	0.548	0.081	6.88E-102	1
C1qbp	7.51E-86	########	0.25	0.006	1.26E-81	1
Samd1	1.18E-167	########	0.497	0.017	1.98E-163	1
Echs1	1.79E-108	########	0.533	0.074	3.00E-104	1
Hist1h2bh	8.74E-179	########	0.447	0.002	1.46E-174	1
Fcor	1.67E-82	########	0.437	0.068	2.79E-78	1
Myeov2	2.03E-22	########	0.874	0.594	3.39E-18	1
Arpc5l	2.75E-74	########	0.722	0.222	4.60E-70	1
Nudt5	3.56E-168	########	0.543	0.03	5.96E-164	1
Mgat2	2.69E-106	########	0.682	0.137	4.49E-102	1
Imp3	1.16E-101	########	0.505	0.07	1.94E-97	1
Pld4	2.35E-36	########	0.131	0.009	3.93E-32	1
Sfr1	1.62E-38	########	0.808	0.401	2.72E-34	1
Rpn1	2.00E-57	########	0.689	0.235	3.35E-53	1
Vma21	2.63E-95	########	0.583	0.115	4.39E-91	1
Wbp11	1.18E-56	########	0.76	0.293	1.97E-52	1
Hist1h2ac	2.45E-165	########	0.475	0.015	4.10E-161	1
AI662270	1.54E-65	########	0.634	0.186	2.58E-61	1
Ptgs1	2.25E-62	########	0.596	0.18	3.77E-58	1
Aspm	1.86E-125	########	0.429	0.026	3.11E-121	1
Ldhb	1.75E-113	########	0.472	0.049	2.93E-109	1
Ndufb9	2.26E-23	########	0.924	0.739	3.79E-19	1
Rexo2	4.47E-85	########	0.381	0.042	7.47E-81	1
Lamtor1	4.52E-26	########	0.859	0.593	7.56E-22	1
Gm10073	1.46E-73	########	0.462	0.084	2.44E-69	1
Mrpl54	5.22E-81	########	0.654	0.165	8.74E-77	1
Cdc7	3.92E-195	########	0.538	0.013	6.55E-191	1
Prmt1	1.59E-117	########	0.553	0.07	2.67E-113	1
B230219D2	4.45E-44	########	0.742	0.34	7.45E-40	1
Pcnt	1.07E-116	########	0.578	0.081	1.79E-112	1
Cep128	1.28E-99	########	0.561	0.096	2.14E-95	1
Dnajc15	5.68E-86	########	0.573	0.116	9.50E-82	1
Kif5b	2.69E-28	########	0.864	0.571	4.50E-24	1
Sec62	1.00E-22	########	0.871	0.601	1.67E-18	1
Ms4a6d	2.44E-32	########	0.116	0.008	4.08E-28	1
Hadha	4.08E-54	########	0.73	0.288	6.82E-50	1
Casp8ap2	1.63E-82	########	0.654	0.157	2.74E-78	1
Usmg5	2.43E-24	########	0.874	0.623	4.07E-20	1
Fip1l1	2.74E-67	########	0.687	0.217	4.58E-63	1
Zcchc17	4.44E-87	########	0.649	0.153	7.42E-83	1
Eif1ad	1.37E-125	########	0.621	0.087	2.30E-121	1
Gars	1.57E-107	########	0.601	0.103	2.63E-103	1
Hnrnph1	7.95E-57	########	0.742	0.265	1.33E-52	1
Kif2c	1.22E-159	########	0.49	0.021	2.04E-155	1
Hmgn1	2.70E-25	########	0.116	0.013	4.51E-21	1
Tmem147	1.24E-113	########	0.485	0.052	2.08E-109	1
Ncaph2	1.27E-120	########	0.631	0.099	2.12E-116	1
Kpnb1	1.07E-89	########	0.641	0.14	1.79E-85	1
Bckdk	1.15E-117	########	0.581	0.085	1.93E-113	1
Sypl	3.92E-37	########	0.79	0.423	6.55E-33	1
Pgam1	1.01E-22	########	0.922	0.734	1.70E-18	1

Ndufa13	1.49E-16	########	0.929	0.756	2.49E-12	1
Adh5	2.43E-87	########	0.634	0.147	4.06E-83	1
Larp7	5.38E-107	########	0.566	0.085	9.00E-103	1
Ppp1r7	1.01E-108	########	0.609	0.105	1.70E-104	1
Ndufs3	3.78E-75	########	0.558	0.127	6.33E-71	1
Llph	6.61E-47	########	0.74	0.298	1.11E-42	1
Ppp2cb	1.39E-24	########	0.823	0.562	2.33E-20	1
Brip1	1.50E-180	########	0.503	0.013	2.51E-176	1
A430005L1	4.62E-166	########	0.53	0.027	7.73E-162	1
Nedd8	3.82E-17	########	0.917	0.7	6.39E-13	1
Mrpl40	1.49E-111	########	0.604	0.099	2.49E-107	1
Stk26	5.57E-41	########	0.707	0.311	9.31E-37	1
Msantd3	4.66E-128	########	0.568	0.068	7.79E-124	1
Nans	1.06E-115	########	0.53	0.067	1.78E-111	1
Lsm12	4.95E-53	########	0.778	0.312	8.29E-49	1
Rad23a	9.59E-103	########	0.616	0.114	1.60E-98	1
Fbl	3.64E-84	########	0.346	0.032	6.10E-80	1
Rrp1	3.21E-75	########	0.644	0.171	5.37E-71	1
0610010K1	7.20E-130	########	0.588	0.072	1.21E-125	1
Ndufa3	1.56E-20	########	0.884	0.706	2.61E-16	1
Aif1	7.23E-31	########	0.119	0.009	1.21E-26	1
Exosc5	4.96E-94	########	0.472	0.064	8.30E-90	1
Cdca7l	2.03E-153	########	0.48	0.023	3.39E-149	1
Gadd45gip	3.02E-137	########	0.472	0.03	5.06E-133	1
Ccl9	1.61E-08	########	0.154	0.067	########	1
Tmem208	4.46E-55	########	0.732	0.283	7.47E-51	1
Nop58	5.90E-135	########	0.447	0.023	9.88E-131	1
Tbcb	7.62E-35	########	0.783	0.396	1.28E-30	1
Fau	1.56E-23	########	0.987	0.995	2.62E-19	1
Trappc6b	1.02E-42	########	0.78	0.37	1.71E-38	1
Sms	2.78E-154	########	0.472	0.02	4.64E-150	1
Tmem165	5.03E-33	########	0.75	0.378	8.42E-29	1
Hspa9	4.17E-87	########	0.369	0.035	6.97E-83	1
Grn	5.57E-16	########	0.848	0.575	9.31E-12	1
Exosc7	1.14E-104	########	0.551	0.085	1.90E-100	1
Rfwd3	1.20E-116	########	0.548	0.072	2.01E-112	1
Cisd1	1.13E-99	########	0.321	0.014	1.89E-95	1
Dnajb11	3.45E-56	########	0.725	0.272	5.77E-52	1
Rgcc	3.48E-60	########	0.813	0.318	5.82E-56	1
Suclg1	1.36E-48	########	0.705	0.295	2.27E-44	1
Med8	3.70E-40	########	0.778	0.382	6.19E-36	1
Lmo2	5.07E-50	########	0.636	0.228	8.49E-46	1
Impdh2	1.42E-137	########	0.467	0.027	2.38E-133	1
Ubap2l	4.60E-36	########	0.816	0.403	7.70E-32	1
Trip13	2.42E-168	########	0.49	0.017	4.05E-164	1
Ltf	5.37E-09	########	0.699	0.439	8.99E-05	1
Ptp4a2	4.14E-33	########	0.859	0.497	6.93E-29	1
Gdi2	1.04E-22	########	0.879	0.623	1.74E-18	1
Cenpq	9.94E-82	########	0.606	0.142	1.66E-77	1
Atad5	5.15E-140	########	0.449	0.023	8.62E-136	1
Khsrp	3.17E-92	########	0.616	0.124	5.30E-88	1
Emc2	1.64E-54	########	0.717	0.265	2.74E-50	1

Taf1d	3.31E-89	########	0.528	0.088	5.53E-85	1
Psmb3	2.44E-17	########	0.889	0.627	4.08E-13	1
Fam49b	1.58E-23	########	0.924	0.816	2.65E-19	1
M6pr	1.13E-89	########	0.715	0.178	1.90E-85	1
Dhfr	2.39E-140	########	0.422	0.015	3.99E-136	1
Ndufv1	6.57E-94	########	0.611	0.125	1.10E-89	1
Rnf168	1.07E-137	########	0.525	0.044	1.79E-133	1
Psma1	1.81E-30	########	0.859	0.512	3.03E-26	1
Strap	1.68E-69	########	0.732	0.238	2.81E-65	1
Chrac1	3.95E-68	########	0.636	0.185	6.60E-64	1
Zranb2	1.29E-56	########	0.664	0.221	2.16E-52	1
Mrpl41	4.21E-82	########	0.646	0.163	7.05E-78	1
Cenpu	5.55E-163	########	0.538	0.032	9.29E-159	1
Cdc123	1.53E-64	########	0.674	0.216	2.55E-60	1
Trim27	8.93E-105	########	0.551	0.087	1.49E-100	1
Atp13a3	1.63E-92	########	0.654	0.14	2.72E-88	1
Polr2k	1.72E-42	########	0.76	0.334	2.88E-38	1
Asf1a	7.16E-125	########	0.538	0.062	1.20E-120	1
Pigyl	9.71E-103	########	0.652	0.127	1.62E-98	1
Rpa1	7.97E-182	########	0.523	0.016	1.33E-177	1
Kpna3	8.00E-133	########	0.515	0.045	1.34E-128	1
Vps36	1.05E-43	########	0.78	0.364	1.75E-39	1
Ube2n	6.61E-39	########	0.828	0.416	1.11E-34	1
Snw1	4.92E-59	########	0.722	0.249	8.22E-55	1
Fam208a	2.84E-107	########	0.578	0.091	4.75E-103	1
Casp8	1.19E-85	########	0.616	0.139	1.98E-81	1
Diaph3	1.75E-152	########	0.475	0.022	2.93E-148	1
Anp32a	1.23E-20	########	0.886	0.761	2.06E-16	1
Rbm17	1.66E-68	########	0.689	0.211	2.78E-64	1
Rpa2	2.59E-144	########	0.439	0.017	4.33E-140	1
Acaa2	1.20E-74	########	0.548	0.122	2.01E-70	1
Hnrnpdl	1.56E-52	########	0.808	0.337	2.61E-48	1
Pxylp1	7.26E-99	########	0.545	0.09	1.21E-94	1
Gmps	7.91E-97	########	0.533	0.087	1.32E-92	1
Lrp1	6.12E-40	########	0.172	0.017	1.02E-35	1
Khdrbs1	2.15E-45	########	0.755	0.328	3.60E-41	1
1700020I14	5.85E-31	########	0.783	0.428	9.79E-27	1
Atp6ap2	1.36E-37	########	0.808	0.41	2.27E-33	1
Isca2	7.85E-78	########	0.581	0.129	1.31E-73	1
Mrps34	9.69E-112	########	0.525	0.07	1.62E-107	1
H2-K1	1.62E-08	########	0.902	0.698	########	1
Mrpl34	5.81E-42	########	0.76	0.345	9.73E-38	1
Snx3	8.38E-31	########	0.871	0.537	1.40E-26	1
Nfu1	1.13E-31	########	0.753	0.386	1.89E-27	1
Cntln	1.01E-141	########	0.465	0.026	1.69E-137	1
Med28	7.51E-70	########	0.715	0.22	1.26E-65	1
Sumo1	1.65E-29	########	0.846	0.577	2.77E-25	1
Anxa5	1.30E-29	########	0.672	0.302	2.17E-25	1
Gins4	3.24E-101	########	0.596	0.109	5.42E-97	1
Lyl1	2.98E-92	########	0.576	0.109	4.98E-88	1
Dnajc8	4.28E-25	########	0.826	0.55	7.16E-21	1
Acat1	1.50E-101	########	0.434	0.045	2.50E-97	1

Pcnp	1.12E-53	########	0.745	0.281	1.87E-49	1
11-Sep	2.25E-138	########	0.404	0.012	3.77E-134	1
Plekhj1	2.45E-47	########	0.72	0.297	4.10E-43	1
Odf2	6.30E-89	########	0.551	0.105	1.05E-84	1
Nsmce2	3.65E-84	########	0.634	0.149	6.11E-80	1
Rheb	1.48E-47	########	0.848	0.389	2.48E-43	1
Ets1	1.87E-24	########	0.705	0.363	3.12E-20	1
Sf3a3	2.82E-125	########	0.563	0.067	4.73E-121	1
Dnajc21	4.48E-113	########	0.545	0.073	7.49E-109	1
Impa1	5.74E-47	########	0.644	0.243	9.60E-43	1
1110001J0	4.54E-75	########	0.571	0.138	7.60E-71	1
Ap1s1	8.88E-86	########	0.616	0.139	1.49E-81	1
Timm50	3.48E-154	########	0.505	0.028	5.82E-150	1
Hacd2	1.60E-30	########	0.75	0.406	2.67E-26	1
Pbdc1	2.03E-128	########	0.556	0.061	3.39E-124	1
Morf4l1	4.18E-24	########	0.924	0.799	7.00E-20	1
Ffar2	1.01E-58	########	0.596	0.177	1.69E-54	1
Vimp	8.10E-50	########	0.639	0.209	1.35E-45	1
Psmb10	1.43E-48	########	0.442	0.117	2.40E-44	1
Cmc1	4.50E-122	########	0.427	0.028	7.54E-118	1
Naa20	7.30E-130	########	0.568	0.066	1.22E-125	1
Blm	2.65E-165	########	0.51	0.022	4.44E-161	1
Rbm8a	1.24E-45	########	0.823	0.403	2.08E-41	1
Nop56	4.98E-104	########	0.394	0.029	8.33E-100	1
Eif3l	4.57E-76	########	0.644	0.163	7.65E-72	1
Eif4g2	7.98E-22	########	0.917	0.717	1.34E-17	1
Cdca5	8.51E-202	########	0.51	0.005	1.42E-197	1
H3f3b	1.16E-33	########	0.982	0.99	1.94E-29	1
Fcgr2b	4.53E-47	########	0.571	0.187	7.58E-43	1
Stoml2	1.14E-93	########	0.561	0.098	1.90E-89	1
Agpat5	2.75E-86	########	0.659	0.15	4.60E-82	1
Zcrb1	7.23E-38	########	0.816	0.443	1.21E-33	1
Mphosph8	5.49E-127	########	0.492	0.041	9.18E-123	1
Kif20a	1.20E-134	########	0.467	0.03	2.01E-130	1
Rsbn1	4.54E-69	########	0.619	0.167	7.60E-65	1
Cyba	8.29E-23	########	0.965	0.968	1.39E-18	1
Atrx	2.30E-24	########	0.856	0.562	3.85E-20	1
Mrpl35	5.16E-123	########	0.5	0.049	8.63E-119	1
Ubxn1	1.10E-26	########	0.879	0.559	1.84E-22	1
Ppp3cb	8.48E-72	########	0.611	0.158	1.42E-67	1
1810043H(	2.55E-71	########	0.598	0.156	4.27E-67	1
Mrpl32	8.91E-76	########	0.646	0.171	1.49E-71	1
Pfdn2	1.91E-71	########	0.679	0.192	3.20E-67	1
Hdlbp	2.71E-39	########	0.77	0.337	4.53E-35	1
Rsrc1	3.42E-103	########	0.568	0.093	5.72E-99	1
Eif1b	2.87E-39	########	0.813	0.411	4.80E-35	1
Ddx1	3.40E-149	########	0.472	0.023	5.69E-145	1
Pole3	1.05E-102	########	0.447	0.049	1.76E-98	1
Htra2	2.43E-120	########	0.563	0.071	4.07E-116	1
Desi1	4.63E-102	########	0.495	0.065	7.75E-98	1
Mydgf	7.33E-58	########	0.47	0.113	1.23E-53	1
Pds5b	5.80E-132	########	0.487	0.038	9.70E-128	1

Ost4	7.85E-20	########	0.899	0.639	1.31E-15	1
Spint2	7.00E-78	########	0.523	0.107	1.17E-73	1
Ncbp2	1.90E-98	########	0.508	0.075	3.18E-94	1
Ndufa9	6.32E-87	########	0.609	0.131	1.06E-82	1
Cnpy2	1.68E-57	########	0.533	0.147	2.81E-53	1
2310036O2	2.99E-25	########	0.899	0.63	5.00E-21	1
Ccdc25	9.57E-93	########	0.586	0.113	1.60E-88	1
Ctdspl	5.46E-117	########	0.53	0.065	9.13E-113	1
Dynll2	7.75E-134	########	0.47	0.03	1.30E-129	1
Acin1	4.89E-27	########	0.818	0.508	8.18E-23	1
Mis18a	1.12E-117	########	0.558	0.07	1.87E-113	1
Nxf1	7.88E-53	########	0.674	0.234	1.32E-48	1
Lamtor5	7.78E-38	########	0.778	0.363	1.30E-33	1
Ccdc88a	4.48E-103	########	0.424	0.039	7.50E-99	1
Ccne1	1.41E-135	########	0.364	0.005	2.37E-131	1
Prkcsh	1.98E-90	########	0.525	0.09	3.31E-86	1
Btf3l4	1.06E-74	########	0.677	0.184	1.77E-70	1
Prpf8	6.98E-54	########	0.657	0.229	1.17E-49	1
Asns	3.76E-48	########	0.556	0.185	6.29E-44	1
Mtx2	2.38E-123	########	0.513	0.054	3.99E-119	1
Ppa1	6.81E-94	########	0.338	0.023	1.14E-89	1
Magohb	9.88E-124	########	0.505	0.05	1.65E-119	1
Tcerg1	1.31E-133	########	0.492	0.037	2.19E-129	1
Psme2	1.32E-21	########	0.843	0.5	2.20E-17	1
Foxm1	3.94E-173	########	0.482	0.012	6.60E-169	1
Cep295	6.01E-150	########	0.51	0.031	1.01E-145	1
Cdk6	1.38E-77	########	0.419	0.063	2.31E-73	1
Psme1	1.70E-17	########	0.866	0.587	2.84E-13	1
Slirp	1.38E-74	########	0.604	0.153	2.31E-70	1
St13	4.35E-51	########	0.639	0.215	7.27E-47	1
Rbbp8	1.75E-116	########	0.573	0.075	2.92E-112	1
Ddx21	2.00E-76	########	0.354	0.041	3.35E-72	1
Srp54b	6.05E-54	########	0.735	0.283	1.01E-49	1
Babam1	3.34E-88	########	0.566	0.11	5.59E-84	1
Mb21d1	3.51E-172	########	0.52	0.02	5.88E-168	1
Smarcc2	2.68E-65	########	0.619	0.171	4.48E-61	1
Mphosph1(	1.70E-82	########	0.533	0.104	2.85E-78	1
Gsto1	2.07E-115	########	0.381	0.02	3.47E-111	1
Smc5	6.68E-142	########	0.505	0.035	1.12E-137	1
Top1	1.18E-27	########	0.894	0.687	1.97E-23	1
Taf1	1.55E-76	########	0.596	0.137	2.59E-72	1
Tpm3	4.93E-20	########	0.962	0.912	8.24E-16	1
Tssc4	5.95E-101	########	0.505	0.071	9.95E-97	1
Ramp1	7.76E-15	########	0.667	0.41	1.30E-10	1
Pmf1	2.88E-89	########	0.606	0.125	4.82E-85	1
Sdf4	8.36E-34	########	0.808	0.423	1.40E-29	1
Tcf19	1.04E-141	########	0.399	0.009	1.74E-137	1
Cenpl	3.19E-112	########	0.528	0.064	5.33E-108	1
Smarce1	7.68E-45	########	0.705	0.291	1.29E-40	1
Vars	7.68E-147	########	0.467	0.023	1.29E-142	1
Lair1	4.44E-81	########	0.51	0.095	7.42E-77	1
Hypk	1.42E-63	########	0.631	0.183	2.38E-59	1

Cnih4	7.74E-37	########	0.831	0.452	1.30E-32	1
Lman1	1.35E-87	########	0.482	0.077	2.25E-83	1
Psmd4	7.10E-22	########	0.841	0.512	1.19E-17	1
Vapa	1.32E-20	########	0.869	0.692	2.20E-16	1
Esyt1	3.67E-71	########	0.634	0.174	6.14E-67	1
Lyar	3.32E-106	########	0.523	0.069	5.56E-102	1
Rab28	3.46E-43	########	0.705	0.308	5.79E-39	1
Tkt	7.48E-10	########	0.881	0.775	1.25E-05	1
Jade1	1.56E-142	########	0.551	0.048	2.61E-138	1
Vcp	1.09E-20	########	0.813	0.471	1.82E-16	1
Prelid1	7.69E-17	########	0.907	0.77	1.29E-12	1
Ostc	2.15E-34	########	0.75	0.347	3.60E-30	1
Cenpp	7.10E-171	########	0.49	0.015	1.19E-166	1
Cops3	1.05E-70	########	0.586	0.149	1.76E-66	1
Dctpp1	3.04E-52	########	0.455	0.111	5.09E-48	1
Tpd52l2	2.13E-53	########	0.649	0.214	3.57E-49	1
Prkar2a	7.40E-79	########	0.545	0.111	1.24E-74	1
Eif3d	4.20E-65	########	0.619	0.172	7.03E-61	1
Pam16	1.53E-90	########	0.379	0.037	2.57E-86	1
Usp24	5.00E-84	########	0.422	0.056	8.36E-80	1
Lrpap1	2.00E-72	########	0.432	0.073	3.35E-68	1
Cdca4	4.03E-129	########	0.543	0.056	6.74E-125	1
Exosc3	1.03E-99	########	0.598	0.108	1.73E-95	1
Tmem216	5.78E-46	########	0.588	0.201	9.68E-42	1
Ica1	3.28E-68	########	0.535	0.13	5.49E-64	1
Thoc3	9.63E-117	########	0.523	0.061	1.61E-112	1
Yaf2	7.54E-39	########	0.705	0.311	1.26E-34	1
Tomm40	1.43E-111	########	0.487	0.053	2.40E-107	1
B4galnt1	1.93E-58	########	0.551	0.153	3.24E-54	1
Smn1	3.90E-72	########	0.609	0.152	6.53E-68	1
Ect2	1.18E-131	########	0.442	0.026	1.98E-127	1
Ufc1	4.02E-42	########	0.694	0.282	6.73E-38	1
Agps	1.79E-37	########	0.712	0.327	2.99E-33	1
Rfc2	2.12E-36	########	0.705	0.313	3.55E-32	1
Yif1b	4.91E-123	########	0.492	0.046	8.22E-119	1
Ilk	1.13E-37	########	0.765	0.37	1.90E-33	1
Cpne3	7.55E-12	########	0.78	0.571	1.26E-07	1
Dars	1.34E-47	########	0.626	0.232	2.25E-43	1
Ak6	7.37E-95	########	0.543	0.091	1.23E-90	1
Luc7l2	6.65E-23	########	0.869	0.627	1.11E-18	1
Tmem248	4.22E-80	########	0.631	0.147	7.07E-76	1
Meaf6	5.00E-151	########	0.492	0.026	8.37E-147	1
Phf10	4.92E-64	########	0.687	0.218	8.23E-60	1
Uqcrc2	2.40E-45	########	0.715	0.287	4.01E-41	1
Vrk1	8.26E-30	########	0.763	0.402	1.38E-25	1
Strbp	8.30E-115	########	0.515	0.057	1.39E-110	1
Arl1	9.90E-72	########	0.639	0.168	1.66E-67	1
Akr1b3	1.31E-92	########	0.389	0.039	2.19E-88	1
Atp2a2	8.62E-64	########	0.551	0.143	1.44E-59	1
Rbm15	1.45E-132	########	0.54	0.051	2.43E-128	1
Psmd11	1.22E-47	########	0.732	0.297	2.04E-43	1
Cdk5rap2	9.04E-135	########	0.47	0.03	1.51E-130	1

Smim11	8.73E-51	########	0.571	0.18	1.46E-46	1
Tial1	3.85E-72	########	0.604	0.146	6.44E-68	1
Acbd6	7.93E-41	########	0.586	0.208	1.33E-36	1
Rock2	1.41E-43	########	0.753	0.326	2.36E-39	1
Sass6	2.60E-73	########	0.545	0.122	4.35E-69	1
Kif18a	7.72E-152	########	0.457	0.017	1.29E-147	1
Ppid	4.29E-97	########	0.535	0.085	7.17E-93	1
Ywhab	1.05E-16	########	0.879	0.692	1.75E-12	1
Gngt2	3.26E-12	########	0.237	0.102	5.45E-08	1
Msl3	7.89E-87	########	0.563	0.113	1.32E-82	1
Gsg2	1.24E-142	########	0.477	0.027	2.08E-138	1
Nolc1	8.86E-93	########	0.323	0.019	1.48E-88	1
Clic1	2.87E-25	########	0.96	0.945	4.80E-21	1
Brd7	5.04E-34	########	0.753	0.359	8.43E-30	1
0610012G0	1.66E-57	########	0.609	0.186	2.78E-53	1
Ly86	5.34E-36	########	0.116	0.005	8.94E-32	1
Ppp1r35	1.77E-48	########	0.631	0.229	2.96E-44	1
Hist1h2ai	1.44E-164	########	0.424	0.004	2.41E-160	1
Trim28	1.53E-118	########	0.47	0.039	2.56E-114	1
Ipo5	1.73E-110	########	0.366	0.019	2.90E-106	1
Gatad1	2.67E-33	########	0.763	0.381	4.48E-29	1
Clint1	4.55E-16	########	0.838	0.554	7.61E-12	1
Vcl	9.24E-21	########	0.745	0.442	1.55E-16	1
Rsl24d1	1.12E-66	########	0.548	0.136	1.88E-62	1
Tspan32	8.07E-36	########	0.629	0.268	1.35E-31	1
Mrps7	5.53E-110	########	0.5	0.06	9.26E-106	1
U2af2	1.94E-54	########	0.649	0.217	3.25E-50	1
Tlk1	3.74E-43	########	0.669	0.269	6.26E-39	1
Naa10	6.04E-80	########	0.515	0.099	1.01E-75	1
Akirin2	5.39E-53	########	0.611	0.203	9.01E-49	1
Calu	2.59E-74	########	0.414	0.065	4.34E-70	1
H1f0	1.70E-54	########	0.225	0.021	2.84E-50	1
Myg1	9.48E-117	########	0.513	0.057	1.59E-112	1
Odc1	5.81E-30	########	0.285	0.075	9.72E-26	1
Bccip	8.62E-86	########	0.351	0.033	1.44E-81	1
Prom1	2.61E-57	########	0.553	0.158	4.36E-53	1
Ckap2	4.66E-124	########	0.391	0.017	7.80E-120	1
Melk	4.60E-148	########	0.432	0.014	7.70E-144	1
Txlna	5.72E-97	########	0.525	0.079	9.57E-93	1
Snrpb2	4.08E-55	########	0.636	0.204	6.83E-51	1
Eif4h	1.79E-33	########	0.806	0.418	3.00E-29	1
Rnf26	2.87E-113	########	0.455	0.042	4.81E-109	1
Mrpl14	1.38E-31	########	0.768	0.395	2.31E-27	1
Nmt1	3.49E-36	########	0.765	0.363	5.83E-32	1
Cd48	5.17E-63	########	0.25	0.021	8.65E-59	1
Timm17a	2.50E-89	########	0.452	0.063	4.19E-85	1
Ms4a4c	5.14E-37	########	0.119	0.005	8.61E-33	1
Wbp4	5.23E-45	########	0.72	0.298	8.75E-41	1
Hspa4	6.09E-45	########	0.76	0.338	1.02E-40	1
Mtch2	2.69E-85	########	0.593	0.127	4.50E-81	1
Zc3h15	2.00E-51	########	0.785	0.322	3.35E-47	1
Spag5	1.49E-140	########	0.444	0.021	2.50E-136	1

Arsb	1.81E-84	########	0.51	0.092	3.02E-80	1
Tomm70a	2.88E-84	########	0.545	0.104	4.82E-80	1
Smchd1	7.09E-44	########	0.758	0.321	1.19E-39	1
Cbfb	1.04E-67	########	0.649	0.186	1.74E-63	1
Hnrnpl	7.79E-36	########	0.755	0.349	1.30E-31	1
Ddx46	3.56E-39	########	0.73	0.318	5.96E-35	1
Brca1	8.18E-166	########	0.424	0.003	1.37E-161	1
Snrpc	4.49E-30	########	0.811	0.421	7.51E-26	1
Cdk2	1.52E-54	########	0.588	0.183	2.54E-50	1
Mms22l	1.58E-161	########	0.437	0.008	2.64E-157	1
Gps2	2.26E-48	########	0.672	0.255	3.77E-44	1
H2-DMa	8.23E-58	########	0.212	0.015	1.38E-53	1
Eif4e2	2.40E-35	########	0.75	0.341	4.01E-31	1
Nudcd2	1.75E-125	########	0.49	0.042	2.92E-121	1
Pcgf5	1.62E-98	########	0.571	0.095	2.71E-94	1
Ppih	2.10E-111	########	0.515	0.062	3.51E-107	1
Brd3	2.27E-23	########	0.715	0.389	3.81E-19	1
Hist1h2bf	2.42E-163	########	0.429	0.006	4.05E-159	1
Hsd17b12	3.31E-80	########	0.455	0.073	5.54E-76	1
Uimc1	6.70E-64	########	0.593	0.158	1.12E-59	1
Blvra	6.07E-123	########	0.497	0.047	1.02E-118	1
Selm	5.50E-52	########	0.199	0.015	9.20E-48	1
Pgrmc2	4.45E-115	########	0.485	0.048	7.45E-111	1
Osgep	6.15E-130	########	0.432	0.023	1.03E-125	1
Serpinb6a	2.25E-20	########	0.548	0.256	3.76E-16	1
Gal	2.26E-24	########	0.116	0.014	3.78E-20	1
Rev1	3.92E-102	########	0.508	0.07	6.56E-98	1
Hist1h4a	8.06E-157	########	0.394	0.002	1.35E-152	1
Ehd4	4.41E-54	########	0.295	0.042	7.38E-50	1
Stil	6.26E-164	########	0.447	0.009	1.05E-159	1
Mis12	2.71E-108	########	0.53	0.069	4.54E-104	1
Mrps25	3.62E-100	########	0.457	0.053	6.05E-96	1
Smek1	1.58E-54	########	0.609	0.186	2.65E-50	1
1-Sep	8.90E-24	########	0.654	0.34	1.49E-19	1
Hist1h4i	1.70E-60	########	0.654	0.197	2.84E-56	1
Ercc6l	2.68E-155	########	0.432	0.01	4.48E-151	1
AI413582	4.09E-79	########	0.611	0.141	6.85E-75	1
1700123O2	2.12E-44	########	0.71	0.288	3.55E-40	1
Rexo1	5.04E-56	########	0.641	0.202	8.43E-52	1
Numa1	3.08E-56	########	0.634	0.2	5.15E-52	1
Npm3	1.44E-78	########	0.404	0.055	2.40E-74	1
Ctbp1	9.00E-38	########	0.755	0.347	1.51E-33	1
Ndufs4	1.74E-24	########	0.816	0.471	2.90E-20	1
Fam132a	1.24E-35	########	0.389	0.121	2.07E-31	1
Chtop	4.95E-66	########	0.621	0.165	8.28E-62	1
Akap9	1.14E-38	########	0.591	0.212	1.91E-34	1
Cfdp1	6.20E-56	########	0.586	0.174	1.04E-51	1
Dap3	1.13E-76	########	0.619	0.149	1.88E-72	1
Utp11l	1.05E-52	########	0.586	0.174	1.76E-48	1
Fh1	9.16E-123	########	0.437	0.031	1.53E-118	1
Higd1a	2.49E-41	########	0.646	0.246	4.17E-37	1
Mtf2	3.42E-91	########	0.583	0.109	5.73E-87	1

Rnaset2b	1.18E-71	########	0.52	0.113	1.98E-67	1
Glo1	7.58E-81	########	0.409	0.056	1.27E-76	1
Ccar1	4.62E-48	########	0.659	0.236	7.73E-44	1
Knop1	6.91E-79	########	0.429	0.063	1.16E-74	1
Psmd3	9.24E-79	########	0.525	0.104	1.55E-74	1
Chchd7	1.76E-84	########	0.439	0.062	2.94E-80	1
Psmg4	4.89E-93	########	0.465	0.063	8.18E-89	1
Bri3bp	7.24E-85	########	0.396	0.049	1.21E-80	1
Naa15	4.10E-103	########	0.508	0.068	6.86E-99	1
Hnrnpu	7.99E-43	########	0.78	0.341	1.34E-38	1
Clec4a3	1.67E-13	########	0.116	0.03	2.79E-09	1
Magt1	1.10E-76	########	0.523	0.104	1.84E-72	1
Apip	8.69E-109	########	0.528	0.068	1.45E-104	1
Polr2c	1.08E-52	########	0.694	0.244	1.80E-48	1
Pycr2	4.46E-99	########	0.452	0.055	7.46E-95	1
Hadhb	5.11E-64	########	0.535	0.129	8.55E-60	1
Ten1	7.09E-48	########	0.535	0.165	1.19E-43	1
Comt	1.93E-63	########	0.389	0.068	3.23E-59	1
Parl	4.06E-73	########	0.553	0.127	6.80E-69	1
Hmgxb4	3.70E-91	########	0.52	0.086	6.19E-87	1
Emc8	2.01E-94	########	0.515	0.081	3.36E-90	1
Timm8b	2.22E-35	########	0.77	0.372	3.71E-31	1
Nap1l4	1.85E-45	########	0.73	0.311	3.10E-41	1
Dera	5.57E-74	########	0.525	0.112	9.32E-70	1
Zc3h18	5.98E-66	########	0.616	0.169	1.00E-61	1
Snrnp25	6.91E-93	########	0.457	0.06	1.16E-88	1
Cope	2.30E-30	########	0.763	0.392	3.85E-26	1
Cwc15	1.41E-25	########	0.818	0.463	2.36E-21	1
Itga6	6.66E-41	########	0.23	0.035	1.11E-36	1
Suv39h1	1.36E-149	########	0.462	0.02	2.27E-145	1
Tiam1	3.75E-66	########	0.508	0.116	6.28E-62	1
Pkig	1.03E-122	########	0.472	0.039	1.73E-118	1
Ttk	1.23E-155	########	0.409	0.005	2.06E-151	1
Clns1a	4.45E-96	########	0.442	0.053	7.44E-92	1
Prdx6	4.67E-21	########	0.914	0.707	7.82E-17	1
Cst7	2.34E-87	########	0.25	0.006	3.91E-83	1
Pih1d1	7.26E-67	########	0.662	0.191	1.22E-62	1
Ppp3r1	1.27E-38	########	0.684	0.278	2.12E-34	1
2-Mar	1.01E-86	########	0.422	0.055	1.69E-82	1
Gins2	6.95E-121	########	0.366	0.013	1.16E-116	1
Anxa3	1.37E-13	########	0.77	0.495	2.29E-09	1
Zfp644	6.26E-63	########	0.588	0.159	1.05E-58	1
Mfsd10	5.13E-67	########	0.535	0.125	8.58E-63	1
Ddb1	7.31E-56	########	0.495	0.128	1.22E-51	1
Srp72	5.58E-46	########	0.672	0.243	9.34E-42	1
Idh3a	5.40E-116	########	0.384	0.02	9.03E-112	1
Gpr160	1.72E-77	########	0.513	0.102	2.87E-73	1
Mif	3.26E-17	########	0.538	0.264	5.45E-13	1
Mrpl55	6.22E-99	########	0.515	0.075	1.04E-94	1
Txn2	1.01E-51	########	0.631	0.21	1.69E-47	1
Lin9	1.79E-131	########	0.432	0.023	2.99E-127	1
Ppp2r4	1.40E-75	########	0.601	0.14	2.35E-71	1

Gpc1	2.68E-62	########	0.487	0.111	4.49E-58	1
Gtf3a	1.79E-90	########	0.47	0.067	2.99E-86	1
C330027C(	1.38E-116	########	0.419	0.028	2.31E-112	1
Dna2	3.72E-106	########	0.467	0.049	6.22E-102	1
Atp2a3	7.77E-35	########	0.677	0.297	1.30E-30	1
Bola3	1.99E-92	########	0.336	0.023	3.33E-88	1
Carnmt1	2.74E-120	########	0.444	0.033	4.59E-116	1
Spcs3	2.47E-61	########	0.51	0.126	4.14E-57	1
Trappc6a	9.22E-46	########	0.626	0.225	1.54E-41	1
Nup62	1.53E-136	########	0.503	0.037	2.56E-132	1
Hdac2	1.92E-111	########	0.477	0.049	3.21E-107	1
Tram1	9.75E-30	########	0.763	0.38	1.63E-25	1
Ehmt1	2.46E-75	########	0.432	0.068	4.12E-71	1
Abcd3	4.77E-87	########	0.439	0.06	7.98E-83	1
Rbm25	3.21E-22	########	0.851	0.561	5.37E-18	1
Impa2	3.27E-41	########	0.654	0.273	5.46E-37	1
Uhrf2	6.83E-78	########	0.558	0.117	1.14E-73	1
Top2b	3.20E-28	########	0.725	0.345	5.36E-24	1
Fzr1	8.32E-69	########	0.523	0.121	1.39E-64	1
Apopt1	2.63E-82	########	0.485	0.084	4.39E-78	1
Psmc1	1.53E-52	########	0.561	0.164	2.55E-48	1
Abcf2	1.13E-87	########	0.48	0.075	1.90E-83	1
Mrpl24	1.84E-41	########	0.677	0.266	3.08E-37	1
Gtf2a2	5.47E-37	########	0.793	0.374	9.16E-33	1
Ccdc50	1.30E-105	########	0.361	0.021	2.18E-101	1
Dynlrb1	3.37E-20	########	0.816	0.464	5.63E-16	1
Kif14	7.73E-142	########	0.419	0.014	1.29E-137	1
Ppwd1	1.00E-71	########	0.561	0.131	1.68E-67	1
Pank2	8.74E-71	########	0.566	0.127	1.46E-66	1
Wls	1.04E-55	########	0.624	0.192	1.74E-51	1
Brip1os	5.20E-111	########	0.495	0.056	8.69E-107	1
Idh3b	1.28E-76	########	0.51	0.099	2.14E-72	1
Puf60	1.19E-52	########	0.672	0.229	1.99E-48	1
Pfdn4	2.82E-83	########	0.515	0.092	4.72E-79	1
Acer3	2.24E-76	########	0.452	0.075	3.75E-72	1
Ruvbl2	3.91E-89	########	0.505	0.081	6.55E-85	1
4930438A(	7.84E-28	########	0.639	0.303	1.31E-23	1
Ccdc82	2.55E-66	########	0.583	0.149	4.26E-62	1
Arhgef6	1.77E-27	########	0.689	0.338	2.96E-23	1
Herc2	1.20E-54	########	0.588	0.173	2.01E-50	1
Fxr1	3.84E-40	########	0.677	0.268	6.43E-36	1
Taf6l	4.85E-38	########	0.755	0.359	8.12E-34	1
Tor1a	1.13E-28	########	0.697	0.367	1.88E-24	1
Lrrfip2	2.37E-17	########	0.821	0.512	3.97E-13	1
Fermt3	2.50E-14	########	0.831	0.57	4.18E-10	1
Iqgap3	4.14E-149	########	0.412	0.009	6.93E-145	1
Ltf	1.04E-159	2.319702	0.825	0.337	1.73E-155	2
Ngp	9.27E-122	1.88573	0.884	0.555	1.55E-117	2
Camp	2.17E-110	1.778456	0.851	0.415	3.64E-106	2
Cybb	2.55E-127	1.551827	0.879	0.525	4.26E-123	2
Ifitm6	4.38E-117	1.540608	0.904	0.795	7.33E-113	2
AA467197	6.17E-154	1.449058	0.908	0.51	1.03E-149	2

Lcn2	2.17E-126	1.404062	0.928	0.923	3.63E-122	2
Anxa1	2.07E-121	1.266772	0.93	0.883	3.47E-117	2
Lyz2	8.53E-113	1.265769	0.953	0.917	1.43E-108	2
Chil3	4.87E-93	1.151544	0.841	0.371	8.14E-89	2
Adpgk	2.66E-101	1.026309	0.864	0.48	4.44E-97	2
Aldh2	1.72E-98	1.013358	0.888	0.687	2.88E-94	2
Serpinb1a	1.98E-84	1.012529	0.824	0.374	3.31E-80	2
Cd177	1.05E-93	1.010926	0.884	0.589	1.76E-89	2
Zmpste24	2.31E-63	########	0.585	0.227	3.87E-59	2
Itgb2l	3.99E-121	########	0.847	0.38	6.68E-117	2
Cpne3	1.64E-84	########	0.828	0.512	2.74E-80	2
Anxa3	4.25E-111	########	0.845	0.413	7.12E-107	2
Dstn	1.63E-82	########	0.917	0.73	2.72E-78	2
Lta4h	2.62E-82	########	0.77	0.319	4.38E-78	2
St3gal5	1.60E-87	########	0.813	0.409	2.67E-83	2
Syne1	4.14E-82	########	0.847	0.49	6.93E-78	2
Capg	4.46E-89	########	0.879	0.47	7.47E-85	2
Ly6c2	1.90E-65	########	0.891	0.631	3.18E-61	2
Golim4	3.17E-103	########	0.761	0.302	5.31E-99	2
Ceacam1	2.07E-80	########	0.825	0.442	3.46E-76	2
Arhgdib	2.67E-81	########	0.943	0.924	4.47E-77	2
Ly6g	2.60E-41	########	0.879	0.663	4.35E-37	2
Cebpe	7.05E-65	########	0.746	0.302	1.18E-60	2
Plbd1	5.02E-72	########	0.896	0.696	8.40E-68	2
Tkt	6.37E-78	########	0.913	0.742	1.07E-73	2
Ltb4r1	1.20E-76	########	0.881	0.664	2.02E-72	2
Ckap4	3.95E-61	########	0.885	0.642	6.61E-57	2
Mgst2	1.49E-61	########	0.783	0.396	2.49E-57	2
Scp2	1.28E-57	########	0.891	0.653	2.15E-53	2
Tecr	5.79E-63	########	0.844	0.495	9.69E-59	2
Ncf1	7.69E-61	########	0.911	0.785	1.29E-56	2
Abca13	1.98E-90	########	0.706	0.259	3.32E-86	2
S100a8	2.96E-105	########	0.985	0.988	4.96E-101	2
Wfdc21	7.56E-57	########	0.931	0.93	1.26E-52	2
Ly75	2.71E-84	########	0.718	0.281	4.53E-80	2
Agpat2	1.03E-62	########	0.761	0.364	1.73E-58	2
Orm1	3.37E-39	########	0.469	0.192	5.64E-35	2
Nhsl2	1.99E-68	########	0.753	0.37	3.34E-64	2
C3	2.41E-44	########	0.877	0.559	4.03E-40	2
G6pdx	4.41E-47	########	0.847	0.593	7.37E-43	2
Ppm1m	3.99E-64	########	0.789	0.411	6.68E-60	2
Ceacam10	3.81E-39	########	0.692	0.374	6.37E-35	2
Lims1	6.04E-53	########	0.858	0.549	1.01E-48	2
Inhba	2.76E-73	########	0.559	0.171	4.63E-69	2
S100a9	1.77E-89	########	0.988	0.982	2.96E-85	2
Pglyrp1	5.62E-45	########	0.936	0.911	9.40E-41	2
Trp53inp2	6.08E-97	########	0.606	0.169	1.02E-92	2
Cyfip2	1.34E-45	########	0.835	0.55	2.25E-41	2
Mgst1	1.07E-42	########	0.887	0.713	1.80E-38	2
Rhou	4.51E-75	########	0.655	0.263	7.54E-71	2
4930438AC	2.85E-80	########	0.658	0.235	4.77E-76	2
Mpc2	2.90E-50	########	0.87	0.619	4.85E-46	2

Ak2	3.68E-59	########	0.786	0.362	6.15E-55	2
Ethe1	7.47E-53	########	0.798	0.413	1.25E-48	2
Smim14	7.05E-61	########	0.812	0.471	1.18E-56	2
Lamtor4	5.72E-36	########	0.877	0.668	9.58E-32	2
B230208H1	3.41E-59	########	0.73	0.366	5.70E-55	2
Lbp	4.94E-68	########	0.625	0.221	8.27E-64	2
Arrb2	4.45E-46	########	0.882	0.695	7.45E-42	2
Mettl9	1.54E-42	########	0.87	0.668	2.57E-38	2
Degs1	8.96E-53	########	0.871	0.569	1.50E-48	2
Txndc17	2.49E-50	########	0.858	0.554	4.17E-46	2
Slc31a2	1.27E-63	########	0.804	0.418	2.12E-59	2
Cyba	3.48E-67	########	0.977	0.963	5.82E-63	2
Tcp11l2	4.59E-51	########	0.81	0.469	7.68E-47	2
Ndufb7	4.53E-41	########	0.902	0.679	7.57E-37	2
Fmo5	8.86E-63	########	0.712	0.333	1.48E-58	2
Acvrl1	6.17E-41	########	0.531	0.227	1.03E-36	2
Tmem216	4.63E-67	########	0.515	0.166	7.74E-63	2
Lgals3	9.12E-34	########	0.968	0.898	1.53E-29	2
Flna	1.86E-41	########	0.913	0.793	3.12E-37	2
Aprt	8.58E-39	########	0.859	0.485	1.44E-34	2
Cd63	5.79E-44	########	0.937	0.641	9.69E-40	2
Emp3	4.23E-34	########	0.897	0.602	7.08E-30	2
S100a13	3.54E-40	########	0.847	0.577	5.93E-36	2
Map1lc3a	7.95E-70	########	0.665	0.276	1.33E-65	2
Lmo1	5.50E-54	########	0.551	0.218	9.20E-50	2
Nfu1	2.08E-56	########	0.72	0.336	3.49E-52	2
Ets1	3.01E-56	########	0.686	0.311	5.03E-52	2
Ndufv3	9.66E-37	########	0.809	0.463	1.62E-32	2
Lrmp	6.66E-64	########	0.622	0.254	1.11E-59	2
Myeov2	4.54E-33	########	0.83	0.564	7.60E-29	2
Abhd5	6.42E-39	########	0.744	0.381	1.07E-34	2
1700047M1	2.28E-57	########	0.619	0.247	3.82E-53	2
1-Sep	3.14E-52	########	0.648	0.287	5.25E-48	2
Clec12a	3.47E-36	########	0.841	0.54	5.81E-32	2
Vcl	1.28E-42	########	0.744	0.389	2.14E-38	2
Gca	3.18E-53	########	0.704	0.297	5.32E-49	2
Clec4a2	1.72E-27	########	0.873	0.646	2.88E-23	2
Myo5a	1.36E-43	########	0.626	0.312	2.27E-39	2
Rbfa	2.68E-42	########	0.75	0.42	4.48E-38	2
Slc17a9	1.63E-65	########	0.508	0.165	2.72E-61	2
Nucb2	1.27E-47	########	0.605	0.278	2.13E-43	2
Tst	1.41E-61	########	0.613	0.25	2.35E-57	2
Olfml2b	1.30E-59	########	0.502	0.167	2.18E-55	2
Tmsb10	1.27E-33	########	0.807	0.535	2.13E-29	2
Mapk13	7.09E-31	########	0.839	0.546	1.19E-26	2
Agtrap	7.54E-48	########	0.657	0.306	1.26E-43	2
Tnnt1	2.60E-50	########	0.507	0.197	4.34E-46	2
Slc25a24	1.57E-47	########	0.726	0.381	2.63E-43	2
Tbc1d8	6.57E-58	########	0.632	0.267	1.10E-53	2
Wipi1	1.35E-48	########	0.626	0.288	2.26E-44	2
Lmo4	6.59E-23	########	0.858	0.613	1.10E-18	2
Ywhab	5.72E-25	########	0.89	0.654	9.57E-21	2

Trem3	1.09E-29	########	0.914	0.759	1.82E-25	2
Npepps	3.13E-36	########	0.798	0.477	5.24E-32	2
Cd47	5.61E-24	########	0.917	0.78	9.39E-20	2
Triobp	2.50E-39	########	0.779	0.448	4.18E-35	2
Rab3d	2.32E-36	########	0.764	0.431	3.89E-32	2
Klhl6	2.89E-49	########	0.631	0.282	4.83E-45	2
Sptbn1	2.68E-46	########	0.645	0.28	4.49E-42	2
Ffar2	8.31E-38	########	0.43	0.177	1.39E-33	2
Ikzf1	2.17E-29	########	0.746	0.47	3.63E-25	2
Mbnl1	3.12E-37	########	0.738	0.385	5.23E-33	2
Fam101b	2.31E-22	########	0.864	0.663	3.87E-18	2
Nceh1	2.76E-36	########	0.458	0.2	4.63E-32	2
F730016J0	4.97E-82	########	0.441	0.087	8.31E-78	2
Hsd11b1	7.43E-29	########	0.842	0.542	1.24E-24	2
Flot2	1.47E-33	########	0.747	0.431	2.46E-29	2
Hk2	2.43E-42	########	0.706	0.357	4.06E-38	2
Tspan32	6.55E-48	########	0.564	0.234	1.10E-43	2
Ero1l	1.27E-25	########	0.639	0.333	2.12E-21	2
Adss	4.11E-42	########	0.567	0.262	6.88E-38	2
Acsl1	3.42E-27	########	0.658	0.377	5.72E-23	2
Rps6ka1	6.84E-31	########	0.747	0.447	1.14E-26	2
Aldh3b1	9.77E-28	########	0.806	0.527	1.63E-23	2
Sh3bp5	2.89E-37	########	0.727	0.393	4.84E-33	2
Vamp5	3.26E-34	########	0.752	0.416	5.45E-30	2
Ndufa1	9.95E-24	########	0.845	0.595	1.67E-19	2
Ncf4	4.61E-24	########	0.943	0.83	7.71E-20	2
Cd55	6.35E-54	########	0.456	0.136	1.06E-49	2
Lpgat1	2.72E-40	########	0.609	0.296	4.56E-36	2
Rinl	1.99E-32	########	0.776	0.475	3.33E-28	2
Msmo1	1.83E-54	########	0.594	0.231	3.06E-50	2
Hexb	3.36E-42	########	0.571	0.245	5.62E-38	2
Rgs18	6.53E-29	########	0.746	0.47	1.09E-24	2
Dach1	2.11E-41	########	0.571	0.263	3.53E-37	2
Megf9	1.94E-29	########	0.681	0.387	3.25E-25	2
Hk3	2.90E-27	########	0.825	0.556	4.86E-23	2
Milr1	5.25E-34	########	0.74	0.408	8.78E-30	2
Hsp90b1	6.19E-27	########	0.614	0.314	1.04E-22	2
5730508B0	2.19E-39	########	0.623	0.299	3.67E-35	2
Abracl	2.15E-23	########	0.879	0.606	3.59E-19	2
Rnf10	5.67E-34	########	0.704	0.398	9.48E-30	2
Pnkp	1.35E-19	########	0.867	0.649	2.26E-15	2
Fdps	2.07E-29	########	0.447	0.205	3.47E-25	2
Agps	4.98E-29	########	0.594	0.312	8.33E-25	2
Ndufs4	2.48E-31	########	0.766	0.433	4.15E-27	2
Usp5	7.24E-41	########	0.496	0.216	1.21E-36	2
Brd3	5.21E-30	########	0.66	0.356	8.71E-26	2
Pgam1	1.36E-17	########	0.893	0.714	2.28E-13	2
Usp39	8.72E-46	########	0.507	0.212	1.46E-41	2
Ubr2	8.18E-33	########	0.603	0.321	1.37E-28	2
Rpe	1.94E-36	########	0.538	0.256	3.24E-32	2
Anxa7	1.97E-39	########	0.626	0.31	3.30E-35	2
Plxnc1	2.71E-38	########	0.608	0.301	4.53E-34	2

Tex15	7.52E-39	########	0.314	0.095	1.26E-34	2
S1pr4	1.23E-39	########	0.672	0.345	2.05E-35	2
Cep19	1.04E-33	########	0.714	0.404	1.74E-29	2
Nt5c3	3.77E-30	########	0.654	0.367	6.30E-26	2
Mlst8	3.93E-52	########	0.403	0.129	6.58E-48	2
Slco4c1	6.91E-38	########	0.557	0.263	1.16E-33	2
Plin3	2.07E-36	########	0.637	0.32	3.46E-32	2
Cib2	4.42E-36	########	0.626	0.321	7.39E-32	2
Nedd4	1.28E-39	########	0.541	0.231	2.15E-35	2
Vimp	5.63E-23	########	0.43	0.226	9.42E-19	2
Cklf	2.18E-28	########	0.68	0.391	3.64E-24	2
Tbcb	1.70E-34	########	0.698	0.366	2.84E-30	2
Smg9	1.22E-43	########	0.484	0.19	2.04E-39	2
Serpinb6a	8.59E-50	########	0.533	0.211	1.44E-45	2
Cd81	2.36E-42	########	0.568	0.237	3.94E-38	2
Gas7	1.50E-38	########	0.521	0.231	2.51E-34	2
Rnaseh2c	1.04E-23	########	0.786	0.457	1.73E-19	2
Cmah	1.70E-30	########	0.654	0.351	2.84E-26	2
Wfdc17	1.20E-221	3.058025	0.915	0.298	2.00E-217	3
Ifitm1	7.47E-190	2.898285	0.879	0.333	1.25E-185	3
Ccl6	2.43E-203	2.467634	0.869	0.261	4.06E-199	3
Gm5483	2.70E-61	2.390475	0.467	0.158	4.52E-57	3
Stfa2l1	1.32E-74	2.355833	0.564	0.23	2.21E-70	3
Csf3r	1.20E-224	2.129935	0.967	0.779	2.00E-220	3
Retnlg	1.05E-175	2.081802	0.979	0.877	1.75E-171	3
Cxcr2	1.92E-244	2.016681	0.966	0.805	3.20E-240	3
Btg1	4.10E-192	1.87134	0.981	0.841	6.85E-188	3
Srgn	3.17E-176	1.866378	0.994	0.58	5.31E-172	3
Tpd52	2.09E-204	1.795531	0.979	0.712	3.49E-200	3
Clec4d	1.61E-191	1.772712	0.956	0.299	2.69E-187	3
Ifi27l2a	1.91E-22	1.75241	0.324	0.167	3.20E-18	3
Il1r2	5.96E-128	1.739466	0.899	0.68	9.97E-124	3
Slpi	9.72E-191	1.735026	0.979	0.921	1.63E-186	3
Fxyd5	1.93E-207	1.704067	0.988	0.899	3.24E-203	3
Grina	1.32E-216	1.680128	0.979	0.821	2.20E-212	3
Pla2g7	1.09E-174	1.676043	0.85	0.239	1.83E-170	3
Selplg	7.05E-204	1.673791	0.945	0.785	1.18E-199	3
G0s2	1.96E-17	1.657489	0.724	0.805	3.27E-13	3
Rnf149	4.80E-159	1.640051	0.943	0.618	8.04E-155	3
Sell	1.30E-195	1.614373	0.936	0.817	2.18E-191	3
Cd300ld	9.99E-176	1.611543	0.829	0.296	1.67E-171	3
Picalm	1.44E-181	1.605715	0.942	0.733	2.41E-177	3
Hdc	3.79E-167	1.583547	0.968	0.87	6.35E-163	3
Acod1	2.41E-66	1.570716	0.532	0.172	4.04E-62	3
Trim30b	2.35E-160	1.556441	0.825	0.416	3.93E-156	3
Txnip	4.66E-142	1.550173	0.899	0.745	7.81E-138	3
Mcl1	1.16E-187	1.537795	0.996	0.96	1.94E-183	3
Marcks	5.87E-136	1.519195	0.821	0.393	9.83E-132	3
Amica1	4.18E-122	1.516748	0.598	0.14	7.00E-118	3
Ccr1	4.86E-172	1.512899	0.946	0.752	8.13E-168	3
Trem1	1.43E-82	1.503497	0.754	0.5	2.39E-78	3
Stk17b	3.72E-171	1.502514	0.935	0.802	6.23E-167	3

Lmnb1	1.30E-97	1.484118	0.892	0.679	2.18E-93	3
Entpd1	2.67E-111	1.476686	0.745	0.386	4.47E-107	3
Fbxl5	2.59E-119	1.470547	0.826	0.73	4.33E-115	3
Slc7a11	1.62E-55	1.464852	0.55	0.239	2.72E-51	3
Malat1	1.98E-212	1.456191	1	0.992	3.31E-208	3
AY036118	8.05E-166	1.451835	0.998	0.987	1.35E-161	3
Cyp4f18	7.14E-144	1.442056	0.798	0.36	1.19E-139	3
Arg2	1.06E-140	1.432551	0.774	0.237	1.77E-136	3
Ifitm2	3.94E-180	1.425267	0.99	0.969	6.58E-176	3
Gcnt2	3.46E-116	1.419872	0.743	0.411	5.79E-112	3
Tgfbi	2.18E-146	1.413017	0.874	0.51	3.64E-142	3
S100a11	1.16E-246	1.411541	0.998	0.991	1.93E-242	3
Cebpb	1.21E-127	1.408437	0.973	0.876	2.02E-123	3
Cd14	1.55E-60	1.403221	0.595	0.253	2.59E-56	3
Emilin2	1.17E-161	1.403135	0.887	0.437	1.95E-157	3
Thbs1	9.80E-16	1.397836	0.287	0.154	1.64E-11	3
Gm42418	3.58E-157	1.387838	1	1	6.00E-153	3
Gda	1.06E-182	1.385266	0.951	0.902	1.77E-178	3
S100a6	7.11E-149	1.383553	0.992	0.899	1.19E-144	3
Lars2	6.17E-161	1.362045	0.994	0.974	1.03E-156	3
Gpcpd1	3.16E-87	1.355199	0.712	0.492	5.28E-83	3
Plek	1.74E-141	1.348476	0.961	0.735	2.91E-137	3
Ddx6	1.78E-125	1.344738	0.879	0.811	2.98E-121	3
Mxd1	2.27E-171	1.33654	0.967	0.903	3.81E-167	3
Map1lc3b	4.66E-183	1.32461	0.969	0.951	7.80E-179	3
Fgl2	6.73E-83	1.322608	0.543	0.191	1.13E-78	3
Cd33	2.32E-144	1.314305	0.891	0.742	3.88E-140	3
Neat1	4.18E-124	1.313204	0.965	0.914	7.00E-120	3
Fth1	6.85E-150	1.309603	0.998	0.997	1.15E-145	3
H2-D1	5.99E-208	1.299598	0.996	0.975	1.00E-203	3
Samhd1	1.12E-143	1.294388	0.893	0.743	1.87E-139	3
Il1rap	1.22E-63	1.292861	0.559	0.274	2.03E-59	3
Lilr4b	4.39E-124	1.286984	0.901	0.719	7.35E-120	3
Slc16a3	2.81E-124	1.285832	0.891	0.786	4.70E-120	3
Isg15	9.86E-30	1.285122	0.364	0.179	1.65E-25	3
Ptprc	3.03E-174	1.274928	0.984	0.943	5.07E-170	3
Adam8	2.37E-109	1.270927	0.825	0.58	3.97E-105	3
Rdh12	3.99E-83	1.249023	0.77	0.64	6.67E-79	3
Il1b	2.31E-112	1.247223	0.744	0.211	3.87E-108	3
Zyx	1.58E-140	1.24521	0.921	0.902	2.64E-136	3
7-Mar	1.94E-96	1.232789	0.803	0.648	3.24E-92	3
Stfa2	7.77E-10	1.224617	0.144	0.066	1.30E-05	3
Cd84	3.41E-90	1.216995	0.745	0.579	5.71E-86	3
Asprv1	9.33E-48	1.213684	0.677	0.489	1.56E-43	3
2810474O1	9.81E-100	1.211146	0.87	0.761	1.64E-95	3
Msrb1	3.30E-179	1.205286	0.984	0.96	5.53E-175	3
Rgs2	2.12E-67	1.204899	0.734	0.674	3.54E-63	3
Antxr2	1.98E-68	1.204286	0.725	0.553	3.32E-64	3
Sorl1	1.05E-155	1.190409	0.946	0.921	1.76E-151	3
Dusp1	6.42E-119	1.181428	0.795	0.296	1.07E-114	3
Lcp1	8.26E-210	1.172055	0.994	0.97	1.38E-205	3
Ier5	7.93E-87	1.169031	0.782	0.572	1.33E-82	3

Litaf	6.77E-131	1.166636	0.932	0.804	1.13E-126	3
Il13ra1	2.86E-112	1.154184	0.596	0.145	4.79E-108	3
Jund	8.81E-108	1.14634	0.904	0.697	1.47E-103	3
Gadd45a	1.10E-69	1.144128	0.707	0.521	1.84E-65	3
Trib1	1.29E-31	1.138613	0.496	0.333	2.16E-27	3
Lsp1	6.94E-132	1.137368	0.93	0.921	1.16E-127	3
Cytip	1.52E-92	1.136382	0.772	0.582	2.55E-88	3
Dhrs9	1.05E-97	1.125701	0.723	0.421	1.76E-93	3
Steap4	6.83E-62	1.125517	0.371	0.075	1.14E-57	3
Tyrobp	5.47E-208	1.119973	0.997	0.978	9.15E-204	3
Slc15a3	3.43E-111	1.116949	0.707	0.244	5.73E-107	3
H2-Q10	4.49E-75	1.105103	0.474	0.12	7.51E-71	3
Tmcc1	8.07E-93	1.102992	0.845	0.841	1.35E-88	3
Snap23	1.25E-98	1.096953	0.787	0.596	2.09E-94	3
Clec4e	6.27E-135	1.095971	0.95	0.653	1.05E-130	3
Myadm	1.41E-85	1.091039	0.633	0.291	2.37E-81	3
Gm5150	2.87E-97	1.089612	0.5	0.09	4.80E-93	3
Ets2	2.78E-36	1.088193	0.556	0.365	4.65E-32	3
Ssh2	2.83E-74	1.083984	0.747	0.675	4.73E-70	3
Klf2	1.65E-102	1.08371	0.92	0.849	2.76E-98	3
Prr13	1.06E-92	1.074499	0.866	0.872	1.78E-88	3
Cpd	2.98E-69	1.074496	0.723	0.648	4.98E-65	3
Iqgap1	3.05E-161	1.064543	0.978	0.969	5.10E-157	3
Fpr1	1.72E-90	1.054525	0.771	0.456	2.88E-86	3
Kctd12	1.12E-79	1.042001	0.793	0.686	1.88E-75	3
Sirpb1b	2.46E-109	1.041865	0.546	0.099	4.12E-105	3
Cd44	1.58E-121	1.038216	0.971	0.938	2.64E-117	3
Nlrp3	2.68E-76	1.037901	0.59	0.173	4.49E-72	3
Tlr13	3.90E-91	1.032927	0.608	0.232	6.53E-87	3
Cd300lf	2.95E-89	1.030714	0.836	0.817	4.93E-85	3
Nfkbiz	8.99E-39	1.030403	0.563	0.339	1.50E-34	3
Btg2	1.53E-96	1.029815	0.851	0.693	2.56E-92	3
Tnfaip2	7.58E-78	1.029813	0.869	0.839	1.27E-73	3
Ypel3	3.71E-93	1.029384	0.799	0.657	6.21E-89	3
Il1f9	7.40E-89	1.028933	0.753	0.509	1.24E-84	3
Zfp36l1	3.40E-70	1.02206	0.572	0.203	5.70E-66	3
Sat1	2.04E-126	1.021804	0.937	0.929	3.41E-122	3
Lilrb4a	2.91E-100	1.020666	0.893	0.821	4.88E-96	3
Ndel1	8.92E-55	1.020336	0.721	0.64	1.49E-50	3
Fyb	9.13E-87	1.017641	0.82	0.747	1.53E-82	3
Oasl2	6.95E-73	1.016065	0.452	0.117	1.16E-68	3
Ankrd33b	3.78E-94	1.015367	0.611	0.209	6.33E-90	3
Pim1	3.50E-65	1.013963	0.819	0.689	5.86E-61	3
Trim30a	5.26E-67	1.012657	0.665	0.473	8.80E-63	3
Rtp4	3.22E-63	1.007798	0.404	0.108	5.39E-59	3
Bcl2l11	1.65E-16	1.006196	0.368	0.25	2.76E-12	3
Smox	7.02E-67	1.004656	0.52	0.188	1.17E-62	3
Mmp9	9.01E-110	1.001535	0.982	0.701	1.51E-105	3
Dmxl2	3.72E-62	1.00093	0.679	0.519	6.23E-58	3
Klf6	2.90E-89	1.000036	0.901	0.867	4.85E-85	3
Slfn2	5.29E-112	########	0.966	0.964	8.85E-108	3
Sgms2	1.43E-26	########	0.676	0.724	2.39E-22	3

Ctsd	1.76E-139	########	0.964	0.943	2.94E-135	3
Lyst	3.41E-85	########	0.859	0.847	5.70E-81	3
Gk	9.54E-72	########	0.592	0.309	1.60E-67	3
Slfn1	1.04E-65	########	0.803	0.721	1.74E-61	3
St8sia4	1.74E-72	########	0.431	0.098	2.91E-68	3
Igfbp6	8.82E-47	########	0.35	0.093	1.48E-42	3
Samsn1	1.36E-47	########	0.893	0.915	2.28E-43	3
Hist1h2bc	5.63E-41	########	0.548	0.382	9.43E-37	3
Prok2	9.17E-39	########	0.431	0.197	1.53E-34	3
Klf3	5.26E-53	########	0.663	0.553	8.80E-49	3
Tpm4	1.04E-83	########	0.707	0.36	1.74E-79	3
Rab8b	5.08E-67	########	0.806	0.789	8.49E-63	3
Emb	1.05E-102	########	0.888	0.823	1.76E-98	3
Junb	1.43E-117	########	0.922	0.569	2.40E-113	3
Cd53	4.96E-93	########	0.947	0.927	8.29E-89	3
Stx11	4.02E-54	########	0.708	0.639	6.73E-50	3
Mefv	3.17E-18	########	0.4	0.294	5.30E-14	3
Gm26740	9.75E-61	########	0.721	0.646	1.63E-56	3
Upp1	7.18E-52	########	0.591	0.335	1.20E-47	3
Adipor1	9.03E-98	########	0.868	0.9	1.51E-93	3
Mrpl33	3.64E-126	########	0.936	0.93	6.08E-122	3
Rab11fip1	1.07E-48	########	0.638	0.473	1.79E-44	3
Timp2	2.50E-77	########	0.764	0.564	4.18E-73	3
Fosl2	1.43E-68	########	0.701	0.472	2.39E-64	3
Lamp2	1.12E-53	########	0.873	0.888	1.88E-49	3
Adgre5	2.14E-28	########	0.674	0.71	3.58E-24	3
Akap13	3.91E-73	########	0.825	0.864	6.55E-69	3
Syk	5.07E-65	########	0.802	0.783	8.49E-61	3
Tspan13	5.98E-77	########	0.431	0.086	1.00E-72	3
Tsc22d3	8.46E-20	########	0.611	0.669	1.42E-15	3
Pten	1.69E-80	########	0.818	0.862	2.83E-76	3
Itgal	2.55E-47	########	0.7	0.664	4.26E-43	3
Slfn4	1.06E-45	########	0.79	0.696	1.78E-41	3
Slc40a1	2.40E-22	########	0.597	0.638	4.01E-18	3
Ptafr	9.07E-31	########	0.626	0.564	1.52E-26	3
Pnrc1	3.21E-56	########	0.825	0.799	5.37E-52	3
Hcar2	4.07E-48	########	0.44	0.149	6.81E-44	3
Mmp8	2.81E-50	########	0.804	0.503	4.70E-46	3
Ube2b	1.67E-104	########	0.881	0.888	2.79E-100	3
Por	1.27E-62	########	0.69	0.536	2.12E-58	3
Notch2	2.06E-63	########	0.626	0.402	3.44E-59	3
Rsrp1	1.72E-107	########	0.943	0.94	2.88E-103	3
Pde4b	5.98E-24	########	0.389	0.224	1.00E-19	3
Ccpg1	8.78E-58	########	0.65	0.514	1.47E-53	3
Zfp36	4.91E-92	########	0.868	0.708	8.22E-88	3
Bst1	1.85E-69	########	0.695	0.398	3.10E-65	3
Adam19	1.68E-74	########	0.458	0.124	2.81E-70	3
Trps1	3.19E-62	########	0.481	0.171	5.33E-58	3
Vmp1	5.22E-41	########	0.575	0.404	8.74E-37	3
Hif1a	1.59E-35	########	0.576	0.462	2.67E-31	3
Rnf11	1.31E-45	########	0.623	0.535	2.20E-41	3
C5ar1	3.76E-89	########	0.882	0.825	6.29E-85	3

Rara	6.08E-64	########	0.544	0.254	1.02E-59	3
Kdm7a	8.39E-57	########	0.799	0.791	1.40E-52	3
Sdcbp	8.68E-99	########	0.922	0.924	1.45E-94	3
Sfxn5	5.36E-68	########	0.496	0.192	8.97E-64	3
Dazap2	1.20E-86	########	0.87	0.861	2.01E-82	3
Atp6v1g1	6.59E-112	########	0.909	0.945	1.10E-107	3
Glipr1	5.96E-58	########	0.652	0.491	9.96E-54	3
Zfp36l2	6.29E-37	########	0.702	0.684	1.05E-32	3
Hdac4	2.73E-39	########	0.553	0.418	4.57E-35	3
Themis2	6.31E-55	########	0.513	0.252	1.06E-50	3
Nfkbia	4.10E-09	########	0.818	0.925	6.86E-05	3
Sh3glb1	1.73E-80	########	0.868	0.759	2.90E-76	3
Sema4a	2.99E-46	########	0.696	0.698	5.00E-42	3
Cd244	1.32E-61	########	0.35	0.066	2.21E-57	3
Whsc1l1	1.72E-45	########	0.737	0.769	2.88E-41	3
Lpcat2	9.16E-61	########	0.736	0.68	1.53E-56	3
Neurl3	6.43E-46	########	0.617	0.446	1.08E-41	3
Sepn1	6.91E-33	########	0.455	0.274	1.16E-28	3
1600010M(	2.08E-53	########	0.505	0.256	3.48E-49	3
Fcgr3	8.94E-82	########	0.869	0.88	1.50E-77	3
Tlr2	2.01E-42	########	0.514	0.271	3.37E-38	3
Fam65b	5.34E-44	########	0.747	0.776	8.93E-40	3
Anxa2	9.96E-106	########	0.937	0.948	1.67E-101	3
Csf2rb	2.65E-57	########	0.786	0.794	4.44E-53	3
Sirpb1c	4.57E-60	########	0.406	0.097	7.64E-56	3
Ncf2	1.78E-93	########	0.926	0.915	2.98E-89	3
Hacd4	7.13E-41	########	0.658	0.598	1.19E-36	3
Pilra	1.09E-67	########	0.835	0.853	1.82E-63	3
Diaph1	8.72E-71	########	0.864	0.888	1.46E-66	3
Ogt	4.96E-43	########	0.661	0.589	8.30E-39	3
Lrg1	7.82E-69	########	0.941	0.905	1.31E-64	3
Dennd4a	6.75E-16	########	0.518	0.516	1.13E-11	3
Cmip	3.18E-64	########	0.858	0.868	5.32E-60	3
Nudt4	1.64E-59	########	0.821	0.866	2.75E-55	3
Il4ra	1.67E-35	########	0.533	0.397	2.79E-31	3
Basp1	8.88E-29	########	0.378	0.167	1.49E-24	3
Casp4	2.51E-23	########	0.539	0.478	4.20E-19	3
Ube2h	1.84E-34	########	0.621	0.571	3.08E-30	3
Dgat1	7.37E-34	########	0.746	0.811	1.23E-29	3
Rsad2	3.60E-23	########	0.223	0.078	6.02E-19	3
Taldo1	2.97E-107	########	0.959	0.953	4.97E-103	3
Trpm2	2.29E-52	########	0.505	0.26	3.83E-48	3
Fgr	6.71E-57	########	0.785	0.808	1.12E-52	3
Il17ra	2.37E-47	########	0.707	0.669	3.96E-43	3
Actg1	2.42E-101	########	0.987	0.98	4.05E-97	3
Lyn	4.90E-71	########	0.879	0.895	8.19E-67	3
Ly6e	1.20E-33	########	0.599	0.456	2.00E-29	3
Osbpl9	3.69E-43	########	0.711	0.739	6.18E-39	3
2310001H1	3.70E-34	########	0.678	0.687	6.18E-30	3
Clec2d	7.26E-13	########	0.579	0.651	1.21E-08	3
Lilra6	1.10E-53	########	0.467	0.197	1.85E-49	3
Socs3	9.17E-53	########	0.478	0.178	1.53E-48	3

Usp32	2.48E-46	########	0.526	0.321	4.15E-42	3
Tbc1d14	1.64E-48	########	0.474	0.24	2.75E-44	3
Chd7	2.99E-18	########	0.575	0.585	5.00E-14	3
D1Ertd622e	5.75E-23	########	0.608	0.663	9.63E-19	3
Xpo6	7.69E-41	########	0.533	0.373	1.29E-36	3
Gm17619	6.30E-46	########	0.386	0.146	1.05E-41	3
Snx20	2.63E-31	########	0.696	0.726	4.40E-27	3
Rnasel	5.93E-32	########	0.57	0.476	9.92E-28	3
Klra2	1.44E-60	########	0.455	0.156	2.40E-56	3
Nlrp12	1.29E-56	########	0.42	0.15	2.15E-52	3
Pira2	1.02E-43	########	0.61	0.443	1.71E-39	3
Cd300lb	6.84E-35	########	0.555	0.427	1.14E-30	3
Slc11a1	2.08E-45	########	0.485	0.226	3.48E-41	3
Tnfrsf1a	2.11E-41	########	0.732	0.74	3.53E-37	3
Tnfrsf1b	2.24E-29	########	0.427	0.235	3.75E-25	3
Gcnt1	9.97E-57	########	0.387	0.11	1.67E-52	3
Tarm1	6.68E-37	########	0.527	0.362	1.12E-32	3
Snx18	3.05E-31	########	0.69	0.706	5.10E-27	3
Gm2a	1.35E-35	########	0.5	0.287	2.26E-31	3
Atg3	1.49E-48	########	0.786	0.864	2.49E-44	3
St3gal6	2.36E-52	########	0.335	0.071	3.95E-48	3
Il18rap	5.05E-32	########	0.671	0.662	8.45E-28	3
Igf1r	1.22E-15	########	0.653	0.729	2.05E-11	3
Eif4ebp1	7.56E-41	########	0.728	0.781	1.26E-36	3
H2-T23	1.53E-30	########	0.669	0.713	2.56E-26	3
Ehd1	6.96E-48	########	0.792	0.801	1.16E-43	3
App	5.49E-35	########	0.758	0.806	9.18E-31	3
Dck	1.39E-26	########	0.483	0.376	2.32E-22	3
Mov10	4.45E-55	########	0.402	0.132	7.44E-51	3
Fmnl1	1.77E-52	########	0.756	0.729	2.96E-48	3
Fabp5	1.01E-69	########	0.661	0.339	1.69E-65	3
Cmtm6	2.90E-38	########	0.703	0.724	4.85E-34	3
Ltb	3.44E-26	########	0.677	0.675	5.76E-22	3
Tmem71	4.17E-32	########	0.56	0.443	6.99E-28	3
Ptpn1	1.63E-43	########	0.792	0.883	2.73E-39	3
Tgoln1	3.73E-07	########	0.431	0.443	########	3
Trim30d	2.92E-27	########	0.292	0.12	4.89E-23	3
Alox5ap	7.05E-104	########	0.986	0.959	1.18E-99	3
Svil	2.96E-34	########	0.73	0.804	4.95E-30	3
Isg20	7.46E-19	########	0.344	0.222	1.25E-14	3
Trp53inp1	2.94E-26	########	0.502	0.409	4.92E-22	3
Ccnl1	4.04E-08	########	0.587	0.69	########	3
Oas3	9.17E-36	########	0.454	0.254	1.53E-31	3
4833407H1	2.41E-43	########	0.399	0.158	4.03E-39	3
AB124611	6.06E-41	########	0.764	0.843	1.01E-36	3
Kdm6b	4.89E-36	########	0.734	0.721	8.18E-32	3
Clk1	1.49E-22	########	0.642	0.682	2.50E-18	3
Trim12c	1.27E-17	########	0.542	0.558	2.13E-13	3
Cyth4	6.68E-27	########	0.572	0.502	1.12E-22	3
Gng2	2.89E-41	########	0.65	0.525	4.83E-37	3
Rab7	4.92E-41	########	0.851	0.899	8.23E-37	3
Zcchc6	5.87E-28	########	0.718	0.765	9.82E-24	3

Bach1	2.56E-24	########	0.589	0.572	4.29E-20	3
Jmjd1c	5.77E-20	########	0.604	0.607	9.66E-16	3
Slc6a6	3.25E-29	########	0.694	0.687	5.44E-25	3
Eif1	1.37E-79	########	0.995	0.99	2.29E-75	3
Gbp2	1.50E-35	########	0.23	0.048	2.51E-31	3
Cfp	7.07E-32	########	0.694	0.698	1.18E-27	3
Rb1cc1	8.91E-20	########	0.646	0.704	1.49E-15	3
Fas	3.78E-40	########	0.442	0.214	6.32E-36	3
Gabarap	1.12E-106	########	0.977	0.981	1.88E-102	3
Ifngr1	2.68E-19	########	0.622	0.643	4.48E-15	3
Cers6	2.64E-30	########	0.567	0.512	4.41E-26	3
Gm14548	5.86E-34	########	0.497	0.31	9.81E-30	3
Ddx60	7.17E-26	########	0.325	0.164	1.20E-21	3
Slc2a3	2.39E-21	########	0.679	0.758	3.99E-17	3
Ifitm3	1.39E-47	########	0.948	0.922	2.32E-43	3
Mirt1	2.93E-18	########	0.523	0.496	4.90E-14	3
A530064D(	3.55E-25	########	0.468	0.346	5.94E-21	3
Apbb1ip	1.25E-46	########	0.813	0.887	2.09E-42	3
Cyth1	1.10E-13	########	0.421	0.366	1.83E-09	3
Gsr	9.59E-91	########	0.973	0.965	1.60E-86	3
Pxn	5.11E-31	########	0.707	0.777	8.55E-27	3
Hbp1	1.69E-27	########	0.574	0.511	2.82E-23	3
Ddx5	3.04E-58	########	0.983	0.988	5.08E-54	3
Il6ra	4.99E-23	########	0.535	0.462	8.34E-19	3
Gadd45b	1.75E-08	########	0.495	0.497	########	3
Nfe2l2	1.38E-09	########	0.685	0.822	2.30E-05	3
Fam32a	1.42E-27	########	0.701	0.798	2.38E-23	3
Cdkn1b	6.80E-21	########	0.656	0.739	1.14E-16	3
Fam129a	1.45E-27	########	0.679	0.716	2.43E-23	3
Zeb2	1.65E-28	########	0.406	0.189	2.76E-24	3
Braf	5.19E-17	########	0.48	0.451	8.69E-13	3
Celf2	2.03E-30	########	0.752	0.833	3.39E-26	3
Arrdc3	3.65E-41	########	0.33	0.113	6.11E-37	3
Rassf3	2.45E-20	########	0.597	0.623	4.09E-16	3
Fam134b	6.99E-26	########	0.544	0.452	1.17E-21	3
Rab32	4.67E-18	########	0.518	0.492	7.82E-14	3
Plaur	1.91E-12	########	0.78	0.867	3.19E-08	3
Il1rn	3.88E-32	########	0.506	0.316	6.49E-28	3
Cd300a	9.72E-22	########	0.536	0.48	1.63E-17	3
Fcgr4	3.49E-22	########	0.547	0.497	5.84E-18	3
4932438A1	3.88E-08	########	0.588	0.697	########	3
Sephs2	4.65E-21	########	0.526	0.493	7.78E-17	3
Myd88	1.87E-22	########	0.549	0.518	3.14E-18	3
Wipf1	9.01E-23	########	0.636	0.667	1.51E-18	3
Pag1	1.56E-25	########	0.651	0.698	2.62E-21	3
Rassf5	2.84E-20	########	0.611	0.671	4.76E-16	3
Plin2	2.72E-08	########	0.604	0.707	########	3
Dusp6	1.28E-25	########	0.367	0.201	2.13E-21	3
Slc2a6	4.61E-57	########	0.383	0.094	7.72E-53	3
Shisa5	4.45E-43	########	0.576	0.353	7.45E-39	3
Bcl10	4.00E-32	########	0.737	0.829	6.69E-28	3
Rhov	2.84E-18	########	0.276	0.14	4.75E-14	3

Hectd1	5.53E-13	########	0.631	0.757	9.26E-09	3
Kpna4	1.74E-16	########	0.562	0.573	2.91E-12	3
Zfp106	5.50E-17	########	0.519	0.503	9.20E-13	3
Rbms1	4.29E-35	########	0.787	0.87	7.17E-31	3
Sub1	6.05E-21	########	0.812	0.914	1.01E-16	3
Irf1	2.55E-15	########	0.511	0.475	4.27E-11	3
Stat3	6.89E-18	########	0.689	0.786	1.15E-13	3
Preb	3.12E-15	########	0.438	0.391	5.22E-11	3
Spi1	5.28E-61	########	0.922	0.929	8.84E-57	3
Tgm2	8.57E-18	########	0.269	0.125	1.43E-13	3
Raf1	2.39E-24	########	0.587	0.574	4.00E-20	3
R3hdm4	7.96E-31	########	0.764	0.867	1.33E-26	3
Laptm5	1.38E-59	########	0.934	0.951	2.31E-55	3
Nedd9	3.82E-18	########	0.505	0.461	6.39E-14	3
Iscu	1.87E-20	########	0.586	0.613	3.13E-16	3
Cdc42se1	4.31E-25	########	0.692	0.772	7.20E-21	3
Srsf5	6.76E-17	########	0.795	0.896	1.13E-12	3
Prex1	1.94E-22	########	0.509	0.439	3.24E-18	3
Sh2d3c	1.43E-17	########	0.464	0.4	2.40E-13	3
Map2k3	3.59E-15	########	0.552	0.579	6.00E-11	3
Stk40	1.10E-19	########	0.441	0.343	1.83E-15	3
Dusp16	7.41E-17	########	0.222	0.098	1.24E-12	3
Supt4a	2.41E-37	########	0.776	0.856	4.03E-33	3
Mcemp1	4.43E-56	########	0.91	0.908	7.41E-52	3
Nabp1	6.20E-21	########	0.488	0.373	1.04E-16	3
Xylt1	5.87E-24	########	0.453	0.336	9.82E-20	3
Klhl2	3.06E-35	########	0.497	0.319	5.12E-31	3
Il10rb	1.83E-12	########	0.511	0.54	3.06E-08	3
Pi16	7.70E-17	########	0.603	0.683	1.29E-12	3
Prr5l	1.66E-47	########	0.24	0.028	2.77E-43	3
Hcls1	4.51E-47	########	0.844	0.899	7.55E-43	3
Sirpa	6.96E-22	########	0.677	0.762	1.16E-17	3
Nampt	5.99E-13	########	0.469	0.459	1.00E-08	3
Dennd5a	1.26E-14	########	0.568	0.621	2.11E-10	3
Ppt1	3.06E-08	########	0.558	0.67	########	3
Rnf144a	6.91E-16	########	0.585	0.658	1.16E-11	3
Birc3	2.58E-12	########	0.427	0.379	4.32E-08	3
Klf7	6.16E-07	########	0.591	0.72	########	3
Gsap	2.07E-13	########	0.447	0.41	3.47E-09	3
Irf2	5.10E-20	########	0.656	0.742	8.54E-16	3
Nadk	1.08E-20	########	0.725	0.828	1.80E-16	3
Bmx	4.00E-17	########	0.522	0.455	6.70E-13	3
N4bp1	2.95E-14	########	0.68	0.807	4.93E-10	3
Wbp1l	2.35E-14	########	0.407	0.344	3.94E-10	3
Cd52	4.39E-75	########	0.993	0.978	7.35E-71	3
Pbxip1	7.71E-24	########	0.67	0.677	1.29E-19	3
Ddx3x	2.06E-08	########	0.618	0.709	########	3
Sgms1	1.40E-28	########	0.427	0.253	2.35E-24	3
Carhsp1	1.19E-13	########	0.579	0.641	1.99E-09	3
Ggt5	2.94E-27	########	0.381	0.217	4.92E-23	3
Sesn2	4.25E-34	########	0.342	0.144	7.11E-30	3
Glipr2	3.78E-23	########	0.737	0.848	6.33E-19	3

Phyh	2.03E-15	########	0.463	0.425	3.39E-11	3
Mrgpra2b	8.22E-10	########	0.585	0.638	1.38E-05	3
Tbc1d15	3.35E-18	########	0.522	0.502	5.60E-14	3
Kmt2e	2.42E-11	########	0.637	0.789	4.04E-07	3
Cd101	1.04E-41	########	0.284	0.071	1.73E-37	3
Arpc3	9.53E-68	########	0.984	0.967	1.59E-63	3
Zfand5	8.82E-14	########	0.613	0.697	1.47E-09	3
Slc2a1	1.20E-22	########	0.335	0.181	2.01E-18	3
Snx10	2.79E-09	########	0.514	0.553	4.68E-05	3
Creg1	2.72E-24	########	0.635	0.633	4.55E-20	3
Coro1a	2.90E-83	########	0.986	0.959	4.86E-79	3
Iqsec1	9.14E-20	########	0.566	0.545	1.53E-15	3
Tnfaip3	5.73E-10	########	0.283	0.18	9.58E-06	3
Baz2b	5.07E-15	########	0.664	0.737	8.48E-11	3
Ccl4	1.47E-12	########	0.215	0.104	2.47E-08	3
Hmha1	4.25E-19	########	0.702	0.826	7.11E-15	3
Ankrd11	1.90E-14	########	0.672	0.78	3.17E-10	3
Mvp	9.09E-17	########	0.437	0.365	1.52E-12	3
Ube2d3	2.20E-47	########	0.913	0.954	3.68E-43	3
Ifi204	1.20E-26	########	0.24	0.073	2.01E-22	3
Rap1b	6.07E-36	########	0.905	0.945	1.02E-31	3
Ttc7	8.28E-10	########	0.432	0.434	1.39E-05	3
Mpeg1	4.87E-25	########	0.479	0.316	8.15E-21	3
Plk3	6.51E-13	########	0.243	0.134	1.09E-08	3
Ikbkap	1.16E-14	########	0.426	0.38	1.94E-10	3
Pygl	4.55E-42	########	0.873	0.898	7.62E-38	3
Skap2	1.02E-40	########	0.851	0.901	1.71E-36	3
Arhgap25	1.79E-16	########	0.523	0.532	2.99E-12	3
Rbm47	3.55E-31	########	0.374	0.18	5.93E-27	3
Klhdc4	4.50E-10	########	0.467	0.467	7.53E-06	3
Furin	9.70E-18	########	0.505	0.462	1.62E-13	3
Ppp1r3d	1.44E-23	########	0.348	0.204	2.42E-19	3
Rasa3	8.21E-13	########	0.363	0.291	1.37E-08	3
Tlr4	4.28E-10	########	0.293	0.223	7.16E-06	3
Siglece	3.56E-14	########	0.655	0.763	5.96E-10	3
Gpr35	5.43E-20	########	0.206	0.071	9.08E-16	3
Ncor1	2.27E-20	########	0.743	0.865	3.80E-16	3
Lcp2	1.38E-10	########	0.564	0.648	2.30E-06	3
Atp1a3	3.03E-16	########	0.375	0.286	5.07E-12	3
Igsf6	3.37E-09	########	0.858	0.909	5.64E-05	3
Trim12a	4.34E-13	########	0.539	0.573	7.27E-09	3
Lrrc25	2.47E-19	########	0.385	0.258	4.14E-15	3
Ptp4a1	9.61E-10	########	0.549	0.62	1.61E-05	3
Nfam1	1.87E-15	########	0.656	0.784	3.12E-11	3
Pkm	1.33E-60	########	0.974	0.981	2.23E-56	3
Tspo	3.69E-44	########	0.958	0.951	6.18E-40	3
Fuca1	2.06E-12	########	0.593	0.688	3.45E-08	3
Ifngr2	2.30E-13	########	0.536	0.572	3.84E-09	3
Jak1	7.05E-27	########	0.765	0.86	1.18E-22	3
Ptbp3	7.86E-35	########	0.86	0.927	1.32E-30	3
Arrdc4	5.14E-12	########	0.262	0.162	8.60E-08	3
Txn1	2.69E-44	########	0.958	0.976	4.50E-40	3

Atxn10	5.04E-19	########	0.721	0.847	8.43E-15	3
Abtb1	2.78E-15	########	0.632	0.737	4.65E-11	3
Atf7ip	4.26E-11	########	0.55	0.592	7.13E-07	3
Hmgcl	2.27E-10	########	0.445	0.459	3.80E-06	3
Rin3	3.89E-14	########	0.649	0.766	6.51E-10	3
Ell2	7.35E-15	########	0.244	0.129	1.23E-10	3
Smpdl3a	1.18E-10	########	0.619	0.74	1.98E-06	3
Atp2b1	6.26E-08	########	0.546	0.601	########	3
Hcst	1.21E-24	########	0.796	0.884	2.02E-20	3
Trim25	6.82E-14	########	0.395	0.318	1.14E-09	3
Pfkfb4	5.77E-09	########	0.539	0.619	9.66E-05	3
Rnf13	2.12E-13	########	0.493	0.476	3.55E-09	3
Mtus1	3.35E-08	########	0.584	0.732	########	3
Chp1	1.09E-09	########	0.57	0.672	1.83E-05	3
Gm9733	5.20E-09	########	0.333	0.286	8.70E-05	3
Fpr2	1.71E-23	########	0.829	0.759	2.87E-19	3
Serp1	5.85E-20	########	0.736	0.855	9.79E-16	3
Smap2	1.22E-15	########	0.641	0.727	2.04E-11	3
Cfap43	1.33E-07	########	0.438	0.463	########	3
Esd	5.93E-14	########	0.701	0.828	9.92E-10	3
Ctnnbip1	1.28E-11	########	0.36	0.274	2.14E-07	3
Tnfsf14	3.91E-16	########	0.288	0.175	6.55E-12	3
Eid1	9.30E-08	########	0.503	0.57	########	3
Pirb	1.97E-28	########	0.879	0.885	3.29E-24	3
Capn1	9.60E-12	########	0.595	0.718	1.61E-07	3
Max	3.30E-13	########	0.612	0.738	5.52E-09	3
Lpin2	2.92E-06	########	0.243	0.194	########	3
Abca1	1.43E-12	########	0.166	0.07	2.40E-08	3
Fos	1.48E-28	########	0.45	0.237	2.48E-24	3
Atp11b	1.87E-12	########	0.674	0.818	3.13E-08	3
Ankrd44	2.94E-13	########	0.583	0.655	4.93E-09	3
Fcer1g	5.17E-56	########	0.993	0.976	8.66E-52	3
Slc35a5	1.85E-09	########	0.467	0.49	3.10E-05	3
H3f3a	1.88E-107	########	0.998	0.993	3.15E-103	3
Cap1	2.38E-34	########	0.855	0.908	3.99E-30	3
Ppp1r3b	1.79E-19	########	0.334	0.204	3.00E-15	3
Chmp4b	4.42E-19	########	0.755	0.879	7.40E-15	3
Arid5a	2.12E-07	########	0.359	0.344	########	3
5031439G(	1.74E-17	########	0.378	0.268	2.91E-13	3
Ddit3	3.78E-11	########	0.28	0.194	6.33E-07	3
Rasgrp4	1.40E-12	########	0.588	0.656	2.34E-08	3
Crebrf	2.82E-11	########	0.423	0.382	4.71E-07	3
Nfat5	4.55E-09	########	0.407	0.384	7.61E-05	3
Arhgap24	3.23E-33	########	0.298	0.106	5.40E-29	3
Actb	2.28E-93	########	1	0.997	3.81E-89	3
Osgin1	2.19E-14	########	0.279	0.169	3.67E-10	3
Tle3	4.56E-08	########	0.454	0.47	########	3
Bcl6	7.88E-13	########	0.396	0.337	1.32E-08	3
Adrb2	3.79E-11	########	0.393	0.337	6.34E-07	3
Oaz2	2.39E-07	########	0.394	0.402	########	3
Hexim1	3.24E-07	########	0.321	0.271	########	3
Rhog	1.07E-20	########	0.773	0.876	1.79E-16	3

Alcam	7.78E-13	########	0.304	0.202	1.30E-08	3
Midn	6.67E-07	########	0.485	0.541	########	3
Atp6v1b2	1.80E-07	########	0.579	0.701	########	3
Crlf2	1.06E-09	########	0.606	0.71	1.78E-05	3
Actn1	3.92E-22	########	0.783	0.862	6.56E-18	3
Bcl3	2.40E-09	########	0.332	0.267	4.01E-05	3
Tmem154	8.41E-09	########	0.577	0.682	########	3
Ogfrl1	1.06E-12	########	0.744	0.849	1.78E-08	3
Pdlim7	3.32E-08	########	0.464	0.48	########	3
Cass4	1.88E-17	########	0.184	0.065	3.15E-13	3
Inpp5d	1.26E-06	########	0.477	0.539	########	3
Map3k5	1.50E-08	########	0.324	0.283	########	3
Tax1bp1	2.83E-19	########	0.841	0.916	4.73E-15	3
Klra17	1.82E-15	########	0.277	0.167	3.05E-11	3
Mctp2	5.94E-14	########	0.339	0.25	9.94E-10	3
Rac1	5.01E-24	########	0.845	0.922	8.38E-20	3
Ppp1r18	1.13E-21	########	0.736	0.851	1.90E-17	3
Aqp9	1.08E-38	########	0.266	0.064	1.81E-34	3
Timm10b	2.02E-07	########	0.643	0.796	########	3
Msn	1.05E-31	########	0.882	0.952	1.76E-27	3
Csnk1e	6.40E-10	########	0.363	0.309	1.07E-05	3
Map3k3	3.45E-07	########	0.394	0.4	########	3
Hip1	1.13E-20	########	0.293	0.153	1.89E-16	3
Irf9	2.87E-12	########	0.363	0.281	4.80E-08	3
B430306N(	7.45E-11	########	0.641	0.761	1.25E-06	3
Phf21a	7.53E-09	########	0.343	0.296	########	3
Ninj1	2.84E-15	########	0.426	0.304	4.74E-11	3
Sipa1l1	3.79E-08	########	0.307	0.255	########	3
Mbnl2	5.49E-08	########	0.474	0.501	########	3
Msl1	3.87E-07	########	0.578	0.711	########	3
Son	1.98E-31	########	0.914	0.943	3.32E-27	3
Lnpep	1.72E-08	########	0.438	0.445	########	3
Slfn5	3.26E-17	########	0.128	0.031	5.46E-13	3
Capza1	3.90E-29	########	0.821	0.899	6.53E-25	3
Dusp5	8.06E-10	########	0.194	0.103	1.35E-05	3
Jun	4.78E-07	########	0.337	0.276	########	3
Sema4d	3.45E-14	########	0.461	0.394	5.77E-10	3
Lbr	4.43E-17	########	0.789	0.882	7.41E-13	3
Ubc	1.26E-15	########	0.926	0.973	2.11E-11	3
Ubb	3.79E-40	########	0.984	0.99	6.35E-36	3
Cxcl2	7.08E-35	########	0.452	0.193	1.18E-30	3
Arhgap31	1.05E-10	########	0.25	0.169	1.76E-06	3
Ttyh3	5.17E-09	########	0.373	0.34	8.65E-05	3
Rgs3	1.79E-14	########	0.364	0.266	3.00E-10	3
Pttg1	1.24E-08	########	0.255	0.182	########	3
Dhrs7	7.06E-24	########	0.859	0.912	1.18E-19	3
Apobr	1.12E-08	########	0.68	0.797	########	3
Rab7b	5.93E-14	########	0.152	0.054	9.93E-10	3
Arap1	1.30E-06	########	0.393	0.391	########	3
Fbxo31	8.30E-16	########	0.258	0.144	1.39E-11	3
Irf7	2.02E-09	########	0.253	0.174	3.38E-05	3
Gpsm3	2.07E-28	########	0.866	0.918	3.46E-24	3

Tgif1	6.26E-09	########	0.19	0.104	########	3
Pnpla2	1.67E-06	########	0.331	0.307	########	3
Gng12	1.36E-11	########	0.65	0.767	2.28E-07	3
4732465J0	4.39E-25	########	0.197	0.054	7.35E-21	3
Mbd2	1.24E-13	########	0.727	0.851	2.07E-09	3
Kdm5b	2.49E-07	########	0.417	0.408	########	3
Cd82	5.44E-07	########	0.487	0.547	########	3
Cdc42	3.95E-50	########	0.968	0.975	6.60E-46	3
Cxcl3	1.16E-11	########	0.197	0.093	1.94E-07	3
Klhl24	7.69E-09	########	0.37	0.328	########	3
Prkcd	1.19E-09	########	0.651	0.793	1.99E-05	3
Ftl1	2.59E-98	########	0.997	0.992	4.34E-94	3
Trpv2	2.67E-17	########	0.265	0.136	4.46E-13	3
Anxa11	2.55E-15	########	0.764	0.856	4.27E-11	3
Egr1	6.91E-13	########	0.24	0.121	1.16E-08	3
Arhgap30	3.77E-08	########	0.686	0.84	########	3
Riok3	1.04E-08	########	0.691	0.847	########	3
Tnfrsf23	1.42E-06	########	0.203	0.135	########	3
Cd68	7.51E-15	########	0.37	0.246	1.26E-10	3
Agpat9	1.31E-08	########	0.262	0.187	########	3
Gm20406	5.72E-14	########	0.238	0.131	9.58E-10	3
Mta3	2.92E-08	########	0.201	0.133	########	3
Rnd1	1.41E-06	########	0.316	0.281	########	3
Zbp1	4.82E-07	########	0.191	0.127	########	3
Trf	1.38E-06	########	0.234	0.172	########	3
Lrrfip1	2.53E-12	########	0.753	0.885	4.24E-08	3
Il15	2.27E-09	########	0.223	0.146	3.81E-05	3
Pet100	4.37E-11	########	0.7	0.837	7.31E-07	3
Grb2	1.06E-17	########	0.79	0.9	1.77E-13	3
Myo1f	5.45E-10	########	0.704	0.86	9.12E-06	3
Camk1d	4.25E-24	########	0.239	0.083	7.12E-20	3
Kcnj2	4.98E-09	########	0.232	0.156	8.33E-05	3
Slc44a1	2.73E-19	########	0.197	0.072	4.57E-15	3
Cd9	5.88E-12	########	0.925	0.964	9.84E-08	3
A130014A(	5.07E-11	########	0.253	0.167	8.49E-07	3
Dock2	5.35E-07	########	0.641	0.764	########	3
Mpzl3	3.61E-25	########	0.182	0.043	6.04E-21	3
Myl12b	7.67E-27	########	0.914	0.935	1.28E-22	3
Dopey2	9.70E-07	########	0.292	0.248	########	3
Gmfg	6.99E-29	########	0.956	0.945	1.17E-24	3
Csf1r	3.92E-23	########	0.273	0.105	6.56E-19	3
Card19	1.28E-22	########	0.749	0.847	2.15E-18	3
P2ry13	2.18E-13	########	0.195	0.094	3.66E-09	3
Tmem156	3.44E-10	########	0.253	0.171	5.76E-06	3
Rab11fip4	1.70E-13	########	0.193	0.094	2.84E-09	3
Fndc3b	1.63E-07	########	0.225	0.154	########	3
Gnai2	7.69E-36	########	0.962	0.972	1.29E-31	3
Cxcr1	1.17E-19	########	0.104	0.011	1.95E-15	3
Apobec1	1.22E-14	########	0.276	0.165	2.04E-10	3
Metrnl	7.63E-20	########	0.282	0.131	1.28E-15	3
Ptgs2os2	2.12E-12	########	0.12	0.038	3.55E-08	3
Tlr6	4.74E-17	########	0.195	0.077	7.93E-13	3

Itgb2	6.04E-20	########	0.874	0.922	1.01E-15	3
Sycp2	1.22E-22	########	0.171	0.043	2.04E-18	3
Dennd3	3.27E-10	########	0.244	0.161	5.46E-06	3
Ppp1r16b	9.06E-15	########	0.126	0.036	1.52E-10	3
Ostf1	2.81E-27	########	0.965	0.969	4.70E-23	3
Arpc5	6.60E-22	########	0.936	0.943	1.10E-17	3
Gnai3	2.91E-06	0.306676	0.691	0.845	########	3
Chsy1	6.67E-08	########	0.218	0.153	########	3
Vsir	4.96E-14	########	0.795	0.892	8.29E-10	3
Glrx	1.73E-08	########	0.788	0.89	########	3
Sema6b	3.97E-11	########	0.16	0.077	6.65E-07	3
Oasl1	7.96E-17	########	0.108	0.02	1.33E-12	3
Gad1-ps	2.23E-20	########	0.15	0.036	3.73E-16	3
Retn	9.55E-17	########	0.134	0.035	1.60E-12	3
Ccdc180	3.48E-07	########	0.267	0.209	########	3
Ucp2	1.69E-12	########	0.788	0.898	2.84E-08	3
Lfng	4.29E-07	########	0.225	0.157	########	3
Vps37b	1.80E-10	########	0.207	0.402	3.01E-06	3
Nr4a3	5.50E-08	########	0.116	0.051	########	3
B2m	1.12E-09	########	0.929	0.967	1.87E-05	3
Fam114a1	6.76E-10	########	0.186	0.105	1.13E-05	3
Cdc42ep2	1.25E-08	########	0.228	0.147	########	3
Vasp	4.25E-15	########	0.896	0.938	7.12E-11	3
Ier3	2.29E-14	########	0.546	0.475	3.83E-10	3
Acta2	1.14E-09	########	0.183	0.102	1.90E-05	3
Rufy4	2.35E-19	########	0.144	0.034	3.93E-15	3
5430427O1	4.88E-11	########	0.17	0.083	8.17E-07	3
Rbm39	9.71E-07	########	0.81	0.908	########	3
Ywhaz	8.61E-10	########	0.851	0.929	1.44E-05	3
H2-T24	1.26E-10	########	0.207	0.121	2.10E-06	3
Ggt1	5.07E-11	########	0.215	0.116	8.48E-07	3
Tmbim6	1.49E-15	########	0.898	0.942	2.49E-11	3
Emd	3.57E-07	########	0.301	0.528	########	3
Sbno2	1.46E-06	########	0.184	0.127	########	3
Cnnm2	2.94E-08	########	0.11	0.049	########	3
Slfn8	8.76E-11	########	0.138	0.058	1.47E-06	3
Psap	5.20E-45	########	0.817	0.841	8.71E-41	3
Arfip1	8.67E-10	########	0.237	0.154	1.45E-05	3

Table S3 Gene lists used for module scoring of neutrophil maturation

Neutrophil maturation

Retnlg
Ccl6
S100a6
Clec4d
Prr13
Cebpb
Slpi
S100a11
Btg1
Cxcr2
Fth1
Grina
Mmp8
Fxyd5
Msrb1
H2-D1
Anxa2
Mmp9
Ftl1
Map1lc3b
Tmcc1
Sat1
Cyp4f18
Junb
Mxd1
Stk17b
Ypel3
Selplg
Il1f9
Dusp1
Slc16a3
Ccr1
Rdh12
Clec4e
Arg2
Cd300ld
Amica1
Ctsd
Gda
Hacd4
Timp2
Fpr1
Ifi27l2a
Slc7a11
Stfa2l1
Il1b
Asprv1
Cxcl2

Gm5483
Ifitm1

Table S4 Gene lists used for module scoring of pathway activation

Jak/Stat sig	FoxO signa	HIF1 signal	TGFb signa	WNT signa	Hippo signa	IL1 signalin	TNF signaling
Il6	Atg12	Aldoa	Cdkn2b	Bambi	Yap1	Il1a	Akt1
Il10	Atm	Angpt1	Crebbp	Ccn4	Wwtr1	Il1b	Akt2
Il10rb	Bcl2l11	Angpt2	E2f4	Ccnd1	Ppp2ca	Il1r1	Akt3
Il10ra	Bcl6	Angpt4	E3f5	Ccnd2	Ppp2cb	Il1rap	Atf2
Jak1	Bnip3	Bcl2	Ep300	Ccnd3	Ppp2r2c	Myd88	Atf4
Jak2	Cat	Cdkn1a	Rbl1	Csnk1e	Ppp2r1a	Irak1	Atf6b
Jak3	Ccnb1	Cdkn1b	Smad2	Csnk2a1	Ppp2r2d	Irak2	Birc2
Tyk2	Ccnb2	Edn1	Smad3	Csnk2a2	Ppp2r2a	Irak4	Birc3
Stat1	Ccnb3	Egf	Smad4	Csnk2b	Ppp2r2b	Map3k7	Casp3
Stat2	Ccnd1	Eno1	Sp1	Ctnnb1	Ppp2r1b	Traf6	Casp7
Stat3	Ccnd2	Eno1b	Tfdp1	Dvl1	Rassf6	Tab1	Casp8
Stat4	Ccng2	Eno2	Tgfb1	Dvl2	Wtip	Tab2	Cebpb
Stat5a	Cdkn1a	Eno3	Tgfb2	Dvl3	Ajuba	Ikbkb	Chuk
Stat5b	Cdkn1b	Eno4	Tgfb3	Fosl1	Limd1	Ikbke	Creb1
Stat6	Cdkn2b	Epo	Tgfbr1	Frat1	Trp73	Nfkb1	Creb3
Irf9	Cdkn2d	Flt1	Tgfbr2	Frat2	Tead1	Map2k1	Creb3l1
Crebbp	Csnk1e	Gapdh	Thbs1	Fzd1	Tead2	Jnk	Creb3l2
Ep300	Fasl	Gapdh-ps1	Fstl1	Fzd10	Tead3	Mapk9	Creb3l3
Bcl2	Fbxo25	Hk1		Fzd2	Tead4	Mapk8	Creb3l4
Mcl1	Fbxo32	Hk2		Fzd3		Mapk11	Creb5
Bcl2l1	Foxo1	Hk3		Fzd4		Mapk13	Dab2ip
Pim1	Foxo3	Hkdc1		Fzd5		Mapk14	Dnm1l
Myc	Foxo4	Hmox1		Fzd6		Mapk3	Fadd
Ccnd1	Foxo6	Ldha		Fzd7		Mapk4	Fos
Ccnd2	G6pc	Nos2		Fzd8		Jun	Ifnb1
Ccnd3	G6pc2	Nos3		Fzd9		Fos	Ikbkb
Cdkn1a	G6pc3	Nppa		Jun		Jdp2	Ikbkg
Aox1	Gabarap	Pdk1		Lef1		Atf2	Irf1
Aox2	Gabarapl1	Pfkfb3		Lrp5		Atf4	Itch
Aox3	Gabarapl2	Pfkl		Lrp6		Atf5	Jun
Aox4	Gadd45a	Pgk1		Mmp7			Lta
Gfap	Gadd45b	Serpine1		Myc			Map2k1
	Gadd45g	Slc2a1		Peg12			Map2k3
	Grm1	Tek		Porcn			Map2k4
	Il10	Tfrc		Ppard			Map2k6
	Il6	Timp1		Rspo1			Map2k7
	Il7r	Trf		Rspo2			Map3k14
	Klf2	Vegfa		Rspo3			Map3k5
	Mapk10			Rspo4			Map3k7
	Mapk11			Tcf7			Map3k8
	Mapk12			Tcf7l1			Mapk1
	Mapk13			Tcf7l2			Mapk10
	Mapk14			Wnt1			Mapk11
	Mapk8			Wnt10a			Mapk12
	Mapk9			Wnt10b			Mapk13
	Pck1			Wnt11			Mapk14
	Pck2			Wnt16			Mapk3
	Plk1			Wnt2			Mapk8

Plk2
Plk3
Plk4
Prkaa1
Prkaa2
Prkab1
Prkab2
Prkag2
Prkag3
Prmt1
Rag1
Rag2
Rbl2
S1pr1
S1pr4
Slc2a4
Sod2
Stat3
Stk11
Tnfsf10

Wnt2b
Wnt3
Wnt3a
Wnt4
Wnt5a
Wnt6
Wnt7a
Wnt7b
Wnt8a
Wnt8b
Wnt9a
Wnt9b
Wnt5b
Axin1
Axin2

Mapk9
Mlkl
Nfkb1
Nfkbia
Pgam5
Pik3ca
Pik3cb
Pik3cd
Pik3r1
Pik3r2
Pik3r3
Rela
Ripk1
Ripk3
Rps6ka4
Rps6ka5
Tab1
Tab2
Tab3
Tnf
Tnfrsf1a
Tnfrsf1b
Tradd
Traf1
Traf2
Traf3
Traf5

Table S5 Gene lists used for module scoring of angiogenesis pathways

Angiogenes	Maturation	Stalk cell	Tip cell
Acvrl1	Cdh5	Hlx1	Kdr
Aggf1	Ddit3	Ackr1	Dll4
Amot	S1pr1	Aqp1	Pdgfb
Ang	Ankrd17	C1qtnf9	Flt4
Angptl3	Lyl1	Cd36	Angpt2
Angptl4	Mmp2	Csrp2	Apln
Atpif1	Reck	Ehd4	Nrp1
Btg1	Acvrl1	Fbln5	Nrp2
C1galt1		Hspb1	Efnb2
Canx		Ligp1	Robo4
Cdh13		Il6st	Unc5b
Chrna7		Jam2	Plxnd1
Col4a2		Lgals3	Igf2
Col4a3		Lrg1	Igfbp4
Egf		Meox2	Adm
Emcn		Plscr2	Ankrd37
Epgn		Sdpr	C1qtnf6
Erap1		Selp	Cldn5
Foxo4		Spint2	Col4a1
Htatip2		Tgfbi	Cotl1
Il17f		Tgm2	Ednrb
Il18		Tmem176a	Fscn1
Il8		Tmem252	Gpihbp1
Myh9		Tspan7	Hspg2
Ncl		Vwf	Igfbp3
Nf1		Flt1	Inhbb
Notch4			Jup
Nppb			Kcne3
Npr1			Kcnj8
Pf4			Lama4
Plg			Lamb1
Pml			Lxn
Prok2			Marcksl1
Rhob			Mcam
Rnh1			Mest
Robo4			N4
Runx1			Nid2
Scg2			Notch4
Serpinf1			Plod1
Shh			Pmepa1
Sphk1			Ptn
Spink5			Ramp3
Stab1			Rbp1
Tgfb2			Rgcc
Thy1			Rhoc
Tnfsf12			Trp53ill
Tnni3			Mmp2
Vegfa			Mmp9

Mmp14
Cxcl12
